# Cyanobacterial diversity held in microbial biological resource centers as a biotechnological asset: the case study of the newly established LEGE culture collection

**DOI:** 10.1007/s10811-017-1369-y

**Published:** 2018-01-06

**Authors:** Vitor Ramos, João Morais, Raquel Castelo-Branco, Ângela Pinheiro, Joana Martins, Ana Regueiras, Ana L. Pereira, Viviana R. Lopes, Bárbara Frazão, Dina Gomes, Cristiana Moreira, Maria Sofia Costa, Sébastien Brûle, Silvia Faustino, Rosário Martins, Martin Saker, Joana Osswald, Pedro N. Leão, Vitor M. Vasconcelos

**Affiliations:** 1 Interdisciplinary Centre of Marine and Environmental Research (CIIMAR/CIMAR), Terminal de Cruzeiros do Porto de Leixões, University of Porto, 4450-208 Matosinhos, Portugal; 2 Faculdade de Ciências, Universidade do Porto, Rua do Campo Alegre, Edifício FC4, 4169-007 Porto, Portugal; 3 Nanotechnology and Functional Materials, Department of Engineering Sciences, Uppsala University, Box 534, 751 21 Uppsala, Sweden; 4 IPMA-Portuguese Institute of Sea and Atmosphere, Rua Alfredo Magalhães Ramalho, 6, 1495-006 Lisbon, Portugal; 5 ICBAS-Instituto de Ciências Biomédicas Abel Salazar, Universidade do Porto, Rua de Jorge Viterbo Ferreira 228, 4050-313 Porto, Portugal; 6 Master 2 Biotechnologie, Université de Bretagne-Sud, BP 92116, 56000 Lorient/Vannes, France; 7 Laboratory of Algae Cultivation and Bioprospection, Federal Amapá University (UNIFAP), Rodovia JK, km 2, Macapá, Amapá Brazil; 8 Health and Environment Research Centre, School of Health, Polytechnic Institute of Porto, Rua Dr. António Bernardino de Almeida, 400, 4200-072 Porto, Portugal; 9 Alpha Environmental Solutions, P.O. Box 37977, Dubai, United Arab Emirates

**Keywords:** Cyanobacteria, Strains, Biodiversity, Chemodiversity, Culture collection, Biological resource centers

## Abstract

Cyanobacteria are a well-known source of bioproducts which renders culturable strains a valuable resource for biotechnology purposes. We describe here the establishment of a cyanobacterial culture collection (CC) and present the first version of the strain catalog and its online database (http://lege.ciimar.up.pt/). The LEGE CC holds 386 strains, mainly collected in coastal (48%), estuarine (11%), and fresh (34%) water bodies, for the most part from Portugal (84%). By following the most recent taxonomic classification, LEGE CC strains were classified into at least 46 genera from six orders (41% belong to the Synechococcales), several of them are unique among the phylogenetic diversity of the cyanobacteria. For all strains, primary data were obtained and secondary data were surveyed and reviewed, which can be reached through the strain sheets either in the catalog or in the online database. An overview on the notable biodiversity of LEGE CC strains is showcased, including a searchable phylogenetic tree and images for all strains. With this work, 80% of the LEGE CC strains have now their 16S rRNA gene sequences deposited in GenBank. Also, based in primary data, it is demonstrated that several LEGE CC strains are a promising source of extracellular polymeric substances (EPS). Through a review of previously published data, it is exposed that LEGE CC strains have the potential or actual capacity to produce a variety of biotechnologically interesting compounds, including common cyanotoxins or unprecedented bioactive molecules. Phylogenetic diversity of LEGE CC strains does not entirely reflect chemodiversity. Further bioprospecting should, therefore, account for strain specificity of the valuable cyanobacterial holdings of LEGE CC.

**Electronic supplementary material:**

The online version of this article (10.1007/s10811-017-1369-y) contains supplementary material, which is available to authorized users.

## Introduction

Microbial biological resource centers (mBRCs) are quality-managed culture collections that ensure the ex situ preservation of microorganisms, while providing public access to their microbial diversity (i.e., to live strains or to genomic DNA from these strains), to relevant data related to it (e.g., taxonomic identification, culture conditions, ecophysiological features, etc.), and also to expertise services such as training or consulting (Antunes et al. 2016). mBRCs are pivotal in underpinning the bioeconomy derived from microbial resources (Smith et al. 2014). In this particular case, cyanobacteria have been pointed out in the past few decades as one of the most promising groups of microorganisms for the discovery of natural compounds with pharmacological and other biotechnological applications (Margesin and Schinner 2001; Abed et al. 2009; Singh et al. 2011; Wijffels et al. 2013). For oncology drugs only, the pharmaceutical value of the estimated marine cyanobacteria diversity was evaluated in US$37.5–181.5 billion, in 2010 dollars (Erwin et al. 2010). One other relevant property of cyanobacteria with biotechnological interest is the production of extracellular polymeric substances (EPSs) (Abed et al. 2009; Pereira et al. 2011).

The Blue Biotechnology and Ecotoxicology (BBE) group, at CIIMAR, Portugal, has recently undertaken a process of organizing its cyanobacterial strains into a culture collection (acronym LEGE). It began as an in-house collection in 1991, when a number of strains from the colonial toxic cyanobacterium *Microcystis aeruginosa* were isolated from freshwater water bodies in Portugal (Vasconcelos et al. 1995). Since then, a good number of strains have been isolated and assessed in ecotoxicological studies or used for the discovery of biologically active compounds, the main research lines of the group. As a consequence, a considerable body of research (e.g., Vasconcelos et al. 1995; Martins et al. 2005, 2013; Leão et al. 2013b; Brito et al. 2015) emphasizes that several strains now deposited at the LEGE culture collection (CC) have the potential or actual capacity to produce a myriad of chemical compounds, including toxins or newly discovered bioactive molecules. Yet, most of the strains at BBE were kept independently by their isolators along these years, and were poorly characterized, named inconsistently or even unidentified.

For these reasons, a decision was made to characterize and organize all the strains (and their associated data) at BBE, and make publicly available this bioresource by establishing a culture collection in accordance to the Organisation for Economic Co-operation and Development (OECD 2007) and World Federation for culture collections (WFCC 2010) guidelines. In this work, we illustrate (1) the process followed to establish LEGE CC, and give an overview of the collection by presenting (2) the catalog of strains, (3) the online database (http://lege.ciimar.up.pt/), and by revealing (4) their phylogenetic diversity. Cyanobacterial strains in LEGE CC were (re-)identified using an approach combining morphological and phylogenetic data, as recommended by Komárek (2016), which confers added value to the collection. Likewise, based on some novel and existing data, we reviewed (5) biotechnologically relevant information from the strains, and make some (6) considerations on the relation between biodiversity and chemodiversity for the discovery of natural compounds from cyanobacterial strains. Altogether, the disclosed data from the strains makes LEGE CC a valuable resource for further bioprospecting, toxicological, and/or taxonomic studies.

## Materials and methods

**Fig. 1:**
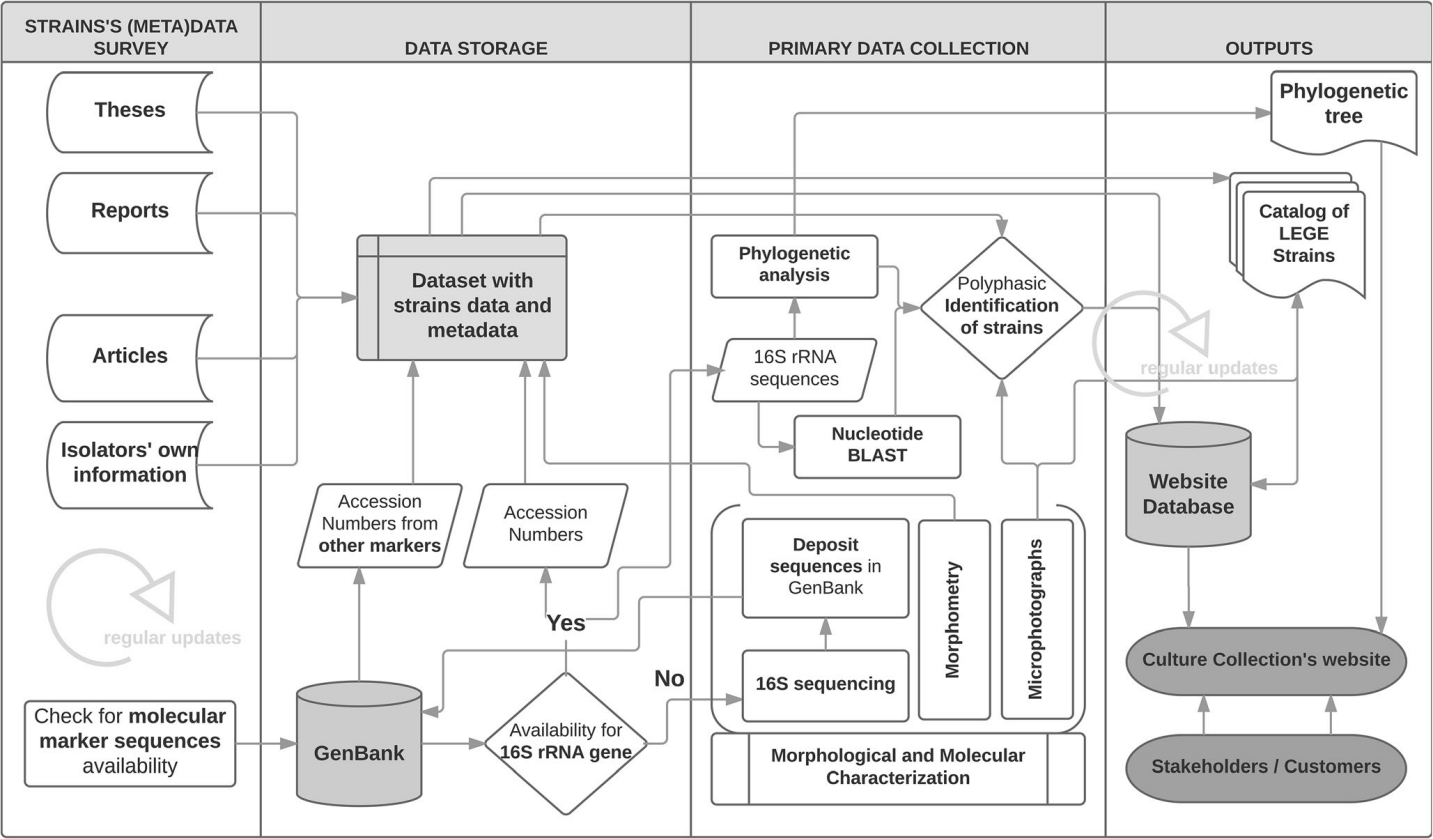
The workflow followed during the data gathering on the LEGE CC strains, the completed and expected outputs of the process and the planned updates (standard flowchart symbols were used). The LEGE CC website can be accessed at http://lege.ciimar.up.pt

Strain codes for all strains at BBE were standardized by using the acronym LEGE followed by a five-digit number. The workflow followed during the establishment of the culture collection is depicted in Fig. 1. The figure shows the processes and methods used for researching and collecting secondary data (e.g., information on secondary metabolite production; some nucleotide sequences) and for generating primary data from the strains (e.g., morphometry, microphotographs, most of the 16S rRNA gene sequences, and evaluation of EPS production). It also indicates the main outputs of these processes, which are presented in this study.

### Literature and data survey

Eighty-three strains had been previously published using other strain names/codes or identifications. For that reason, all existing synonyms for a same strain were considered during the literature search and data survey. Strain synonyms and references where they appear are provided in the catalog, in Online Resource 3. Strains having any type of data on natural products were recorded.

### Light microscopy and morphological characterization

Morphological characteristics of LEGE CC strains were examined and microphotographed using a Leica DMLB light microscope coupled to a Leica ICC50 HD digital camera (Leica Microsystems, Germany). Morphometric measurements were then performed using the image analysis software Leica Application Suite version 4.2.0 (Leica Microsystems). Strains were analyzed during the exponential phase of growth (i.e., 2- to 3-week old cultures, depending on the strain; culture conditions for each strain can be found in the catalog (Online Resource 3). Each quantifiable morphological character was measured at least 20 times, along different positions of the slide preparation. These include size of vegetative, specialized, or dormant cells, and of filaments or colonies.

Additionally, to evaluate the production of EPSs by the strains, early stationary-phase cultures (i.e., 3- to 5-week old cultures, depending on the strain) were stained with 0.5% Alcian Blue solutions (Sigma A-3157), prepared either in 50% ethanol (*v*/*v*) or in 1% acetic acid (*v*/*v*) (Di Pippo et al. 2013). Cultures were also negatively stained using India ink (Micheletti et al. 2008). Images were acquired using the abovementioned equipment and software.

When relevant, other qualitative morphological features and distinguishing traits were recorded (e.g., the shape and arrangement of cells or filaments, the color of the cultures, the presence or absence of sheaths, motility, the existence of constrictions at the cross-wall of filaments, and the formation of hormogonia and necridial cells).

### DNA extraction, PCR, and sequencing

Cells were harvested from log-phase cultures, and total genomic DNA (gDNA) of each strain was extracted using the commercial PureLink Genomic DNA Mini Kit (Invitrogen, USA), according the to the manufacturer’s instructions provided for Gram-negative bacteria. The DNA integrity was confirmed with agarose gel electrophoresis using GelRed (Biotium, USA) staining. Cyanobacteria-specific primers CYA-106F and CYA-785R (Nübel et al. 1997; Muhling et al. 2008) were used for the amplification of a portion of the 16S rRNA gene. PCR reactions were performed in a final volume of 20 μL containing 1× Green GoTaq Flexi Buffer, 2.5 mM MgCl_2_, 125.0 mM of each deoxynucleotide triphosphate, 1.0 μM of each primer, 0.5 U of GoTaq Flexi DNA Polymerase (Promega, USA), 10 mg mL^−1^ of bovine serum albumin (BSA), and 10–30 ng of template DNA, on a TProfessional Standard thermal cycler (Biometra, Germany). The PCR conditions were as follows: initial denaturation at 94 °C for 4 min, followed by 35 cycles of denaturation at 94 °C for 30 s, annealing at 52 °C for 30 s, and extension at 72 °C for 45 s, with a final extension step at 72 °C for 6 min. PCR products were separated with a 1.5% (*w*/*v*) agarose gel stained with GelRed (Biotium, USA) and DNA fragments with the expected size were excised from the gel and purified using the NucleoSpin Gel and PCR Clean-up Kit (Macherey-Nagel, Germany), according to the manufacturer’s instructions. Sequences were obtained by either directly sequencing the purified amplicons at GATC Biotech (Germany) or after cloning these into pGEM-T Easy vector (Promega, USA). In the latter case, vectors containing inserts were then transformed into *Escherichia coli* TOP10 chemically competent cells (Invitrogen, San Diego, CA). Plasmid DNA was isolated using NZY Miniprep kit (NYZtech, Portugal) and sequenced at GATC Biotech using M13 primers. All nucleotide sequences were manually inspected for quality and assembled for each strain using the Geneious (v8.1.8) software package (Biomatters Limited, New Zealand). Two hundred and twenty-four novel sequences associated with this study were deposited in the GenBank database under the accession numbers KU951663–KU951886.

### Phylogenetic analyses

Molecular-based analyses were conducted using the bioinformatics software package MEGA7 (Kumar et al. 2016). Two phylogenetic analyses based on 16S rRNA gene sequences were performed, one that reflects the overall cyanobacterial diversity present at LEGE CC and a second analysis that highlights the connection between such biodiversity and its associated chemodiversity. In both cases, sequences were aligned using the ClustalW algorithm (Thompson et al. 1994) and phylogenies were inferred by using the Maximum Likelihood (ML) method (Felsenstein 1981) based on the General Time Reversible model (Rodriguez et al. 1990), which was the nucleotide substitution model that best fitted the alignments data as evaluated by the corrected Akaike Information Criterion (Sugiura 1978). For both analyses also, a discrete Gamma distribution (+G) was used to model evolutionary rate differences among sites, while the rate variation model allowed for some sites to be evolutionarily invariable (+I). In the first case, the analysis involved 457 nucleotide sequences from LEGE CC strains and from relevant strains included in CyanoType v.1 (see Ramos et al. 2017). These include: (1) Type strains (T) of Type species (i.e. cyanobacterial strains that were used to describe a new genus); (2) strains known to have the same phylogenetic placement as the Type species (t), when the sequence from the latter is not available; (3) Reference strains (R) from the Bergey’s Manual of Systematic Bacteriology (Castenholz et al. 2001); and, (4) strains known to be included in the same phylogenetic cluster as the Reference strain (r), as mentioned in the Bergey’s Manual (Castenholz et al. 2001). There were a total of 563 positions in the final dataset. The tree was rooted with the outgroup *Chloroflexus aurantiacus* J-10-fl (NR_074263). In the second case, the phylogenetic analysis involved 165 nucleotide sequences from LEGE CC strains only and there were a total of 252 positions in the final dataset.

### Strain identification

By using data generated in this study, the taxonomic assignments of previously identified strains were reevaluated by an approach combining morphological and phylogenetic data. The most recent classification, recommendations and advice for the identification of cyanobacteria (Komárek et al. 2014; Dvořák et al. 2015; Komárek 2016) were followed, namely the adoption of a conservative approach (Dvořák et al. 2015; Komárek 2016). Previously unidentified strains were identified following the same procedures and principles. First, standard identification keys were used for the morphological-based identification of the strains (Komárek and Anagnostidis 1998, 2005; Komárek 2013). Then, each strain identification was compared with its phylogenetic placement (namely, assessing if the LEGE strain is closely related to any Type strain) and with the recent taxonomic classification proposed by Komárek et al. (2014), at low (i.e., genus) and high (i.e., order) taxonomic levels. If existing, taxonomic notes for a strain (e.g., incongruities between classification schemes) were added to the correspondent catalog sheet (Online Resource 3).

## Results and discussion

**Fig. 2:**
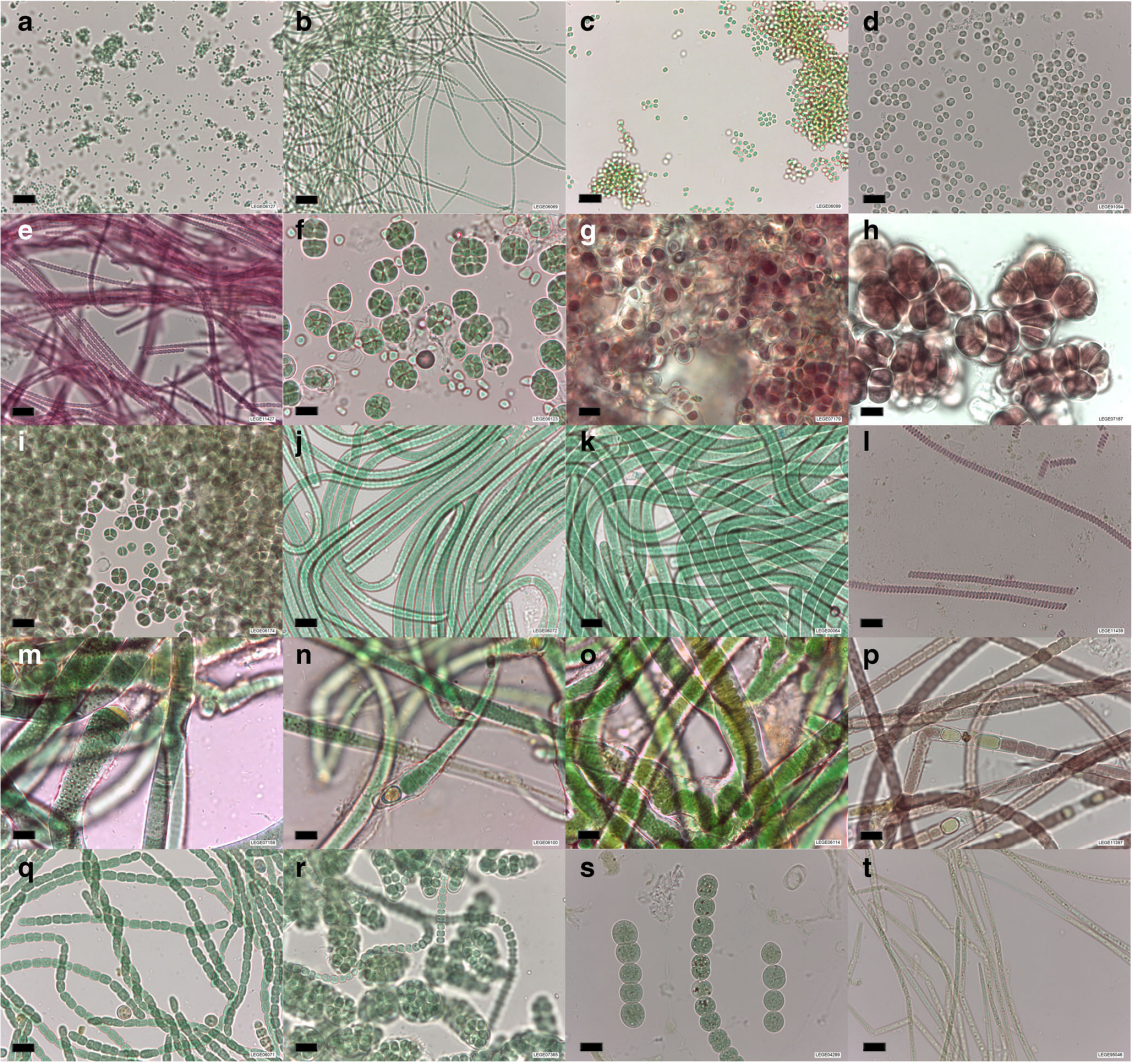
Example of morphological diversity among cyanobacterial strains from LEGE CC. Strains belong to the orders: a–b Synechococcales, c–e Chroococcales, f Chroococcidiopsidales, g–i Pleurocapsales, j–l Oscillatoriales, and m–t Nostocales. Identifications are as follows: a *Cyanobium* sp. LEGE 06127; b *Nodosilinea* sp. LEGE 06069; c *Synechocystis salina* LEGE 06099; d *Microcystis aeruginosa* LEGE 91094, a microcystin producer strain highly used in the literature (see also Fig. 3); e unidentified filamentous Chroococcales LEGE 11427; f *Gloeocapsopsis crepidinum* LEGE 06123; g *Hyella patelloides* LEGE 07179; h *Chroococcopsis* sp. LEGE 07187; *i Chroococcidiopsis* sp. LEGE 06174; j cf. *Oxynema acuminatum* LEGE 06072; k *Phormidium* sp. LEGE 00064; l cf. Spirulina sp. LEGE 11439; m *Rivularia* sp. LEGE 07159; n *Calothrix* sp. LEGE 06100; o *Plectonema* cf. *radiosum* LEGE 06114; p *Tolypothrix* sp. LEGE 11397; q *Nodularia* sp. LEGE 06071; r *Nostoc* sp. LEGE 07365; s *Dolichospermum flosaquae* LEGE 04289, an anatoxin-a producer strain; t *Cylindrospermopsis raciborskii* LEGE 95046, a non-cylindrospermopsin producer often used in the literature (see also Fig. 3). Scale bars represent 10 μm

Three hundred and eighty-six cyanobacterial strains are included in the first version of the catalog of LEGE CC (see Fig. 2 for a morphological overview). For each particular strain, primary and secondary data collected in this study (Fig. 1), such as species identification, origin, morphometric information, morphological description, and ecophysiological properties of the strain, microphotographs, literature references, synonyms for the strain, accession numbers for sequences, etc., can be retrieved in the corresponding catalog sheet (Online Resource 3) or be searched in the website database of the culture collection at http://lege.ciimar.up.pt.

### LEGE CC conditions

The LEGE CC is hosted in a new building, with modern facilities at CIIMAR, Matosinhos, Portugal. It includes cyanobacterial strains collected since 1991. LEGE CC strains are normally kept at 10–30 μmol photons m^−2^ s^−1^ under 12/12 h or 14/10 h light/dark cycles. The range of controlled temperature conditions at LEGE CC are 14, 19 (for most strains), and 25 °C. Strains are maintained by subculturing transfers (held every 6 months for most of the strains), but soon, a stock comprising the full collection will be cryopreserved and stored at − 150 °C (some strains are cryopreserved at − 80 °C; see also Rastoll et al. 2013). Despite the fact that axenicization of LEGE CC strains will be attempted in the future, currently, all are xenic, unicyanobacterial, and clonal.

### General statistics of holdings

**Fig. 3:**
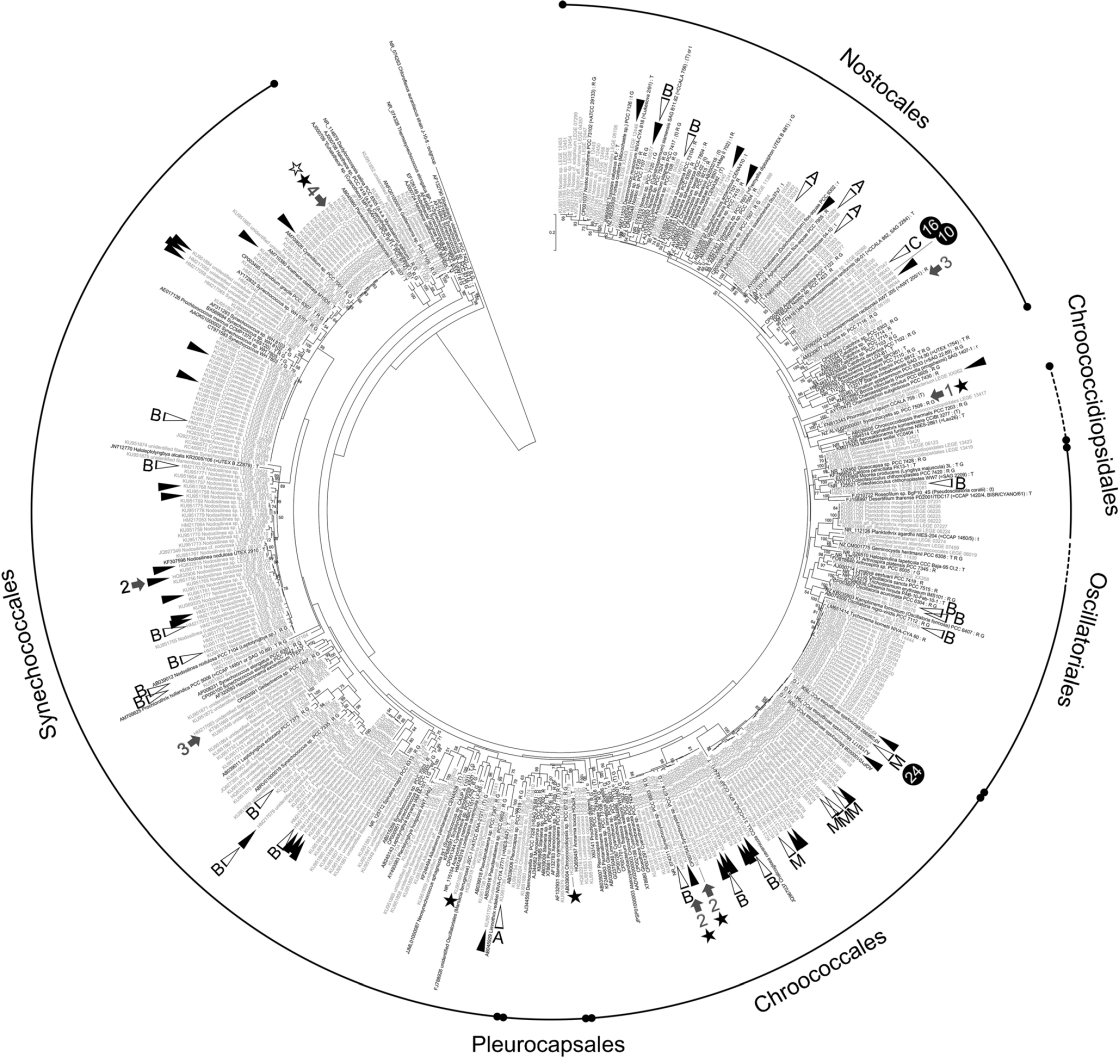
Circular ML tree (− lnl = 25,944.6863) of 16S rRNA gene sequences illustrating the phylogenetic diversity of LEGE CC strains (in gray), their placement at the order level, and some traits or information relevant for biotechnological purposes. One hundred and fifty-two sequences from reference material (Ramos et al. 2017) were included to disclose the cyanobacterial “Tree of Life” (T or t stand for type strains designated as representing type species, R or r for reference strains sensu Bergey’s Manual (Castenholz et al. 2001); and G for genome sequences available; see also Material and methods section for details). Accession numbers for all sequences are shown. Only bootstrap support values over 50% are given. Black arrowheads indicate strains capable of producing good amounts of EPSs. White arrowheads denote strains producing the following cyanotoxins, as demonstrated by analytical chemistry methods: A anatoxin-a, e.g., Osswald et al. (2009); B BMAA (Cianca et al. 2012); C cylindrospermopsin, e.g., Saker and Eaglesham (1999); and M microcystin, e.g., Vasconcelos et al. (1995). Arrows point to strains used to isolate and elucidate the structure of the following secondary metabolites: 1 hierridin B (Leão et al. 2013b), 2 portoamides (Leão et al. 2010), 3 bartolosides (Leão et al. 2015; Afonso et al. 2016), and 4 dehydroabietic acid (Costa et al. 2016). Black stars indicate strains having (or soon will have) their genome sequenced, and the white star stands for a strain that has a submitted patent application. Black circles and numbers within refer to highly used strains and to the number of times they appear in the literature, respectively

LEGE CC strains were isolated from samples mainly collected in Portugal (84%), including Madeira and Azores Islands. There are also strains from South (5%) and North (2%) America, Africa (3%), other European countries (1%), Oceania (1%), Antarctica (1%), and Asia (one strain). In relation to the habitat, LEGE CC strains were mainly collected from aquatic environments, including marine (46%), freshwater (34%), brackish (11%), and hypersaline (2%) environments, while some strains are of terrestrial origin (3%). Concerning taxonomy, LEGE CC strains are distributed by the orders Synechococcales (41%), Chroococcales (17%), Nostocales (17%), Oscillatoriales (8%), Pleurocapsales (2%), and Chroococcidiopsidales (2%) (see also Fig. 3). One-hundred and eleven LEGE CC strains are identified at the species level, 205 at the genus, while 70 strains remain unidentified at the genus level. Of course, the ever-changing nature of taxonomy causes identification to change over time, and thus, these numbers are expected to change in the next versions of the catalog. Three-hundred and seven LEGE CC strains (80% of the entire collection) have now their 16S rRNA gene sequences deposited in GenBank, which contrast with the 110 (28%) sequences from LEGE CC strains that existed before this study (see Online Resource 1).

**Fig. 4:**
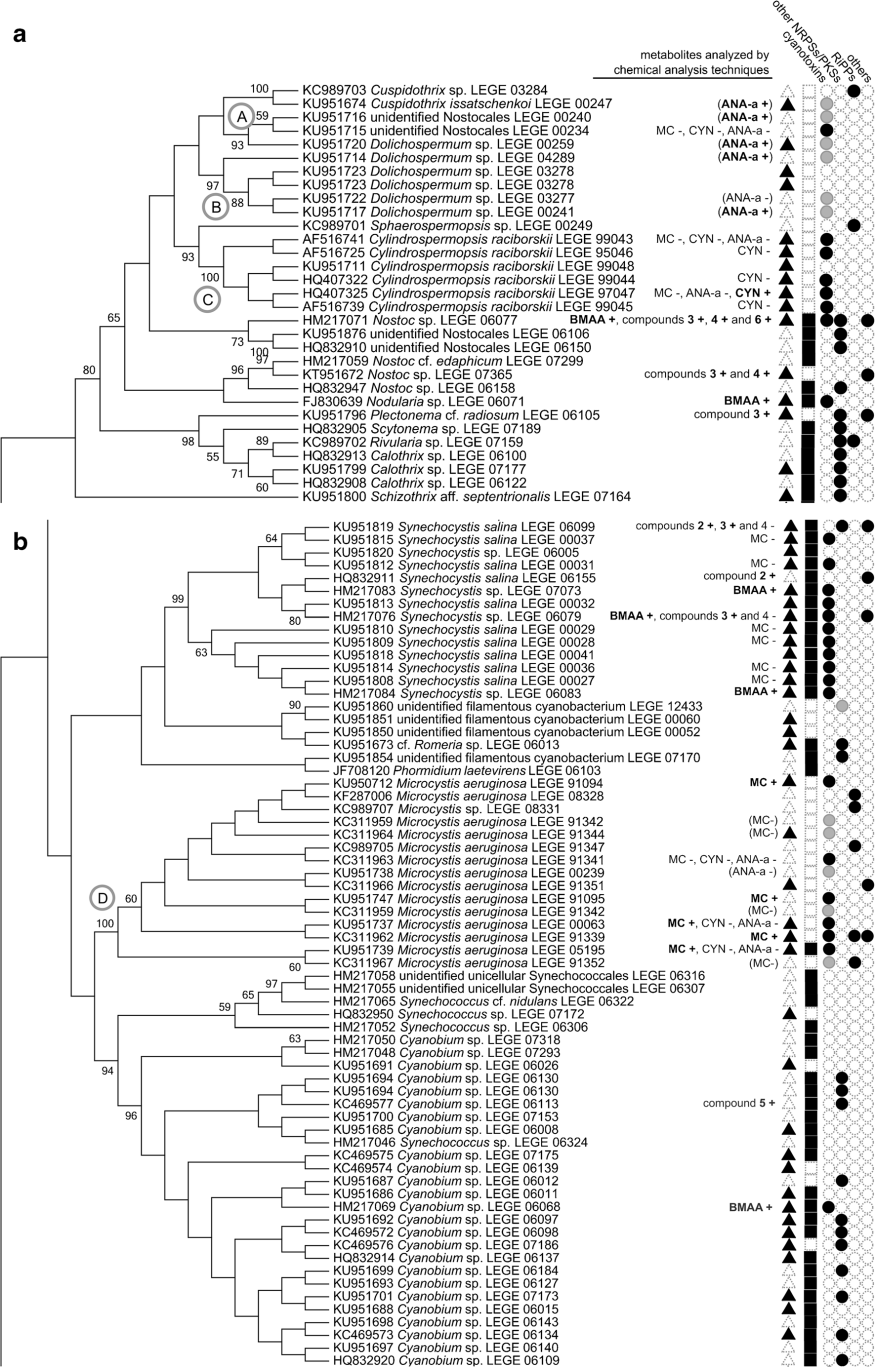

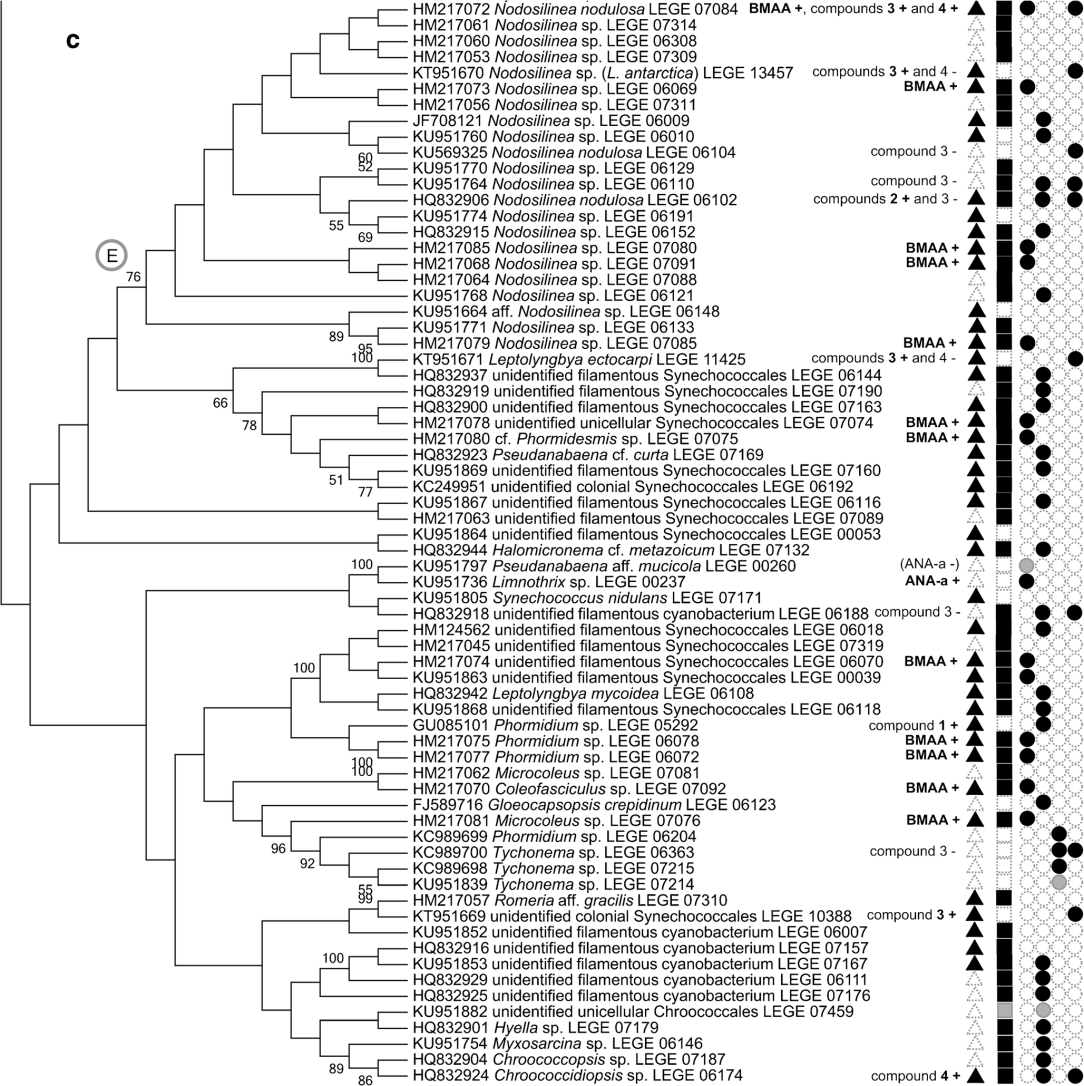
ML cladogram (− lnl = 3431.5512) for 165 LEGE CC strains having available data related to natural products. Capital letters in the tree highlight clades encompassing close-related strains for which the production of some of the following specific metabolites were detected (+) or not (−): Cyanotoxins: ANA-a anatoxin-a, BMAA β-Methylamino-L-alanine, CYN cylindrospermopsin, and MC microcystin. Bioactive compounds: 1 portoamides, 2 bartolosides, 3 dehydroabietic acid, 4 abietic acid, 5 hierridin B, and 6 anabaenopeptins A and D. Notice that the production (+) or absence of production (−) of the different compounds were confirmed by analytical techniques such as HPLC, LC-MS, or NMR. Metabolites between parentheses and symbols in gray indicate unpublished data. Symbols indicate the existence of data (either for the detection or non-detection) on: toxicity, bioactivity, or allelopathy assays (▲); screening of metabolites by MALDI-TOF Mass Spectrometry or by LC–MS analysis coupled with molecular networking [13] (■); cyanotoxins (•, first column); other than cyanotoxins nonribosomal peptide synthetases, polyketide synthases, or hybrid NRPS-PKS (•, second column); ribosomally synthesized and post-translationally modified peptides (Martins et al. 2013) (•, third column); and other family of compounds such as terpenes, glycolipids, etc. (•, fourth column). To get at the data on a particular strain, please find the literature references in the corresponding catalog sheet (Online Resource 3)

Several LEGE CC strains have been used in academia, most of them in research related to cyanobacterial natural products, as underlined by data available in the literature. Such information was found to be disseminated through 98 different journal articles (for a reference list, see Online Resource 3). In December 2016, 171 strains (44% of the total) had some sort of data available in published journal articles, from which 165 (43%) concerned natural products, including toxins (see also Figs. 3 and 4 and Online Resource 3). The three most frequently reported LEGE CC strains were found to be included in ten or more journal articles (Fig. 3 and Online Resource 3). These are the microcystin-producing (MC) strain *Microcystis aeruginosa* LEGE 91094 (Fig. 2d), the cylindrospermopsin-producing (CYN) strain *Cylindrospermopsis raciborskii* LEGE 97047, and *Cylindrospermopsis raciborskii* LEGE 95046, a non-CYN producer (Fig. 2t).

### Strain characterization and identification

The morphological and molecular-based characterization exposed the wide diversity of LEGE CC strains (Figs. 2 and 3), being included in six orders (Fig. 3) and 46 genera (Table 1). Komárek et al. (2014) have recently proposed a new taxonomy classification for cyanobacteria. Based on phylogenetic systematics, these authors have either erected new cyanobacterial orders or redefined the classical ones. For instance, unicellular or colonial cyanobacteria formerly included in the classical order Chroococcales (Komárek and Anagnostidis 1998) are now distributed in the new order Synechococcales and/or in the revisited Pleurocapsales (Komárek et al. 2014). The same is true to filamentous non-heterocytous cyanobacteria, which were traditionally included in the Oscillatoriales (Komárek and Anagnostidis 2005) and are now distributed in the redefined orders Chroococcales or Oscillatoriales (Komárek et al. 2014). Accordingly, several LEGE CC strains that were previously assigned to those classical orders (e.g., Brito et al. 2012; Lopes et al. 2012) were now re-classified by using this new classification scheme (Komárek et al. 2014) and by assessing their phylogenetic position, as depicted in Fig. 3 (also provided in a scalable, high quality vector format in Online Resource 1). For this purpose, sequences from the same reference strains included in the phylogeny performed by Komárek et al. (2014) were used in our analysis, which has permitted to map out the orders in the phylogenetic tree (Fig. 3).

**Table 1:** Number of cyanobacterial strains, by taxa, in LEGE CC (386 strains in total)

Order	Genus (reference)	Number of strains^§^	Origin	Ecology
Chroococcales	*Cyanobacterium*	1	*Por*	f
	*Geminobacterium*^&^ (Brito et al. 2017)	1*	*Por*	m
	*Microcystis*	37 (9)	*Bra*, *Gre*, *Mex*, *Mor*, *Por*	f
	*Synechocystis*	21 (3)	*Por*	b, f, and m
	unidentified Chroococcales	4	*Por*	m
Chroococcidiopsidales	*Gloeocapsa-*like^*+*^	1	*Por*	m
	*Gloeocapsopsis* ^*+*^	4	*Por*	m
	unidentified Chroococcidiopsidales	3	unknown	unknown
Nostocales	*Anabaena*	3 (1)	*Fin*	f
	*Calothrix*	2	*Por*	m
	*Chrysosporum*^&^ (Zapomělová et al. 2009)	1 (1)	*Isr*	f
	*Cuspidothrix*^&^ (Rajaniemi et al. 2005)	4 (1)	*Por*	f
	*Cylindrospermopsis*	6 (1)	*Aus*, *Por*	f
	*Desmonostoc*^&^ (Hrouzek et al. 2013)	1	*Por*	t
	*Dolichospermum*^&^ (Wacklin et al. 2009)	11 (4)	*Por*	f
	*Fortiea*	1	unknown	unknown
	*Nodularia*	2 (1)	*Por*	b, f
	*Nostoc*	14 (1)	*Mor*, *Por*	b, m, and t
	*Plectonema* ^*+*^	2	*Por*	m
	*Rivularia*	3	*Por*	m
	*Roholtiella*^&^ (Bohunická et al. 2015)	1	*Por*	f
	*Scytonema*	2	*Por*	m
	*Sphaerospermopsis*^&^ (Zapomělová et al. 2009)	4	*Mex*, *Por*	f
	*Tolypothrix*	1	*Por*	f
	unidentified Nostocales	9	*Col*, *Por*	f, m, and t
Oscillatoriales	*Coleofasciculus*^&^ (Siegesmund et al. 2008)	2 (1)	*Por*	b
	*Limnoraphis*^&^ (Komárek et al. 2013)	1	unknown	f
	*Lusitaniella*^&^ (Brito et al. 2017)	4*	*Por*	m
	*Microcoleus*	1 (1)	*Por*	b
	*Oxynema*^&^ (Chatchawan et al. 2012)	2 (2)	*Por*	b, m
	*Phormidium*	5	*Mor*, *Por*	f, m
	*Planktothrix*	10	*Por*	f
	*Spirulina* ^*+*^	1	*Por*	m
	*Tychonema*	21	*Col*, *Por*	f
	unidentified Oscillatoriales	4	*Mor*, *Por*	f, m
Pleurocapsales	*Chroococcidiopsis* ^*+*^	1	*Por*	m
	*Chroococcopsis*	2	*Por*	m
	*Hyella*	1	*Por*	m
	*Myxosarcina*	1	*Por*	m
	unidentified Pleurocapsales	2	*Por*	m
Synechococcales	*Alkalinema*^&^ (Vaz et al. 2015)	1	*Bra*	f
	*Calenema*^&^ (Brito et al. 2017)	1*	*Por*	m
	*Cyanobium*	48 (1)	*Chi*, *Mor*, *Por*	b, f, and m
	*Geitlerinema*	4	*Bra*	h
	*Halomicronema*^&^ (Abed et al. 2002)	1	*Por*	m
	*Jaaginema*	1	*Por*	m
	*Leptolyngbya*	13	*Bra*, *Por*	f, h, and m
	*Limnothrix*	1 (1)	*Por*	f
	*Nodosilinea*^&^ (Perkerson 3rd et al. 2011)	44 (5)	*Ant*, *Por*	b, f, m, and t
	*Oculatella*^&^ (Zammit et al. 2012)	1	*Por*	m
	*Phormidesmis*^&^ (Turicchia et al. 2009)	5	*Por*	m
	*Pseudanabaena*	3	*Por*	f, m
	*Romeria*	4	*Por*	b, m
	*Schizothrix*	1	*Por*	m
	*Synechococcus*	12	*Bra*, *Por*	b, h, and m
	*Toxifilum*^&^ (Zimba et al. 2017)	1	*Por*	m
	unidentified Synechococcales	32 (3)	*Chi*, *Mex*, *Mor*, *Por*	b, f, h, and m
Unclear taxa	unidentified cyanobacterium	16	*Bra*, *Chi*, *Mor*, *Por*	f, h, and m

*Ant*, Antarctica; *Aus*, Australia; *Bra*, Brazil; *Chi*, Chile; *Col*, Colombia; *Fin*, Finland; *Gre*, Greece; *Isr*, Israel; *Mex*, Mexico; *Mor*, Morocco; *Por*, Portugal; *b*, brackish water; *f*, freshwater; *h*, hypersaline; *m*, marine; *t*, terrestrial

^&^Recently described taxa; references only to these genera

^§^In parentheses indicated the number of strains known to produce common cyanotoxins (including BMAA)

^+^The inconsistency between genus and order assignments (as in Komárek et al. 2014) seems to indicate that taxonomic revision of these taxa is in need (the order placement was defined by phylogeny; see Fig. 3 or Online Resource 1)

*Including the strain used to describe the genus (see Brito et al. 2017)

The abovementioned grouping of unicellular and filamentous non-heterocytous forms into new orders is illustrated by a selection of LEGE CC strains included in Fig. 2. Colonial forms that were divided by multiple fission (Fig. 2g–i) and heterocytous strains (Fig. 2m–t) from the LEGE CC were found to be part of the Pleurocapsales and Nostocales clades, respectively (Fig. 3).

Interestingly, the filamentous cyanobacterium *Plectonema* cf. *radiosum* LEGE 06114 (Fig. 2o), which lacks heterocytes and akinetes, exhibits discoid cells and rounded apical cells, and shows visible sheaths and double false-branching (Brito et al. 2012), is phylogenetically placed within the Nostocales (Fig. 3). *Plectonema* is traditionally classified in the Oscillatoriales as it lacks specialized cells (Komárek and Anagnostidis 2005; Komárek et al. 2014), but its taxonomy is debatable and requires revision (Komárek and Anagnostidis 2005). For instance, as observed with *Plectonema* cf. *radiosum* LEGE 06114, some *Plectonema* species exhibit double false-branching similar to those found in Nostocales genera (e.g., *Scytonema*, (Komárek 2013)) and could be transferred to this order according to Komárek and Anagnostidis (2005). Information on these and other (apparent) taxonomic incongruities, peculiarities, or doubts that may have arisen after the identification of LEGE CC strains were included in the catalog sheet of the corresponding strain, as taxonomic notes (Online Resource 3).

The so-called modern approach currently recommended for identification of cyanobacteria (e.g., Komárek 2016) has cause and will continue to result in important changes for the taxonomy of cyanobacteria (Komárek et al. 2014). Traditional genera or species, especially those with little phenotypic differentiation, very often exhibit polyphyly in phylogenetic studies (see Dvořák et al. (2015) for a review). Such findings suggest that extensive taxonomic revisions of those taxa are in need (Komárek et al. 2014; Dvořák et al. 2015, Komárek 2016). As a consequence, the number of new genera that are being described using combined taxonomy of morphology and molecular phylogeny is growing rapidly, being that several of these genera represent earlier entangled, cryptic taxa that have emerged from traditional genera (Dvořák et al. 2015; Komárek 2016). Given the current status of taxonomy, and as implicitly recommended by Dvořák et al. (2015), we have adopted a conservative approach for the identification of LEGE CC strains at low taxonomic levels. The availability and inclusion of sequences from Type strains (Ramos et al. 2017) in the phylogenetic analysis (Fig. 3) was essential to accurately identify the strains, namely to ascertain if they could belong to recently proposed genera not covered by the classification keys used (Komárek and Anagnostidis 1998, 2005; Komárek 2013). Therefore, previous morphology-based identifications of the strains were not considered if the phylogeny indicated that the strains belong to such recent genera, or if they were phylogenetically placed away from the holotype in question (i.e., Type strain used to describe a genus) (Fig. 3). Applying these criteria resulted in 70 LEGE CC strains remaining unidentified since it was not possible to achieve an unequivocal identification at the genus level, even if in most cases it was possible to achieve an assignment at the order level (Table 1). On the other hand, 86 strains were identified as belonging to 18 recently described genera by means of modern taxonomy (see Table 1).

Well represented genera at LEGE CC include the picocyanobacterium *Cyanobium* (48 strains; Fig. 2a), the filamentous non-heterocytous *Nodosilinea* (44; Fig. 2b), the bloom forming *Microcystis* (37, including both microcystin and non-microcystin producers; Fig. 2d), the unicellular *Synechocystis* (21), and the filamentous non-heterocytous *Tychonema* (21).

LEGE CC aims to value its cyanobacterial diversity in a way that can be perceived by others, namely by stakeholders from the biotechnology sector. As such, strains are characterized in order to highlight features that may have interest from an applied point of view. As depicted from the qualitative evaluation made by different staining techniques (see as an example Online Resource 2), several LEGE CC strains produce considerable amounts of EPSs (Fig. 3), a feature that may have biotechnological applications. For instance, cyanobacterial EPSs can be used for heavy metal removal from contaminated waters (Pereira et al. 2011), as was already demonstrated for one of our strains, *Synechocystis* sp. LEGE 00032 (Ribeiro et al. 2008). Also, six strains (Fig. 3) have had their genomes sequenced and these will be made publicly available, following curation. One such strain, *Cyanobium* sp. LEGE 06113, has been included on a submitted patent application for a promising anti-malarial compound. Some strains held in LEGE CC have an earthy odor, something that may indicate the presence of odiferous metabolites such as 2-methlyisoborneol or geosmin (Giglio et al. 2010), two volatile organic compounds that pose problems in drinking water supply systems. This qualitative data was included in the catalog of strains (Online Resource 3).

### LEGE CC strains and their (potential) chemodiversity

Since the main research lines of BBE are ecotoxicology and the discovery of new natural products, in particular, those with biotechnological potential, it is not surprising that a considerable fraction of LEGE CC strains (43%) have been studied and/or used for their potential production of bioactive secondary metabolites (see Fig. 4).

In total, there are 37 strains in LEGE CC known to produce common cyanotoxins (Fig. 3). Details and information related to shipment, handling, and disposal of toxic strains, verification of toxin production by LEGE CC, expertise services, etc. are included in the catalog (Online Resource 3). Nine out of 32 *Microcystis aeruginosa* strains included in LEGE CC are MC producers. Other toxin-producing strains include the anatoxin-a (ANA-a) producers *Dolichospermum* spp. LEGE 00240, 00241, and 04289, and *Limnothrix* sp. LEGE 00237, the CYN producer *Cylindrospermopsis raciborskii* LEGE 97047, as well as several strains, belonging to different taxa, that produce β-methylamino-L-alanine (BMAA), a toxin shown to be widespread among cyanobacteria (Cox et al. 2005; Cianca et al. 2012) (see also Fig. 3). Besides toxins, other secondary metabolites (e.g., hierridin B, portoamides, and bartolosides) are known to be produced by LEGE CC strains. Indeed, some LEGE CC strains were used (Leão et al. 2010, 2013a, 2015; Costa et al. 2016) to isolate novel and known bioactive metabolites (Fig. 3).

Currently, 165 cyanobacterial LEGE CC strains, representing 43% of the total number of strains, have some associated data (mostly published) concerning the production of natural products or information on biological activity of their constituents (Fig. 4). The phylogenetic relationships among these strains and associated data are depicted in the unrooted tree shown in Fig. 4. If available, data can be reached through the publications mentioned in the catalog sheet for a particular strain, whereas the full references are listed at the end of the catalog (Online Resource 3).

LEGE CC strains have the potential (e.g., presence of genes involved in the biosynthesis of secondary metabolites) or the effective capacity to produce different chemical compounds (Fig. 4, see also Leão et al. 2013b; Martins et al. 2013; Brito et al. 2015). Several of those compounds being produced by LEGE CC strains exhibit anti-cancer (e.g., portoamides and hierridin B; Leão et al. 2010, 2013a), anti-viral (crude extract; Lopes et al. 2011), anti-microbial (fractions; Costa et al. 2014, 2015; Leão et al. 2013a), or anti-biofouling (crude extract; Almeida et al. 2015) properties. Dittmann et al. (2015) claim that more than 1100 secondary metabolites already known to be produced by cyanobacteria are just a fraction of the true metabolic potential of these microorganisms. As an example, some LEGE CC strains were used to isolate unprecedented bioactive secondary metabolites (Figs. 3 and 4, compounds 1 and 2), the lipopeptides portoamides (*Phormidium* sp. LEGE 05292) (Leão et al. 2010) and the dialkylresorcinol glycolipids bartolosides (*Synechocystis salina* LEGE 06155 and *Nodosilinea nodulosa* LEGE 06102) (Leão et al. 2015). The diterpenoid dehydroabietic acid, isolated from *Plectonema* cf. *radiosum* LEGE 06105 and the unidentified colonial Synechococcales LEGE 10388 (Figs. 3 and 4), was for the first time detected in an organism other than gymnosperms (Costa et al. 2016). By screening 15 LEGE CC strains, Costa et al. (2016) soon demonstrated that this and one other terpenoid, the abietic acid, are present in a wide range of cyanobacteria (Fig. 4, compounds 3 and 4). In the same study, it was also shown that in some cases the two compounds could not be detected in strains closely related to the diterpenoid-producing cyanobacteria. The same pattern can be observed in different cyanobacterial clades (A-E) highlighted in Fig. 4, for different metabolites studied by analytical methods. For instance, regarding toxins, there are closely related LEGE CC strains assigned as ANA-a producers and non-producers, in clade A and B, CYN producers and non-producers in clade C, and MC producers and non-producers in clade D. Closely related strains that produce or did not produce the diterpenoids are included in clade E. Of course, a metabolite can remain undetected if it is being produced at low levels, below the limit of detection of the analytical technique. It is also possible that some of the biological activities observed are related to the microbiota associated with the cyanobacteria and not to the cyanobacteria themselves; however, in light of the well-recognized ability of cyanobacteria to produce bioactive compounds and to the low densities of heterotrophic bacteria in these unicyanobacterial cultures, we find that this is rather unlikely. It can also happen that, under some conditions (e.g., lack of environmental stimuli), a cyanobacterium does not produce a particular metabolite despite possessing the biosynthetic pathway to produce it (Watanabe and Oishi 1985; Boopathi and Ki 2014). It is also possible that the biosynthetic machinery is inactive (e.g., due to gene mutation events) (Leikoski et al. 2012; Vestola et al. 2014). Comparative genomics studies on diverse cyanobacterial taxa have demonstrated that closely related strains (i.e., at the subspecies level) may present high levels of genome divergence (Rocap et al. 2003; Shih et al. 2013; Bombar et al. 2014; Calteau et al. 2014). For instance, some of those phylogenetically highly related strains may possess functionally active genes (or gene clusters) linked to the production of natural products, while others do not (Shih et al. 2013; Sinha et al. 2014; Calteau et al. 2014; Dittmann et al. 2015). On the other hand, it reinforces the importance of the clonal status of strains for securing reproducibility of results, since strains from the same population may exhibit very different biosynthetic potential as depicted from the study of Shih et al. (2013). All these issues have important implications for the discovery of natural compounds from cyanobacteria. In particular, it indicates that, for an exploration of the full potential of these microorganisms as a source of natural products, bioprospection should be ideally conducted strain-by-strain rather than taxonomically or phylogenetically guided (Dittmann et al. 2015).

## Conclusions

Acting as repositories of strains and of their genetic material, mBRCs facilitate the access to their diversity, their (meta)data and their associated natural compounds, being able to satisfy the needs of academia or the industry. With this in mind, we decided to organize our cyanobacterial strains into a publicly available culture collection. The cyanobacterial diversity that currently makes up the LEGE CC is an increasingly important bioresource, either from the taxonomic point of view (e.g. Ramos et al. 2010 in Komárek et al. 2014; Brito et al. 2017) or from a biotechnological perspective (e.g., Brito et al. 2015). LEGE CC is now a member of the WFCC (WDCM #1089), also part of EMBRC.PT, the Portuguese node of the research infrastructure European Marine Biological Resource Centre. Possible biotechnological applications for LEGE CC strains and their bioproducts were described in several studies, synopsized here, and are related to their anti-cancer, anti-viral, anti-microbial, or anti-biofouling properties. Even though using phylogenetic diversity data is a valid strategy for directing strain selection for natural product screening, this study illustrates that natural product discovery programs should consider a strain-by-strain assessment.

## Electronic supplementary material

ESM 1 Online Resource 1 Scalable and searchable, high quality vector format of Fig. 3. ML tree of 16S rRNA gene sequences illustrating the phylogenetic diversity of 307 LEGE CC strains (colored labels), their placement at the order level, and some traits or information relevant for biotechnological purposes (see the caption of Fig. 3 for full details). Novel sequences obtained in this study are labelled in dark blue (194 totally original sequences) or light blue (29 assembled sequences that extend existing sequences), while existing sequences (84) already in GenBank are labelled in green (PDF 192 kb)

ESM 2 Online Resource 2 Examples of strains evaluated for exopolysaccharides (EPS) production. Rows (a-g) refer to different strains, and columns (1-4) to different types of staining techniques. (a) *Microcystis aeruginosa* LEGE 91353; (b) *Nostoc* sp. LEGE 06077; (c) *Planktothrix mougeotii* LEGE 06225; (d) *Myxosarcina* sp. LEGE 06146; (e) *Synechococcus nidulans* LEGE 07171; (f) unidentified filamentous Synechococcales LEGE 06018; (g) unidentified filamentous cyanobacterium LEGE 00060. (1) no staining; (2) stained with 0,5% Alcian Blue (*w*/*v*) in 50% ethanol; (3) stained with 0,5% Alcian Blue (w/v) in acetic acid; (4) negative staining with India ink. Scale bars represent 20 µm (JPG 22.9 mb)

ESM 3 Online Resource 3 First version of the LEGE CC catalog of strains (PDF 9.71 mb)
