# Supplementary figures and images for: Cyanobacterial diversity held in microbial biological resource centers as a biotechnological asset: the case study of the newly established LEGE culture collection
Source: J Appl Phycol. 2018 Jan 6;30(3):1437–51. doi: 10.1007/s10811-017-1369-y (PMC5982461; doi:10.1007/s10811-017-1369-y)

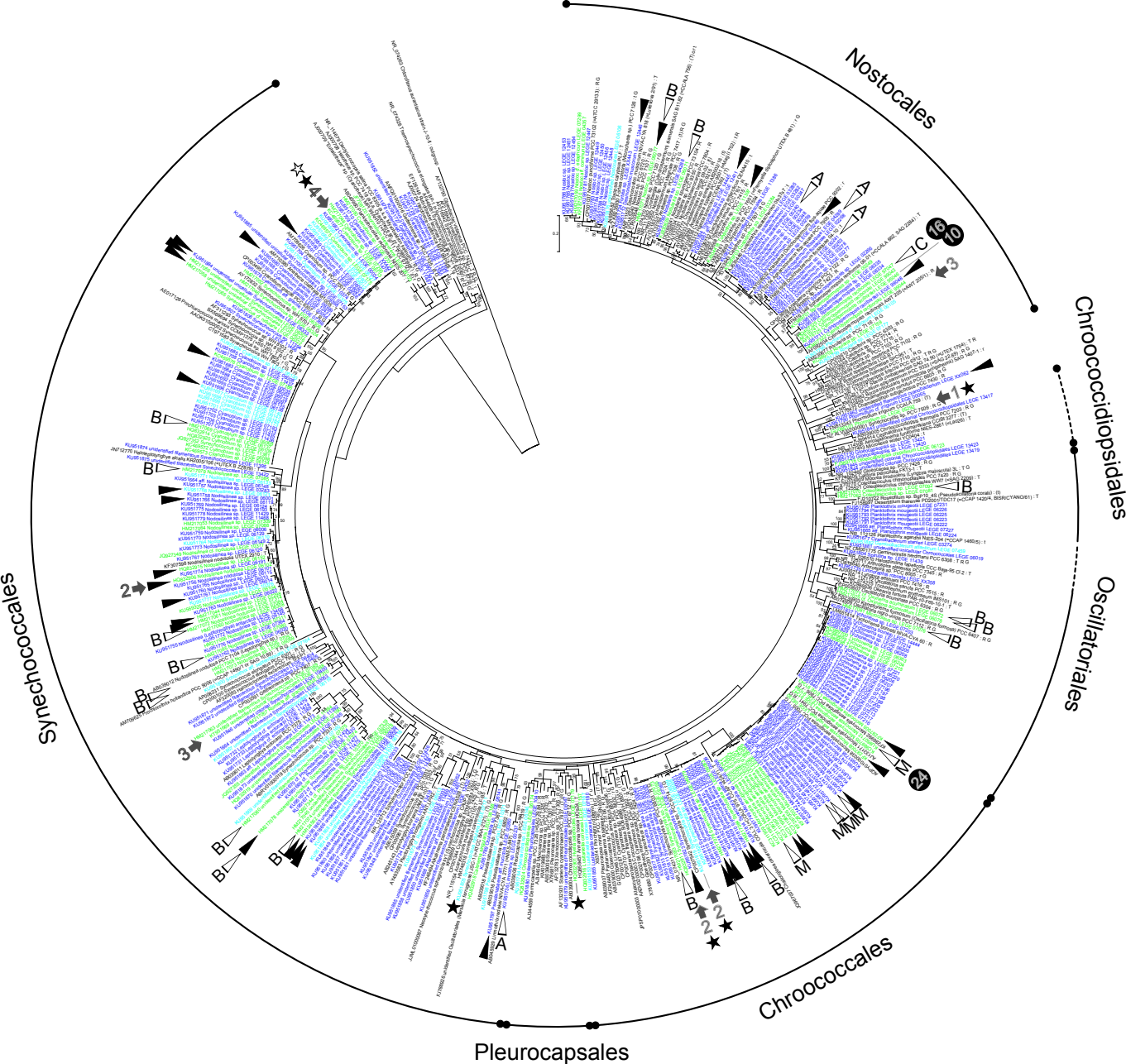

Supplement: Supplementary file 1 — Online Resource 1 Scalable and searchable, high quality vector format of Fig. 3. ML tree of 16S rRNA gene sequences illustrating the phylogenetic diversity of 307 LEGE CC strains (colored labels), their placement at the order level, and some traits or information relevant for biotechnological purposes (see the caption of Fig. 3 for full details). Novel sequences obtained in this study are labelled in dark blue (194 totally original sequences) or light blue (29 assembled sequences that extend existing sequences), while existing sequences (84) already in GenBank are labelled in green (PDF 192 kb) [file 10811_2017_1369_MOESM1_ESM.pdf]

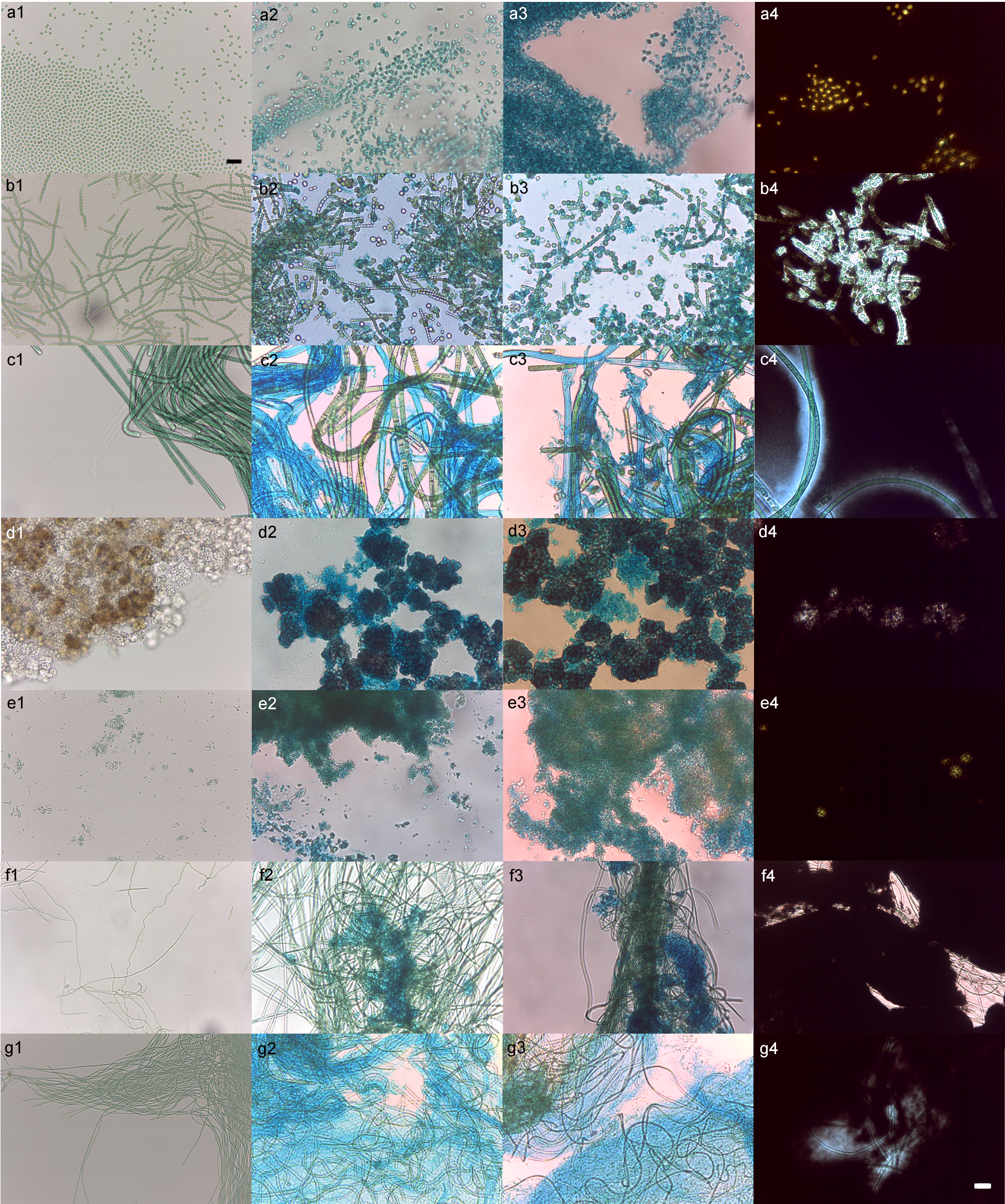

Supplement: Supplementary file 2 — Online Resource 2 Examples of strains evaluated for exopolysaccharides (EPS) production. Rows (a-g) refer to different strains, and columns (1-4) to different types of staining techniques. (a) Microcystis aeruginosa LEGE 91353; (b) Nostoc sp. LEGE 06077; (c) Planktothrix mougeotii LEGE 06225; (d) Myxosarcina sp. LEGE 06146; (e) Synechococcus nidulans LEGE 07171; (f) unidentified filamentous Synechococcales LEGE 06018; (g) unidentified filamentous cyanobacterium LEGE 00060. (1) no staining; (2) stained with 0,5% Alcian Blue (w/v) in 50% ethanol; (3) stained with 0,5% Alcian Blue (w/v) in acetic acid; (4) negative staining with India ink. Scale bars represent 20 µm (JPG 22.9 mb) [file 10811_2017_1369_MOESM2_ESM.jpg]
