# Supplementary material for: Cyanobacterial diversity held in microbial biological resource centers as a biotechnological asset: the case study of the newly established LEGE culture collection
Source: J Appl Phycol. 2018 Jan 6;30(3):1437–51. doi: 10.1007/s10811-017-1369-y (PMC5982461; doi:10.1007/s10811-017-1369-y)
Supplement: Supplementary file 3 — Online Resource 3 First version of the LEGE CC catalog of strains (PDF 9.71 mb) [file 10811_2017_1369_MOESM3_ESM.pdf]

LEGE CULTURE COLLECTION

Strain Catalog

2017

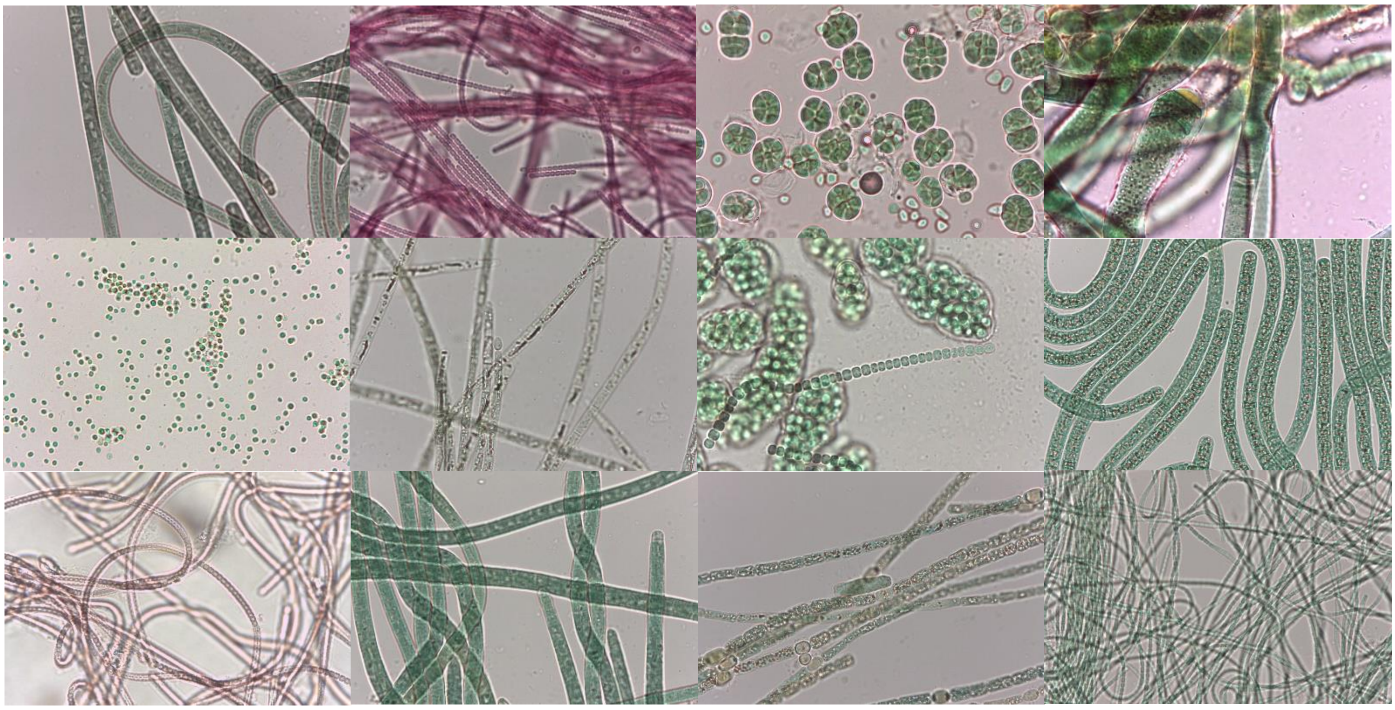

Version 1.0

CIIMAR, Matosinhos, Portugal

LEGE Culture Collection

Strain Catalog

Blue Biotechnology and Ecotoxicology Culture Collection (acronym **LEGE**)

Member of the World Federation for Culture Collections (WFCC - WDCM #1089)

764 Figures

Version 1.0

2017

If using information from this catalog, **please consider to cite**:

Ramos, V., Morais, J., Castelo-Branco, R., Pinheiro, Â., Martins, J., Regueiras, A., Pereira, A.L., Lopes, V., Frazão, B. Gomes, D., Moreira, C., Costa, M.S., Brûle, S., Faustino, S. Martins, R., Saker, M., Osswald, J., Leão, P.N., Vasconcelos, V.M. (2018). Cyanobacterial diversity held in mBRCs as a biotechnological asset: the case study of the newly established LEGE Culture Collection. *Journal of Applied Phycology*. DOI: 10.1007/s10811-017-1369-y

Address and contacts

LEGE Culture Collection - Blue Biotechnology and Ecotoxicology
Terminal de Cruzeiros do Porto de Leixões
Avenida General Norton de Matos, S/N
4450-208 Matosinhos | Portugal

Tel.: +351 22 340 18 37

LEGE Website: http://lege.ciimar.up.pt/

Email: legeculturecollection@gmail.com

Acknowledgement

The Project NOMORFILM has received funding from the European Union's Horizon 2020 Research and Innovation Programme under grant agreement No 634588. This work was partially funded by FCT Project UID/Multi/04423/2013 and by the Structured Program of R&D&I INNOVMAR - Innovation and Sustainability in the Management and Exploitation of Marine Resources (reference NORTE-01- 0145-FEDER-000035, Research Line NOVELMAR), funded by the Northern Regional Operational Program (NORTE2020) through the European Regional Development Fund (ERDF).

Table of contents

General Information **iii**

Diversity and Taxonomic Issues iii

Preservation and Quality Control iv

Other Services v

Terms and Conditions **v**

Ordering v

Prices iv

Culture Supply v

LEGE Acknowledgement vi

Handling and Safety Issues vi

Deposit vi

Catalog - Strain Sheets **1**

References **386**

Index of Strains **396**

lege CC
Blue Biotechnology and Ecotoxicology Culture Collection

General Information

LEGE is the acronym for the Blue Biotechnology and Ecotoxicology Culture Collection at CIIMAR, Portugal. It comprises more than 380 cyanobacterial strains, several of them unique among the phylogenetic diversity of the group. **LEGE** is a comprehensive Biological Resource Centre of living culture material of cyanobacteria. Strains currently at **LEGE** were primarily isolated by members from the Blue Biotechnology and Ecotoxicology lab group, at CIIMAR. They have been obtained since 1991, from samples collected in different environments and locations mainly in Portugal (including Islands).

LEGE is member of World Federation for Culture Collections (WFCC, WDCM #1089), and it is also part of EMBRC.PT, the Portuguese node of the research infrastructure European Marine Biological Resource Centre.

LEGE seeks to provide products (i.e. strain cultures), services and expertise to national, regional and international stakeholders, for different aims (e.g. R&D in academia and industry; science education & dissemination).

Diversity and Taxonomic Issues

Currently, the **LEGE** collection maintains over 380 strains of living cyanobacteria, which represent 46 genera spread across the phylogenetic diversity of these organisms. The strains were isolated mainly from saline and freshwater habitats in Portugal. An online-database can also be used to search for strains and associated data.

Although primarily focused on cyanobacteria, in the near future the collection will harbor eukaryotic microalgae.

Strains are characterized by means of genotypic and phenotypic-based approaches, in accordance with recent recommendations for identification. Currently, 80% of the LEGE strains have their 16S rRNA gene sequences deposited in GenBank.

The ever-changing nature of taxonomy causes identification to change over time. The current status of taxonomy of cyanobacteria and microalgae also renders difficult to keep the identification of strains present in a collection up-to-date. **LEGE** strains identifications are revised periodically and any taxonomic update for a strain will be added in the online-database. **LEGE** always revises the identification of deposited strains, which are usually identified by the depositor. Suggestions for the correction of names are welcomed.

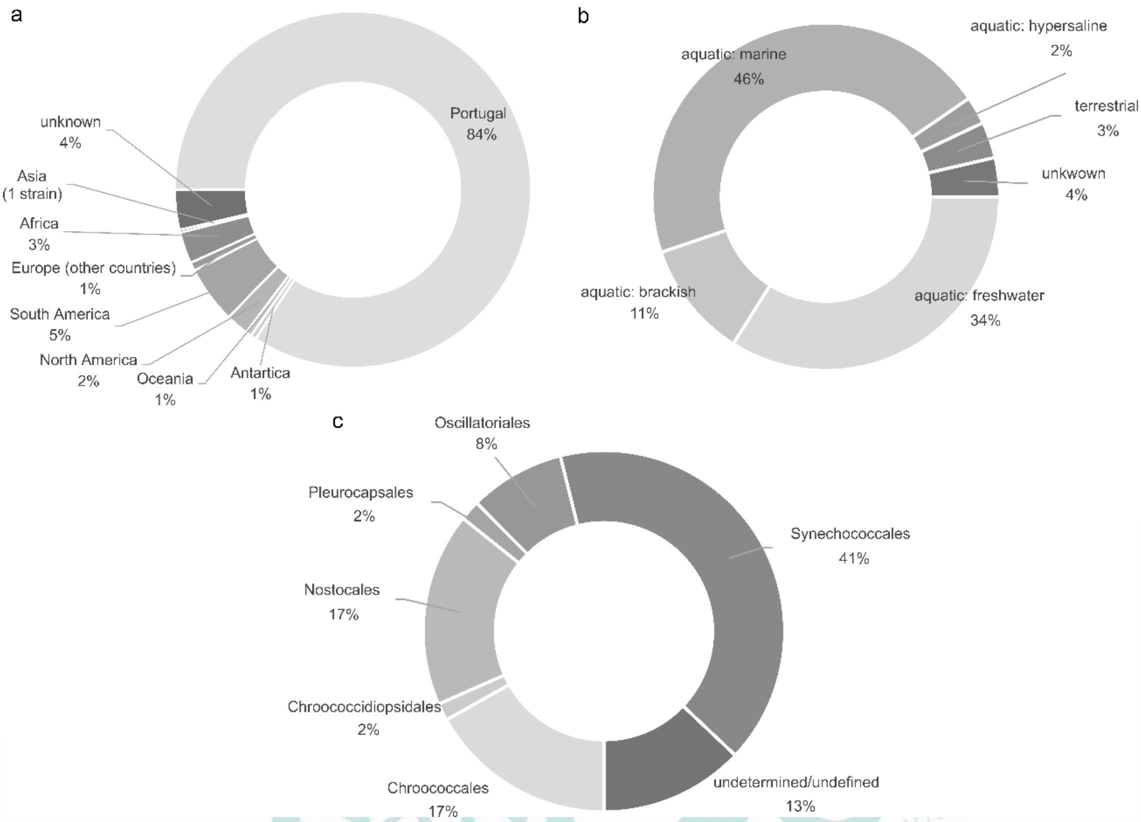

General statistics for the 386 LEGE cyanobacterial strains concerning (a) geographical origin, (b) source/type of habitat, and (c) taxonomic classification at the order level

Preservation and Quality Control

Cyanobacterial LEGE strains are normally kept at 10–30 µmol photons m^{-2} s^{-1} under 12h/12h or 14h/10h light/dark cycles. The range of controlled temperature conditions at LEGE are 14ºC, 19ºC (for most strains), and 25ºC. For specific culture conditions of a strain, please see the respective catalog sheet.

Strains included in this catalog version are maintained by subculturing, in four different media (see p. 1). Subculturing transfers range from 2 (e.g. sensitive/delicate strains) to 6 months. Some strains are also cryopreserved at -80ºC. Soon, a stock comprising the full collection will be cryopreserved and stored at -150ºC. Any new entry in **LEGE** will be immediately cryopreserved. Strain replicates will be regularly thawed and evaluated for quality control, and the stock replenished. This long-term preservation procedure allows the genetic stability of **LEGE** strains and reduces the risk of strain contamination or loss.

LEGE strains included in this first version of the catalog are unialgal/unicyanobacterial, clonal, and xenic (non-axenic). Axenicization of strains will be attempted in the future; still, the original xenic culture will be retained (in such cases, the strain code of both cultures will be slightly different - an “A” will be added to the axenic culture).

Prior to any shipment, the morphological traits, growth and fitness of the strain are visually inspected.

Cyanotoxin production by toxic strains are regularly evaluated by analytical chemistry methods, using CIIMAR facilities.

Other Services

- Identification
- Isolation
- Cryopreservation of private strains
- Training and courses (basic microbiology; identification; molecular methods; phylogenetic analysis; cryopreservation)
- Consultancy advisory (cultivation; algal biotechnology)

Send us an expression of interest by using the contact form in the online-database or through our email.

Terms and Conditions

Ordering

Send us an expression of interest by using the contact form in the online-database or through our email.

Full details and documentation for order confirmation, and instructions for payment will be sent at that time.

For instance, before the supply of strain cultures from the **LEGE** collection, the customer shall complete and sign an Order Form and a Material Transfer Agreement.

By ordering strains from **LEGE**, the customer agrees to not resell, borrow, transfer, or give our strains to third parties.

Prices

For academic/non-profit use: 60€. For commercial use: 150€ (each strain).

Since our prices can change, for a current value users are advised to consult the online-database or to contact us directly by email.

For prices on other services, please contact us.

Culture Supply

Typically, for each strain ordered, **LEGE** supplies 2 x 5 ml tubes of culture in liquid medium.

Shipping costs are borne by customers. For Europe, cultures will normally be shipped by regular postal service. For other regions, as a rule, it shall be by express or priority shipping services.

Generally, shipping is performed 3-4 weeks after ordering and payment. Supply of strains at shorter notice cannot be guaranteed.

We cannot guarantee the viability of strain cultures upon receipt. Yet, provided that **LEGE** is notified within 2 weeks of dispatch, if cultures arrive in an unsatisfactory condition we can replace them. In this case the customer will be charged only for shipping.

LEGE Acknowledgement

Customers agree to cite properly the **LEGE** strain number(s) in all publications or patent applications and to acknowledge the **LEGE** collection as the source of the strains. Customers are kindly requested to inform us of any publication including **LEGE** strains.

Handling and Safety Issues

Tubes/cultures sent by **LEGE** should only be opened and used by trained persons, in suitable laboratory conditions. The volume and concentration of culture supplied is unlikely to be a serious hazard to laboratory workers, the community, livestock, or the environment. **LEGE** is not responsible to any damage or injury that might happen after arrival.

The customer should maintain, grow, use or dispose the cultures in accordance with appropriate procedures and precautions to avoid risks to persons and to the environment. Some LEGE strains are known to produce common cyanotoxins. In such cases, customers will be warned about it. Specific hazards that may be encountered when processing toxic cyanobacteria and recommended control measures can be found in Stewart et al. (2009). Nevertheless, since other **LEGE** strains may produce other secondary metabolites with unknown toxicity, all strains should be treated with caution.

In all situations, customers are advised to use a Class II biosafety cabinet for work with the cultures, and an autoclave or other suitable method (see Stewart et al., 2009) for disposal and decontamination within the lab.

Deposit

LEGE is open to deposition of strains, as long as the organisms adapt to growth conditions present in the collection. Candidate strains for deposition will be examined for contamination, proper identification and data accuracy.

Deposit of strains in **LEGE** is free of charge. However, shipping and transport costs are borne by the depositor.

Prior to the deposit process, depositor should review the status of the strains with regards to the Convention on Biological Diversity, the Nagoya Protocol or other related legal issues. All deposited strains are open to the public once their acceptance is approved.

legecc
Blue Biotechnology and Ecotoxicology Culture Collection

Catalog - Strain Sheets

Sheet fields' explanation and notes

For non-self-explanatory fields, only.

Order (Older Classification Scheme)

Classification schemes followed for the Order assignment:

- Chroococcales, except Pleurocapsales → Komárek & Anagnostidis (1998)
- Pleurocapsales → Waterbury & Stanier (1978) and Rippka et al. (2001)
- Oscillatoriales → Komárek & Anagnostidis (2005)
- Nostocales → Komarek (2013)

Order 2014 (Modern Taxonomy)

Classification scheme followed for the Order assignment:

- All Orders → Komárek et al. (2014)

Morphometrics

Quantifiable morphological characters were measured at least 20 times.

Culture media

For media recipes see, e.g.:

Kotai (1972) → Z8, and Rippka (1988) → BG11, $BG11_0$ and MN

* 25‰ TM sea salt – 25 g/L of Tropical Marin® sea salt
* vit. B12 – Filter-sterilized vitamin B_{12} (or cyanocobalamin) is added to media after autoclaving, at a final concentration of 10 µg/L

Taxonomy Notes

Remarks on the identification or taxonomic position of the strain.

Notes on ecophysiological traits

Details or elucidations about ecophysiological traits.

Taxonomic notes/diacritical features

Some morphological notes which may have taxonomic relevance.

Strain ID	**Alkalinema aff. pantanalense LEGE 15481**		
**Strain Taxonomy**	Alkalinema pantanalense Vaz, MGGV; Genuário, DB; Andreote, APDA; Malone, CFS; Sant'Anna, AL & Fiore, MF (2015)		
**Microphotograph 400×**	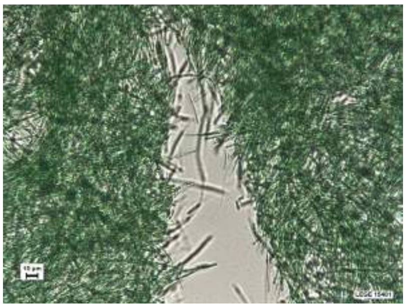	**Microphotograph 1000×**	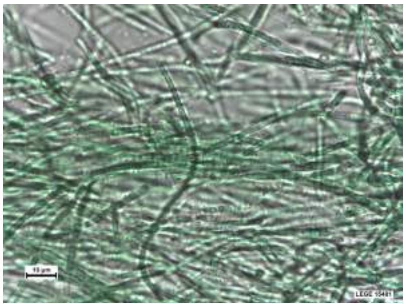
**Coidentity**	n.a.	**Morphometrics (Width × Length or Diameter) (µm)**	1,7±0,3 × 1,6±0,3
**Other Code(s)**	IEP	**Taxonomic notes/diacritical features**	
**Parent Sample**		**Macroscopic growth features**	color: cyano; forming a smooth biofilm
**Isolation Date**		**Environment**	aquatic, freshwater
**Order (Older Classification Scheme)**	Oscillatoriales	**Habitat Sample Description**	water sample, near a public water supply collection point
**Order 2014 (Modern Taxonomy)**	Synechococcales	**Ecophysiological traits**	
**Taxonomy Notes**		**Notes on ecophysiological traits**	
**Accession Number(s)_16S**	KU951668	**Isolator**	Silvia Faustino
**Accession Number(s)_others (product/gene /region)**		**Medium**	Z8
**Collector**	Sílvia Faustino	**Strain Status**	non-axenic, unicyanobacterial
**Collection Date**	2015	**Preservation type**	subculturing
**Location**	Brazil: Macapá, Amazon river	**Light:dark cycle**	14:10-h
**Latitude & Longitude**	0.022443 N 51.054853 W	**Temperature (ºC)**	25
		**Light intensity**	10–30 µmol photons m−2 s−1
**References Publicly Available Articles**			
**References Publicly Available Theses**			

**Strain ID**	**Anabaena aphanizomenoides LEGE 00250**		
**Strain Taxonomy**	Anabaena aphanizomenoides Forti, A (1911)		
**Microphotograph 400×**	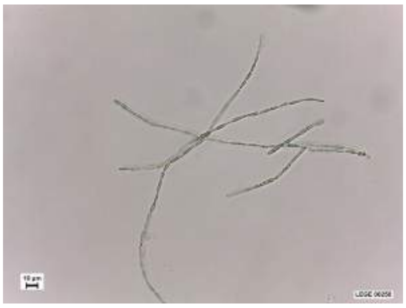	**Microphotograph 1000×**	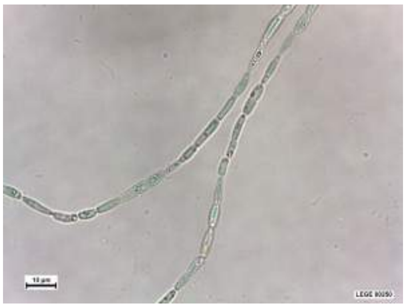
**Coidentity**	n.a.	**Morphometrics (Width × Length or Diameter) (µm)**	2,4±0,8 × 8,8±2,5
**Other Code(s)**	J27, LEANJ.27	**Taxonomic notes/diacritical features**	
**Parent Sample**		**Macroscopic growth features**	color: cyano; growing homogeneously, but also forming aggregates
**Isolation Date**		**Environment**	aquatic, freshwater
**Order (Older Classification Scheme)**	Nostocales	**Habitat Sample Description**	water sample, dam reservoir
**Order 2014 (Modern Taxonomy)**	Nostocales	**Ecophysiological traits**	
**Taxonomy Notes**		**Notes on ecophysiological traits**	
**Accession Number(s)_16S**		**Isolator**	Joana Osswald
**Accession Number(s)_others (product/gene /region)**		**Medium**	Z8
**Collector**	Joana Oswald	**Strain Status**	non-axenic, unicyanobacterial
**Collection Date**	2000/07/07	**Preservation type**	subculturing
**Location**	Portugal: Maranhão dam reservoir, Benavila	**Light:dark cycle**	12:12-h
**Latitude & Longitude**	unknown	**Temperature (ºC)**	19
		**Light intensity**	10–30 µmol photons m−2 s−1
**References Publicly Available Articles**			
**References Publicly Available Theses**	Osswald J. (2002) MSc Dissertation, FCUP, University of Porto, Porto, Portugal; Chaves R. (2013) MSc Dissertation, ICBAS, University of Porto, Porto, Portugal		

Strain ID **Anabaena cf. cylindrica LEGE 00235**

**Strain Taxonomy**	Anabaena cylindrica Lemmermann, E (1896)		
**Microphotograph 400×**	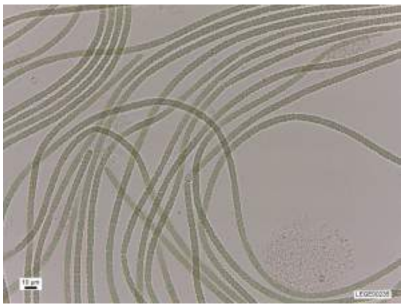	**Microphotograph 1000×**	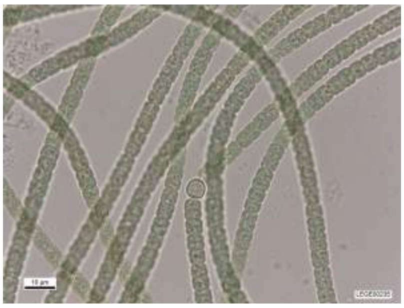
**Coidentity**	n.a.	**Morphometrics (Width × Length or Diameter) (µm)**	5,1±0,5 × 2,7±0,5 (Cells); Ø 5,4±0,6 (Heterocytes)
**Other Code(s)**	J4, LEANJ.4	**Taxonomic notes/diacritical features**	
**Parent Sample**		**Macroscopic growth features**	color: cyano; growing homogeneously; mucilaginous
**Isolation Date**		**Environment**	aquatic, freshwater
**Order (Older Classification Scheme)**	Nostocales	**Habitat Sample Description**	water sample
**Order 2014 (Modern Taxonomy)**	Nostocales	**Ecophysiological traits**	EPS production
**Taxonomy Notes**		**Notes on ecophysiological traits**	
**Accession Number(s)_16S**		**Isolator**	Joana Osswald
**Accession Number(s)_others (product/gene /region)**		**Medium**	
**Collector**	Joana Oswald	**Strain Status**	non-axenic, unicyanobacterial
**Collection Date**	2000/07/07	**Preservation type**	subculturing
**Location**	Portugal: Maranhão dam reservoir, Benavila	**Light:dark cycle**	12:12-h
**Latitude & Longitude**	unknown	**Temperature (ºC)**	19
		**Light intensity**	10–30 µmol photons m−2 s−1
**References Publicly Available Articles**			
**References Publicly Available Theses**	Osswald J. (2002) MSc Dissertation, FCUP, University of Porto, Porto, Portugal		

Strain ID	**Anabaena sp. LEGE X-002**		
**Strain Taxonomy**	Anabaena Bory ex Bornet, É & Flahault, C (1886 '1888')		
**Microphotograph 400×**	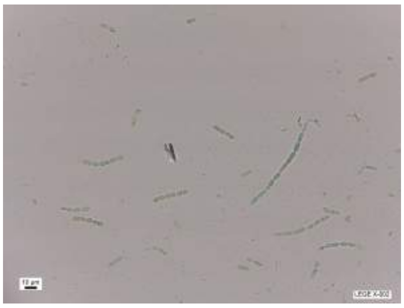	**Microphotograph 1000×**	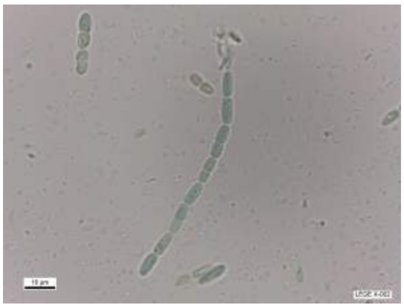
**Coidentity**	n.a.	**Morphometrics (Width × Length or Diameter) (µm)**	3,0±0,9 × 4,9±0,8
**Other Code(s)**	ANA-37, Sivonen	**Taxonomic notes/diacritical features**	
**Parent Sample**		**Macroscopic growth features**	color: cyano; forming aggregates
**Isolation Date**		**Environment**	aquatic, freshwater
**Order (Older Classification Scheme)**	Nostocales	**Habitat Sample Description**	water sample
**Order 2014 (Modern Taxonomy)**	Nostocales	**Ecophysiological traits**	toxin production, nitrogen-fixation
**Taxonomy Notes**		**Notes on ecophysiological traits**	ANTX-a producing strain
**Accession Number(s)_16S**		**Isolator**	Kaarina Sivonen
**Accession Number(s)_others (product/gene /region)**		**Medium**	Z8
**Collector**	Kaarina Sivonen	**Strain Status**	non-axenic, unicyanobacterial
**Collection Date**	1985/06/09	**Preservation type**	subculturing
**Location**	Finland: Lake Sääskjärvi	**Light:dark cycle**	12:12-h
**Latitude & Longitude**	unknown	**Temperature (ºC)**	19
		**Light intensity**	10–30 µmol photons m−2 s−1
**References Publicly Available Articles**	Sivonen K., et al. (1989) Toxicity Assess. 4, 339–352; Osswald et al. (2007) Environ. Int. 33, 1070–1089; Osswald et al. (2008) Chemosphere, 72(9), 1235-1241; Rantala-Ylinen, et al. (2011) Appl. Environm. Microbiol. 77(20), 7271-7278		
**References Publicly Available Theses**			

**Strain ID**	**Calenema singularis LEGE 06188**		
**Strain Taxonomy**	Calenema singularis Ramos, V; Brito, A & Kaštovský, J in Brito et al (2017)		
**Microphotograph 400×**	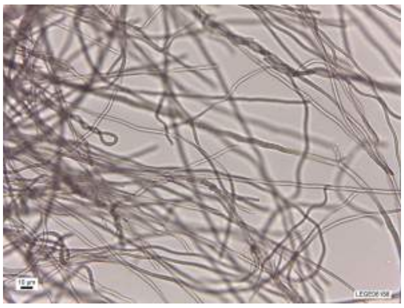	**Microphotograph 1000×**	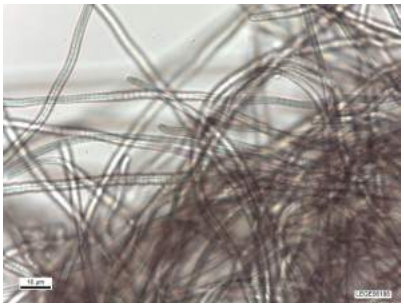
**Coidentity**	n.a.	**Morphometrics (Width × Length or Diameter) (µm)**	2,5±0,5 × 2,0±0,3
**Other Code(s)**	LEAN 153	**Taxonomic notes/diacritical features**	some helical filaments; often, the end of the trichome is distinctly hooked; with hormogonia and distinguishable centro- and chromatoplasm; with sheath;
**Parent Sample**	O.06 LAV p1 - AA	**Macroscopic growth features**	color: dark brown; forming tuft-like aggregates
**Isolation Date**		**Environment**	aquatic, marine
**Order (Older Classification Scheme)**	Oscillatoriales	**Habitat Sample Description**	sea water sample, coastal, surf zone
**Order 2014 (Modern Taxonomy)**	undefined	**Ecophysiological traits**	
**Taxonomy Notes**	This is the Type strain of Calenema singularis Ramos, Brito et Kaštovský gen. nov., sp. nov.	**Notes on ecophysiological traits**	
**Accession Number(s)_16S**	HQ832918	**Isolator**	Vitor Ramos
**Accession Number(s)_others (product/gene /region)**	KC256766 (dinitrogenase reductase, nifH), KC842318, KC842319 (polyketide synthase)	**Medium**	Z8 25‰ TM sea salt w/ vit. B12
**Collector**	Rui Seabra/Vitor Ramos	**Strain Status**	non-axenic, unicyanobacterial
**Collection Date**	2006/11/22	**Preservation type**	subculturing
**Location**	Portugal: Praia de Lavadores, Canidelo	**Light:dark cycle**	12:12-h
**Latitude & Longitude**	41.12919 N 8.668578 W	**Temperature (ºC)**	19
		**Light intensity**	10–30 µmol photons m−2 s−1
**References Publicly Available Articles**	Brito et al. (2012) Syst. Appl. Microbiol. 35, 110–119; Brito et al. (2015) Algal Res. 9, 218-226; Costa et al. (2016) Sci. Rep. 6, 23436; Brito et al. (2017) Mol. Phylog. Evol. doi:10.1016/j.ympev.2017.03.006		
**References Publicly Available Theses**			

**Strain ID**	**Calothrix sp. LEGE 06100**		
**Strain Taxonomy**	Calothrix Agardh, C ex Bornet, É & Flahault, C (1886)		
**Microphotograph 400×**	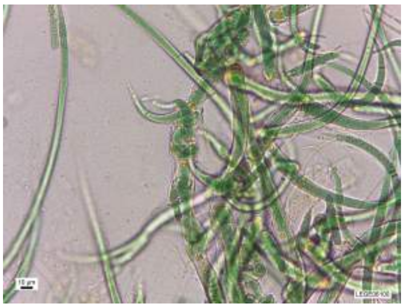	**Microphotograph 1000×**	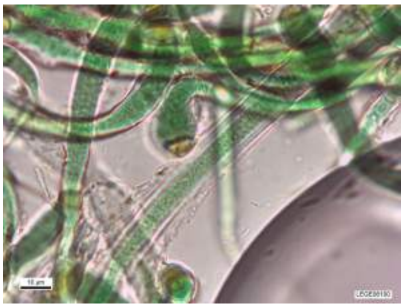
**Coidentity**	n.a.	**Morphometrics (Width × Length or Diameter) (µm)**	7,5±2,5 × 2,7±0,8 (Cells); Ø 5,0±0,9 (Heterocytes); Sheath up to 1,2±0,2
**Other Code(s)**	LEAN 004	**Taxonomic notes/diacritical features**	heteropolar trichomes; false-branching observed; with necridic cells and hormogonia; with sheath
**Parent Sample**	V.06 LAV 2 - AA	**Macroscopic growth features**	color: cyano; forming tuft-like aggregates
**Isolation Date**	2007/03/08	**Environment**	aquatic, marine
**Order (Older Classification Scheme)**	Nostocales	**Habitat Sample Description**	tide pool, wave-exposed rock, epilithic
**Order 2014 (Modern Taxonomy)**	Nostocales	**Ecophysiological traits**	nitrogen-fixation
**Taxonomy Notes**		**Notes on ecophysiological traits**	
**Accession Number(s)_16S**	HQ832913	**Isolator**	Vitor Ramos
**Accession Number(s)_others (product/gene /region)**	KC813180, KC813181 (non-ribosomal peptide synthetase), KC842315 (polyketide synthase)	**Medium**	MN w/ vit. B12
**Collector**	Rui Seabra	**Strain Status**	non-axenic, unicyanobacterial
**Collection Date**	2006/06/27	**Preservation type**	subculturing
**Location**	Portugal: Praia de Lavadores, Canidelo	**Light:dark cycle**	12:12-h
**Latitude & Longitude**	41.12919 N 8.668578 W	**Temperature (ºC)**	19
		**Light intensity**	10–30 µmol photons m−2 s−1
**References Publicly Available Articles**	Brito et al. (2012) Syst. Appl. Microbiol. 35, 110–119; Brito et al. (2015) Algal Res. 9, 218-226		
**References Publicly Available Theses**	Macieira R. (2013) MSc Dissertation, FCUP, University of Porto, Porto, Portugal		

**Strain ID**	**Calothrix sp. LEGE 06122**		
**Strain Taxonomy**	Calothrix Agardh, C ex Bornet, É & Flahault, C (1886)		
**Microphotograph 400×**	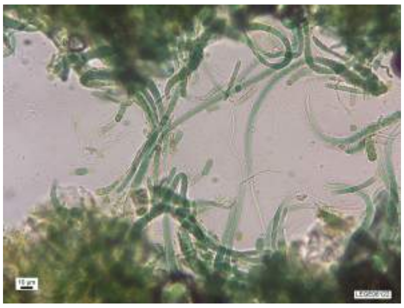	**Microphotograph 1000×**	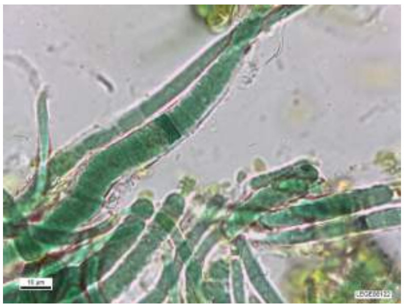
**Coidentity**	n.a.	**Morphometrics (Width × Length or Diameter) (µm)**	4,1±0,7 × 2,7±0,5 (Cells); Sheath up to 1,6±0,5
**Other Code(s)**	LEAN 028	**Taxonomic notes/diacritical features**	heteropolar trichomes; false-branching observed; with necridic cells and hormogonia; cell ultrastructure data available
**Parent Sample**	O.06 BAR 3 - BBEA	**Macroscopic growth features**	color: cyano; forming aggregates
**Isolation Date**	2007	**Environment**	aquatic, marine
**Order (Older Classification Scheme)**	Nostocales	**Habitat Sample Description**	wave-exposed tide pool, on a submerged stone
**Order 2014 (Modern Taxonomy)**	Nostocales	**Ecophysiological traits**	nitrogen-fixation
**Taxonomy Notes**		**Notes on ecophysiological traits**	
**Accession Number(s)_16S**	HQ832908	**Isolator**	Vitor Ramos
**Accession Number(s)_others (product/gene /region)**	KC842309-KC842311 (polyketide synthase)	**Medium**	MN w/ vit. B12
**Collector**	Vitor Ramos	**Strain Status**	non-axenic, unicyanobacterial
**Collection Date**	2006/11/21	**Preservation type**	subculturing
**Location**	Portugal: Praia de São Bartolomeu do Mar, Esposende	**Light:dark cycle**	12:12-h
**Latitude & Longitude**	41.57377 N 8.798558 W	**Temperature (ºC)**	19
		**Light intensity**	10–30 µmol photons m−2 s−1
**References Publicly Available Articles**	Brito et al. (2012) Syst. Appl. Microbiol. 35, 110–119; Brito et al. (2015) Algal Res. 9, 218-226		
**References Publicly Available Theses**			

**Strain ID**	**Chroococcidiopsis sp. LEGE 06174**		
**Strain Taxonomy**	Chroococcidiopsis Geitler, L (1933)		
**Microphotograph 400×**	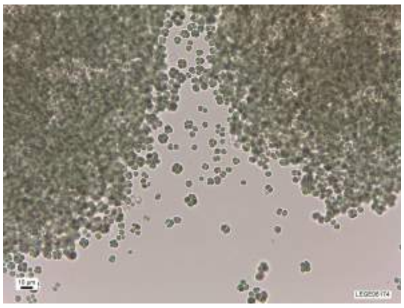	**Microphotograph 1000×**	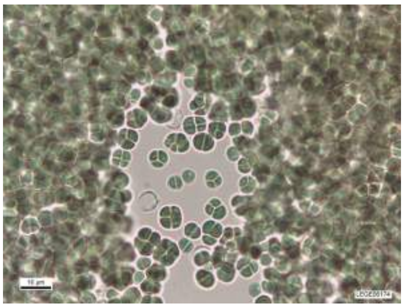
**Coidentity**	n.a.	**Morphometrics (Width × Length or Diameter) (µm)**	Ø 3,8±0,7 (Cells); Ø 7,4±0,8 (Group of 4 Cells)
**Other Code(s)**	LEAN 088	**Taxonomic notes/diacritical features**	mucilaginous envelope
**Parent Sample**	O.06 AGU p2 - C	**Macroscopic growth features**	color: olive green; forming aggregates
**Isolation Date**	2007	**Environment**	aquatic, marine
**Order (Older Classification Scheme)**	Chroococcales	**Habitat Sample Description**	sea water sample, coastal, surf zone
**Order 2014 (Modern Taxonomy)**	Pleurocapsales**	**Ecophysiological traits**	
**Taxonomy Notes**	**inconsistent; assignment supported by phylogeny	**Notes on ecophysiological traits**	abietic acid production (unknown ecophysiological role)
**Accession Number(s)_16S**	HQ832924	**Isolator**	Vitor Ramos
**Accession Number(s)_others (product/gene /region)**	KC842328, KC842329 (polyketide synthase)	**Medium**	Z8 25‰ TM sea salt w/ vit. B12
**Collector**	Rui Seabra/Vitor Ramos	**Strain Status**	non-axenic, unicyanobacterial
**Collection Date**	2006/11/22	**Preservation type**	subculturing
**Location**	Portugal: Praia da Aguda, Arcozelo	**Light:dark cycle**	12:12-h
**Latitude & Longitude**	41.04954 N 8.655339 W	**Temperature (ºC)**	19
		**Light intensity**	10–30 µmol photons m−2 s−1
**References Publicly Available Articles**	Brito et al. (2012) Syst. Appl. Microbiol. 35, 110–119; Rastoll et al. (2013) J. Appl. Phycol. 25, 1483-1493; Brito et al. (2015) Algal Res. 9, 218-226; Costa et al. (2016) Sci. Rep. 6, 23436		
**References Publicly Available Theses**			

Strain ID	**Chroococcopsis sp. LEGE 07168**		
**Strain Taxonomy**	Chroococcopsis Geitler, L (1925)		
**Microphotograph 400×**	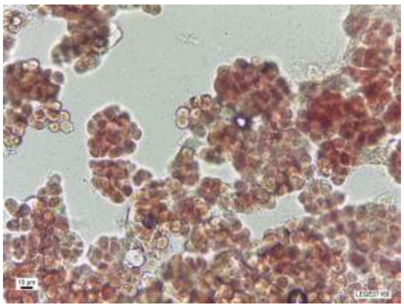	**Microphotograph 1000×**	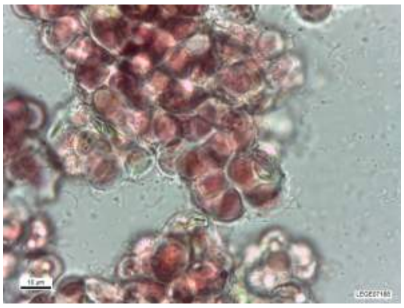
**Coidentity**	n.a.	**Morphometrics (Width × Length or Diameter) (µm)**	Ø 7,6±1,1 (Cells); Ø 46,8±16,1 (Group of Cells)
**Other Code(s)**	LEAN 082	**Taxonomic notes/diacritical features**	with baeocytes; mother cells divide by multiple fission
**Parent Sample**	P.07 LAV 1 - EA	**Macroscopic growth features**	color: brown-reddish; forming aggregates/colonies
**Isolation Date**		**Environment**	aquatic, marine
**Order (Older Classification Scheme)**	Pleurocapsales	**Habitat Sample Description**	intertidal zone, on a brown macroalga
**Order 2014 (Modern Taxonomy)**	Pleurocapsales	**Ecophysiological traits**	
**Taxonomy Notes**		**Notes on ecophysiological traits**	
**Accession Number(s)_16S**		**Isolator**	Vitor Ramos
**Accession Number(s)_others (product/gene /region)**		**Medium**	MN w/ vit. B12
**Collector**	Rui Seabra/Vitor Ramos	**Strain Status**	non-axenic, unicyanobacterial
**Collection Date**	2007/05/15	**Preservation type**	subculturing
**Location**	Portugal: Praia de Lavadores, Canidelo	**Light:dark cycle**	12:12-h
**Latitude & Longitude**	41.12919 N 8.668578 W	**Temperature (ºC)**	19
		**Light intensity**	10–30 µmol photons m−2 s−1
**References Publicly Available Articles**			
**References Publicly Available Theses**	Macieira R. (2013) MSc Dissertation, FCUP, University of Porto, Porto, Portugal		

Strain ID	**Chroococcopsis sp. LEGE 07187**		
**Strain Taxonomy**	Chroococcopsis Geitler, L (1925)		
**Microphotograph 400×**	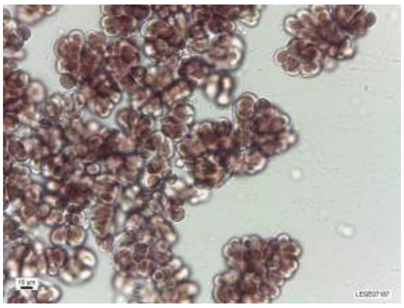	**Microphotograph 1000×**	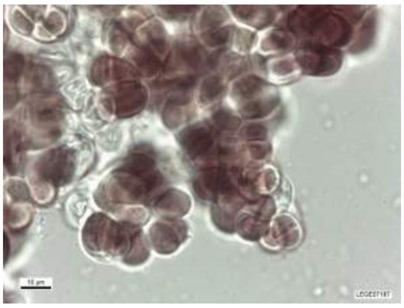
**Coidentity**	n.a.	**Morphometrics (Width × Length or Diameter) (µm)**	Ø 8,9±2,9 (Hemispherical Cells); Sheath up to 1,2±0,1
**Other Code(s)**	LEAN 151	**Taxonomic notes/diacritical features**	cell ultrastructure data available; with sheath
**Parent Sample**	P.07 MOL 3 - ABA	**Macroscopic growth features**	color: brownish; forming aggregates
**Isolation Date**	2007	**Environment**	aquatic, marine
**Order (Older Classification Scheme)**	Pleurocapsales	**Habitat Sample Description**	intertidal zone, on a Gibbula sp. shell
**Order 2014 (Modern Taxonomy)**	Pleurocapsales	**Ecophysiological traits**	
**Taxonomy Notes**		**Notes on ecophysiological traits**	
**Accession Number(s)_16S**	HQ832904	**Isolator**	Vitor Ramos
**Accession Number(s)_others (product/gene /region)**	KC842293, KC842294 (polyketide synthase)	**Medium**	MN w/ vit. B12
**Collector**	Rui Seabra/Vitor Ramos	**Strain Status**	non-axenic, unicyanobacterial
**Collection Date**	2007/05/21	**Preservation type**	subculturing, cryopreserved
**Location**	Portugal: Praia de Moledo, Caminha	**Light:dark cycle**	12:12-h
**Latitude & Longitude**	41.84963 N 8.866717 W	**Temperature (ºC)**	19
		**Light intensity**	10–30 µmol photons m−2 s−1
**References Publicly Available Articles**	Brito et al. (2012) Syst. Appl. Microbiol. 35, 110–119; Rastoll et al. (2013) J. Appl. Phycol. 25, 1483-1493; Brito et al. (2015) Algal Res. 9, 218-226		
**References Publicly Available Theses**			

**Strain ID**	**Chrysosporum ovalisporum LEGE X-001**		
**Strain Taxonomy**	Chrysosporum ovalisporum (Forti) Zapomelová, E; Skácelová, O; Pumann, P; Kopp, R & Janecek, E (2012)		
**Microphotograph 400×**	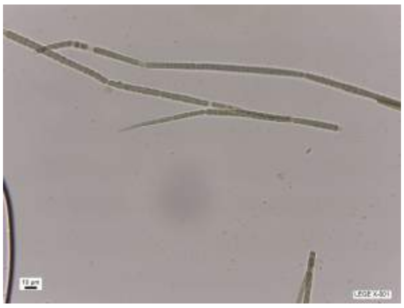	**Microphotograph 1000×**	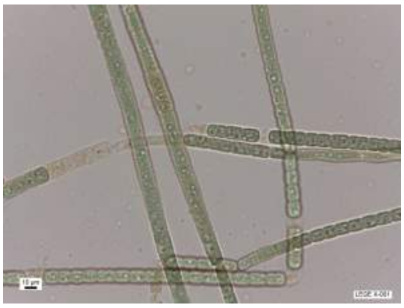
**Coidentity**	n.a.	**Morphometrics (Width × Length or Diameter) (µm)**	4,3±1,0 × 4,6±1,0
**Other Code(s)**	APH OVAL	**Taxonomic notes/diacritical features**	
**Parent Sample**		**Macroscopic growth features**	color: cyano; growing homogeneously, but also forming aggregates
**Isolation Date**		**Environment**	aquatic, freshwater
**Order (Older Classification Scheme)**	Nostocales	**Habitat Sample Description**	bloom sample
**Order 2014 (Modern Taxonomy)**	Nostocales	**Ecophysiological traits**	toxin production, nitrogen-fixation
**Taxonomy Notes**		**Notes on ecophysiological traits**	CYN producing strain
**Accession Number(s)_16S**		**Isolator**	Banker, et al. (1997)
**Accession Number(s)_others (product/gene /region)**		**Medium**	Z8
**Collector**	(Banker, et al. (1997)	**Strain Status**	non-axenic, unicyanobacterial
**Collection Date**	Fall 1994	**Preservation type**	subculturing
**Location**	Israel: Lake Kinnerett	**Light:dark cycle**	12:12-h
**Latitude & Longitude**	unknown	**Temperature (ºC)**	19
		**Light intensity**	10–30 µmol photons m−2 s−1
**References Publicly Available Articles**	Banker, et al. (1997) J. Phycol. 33: 613–616; Berry et al. (2009) Toxicon 53, 289–299; Leão et al. (2009) Eur. J. Phycol. 44, 347-355		
**References Publicly Available Theses**	Leão P.N. (2010) PhD Thesis, FCUP, University of Porto, Porto, Portugal		

**Strain ID**	**Coleofasciculus sp. LEGE 07081**		
**Strain Taxonomy**	Coleofasciculus Siegesmund, MA; Johansen, JR & Friedl, T (2008)		
**Microphotograph 400×**	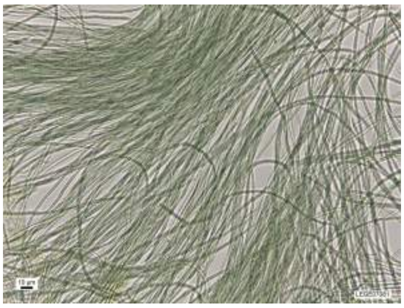	**Microphotograph 1000×**	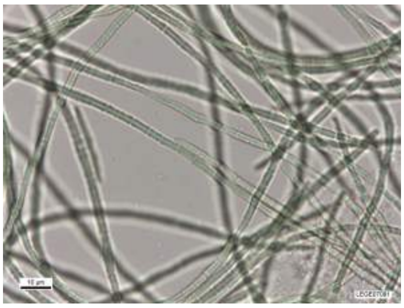
**Coidentity**	n.a.	**Morphometrics (Width × Length or Diameter) (µm)**	2,0±0,2 × 5,3±0,1
**Other Code(s)**		**Taxonomic notes/diacritical features**	apical cells rounded or conical-rounded
**Parent Sample**	VL 345	**Macroscopic growth features**	color: cyano; forming a firm biofilm
**Isolation Date**		**Environment**	aquatic, brackish
**Order (Older Classification Scheme)**	Oscillatoriales	**Habitat Sample Description**	mesotidal zone, benthic
**Order 2014 (Modern Taxonomy)**	Oscillatoriales	**Ecophysiological traits**	
**Taxonomy Notes**		**Notes on ecophysiological traits**	
**Accession Number(s)_16S**	HM217062	**Isolator**	Viviana Lopes
**Accession Number(s)_others (product/gene /region)**		**Medium**	Z8
**Collector**	Viviana Lopes	**Strain Status**	non-axenic, unicyanobacterial
**Collection Date**	2006/10/01	**Preservation type**	subculturing
**Location**	Portugal: Minho estuary, Caminha	**Light:dark cycle**	12:12-h
**Latitude & Longitude**	41.87933 N 8.838472 W	**Temperature (ºC)**	19
		**Light intensity**	10–30 µmol photons m−2 s−1
**References Publicly Available Articles**	Lopes et al. (2012) Mar. Environ. Res. 73, 7–16		
**References Publicly Available Theses**	Lopes V.R. (2011) PhD Thesis, FCUP, University of Porto, Porto, Portugal		

**Strain ID**	**Coleofasciculus sp. LEGE 07092**		
**Strain Taxonomy**	Coleofasciculus Siegesmund, MA; Johansen, JR & Friedl, T (2008)		
**Microphotograph 400×**	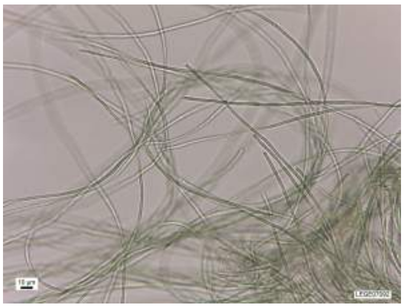	**Microphotograph 1000×**	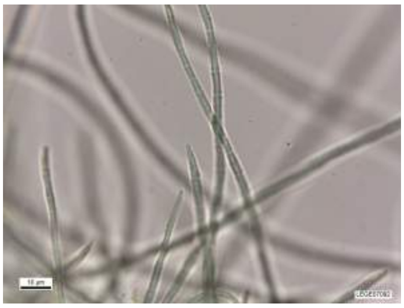
**Coidentity**	n.a.	**Morphometrics (Width × Length or Diameter) (µm)**	1,0±0,1 × 2,2±0,5
**Other Code(s)**		**Taxonomic notes/diacritical features**	terminal cells rounded or conical-rounded
**Parent Sample**	VL 343	**Macroscopic growth features**	color: cyano; forming a firm biofilm
**Isolation Date**		**Environment**	aquatic, brackish
**Order (Older Classification Scheme)**	Oscillatoriales	**Habitat Sample Description**	mesotidal zone, benthic
**Order 2014 (Modern Taxonomy)**	Oscillatoriales	**Ecophysiological traits**	
**Taxonomy Notes**		**Notes on ecophysiological traits**	
**Accession Number(s)_16S**	HM217070	**Isolator**	Viviana Lopes
**Accession Number(s)_others (product/gene /region)**		**Medium**	Z8
**Collector**	Viviana Lopes	**Strain Status**	non-axenic, unicyanobacterial
**Collection Date**	Oct-2007	**Preservation type**	subculturing
**Location**	Portugal: Vouga estuary, Ria de Aveiro, São Jacinto	**Light:dark cycle**	12:12-h
**Latitude & Longitude**	40.66644 N 8.725806 W	**Temperature (ºC)**	19
		**Light intensity**	10–30 µmol photons m−2 s−1
**References Publicly Available Articles**	Lopes et al. (2010) Mar. Drugs 8, 471-482; Baptista et al. (2011) Toxicon 58, 410–414; Lopes et al. (2011) Toxicol. In Vitro 25, 944–950; Lopes & Vasconcelos (2011) Mar. Drugs 9, 790-802; Cianca et al. (2012) Amino Acids 42, 2473–2479; Lopes et al. (2012) Mar. Environ. Res. 73, 7–16; Almeida et al. (2015) Toxins 7(8), 2739-2756; Baptista et al. (2015) Environ. Sci. Pollut. Res., 22, 12501-12510		
**References Publicly Available Theses**	Lopes V.R. (2011) PhD Thesis, FCUP, University of Porto, Porto, Portugal; Monteiro M.I. (2015) MSc Dissertation, FCUP, University of Porto, Porto, Portugal		

**Strain ID**	**Cuspidothrix issatschenkoi LEGE 00247**		
**Strain Taxonomy**	Cuspidothrix issatschenkoi (Usachev) Rajaniemi, P; Komárek, J; Willame, R; Hrouzek, P; Kastovská, K; Hoffmann, L & Sivonen, K (2005)		
**Microphotograph 400×**	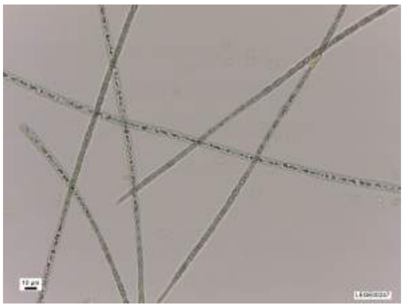	**Microphotograph 1000×**	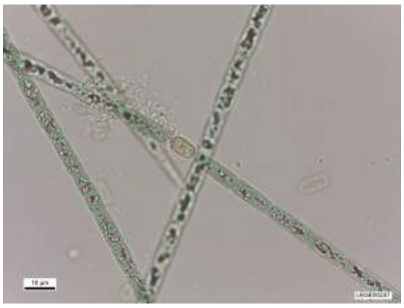
**Coidentity**	n.a.	**Morphometrics (Width × Length or Diameter) (µm)**	4,9±0,6 × 10,0±4,5 (cells); 4,8±0,5 × 8,0±0,9 (Heterocytes);; 7,9±0,9 × 16,9±2,5 (Akinetes)
**Other Code(s)**	J20, LEANJ.20	**Taxonomic notes/diacritical features**	trichomes with vacuolated cells and narrowed toward ends
**Parent Sample**		**Macroscopic growth features**	color: cyano; forming a smooth biofilm and aggregates
**Isolation Date**		**Environment**	aquatic, freshwater
**Order (Older Classification Scheme)**	Nostocales	**Habitat Sample Description**	water sample
**Order 2014 (Modern Taxonomy)**	Nostocales	**Ecophysiological traits**	toxin production
**Taxonomy Notes**		**Notes on ecophysiological traits**	ANTX-a producing strain
**Accession Number(s)_16S**	KU951674	**Isolator**	Joana Osswald
**Accession Number(s)_others (product/gene /region)**		**Medium**	Z8
**Collector**	Joana Oswald	**Strain Status**	non-axenic, unicyanobacterial
**Collection Date**	2000/07/07	**Preservation type**	subculturing
**Location**	Portugal: Maranhão dam reservoir, Benavila	**Light:dark cycle**	12:12-h
**Latitude & Longitude**	unknown	**Temperature (ºC)**	19
		**Light intensity**	10–30 µmol photons m−2 s−1
**References Publicly Available Articles**	Leão et al. (2009) Eur. J. Phycol. 44, 347-355		
**References Publicly Available Theses**	Osswald J. (2002) MSc Dissertation, FCUP, University of Porto, Porto, Portugal; Osswald J. (2007) PhD Thesis, FCUP, University of Porto, Porto, Portugal; Leão P.N. (2010) PhD Thesis, FCUP, University of Porto, Porto, Portugal		

**Strain ID**	**Cuspidothrix issatschenkoi LEGE 03282**		
**Strain Taxonomy**	Cuspidothrix issatschenkoi (Usachev) Rajaniemi, P; Komárek, J; Willame, R; Hrouzek, P; Kastovská, K; Hoffmann, L & Sivonen, K (2005)		
**Microphotograph 400×**	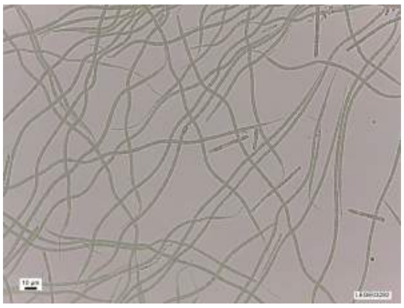	**Microphotograph 1000×**	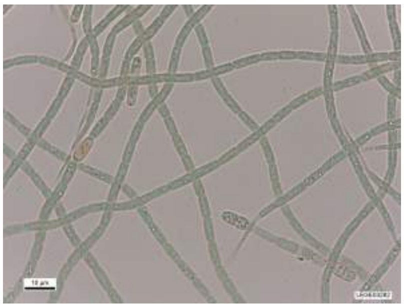
**Coidentity**	n.a.	**Morphometrics (Width × Length or Diameter) (µm)**	2,9±0,7 × 6,6±1,0 (Cells); 3,2±0,9 × 8,4±1,3 (akinetes)
**Other Code(s)**	J76, LEANJ.76	**Taxonomic notes/diacritical features**	with akinetes
**Parent Sample**		**Macroscopic growth features**	color: cyano; growing homogeneously
**Isolation Date**		**Environment**	aquatic, freshwater
**Order (Older Classification Scheme)**	Nostocales	**Habitat Sample Description**	water sample, dam reservoir
**Order 2014 (Modern Taxonomy)**	Nostocales	**Ecophysiological traits**	
**Taxonomy Notes**		**Notes on ecophysiological traits**	
**Accession Number(s)_16S**	KU951675	**Isolator**	Joana Osswald
**Accession Number(s)_others (product/gene /region)**		**Medium**	Z8
**Collector**	Joana Oswald	**Strain Status**	non-axenic, unicyanobacterial
**Collection Date**	2003/10/01	**Preservation type**	subculturing
**Location**	Portugal: Maranhão dam reservoir, campsite at Montargil	**Light:dark cycle**	12:12-h
**Latitude & Longitude**	unknown	**Temperature (ºC)**	19
		**Light intensity**	10–30 µmol photons m−2 s−1
**References Publicly Available Articles**			
**References Publicly Available Theses**			

**Strain ID**	**Cuspidothrix issatschenkoi LEGE 03284**		
**Strain Taxonomy**	Cuspidothrix issatschenkoi (Usachev) Rajaniemi, P; Komárek, J; Willame, R; Hrouzek, P; Kastovská, K; Hoffmann, L & Sivonen, K (2005)		
**Microphotograph 400×**	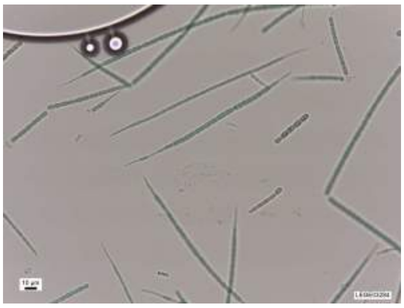	**Microphotograph 1000×**	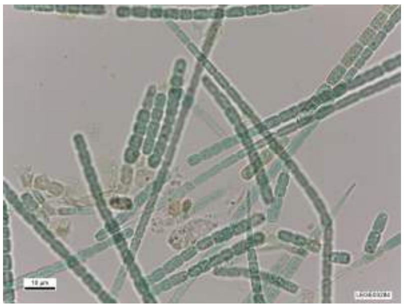
**Coidentity**	n.a.	**Morphometrics (Width × Length or Diameter) (µm)**	2,8±0,4 × 4,7±1,0 (Cells); 4,1±0,6 × 15,0±6,5 (akinetes)
**Other Code(s)**	J78, LEANJ.78	**Taxonomic notes/diacritical features**	trichomes narrowed toward ends
**Parent Sample**	n.d.	**Macroscopic growth features**	color: cyano; growing homogeneously
**Isolation Date**		**Environment**	aquatic, freshwater
**Order (Older Classification Scheme)**	Nostocales	**Habitat Sample Description**	water, dam reservoir
**Order 2014 (Modern Taxonomy)**	Nostocales	**Ecophysiological traits**	nitrogen-fixation
**Taxonomy Notes**		**Notes on ecophysiological traits**	
**Accession Number(s)_16S**	KC989703	**Isolator**	Joana Osswald
**Accession Number(s)_others (product/gene /region)**	KF008260 (cyanobactin, N-terminal protease (A))	**Medium**	Z8
**Collector**	Joana Oswald	**Strain Status**	non-axenic, unicyanobacterial
**Collection Date**	2003/10/03	**Preservation type**	subculturing
**Location**	Portugal: Maranhão dam reservoir, campsite at Montargil	**Light:dark cycle**	12:12-h
**Latitude & Longitude**	unknown	**Temperature (ºC)**	19
		**Light intensity**	10–30 µmol photons m−2 s−1
**References Publicly Available Articles**	Martins et al. (2013) Mar. Drugs 11 (12), 4902-4916		
**References Publicly Available Theses**	Macieira R. (2013) MSc Dissertation, FCUP, University of Porto, Porto, Portugal		

**Strain ID**	**Cuspidothrix issatschenkoi LEGE 03285**		
**Strain Taxonomy**	Cuspidothrix issatschenkoi (Usachev) Rajaniemi, P; Komárek, J; Willame, R; Hrouzek, P; Kastovská, K; Hoffmann, L & Sivonen, K (2005)		
**Microphotograph 400×**	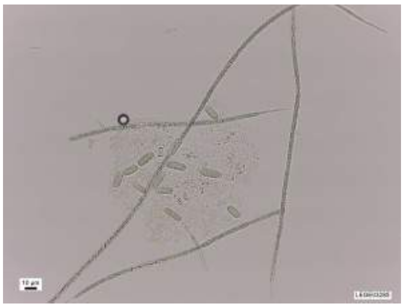	**Microphotograph 1000×**	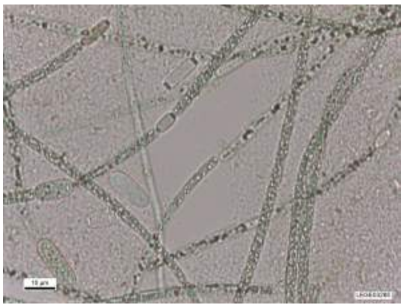
**Coidentity**	n.a.	**Morphometrics (Width × Length or Diameter) (µm)**	2,7±0,3 × 7,1±0,7 (Cells); 3,9±0,5 × 9,5±2,6 (Heterocytes); 6,2±0,9 × 15,0±2,7 (Akinetes)
**Other Code(s)**	J79, LEANJ.79	**Taxonomic notes/diacritical features**	trichomes narrowed toward ends; vacuolated cells
**Parent Sample**		**Macroscopic growth features**	color: cyano; forming tuft-like aggregates
**Isolation Date**		**Environment**	aquatic, freshwater
**Order (Older Classification Scheme)**	Nostocales	**Habitat Sample Description**	water sample
**Order 2014 (Modern Taxonomy)**	Nostocales	**Ecophysiological traits**	
**Taxonomy Notes**		**Notes on ecophysiological traits**	
**Accession Number(s)_16S**	KU951676	**Isolator**	Joana Osswald
**Accession Number(s)_others (product/gene /region)**		**Medium**	Z8
**Collector**	Joana Osswald	**Strain Status**	non-axenic, unicyanobacterial
**Collection Date**	2003/10/04	**Preservation type**	subculturing
**Location**	Portugal: Maranhão dam reservoir, campsite at Montargil	**Light:dark cycle**	12:12-h
**Latitude & Longitude**	unknown	**Temperature (ºC)**	19
		**Light intensity**	10–30 µmol photons m−2 s−1
**References Publicly Available Articles**			
**References Publicly Available Theses**			

Strain ID	**Cyanobacterium stanieri LEGE 03274**		
**Strain Taxonomy**	Cyanobacterium stanieri Rippka, R & Cohen-Bazire, G (1983)		
**Microphotograph 400×**	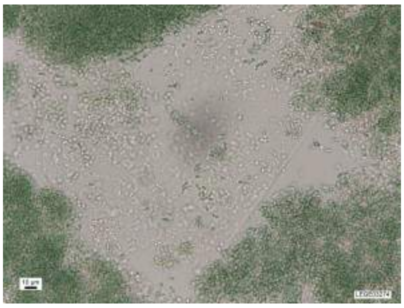	**Microphotograph 1000×**	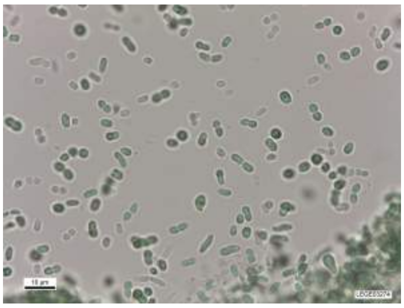
**Coidentity**	n.a.	**Morphometrics (Width × Length or Diameter) (µm)**	1,8±0,4 × 2,5±0,5
**Other Code(s)**	J70, LEANJ.70	**Taxonomic notes/diacritical features**	rod-shaped to widely oval cells
**Parent Sample**		**Macroscopic growth features**	color: cyano; forming aggregates
**Isolation Date**		**Environment**	aquatic, freshwater
**Order (Older Classification Scheme)**	Chroococcales	**Habitat Sample Description**	water sample
**Order 2014 (Modern Taxonomy)**	Chroococcales	**Ecophysiological traits**	
**Taxonomy Notes**		**Notes on ecophysiological traits**	
**Accession Number(s)_16S**	KU951677	**Isolator**	Joana Osswald
**Accession Number(s)_others (product/gene /region)**		**Medium**	Z8
**Collector**	Joana Oswald	**Strain Status**	non-axenic, unicyanobacterial
**Collection Date**	2003/09/26	**Preservation type**	subculturing
**Location**	Portugal: Lagoa dos Salgados, Albufeira	**Light:dark cycle**	12:12-h
**Latitude & Longitude**	unknown	**Temperature (ºC)**	19
		**Light intensity**	10–30 µmol photons m−2 s−1
**References Publicly Available Articles**			
**References Publicly Available Theses**			

Strain ID	**Cyanobium aff. gracile LEGE 07366**		
**Strain Taxonomy**	Cyanobium gracile Rippka, R & Cohen-Bazire, G (1983)		
**Microphotograph 400×**	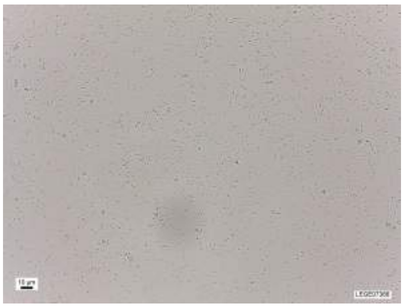	**Microphotograph 1000×**	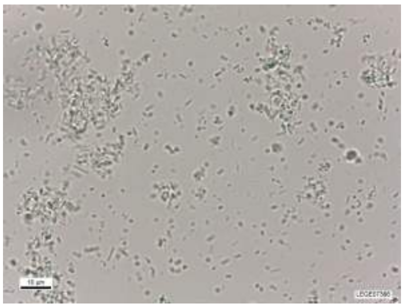
**Coidentity**	n.a.	**Morphometrics (Width × Length or Diameter) (µm)**	Ø 1,0±0,2 (Spherical Cells); 1,0±0,2×10,6±5,5 (Involution Cells); 1,0±0,2×1,4±0,3 (Elongated Cells)
**Other Code(s)**		**Taxonomic notes/diacritical features**	some elongated, involution cells observed
**Parent Sample**	VL MS 612 ou 6 12?	**Macroscopic growth features**	color: cyano; growing homogeneously
**Isolation Date**		**Environment**	aquatic, brackish
**Order (Older Classification Scheme)**	Chroococcales	**Habitat Sample Description**	unknown
**Order 2014 (Modern Taxonomy)**	Synechococcales	**Ecophysiological traits**	
**Taxonomy Notes**		**Notes on ecophysiological traits**	
**Accession Number(s)_16S**	KU951678	**Isolator**	Viviana Lopes
**Accession Number(s)_others (product/gene /region)**		**Medium**	Z8
**Collector**	Viviana Lopes	**Strain Status**	non-axenic, unicyanobacterial
**Collection Date**	2007	**Preservation type**	subculturing
**Location**	Portugal	**Light:dark cycle**	12:12-h
**Latitude & Longitude**	unknown	**Temperature (ºC)**	19
		**Light intensity**	10–30 µmol photons m−2 s−1
**References Publicly Available Articles**			
**References Publicly Available Theses**			

**Strain ID**	**Cyanobium gracile LEGE 00054**		
**Strain Taxonomy**	Cyanobium gracile Rippka, R & Cohen-Bazire, G (1983)		
**Microphotograph 400×**	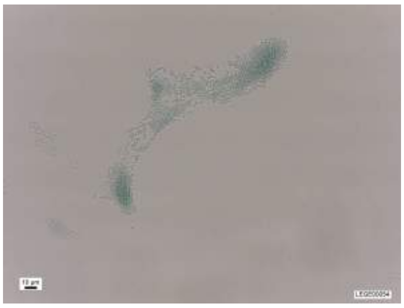	**Microphotograph 1000×**	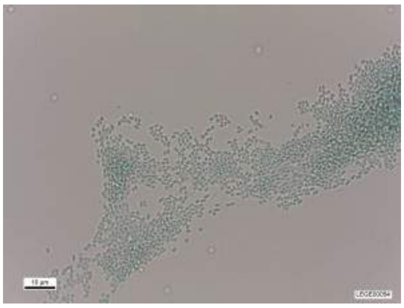
**Coidentity**	n.a.	**Morphometrics (Width × Length or Diameter) (µm)**	Ø 0,7±0,1 (Spherical Cells)
**Other Code(s)**		**Taxonomic notes/diacritical features**	
**Parent Sample**	Infout Pico	**Macroscopic growth features**	color: cyano; growing homogeneously
**Isolation Date**		**Environment**	aquatic, freshwater
**Order (Older Classification Scheme)**	Chroococcales	**Habitat Sample Description**	water sample
**Order 2014 (Modern Taxonomy)**	Synechococcales	**Ecophysiological traits**	
**Taxonomy Notes**		**Notes on ecophysiological traits**	
**Accession Number(s)_16S**	KU951679	**Isolator**	Martin Saker
**Accession Number(s)_others (product/gene /region)**		**Medium**	Z8
**Collector**	unknown	**Strain Status**	non-axenic, unicyanobacterial
**Collection Date**	2000	**Preservation type**	subculturing
**Location**	Morocco: Imfout reservoir, Oum Rabiaa basin	**Light:dark cycle**	12:12-h
**Latitude & Longitude**	unknown	**Temperature (ºC)**	19
		**Light intensity**	10–30 µmol photons m−2 s−1
**References Publicly Available Articles**			
**References Publicly Available Theses**			

**Strain ID**	**Cyanobium gracile LEGE 09399**		
**Strain Taxonomy**	Cyanobium gracile Rippka, R & Cohen-Bazire, G (1983)		
**Microphotograph 400×**	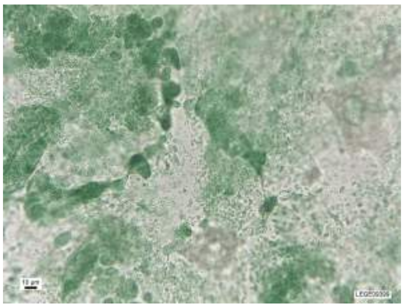	**Microphotograph 1000×**	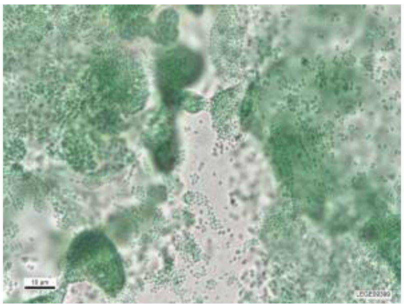
**Coidentity**	n.a.	**Morphometrics (Width × Length or Diameter) (µm)**	Ø 0,8±0,1
**Other Code(s)**		**Taxonomic notes/diacritical features**	several mucilaginous cell clusters
**Parent Sample**	Vela 1,2 um B	**Macroscopic growth features**	color: cyano; forming a smooth biofilm and aggregates
**Isolation Date**		**Environment**	aquatic, freshwater
**Order (Older Classification Scheme)**	Chroococcales	**Habitat Sample Description**	water sample, from a pond
**Order 2014 (Modern Taxonomy)**	Synechococcales	**Ecophysiological traits**	EPS production
**Taxonomy Notes**		**Notes on ecophysiological traits**	
**Accession Number(s)_16S**	KU951680	**Isolator**	Vitor Ramos
**Accession Number(s)_others (product/gene /region)**		**Medium**	Z8
**Collector**	António J. Martins	**Strain Status**	non-axenic, unicyanobacterial
**Collection Date**	2009	**Preservation type**	subculturing
**Location**	Portugal: Lagoa da Vela, Mira	**Light:dark cycle**	12:12-h
**Latitude & Longitude**	unknown	**Temperature (ºC)**	19
		**Light intensity**	10–30 µmol photons m−2 s−1
**References Publicly Available Articles**			
**References Publicly Available Theses**			

Strain ID	**Cyanobium gracile LEGE 12431**		
**Strain Taxonomy**	Cyanobium gracile Rippka, R & Cohen-Bazire, G (1983)		
**Microphotograph 400×**	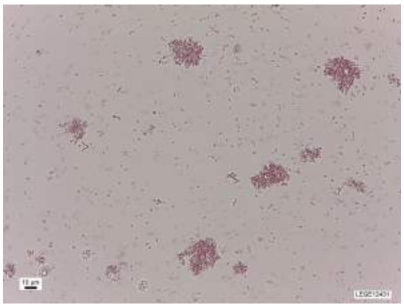	**Microphotograph 1000×**	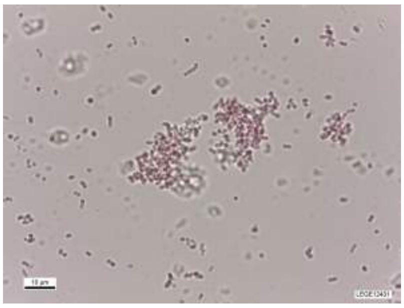
**Coidentity**	n.a.	**Morphometrics (Width × Length or Diameter) (µm)**	Ø 1,1±0,1 (Spherical Cells); 1,1±0,1 × 7,5±3,2 (Involution Cells); 1,1±0,1 ×1,6±0,3 (Elongated cells)
**Other Code(s)**		**Taxonomic notes/diacritical features**	some very elongated, involution cells observed
**Parent Sample**	LC5-BBA	**Macroscopic growth features**	color: reddish; forming aggregates
**Isolation Date**		**Environment**	aquatic, freshwater
**Order (Older Classification Scheme)**	Chroococcales	**Habitat Sample Description**	water sample
**Order 2014 (Modern Taxonomy)**	Synechococcales	**Ecophysiological traits**	chromatic adaptation
**Taxonomy Notes**		**Notes on ecophysiological traits**	
**Accession Number(s)_16S**	KU951681	**Isolator**	Vitor Ramos
**Accession Number(s)_others (product/gene /region)**		**Medium**	Z8
**Collector**	Jorge Nimptsch	**Strain Status**	non-axenic, unicyanobacterial
**Collection Date**	2012	**Preservation type**	subculturing, cryopreserved
**Location**	Chile: Caburgua Lake, La Araucania region	**Light:dark cycle**	12:12-h
**Latitude & Longitude**	unknown	**Temperature (ºC)**	19
**References Publicly Available Articles**		**Light intensity**	10–30 µmol photons m−2 s−1
**References Publicly Available Theses**			

**Strain ID**	**Cyanobium sp. LEGE 00034**		
**Strain Taxonomy**	Cyanobium Rippka, R & Cohen-Bazire, G (1983)		
**Microphotograph 400×**	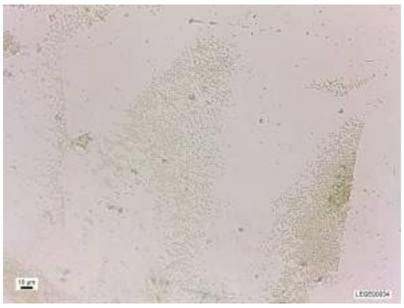	**Microphotograph 1000×**	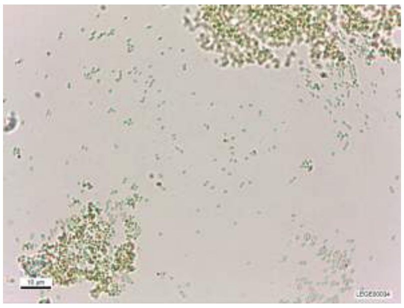
**Coidentity**	n.a.	**Morphometrics (Width × Length or Diameter) (µm)**	Ø 0,8±0,1
**Other Code(s)**	LEAN 208	**Taxonomic notes/diacritical features**	
**Parent Sample**	Aguda 13 (n)	**Macroscopic growth features**	color: cyano; forming aggregates
**Isolation Date**		**Environment**	aquatic, marine
**Order (Older Classification Scheme)**	Chroococcales	**Habitat Sample Description**	sea water sample, intertidal zone, from a tide pool
**Order 2014 (Modern Taxonomy)**	Synechococcales	**Ecophysiological traits**	
**Taxonomy Notes**		**Notes on ecophysiological traits**	
**Accession Number(s)_16S**	KU951682	**Isolator**	Rosario Martins
**Accession Number(s)_others (product/gene /region)**		**Medium**	Z8 25‰ TM sea salt w/ vit. B12
**Collector**	Rosario Martins	**Strain Status**	non-axenic, unicyanobacterial
**Collection Date**	2000/05/31	**Preservation type**	subculturing
**Location**	Portugal: Praia da Aguda, Arcozelo	**Light:dark cycle**	12:12-h
**Latitude & Longitude**	41.04954 N 8.655339 W	**Temperature (ºC)**	19
		**Light intensity**	10–30 µmol photons m−2 s−1
**References Publicly Available Articles**			
**References Publicly Available Theses**			

Strain ID	**Cyanobium sp. LEGE 00035**		
**Strain Taxonomy**	Cyanobium Rippka, R & Cohen-Bazire, G (1983)		
**Microphotograph 400×**	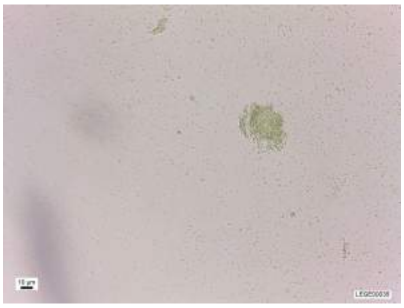	**Microphotograph 1000×**	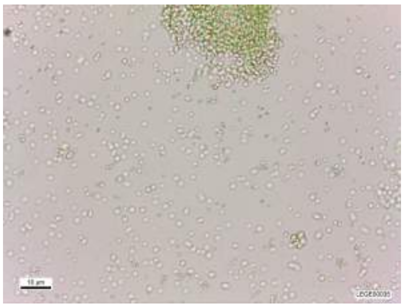
**Coidentity**	n.a.	**Morphometrics (Width × Length or Diameter) (µm)**	Ø 0,8±0,1
**Other Code(s)**	LEAN 209	**Taxonomic notes/diacritical features**	
**Parent Sample**	Olhos d'Água	**Macroscopic growth features**	color: cyano; growing homogeneously; mucilaginous
**Isolation Date**		**Environment**	aquatic, marine
**Order (Older Classification Scheme)**	Chroococcales	**Habitat Sample Description**	sea water sample, intertidal zone, from a tide pool
**Order 2014 (Modern Taxonomy)**	Synechococcales	**Ecophysiological traits**	EPS production
**Taxonomy Notes**		**Notes on ecophysiological traits**	
**Accession Number(s)_16S**	KU951683	**Isolator**	Rosario Martins
**Accession Number(s)_others (product/gene /region)**		**Medium**	Z8 25‰ TM sea salt w/ vit. B12
**Collector**	Rosario Martins	**Strain Status**	non-axenic, unicyanobacterial
**Collection Date**	2000/09/01	**Preservation type**	subculturing
**Location**	Portugal: Praia de Olhos d'Água, Albufeira	**Light:dark cycle**	12:12-h
**Latitude & Longitude**	37.08973 N 8.191017 W	**Temperature (ºC)**	19
		**Light intensity**	10–30 µmol photons m−2 s−1
**References Publicly Available Articles**			
**References Publicly Available Theses**			

**Strain ID**	**Cyanobium sp. LEGE 06002**		
**Strain Taxonomy**	Cyanobium Rippka, R & Cohen-Bazire, G (1983)		
**Microphotograph 400×**	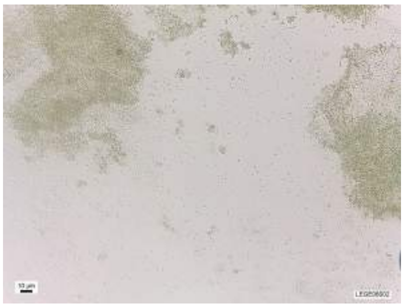	**Microphotograph 1000×**	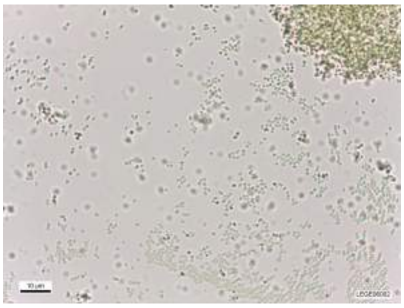
**Coidentity**	n.a.	**Morphometrics (Width × Length or Diameter) (µm)**	0,9±0,2 × 1,0±0,1
**Other Code(s)**	LEAN 123	**Taxonomic notes/diacritical features**	
**Parent Sample**	7 Buarcos	**Macroscopic growth features**	color: cyano; forming aggregates
**Isolation Date**		**Environment**	aquatic, marine
**Order (Older Classification Scheme)**	Chroococcales	**Habitat Sample Description**	intertidal zone
**Order 2014 (Modern Taxonomy)**	Synechococcales	**Ecophysiological traits**	
**Taxonomy Notes**		**Notes on ecophysiological traits**	
**Accession Number(s)_16S**	KU951684	**Isolator**	Bárbara Frazão
**Accession Number(s)_others (product/gene /region)**		**Medium**	Z8 25‰ TM sea salt w/ vit. B12
**Collector**	Bárbara Frazão	**Strain Status**	non-axenic, unicyanobacterial
**Collection Date**	Fall 2006	**Preservation type**	subculturing
**Location**	Portugal: Praia de Buarcos	**Light:dark cycle**	12:12-h
**Latitude & Longitude**	40.15621 N 8.871803 W	**Temperature (ºC)**	19
		**Light intensity**	10–30 µmol photons m−2 s−1
**References Publicly Available Articles**			
**References Publicly Available Theses**			

Strain ID	**Cyanobium sp. LEGE 06004**		
**Strain Taxonomy**	Cyanobium Rippka, R & Cohen-Bazire, G (1983)		
**Microphotograph 400×**	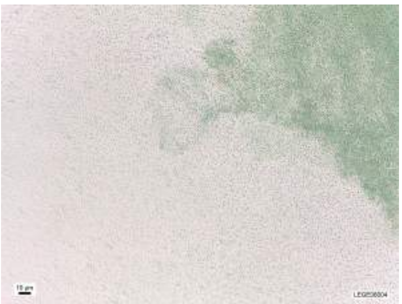	**Microphotograph 1000×**	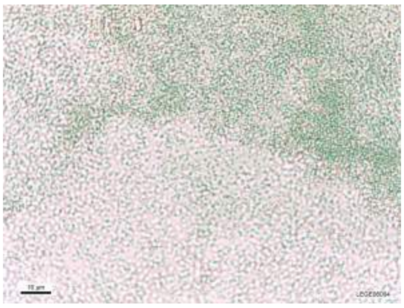
**Coidentity**	n.a.	**Morphometrics (Width × Length or Diameter) (µm)**	0,7±0,1 × 0,8±0,1
**Other Code(s)**	LEAN 125	**Taxonomic notes/diacritical features**	
**Parent Sample**	8 Buarcos	**Macroscopic growth features**	color: cyano; growing homogeneously, but also forming aggregates
**Isolation Date**		**Environment**	aquatic, marine
**Order (Older Classification Scheme)**	Chroococcales	**Habitat Sample Description**	unknown
**Order 2014 (Modern Taxonomy)**	Synechococcales	**Ecophysiological traits**	
**Taxonomy Notes**		**Notes on ecophysiological traits**	
**Accession Number(s)_16S**		**Isolator**	Bárbara Frazão
**Accession Number(s)_others (product/gene /region)**		**Medium**	Z8 25‰ TM sea salt w/ vit. B12
**Collector**	Bárbara Frazão	**Strain Status**	non-axenic, unicyanobacterial
**Collection Date**	Fall 2006	**Preservation type**	subculturing
**Location**	Portugal: Praia de Buarcos	**Light:dark cycle**	12:12-h
**Latitude & Longitude**	40.15621 N 8.871803 W	**Temperature (ºC)**	19
		**Light intensity**	10–30 µmol photons m−2 s−1
**References Publicly Available Articles**			
**References Publicly Available Theses**			

**Strain ID**	**Cyanobium sp. LEGE 06008**		
**Strain Taxonomy**	Cyanobium Rippka, R & Cohen-Bazire, G (1983)		
**Microphotograph 400×**	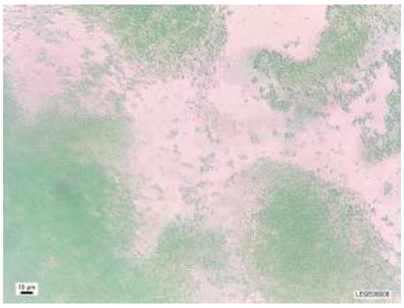	**Microphotograph 1000×**	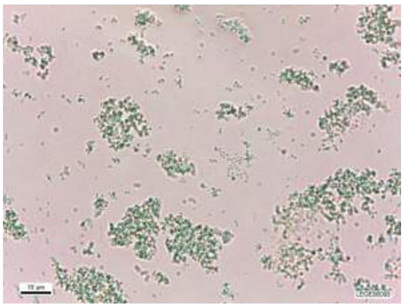
**Coidentity**	n.a.	**Morphometrics (Width × Length or Diameter) (µm)**	Ø 0,8±0,2
**Other Code(s)**	strain 11, LEAN 104	**Taxonomic notes/diacritical features**	
**Parent Sample**	Ref. 11	**Macroscopic growth features**	color: cyano; forming aggregates
**Isolation Date**		**Environment**	aquatic, marine
**Order (Older Classification Scheme)**	Chroococcales	**Habitat Sample Description**	intertidal zone, wave-exposed rock
**Order 2014 (Modern Taxonomy)**	Synechococcales	**Ecophysiological traits**	
**Taxonomy Notes**		**Notes on ecophysiological traits**	
**Accession Number(s)_16S**	KU951685	**Isolator**	Bárbara Frazão
**Accession Number(s)_others (product/gene /region)**		**Medium**	Z8 25‰ TM sea salt w/ vit. B12
**Collector**	Bárbara Frazão	**Strain Status**	non-axenic, unicyanobacterial
**Collection Date**	Fall 2006	**Preservation type**	subculturing
**Location**	Portugal: Praia da Foz do Arelho, Caldas da Rainha	**Light:dark cycle**	12:12-h
**Latitude & Longitude**	39.43327 N 9.230275 W	**Temperature (ºC)**	19
		**Light intensity**	10–30 µmol photons m−2 s−1
**References Publicly Available Articles**	Frazão et al. (2010) Mar. Drugs 8, 1908-1919		
**References Publicly Available Theses**	Frazão B. (2008) MSc Dissertation, FCUP, University of Porto, Porto, Portugal		

**Strain ID**	**Cyanobium sp. LEGE 06011**		
**Strain Taxonomy**	Cyanobium Rippka, R & Cohen-Bazire, G (1983)		
**Microphotograph 400×**	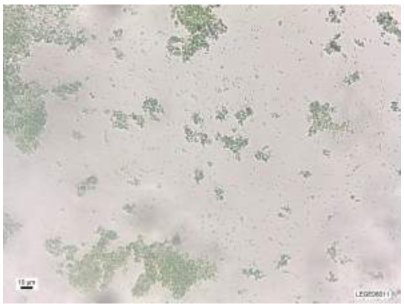	**Microphotograph 1000×**	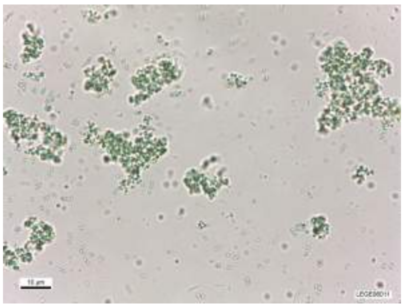
**Coidentity**	LEGE 06012	**Morphometrics (Width × Length or Diameter) (µm)**	Ø 1,0±0,2
**Other Code(s)**	strain 4, LEAN 108	**Taxonomic notes/diacritical features**	facultative sheath
**Parent Sample**	Ref. 4	**Macroscopic growth features**	color: cyano; forming aggregates
**Isolation Date**		**Environment**	aquatic, marine
**Order (Older Classification Scheme)**	Chroococcales	**Habitat Sample Description**	intertidal zone, wave-exposed rock
**Order 2014 (Modern Taxonomy)**	Synechococcales	**Ecophysiological traits**	
**Taxonomy Notes**		**Notes on ecophysiological traits**	
**Accession Number(s)_16S**	KU951686	**Isolator**	Bárbara Frazão
**Accession Number(s)_others (product/gene /region)**		**Medium**	Z8 25‰ TM sea salt w/ vit. B12
**Collector**	Bárbara Frazão	**Strain Status**	non-axenic, unicyanobacterial
**Collection Date**	Fall 2006	**Preservation type**	subculturing
**Location**	Portugal: Praia da Foz do Arelho, Caldas da Rainha	**Light:dark cycle**	12:12-h
**Latitude & Longitude**	39.43327 N 9.230275 W	**Temperature (ºC)**	19
		**Light intensity**	10–30 µmol photons m−2 s−1
**References Publicly Available Articles**	Frazão et al. (2010) Mar. Drugs 8, 1908-1919; Rastoll et al. (2013) J. Appl. Phycol. 25, 1483-1493		
**References Publicly Available Theses**	Frazão B. (2008) MSc Dissertation, FCUP, University of Porto, Porto, Portugal		

Strain ID	**Cyanobium sp. LEGE 06012**		
**Strain Taxonomy**	Cyanobium Rippka, R & Cohen-Bazire, G (1983)		
**Microphotograph 400×**	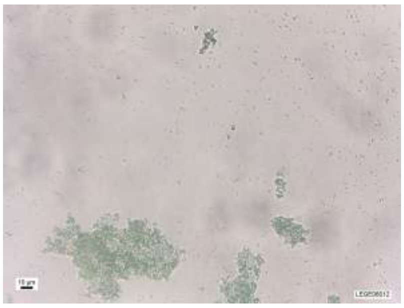	**Microphotograph 1000×**	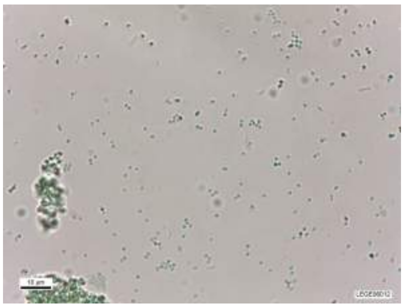
**Coidentity**	LEGE 06011	**Morphometrics (Width × Length or Diameter) (µm)**	Ø 0,9±0,2
**Other Code(s)**	strain 4, LEAN 114	**Taxonomic notes/diacritical features**	
**Parent Sample**	Ref. 4	**Macroscopic growth features**	color: cyano; forming aggregates
**Isolation Date**		**Environment**	aquatic, marine
**Order (Older Classification Scheme)**	Chroococcales	**Habitat Sample Description**	intertidal zone, wave-exposed rock
**Order 2014 (Modern Taxonomy)**	Synechococcales	**Ecophysiological traits**	
**Taxonomy Notes**		**Notes on ecophysiological traits**	
**Accession Number(s)_16S**	KU951687	**Isolator**	Bárbara Frazão
**Accession Number(s)_others (product/gene /region)**	KC842333 (polyketide synthase)	**Medium**	Z8 25‰ TM sea salt w/ vit. B12
**Collector**	Bárbara Frazão	**Strain Status**	non-axenic, unicyanobacterial
**Collection Date**	Fall 2006	**Preservation type**	subculturing
**Location**	Portugal: Praia da Foz do Arelho, Caldas da Rainha	**Light:dark cycle**	12:12-h
**Latitude & Longitude**	39.43327 N 9.230275 W	**Temperature (ºC)**	19
		**Light intensity**	10–30 µmol photons m−2 s−1
**References Publicly Available Articles**	Brito et al. (2012) Syst. Appl. Microbiol. 35, 110–119; Brito et al. (2015) Algal Res. 9, 218-226		
**References Publicly Available Theses**	Frazão B. (2008) MSc Dissertation, FCUP, University of Porto, Porto, Portugal		

**Strain ID**	**Cyanobium sp. LEGE 06015**		
**Strain Taxonomy**	Cyanobium Rippka, R & Cohen-Bazire, G (1983)		
**Microphotograph 400×**	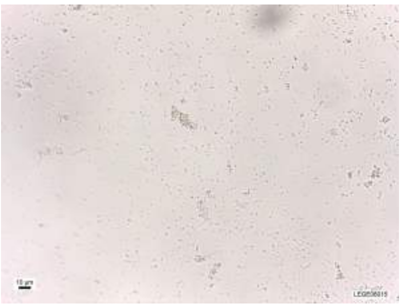	**Microphotograph 1000×**	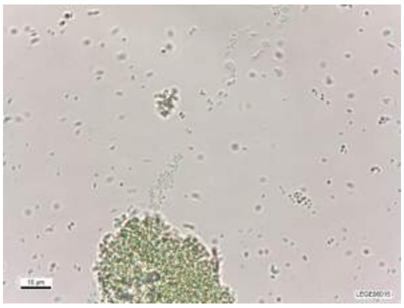
**Coidentity**	n.a.	**Morphometrics (Width × Length or Diameter) (µm)**	Ø 0,8±0,2
**Other Code(s)**	strain 7, LEAN 102	**Taxonomic notes/diacritical features**	
**Parent Sample**	Ref. 7	**Macroscopic growth features**	color: cyano; growing homogeneously, but also forming aggregates
**Isolation Date**		**Environment**	aquatic, marine
**Order (Older Classification Scheme)**	Chroococcales	**Habitat Sample Description**	intertidal zone, on a Patella sp. shell
**Order 2014 (Modern Taxonomy)**	Synechococcales	**Ecophysiological traits**	
**Taxonomy Notes**		**Notes on ecophysiological traits**	
**Accession Number(s)_16S**	KU951688	**Isolator**	Bárbara Frazão
**Accession Number(s)_others (product/gene /region)**		**Medium**	Z8 25‰ TM sea salt w/ vit. B12
**Collector**	Bárbara Frazão	**Strain Status**	non-axenic, unicyanobacterial
**Collection Date**	Fall 2006	**Preservation type**	subculturing
**Location**	Portugal: Baleal Norte, Ferrel	**Light:dark cycle**	12:12-h
**Latitude & Longitude**	39.37417 N 9.338722 W	**Temperature (ºC)**	19
		**Light intensity**	10–30 µmol photons m−2 s−1
**References Publicly Available Articles**	Frazão et al. (2010) Mar. Drugs 8, 1908-1919; Rastoll et al. (2013) J. Appl. Phycol. 25, 1483-1493		
**References Publicly Available Theses**	Frazão B. (2008) MSc Dissertation, FCUP, University of Porto, Porto, Portugal		

**Strain ID**	**Cyanobium sp. LEGE 06016**		
**Strain Taxonomy**	Cyanobium Rippka, R & Cohen-Bazire, G (1983)		
**Microphotograph 400×**	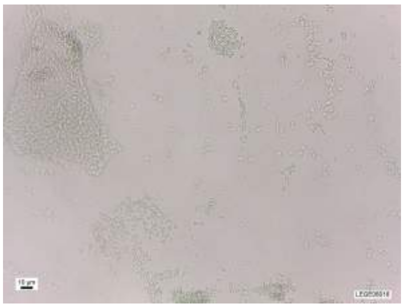	**Microphotograph 1000×**	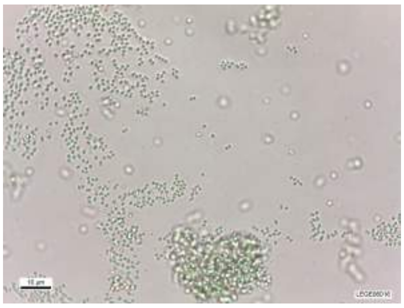
**Coidentity**	n.a.	**Morphometrics (Width × Length or Diameter) (µm)**	Ø 0,9±0,1
**Other Code(s)**	strain 22, LEAN 122	**Taxonomic notes/diacritical features**	
**Parent Sample**	Ref. 22, Baleal planktonic	**Macroscopic growth features**	color: cyano; growing homogeneously
**Isolation Date**		**Environment**	aquatic, marine
**Order (Older Classification Scheme)**	Chroococcales	**Habitat Sample Description**	sea water sample, coastal, surf zone
**Order 2014 (Modern Taxonomy)**	Synechococcales	**Ecophysiological traits**	
**Taxonomy Notes**		**Notes on ecophysiological traits**	
**Accession Number(s)_16S**		**Isolator**	Bárbara Frazão
**Accession Number(s)_others (product/gene /region)**		**Medium**	Z8 25‰ TM sea salt w/ vit. B12
**Collector**	Bárbara Frazão	**Strain Status**	non-axenic, unicyanobacterial
**Collection Date**	Fall 2006	**Preservation type**	subculturing
**Location**	Portugal: Baleal Norte, Ferrel	**Light:dark cycle**	12:12-h
**Latitude & Longitude**	39.37417 N 9.338722 W	**Temperature (ºC)**	19
		**Light intensity**	10–30 µmol photons m−2 s−1
**References Publicly Available Articles**			
**References Publicly Available Theses**			

**Strain ID**	**Cyanobium sp. LEGE 06023**		
**Strain Taxonomy**	Cyanobium Rippka, R & Cohen-Bazire, G (1983)		
**Microphotograph 400×**	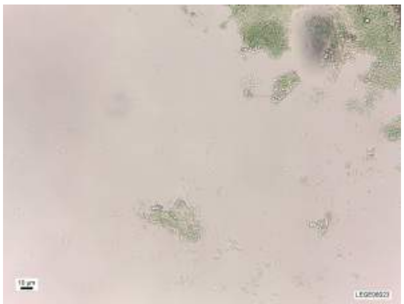	**Microphotograph 1000×**	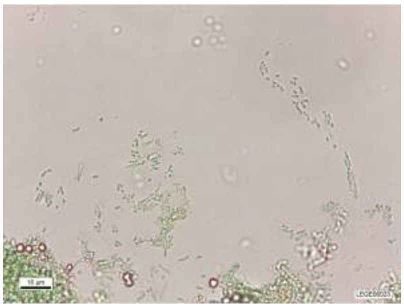
**Coidentity**	n.a.	**Morphometrics (Width × Length or Diameter) (µm)**	Ø 0,8±0,2
**Other Code(s)**	strain 12, LEAN 110	**Taxonomic notes/diacritical features**	
**Parent Sample**	Ref. 12	**Macroscopic growth features**	color: cyano; forming aggregates
**Isolation Date**		**Environment**	aquatic, marine
**Order (Older Classification Scheme)**	Chroococcales	**Habitat Sample Description**	intertidal zone, wave-exposed rock
**Order 2014 (Modern Taxonomy)**	Synechococcales	**Ecophysiological traits**	
**Taxonomy Notes**		**Notes on ecophysiological traits**	
**Accession Number(s)_16S**	KU951689	**Isolator**	Bárbara Frazão
**Accession Number(s)_others (product/gene /region)**		**Medium**	Z8 25‰ TM sea salt w/ vit. B12
**Collector**	Bárbara Frazão	**Strain Status**	non-axenic, unicyanobacterial
**Collection Date**	Fall 2006	**Preservation type**	subculturing
**Location**	Portugal: Praia da Empa, Ericeira	**Light:dark cycle**	12:12-h
**Latitude & Longitude**	38.98079 N 9.421944 W	**Temperature (ºC)**	19
		**Light intensity**	10–30 µmol photons m−2 s−1
**References Publicly Available Articles**			
**References Publicly Available Theses**	Frazão B. (2008) MSc Dissertation, FCUP, University of Porto, Porto, Portugal		

**Strain ID**	**Cyanobium sp. LEGE 06024**		
**Strain Taxonomy**	Cyanobium Rippka, R & Cohen-Bazire, G (1983)		
**Microphotograph 400×**	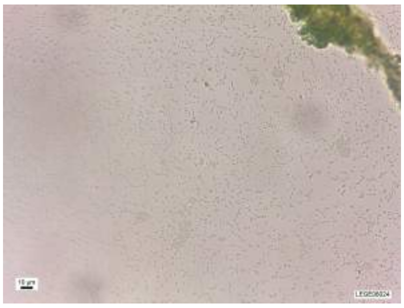	**Microphotograph 1000×**	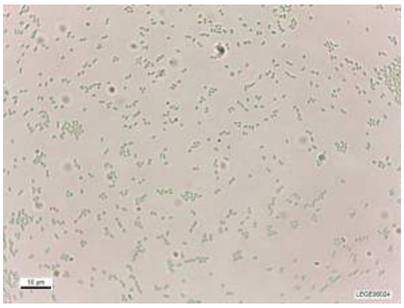
**Coidentity**	n.a.	**Morphometrics (Width × Length or Diameter) (µm)**	Ø 0,8±0,1
**Other Code(s)**	strain 2, LEAN 112	**Taxonomic notes/diacritical features**	
**Parent Sample**	Ref. 2	**Macroscopic growth features**	color: cyano; growing homogeneously, but also forming aggregates
**Isolation Date**		**Environment**	aquatic, marine
**Order (Older Classification Scheme)**	Chroococcales	**Habitat Sample Description**	intertidal zone, wave-sheltered rock
**Order 2014 (Modern Taxonomy)**	Synechococcales	**Ecophysiological traits**	
**Taxonomy Notes**		**Notes on ecophysiological traits**	
**Accession Number(s)_16S**	KU951690	**Isolator**	Bárbara Frazão
**Accession Number(s)_others (product/gene /region)**		**Medium**	Z8 25‰ TM sea salt w/ vit. B12
**Collector**	Bárbara Frazão	**Strain Status**	non-axenic, unicyanobacterial
**Collection Date**	Fall 2006	**Preservation type**	subculturing
**Location**	Portugal: Praia da Empa, Ericeira	**Light:dark cycle**	12:12-h
**Latitude & Longitude**	38.98079 N 9.421944 W	**Temperature (ºC)**	19
		**Light intensity**	10–30 µmol photons m−2 s−1
**References Publicly Available Articles**	Rastoll et al. (2013) J. Appl. Phycol. 25, 1483-1493		
**References Publicly Available Theses**	Frazão B. (2008) MSc Dissertation, FCUP, University of Porto, Porto, Portugal		

**Strain ID**	**Cyanobium sp. LEGE 06026**		
**Strain Taxonomy**	Cyanobium Rippka, R & Cohen-Bazire, G (1983)		
**Microphotograph 400×**	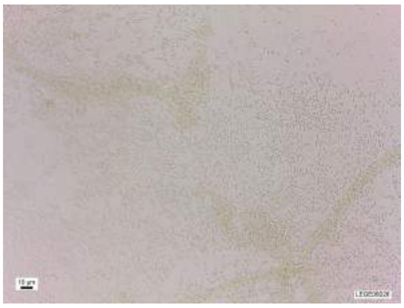	**Microphotograph 1000×**	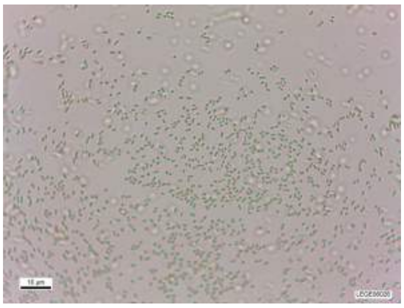
**Coidentity**	n.a.	**Morphometrics (Width × Length or Diameter) (µm)**	Ø 0,7±0,1
**Other Code(s)**	strain 9, LEAN 117	**Taxonomic notes/diacritical features**	
**Parent Sample**	Ref. 9	**Macroscopic growth features**	color: cyano; growing homogeneously; mucilaginous
**Isolation Date**		**Environment**	aquatic, marine
**Order (Older Classification Scheme)**	Chroococcales	**Habitat Sample Description**	intertidal zone, wave-sheltered rock
**Order 2014 (Modern Taxonomy)**	Synechococcales	**Ecophysiological traits**	EPS production
**Taxonomy Notes**		**Notes on ecophysiological traits**	
**Accession Number(s)_16S**	KU951691	**Isolator**	Bárbara Frazão
**Accession Number(s)_others (product/gene /region)**		**Medium**	Z8 25‰ TM sea salt w/ vit. B12
**Collector**	Bárbara Frazão	**Strain Status**	non-axenic, unicyanobacterial
**Collection Date**	Fall 2006	**Preservation type**	subculturing, cryopreserved
**Location**	Portugal: Praia da Empa, Ericeira	**Light:dark cycle**	12:12-h
**Latitude & Longitude**	38.98079 N 9.421944 W	**Temperature (ºC)**	19
		**Light intensity**	10–30 µmol photons m−2 s−1
**References Publicly Available Articles**	Costa et al. (2014) Mar. Drugs 12, 98-114		
**References Publicly Available Theses**	Frazão B. (2008) MSc Dissertation, FCUP, University of Porto, Porto, Portugal		

**Strain ID**	**Cyanobium sp. LEGE 06068**		
**Strain Taxonomy**	Cyanobium Rippka, R & Cohen-Bazire, G (1983)		
**Microphotograph 400×**	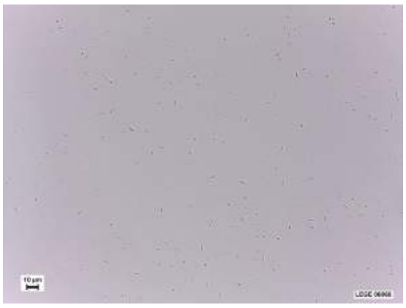	**Microphotograph 1000×**	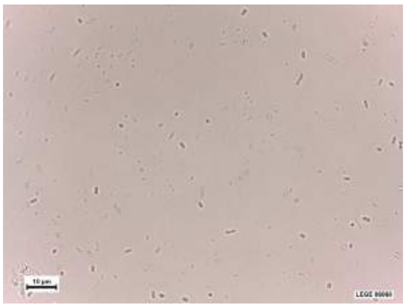
**Coidentity**	n.a.	**Morphometrics (Width × Length or Diameter) (µm)**	Ø 0,8±0,2
**Other Code(s)**	15	**Taxonomic notes/diacritical features**	
**Parent Sample**	n.d.	**Macroscopic growth features**	color: cyano; growing homogeneously
**Isolation Date**		**Environment**	aquatic, brackish
**Order (Older Classification Scheme)**	Chroococcales	**Habitat Sample Description**	mesotidal zone, benthic
**Order 2014 (Modern Taxonomy)**	Synechococcales	**Ecophysiological traits**	
**Taxonomy Notes**		**Notes on ecophysiological traits**	
**Accession Number(s)_16S**	HM217069	**Isolator**	Viviana Lopes
**Accession Number(s)_others (product/gene /region)**		**Medium**	Z8
**Collector**	Viviana Lopes	**Strain Status**	non-axenic, unicyanobacterial
**Collection Date**	2006/10/11	**Preservation type**	subculturing
**Location**	Portugal: Douro estuary, Porto	**Light:dark cycle**	12:12-h
**Latitude & Longitude**	41.14792 N 8.651611 W	**Temperature (ºC)**	19
		**Light intensity**	10–30 µmol photons m−2 s−1
**References Publicly Available Articles**	Lopes et al. (2010) Mar. Drugs 8, 471-482; Baptista et al. (2011) Toxicon 58, 410–414; Lopes et al. (2011) Toxicol. In Vitro 25, 944–950; Cianca et al. (2012) Amino Acids 42, 2473–2479; Lopes et al. (2012) Mar. Environ. Res. 73, 7–16; Almeida et al. (2015) Toxins 7(8), 2739-2756		
**References Publicly Available Theses**	Lopes V.R. (2011) PhD Thesis, FCUP, University of Porto, Porto, Portugal		

**Strain ID**	**Cyanobium sp. LEGE 06097**		
**Strain Taxonomy**	Cyanobium Rippka, R & Cohen-Bazire, G (1983)		
**Microphotograph 400×**	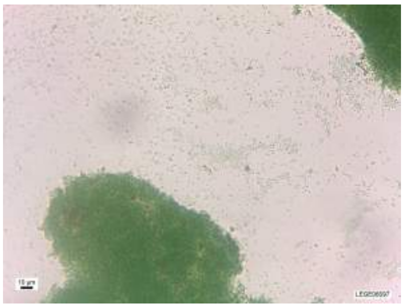	**Microphotograph 1000×**	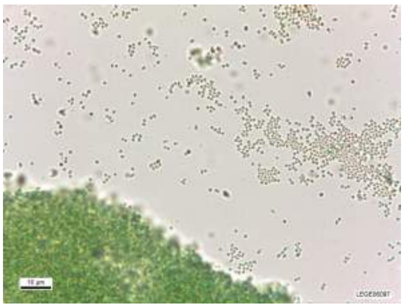
**Coidentity**	n.a.	**Morphometrics (Width × Length or Diameter) (µm)**	Ø 1,1±0,1
**Other Code(s)**	LEAN 001	**Taxonomic notes/diacritical features**	cell ultrastructure data available
**Parent Sample**	V.06 MAR 5 - BA	**Macroscopic growth features**	color: cyano; forming a smooth biofilm
**Isolation Date**	2007/03/01	**Environment**	aquatic, marine
**Order (Older Classification Scheme)**	Chroococcales	**Habitat Sample Description**	intertidal zone, on a green macroalga
**Order 2014 (Modern Taxonomy)**	Synechococcales	**Ecophysiological traits**	
**Taxonomy Notes**		**Notes on ecophysiological traits**	
**Accession Number(s)_16S**	KU951692	**Isolator**	Vitor Ramos
**Accession Number(s)_others (product/gene /region)**	KC842336 (polyketide synthase)	**Medium**	Z8 25‰ TM sea salt w/ vit. B12
**Collector**	Rui Seabra	**Strain Status**	non-axenic, unicyanobacterial
**Collection Date**	2006/07/16	**Preservation type**	subculturing
**Location**	Portugal: Praia do Martinhal, Vila do Bispo	**Light:dark cycle**	12:12-h
**Latitude & Longitude**	37.01869 N 8.926714 W	**Temperature (ºC)**	19
		**Light intensity**	10–30 µmol photons m−2 s−1
**References Publicly Available Articles**	Brito et al. (2012) Syst. Appl. Microbiol. 35, 110–119; Costa et al. (2014) Mar. Drugs 12, 98-114; Brito et al. (2015) Algal Res. 9, 218-226		
**References Publicly Available Theses**			

**Strain ID**	**Cyanobium sp. LEGE 06098**		
**Strain Taxonomy**	Cyanobium Rippka, R & Cohen-Bazire, G (1983)		
**Microphotograph 400×**	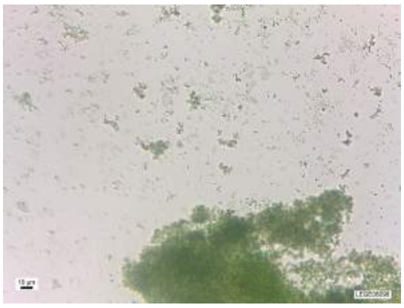	**Microphotograph 1000×**	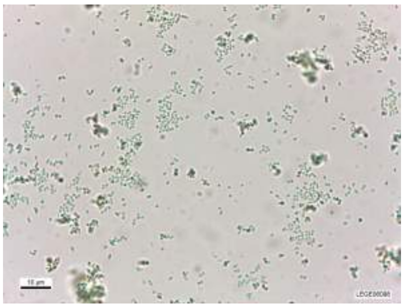
**Coidentity**	n.a.	**Morphometrics (Width × Length or Diameter) (µm)**	Ø 0,9±0,2
**Other Code(s)**	LEAN 002	**Taxonomic notes/diacritical features**	
**Parent Sample**	V.06 MAR 5 - BBA	**Macroscopic growth features**	color: cyano; forming a smooth biofilm
**Isolation Date**	2007/03/23	**Environment**	aquatic, marine
**Order (Older Classification Scheme)**	Chroococcales	**Habitat Sample Description**	intertidal zone, on a green macroalga
**Order 2014 (Modern Taxonomy)**	Synechococcales	**Ecophysiological traits**	
**Taxonomy Notes**		**Notes on ecophysiological traits**	
**Accession Number(s)_16S**	KC469572	**Isolator**	Vitor Ramos
**Accession Number(s)_others (product/gene /region)**	KC813175 (non-ribosomal peptide synthetase)	**Medium**	Z8 25‰ TM sea salt w/ vit. B12
**Collector**	Rui Seabra	**Strain Status**	non-axenic, unicyanobacterial
**Collection Date**	2006/07/16	**Preservation type**	subculturing
**Location**	Portugal: Praia do Martinhal, Vila do Bispo	**Light:dark cycle**	12:12-h
**Latitude & Longitude**	37.01869 N 8.926714 W	**Temperature (ºC)**	19
		**Light intensity**	10–30 µmol photons m−2 s−1
**References Publicly Available Articles**	Costa et al. (2015) J. Toxicol. Env. Heal. A 78, 432-442; Costa et al. (2014) Mar. Drugs 12, 98-114; Brito et al. (2015) Algal Res. 9, 218-226		
**References Publicly Available Theses**	Costa M.S. (2011) MSc Dissertation, ICBAS and FCUP, University of Porto, Porto, Portugal; Ribeiro M.J. (2012) MSc Dissertation, Department of Chemistry, University of Aveiro, Aveiro, Portugal;		

**Strain ID**	**Cyanobium sp. LEGE 06109**		
**Strain Taxonomy**	Cyanobium Rippka, R & Cohen-Bazire, G (1983)		
**Microphotograph 400×**	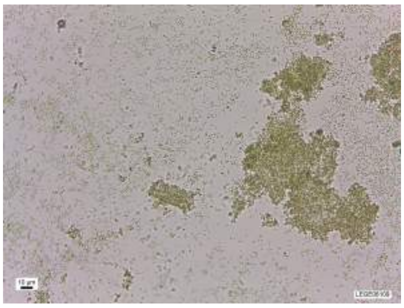	**Microphotograph 1000×**	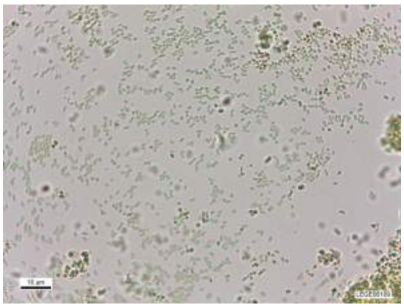
**Coidentity**	n.a.	**Morphometrics (Width × Length or Diameter) (µm)**	Ø 0,9±0,1
**Other Code(s)**	LEAN 014	**Taxonomic notes/diacritical features**	
**Parent Sample**	V.06 AGU 6 - GA	**Macroscopic growth features**	color: cyano; forming aggregates
**Isolation Date**	2007/03/01	**Environment**	aquatic, marine
**Order (Older Classification Scheme)**	Chroococcales	**Habitat Sample Description**	intertidal zone, Sabellaria sp. reef, epipsamic
**Order 2014 (Modern Taxonomy)**	Synechococcales	**Ecophysiological traits**	
**Taxonomy Notes**		**Notes on ecophysiological traits**	
**Accession Number(s)_16S**	HQ832920	**Isolator**	Vitor Ramos
**Accession Number(s)_others (product/gene /region)**	KC813182 (non-ribosomal peptide synthetase), KC842325-KC842325 (polyketide synthase)	**Medium**	Z8 25‰ TM sea salt w/ vit. B12
**Collector**	Rui Seabra	**Strain Status**	non-axenic, unicyanobacterial
**Collection Date**	2006/06/26	**Preservation type**	subculturing
**Location**	Portugal: Praia da Aguda, Arcozelo	**Light:dark cycle**	12:12-h
**Latitude & Longitude**	41.04954 N 8.655339 W	**Temperature (ºC)**	19
		**Light intensity**	10–30 µmol photons m−2 s−1
**References Publicly Available Articles**	Brito et al. (2012) Syst. Appl. Microbiol. 35, 110–119; Brito et al. (2015) Algal Res. 9, 218-226		
**References Publicly Available Theses**			

Strain ID	**Cyanobium sp. LEGE 06113**		
**Strain Taxonomy**	Cyanobium Rippka, R & Cohen-Bazire, G (1983)		
**Microphotograph 400×**	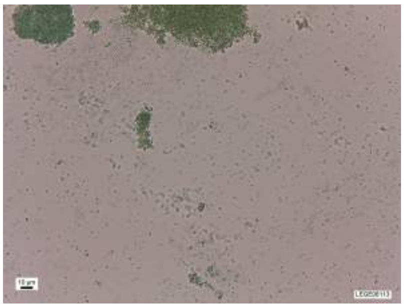	**Microphotograph 1000×**	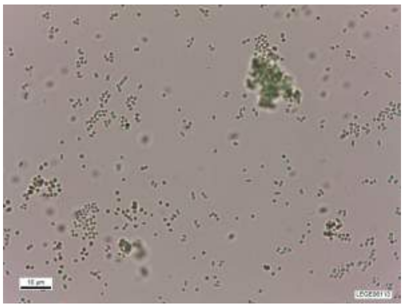
**Coidentity**	n.a.	**Morphometrics (Width × Length or Diameter) (µm)**	Ø 1,2±0,2
**Other Code(s)**	LEAN 018	**Taxonomic notes/diacritical features**	
**Parent Sample**	V.06 AGU 12 - CA	**Macroscopic growth features**	color: cyano; forming aggregates
**Isolation Date**	2007/03/08	**Environment**	aquatic, marine
**Order (Older Classification Scheme)**	Chroococcales	**Habitat Sample Description**	intertidal zone, Sabellaria sp. reef, epipsamic
**Order 2014 (Modern Taxonomy)**	Synechococcales	**Ecophysiological traits**	
**Taxonomy Notes**		**Notes on ecophysiological traits**	hierridin B production (unknown ecophysiological role)
**Accession Number(s)_16S**	KC469577	**Isolator**	Vitor Ramos
**Accession Number(s)_others (product/gene /region)**	KC842298 (polyketide synthase), KF010866 (polyketide synthase, ketosynthase domain)	**Medium**	Z8 25‰ TM sea salt w/ vit. B12
**Collector**	Rui Seabra	**Strain Status**	non-axenic, unicyanobacterial
**Collection Date**	2006/06/26	**Preservation type**	subculturing
**Location**	Portugal: Praia da Aguda, Arcozelo	**Light:dark cycle**	12:12-h
**Latitude & Longitude**	41.04954 N 8.655339 W	**Temperature (ºC)**	19
		**Light intensity**	10–30 µmol photons m−2 s−1
**References Publicly Available Articles**	Leão et al. (2013) PLoS ONE 8 (7), e69562; Costa et al. (2014) Mar. Drugs 12, 98-114; Brito et al. (2015) Algal Res. 9, 218-226		
**References Publicly Available Theses**	Ribeiro M.J. (2012) MSc Dissertation, Department of Chemistry, University of Aveiro, Aveiro, Portugal; Freitas S. (2013) MSc Dissertation, ICBAS, University of Porto, Porto, Portugal		

**Strain ID**	**Cyanobium sp. LEGE 06127**		
**Strain Taxonomy**	Cyanobium Rippka, R & Cohen-Bazire, G (1983)		
**Microphotograph 400×**	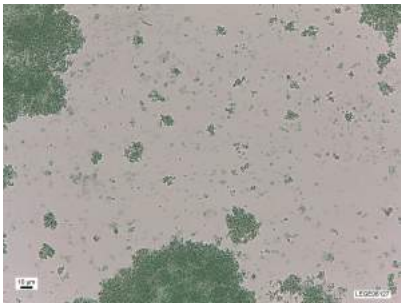	**Microphotograph 1000×**	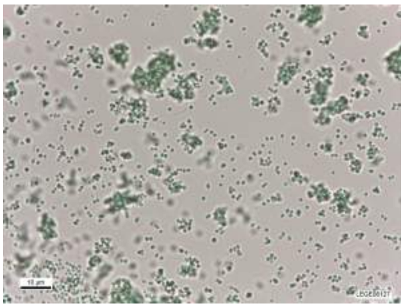
**Coidentity**	n.a.	**Morphometrics (Width × Length or Diameter) (µm)**	Ø 1,1±0,1
**Other Code(s)**	LEAN 032	**Taxonomic notes/diacritical features**	several cell clusters
**Parent Sample**	O.06 BUR 5 - CA	**Macroscopic growth features**	color: cyano; forming aggregates
**Isolation Date**	2007/06/18	**Environment**	aquatic, marine
**Order (Older Classification Scheme)**	Chroococcales	**Habitat Sample Description**	intertidal zone, Sabellaria alveolata reef, epipsamic
**Order 2014 (Modern Taxonomy)**	Synechococcales	**Ecophysiological traits**	
**Taxonomy Notes**		**Notes on ecophysiological traits**	
**Accession Number(s)_16S**	KU951693	**Isolator**	Vitor Ramos
**Accession Number(s)_others (product/gene /region)**		**Medium**	Z8 25‰ TM sea salt w/ vit. B12
**Collector**	Rui Seabra	**Strain Status**	non-axenic, unicyanobacterial
**Collection Date**	2006/12/11	**Preservation type**	subculturing
**Location**	Portugal: Praia de Burgau, Budens	**Light:dark cycle**	12:12-h
**Latitude & Longitude**	37.07156 N 8.774722 W	**Temperature (ºC)**	19
		**Light intensity**	10–30 µmol photons m−2 s−1
**References Publicly Available Articles**	Brito et al. (2012) Syst. Appl. Microbiol. 35, 110–119		
**References Publicly Available Theses**			

**Strain ID**	**Cyanobium sp. LEGE 06130**		
**Strain Taxonomy**	Cyanobium Rippka, R & Cohen-Bazire, G (1983)		
**Microphotograph 400×**	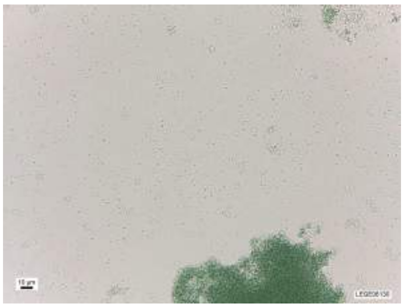	**Microphotograph 1000×**	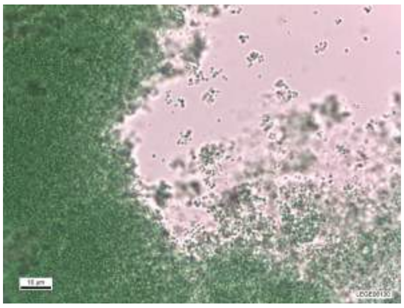
**Coidentity**	n.a.	**Morphometrics (Width × Length or Diameter) (µm)**	Ø 1,0±0,1
**Other Code(s)**	LEAN 038	**Taxonomic notes/diacritical features**	
**Parent Sample**	V.06 ODA XX - ACAA	**Macroscopic growth features**	color: cyano; forming aggregates
**Isolation Date**	2007/06/22	**Environment**	aquatic, marine
**Order (Older Classification Scheme)**	Chroococcales	**Habitat Sample Description**	wave-exposed tide puddle, epilithic
**Order 2014 (Modern Taxonomy)**	Synechococcales	**Ecophysiological traits**	
**Taxonomy Notes**		**Notes on ecophysiological traits**	
**Accession Number(s)_16S**	KU951694	**Isolator**	Vitor Ramos
**Accession Number(s)_others (product/gene /region)**	KC842364, KC842365 (polyketide synthase)	**Medium**	Z8 25‰ TM sea salt w/ vit. B12
**Collector**	Rui Seabra	**Strain Status**	non-axenic, unicyanobacterial
**Collection Date**	2006/12/12	**Preservation type**	subculturing
**Location**	Portugal: Praia de Olhos d'Água, Albufeira	**Light:dark cycle**	12:12-h
**Latitude & Longitude**	37.08973 N 8.191017 W	**Temperature (ºC)**	19
		**Light intensity**	10–30 µmol photons m−2 s−1
**References Publicly Available Articles**	Brito et al. (2012) Syst. Appl. Microbiol. 35, 110–119; Brito et al. (2015) Algal Res. 9, 218-226		
**References Publicly Available Theses**			

**Strain ID**	**Cyanobium sp. LEGE 06134**		
**Strain Taxonomy**	Cyanobium Rippka, R & Cohen-Bazire, G (1983)		
**Microphotograph 400×**	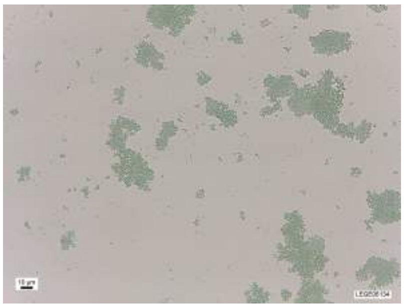	**Microphotograph 1000×**	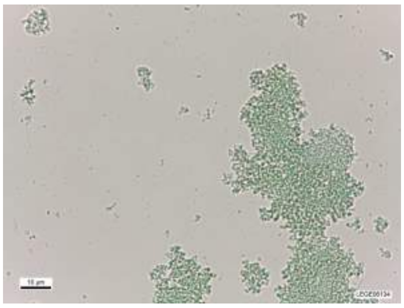
**Coidentity**	n.a.	**Morphometrics (Width × Length or Diameter) (µm)**	Ø 0,7±0,1
**Other Code(s)**	LEAN 043	**Taxonomic notes/diacritical features**	several cell clusters
**Parent Sample**	V.06 MOL 12 - ACA	**Macroscopic growth features**	color: cyano; growing homogeneously, but also forming aggregates
**Isolation Date**	2007/06/22	**Environment**	aquatic, marine
**Order (Older Classification Scheme)**	Chroococcales	**Habitat Sample Description**	intertidal zone, Sabellaria sp. reef, epipsamic
**Order 2014 (Modern Taxonomy)**	Synechococcales	**Ecophysiological traits**	
**Taxonomy Notes**		**Notes on ecophysiological traits**	
**Accession Number(s)_16S**	KC469573	**Isolator**	Vitor Ramos
**Accession Number(s)_others (product/gene /region)**	KC842299 (polyketide synthase)	**Medium**	Z8 25‰ TM sea salt w/ vit. B12
**Collector**	Rui Seabra	**Strain Status**	non-axenic, unicyanobacterial
**Collection Date**	2006/06/30	**Preservation type**	subculturing
**Location**	Portugal: Praia de Moledo, Caminha	**Light:dark cycle**	12:12-h
**Latitude & Longitude**	41.84963 N 8.866717 W	**Temperature (ºC)**	19
		**Light intensity**	10–30 µmol photons m−2 s−1
**References Publicly Available Articles**	Costa et al. (2014) Mar. Drugs 12, 98-114; Costa et al. (2015) J. Toxicol. Env. Heal. A 78, 432-442; Brito et al. (2015) Algal Res. 9, 218-226		
**References Publicly Available Theses**	Costa M.S. (2011) MSc Dissertation, ICBAS and FCUP, University of Porto, Porto, Portugal		

Strain ID	**Cyanobium sp. LEGE 06135**		
**Strain Taxonomy**	Cyanobium Rippka, R & Cohen-Bazire, G (1983)		
**Microphotograph 400×**	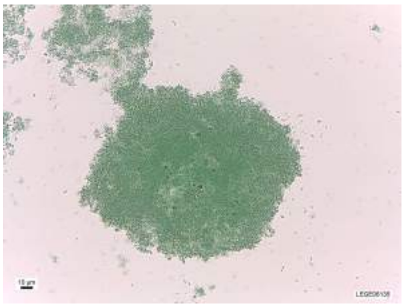	**Microphotograph 1000×**	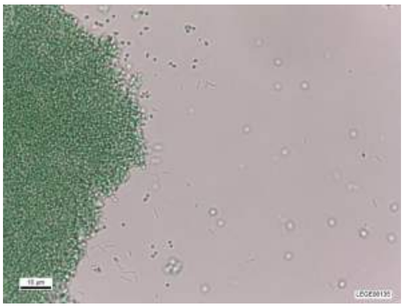
**Coidentity**	n.a.	**Morphometrics (Width × Length or Diameter) (µm)**	Ø 1,2±0,2
**Other Code(s)**	LEAN 045	**Taxonomic notes/diacritical features**	several cell clusters
**Parent Sample**	V.06 BAR 8 - ABA	**Macroscopic growth features**	color: cyano; forming aggregates
**Isolation Date**		**Environment**	aquatic, marine
**Order (Older Classification Scheme)**	Chroococcales	**Habitat Sample Description**	intertidal zone, Sabellaria alveolata reef, epipsamic
**Order 2014 (Modern Taxonomy)**	Synechococcales	**Ecophysiological traits**	
**Taxonomy Notes**		**Notes on ecophysiological traits**	
**Accession Number(s)_16S**	KU951695	**Isolator**	Vitor Ramos
**Accession Number(s)_others (product/gene /region)**		**Medium**	Z8 25‰ TM sea salt w/ vit. B12
**Collector**	Rui Seabra	**Strain Status**	non-axenic, unicyanobacterial
**Collection Date**	2006/06/28	**Preservation type**	subculturing, cryopreserved
**Location**	Portugal: Praia de São Bartolomeu do Mar, Esposende	**Light:dark cycle**	12:12-h
**Latitude & Longitude**	41.57377 N 8.798558 W	**Temperature (ºC)**	19
		**Light intensity**	10–30 µmol photons m−2 s−1
**References Publicly Available Articles**			
**References Publicly Available Theses**			

**Strain ID**	**Cyanobium sp. LEGE 06137**		
**Strain Taxonomy**	Cyanobium Rippka, R & Cohen-Bazire, G (1983)		
**Microphotograph 400×**	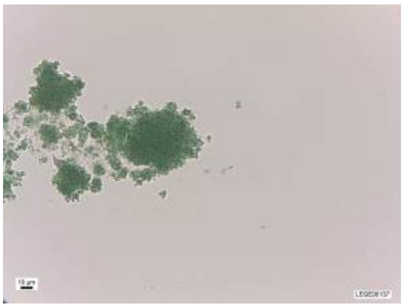	**Microphotograph 1000×**	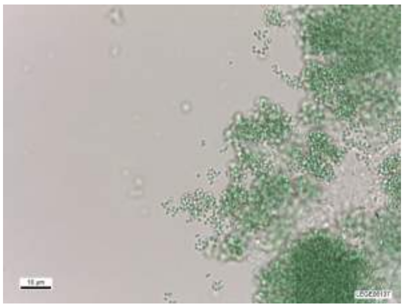
**Coidentity**	n.a.	**Morphometrics (Width × Length or Diameter) (µm)**	Ø 1,0±0,2
**Other Code(s)**	LEAN 047	**Taxonomic notes/diacritical features**	
**Parent Sample**	V.06 LAV 2 - CA	**Macroscopic growth features**	color: cyano; forming aggregates
**Isolation Date**	2007/06/18	**Environment**	aquatic, marine
**Order (Older Classification Scheme)**	Chroococcales	**Habitat Sample Description**	intertidal zone, wave-exposed rock, surface scraping
**Order 2014 (Modern Taxonomy)**	Synechococcales	**Ecophysiological traits**	
**Taxonomy Notes**		**Notes on ecophysiological traits**	
**Accession Number(s)_16S**	HQ832914	**Isolator**	Vitor Ramos
**Accession Number(s)_others (product/gene /region)**		**Medium**	Z8 25‰ TM sea salt w/ vit. B12
**Collector**	Rui Seabra	**Strain Status**	non-axenic, unicyanobacterial
**Collection Date**	2006/06/27	**Preservation type**	subculturing
**Location**	Portugal: Praia de Lavadores, Canidelo	**Light:dark cycle**	12:12-h
**Latitude & Longitude**	41.12919 N 8.668578 W	**Temperature (ºC)**	19
		**Light intensity**	10–30 µmol photons m−2 s−1
**References Publicly Available Articles**	Brito et al. (2012) Syst. Appl. Microbiol. 35, 110–119; Costa et al. (2014) Mar. Drugs 12, 98-114		
**References Publicly Available Theses**			

Strain ID	**Cyanobium sp. LEGE 06138**		
**Strain Taxonomy**	Cyanobium Rippka, R & Cohen-Bazire, G (1983)		
**Microphotograph 400×**	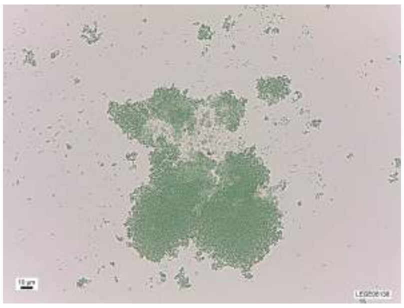	**Microphotograph 1000×**	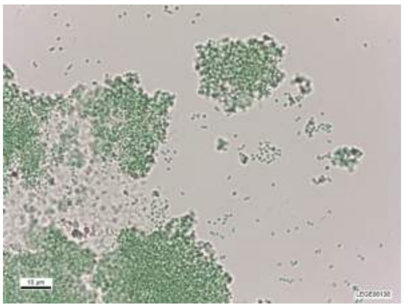
**Coidentity**	n.a.	**Morphometrics (Width × Length or Diameter) (µm)**	Ø 1,1±0,1
**Other Code(s)**	LEAN 048	**Taxonomic notes/diacritical features**	
**Parent Sample**	V.06 AGU 1 - ABA	**Macroscopic growth features**	color: cyano; forming aggregates
**Isolation Date**		**Environment**	aquatic, marine
**Order (Older Classification Scheme)**	Chroococcales	**Habitat Sample Description**	intertidal zone, Sabellaria alveolata reef, epipsamic
**Order 2014 (Modern Taxonomy)**	Synechococcales	**Ecophysiological traits**	
**Taxonomy Notes**		**Notes on ecophysiological traits**	
**Accession Number(s)_16S**	KU951696	**Isolator**	Vitor Ramos
**Accession Number(s)_others (product/gene /region)**		**Medium**	Z8 25‰ TM sea salt w/ vit. B12
**Collector**	Rui Seabra	**Strain Status**	non-axenic, unicyanobacterial
**Collection Date**	2006/06/26	**Preservation type**	subculturing
**Location**	Portugal: Praia da Aguda, Arcozelo	**Light:dark cycle**	12:12-h
**Latitude & Longitude**	41.04954 N 8.655339 W	**Temperature (ºC)**	19
		**Light intensity**	10–30 µmol photons m−2 s−1
**References Publicly Available Articles**			
**References Publicly Available Theses**	Costa M.S. (2011) MSc Dissertation, ICBAS and FCUP, University of Porto, Porto, Portugal		

**Strain ID**	**Cyanobium sp. LEGE 06139**		
**Strain Taxonomy**	Cyanobium Rippka, R & Cohen-Bazire, G (1983)		
**Microphotograph 400×**	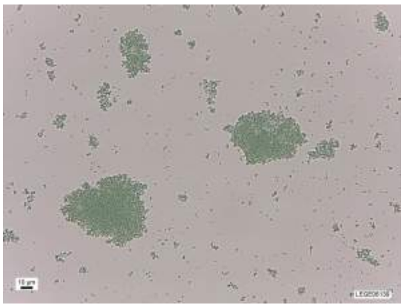	**Microphotograph 1000×**	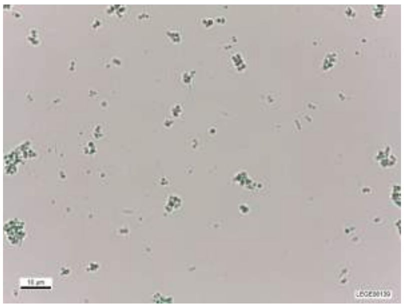
**Coidentity**	n.a.	**Morphometrics (Width × Length or Diameter) (µm)**	Ø 1,1±0,1
**Other Code(s)**	LEAN 049	**Taxonomic notes/diacritical features**	
**Parent Sample**	V.06 AGU 5 - A	**Macroscopic growth features**	color: cyano; growing homogeneously, but also forming aggregates
**Isolation Date**	2007/06/18	**Environment**	aquatic, marine
**Order (Older Classification Scheme)**	Chroococcales	**Habitat Sample Description**	intertidal zone, on a Mytilus sp. shell
**Order 2014 (Modern Taxonomy)**	Synechococcales	**Ecophysiological traits**	
**Taxonomy Notes**		**Notes on ecophysiological traits**	
**Accession Number(s)_16S**	KC469574	**Isolator**	Vitor Ramos
**Accession Number(s)_others (product/gene /region)**		**Medium**	Z8 25‰ TM sea salt w/ vit. B12
**Collector**	Rui Seabra	**Strain Status**	non-axenic, unicyanobacterial
**Collection Date**	2006/06/26	**Preservation type**	subculturing
**Location**	Portugal: Praia da Aguda, Arcozelo	**Light:dark cycle**	12:12-h
**Latitude & Longitude**	41.04954 N 8.655339 W	**Temperature (ºC)**	19
		**Light intensity**	10–30 µmol photons m−2 s−1
**References Publicly Available Articles**	Costa et al. (2014) Mar. Drugs 12, 98-114; Costa et al. (2015) J. Toxicol. Env. Heal. A 78, 432-442		
**References Publicly Available Theses**	Costa M.S. (2011) MSc Dissertation, ICBAS and FCUP, University of Porto, Porto, Portugal		

**Strain ID**	**Cyanobium sp. LEGE 06140**		
**Strain Taxonomy**	Cyanobium Rippka, R & Cohen-Bazire, G (1983)		
**Microphotograph 400×**	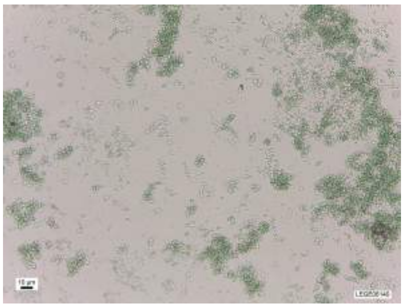	**Microphotograph 1000×**	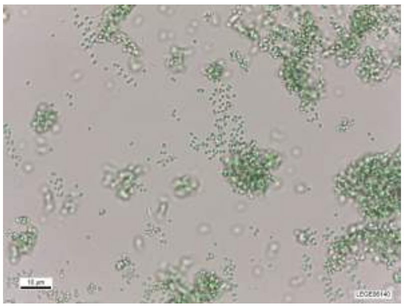
**Coidentity**	n.a.	**Morphometrics (Width × Length or Diameter) (µm)**	Ø 1,1±0,1
**Other Code(s)**	LEAN 050	**Taxonomic notes/diacritical features**	
**Parent Sample**	V.06 AGU 8 - BA	**Macroscopic growth features**	color: cyano; growing homogeneously; mucilaginous
**Isolation Date**	2007	**Environment**	aquatic, marine
**Order (Older Classification Scheme)**	Chroococcales	**Habitat Sample Description**	intertidal zone, wave-exposed rock
**Order 2014 (Modern Taxonomy)**	Synechococcales	**Ecophysiological traits**	EPS production
**Taxonomy Notes**		**Notes on ecophysiological traits**	
**Accession Number(s)_16S**	KU951697	**Isolator**	Vitor Ramos
**Accession Number(s)_others (product/gene /region)**		**Medium**	Z8 25‰ TM sea salt w/ vit. B12
**Collector**	Rui Seabra	**Strain Status**	non-axenic, unicyanobacterial
**Collection Date**	2006/06/26	**Preservation type**	subculturing
**Location**	Portugal: Praia da Aguda, Arcozelo	**Light:dark cycle**	12:12-h
**Latitude & Longitude**	41.04954 N 8.655339 W	**Temperature (ºC)**	19
		**Light intensity**	10–30 µmol photons m−2 s−1
**References Publicly Available Articles**	Brito et al. (2012) Syst. Appl. Microbiol. 35, 110–119		
**References Publicly Available Theses**			

Strain ID	**Cyanobium sp. LEGE 06142**		
**Strain Taxonomy**	Cyanobium Rippka, R & Cohen-Bazire, G (1983)		
**Microphotograph 400×**	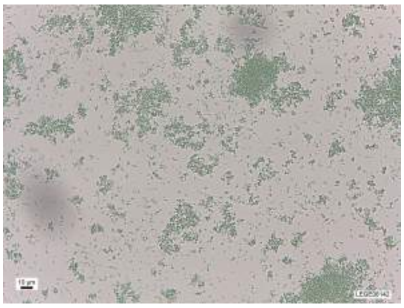	**Microphotograph 1000×**	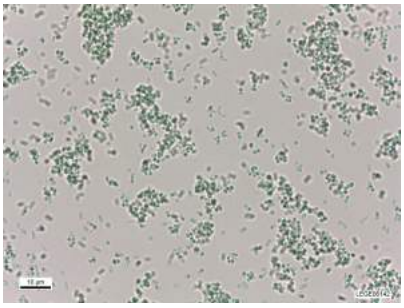
**Coidentity**	n.a.	**Morphometrics (Width × Length or Diameter) (µm)**	Ø 1,1±0,1
**Other Code(s)**	LEAN 053	**Taxonomic notes/diacritical features**	
**Parent Sample**	V.06 MAR 6 - AAA	**Macroscopic growth features**	color: cyano; growing homogeneously
**Isolation Date**		**Environment**	aquatic, marine
**Order (Older Classification Scheme)**	Chroococcales	**Habitat Sample Description**	intertidal zone, wave-exposed rock surface scraping
**Order 2014 (Modern Taxonomy)**	Synechococcales	**Ecophysiological traits**	
**Taxonomy Notes**		**Notes on ecophysiological traits**	
**Accession Number(s)_16S**		**Isolator**	Vitor Ramos
**Accession Number(s)_others (product/gene /region)**		**Medium**	Z8 25‰ TM sea salt w/ vit. B12
**Collector**	Rui Seabra	**Strain Status**	non-axenic, unicyanobacterial
**Collection Date**	2006/07/16	**Preservation type**	subculturing
**Location**	Portugal: Praia do Martinhal, Vila do Bispo	**Light:dark cycle**	12:12-h
**Latitude & Longitude**	37.01869 N 8.926714 W	**Temperature (ºC)**	19
		**Light intensity**	10–30 µmol photons m−2 s−1
**References Publicly Available Articles**			
**References Publicly Available Theses**			

Strain ID	**Cyanobium sp. LEGE 06143**		
**Strain Taxonomy**	Cyanobium Rippka, R & Cohen-Bazire, G (1983)		
**Microphotograph 400×**	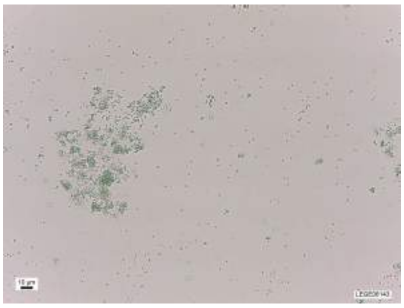	**Microphotograph 1000×**	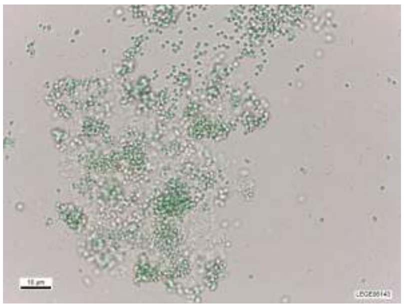
**Coidentity**	n.a.	**Morphometrics (Width × Length or Diameter) (µm)**	Ø 1,2±0,1
**Other Code(s)**	LEAN 055	**Taxonomic notes/diacritical features**	
**Parent Sample**	V.06 BUR 3 - AA	**Macroscopic growth features**	color: cyano; forming aggregates
**Isolation Date**	2007/06/18	**Environment**	aquatic, marine
**Order (Older Classification Scheme)**	Chroococcales	**Habitat Sample Description**	intertidal zone, wave-sheltered zone, sand
**Order 2014 (Modern Taxonomy)**	Synechococcales	**Ecophysiological traits**	
**Taxonomy Notes**		**Notes on ecophysiological traits**	
**Accession Number(s)_16S**	KU951698	**Isolator**	Vitor Ramos
**Accession Number(s)_others (product/gene /region)**		**Medium**	Z8 25‰ TM sea salt w/ vit. B12
**Collector**	Rui Seabra	**Strain Status**	non-axenic, unicyanobacterial
**Collection Date**	2006/07/17	**Preservation type**	subculturing
**Location**	Portugal: Praia de Burgau, Budens	**Light:dark cycle**	12:12-h
**Latitude & Longitude**	37.07156 N 8.774722 W	**Temperature (ºC)**	19
		**Light intensity**	10–30 µmol photons m−2 s−1
**References Publicly Available Articles**	Brito et al. (2012) Syst. Appl. Microbiol. 35, 110–119		
**References Publicly Available Theses**			

Strain ID	**Cyanobium sp. LEGE 06184**		
**Strain Taxonomy**	Cyanobium Rippka, R & Cohen-Bazire, G (1983)		
**Microphotograph 400×**	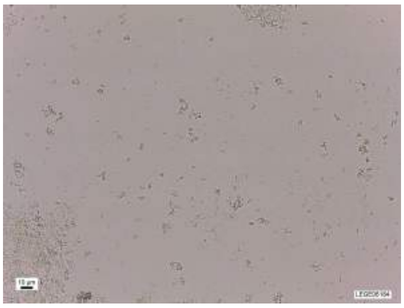	**Microphotograph 1000×**	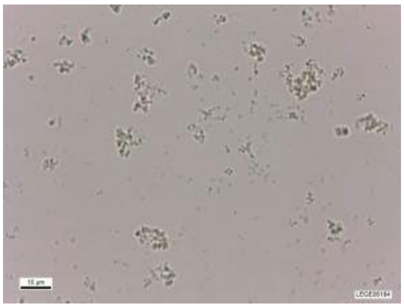
**Coidentity**	n.a.	**Morphometrics (Width × Length or Diameter) (µm)**	Ø 1,0±0,2
**Other Code(s)**	LEAN 098	**Taxonomic notes/diacritical features**	
**Parent Sample**	V.06 BUR p4 - BA	**Macroscopic growth features**	color: cyano; forming aggregates
**Isolation Date**	2007	**Environment**	aquatic, marine
**Order (Older Classification Scheme)**	Chroococcales	**Habitat Sample Description**	sea water sample, coastal
**Order 2014 (Modern Taxonomy)**	Synechococcales	**Ecophysiological traits**	
**Taxonomy Notes**		**Notes on ecophysiological traits**	
**Accession Number(s)_16S**	KU951699	**Isolator**	Vitor Ramos
**Accession Number(s)_others (product/gene /region)**	KC842354 (polyketide synthase)	**Medium**	Z8 25‰ TM sea salt w/ vit. B12
**Collector**	Rui Seabra	**Strain Status**	non-axenic, unicyanobacterial
**Collection Date**	2006/07/17	**Preservation type**	subculturing
**Location**	Portugal: Praia de Burgau, Budens	**Light:dark cycle**	12:12-h
**Latitude & Longitude**	37.07156 N 8.774722 W	**Temperature (ºC)**	19
		**Light intensity**	10–30 µmol photons m−2 s−1
**References Publicly Available Articles**	Brito et al. (2012) Syst. Appl. Microbiol. 35, 110–119; Brito et al. (2015) Algal Res. 9, 218-226		
**References Publicly Available Theses**	Macieira R. (2013) MSc Dissertation, FCUP, University of Porto, Porto, Portugal		

**Strain ID**	**Cyanobium sp. LEGE 07153**		
**Strain Taxonomy**	Cyanobium Rippka, R & Cohen-Bazire, G (1983)		
**Microphotograph 400×**	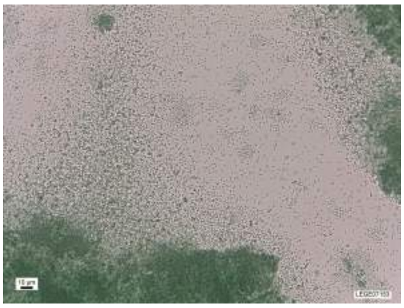	**Microphotograph 1000×**	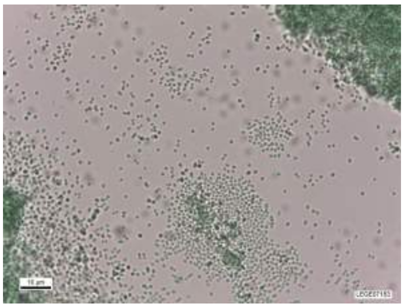
**Coidentity**	n.a.	**Morphometrics (Width × Length or Diameter) (µm)**	Ø 1,2±0,1
**Other Code(s)**	LEAN 067	**Taxonomic notes/diacritical features**	
**Parent Sample**	P.07 MAR 2 - CAA	**Macroscopic growth features**	color: cyano; forming a firm biofilm and also aggregates
**Isolation Date**	2007	**Environment**	aquatic, marine
**Order (Older Classification Scheme)**	Chroococcales	**Habitat Sample Description**	wave-sheltered zone, sand
**Order 2014 (Modern Taxonomy)**	Synechococcales	**Ecophysiological traits**	
**Taxonomy Notes**		**Notes on ecophysiological traits**	
**Accession Number(s)_16S**	KU951700	**Isolator**	Vitor Ramos
**Accession Number(s)_others (product/gene /region)**		**Medium**	Z8 25‰ TM sea salt w/ vit. B12
**Collector**	Rui Seabra/Vitor Ramos	**Strain Status**	non-axenic, unicyanobacterial
**Collection Date**	2007/05/02	**Preservation type**	subculturing
**Location**	Portugal: Praia do Martinhal, Vila do Bispo	**Light:dark cycle**	12:12-h
**Latitude & Longitude**	37.01869 N 8.926714 W	**Temperature (ºC)**	19
		**Light intensity**	10–30 µmol photons m−2 s−1
**References Publicly Available Articles**	Brito et al. (2012) Syst. Appl. Microbiol. 35, 110–119		
**References Publicly Available Theses**			

Strain ID	**Cyanobium sp. LEGE 07173**		
**Strain Taxonomy**	Cyanobium Rippka, R & Cohen-Bazire, G (1983)		
**Microphotograph 400×**	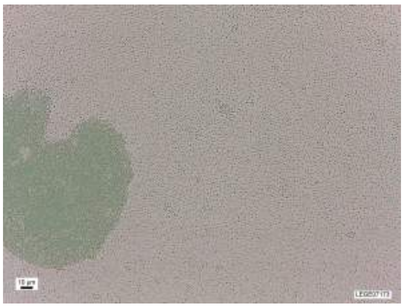	**Microphotograph 1000×**	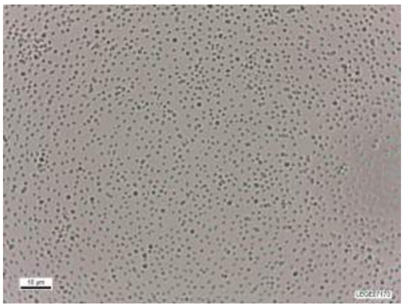
**Coidentity**	n.a.	**Morphometrics (Width × Length or Diameter) (µm)**	Ø 1,2±0,1
**Other Code(s)**	LEAN 087	**Taxonomic notes/diacritical features**	
**Parent Sample**	P.07 ODA 3 - AAA	**Macroscopic growth features**	color: cyano; growing homogeneously
**Isolation Date**		**Environment**	aquatic, marine
**Order (Older Classification Scheme)**	Chroococcales	**Habitat Sample Description**	tide puddle, on a submerged stone
**Order 2014 (Modern Taxonomy)**	Synechococcales	**Ecophysiological traits**	
**Taxonomy Notes**		**Notes on ecophysiological traits**	
**Accession Number(s)_16S**	KU951701	**Isolator**	Vitor Ramos
**Accession Number(s)_others (product/gene /region)**	KC813193, KC813194 (non-ribosomal peptide synthetase), KC842372 (polyketide synthase)	**Medium**	Z8 25‰ TM sea salt w/ vit. B12
**Collector**	Rui Seabra/Vitor Ramos	**Strain Status**	non-axenic, unicyanobacterial
**Collection Date**	2007/05/02	**Preservation type**	subculturing
**Location**	Portugal: Praia de Olhos d'Água, Albufeira	**Light:dark cycle**	12:12-h
**Latitude & Longitude**	37.08973 N 8.191017 W	**Temperature (ºC)**	19
		**Light intensity**	10–30 µmol photons m−2 s−1
**References Publicly Available Articles**	Brito et al. (2012) Syst. Appl. Microbiol. 35, 110–119; Costa et al. (2014) Mar. Drugs 12, 98-114; Brito et al. (2015) Algal Res. 9, 218-226		
**References Publicly Available Theses**			

**Strain ID**	**Cyanobium sp. LEGE 07175**		
**Strain Taxonomy**	Cyanobium Rippka, R & Cohen-Bazire, G (1983)		
**Microphotograph 400×**	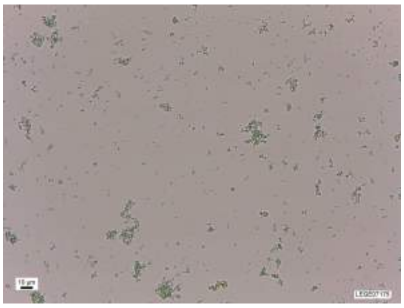	**Microphotograph 1000×**	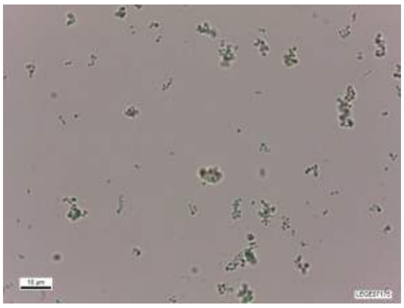
**Coidentity**	n.a.	**Morphometrics (Width × Length or Diameter) (µm)**	Ø 1,0±0,1
**Other Code(s)**	LEAN 089	**Taxonomic notes/diacritical features**	several cell clusters
**Parent Sample**	P.07 MAR p1 - B	**Macroscopic growth features**	color: cyano; growing homogeneously, but also forming aggregates
**Isolation Date**	2007	**Environment**	aquatic, marine
**Order (Older Classification Scheme)**	Chroococcales	**Habitat Sample Description**	sea water sample, coastal, surf zone
**Order 2014 (Modern Taxonomy)**	Synechococcales	**Ecophysiological traits**	
**Taxonomy Notes**		**Notes on ecophysiological traits**	
**Accession Number(s)_16S**	KC469575	**Isolator**	Vitor Ramos
**Accession Number(s)_others (product/gene /region)**		**Medium**	Z8 25‰ TM sea salt w/ vit. B12
**Collector**	Rui Seabra/Vitor Ramos	**Strain Status**	non-axenic, unicyanobacterial
**Collection Date**	2007/05/02	**Preservation type**	subculturing
**Location**	Portugal: Praia do Martinhal, Vila do Bispo	**Light:dark cycle**	12:12-h
**Latitude & Longitude**	37.01869 N 8.926714 W	**Temperature (ºC)**	19
		**Light intensity**	10–30 µmol photons m−2 s−1
**References Publicly Available Articles**	Costa et al. (2014) Mar. Drugs 12, 98-114; Costa et al. (2015) J. Toxicol. Env. Heal. A 78, 432-442		
**References Publicly Available Theses**	Costa M.S. (2011) MSc Dissertation, ICBAS and FCUP, University of Porto, Porto, Portugal		

**Strain ID**	**Cyanobium sp. LEGE 07183**		
**Strain Taxonomy**	Cyanobium Rippka, R & Cohen-Bazire, G (1983)		
**Microphotograph 400×**	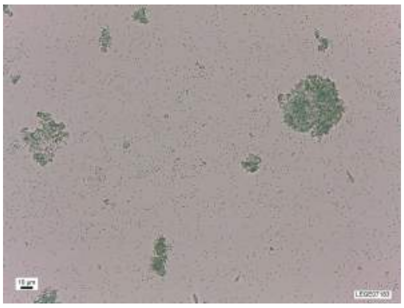	**Microphotograph 1000×**	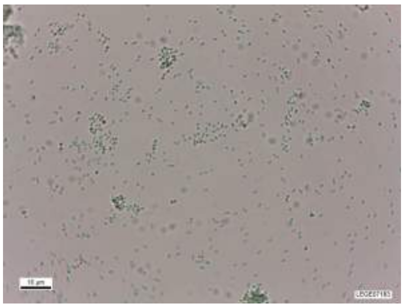
**Coidentity**	n.a.	**Morphometrics (Width × Length or Diameter) (µm)**	Ø 0,7±0,1
**Other Code(s)**	LEAN 097	**Taxonomic notes/diacritical features**	short rod-shaped cells
**Parent Sample**	P.07 ODA 1 - AAA	**Macroscopic growth features**	color: cyano; forming a firm biofilm
**Isolation Date**		**Environment**	aquatic, marine
**Order (Older Classification Scheme)**	Chroococcales	**Habitat Sample Description**	tide puddle, rock surface scraping
**Order 2014 (Modern Taxonomy)**	Synechococcales	**Ecophysiological traits**	
**Taxonomy Notes**		**Notes on ecophysiological traits**	
**Accession Number(s)_16S**	KU951702	**Isolator**	Vitor Ramos
**Accession Number(s)_others (product/gene /region)**		**Medium**	Z8 25‰ TM sea salt w/ vit. B12
**Collector**	Rui Seabra/Vitor Ramos	**Strain Status**	non-axenic, unicyanobacterial
**Collection Date**	2007/05/02	**Preservation type**	subculturing
**Location**	Portugal: Praia de Olhos d'Água, Albufeira	**Light:dark cycle**	12:12-h
**Latitude & Longitude**	37.08973 N 8.191017 W	**Temperature (ºC)**	19
		**Light intensity**	10–30 µmol photons m−2 s−1
**References Publicly Available Articles**			
**References Publicly Available Theses**	Costa M.S. (2011) MSc Dissertation, ICBAS and FCUP, University of Porto, Porto, Portugal		

**Strain ID**	**Cyanobium sp. LEGE 07186**		
**Strain Taxonomy**	Cyanobium Rippka, R & Cohen-Bazire, G (1983)		
**Microphotograph 400×**	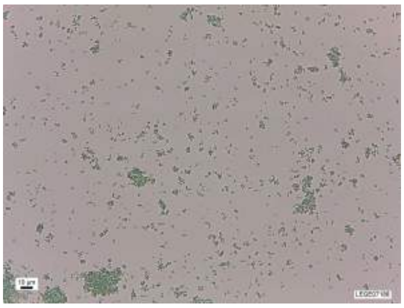	**Microphotograph 1000×**	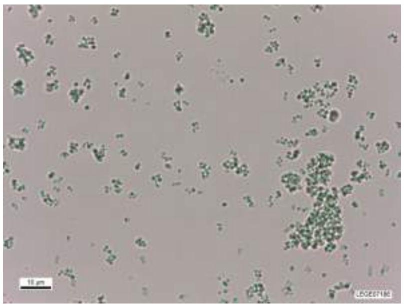
**Coidentity**	n.a.	**Morphometrics (Width × Length or Diameter) (µm)**	Ø 1,2±0,2
**Other Code(s)**	LEAN 150	**Taxonomic notes/diacritical features**	
**Parent Sample**	P.07 MAR 6 - p96, dil. 1/4 - AB	**Macroscopic growth features**	color: cyano; growing homogeneously, but also forming aggregates
**Isolation Date**	2007	**Environment**	aquatic, marine
**Order (Older Classification Scheme)**	Chroococcales	**Habitat Sample Description**	tide pool, on a submerged stone
**Order 2014 (Modern Taxonomy)**	Synechococcales	**Ecophysiological traits**	
**Taxonomy Notes**		**Notes on ecophysiological traits**	
**Accession Number(s)_16S**	KC469576	**Isolator**	Vitor Ramos
**Accession Number(s)_others (product/gene /region)**	KC842302, KC842303 (polyketide synthase)	**Medium**	Z8 25‰ TM sea salt w/ vit. B12
**Collector**	Rui Seabra/Vitor Ramos	**Strain Status**	non-axenic, unicyanobacterial
**Collection Date**	2007/05/02	**Preservation type**	subculturing
**Location**	Portugal: Praia do Martinhal, Vila do Bispo	**Light:dark cycle**	12:12-h
**Latitude & Longitude**	37.01869 N 8.926714 W	**Temperature (ºC)**	19
		**Light intensity**	10–30 µmol photons m−2 s−1
**References Publicly Available Articles**	Costa et al. (2014) Mar. Drugs 12, 98-114; Costa et al. (2015) J. Toxicol. Env. Heal. A 78, 432-442; Brito et al. (2015) Algal Res. 9, 218-226		
**References Publicly Available Theses**	Costa M.S. (2011) MSc Dissertation, ICBAS and FCUP, University of Porto, Porto, Portugal		

Strain ID	**Cyanobium sp. LEGE 07293**		
**Strain Taxonomy**	Cyanobium Rippka, R & Cohen-Bazire, G (1983)		
**Microphotograph 400×**		**Microphotograph 1000×**	
**Coidentity**	n.a.	**Morphometrics (Width × Length or Diameter) (µm)**	
**Other Code(s)**	SE 199#2	**Taxonomic notes/diacritical features**	
**Parent Sample**	n.d.	**Macroscopic growth features**	color: brownish; forming a firm biofilm and aggregates
**Isolation Date**		**Environment**	aquatic, brackish
**Order (Older Classification Scheme)**	Chroococcales	**Habitat Sample Description**	mesotidal zone, benthic
**Order 2014 (Modern Taxonomy)**	Synechococcales	**Ecophysiological traits**	
**Taxonomy Notes**		**Notes on ecophysiological traits**	
**Accession Number(s)_16S**	HM217048	**Isolator**	Viviana Lopes
**Accession Number(s)_others (product/gene/region)**		**Medium**	Z8
**Collector**	Viviana Lopes	**Strain Status**	non-axenic, unicyanobacterial, delicate
**Collection Date**	2007/04/03	**Preservation type**	subculturing
**Location**	Portugal: Minho estuary, Seixas	**Light:dark cycle**	12:12-h
**Latitude & Longitude**	41.90181 N 8.813139 W	**Temperature (ºC)**	19
		**Light intensity**	10–30 µmol photons m−2 s−1
**References Publicly Available Articles**	Lopes et al. (2012) Mar. Environ. Res. 73, 7–16		
**References Publicly Available Theses**	Lopes V.R. (2011) PhD Thesis, FCUP, University of Porto, Porto, Portugal		

**Strain ID**	**Cyanobium sp. LEGE 07313**		
**Strain Taxonomy**	Cyanobium Rippka, R & Cohen-Bazire, G (1983)		
**Microphotograph 400×**	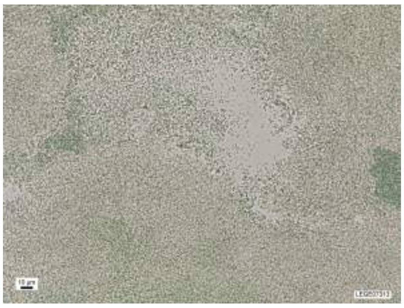	**Microphotograph 1000×**	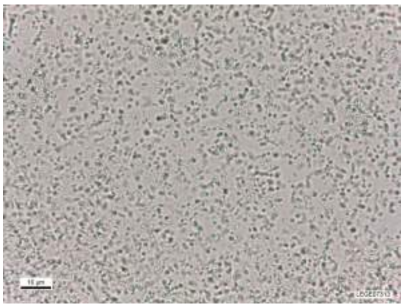
**Coidentity**	n.a.	**Morphometrics (Width × Length or Diameter) (µm)**	Ø 0,8±0,2 (spherical cells); 0,8±0,2 × 1,6±0,3 (elongated cells); 0,8±0,2 × 5,0±1,8 (involution cells)
**Other Code(s)**		**Taxonomic notes/diacritical features**	rod-shaped cells; some elongated, involution cells observed
**Parent Sample**	363_4Flu	**Macroscopic growth features**	color: cyano; growing homogeneously, but also forming aggregates
**Isolation Date**		**Environment**	aquatic, brackish
**Order (Older Classification Scheme)**	Chroococcales	**Habitat Sample Description**	mesotidal zone, benthic
**Order 2014 (Modern Taxonomy)**	Synechococcales	**Ecophysiological traits**	
**Taxonomy Notes**		**Notes on ecophysiological traits**	
**Accession Number(s)_16S**	KU951703	**Isolator**	Viviana Lopes
**Accession Number(s)_others (product/gene /region)**		**Medium**	Z8
**Collector**	Viviana Lopes	**Strain Status**	non-axenic, unicyanobacterial
**Collection Date**	2007	**Preservation type**	subculturing
**Location**	Portugal	**Light:dark cycle**	12:12-h
**Latitude & Longitude**	unknown	**Temperature (ºC)**	19
		**Light intensity**	10–30 µmol photons m−2 s−1
**References Publicly Available Articles**			
**References Publicly Available Theses**			

Strain ID	**Cyanobium sp. LEGE 07318**		
**Strain Taxonomy**	Cyanobium Rippka, R & Cohen-Bazire, G (1983)		
**Microphotograph 400×**	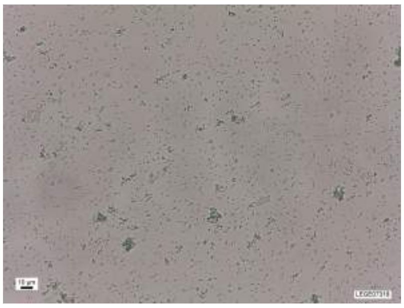	**Microphotograph 1000×**	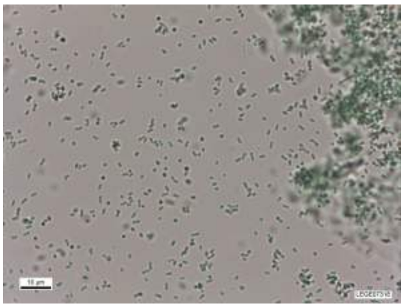
**Coidentity**	n.a.	**Morphometrics (Width × Length or Diameter) (µm)**	Ø 0,8±0,2
**Other Code(s)**	Por357	**Taxonomic notes/diacritical features**	shortly rod-shaped cells; several cell clusters
**Parent Sample**	n.d.	**Macroscopic growth features**	color: cyano; forming a firm biofilm and aggregates
**Isolation Date**		**Environment**	aquatic, brackish
**Order (Older Classification Scheme)**	Chroococcales	**Habitat Sample Description**	mesotidal zone, benthic
**Order 2014 (Modern Taxonomy)**	Synechococcales	**Ecophysiological traits**	
**Taxonomy Notes**		**Notes on ecophysiological traits**	
**Accession Number(s)_16S**	HM217050	**Isolator**	Viviana Lopes
**Accession Number(s)_others (product/gene /region)**		**Medium**	Z8 25‰ TM sea salt w/ vit. B12
**Collector**	Viviana Lopes	**Strain Status**	non-axenic, unicyanobacterial
**Collection Date**	2007/10/10	**Preservation type**	subculturing
**Location**	Portugal: Douro estuary, Porto	**Light:dark cycle**	12:12-h
**Latitude & Longitude**	41.14658 N 8.657972 W	**Temperature (ºC)**	19
		**Light intensity**	10–30 µmol photons m−2 s−1
**References Publicly Available Articles**	Lopes et al. (2012) Mar. Environ. Res. 73, 7–16		
**References Publicly Available Theses**	Lopes V.R. (2011) PhD Thesis, FCUP, University of Porto, Porto, Portugal		

**Strain ID**	**Cyanobium sp. LEGE 10374**		
**Strain Taxonomy**	Cyanobium Rippka, R & Cohen-Bazire, G (1983)		
**Microphotograph 400×**	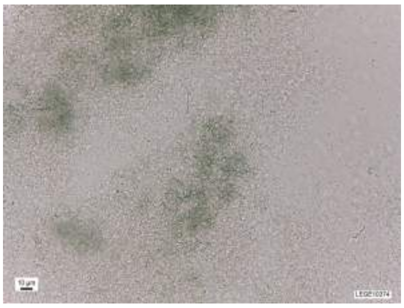	**Microphotograph 1000×**	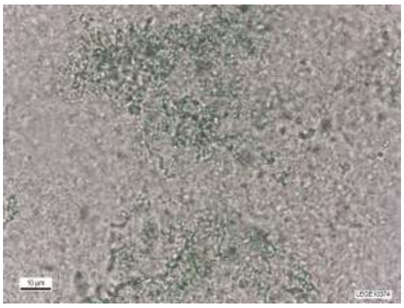
**Coidentity**	n.a.	**Morphometrics (Width × Length or Diameter) (µm)**	Ø 0,7±0,1 (Spherical Cells); 1,3±0,1 × 13,2±3,5 (Involution cells); 1,1±0,2 × 1,3±0,3 (Elongated cells)
**Other Code(s)**	16Hp1	**Taxonomic notes/diacritical features**	some elongated, involution cells observed
**Parent Sample**		**Macroscopic growth features**	
**Isolation Date**		**Environment**	aquatic, marine
**Order (Older Classification Scheme)**	Chroococcales	**Habitat Sample Description**	intertidal zone, on a marine sponge
**Order 2014 (Modern Taxonomy)**	Synechococcales	**Ecophysiological traits**	
**Taxonomy Notes**		**Notes on ecophysiological traits**	
**Accession Number(s)_16S**	KU951704	**Isolator**	Ana Regueiras
**Accession Number(s)_others (product/gene /region)**		**Medium**	Z8 25‰ TM sea salt w/ vit. B12
**Collector**	Ana Regueiras	**Strain Status**	non-axenic, unicyanobacterial
**Collection Date**	2010	**Preservation type**	subculturing
**Location**	Portugal	**Light:dark cycle**	12:12-h
**Latitude & Longitude**	unknown	**Temperature (ºC)**	19
		**Light intensity**	10–30 µmol photons m−2 s−1
**References Publicly Available Articles**			
**References Publicly Available Theses**	Vivas J. (2013) MSc Dissertation, FCUP, University of Porto, Porto, Portugal		

**Strain ID**	**Cyanobium sp. LEGE 10375**		
**Strain Taxonomy**	Cyanobium Rippka, R & Cohen-Bazire, G (1983)		
**Microphotograph 400×**	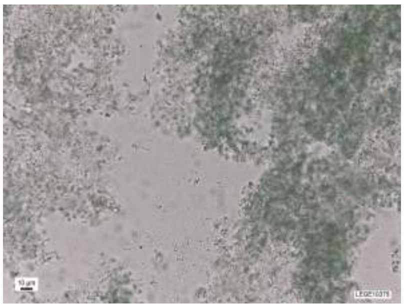	**Microphotograph 1000×**	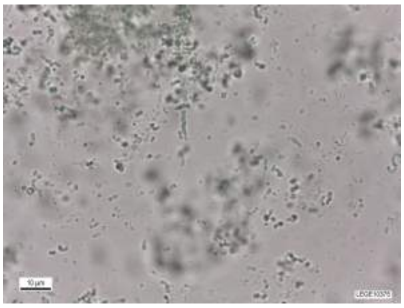
**Coidentity**	n.a.	**Morphometrics (Width × Length or Diameter) (µm)**	Ø 1,2±0,3 (Cells); 1,2±0,2 × 6,4±3,2 (Involution Cells); 1,2±0,2 × 1,8±0,4 (Elongated Cells)
**Other Code(s)**	16Hp2	**Taxonomic notes/diacritical features**	some sigmoid, elongated, involution cells; distinguishable centro- and chromatoplasm
**Parent Sample**		**Macroscopic growth features**	color: cyano; forming a smooth biofilm
**Isolation Date**		**Environment**	aquatic, marine
**Order (Older Classification Scheme)**	Chroococcales	**Habitat Sample Description**	intertidal zone, on a marine sponge
**Order 2014 (Modern Taxonomy)**	Synechococcales	**Ecophysiological traits**	
**Taxonomy Notes**		**Notes on ecophysiological traits**	
**Accession Number(s)_16S**	KU951705	**Isolator**	Ana Regueiras
**Accession Number(s)_others (product/gene /region)**		**Medium**	Z8 25‰ TM sea salt w/ vit. B12
**Collector**	Ana Regueiras	**Strain Status**	non-axenic, unicyanobacterial
**Collection Date**	2010/10/24	**Preservation type**	subculturing
**Location**	Portugal: Praia de São Bartolomeu do Mar, Esposende	**Light:dark cycle**	12:12-h
**Latitude & Longitude**	41.57378 N 8.798556 W	**Temperature (ºC)**	19
		**Light intensity**	10–30 µmol photons m−2 s−1
**References Publicly Available Articles**			
**References Publicly Available Theses**	Vivas J. (2013) MSc Dissertation, FCUP, University of Porto, Porto, Portugal		

**Strain ID**	**Cyanobium sp. LEGE 11435**		
**Strain Taxonomy**	Cyanobium Rippka, R & Cohen-Bazire, G (1983)		
**Microphotograph 400×**	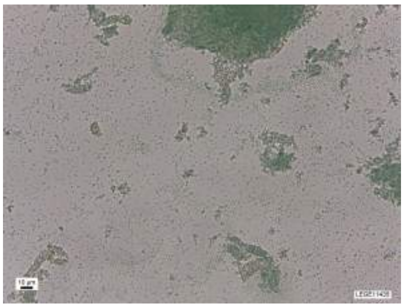	**Microphotograph 1000×**	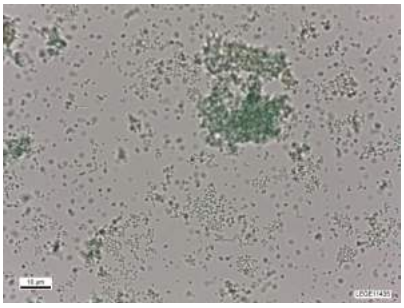
**Coidentity**	n.a.	**Morphometrics (Width × Length or Diameter) (µm)**	Ø 0,8±0,2
**Other Code(s)**		**Taxonomic notes/diacritical features**	several cell clusters
**Parent Sample**	3.003.10.11 2ª MN_AP.E-A/b	**Macroscopic growth features**	color: cyano; forming aggregates
**Isolation Date**		**Environment**	aquatic, marine
**Order (Older Classification Scheme)**	Chroococcales	**Habitat Sample Description**	subtidal sample, epilithic (13m depth), about 200 m off the shore
**Order 2014 (Modern Taxonomy)**	Synechococcales	**Ecophysiological traits**	
**Taxonomy Notes**		**Notes on ecophysiological traits**	
**Accession Number(s)_16S**	KU951706	**Isolator**	Ângela Pinheiro
**Accession Number(s)_others (product/gene /region)**		**Medium**	Z8 25‰ TM sea salt w/ vit. B12
**Collector**	Pedro Leão/Aldo Barreiro	**Strain Status**	non-axenic, unicyanobacterial
**Collection Date**	2011/10/03	**Preservation type**	subculturing
**Location**	Portugal: 'A Pedra', diving spot in front of the fort 'Castelo do Queijo'	**Light:dark cycle**	12:12-h
**Latitude & Longitude**	41.185809 N 8.719079 W	**Temperature (ºC)**	19
		**Light intensity**	10–30 µmol photons m−2 s−1
**References Publicly Available Articles**			
**References Publicly Available Theses**			

**Strain ID**	**Cyanobium sp. LEGE 11436**		
**Strain Taxonomy**	Cyanobium Rippka, R & Cohen-Bazire, G (1983)		
**Microphotograph 400×**	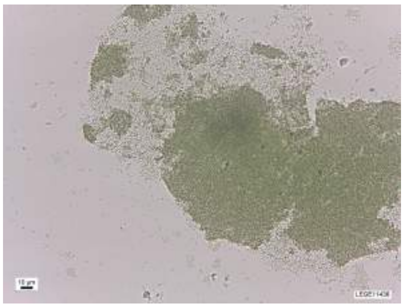	**Microphotograph 1000×**	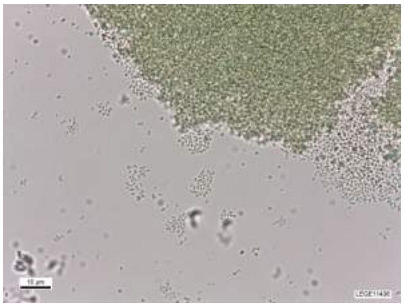
**Coidentity**	n.a.	**Morphometrics (Width × Length or Diameter) (µm)**	Ø 0,8±0,2
**Other Code(s)**		**Taxonomic notes/diacritical features**	several cell clusters
**Parent Sample**	3.003.10.11 2ª MN_AP.N(A)/a	**Macroscopic growth features**	color: cyano; forming aggregates
**Isolation Date**		**Environment**	aquatic, marine
**Order (Older Classification Scheme)**	Chroococcales	**Habitat Sample Description**	subtidal sample, epilithic (13m depth), about 200 m off the shore
**Order 2014 (Modern Taxonomy)**	Synechococcales	**Ecophysiological traits**	
**Taxonomy Notes**		**Notes on ecophysiological traits**	
**Accession Number(s)_16S**	KU951707	**Isolator**	Ângela Pinheiro
**Accession Number(s)_others (product/gene /region)**		**Medium**	Z8 25‰ TM sea salt w/ vit. B12
**Collector**	Pedro Leão/Aldo Barreiro	**Strain Status**	non-axenic, unicyanobacterial
**Collection Date**	2011/10/03	**Preservation type**	subculturing
**Location**	Portugal: 'A Pedra', diving spot in front of the fort 'Castelo do Queijo'	**Light:dark cycle**	12:12-h
**Latitude & Longitude**	41.185809 N 8.719079 W	**Temperature (ºC)**	19
		**Light intensity**	10–30 µmol photons m−2 s−1
**References Publicly Available Articles**			
**References Publicly Available Theses**			

**Strain ID**	**Cyanobium sp. LEGE 11437**		
**Strain Taxonomy**	Cyanobium Rippka, R & Cohen-Bazire, G (1983)		
**Microphotograph 400×**	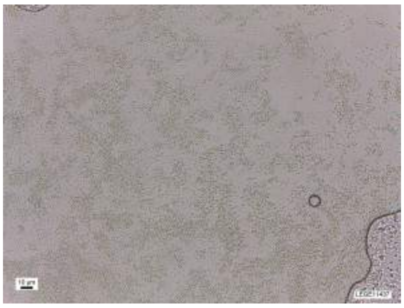	**Microphotograph 1000×**	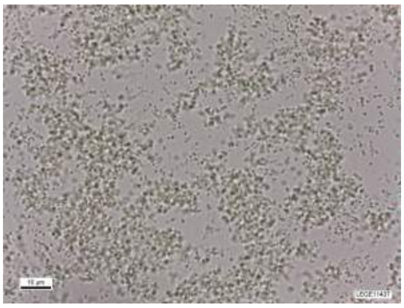
**Coidentity**	n.a.	**Morphometrics (Width × Length or Diameter) (µm)**	Ø 0,9±0,2
**Other Code(s)**		**Taxonomic notes/diacritical features**	pseudofilaments observed
**Parent Sample**	3.002.09.11 MN	**Macroscopic growth features**	color: greenish; forming aggregates
**Isolation Date**		**Environment**	aquatic, marine
**Order (Older Classification Scheme)**	Chroococcales	**Habitat Sample Description**	subtidal sample, epilithic (10-13m depth), less than 1 km off the shore
**Order 2014 (Modern Taxonomy)**	Synechococcales	**Ecophysiological traits**	
**Taxonomy Notes**		**Notes on ecophysiological traits**	
**Accession Number(s)_16S**	KU951708	**Isolator**	Sofia Costa
**Accession Number(s)_others (product/gene /region)**		**Medium**	Z8 25‰ TM sea salt w/ vit. B12
**Collector**	Pedro Leão/Aldo Barreiro	**Strain Status**	non-axenic, unicyanobacterial
**Collection Date**	2011/09/26	**Preservation type**	subculturing
**Location**	Portugal: 'Pêlo Negro', diving spot near Leixões Harbour	**Light:dark cycle**	12:12-h
**Latitude & Longitude**	41.18557 N 8.719100 W	**Temperature (ºC)**	19
		**Light intensity**	10–30 µmol photons m−2 s−1
**References Publicly Available Articles**			
**References Publicly Available Theses**			

**Strain ID**	**Cyanobium sp. LEGE 11470**		
**Strain Taxonomy**	Cyanobium Rippka, R & Cohen-Bazire, G (1983)		
**Microphotograph 400×**	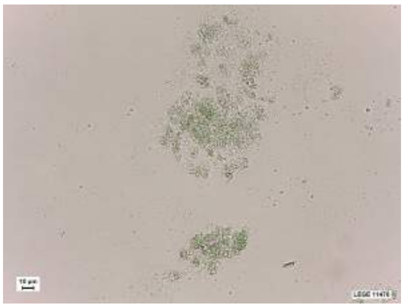	**Microphotograph 1000×**	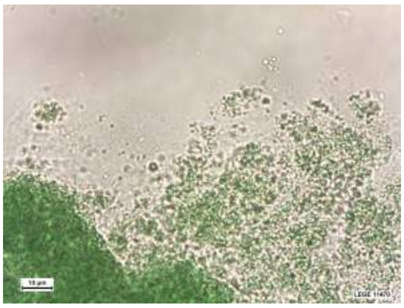
**Coidentity**	n.a.	**Morphometrics (Width × Length or Diameter) (µm)**	Ø 0,9±0,1
**Other Code(s)**		**Taxonomic notes/diacritical features**	
**Parent Sample**	15.003.1011	**Macroscopic growth features**	color: cyano; growing homogeneously and forming a firm biofilm
**Isolation Date**		**Environment**	aquatic, marine
**Order (Older Classification Scheme)**	Chroococcales	**Habitat Sample Description**	subtidal sample, epilithic (13m depth), about 200 m off the shore
**Order 2014 (Modern Taxonomy)**	Synechococcales	**Ecophysiological traits**	
**Taxonomy Notes**		**Notes on ecophysiological traits**	
**Accession Number(s)_16S**	KU951709	**Isolator**	Ângela Pinheiro
**Accession Number(s)_others (product/gene /region)**		**Medium**	Z8 25‰ TM sea salt w/ vit. B12
**Collector**	Pedro Leão/Aldo Barreiro	**Strain Status**	non-axenic, unicyanobacterial
**Collection Date**	2011/10/03	**Preservation type**	subculturing
**Location**	Portugal: 'A Pedra', diving spot in front of the fort 'Castelo do Queijo'	**Light:dark cycle**	12:12-h
**Latitude & Longitude**	41.185809 N 8.719079 W	**Temperature (ºC)**	19
		**Light intensity**	10–30 µmol photons m−2 s−1
**References Publicly Available Articles**			
**References Publicly Available Theses**			

Strain ID	**Cyanobium sp. LEGE 11482**		
**Strain Taxonomy**	Cyanobium Rippka, R & Cohen-Bazire, G (1983)		
**Microphotograph 400×**	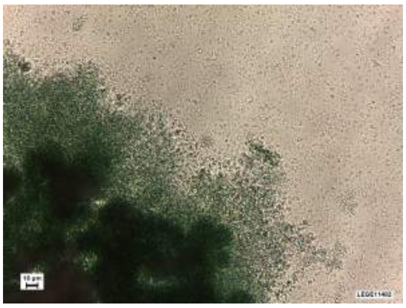	**Microphotograph 1000×**	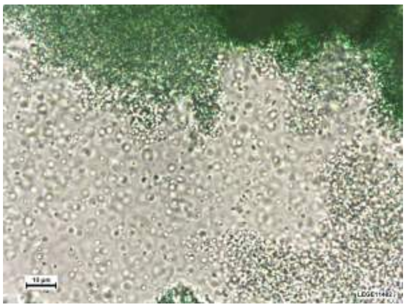
**Coidentity**	n.a.	**Morphometrics (Width × Length or Diameter) (µm)**	Ø 0,8±0,2
**Other Code(s)**		**Taxonomic notes/diacritical features**	
**Parent Sample**	15.003.1011 wall A/B	**Macroscopic growth features**	
**Isolation Date**		**Environment**	aquatic, marine
**Order (Older Classification Scheme)**	Chroococcales	**Habitat Sample Description**	subtidal sample, epilithic (13m depth), about 200 m off the shore
**Order 2014 (Modern Taxonomy)**	Synechococcales	**Ecophysiological traits**	
**Taxonomy Notes**		**Notes on ecophysiological traits**	
**Accession Number(s)_16S**	ready to submit	**Isolator**	Ângela Pinheiro
**Accession Number(s)_others (product/gene /region)**		**Medium**	Z8 25‰ TM sea salt w/ vit. B12
**Collector**	Pedro Leão/Aldo Barreiro	**Strain Status**	non-axenic, unicyanobacterial
**Collection Date**	2011/10/03	**Preservation type**	subculturing
**Location**	Portugal: 'A Pedra', diving spot in front of the fort 'Castelo do Queijo'	**Light:dark cycle**	12:12-h
**Latitude & Longitude**	41.185809 N 8.719079 W	**Temperature (ºC)**	19
		**Light intensity**	10–30 µmol photons m−2 s−1
**References Publicly Available Articles**			
**References Publicly Available Theses**			

Strain ID	**Cyanobium sp. LEGE XX442**		
**Strain Taxonomy**	Cyanobium Rippka, R & Cohen-Bazire, G (1983)		
**Microphotograph 400×**	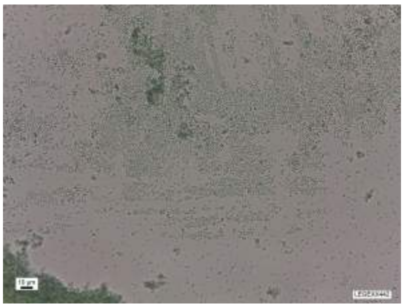	**Microphotograph 1000×**	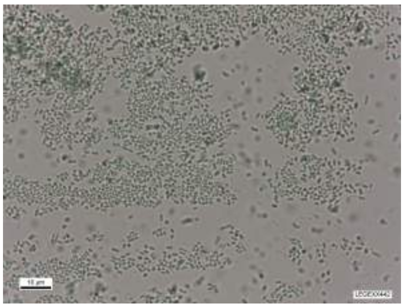
**Coidentity**	n.a.	**Morphometrics (Width × Length or Diameter) (µm)**	Ø 0,8±0,1 (spherical cells); 0,8±0,1× 3,0±0,3 (involution cells); 0,8±0,1× 1,7±0,1 (elongated cells)
**Other Code(s)**		**Taxonomic notes/diacritical features**	shortly rod-shaped cells; some elongated, involution cells observed
**Parent Sample**	VL Por 1	**Macroscopic growth features**	color: cyano; growing homogeneously, but also forming aggregates
**Isolation Date**		**Environment**	aquatic, brackish
**Order (Older Classification Scheme)**	Chroococcales	**Habitat Sample Description**	mesotidal zone
**Order 2014 (Modern Taxonomy)**	Synechococcales	**Ecophysiological traits**	
**Taxonomy Notes**		**Notes on ecophysiological traits**	
**Accession Number(s)_16S**	KU951710	**Isolator**	Viviana Lopes
**Accession Number(s)_others (product/gene /region)**		**Medium**	MN w/ vit. B12
**Collector**	Viviana Lopes	**Strain Status**	non-axenic, unicyanobacterial
**Collection Date**	unknown	**Preservation type**	subculturing
**Location**	Portugal: Douro estuary, Porto	**Light:dark cycle**	12:12-h
**Latitude & Longitude**	41.14739 N 8.667639 W	**Temperature (ºC)**	19
		**Light intensity**	10–30 µmol photons m−2 s−1
**References Publicly Available Articles**			
**References Publicly Available Theses**			

Strain ID	**Cylindrospermopsis raciborskii LEGE 95046**		
**Strain Taxonomy**	Cylindrospermopsis raciborskii (Woloszynska) Seenayya, G & Subba Raju, N (1972)		
**Microphotograph 400×**	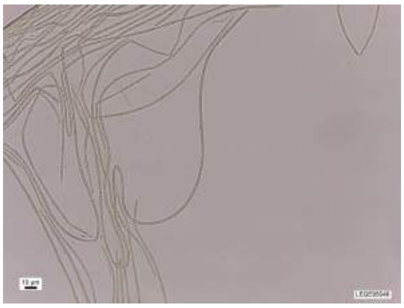	**Microphotograph 1000×**	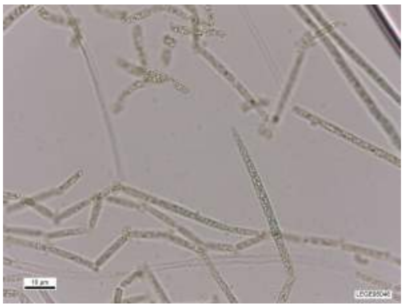
**Coidentity**	n.a.	**Morphometrics (Width × Length or Diameter) (µm)**	2,6±0,8 × 12,4±3,0 (Cells); 3,6±0,4 × 6,0±3,3 (Heterocytes)
**Other Code(s)**	CR7, LJ	**Taxonomic notes/diacritical features**	trichomes with vacuolated cells and narrowed toward ends
**Parent Sample**	n.d.	**Macroscopic growth features**	color: cyano; mucilaginous
**Isolation Date**	1995	**Environment**	aquatic, freshwater
**Order (Older Classification Scheme)**	Nostocales	**Habitat Sample Description**	water sample from a potable supply
**Order 2014 (Modern Taxonomy)**	Nostocales	**Ecophysiological traits**	nitrogen-fixation, buoyancy, EPS production
**Taxonomy Notes**		**Notes on ecophysiological traits**	non-CYN producing strain (by HPLC); nontoxic by mouse bioassay
**Accession Number(s)_16S**	AF516725	**Isolator**	Martin Saker
**Accession Number(s)_others (product/gene /region)**	HQ407336 (rpoC1), HQ407347 (16S-23S rRNA ITS, longer spacer), HQ407360 (16S-23S rRNA ITS, shorter spacer)	**Medium**	Z8
**Collector**	Martin Saker	**Strain Status**	non-axenic, unicyanobacterial
**Collection Date**	1995/06/17	**Preservation type**	subculturing
**Location**	Australia: Lake Julius, Queensland	**Light:dark cycle**	12:12-h
**Latitude & Longitude**	unknown	**Temperature (ºC)**	19
		**Light intensity**	10–30 µmol photons m−2 s−1
**References Publicly Available Articles**	Saker & Griffiths (2000) Phycologia, 39, 349-354; Saker & Neilan (2001) Appl Environ Microbiol 67,1839–45; Saker et al. (2003) Mol. Ecol. 12 (1), 133-140; Saker et al. (2003) Limnetica 22, 129-136; Saker et al. (2003) Ecotox. Environ. Safe. 55, 243–250; Neilan et al. (2003) Mol. Ecol. 12 (1), 133-140; Berry et al. (2009) Toxicon 53, 289–299; Stucken et al. (2009) Syst. Appl. Microbiol. 32, 37–48; Moreira et al. (2011) Curr. Microbiol. 62 (5), 1590-1595; Puerto et al. (2011) Aquat. Toxicol. 101, 109–116		
**References Publicly Available Theses**	Saker M.L. (2000) PhD Thesis, James Cook University, Townsville, Australia		

**Strain ID**	**Cylindrospermopsis raciborskii LEGE 97047**		
**Strain Taxonomy**	Cylindrospermopsis raciborskii (Woloszynska) Seenayya, G & Subba Raju, N (1972)		
**Microphotograph 400×**	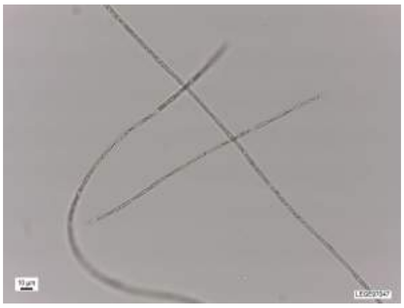	**Microphotograph 1000×**	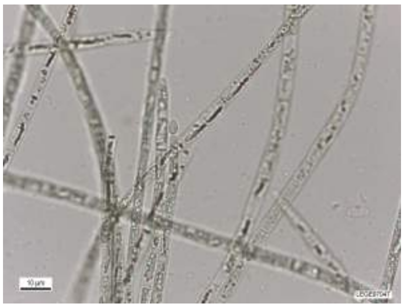
**Coidentity**	n.a.	**Morphometrics (Width × Length or Diameter) (µm)**	3,2±0,8 × 9,4±2,4 (Cells); 2,9±0,5 × 4,7±0,7 (Heterocytes)
**Other Code(s)**	CR3, AQS, Cylin-A	**Taxonomic notes/diacritical features**	trichomes with vacuolated cells and narrowed toward ends
**Parent Sample**	n.d.	**Macroscopic growth features**	color: cyano; forming aggregates
**Isolation Date**	Aug-1997	**Environment**	aquatic, freshwater
**Order (Older Classification Scheme)**	Nostocales	**Habitat Sample Description**	from an aquaculture pond
**Order 2014 (Modern Taxonomy)**	Nostocales	**Ecophysiological traits**	toxin production, nitrogen-fixation, buoyancy
**Taxonomy Notes**		**Notes on ecophysiological traits**	CYN producing strain
**Accession Number(s)_16S**	HQ407325	**Isolator**	Martin Saker
**Accession Number(s)_others (product/gene /region)**	HQ407337 (rpoC1), HQ407348 (16S-23S rRNA ITS, longer spacer), HQ407361 (16S-23S rRNA ITS, shorter spacer)	**Medium**	Z8
**Collector**	Martin Saker	**Strain Status**	non-axenic, unicyanobacterial
**Collection Date**	Aug-1997	**Preservation type**	subculturing, cryopreserved
**Location**	Australia: Townsville, Queensland	**Light:dark cycle**	12:12-h
**Latitude & Longitude**	unknown	**Temperature (ºC)**	19
		**Light intensity**	10–30 µmol photons m−2 s−1
**References Publicly Available Articles**	Saker and Eaglesham (1999) Toxicon 37, 1065-1077; Saker & Neilan (2001) Appl Environ Microbiol 67,1839–45; Metcalf et al. (2002) FEMS Microbiol. Lett. 216, 159-164; Nogueira et al. (2004) Environ. Toxicol. 19(5), 453-459; Saker et al. (2004) Toxicon 43, 185–194; Berry et al. (2009) Toxicon 53, 289–299; Leão et al. (2009) Eur. J. Phycol. 44, 347-355; Stucken et al. (2009) Syst. Appl. Microbiol. 32, 37–48; Silva & Vasconcelos (2010) Chem. Ecol. 26(4), 263-271; Moreira et al. (2011) Appl. Microbiol. Biotechnol. 92(1), 189-197; Moreira et al. (2011) Curr. Microbiol. 62 (5), 1590-1595; Puerto et al. (2011) Aquat. Toxicol. 101, 109–116; Fraga et al. (2014) Anal. Chim. Acta 850, 57-64; Freitas et al. (2015) Ecotoxicol. Environ. Saf. 116, 59-67; Freitas et al. (2015) Phytochemistry 110, 91-103; Pinho et al. (2015) Chem. Eng. J. 268, 144–152		
**References Publicly Available Theses**	Leão P.N. (2010) PhD Thesis, FCUP, University of Porto, Porto, Portugal; de Matos A. (2014) MSc Dissertation, FCUP, University of Porto, Porto, Portugal; Mendes R.A. (2014) MSc Dissertation, FCUP, University of Porto, Porto, Portugal; Pinho L. (2014) PhD Thesis, FEUP, University of Porto, Porto, Portugal		

Strain ID	**Cylindrospermopsis raciborskii LEGE 99043**		
**Strain Taxonomy**	Cylindrospermopsis raciborskii (Woloszynska) Seenayya, G & Subba Raju, N (1972)		
**Microphotograph 400×**	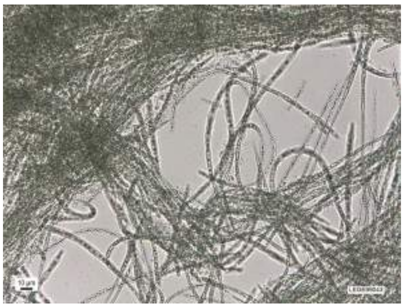	**Microphotograph 1000×**	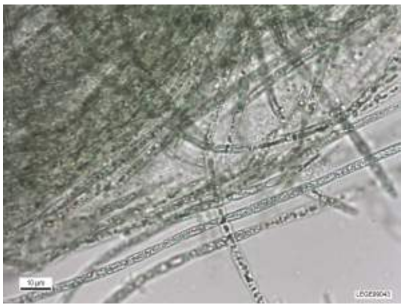
**Coidentity**	n.a.	**Morphometrics (Width × Length or Diameter) (µm)**	2,4±0,6 × 6,9±1,9
**Other Code(s)**	4799, PT1	**Taxonomic notes/diacritical features**	trichomes with vacuolated cells and narrowed toward ends
**Parent Sample**	n.d.	**Macroscopic growth features**	color: cyano; growing homogeneously, but also forming aggregates; mucilaginous
**Isolation Date**	1999	**Environment**	aquatic, freshwater
**Order (Older Classification Scheme)**	Nostocales	**Habitat Sample Description**	water sample
**Order 2014 (Modern Taxonomy)**	Nostocales	**Ecophysiological traits**	nitrogen-fixation, buoyancy
**Taxonomy Notes**		**Notes on ecophysiological traits**	non-CYN producing strain (by HPLC); toxic by mouse bioassay
**Accession Number(s)_16S**	AF516741	**Isolator**	Martin Saker
**Accession Number(s)_others (product/gene /region)**	HQ407330 (rpoC1), HQ407340 (16S-23S rRNA ITS, longer spacer), HQ407353 (16S-23S rRNA ITS, shorter spacer)	**Medium**	Z8
**Collector**	Martin Saker	**Strain Status**	non-axenic, unicyanobacterial
**Collection Date**	1999	**Preservation type**	subculturing, cryopreserved
**Location**	Portugal: Odivelas dam reservoir, Ferreira do Alentejo	**Light:dark cycle**	12:12-h
**Latitude & Longitude**	unknown	**Temperature (ºC)**	19
		**Light intensity**	10–30 µmol photons m−2 s−1
**References Publicly Available Articles**	Saker et al. (2003) Ecotox. Environ. Safe. 55, 243–250; Saker et al. (2004) Limnetica 23(1-2), 145-152; Neilan et al. (2003) Mol. Ecol. 12 (1), 133-140.; Berry et al. (2009) Toxicon 53, 289–299; Leão et al. (2009) Eur. J. Phycol. 44, 347-355; Moreira et al. (2011) Curr. Microbiol. 62 (5), 1590-1595; Antunes et al. (2012) Microb. Ecol. 64 (3): 584-592; Fraga et al. (2014) Anal. Chim. Acta 850, 57-64; Almeida et al. (2015) Toxins 7(8), 2739-2756		
**References Publicly Available Theses**	Leão P.N. (2010) PhD Thesis, FCUP, University of Porto, Porto, Portugal; Antunes J. (2011) MSc Dissertation, FCUP, University of Porto, Porto, Portugal; Macieira R. (2013) MSc Dissertation, FCUP, University of Porto, Porto, Portugal		

Strain ID	**Cylindrospermopsis raciborskii LEGE 99044**		
**Strain Taxonomy**	Cylindrospermopsis raciborskii (Woloszynska) Seenayya, G & Subba Raju, N (1972)		
**Microphotograph 400×**	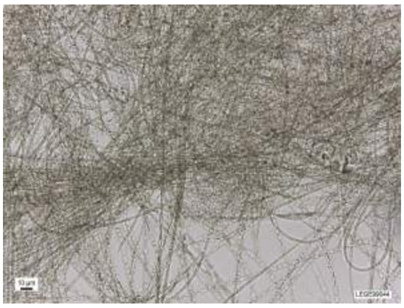	**Microphotograph 1000×**	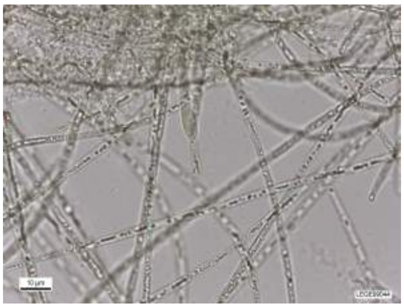
**Coidentity**	n.a.	**Morphometrics (Width × Length or Diameter) (µm)**	2,0±0,4 × 7,8±2,4 (Cells); 4,9±1,2 × 16,6±4,4 (akinetes)
**Other Code(s)**	4899, PT2	**Taxonomic notes/diacritical features**	trichomes with vacuolated cells and narrowed toward ends
**Parent Sample**	n.d.	**Macroscopic growth features**	color: yellowish-green; growing homogeneously
**Isolation Date**	1999	**Environment**	aquatic, freshwater
**Order (Older Classification Scheme)**	Nostocales	**Habitat Sample Description**	water sample
**Order 2014 (Modern Taxonomy)**	Nostocales	**Ecophysiological traits**	nitrogen-fixation, buoyancy
**Taxonomy Notes**		**Notes on ecophysiological traits**	non-CYN producing strain
**Accession Number(s)_16S**	HQ407322	**Isolator**	Martin Saker
**Accession Number(s)_others (product/gene /region)**	HQ407331 (rpoC1), HQ407341 (16S-23S rRNA ITS, longer spacer), HQ407354 (16S-23S rRNA ITS, shorter spacer)	**Medium**	Z8
**Collector**	Martin Saker	**Strain Status**	non-axenic, unicyanobacterial
**Collection Date**	1999	**Preservation type**	subculturing
**Location**	Portugal: Ardila river	**Light:dark cycle**	12:12-h
**Latitude & Longitude**	unknown	**Temperature (ºC)**	19
		**Light intensity**	10–30 µmol photons m−2 s−1
**References Publicly Available Articles**	Saker et al. (2003) Ecotox. Environ. Safe. 55, 243–250; Saker et al. (2004) Limnetica 23(1-2), 145-152; Berry et al. (2009) Toxicon 53, 289–299; Leão et al. (2009) Eur. J. Phycol. 44, 347-355; Moreira et al. (2011) Curr. Microbiol. 62 (5), 1590-1595		
**References Publicly Available Theses**	Leão P.N. (2010) PhD Thesis, FCUP, University of Porto, Porto, Portugal		

Strain ID	**Cylindrospermopsis raciborskii LEGE 99045**		
**Strain Taxonomy**	Cylindrospermopsis raciborskii (Woloszynska) Seenayya, G & Subba Raju, N (1972)		
**Microphotograph 400×**		**Microphotograph 1000×**	
**Coidentity**	n.a.	**Morphometrics (Width × Length or Diameter) (µm)**	
**Other Code(s)**	Marau, Marau 1	**Taxonomic notes/diacritical features**	
**Parent Sample**	n.d.	**Macroscopic growth features**	color: yellowish-green; growing homogeneously
**Isolation Date**	1999	**Environment**	aquatic, freshwater
**Order (Older Classification Scheme)**	Nostocales	**Habitat Sample Description**	water sample
**Order 2014 (Modern Taxonomy)**	Nostocales	**Ecophysiological traits**	nitrogen-fixation, buoyancy
**Taxonomy Notes**		**Notes on ecophysiological traits**	non-CYN producing strain (by HPLC); toxic by mouse bioassay
**Accession Number(s)_16S**	AF516739	**Isolator**	Martin Saker
**Accession Number(s)_others (product/gene/region)**	HQ407333 (rpoC1), HQ407343 (16S-23S rRNA ITS, longer spacer), HQ407356 (16S-23S rRNA ITS, shorter spacer)	**Medium**	Z8
**Collector**	Martin Saker	**Strain Status**	non-axenic, unicyanobacterial
**Collection Date**	1999	**Preservation type**	subculturing
**Location**	Portugal: Maranhão dam reservoir, Avis	**Light:dark cycle**	12:12-h
**Latitude & Longitude**	unknown	**Temperature (ºC)**	19
		**Light intensity**	10–30 µmol photons m−2 s−1
**References Publicly Available Articles**	Neilan et al. (2003) Mol. Ecol. 12 (1), 133-140; Berry et al. (2009) Toxicon 53, 289–299; Leão et al. (2009) Eur. J. Phycol. 44, 347-355; Leão et al. (2010) Proc. Natl. Acad. Sci. U.S.A. 107 (25), 11183-11188.; Moreira et al. (2011) Curr. Microbiol. 62 (5), 1590-1595		
**References Publicly Available Theses**	Leão P.N. (2010) PhD Thesis, FCUP, University of Porto, Porto, Portugal		

**Strain ID**	**Cylindrospermopsis raciborskii LEGE 99048**		
**Strain Taxonomy**	Cylindrospermopsis raciborskii (Woloszynska) Seenayya, G & Subba Raju, N (1972)		
**Microphotograph 400×**	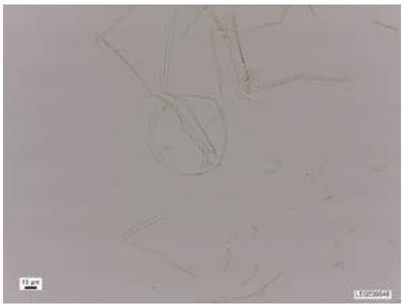	**Microphotograph 1000×**	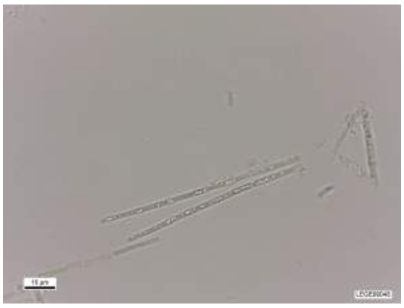
**Coidentity**	n.a.	**Morphometrics (Width × Length or Diameter) (µm)**	1,8±0,4 × 8,4±3,5 (Cells); 1,9±0,4 × 4,5±0,3 (Heterocytes)
**Other Code(s)**	4899 Osc	**Taxonomic notes/diacritical features**	conical-rounded terminal Heterocytes; with sheath
**Parent Sample**		**Macroscopic growth features**	color: yellowish-green; growing homogeneously
**Isolation Date**		**Environment**	aquatic, freshwater
**Order (Older Classification Scheme)**	Nostocales	**Habitat Sample Description**	water sample
**Order 2014 (Modern Taxonomy)**	Nostocales	**Ecophysiological traits**	
**Taxonomy Notes**		**Notes on ecophysiological traits**	
**Accession Number(s)_16S**	KU951711	**Isolator**	Martin Saker
**Accession Number(s)_others (product/gene /region)**		**Medium**	Z8
**Collector**	Martin Saker	**Strain Status**	non-axenic, unicyanobacterial
**Collection Date**	1999	**Preservation type**	subculturing
**Location**	Portugal: Ardila river	**Light:dark cycle**	12:12-h
**Latitude & Longitude**	unknown	**Temperature (ºC)**	19
		**Light intensity**	10–30 µmol photons m−2 s−1
**References Publicly Available Articles**	Leão et al. (2009) Eur. J. Phycol. 44, 347-355		
**References Publicly Available Theses**	Leão P.N. (2010) PhD Thesis, FCUP, University of Porto, Porto, Portugal		

**Strain ID**	**Desmonostoc muscorum LEGE 12446**		
**Strain Taxonomy**	Desmonostoc muscorum (Agardh ex Bornet & Flahault) Hrouzel, P & Ventura, S in Hrouzel et al (2013)		
**Microphotograph 400×**	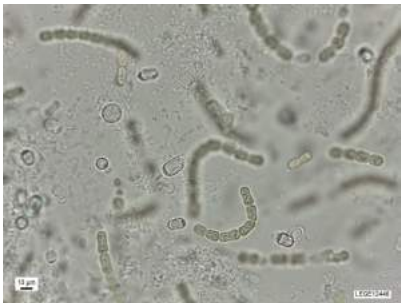	**Microphotograph 1000×**	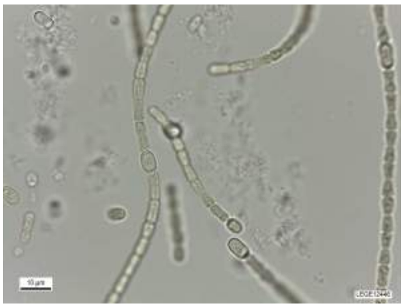
**Coidentity**	n.a.	**Morphometrics (Width × Length or Diameter) (µm)**	2,8±0,6 × 4,5±0,9 (Cells); 6,1±1,5 × 3,6±0,4 (Heterocytes); 7,0 × 4,7 (Akinetes)
**Other Code(s)**	CR1	**Taxonomic notes/diacritical features**	terminal or intercalar heterocytes; with akinetes
**Parent Sample**		**Macroscopic growth features**	color: yellowish-green; growing homogeneously; mucilaginous
**Isolation Date**		**Environment**	terrestrial
**Order (Older Classification Scheme)**	Nostocales	**Habitat Sample Description**	cycad coralloid root (host: Cycas revoluta)
**Order 2014 (Modern Taxonomy)**	Nostocales	**Ecophysiological traits**	EPS production
**Taxonomy Notes**		**Notes on ecophysiological traits**	
**Accession Number(s)_16S**	KU951712	**Isolator**	Ana L. Pereira
**Accession Number(s)_others (product/gene /region)**		**Medium**	BG11o
**Collector**	Ana L. Pereira	**Strain Status**	non-axenic, unicyanobacterial
**Collection Date**	2012	**Preservation type**	subculturing
**Location**	Portugal: Botanical Garden of the University of Coimbra	**Light:dark cycle**	12:12-h
**Latitude & Longitude**	40.20622 N 8.421698 W	**Temperature (ºC)**	19
		**Light intensity**	10–30 µmol photons m−2 s−1
**References Publicly Available Articles**			
**References Publicly Available Theses**			

**Strain ID**	**Dolichospermum flosaquae LEGE 02268**		
**Strain Taxonomy**	Dolichospermum flosaquae (Brébisson ex Bornet & Flahault) Wacklin, P; Hoffmann, L & Komárek, J (2009)		
**Microphotograph 400×**	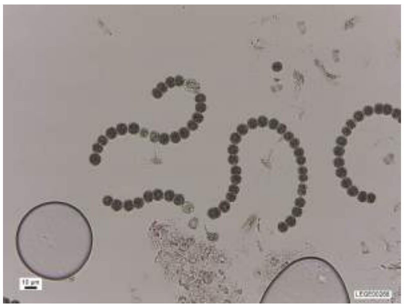	**Microphotograph 1000×**	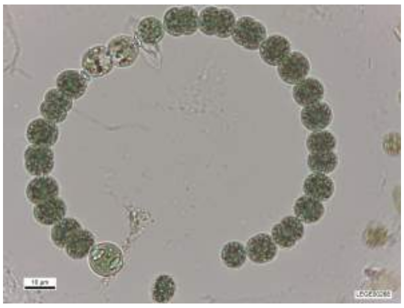
**Coidentity**	n.a.	**Morphometrics (Width × Length or Diameter) (µm)**	8,5±0,3 × 8,1±1,2 (Cells); Ø 9,8±0,7 (Heterocytes); 12,7±0,5 × 15,2±5,0 (akinetes)
**Other Code(s)**	J61, LEANJ.61	**Taxonomic notes/diacritical features**	highly curved or coiled trichomes; with oval akinetes
**Parent Sample**		**Macroscopic growth features**	color: cyano; growing homogeneously, but also forming aggregates
**Isolation Date**		**Environment**	aquatic, freshwater
**Order (Older Classification Scheme)**	Nostocales	**Habitat Sample Description**	water sample
**Order 2014 (Modern Taxonomy)**	Nostocales	**Ecophysiological traits**	akinetes production
**Taxonomy Notes**		**Notes on ecophysiological traits**	
**Accession Number(s)_16S**	KU951713	**Isolator**	Joana Osswald
**Accession Number(s)_others (product/gene /region)**		**Medium**	
**Collector**	Joana Oswald	**Strain Status**	non-axenic, unicyanobacterial
**Collection Date**	2002/07/25	**Preservation type**	subculturing
**Location**	Portugal: Cansado, Montargil	**Light:dark cycle**	12:12-h
**Latitude & Longitude**	unknown	**Temperature (ºC)**	19
		**Light intensity**	10–30 µmol photons m−2 s−1
**References Publicly Available Articles**			
**References Publicly Available Theses**			

Strain ID	**Dolichospermum flosaquae LEGE 04289**		
**Strain Taxonomy**	Dolichospermum flosaquae (Brébisson ex Bornet & Flahault) Wacklin, P; Hoffmann, L & Komárek, J (2009)		
**Microphotograph 400×**	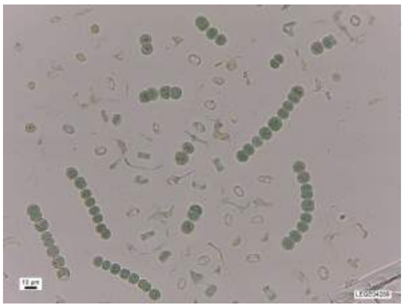	**Microphotograph 1000×**	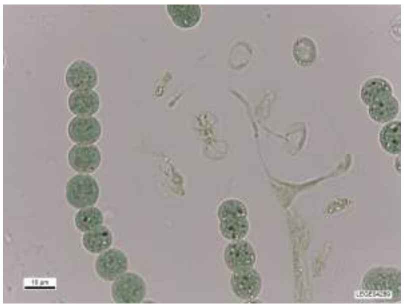
**Coidentity**	n.a.	**Morphometrics (Width × Length or Diameter) (µm)**	Ø 9,7±1,0 (Cells); 15,8±5,7 × 29,6±11,6 (Akinetes)
**Other Code(s)**	J83, LEANJ.83	**Taxonomic notes/diacritical features**	highly curved or coiled trichomes
**Parent Sample**		**Macroscopic growth features**	color: cyano; growing homogeneously
**Isolation Date**		**Environment**	aquatic, freshwater
**Order (Older Classification Scheme)**	Nostocales	**Habitat Sample Description**	water sample
**Order 2014 (Modern Taxonomy)**	Nostocales	**Ecophysiological traits**	toxin production
**Taxonomy Notes**		**Notes on ecophysiological traits**	ANTX-a producing strain
**Accession Number(s)_16S**	KU951714	**Isolator**	Joana Osswald
**Accession Number(s)_others (product/gene /region)**		**Medium**	Z8
**Collector**	Joana Osswald	**Strain Status**	non-axenic, unicyanobacterial
**Collection Date**	2004/07/13	**Preservation type**	subculturing
**Location**	Portugal: Marco de Canaveses	**Light:dark cycle**	12:12-h
**Latitude & Longitude**	unknown	**Temperature (ºC)**	19
		**Light intensity**	10–30 µmol photons m−2 s−1
**References Publicly Available Articles**			
**References Publicly Available Theses**	Osswald J. (2007) PhD Thesis, FCUP, University of Porto, Porto, Portugal; Chaves R. (2013) MSc Dissertation, ICBAS, University of Porto, Porto, Portugal		

**Strain ID**	**Dolichospermum sp. LEGE 00234**		
**Strain Taxonomy**	Dolichospermum (Ralfs ex Bornet & Flahault) Wacklin, P; Hoffmann, L & Komárek, J (2009)		
**Microphotograph 400×**	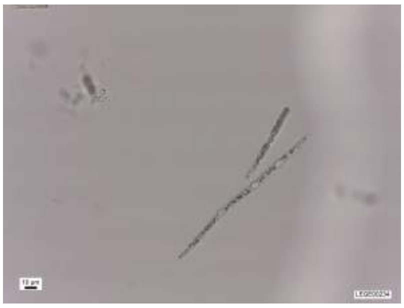	**Microphotograph 1000×**	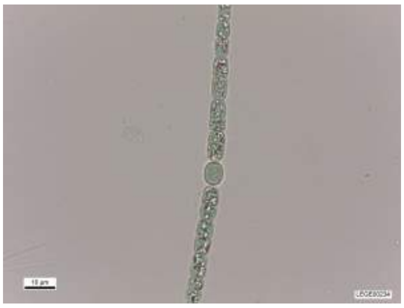
**Coidentity**	n.a.	**Morphometrics (Width × Length or Diameter) (µm)**	4,8±1,2 × 5,5±1,5 (Cells); 5,4±0,6 × 6,2±1,0 (Heterocytes); 4,7±0,7 × 20,4±2,4 (akinetes)
**Other Code(s)**	J3, LEANJ.3	**Taxonomic notes/diacritical features**	highly vacuolated trichomes, causing bright refractive cells; with akinetes
**Parent Sample**		**Macroscopic growth features**	color: cyano; growing homogeneously
**Isolation Date**		**Environment**	aquatic, freshwater
**Order (Older Classification Scheme)**	Nostocales	**Habitat Sample Description**	water sample
**Order 2014 (Modern Taxonomy)**	Nostocales	**Ecophysiological traits**	
**Taxonomy Notes**		**Notes on ecophysiological traits**	non-ANTX-a producing strain
**Accession Number(s)_16S**	KU951715	**Isolator**	Joana Osswald
**Accession Number(s)_others (product/gene /region)**		**Medium**	Z8
**Collector**	Joana Oswald	**Strain Status**	non-axenic, unicyanobacterial
**Collection Date**	2000/07/07	**Preservation type**	subculturing
**Location**	Portugal: Maranhão dam reservoir, Benavila	**Light:dark cycle**	12:12-h
**Latitude & Longitude**	unknown	**Temperature (ºC)**	19
		**Light intensity**	10–30 µmol photons m−2 s−1
**References Publicly Available Articles**	Fraga et al. (2014) Anal. Chim. Acta 850, 57-64; Sanchez et al. (2014) Toxins 6, 402-415		
**References Publicly Available Theses**	Osswald J. (2002) MSc Dissertation, FCUP, University of Porto, Porto, Portugal		

**Strain ID**	**Dolichospermum sp. LEGE 00240**		
**Strain Taxonomy**	Dolichospermum (Ralfs ex Bornet & Flahault) Wacklin, P; Hoffmann, L & Komárek, J (2009)		
**Microphotograph 400×**	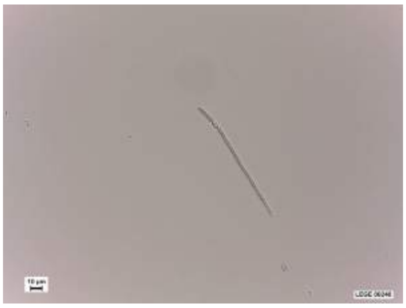	**Microphotograph 1000×**	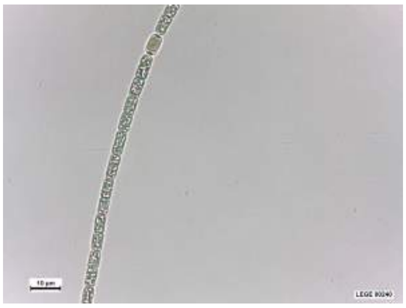
**Coidentity**	n.a.	**Morphometrics (Width × Length or Diameter) (µm)**	3,3±0,3 × 7,9±3,9 (Cells); 4,4±0,9 × 6,0±0,6 (Heterocytes); 4,6±1,1 × 27,0±4,2 (akinetes)
**Other Code(s)**	J12, LEANJ.12	**Taxonomic notes/diacritical features**	highly vacuolated trichomes, causing bright refractive cells; with akinetes
**Parent Sample**		**Macroscopic growth features**	color: cyano; growing homogeneously
**Isolation Date**		**Environment**	aquatic, freshwater
**Order (Older Classification Scheme)**	Nostocales	**Habitat Sample Description**	water sample
**Order 2014 (Modern Taxonomy)**	Nostocales	**Ecophysiological traits**	toxin production
**Taxonomy Notes**		**Notes on ecophysiological traits**	ANTX-a producing strain
**Accession Number(s)_16S**	KU951716	**Isolator**	Joana Osswald
**Accession Number(s)_others (product/gene /region)**		**Medium**	Z8
**Collector**	Joana Oswald	**Strain Status**	non-axenic, unicyanobacterial
**Collection Date**	2000/07/07	**Preservation type**	subculturing
**Location**	Portugal: Maranhão dam reservoir, campsite at Avis	**Light:dark cycle**	12:12-h
**Latitude & Longitude**	unknown	**Temperature (ºC)**	19
		**Light intensity**	10–30 µmol photons m−2 s−1
**References Publicly Available Articles**			
**References Publicly Available Theses**	Osswald J. (2002) MSc Dissertation, FCUP, University of Porto, Porto, Portugal; Osswald J. (2007) PhD Thesis, FCUP, University of Porto, Porto, Portugal		

**Strain ID**	**Dolichospermum sp. LEGE 00241**		
**Strain Taxonomy**	Dolichospermum (Ralfs ex Bornet & Flahault) Wacklin, P; Hoffmann, L & Komárek, J (2009)		
**Microphotograph 400×**	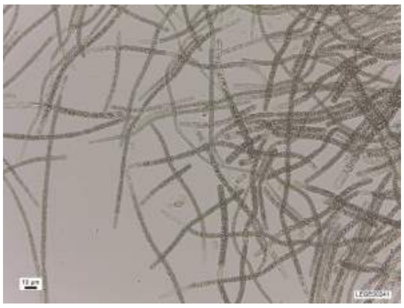	**Microphotograph 1000×**	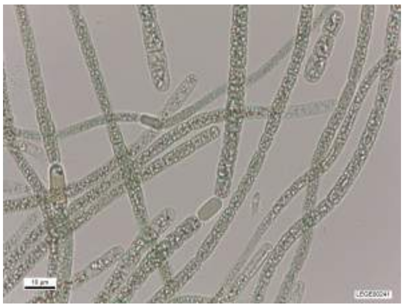
**Coidentity**	n.a.	**Morphometrics (Width × Length or Diameter) (µm)**	4,0±0,6 × 5,4±0,9 (Cells); 4,6±0,3 × 8,0±1,9 (Heterocytes); 4,0±1,1 × 9,9±3,7 (akinetes)
**Other Code(s)**	J14, LEANJ.14	**Taxonomic notes/diacritical features**	highly vacuolated trichomes, causing bright refractive cells; with akinetes
**Parent Sample**		**Macroscopic growth features**	color: cyano; forming a smooth biofilm and aggregates
**Isolation Date**		**Environment**	aquatic, freshwater
**Order (Older Classification Scheme)**	Nostocales	**Habitat Sample Description**	water sample
**Order 2014 (Modern Taxonomy)**	Nostocales	**Ecophysiological traits**	toxin production
**Taxonomy Notes**		**Notes on ecophysiological traits**	ANTX-a producing strain
**Accession Number(s)_16S**	KU951717	**Isolator**	Joana Osswald
**Accession Number(s)_others (product/gene /region)**		**Medium**	Z8
**Collector**	Joana Oswald	**Strain Status**	non-axenic, unicyanobacterial
**Collection Date**	2000/07/07	**Preservation type**	subculturing, cryopreserved
**Location**	Portugal: Maranhão dam reservoir, campsite at Avis	**Light:dark cycle**	12:12-h
**Latitude & Longitude**	unknown	**Temperature (ºC)**	19
		**Light intensity**	10–30 µmol photons m−2 s−1
**References Publicly Available Articles**			
**References Publicly Available Theses**	Osswald J. (2002) MSc Dissertation, FCUP, University of Porto, Porto, Portugal; Osswald J. (2007) PhD Thesis, FCUP, University of Porto, Porto, Portugal; Chaves R. (2013) MSc Dissertation, ICBAS, University of Porto, Porto, Portugal		

**Strain ID**	**Dolichospermum sp. LEGE 00246**		
**Strain Taxonomy**	Dolichospermum (Ralfs ex Bornet & Flahault) Wacklin, P; Hoffmann, L & Komárek, J (2009)		
**Microphotograph 400×**	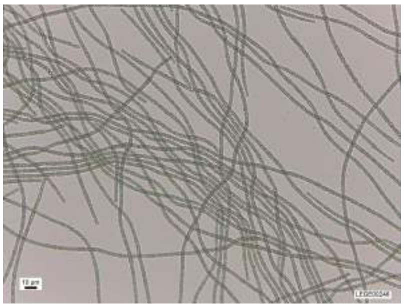	**Microphotograph 1000×**	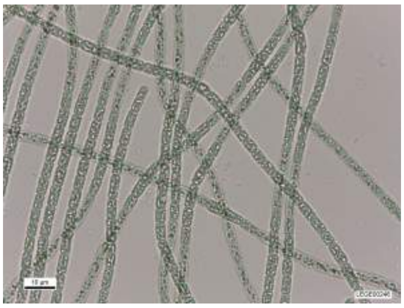
**Coidentity**	n.a.	**Morphometrics (Width × Length or Diameter) (µm)**	2,9±0,3 × 4,9±0,8
**Other Code(s)**	J19, LEANJ.19	**Taxonomic notes/diacritical features**	highly vacuolated trichomes, causing bright refractive cells
**Parent Sample**		**Macroscopic growth features**	color: cyano; growing homogeneously, but also forming aggregates; mucilaginous
**Isolation Date**		**Environment**	aquatic, freshwater
**Order (Older Classification Scheme)**	Nostocales	**Habitat Sample Description**	water sample
**Order 2014 (Modern Taxonomy)**	Nostocales	**Ecophysiological traits**	EPS production
**Taxonomy Notes**		**Notes on ecophysiological traits**	
**Accession Number(s)_16S**	KU951718	**Isolator**	Joana Osswald
**Accession Number(s)_others (product/gene /region)**		**Medium**	Z8
**Collector**	Joana Oswald	**Strain Status**	non-axenic, unicyanobacterial
**Collection Date**	2000/07/07	**Preservation type**	subculturing
**Location**	Portugal: Maranhão dam reservoir, Benavila	**Light:dark cycle**	12:12-h
**Latitude & Longitude**	unknown	**Temperature (ºC)**	19
		**Light intensity**	10–30 µmol photons m−2 s−1
**References Publicly Available Articles**			
**References Publicly Available Theses**	Osswald J. (2002) MSc Dissertation, FCUP, University of Porto, Porto, Portugal; Chaves R. (2013) MSc Dissertation, ICBAS, University of Porto, Porto, Portugal		

**Strain ID**	**Dolichospermum sp. LEGE 00248**		
**Strain Taxonomy**	Dolichospermum (Ralfs ex Bornet & Flahault) Wacklin, P; Hoffmann, L & Komárek, J (2009)		
**Microphotograph 400×**	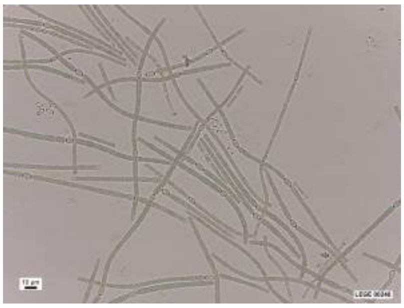	**Microphotograph 1000×**	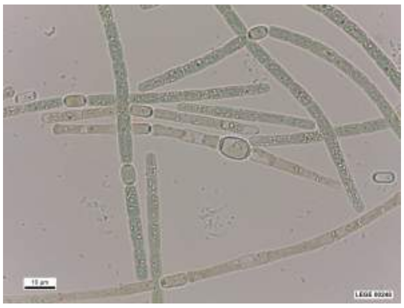
**Coidentity**	n.a.	**Morphometrics (Width × Length or Diameter) (µm)**	3,7±0,5 × 6,4±1,2 (Cells); 3,7±0,6 × 6,4±1,0 (Heterocytes)
**Other Code(s)**	J21, LEANJ.21	**Taxonomic notes/diacritical features**	
**Parent Sample**		**Macroscopic growth features**	color: cyano; growing homogeneously
**Isolation Date**		**Environment**	aquatic, freshwater
**Order (Older Classification Scheme)**	Nostocales	**Habitat Sample Description**	water sample
**Order 2014 (Modern Taxonomy)**	Nostocales	**Ecophysiological traits**	
**Taxonomy Notes**		**Notes on ecophysiological traits**	
**Accession Number(s)_16S**	KU951719	**Isolator**	Joana Osswald
**Accession Number(s)_others (product/gene /region)**		**Medium**	Z8
**Collector**	Joana Oswald	**Strain Status**	non-axenic, unicyanobacterial
**Collection Date**	2000/07/07	**Preservation type**	subculturing
**Location**	Portugal: Maranhão dam reservoir, Benavila	**Light:dark cycle**	12:12-h
**Latitude & Longitude**	unknown	**Temperature (ºC)**	19
		**Light intensity**	10–30 µmol photons m−2 s−1
**References Publicly Available Articles**			
**References Publicly Available Theses**	Osswald J. (2002) MSc Dissertation, FCUP, University of Porto, Porto, Portugal; Chaves R. (2013) MSc Dissertation, ICBAS, University of Porto, Porto, Portugal		

Strain ID	**Dolichospermum sp. LEGE 00259**		
**Strain Taxonomy**	Dolichospermum (Ralfs ex Bornet & Flahault) Wacklin, P; Hoffmann, L & Komárek, J (2009)		
**Microphotograph 400×**	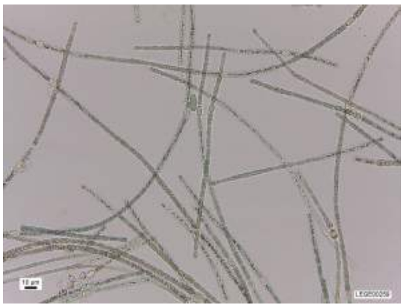	**Microphotograph 1000×**	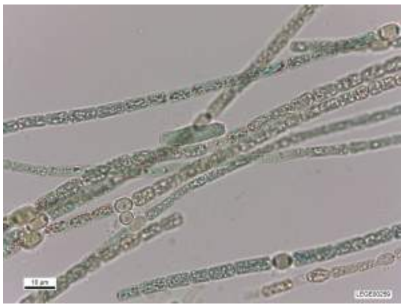
**Coidentity**	n.a.	**Morphometrics (Width × Length or Diameter) (µm)**	3,8±0,8 × 7,1±2,1 (cells); 4,7±1,4 × 5,5±0,8 (heterocytes); 5,2±0,4 × 23,0±9,0 (akinetes)
**Other Code(s)**	J46, LEANJ.46	**Taxonomic notes/diacritical features**	highly vacuolated trichomes, causing bright refractive cells; with akinetes
**Parent Sample**		**Macroscopic growth features**	color: cyano; growing homogeneously, but also forming aggregates
**Isolation Date**		**Environment**	aquatic, freshwater
**Order (Older Classification Scheme)**	Nostocales	**Habitat Sample Description**	water sample
**Order 2014 (Modern Taxonomy)**	Nostocales	**Ecophysiological traits**	toxin production
**Taxonomy Notes**		**Notes on ecophysiological traits**	ANTX-a producing strain
**Accession Number(s)_16S**	KU951720	**Isolator**	Joana Osswald
**Accession Number(s)_others (product/gene /region)**		**Medium**	Z8
**Collector**	Joana Oswald	**Strain Status**	non-axenic, unicyanobacterial
**Collection Date**	2000/07/07	**Preservation type**	subculturing
**Location**	Portugal: Maranhão dam reservoir, Benavila	**Light:dark cycle**	14:10-h
**Latitude & Longitude**	unknown	**Temperature (ºC)**	25
		**Light intensity**	10–30 µmol photons m−2 s−1
**References Publicly Available Articles**	Leão et al. (2009) Eur. J. Phycol. 44, 347-355		
**References Publicly Available Theses**	Osswald J. (2002) MSc Dissertation, FCUP, University of Porto, Porto, Portugal; Osswald J. (2007) PhD Thesis, FCUP, University of Porto, Porto, Portugal; Leão P.N. (2010) PhD Thesis, FCUP, University of Porto, Porto, Portugal; Chaves R. (2013) MSc Dissertation, ICBAS, University of Porto, Porto, Portugal		

Strain ID	**Dolichospermum sp. LEGE 00263**		
**Strain Taxonomy**	Dolichospermum (Ralfs ex Bornet & Flahault) Wacklin, P; Hoffmann, L & Komárek, J (2009)		
**Microphotograph 400×**	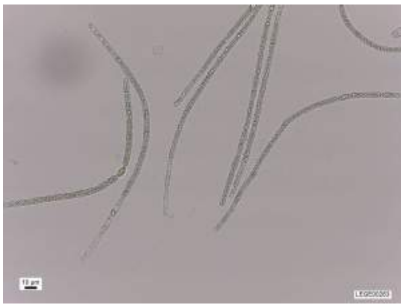	**Microphotograph 1000×**	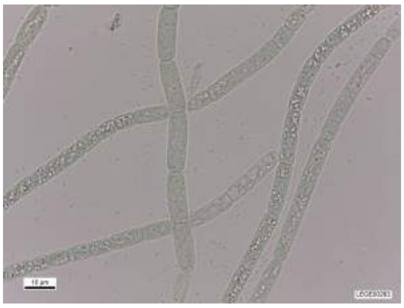
**Coidentity**	n.a.	**Morphometrics (Width × Length or Diameter) (µm)**	4,3±1,0 × 8,7±2,9 (Cells); 5,1±1,0 × 9,5±0,5 (heterocytes)
**Other Code(s)**	J53, LEANJ.53	**Taxonomic notes/diacritical features**	vacuolated cells
**Parent Sample**		**Macroscopic growth features**	color: cyano; growing homogeneously, but also forming aggregates
**Isolation Date**		**Environment**	aquatic, freshwater
**Order (Older Classification Scheme)**	Nostocales	**Habitat Sample Description**	water sample
**Order 2014 (Modern Taxonomy)**	Nostocales	**Ecophysiological traits**	
**Taxonomy Notes**		**Notes on ecophysiological traits**	
**Accession Number(s)_16S**	KU951721	**Isolator**	Joana Osswald
**Accession Number(s)_others (product/gene /region)**		**Medium**	Z8
**Collector**	Joana Oswald	**Strain Status**	non-axenic, unicyanobacterial
**Collection Date**	2000/07/07	**Preservation type**	subculturing
**Location**	Portugal: Maranhão dam reservoir, Benavila	**Light:dark cycle**	12:12-h
**Latitude & Longitude**	unknown	**Temperature (ºC)**	19
		**Light intensity**	10–30 µmol photons m−2 s−1
**References Publicly Available Articles**			
**References Publicly Available Theses**	Osswald J. (2002) MSc Dissertation, FCUP, University of Porto, Porto, Portugal		

**Strain ID**	**Dolichospermum sp. LEGE 03277**		
**Strain Taxonomy**	Dolichospermum (Ralfs ex Bornet & Flahault) Wacklin, P; Hoffmann, L & Komárek, J (2009)		
**Microphotograph 400×**	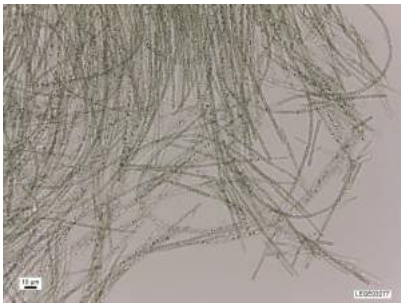	**Microphotograph 1000×**	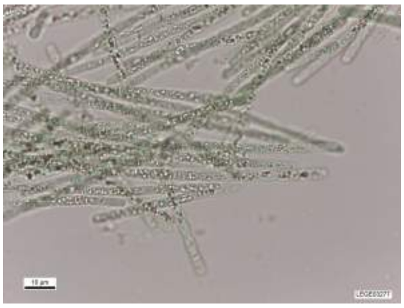
**Coidentity**	n.a.	**Morphometrics (Width × Length or Diameter) (µm)**	2,6±0,5 × 6,0±2,0
**Other Code(s)**	J73, LEANJ.73	**Taxonomic notes/diacritical features**	vacuolated cells
**Parent Sample**		**Macroscopic growth features**	color: cyano; forming a smooth biofilm
**Isolation Date**		**Environment**	aquatic, freshwater
**Order (Older Classification Scheme)**	Nostocales	**Habitat Sample Description**	water sample, dam reservoir
**Order 2014 (Modern Taxonomy)**	Nostocales	**Ecophysiological traits**	
**Taxonomy Notes**		**Notes on ecophysiological traits**	non-ANTX-a producing strain
**Accession Number(s)_16S**	KU951722	**Isolator**	Joana Osswald
**Accession Number(s)_others (product/gene /region)**		**Medium**	Z8
**Collector**	Joana Oswald	**Strain Status**	non-axenic, unicyanobacterial
**Collection Date**	2003/10/29	**Preservation type**	subculturing
**Location**	Portugal: Crestuma-Lever reservoir	**Light:dark cycle**	12:12-h
**Latitude & Longitude**	unknown	**Temperature (ºC)**	19
		**Light intensity**	10–30 µmol photons m−2 s−1
**References Publicly Available Articles**			
**References Publicly Available Theses**	Macieira R. (2013) MSc Dissertation, FCUP, University of Porto, Porto, Portugal		

**Strain ID**	**Dolichospermum sp. LEGE 03278**		
**Strain Taxonomy**	Dolichospermum (Ralfs ex Bornet & Flahault) Wacklin, P; Hoffmann, L & Komárek, J (2009)		
**Microphotograph 400×**	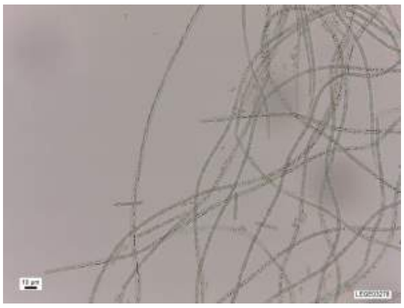	**Microphotograph 1000×**	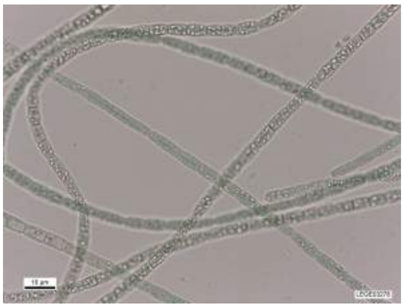
**Coidentity**	n.a.	**Morphometrics (Width × Length or Diameter) (µm)**	3,1±0,6 × 5,6±1,3
**Other Code(s)**	J74, LEANJ.74	**Taxonomic notes/diacritical features**	vacuolated cells
**Parent Sample**		**Macroscopic growth features**	color: cyano; forming a smooth biofilm
**Isolation Date**		**Environment**	aquatic, freshwater
**Order (Older Classification Scheme)**	Nostocales	**Habitat Sample Description**	water sample, dam reservoir
**Order 2014 (Modern Taxonomy)**	Nostocales	**Ecophysiological traits**	
**Taxonomy Notes**		**Notes on ecophysiological traits**	
**Accession Number(s)_16S**	KU951723	**Isolator**	Joana Osswald
**Accession Number(s)_others (product/gene /region)**		**Medium**	Z8
**Collector**	Joana Oswald	**Strain Status**	non-axenic, unicyanobacterial
**Collection Date**	2003/10/29	**Preservation type**	subculturing
**Location**	Portugal: Crestuma-Lever reservoir	**Light:dark cycle**	12:12-h
**Latitude & Longitude**	unknown	**Temperature (ºC)**	19
		**Light intensity**	10–30 µmol photons m−2 s−1
**References Publicly Available Articles**	Leão et al. (2009) Eur. J. Phycol. 44, 347-355; Leão et al. (2010) Proc. Natl. Acad. Sci. U.S.A. 107 (25), 11183-11188		
**References Publicly Available Theses**	Leão P.N. (2010) PhD Thesis, FCUP, University of Porto, Porto, Portugal; Macieira R. (2013) MSc Dissertation, FCUP, University of Porto, Porto, Portugal		

**Strain ID**	**Fortiea sp. LEGE XX443**		
**Strain Taxonomy**	Fortiea De Toni, G (1936)		
**Microphotograph 400×**	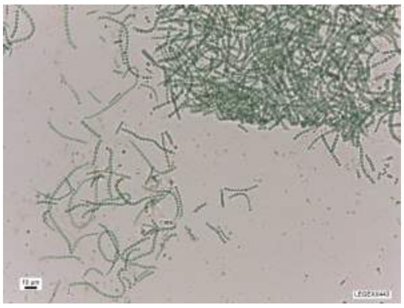	**Microphotograph 1000×**	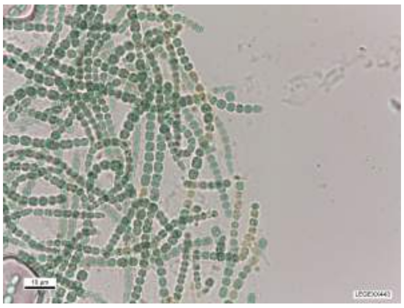
**Coidentity**	n.a.	**Morphometrics (Width × Length or Diameter) (µm)**	2,4±0,4 × 2,5±0,4
**Other Code(s)**		**Taxonomic notes/diacritical features**	trichomes highly constricted at cross-walls; some conical-rounded terminal cells
**Parent Sample**		**Macroscopic growth features**	color: cyano; forming a firm biofilm
**Isolation Date**		**Environment**	unknown
**Order (Older Classification Scheme)**	Nostocales	**Habitat Sample Description**	unknown
**Order 2014 (Modern Taxonomy)**	Nostocales	**Ecophysiological traits**	nitrogen-fixation
**Taxonomy Notes**		**Notes on ecophysiological traits**	
**Accession Number(s)_16S**	KU951724	**Isolator**	unknown
**Accession Number(s)_others (product/gene /region)**		**Medium**	Z8
**Collector**	unknown	**Strain Status**	non-axenic, unicyanobacterial
**Collection Date**	unknown	**Preservation type**	subculturing
**Location**	unknown	**Light:dark cycle**	12:12-h
**Latitude & Longitude**	unknown	**Temperature (ºC)**	19
		**Light intensity**	10–30 µmol photons m−2 s−1
**References Publicly Available Articles**			
**References Publicly Available Theses**			

**Strain ID**	**Geitlerinema sp. LEGE 11390**		
**Strain Taxonomy**	Geitlerinema (Anagnostidis & Komárek) Anagnostidis, K (1989)		
**Microphotograph 400×**	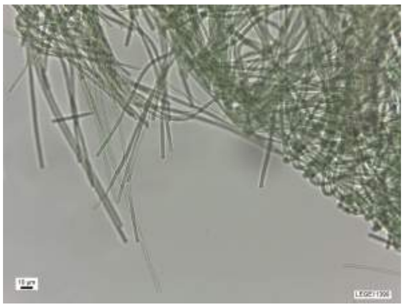	**Microphotograph 1000×**	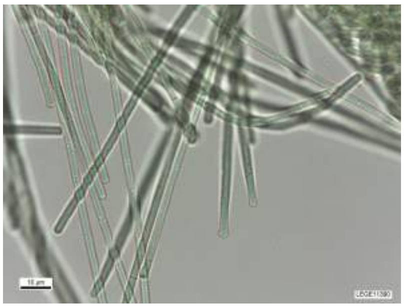
**Coidentity**	n.a.	**Morphometrics (Width × Length or Diameter) (µm)**	2,1±0,2 × 7,6±1,6
**Other Code(s)**		**Taxonomic notes/diacritical features**	gliding motility
**Parent Sample**	EB1-1-ABAAA	**Macroscopic growth features**	color: cyano; forming a firm biofilm
**Isolation Date**		**Environment**	aquatic, hypersaline
**Order (Older Classification Scheme)**	Oscillatoriales	**Habitat Sample Description**	upper layer of a microbial mat
**Order 2014 (Modern Taxonomy)**	Synechococcales**	**Ecophysiological traits**	
**Taxonomy Notes**	**inconsistent; assignment supported by phylogeny	**Notes on ecophysiological traits**	
**Accession Number(s)_16S**	KU951725	**Isolator**	Vitor Ramos
**Accession Number(s)_others (product/gene /region)**		**Medium**	MN w/ vit. B12
**Collector**	Frederico Sobrinho/Sinda Carvalhal	**Strain Status**	non-axenic, unicyanobacterial
**Collection Date**	9-Feb-2011	**Preservation type**	subculturing
**Location**	Brazil: Araruama (main) lagoon, in the state of Rio de Janeiro	**Light:dark cycle**	12:12-h
**Latitude & Longitude**	22.94333 S 42.10056 W	**Temperature (ºC)**	19
		**Light intensity**	10–30 µmol photons m−2 s−1
**References Publicly Available Articles**			
**References Publicly Available Theses**			

**Strain ID**	**Geitlerinema sp. LEGE 11391**		
**Strain Taxonomy**	Geitlerinema (Anagnostidis & Komárek) Anagnostidis, K (1989)		
**Microphotograph 400×**	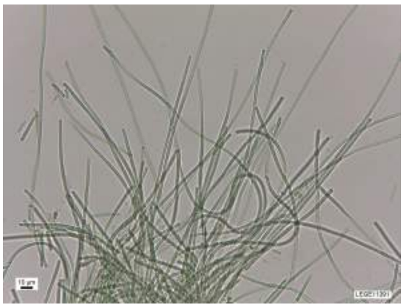	**Microphotograph 1000×**	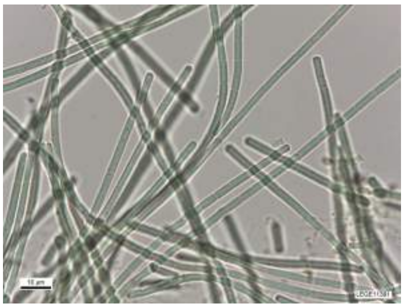
**Coidentity**	n.a.	**Morphometrics (Width × Length or Diameter) (µm)**	2,2±0,3 × 6,8±1,7
**Other Code(s)**		**Taxonomic notes/diacritical features**	gliding motility
**Parent Sample**	EB1-3-ECAA	**Macroscopic growth features**	color: cyano; forming a firm biofilm
**Isolation Date**		**Environment**	aquatic, hypersaline
**Order (Older Classification Scheme)**	Oscillatoriales	**Habitat Sample Description**	upper layer of a microbial mat
**Order 2014 (Modern Taxonomy)**	Synechococcales**	**Ecophysiological traits**	
**Taxonomy Notes**	**inconsistent; assignment supported by phylogeny	**Notes on ecophysiological traits**	
**Accession Number(s)_16S**	KU951726	**Isolator**	Vitor Ramos
**Accession Number(s)_others (product/gene /region)**		**Medium**	MN w/ vit. B12
**Collector**	Frederico Sobrinho/Sinda Carvalhal	**Strain Status**	non-axenic, unicyanobacterial
**Collection Date**	9-Feb-2011	**Preservation type**	subculturing
**Location**	Brazil: Araruama (main) lagoon, in the state of Rio de Janeiro	**Light:dark cycle**	12:12-h
**Latitude & Longitude**	22.94333 S 42.10056 W	**Temperature (ºC)**	19
		**Light intensity**	10–30 µmol photons m−2 s−1
**References Publicly Available Articles**			
**References Publicly Available Theses**			

**Strain ID**	**Geitlerinema sp. LEGE 11393**		
**Strain Taxonomy**	Geitlerinema (Anagnostidis & Komárek) Anagnostidis, K (1989)		
**Microphotograph 400×**	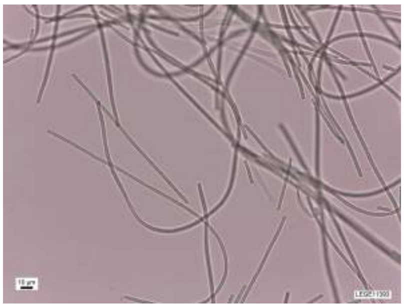	**Microphotograph 1000×**	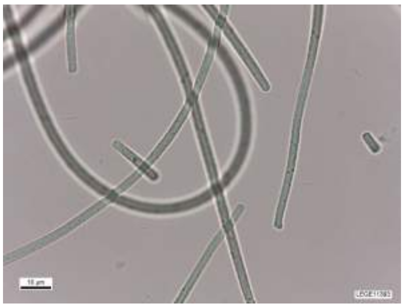
**Coidentity**	n.a.	**Morphometrics (Width × Length or Diameter) (µm)**	2,6±0,3 × 3,9±0,6
**Other Code(s)**		**Taxonomic notes/diacritical features**	gliding motility
**Parent Sample**	EB5-1-DAAA (EB5=EB2)	**Macroscopic growth features**	color: cyano; forming a firm biofilm
**Isolation Date**		**Environment**	aquatic, hypersaline
**Order (Older Classification Scheme)**	Oscillatoriales	**Habitat Sample Description**	upper layer of a microbial mat
**Order 2014 (Modern Taxonomy)**	Synechococcales**	**Ecophysiological traits**	
**Taxonomy Notes**	**inconsistent; assignment supported by phylogeny	**Notes on ecophysiological traits**	
**Accession Number(s)_16S**	KU951727	**Isolator**	Vitor Ramos
**Accession Number(s)_others (product/gene /region)**		**Medium**	MN w/ vit. B12
**Collector**	Frederico Sobrinho/Sinda Carvalhal	**Strain Status**	non-axenic, unicyanobacterial
**Collection Date**	9-Feb-2011	**Preservation type**	subculturing
**Location**	Brazil: Pernambuco lagoon (Araruama system), in the state of Rio de Janeiro	**Light:dark cycle**	12:12-h
**Latitude & Longitude**	22.93056 S 42.32389 W	**Temperature (ºC)**	19
		**Light intensity**	10–30 µmol photons m−2 s−1
**References Publicly Available Articles**			
**References Publicly Available Theses**			

**Strain ID**	**Geitlerinema sp. LEGE 11396**		
**Strain Taxonomy**	Geitlerinema (Anagnostidis & Komárek) Anagnostidis, K (1989)		
**Microphotograph 400×**	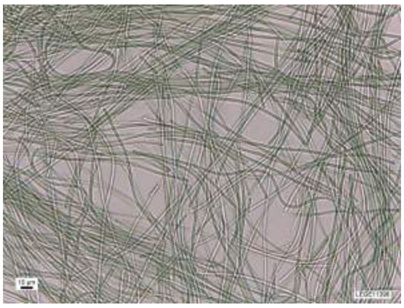	**Microphotograph 1000×**	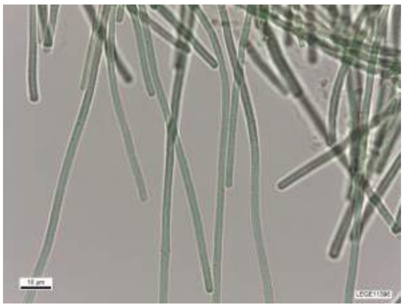
**Coidentity**	n.a.	**Morphometrics (Width × Length or Diameter) (µm)**	2,2±0,5 × 6,7±1,8
**Other Code(s)**		**Taxonomic notes/diacritical features**	gliding motility
**Parent Sample**	EB1-3-EB1A	**Macroscopic growth features**	color: cyano; forming a firm biofilm
**Isolation Date**		**Environment**	aquatic, hypersaline
**Order (Older Classification Scheme)**	Oscillatoriales	**Habitat Sample Description**	upper layer of a microbial mat
**Order 2014 (Modern Taxonomy)**	Synechococcales*	**Ecophysiological traits**	
**Taxonomy Notes**	*inconsistent; assignment supported by phylogeny	**Notes on ecophysiological traits**	
**Accession Number(s)_16S**	KU951728	**Isolator**	Vitor Ramos
**Accession Number(s)_others (product/gene /region)**		**Medium**	MN w/ vit. B12
**Collector**	Frederico Sobrinho/Sinda Carvalhal	**Strain Status**	non-axenic, unicyanobacterial
**Collection Date**	9-Feb-2011	**Preservation type**	subculturing
**Location**	Brazil: Araruama (main) lagoon, in the state of Rio de Janeiro	**Light:dark cycle**	12:12-h
**Latitude & Longitude**	22.94333 S 42.10056 W	**Temperature (ºC)**	19
		**Light intensity**	10–30 µmol photons m−2 s−1
**References Publicly Available Articles**			
**References Publicly Available Theses**			

**Strain ID**	**Geminobacterium atlanticum LEGE 07459**		
**Strain Taxonomy**	Geminobacterium atlanticum Ramos, V; Brito, A & Kaštovský, J in Brito et al (2017)		
**Microphotograph 400×**	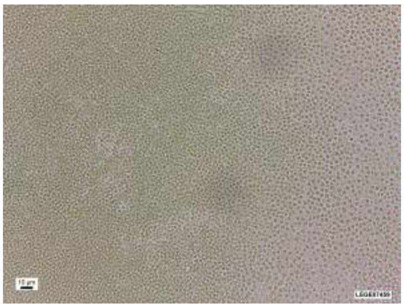	**Microphotograph 1000×**	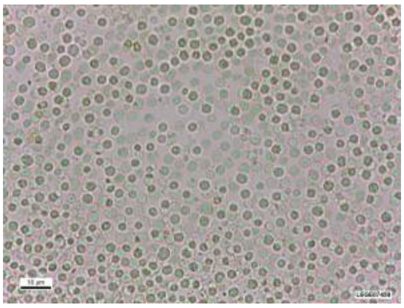
**Coidentity**	n.a.	**Morphometrics (Width × Length or Diameter) (µm)**	Ø 2,4±0,4
**Other Code(s)**	LEAN 075.2	**Taxonomic notes/diacritical features**	
**Parent Sample**	P.07 MAR 4 - p96, dil. 1/16	**Macroscopic growth features**	color: cyano; forming aggregates
**Isolation Date**	2007	**Environment**	aquatic, marine
**Order (Older Classification Scheme)**	Chroococcales	**Habitat Sample Description**	intertidal zone, on a Patella sp. shell
**Order 2014 (Modern Taxonomy)**	Chroococcales	**Ecophysiological traits**	
**Taxonomy Notes**	This is the Type strain of Geminobacterium atlanticum Ramos, Brito et Kaštovský gen. nov., sp. nov.	**Notes on ecophysiological traits**	
**Accession Number(s)_16S**	KU951882	**Isolator**	Vitor Ramos
**Accession Number(s)_others (product/gene /region)**	KR676352 (16S rRNA, 16S-23S rRNA ITS, 23S rRNA)	**Medium**	MN w/ vit. B12
**Collector**	Rui Seabra/Vitor Ramos	**Strain Status**	non-axenic, unicyanobacterial
**Collection Date**	2007/05/02	**Preservation type**	subculturing, cryopreserved
**Location**	Portugal: Praia do Martinhal, Vila do Bispo	**Light:dark cycle**	12:12-h
**Latitude & Longitude**	37.01869 N 8.926714 W	**Temperature (ºC)**	19
		**Light intensity**	10–30 µmol photons m−2 s−1
**References Publicly Available Articles**	Brito et al. (2017) Mol. Phylog. Evol. doi:10.1016/j.ympev.2017.03.006		
**References Publicly Available Theses**			

Strain ID	**Gloeocapsa-like sp. LEGE 10406**		
**Strain Taxonomy**	Gloeocapsa Kützing, FT (1843)		
**Microphotograph 400×**	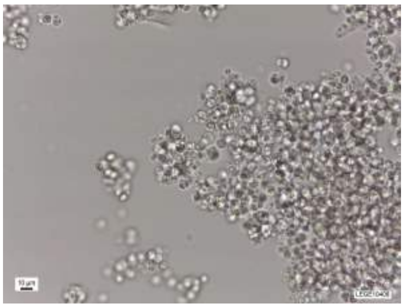	**Microphotograph 1000×**	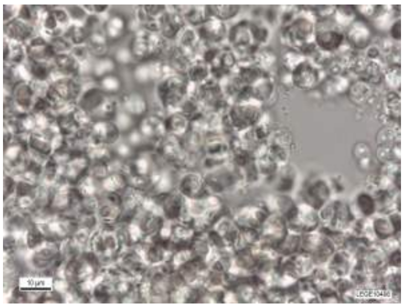
**Coidentity**	n.a.	**Morphometrics (Width × Length or Diameter) (µm)**	Ø 3,6±0,6 (more or less spherical cells)
**Other Code(s)**		**Taxonomic notes/diacritical features**	with sheath
**Parent Sample**	S9-BA	**Macroscopic growth features**	color: brownish; forming aggregates, mucilaginous
**Isolation Date**		**Environment**	aquatic, marine
**Order (Older Classification Scheme)**	Chroococcales	**Habitat Sample Description**	intertidal zone
**Order 2014 (Modern Taxonomy)**	not determined	**Ecophysiological traits**	EPS production
**Taxonomy Notes**		**Notes on ecophysiological traits**	capsules
**Accession Number(s)_16S**		**Isolator**	Vitor Ramos
**Accession Number(s)_others (product/gene /region)**		**Medium**	Z8 25‰ TM sea salt w/ vit. B12
**Collector**	Bárbara Frazão	**Strain Status**	non-axenic, unicyanobacterial
**Collection Date**	2010/04/01	**Preservation type**	subculturing
**Location**	Portugal: Selvagens Islands	**Light:dark cycle**	14:10-h
**Latitude & Longitude**	30.03458 N 16.03267 W	**Temperature (ºC)**	25
		**Light intensity**	10–30 µmol photons m−2 s−1
**References Publicly Available Articles**			
**References Publicly Available Theses**			

**Strain ID**	**Gloeocapsopsis crepidinum LEGE 06123**		
**Strain Taxonomy**	Gloeocapsopsis crepidinum (Thuret) Geitler, L ex Komárek, J (1993)		
**Microphotograph 400×**	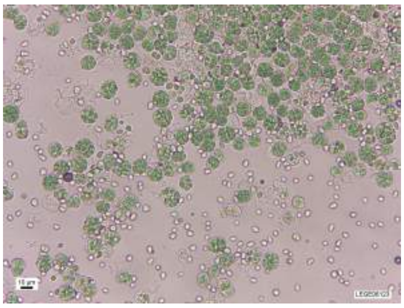	**Microphotograph 1000×**	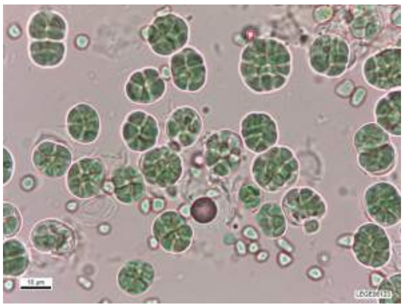
**Coidentity**	CCAP 1425/1	**Morphometrics (Width × Length or Diameter) (µm)**	Ø 4,0±0,4 (Cells); Ø 11,2±0,5 (Group of 6-7 cells)
**Other Code(s)**	LEAN 026	**Taxonomic notes/diacritical features**	cells with irregular forms; daughter cells released by disruption of mother cell envelope; cell ultrastructure data available
**Parent Sample**	O.06 LUZ 3 - BAFA	**Macroscopic growth features**	color: cyano; forming aggregates
**Isolation Date**	2007/06/12	**Environment**	aquatic, marine
**Order (Older Classification Scheme)**	Chroococcales	**Habitat Sample Description**	intertidal zone, on green algae
**Order 2014 (Modern Taxonomy)**	Chroococcidiopsidales**	**Ecophysiological traits**	
**Taxonomy Notes**	**inconsistent; assignment supported by phylogeny	**Notes on ecophysiological traits**	
**Accession Number(s)_16S**	FJ589716	**Isolator**	Vitor Ramos
**Accession Number(s)_others (product/gene /region)**	GU597364 (nifH), FJ536846 (nifK), FJ536848 (cpcA-IGS-cpcB), FJ589716 (16S-ITS-23S rRNA), KC813188, KC813189 (non-ribosomal peptide synthetase)	**Medium**	MN w/ vit. B12
**Collector**	Rui Seabra	**Strain Status**	non-axenic, unicyanobacterial
**Collection Date**	2006/12/12	**Preservation type**	subculturing
**Location**	Portugal: Praia da Luz, Lagos	**Light:dark cycle**	12:12-h
**Latitude & Longitude**	37.08622 N 8.725375 W	**Temperature (ºC)**	19
		**Light intensity**	10–30 µmol photons m−2 s−1
**References Publicly Available Articles**	Ramos et al. (2010) Eur. J. Phycol. 45, 394-403; Brito et al. (2012) Syst. Appl. Microbiol. 35, 110–119; Rastoll et al. (2013) J. Appl. Phycol. 25, 1483-1493; Brito et al. (2015) Algal Res. 9, 218-226		
**References Publicly Available Theses**	Macieira R. (2013) MSc Dissertation, FCUP, University of Porto, Porto, Portugal		

Strain ID	**Gloeocapsopsis sp. LEGE 13414**		
**Strain Taxonomy**	Gloeocapsopsis Geitler, L ex Komárek, J (1993)		
**Microphotograph 400×**	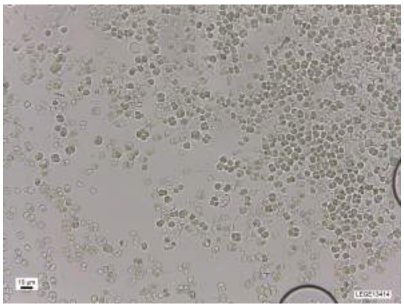	**Microphotograph 1000×**	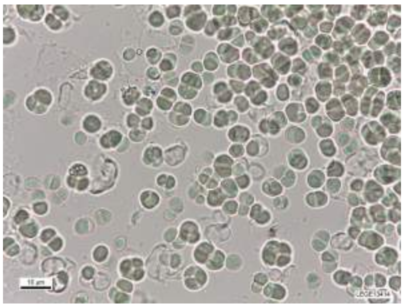
**Coidentity**	n.a.	**Morphometrics (Width × Length or Diameter) (µm)**	Ø 4,2± 0,6 (Cells) ; Ø 6,3±0,6 (Group of 4 cells)
**Other Code(s)**		**Taxonomic notes/diacritical features**	small colonies; with sheath
**Parent Sample**	S6C sal	**Macroscopic growth features**	color: greenish; forming aggregates
**Isolation Date**		**Environment**	unknown
**Order (Older Classification Scheme)**	Chroococcales	**Habitat Sample Description**	"Spirulina" dietary supplements, capsules form. Producer/brand: Solaray
**Order 2014 (Modern Taxonomy)**	not determined	**Ecophysiological traits**	
**Taxonomy Notes**		**Notes on ecophysiological traits**	
**Accession Number(s)_16S**		**Isolator**	Dina Gomes
**Accession Number(s)_others (product/gene /region)**		**Medium**	Z8 25‰ TM sea salt w/ vit. B12
**Collector**	Dina Gomes	**Strain Status**	non-axenic, unicyanobacterial
**Collection Date**	2013	**Preservation type**	subculturing
**Location**	original source unknown: acquired in the Porto metropolitan area, Portugal	**Light:dark cycle**	12:12-h
**Latitude & Longitude**	unknown	**Temperature (ºC)**	19
		**Light intensity**	10–30 µmol photons m−2 s−1
**References Publicly Available Articles**			
**References Publicly Available Theses**	Gomes D. (2013) MSc Dissertation, ICBAS, University of Porto, Porto, Portugal		

**Strain ID**	**Gloeocapsopsis sp. LEGE 13420**		
**Strain Taxonomy**	Gloeocapsopsis Geitler, L ex Komárek, J (1993)		
**Microphotograph 400×**	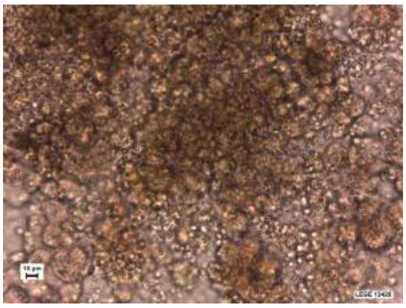	**Microphotograph 1000×**	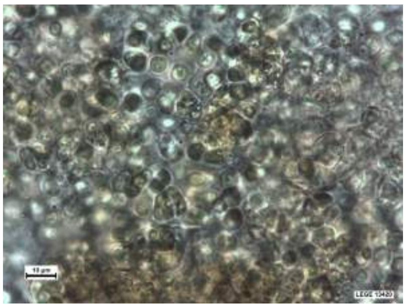
**Coidentity**	n.a.	**Morphometrics (Width × Length or Diameter) (µm)**	Ø 2,2±0,4 (more or less spherical cells)
**Other Code(s)**	S4C	**Taxonomic notes/diacritical features**	with sheath
**Parent Sample**		**Macroscopic growth features**	color: cyano; forming aggregates
**Isolation Date**		**Environment**	unknown
**Order (Older Classification Scheme)**	Chroococcales	**Habitat Sample Description**	"Spirulina" dietary supplements, tablet form. Producer/brand: Marcus Rohrer
**Order 2014 (Modern Taxonomy)**	Chroococcidiopsidales**	**Ecophysiological traits**	
**Taxonomy Notes**	**inconsistent; assignment supported by phylogeny	**Notes on ecophysiological traits**	
**Accession Number(s)_16S**	KU951729	**Isolator**	Dina Gomes
**Accession Number(s)_others (product/gene /region)**		**Medium**	Z8
**Collector**	Dina Gomes	**Strain Status**	non-axenic, unicyanobacterial, delicate
**Collection Date**	2013	**Preservation type**	subculturing
**Location**	original source unknown: acquired in the Porto metropolitan area, Portugal	**Light:dark cycle**	12:12-h
**Latitude & Longitude**	unknown	**Temperature (ºC)**	19
		**Light intensity**	10–30 µmol photons m−2 s−1
**References Publicly Available Articles**			
**References Publicly Available Theses**	Gomes D. (2013) MSc Dissertation, ICBAS, University of Porto, Porto, Portugal		

Strain ID	**Gloeocapsopsis sp. LEGE 13421**		
**Strain Taxonomy**	Gloeocapsopsis Geitler, L ex Komárek, J (1993)		
**Microphotograph 400×**	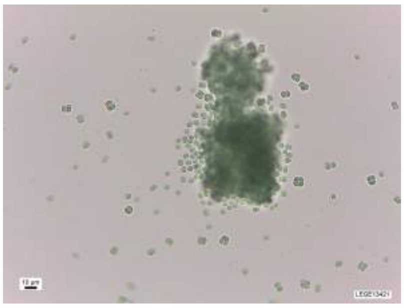	**Microphotograph 1000×**	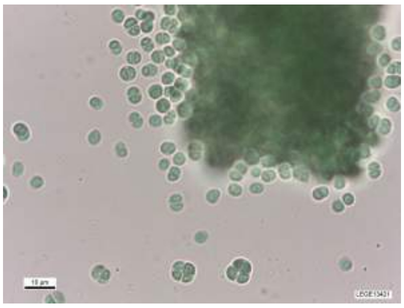
**Coidentity**	n.a.	**Morphometrics (Width × Length or Diameter) (µm)**	Ø 2,6±0,4 (more or less spherical cells)
**Other Code(s)**	S5C	**Taxonomic notes/diacritical features**	with sheath
**Parent Sample**		**Macroscopic growth features**	color: cyano; forming aggregates
**Isolation Date**		**Environment**	unknown
**Order (Older Classification Scheme)**	Chroococcales	**Habitat Sample Description**	"Spirulina" dietary supplements, tablet form. Producer/brand: Biover
**Order 2014 (Modern Taxonomy)**	Chroococcidiopsidales**	**Ecophysiological traits**	
**Taxonomy Notes**	**inconsistent; assignment supported by phylogeny	**Notes on ecophysiological traits**	
**Accession Number(s)_16S**	KU951730	**Isolator**	Dina Gomes
**Accession Number(s)_others (product/gene /region)**		**Medium**	Z8
**Collector**	Dina Gomes	**Strain Status**	non-axenic, unicyanobacterial
**Collection Date**	2013	**Preservation type**	subculturing
**Location**	original source unknown: acquired in the Porto metropolitan area, Portugal	**Light:dark cycle**	12:12-h
**Latitude & Longitude**	unknown	**Temperature (ºC)**	19
		**Light intensity**	10–30 µmol photons m−2 s−1
**References Publicly Available Articles**			
**References Publicly Available Theses**	Gomes D. (2013) MSc Dissertation, ICBAS, University of Porto, Porto, Portugal		

**Strain ID**	**Halomicronema cf. metazoicum LEGE 07132**		
**Strain Taxonomy**	Halomicronema metazoicum Caroppo, C; Pagliara, P & Albertano, P in Caroppo et al (2012)		
**Microphotograph 400×**	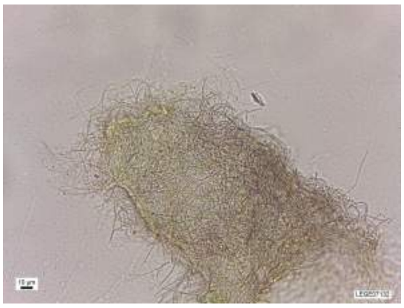	**Microphotograph 1000×**	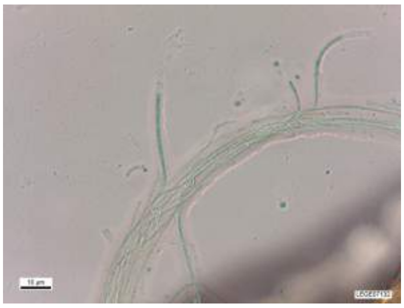
**Coidentity**	n.a.	**Morphometrics (Width × Length or Diameter) (µm)**	1,3±0,3 × 1,1±0,4
**Other Code(s)**	LEAN 041	**Taxonomic notes/diacritical features**	
**Parent Sample**	P.07 LUZ 3 - BA	**Macroscopic growth features**	color: greenish to brownish; forming a firm biofilm
**Isolation Date**		**Environment**	aquatic, marine
**Order (Older Classification Scheme)**	Oscillatoriales	**Habitat Sample Description**	intertidal zone, wave-exposed rock, surface scraping
**Order 2014 (Modern Taxonomy)**	not determined	**Ecophysiological traits**	
**Taxonomy Notes**		**Notes on ecophysiological traits**	chromatic adaptation?
**Accession Number(s)_16S**	HQ832944	**Isolator**	Vitor Ramos
**Accession Number(s)_others (product/gene /region)**	KC256767 (dinitrogenase reductase, nifH), KC842359-KC842361 (polyketide synthase)	**Medium**	MN w/ vit. B12
**Collector**	Rui Seabra/Vitor Ramos	**Strain Status**	non-axenic, unicyanobacterial
**Collection Date**	2007/05/02	**Preservation type**	subculturing
**Location**	Portugal: Praia da Luz, Lagos	**Light:dark cycle**	12:12-h
**Latitude & Longitude**	37.08622 N 8.725375 W	**Temperature (ºC)**	19
		**Light intensity**	10–30 µmol photons m−2 s−1
**References Publicly Available Articles**	Brito et al. (2012) Syst. Appl. Microbiol. 35, 110–119; Leão et al. (2013) Mar. Drugs 11 (4), 1316-1335; Brito et al. (2015) Algal Res. 9, 218-226		
**References Publicly Available Theses**			

Strain ID	**Hyella patelloides LEGE 07179**		
**Strain Taxonomy**	Hyella patelloides Ramos, V; Brito, A & Kaštovský, J in Brito et al (2017)		
**Microphotograph 400×**	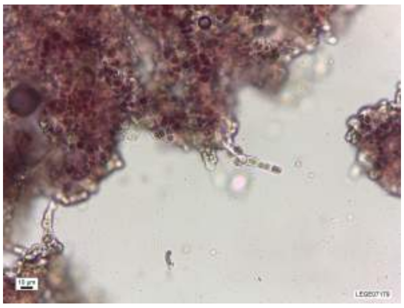	**Microphotograph 1000×**	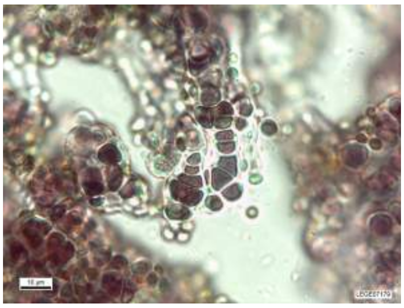
**Coidentity**	n.a.	**Morphometrics (Width × Length or Diameter) (µm)**	5,8±1,2 × 3,0±1,2 (Cells); Sheath up to 1,2±0,4
**Other Code(s)**	LEAN 093	**Taxonomic notes/diacritical features**	uniseriate pseudofilaments; with sheath;
**Parent Sample**	P.07 MOL 4 - C	**Macroscopic growth features**	color: dark brown; forming aggregates/colonies
**Isolation Date**	2007	**Environment**	aquatic, marine
**Order (Older Classification Scheme)**	Chroococcales	**Habitat Sample Description**	intertidal zone, on a Patella sp. shell, euendolithic
**Order 2014 (Modern Taxonomy)**	Pleurocapsales	**Ecophysiological traits**	
**Taxonomy Notes**	This is the Type strain of Hyella patelloides Ramos, Brito et Kaštovský sp. nov.	**Notes on ecophysiological traits**	
**Accession Number(s)_16S**	HQ832901	**Isolator**	Vitor Ramos
**Accession Number(s)_others (product/gene /region)**	KC256774 (dinitrogenase reductase, nifH), KC813174 (non-ribosomal peptide synthetase), KC842291, KC842292 (polyketide synthase)	**Medium**	MN w/ vit. B12
**Collector**	Rui Seabra/Vitor Ramos	**Strain Status**	non-axenic, unicyanobacterial
**Collection Date**	2007/05/21	**Preservation type**	subculturing, cryopreserved
**Location**	Portugal: Praia de Moledo, Caminha	**Light:dark cycle**	12:12-h
**Latitude & Longitude**	41.84963 N 8.866717 W	**Temperature (ºC)**	19
		**Light intensity**	10–30 µmol photons m−2 s−1
**References Publicly Available Articles**	Brito et al. (2012) Syst. Appl. Microbiol. 35, 110–119; Rastoll et al. (2013) J. Appl. Phycol. 25, 1483-1493; Brito et al. (2015) Algal Res. 9, 218-226; Brito et al. (2017) Mol. Phylog. Evol. doi:10.1016/j.ympev.2017.03.006		
**References Publicly Available Theses**			

**Strain ID**	**Jaaginema litorale LEGE 07176**		
**Strain Taxonomy**	Jaaginema litorale Ramos, V; Brito, A & Kaštovský, J in Brito et al (2017)		
**Microphotograph 400×**	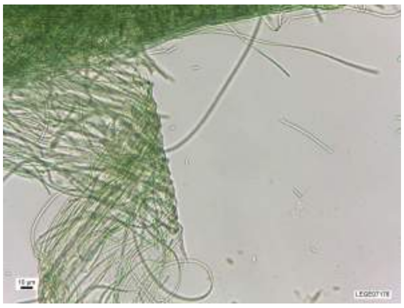	**Microphotograph 1000×**	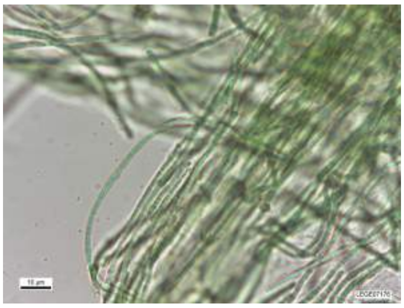
**Coidentity**	n.a.	**Morphometrics (Width × Length or Diameter) (µm)**	1,5±0,2 × 2,7±0,6
**Other Code(s)**	LEAN 090	**Taxonomic notes/diacritical features**	
**Parent Sample**	P.07 AGU 5 - BAA	**Macroscopic growth features**	color: cyano; forming a firm biofilm
**Isolation Date**		**Environment**	aquatic, marine
**Order (Older Classification Scheme)**	Oscillatoriales	**Habitat Sample Description**	intertidal zone, wave-exposed rock
**Order 2014 (Modern Taxonomy)**	undefined	**Ecophysiological traits**	
**Taxonomy Notes**	This is the Type strain of Jaaginema litorale Ramos, Brito et Kaštovský sp. nov.	**Notes on ecophysiological traits**	
**Accession Number(s)_16S**	HQ832925	**Isolator**	Vitor Ramos
**Accession Number(s)_others (product/gene /region)**	KC842330 (polyketide synthase)	**Medium**	MN w/ vit. B12
**Collector**	Rui Seabra/Vitor Ramos	**Strain Status**	non-axenic, unicyanobacterial
**Collection Date**	2007/05/15	**Preservation type**	subculturing
**Location**	Portugal: Praia da Aguda, Arcozelo	**Light:dark cycle**	12:12-h
**Latitude & Longitude**	41.04954 N 8.655339 W	**Temperature (ºC)**	19
		**Light intensity**	10–30 µmol photons m−2 s−1
**References Publicly Available Articles**	Brito et al. (2012) Syst. Appl. Microbiol. 35, 110–119; Brito et al. (2015) Algal Res. 9, 218-226; Brito et al. (2017) Mol. Phylog. Evol. doi:10.1016/j.ympev.2017.03.006		
**References Publicly Available Theses**			

Strain ID	**Leptolyngbya cf. ectocarpi LEGE 11389**		
**Strain Taxonomy**	Leptolyngbya ectocarpi (Gomont) Anagnostidis, K & Komárek, J (1988)		
**Microphotograph 400×**	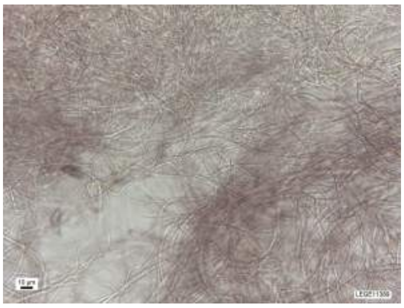	**Microphotograph 1000×**	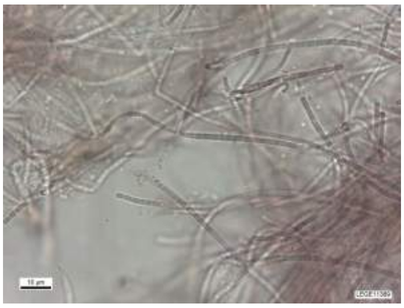
**Coidentity**	n.a.	**Morphometrics (Width × Length or Diameter) (µm)**	1,5±0,3 × 2,1±0,4
**Other Code(s)**		**Taxonomic notes/diacritical features**	with sheath
**Parent Sample**	EB1-3-EAAA	**Macroscopic growth features**	color: brownish; forming a firm biofilm
**Isolation Date**		**Environment**	aquatic, hypersaline
**Order (Older Classification Scheme)**	Oscillatoriales	**Habitat Sample Description**	upper layer of a microbial mat
**Order 2014 (Modern Taxonomy)**	Synechococcales	**Ecophysiological traits**	
**Taxonomy Notes**		**Notes on ecophysiological traits**	
**Accession Number(s)_16S**	KU951731	**Isolator**	Vitor Ramos
**Accession Number(s)_others (product/gene /region)**		**Medium**	MN w/ vit. B12
**Collector**	Frederico Sobrinho/Sinda Carvalhal	**Strain Status**	non-axenic, unicyanobacterial
**Collection Date**	9-Feb-2011	**Preservation type**	subculturing
**Location**	Brazil: Araruama (main) lagoon, in the state of Rio de Janeiro	**Light:dark cycle**	12:12-h
**Latitude & Longitude**	22.94333 S 42.10056 W	**Temperature (ºC)**	19
		**Light intensity**	10–30 µmol photons m−2 s−1
**References Publicly Available Articles**			
**References Publicly Available Theses**			

Strain ID	**Leptolyngbya cf. ectocarpi LEGE 11479**		
**Strain Taxonomy**	Leptolyngbya ectocarpi (Gomont) Anagnostidis, K & Komárek, J (1988)		
**Microphotograph 400×**	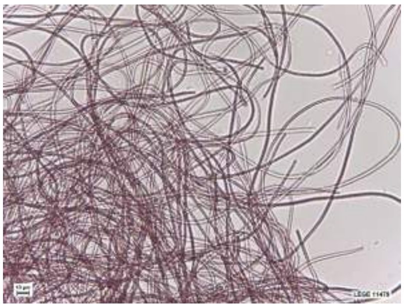	**Microphotograph 1000×**	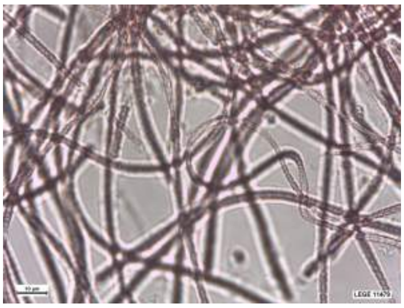
**Coidentity**	n.a.	**Morphometrics (Width × Length or Diameter) (µm)**	1,8±0,3 × 1,6±0,4
**Other Code(s)**		**Taxonomic notes/diacritical features**	with sheath
**Parent Sample**	1.009.0911	**Macroscopic growth features**	color: brownish; forming a firm biofilm
**Isolation Date**		**Environment**	aquatic, marine
**Order (Older Classification Scheme)**	Oscillatoriales	**Habitat Sample Description**	subtidal sample, epilithic (10-13m depth), less than 1 km off the shore
**Order 2014 (Modern Taxonomy)**	Synechococcales	**Ecophysiological traits**	
**Taxonomy Notes**		**Notes on ecophysiological traits**	
**Accession Number(s)_16S**	KU951732	**Isolator**	Ângela Pinheiro
**Accession Number(s)_others (product/gene /region)**		**Medium**	Z8 25‰ TM sea salt w/ vit. B12
**Collector**	Pedro Leão/Aldo Barreiro	**Strain Status**	non-axenic, unicyanobacterial
**Collection Date**	2011/09/26	**Preservation type**	subculturing
**Location**	Portugal: 'Pêlo Negro', diving spot near Leixões Harbour	**Light:dark cycle**	12:12-h
**Latitude & Longitude**	41.18557 N 8.719100 W	**Temperature (ºC)**	19
		**Light intensity**	10–30 µmol photons m−2 s−1
**References Publicly Available Articles**			
**References Publicly Available Theses**			

**Strain ID**	**Leptolyngbya ectocarpi LEGE 11425**		
**Strain Taxonomy**	Leptolyngbya ectocarpi (Gomont) Anagnostidis, K & Komárek, J (1988)		
**Microphotograph 400×**	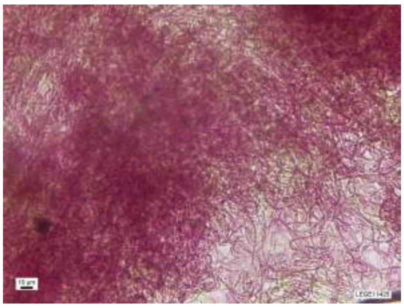	**Microphotograph 1000×**	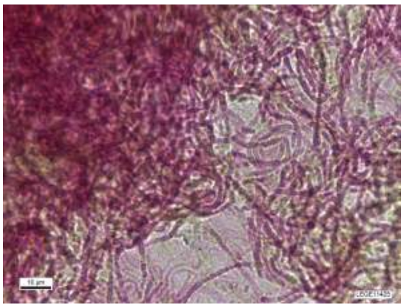
**Coidentity**	n.a.	**Morphometrics (Width × Length or Diameter) (µm)**	1,5±0,1 × 2,4±0,4
**Other Code(s)**		**Taxonomic notes/diacritical features**	
**Parent Sample**	SC-Gi-A, 8.002.0911 MN + P	**Macroscopic growth features**	color: reddish; forming a firm biofilm
**Isolation Date**		**Environment**	aquatic, marine
**Order (Older Classification Scheme)**	Oscillatoriales	**Habitat Sample Description**	subtidal sample, epilithic (10-13m depth), less than 1 km off the shore
**Order 2014 (Modern Taxonomy)**	Synechococcales	**Ecophysiological traits**	
**Taxonomy Notes**		**Notes on ecophysiological traits**	dehydroabietic acid production (unknown ecophysiological role)
**Accession Number(s)_16S**	New Seq + KT951671	**Isolator**	Sofia Costa
**Accession Number(s)_others (product/gene /region)**		**Medium**	Z8 25‰ TM sea salt w/ vit. B12
**Collector**	Pedro Leão/Aldo Barreiro	**Strain Status**	non-axenic, unicyanobacterial
**Collection Date**	2011/09/26	**Preservation type**	subculturing
**Location**	Portugal: 'Pêlo Negro', diving spot near Leixões Harbour	**Light:dark cycle**	12:12-h
**Latitude & Longitude**	41.18557 N 8.719100 W	**Temperature (ºC)**	19
		**Light intensity**	5 µmol photons m−2 s−1
**References Publicly Available Articles**	Costa et al. (2016) Sci. Rep. 6, 23436		
**References Publicly Available Theses**			

**Strain ID**	**Leptolyngbya ectocarpi LEGE 11474**		
**Strain Taxonomy**	Leptolyngbya ectocarpi (Gomont) Anagnostidis, K & Komárek, J (1988)		
**Microphotograph 400×**	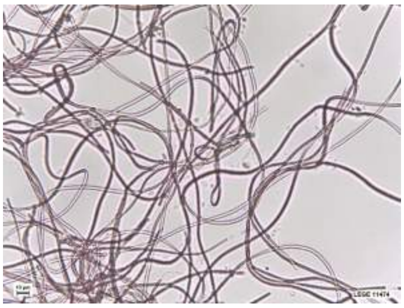	**Microphotograph 1000×**	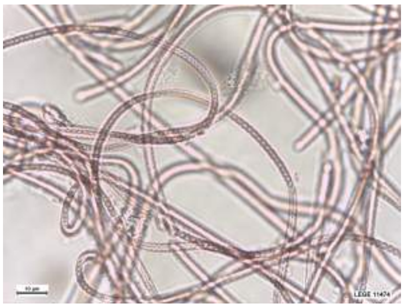
**Coidentity**	n.a.	**Morphometrics (Width × Length or Diameter) (µm)**	1,9±0,3 × 1,8±0,4
**Other Code(s)**		**Taxonomic notes/diacritical features**	with sheath
**Parent Sample**	1.009.1011	**Macroscopic growth features**	color: brownish; forming a smooth biofilm and aggregates
**Isolation Date**		**Environment**	aquatic, marine
**Order (Older Classification Scheme)**	Oscillatoriales	**Habitat Sample Description**	subtidal sample, epilithic (13m depth), about 200 m off the shore
**Order 2014 (Modern Taxonomy)**	Synechococcales	**Ecophysiological traits**	
**Taxonomy Notes**		**Notes on ecophysiological traits**	
**Accession Number(s)_16S**	KU951733	**Isolator**	Ângela Pinheiro
**Accession Number(s)_others (product/gene /region)**		**Medium**	Z8 25‰ TM sea salt w/ vit. B12
**Collector**	Pedro Leão/Aldo Barreiro	**Strain Status**	non-axenic, unicyanobacterial
**Collection Date**	2011/10/03	**Preservation type**	subculturing
**Location**	Portugal: 'A Pedra', diving spot in front of the fort 'Castelo do Queijo'	**Light:dark cycle**	12:12-h
**Latitude & Longitude**	41.185809 N 8.719079 W	**Temperature (ºC)**	19
		**Light intensity**	10–30 µmol photons m−2 s−1
**References Publicly Available Articles**			
**References Publicly Available Theses**			

**Strain ID**	**Leptolyngbya minuta LEGE 07128**		
**Strain Taxonomy**	Leptolyngbya minuta (Lindstedt) Anagnostidis, K & Komárek, J (1988)		
**Microphotograph 400×**	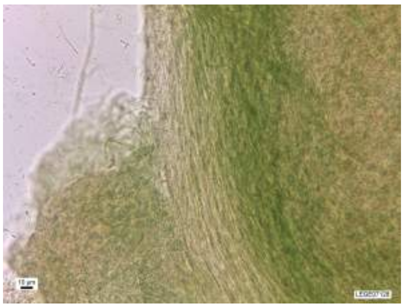	**Microphotograph 1000×**	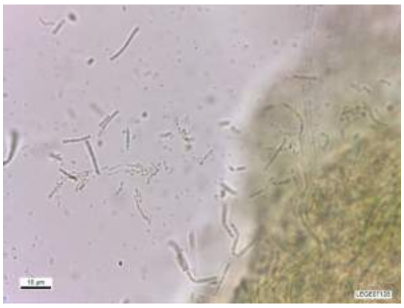
**Coidentity**	n.a.	**Morphometrics (Width × Length or Diameter) (µm)**	1,1±0,2 × 1,6±0,5
**Other Code(s)**	LEAN 035	**Taxonomic notes/diacritical features**	
**Parent Sample**	P.07 ODA 5 - BE	**Macroscopic growth features**	color: cyano; forming aggregates
**Isolation Date**		**Environment**	aquatic, marine
**Order (Older Classification Scheme)**	Oscillatoriales	**Habitat Sample Description**	tide puddle, rock surface scraping
**Order 2014 (Modern Taxonomy)**	not determined	**Ecophysiological traits**	
**Taxonomy Notes**		**Notes on ecophysiological traits**	
**Accession Number(s)_16S**		**Isolator**	Vitor Ramos
**Accession Number(s)_others (product/gene /region)**		**Medium**	MN w/ vit. B12
**Collector**	Rui Seabra/Vitor Ramos	**Strain Status**	non-axenic, unicyanobacterial
**Collection Date**	2007/05/02	**Preservation type**	subculturing
**Location**	Portugal: Praia de Olhos d'Água, Albufeira	**Light:dark cycle**	12:12-h
**Latitude & Longitude**	37.08973 N 8.191017 W	**Temperature (ºC)**	19
		**Light intensity**	10–30 µmol photons m−2 s−1
**References Publicly Available Articles**			
**References Publicly Available Theses**			

**Strain ID**	**Leptolyngbya saxicola LEGE 06131**		
**Strain Taxonomy**	Leptolyngbya saxicola (Gardner) Anagnostidis,K (2001)		
**Microphotograph 400×**	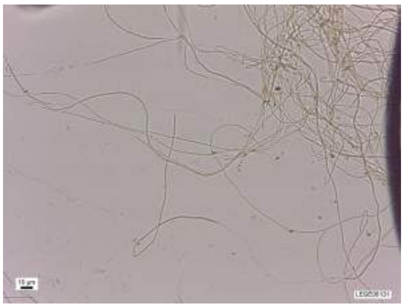	**Microphotograph 1000×**	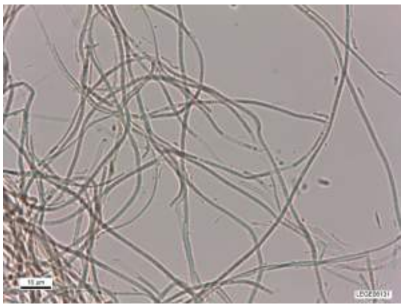
**Coidentity**	n.a.	**Morphometrics (Width × Length or Diameter) (µm)**	1,4±0,2 × 3,7±1,0
**Other Code(s)**	LEAN 040	**Taxonomic notes/diacritical features**	with sheath
**Parent Sample**	O.06 BUR 6 - EA	**Macroscopic growth features**	color: greenish to brownish; forming a firm biofilm
**Isolation Date**		**Environment**	aquatic, marine
**Order (Older Classification Scheme)**	Oscillatoriales	**Habitat Sample Description**	intertidal zone, wave-exposed rock, surface scraping
**Order 2014 (Modern Taxonomy)**	not determined	**Ecophysiological traits**	
**Taxonomy Notes**		**Notes on ecophysiological traits**	chromatic adaptation?
**Accession Number(s)_16S**		**Isolator**	Vitor Ramos
**Accession Number(s)_others (product/gene /region)**		**Medium**	MN w/ vit. B12
**Collector**	Rui Seabra	**Strain Status**	non-axenic, unicyanobacterial
**Collection Date**	2006/12/11	**Preservation type**	subculturing
**Location**	Portugal: Praia de Burgau, Budens	**Light:dark cycle**	12:12-h
**Latitude & Longitude**	37.07156 N 8.774722 W	**Temperature (ºC)**	19
		**Light intensity**	10–30 µmol photons m−2 s−1
**References Publicly Available Articles**			
**References Publicly Available Theses**			

Strain ID	**Leptolyngbya sp. LEGE 06117**		
**Strain Taxonomy**	Leptolyngbya Anagnostidis, K & Komárek, J (1988)		
**Microphotograph 400×**	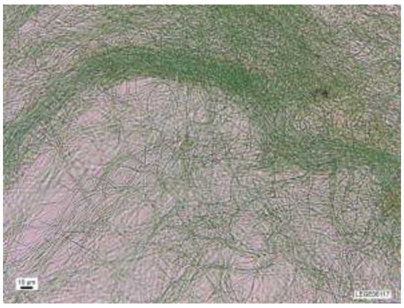	**Microphotograph 1000×**	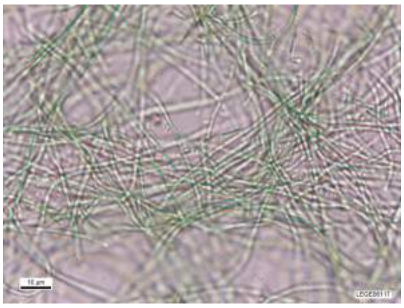
**Coidentity**	n.a.	**Morphometrics (Width × Length or Diameter) (µm)**	1,0±0,1 × 2,7±0,5
**Other Code(s)**	LEAN 022	**Taxonomic notes/diacritical features**	
**Parent Sample**	O.06 MAR 5 - A (II)	**Macroscopic growth features**	color: cyano; forming a firm biofilm
**Isolation Date**		**Environment**	aquatic, marine
**Order (Older Classification Scheme)**	Oscillatoriales	**Habitat Sample Description**	wave-exposed tide pool, rock surface scraping
**Order 2014 (Modern Taxonomy)**	not determined	**Ecophysiological traits**	
**Taxonomy Notes**		**Notes on ecophysiological traits**	
**Accession Number(s)_16S**		**Isolator**	Vitor Ramos
**Accession Number(s)_others (product/gene /region)**		**Medium**	MN w/ vit. B12
**Collector**	Rui Seabra	**Strain Status**	non-axenic, unicyanobacterial
**Collection Date**	2006/12/11	**Preservation type**	subculturing
**Location**	Portugal: Praia do Martinhal, Vila do Bispo	**Light:dark cycle**	12:12-h
**Latitude & Longitude**	37.01869 N 8.926714 W	**Temperature (ºC)**	19
		**Light intensity**	10–30 µmol photons m−2 s−1
**References Publicly Available Articles**			
**References Publicly Available Theses**			

**Strain ID**	**Leptolyngbya sp. LEGE 06361**		
**Strain Taxonomy**	Leptolyngbya Anagnostidis, K & Komárek, J (1988)		
**Microphotograph 400×**	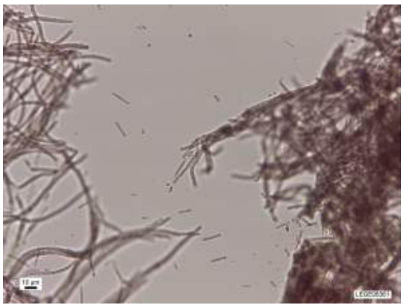	**Microphotograph 1000×**	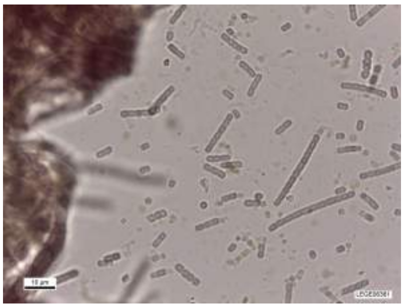
**Coidentity**	n.a.	**Morphometrics (Width × Length or Diameter) (µm)**	1,8±0,2 × 2,5±0,5
**Other Code(s)**		**Taxonomic notes/diacritical features**	trichomes slightly constricted at cross-walls; facultative sheath
**Parent Sample**		**Macroscopic growth features**	color: brownish; forming a firm biofilm
**Isolation Date**		**Environment**	aquatic, freshwater
**Order (Older Classification Scheme)**	Oscillatoriales	**Habitat Sample Description**	biofilm, from a secondary decanter tank bank
**Order 2014 (Modern Taxonomy)**	not determined	**Ecophysiological traits**	
**Taxonomy Notes**		**Notes on ecophysiological traits**	
**Accession Number(s)_16S**		**Isolator**	Joana Martins
**Accession Number(s)_others (product/gene /region)**		**Medium**	Z8
**Collector**	Joana Martins	**Strain Status**	non-axenic, unicyanobacterial
**Collection Date**	Oct-2006	**Preservation type**	subculturing
**Location**	Portugal: wastewater treatment plant at Febros river, Vila Nova de Gaia	**Light:dark cycle**	12:12-h
**Latitude & Longitude**	41.11812 N 8.570944 W	**Temperature (ºC)**	19
		**Light intensity**	10–30 µmol photons m−2 s−1
**References Publicly Available Articles**			
**References Publicly Available Theses**			

Strain ID	**Leptolyngbya sp. LEGE 07154**		
**Strain Taxonomy**	Leptolyngbya Anagnostidis, K & Komárek, J (1988)		
**Microphotograph 400×**	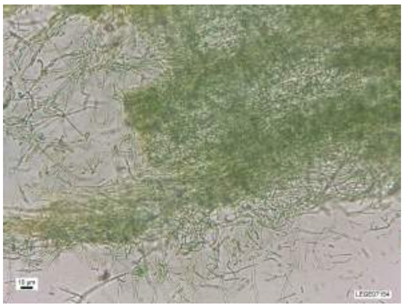	**Microphotograph 1000×**	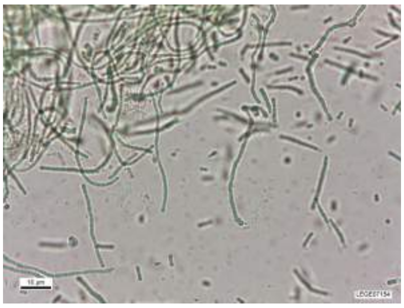
**Coidentity**	n.a.	**Morphometrics (Width × Length or Diameter) (µm)**	1,1±0,2 × 2,1±0,3
**Other Code(s)**	LEAN 068	**Taxonomic notes/diacritical features**	
**Parent Sample**	P.07 MAR 2 - AB	**Macroscopic growth features**	color: cyano; forming a firm biofilm
**Isolation Date**		**Environment**	aquatic, marine
**Order (Older Classification Scheme)**	Oscillatoriales	**Habitat Sample Description**	tide pool, emersed rock, surface scraping
**Order 2014 (Modern Taxonomy)**	not determined	**Ecophysiological traits**	
**Taxonomy Notes**		**Notes on ecophysiological traits**	
**Accession Number(s)_16S**		**Isolator**	Vitor Ramos
**Accession Number(s)_others (product/gene /region)**		**Medium**	MN w/ vit. B12
**Collector**	Rui Seabra/Vitor Ramos	**Strain Status**	non-axenic, unicyanobacterial
**Collection Date**	2007/05/02	**Preservation type**	subculturing
**Location**	Portugal: Praia do Martinhal, Vila do Bispo	**Light:dark cycle**	12:12-h
**Latitude & Longitude**	37.01869 N 8.926714 W	**Temperature (ºC)**	19
		**Light intensity**	10–30 µmol photons m−2 s−1
**References Publicly Available Articles**			
**References Publicly Available Theses**			

**Strain ID**	**Leptolyngbya sp. LEGE 13418**		
**Strain Taxonomy**	Leptolyngbya Anagnostidis, K & Komárek, J (1988)		
**Microphotograph 400×**	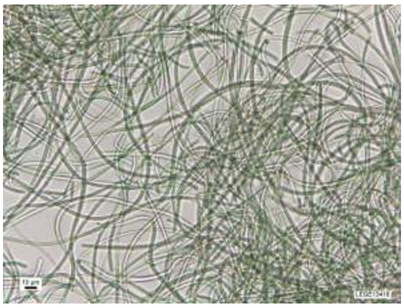	**Microphotograph 1000×**	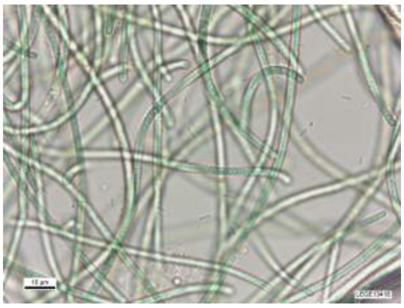
**Coidentity**	n.a.	**Morphometrics (Width × Length or Diameter) (µm)**	2,4±0,2 × 1,4±0,3
**Other Code(s)**	S1F	**Taxonomic notes/diacritical features**	
**Parent Sample**		**Macroscopic growth features**	color: cyano; forming aggregates
**Isolation Date**		**Environment**	unknown
**Order (Older Classification Scheme)**	Oscillatoriales	**Habitat Sample Description**	"Spirulina" dietary supplements, powder form. Producer/brand: Iswari
**Order 2014 (Modern Taxonomy)**	Synechococcales	**Ecophysiological traits**	
**Taxonomy Notes**		**Notes on ecophysiological traits**	
**Accession Number(s)_16S**	KU951734	**Isolator**	Dina Gomes
**Accession Number(s)_others (product/gene /region)**		**Medium**	Z8
**Collector**	Dina Gomes	**Strain Status**	non-axenic, unicyanobacterial
**Collection Date**	2013	**Preservation type**	subculturing
**Location**	original source unknown: acquired in the Porto metropolitan area, Portugal	**Light:dark cycle**	12:12-h
**Latitude & Longitude**	unknown	**Temperature (ºC)**	19
		**Light intensity**	10–30 µmol photons m−2 s−1
**References Publicly Available Articles**			
**References Publicly Available Theses**	Gomes D. (2013) MSc Dissertation, ICBAS, University of Porto, Porto, Portugal		

**Strain ID**	**Leptolyngbya-like sp. LEGE 13412**		
**Strain Taxonomy**	Leptolyngbya Anagnostidis, K & Komárek, J (1988)		
**Microphotograph 400×**	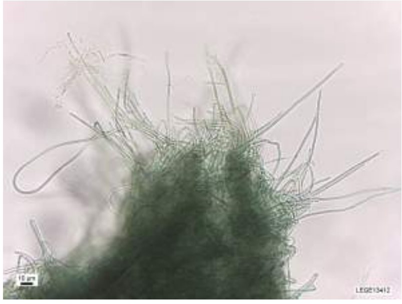	**Microphotograph 1000×**	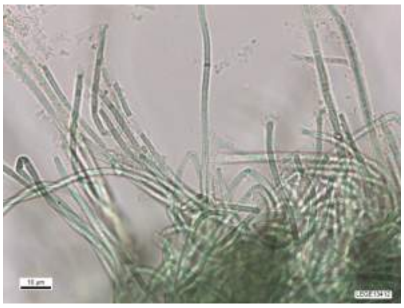
**Coidentity**	n.a.	**Morphometrics (Width × Length or Diameter) (µm)**	1,7±0,3 × 2,6±0,6
**Other Code(s)**		**Taxonomic notes/diacritical features**	hardly distinguished cell walls; distinguishable centro- and chromatoplasm; with sheath
**Parent Sample**	S1F sal	**Macroscopic growth features**	color: cyano; forming tuft-like aggregates; mucilaginous
**Isolation Date**		**Environment**	unknown
**Order (Older Classification Scheme)**	Oscillatoriales	**Habitat Sample Description**	"Spirulina" dietary supplements, powder form. Producer/brand: Iswari
**Order 2014 (Modern Taxonomy)**	not determined	**Ecophysiological traits**	EPS production
**Taxonomy Notes**		**Notes on ecophysiological traits**	
**Accession Number(s)_16S**		**Isolator**	Dina Gomes
**Accession Number(s)_others (product/gene /region)**		**Medium**	Z8 25‰ TM sea salt w/ vit. B12
**Collector**	Dina Gomes	**Strain Status**	non-axenic, unicyanobacterial
**Collection Date**	2013	**Preservation type**	subculturing
**Location**	original source unknown: acquired in the Porto metropolitan area, Portugal	**Light:dark cycle**	12:12-h
**Latitude & Longitude**	unknown	**Temperature (ºC)**	19
		**Light intensity**	10–30 µmol photons m−2 s−1
**References Publicly Available Articles**			
**References Publicly Available Theses**	Gomes D. (2013) MSc Dissertation, ICBAS, University of Porto, Porto, Portugal		

Strain ID	**Limnoraphis robusta LEGE XX358**		
**Strain Taxonomy**	Limnoraphis robusta (Paracutty) Komárek, J; Zapomelová, E; Smarda, J; Kopecky, J; Rejmánková, E; Woodhouse, J; Neilan, BA & Komárková, J (2013)		
**Microphotograph 400×**	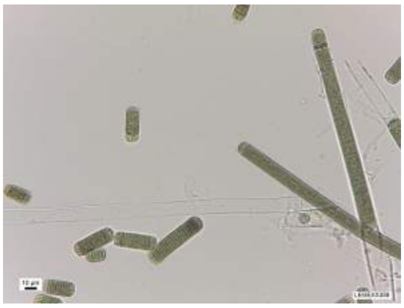	**Microphotograph 1000×**	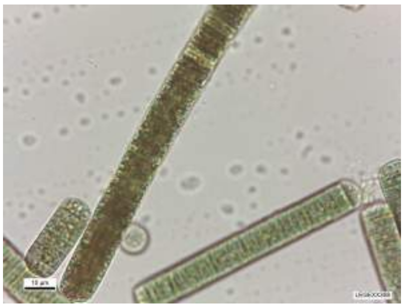
**Coidentity**	n.a.	**Morphometrics (Width × Length or Diameter) (µm)**	11,0±1,5 × 2,0±0,4
**Other Code(s)**		**Taxonomic notes/diacritical features**	straight trichomes, with necridic cells and hormogonia; with sheath
**Parent Sample**	Infout Osc III	**Macroscopic growth features**	color: olive green; forming a leather-like biofilm
**Isolation Date**		**Environment**	aquatic, freshwater
**Order (Older Classification Scheme)**	Oscillatoriales	**Habitat Sample Description**	unknown
**Order 2014 (Modern Taxonomy)**	Oscillatoriales	**Ecophysiological traits**	
**Taxonomy Notes**		**Notes on ecophysiological traits**	
**Accession Number(s)_16S**	KU951735	**Isolator**	Martin Saker
**Accession Number(s)_others (product/gene /region)**		**Medium**	Z8
**Collector**	Martin Saker	**Strain Status**	non-axenic, unicyanobacterial
**Collection Date**	unknown	**Preservation type**	subculturing
**Location**	unknown	**Light:dark cycle**	12:12-h
**Latitude & Longitude**	unknown	**Temperature (ºC)**	19
		**Light intensity**	10–30 µmol photons m−2 s−1
**References Publicly Available Articles**			
**References Publicly Available Theses**			

Strain ID	**Limnothrix sp. LEGE 00237**		
**Strain Taxonomy**	Limnothrix Meffert, E (1988)		
**Microphotograph 400×**	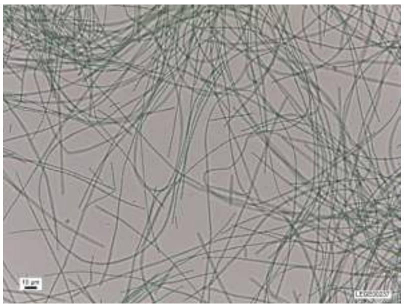	**Microphotograph 1000×**	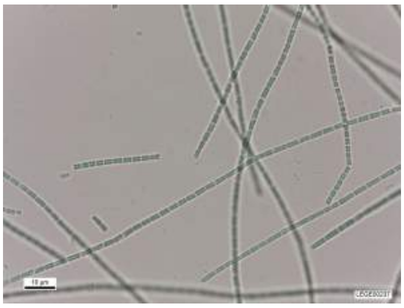
**Coidentity**	n.a.	**Morphometrics (Width × Length or Diameter) (µm)**	1,5±0,2 × 3,9±1,0
**Other Code(s)**	J7, LEANJ.7	**Taxonomic notes/diacritical features**	distinguishable centro- and chromatoplasm
**Parent Sample**		**Macroscopic growth features**	color: cyano; forming a smooth biofilm
**Isolation Date**		**Environment**	aquatic, freshwater
**Order (Older Classification Scheme)**	Oscillatoriales	**Habitat Sample Description**	water sample
**Order 2014 (Modern Taxonomy)**	Synechococcales	**Ecophysiological traits**	toxin production
**Taxonomy Notes**		**Notes on ecophysiological traits**	ANTX-a producing strain
**Accession Number(s)_16S**	KU951736	**Isolator**	Joana Osswald
**Accession Number(s)_others (product/gene /region)**		**Medium**	Z8
**Collector**	Joana Oswald	**Strain Status**	non-axenic, unicyanobacterial
**Collection Date**	2000/07/18	**Preservation type**	subculturing
**Location**	Portugal: Mortagua, water treatment station	**Light:dark cycle**	12:12-h
**Latitude & Longitude**	unknown	**Temperature (ºC)**	19
		**Light intensity**	10–30 µmol photons m−2 s−1
**References Publicly Available Articles**	Osswald et al, 2009. Ecotoxicology 18(8): 1110-1115		
**References Publicly Available Theses**	Osswald J. (2007) PhD Thesis, FCUP, University of Porto, Porto, Portugal		

Strain ID	**Lusitaniella coriacea LEGE 06111**		
**Strain Taxonomy**	Lusitaniella coriacea Ramos, V; Brito, A & Kaštovský, J in Brito et al (2017)		
**Microphotograph 400×**	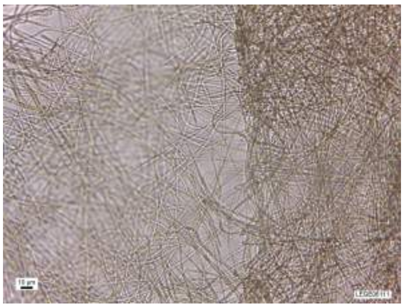	**Microphotograph 1000×**	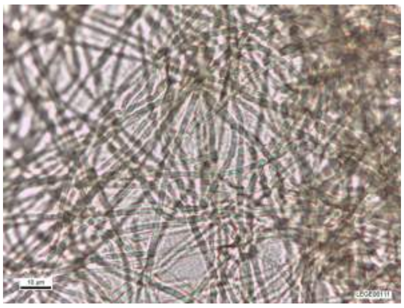
**Coidentity**	n.a.	**Morphometrics (Width × Length or Diameter) (µm)**	1,7±0,2 × 3,3±0,7 (Cells); Sheath up to 1,1±0,4
**Other Code(s)**	LEAN 016	**Taxonomic notes/diacritical features**	often, bright refractive granules (gas vesicles?) at cell ends;
**Parent Sample**	O.06 MAR 5 - E (I)	**Macroscopic growth features**	color: olive green; forming a leather-like biofilm
**Isolation Date**		**Environment**	aquatic, marine
**Order (Older Classification Scheme)**	Oscillatoriales	**Habitat Sample Description**	tidal pool, on emersed rock
**Order 2014 (Modern Taxonomy)**	undefined	**Ecophysiological traits**	
**Taxonomy Notes**	Paratype of Lusitaniella coriacea Ramos, Brito et Kaštovský gen. nov., sp. nov.	**Notes on ecophysiological traits**	
**Accession Number(s)_16S**	HQ832929	**Isolator**	Vitor Ramos
**Accession Number(s)_others (product/gene /region)**	KC813183 (non-ribosomal peptide synthetase), KC842337-KC842340 (polyketide synthase)	**Medium**	MN w/ vit. B12
**Collector**	Rui Seabra	**Strain Status**	non-axenic, unicyanobacterial
**Collection Date**	2006/12/11	**Preservation type**	subculturing
**Location**	Portugal: Praia do Martinhal, Vila do Bispo	**Light:dark cycle**	12:12-h
**Latitude & Longitude**	37.01869 N 8.926714 W	**Temperature (ºC)**	19
		**Light intensity**	10–30 µmol photons m−2 s−1
**References Publicly Available Articles**	Brito et al. (2012) Syst. Appl. Microbiol. 35, 110–119; Brito et al. (2015) Algal Res. 9, 218-226; Brito et al. (2017) Mol. Phylog. Evol. doi:10.1016/j.ympev.2017.03.006		
**References Publicly Available Theses**			

**Strain ID**	**Lusitaniella coriacea LEGE 07157**		
**Strain Taxonomy**	Lusitaniella coriacea Ramos, V; Brito, A & Kaštovský, J in Brito et al (2017)		
**Microphotograph 400×**	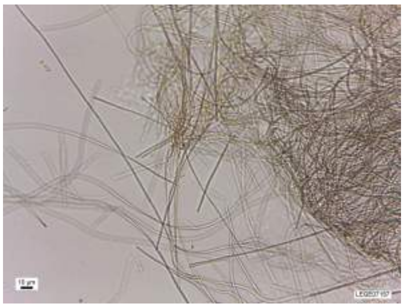	**Microphotograph 1000×**	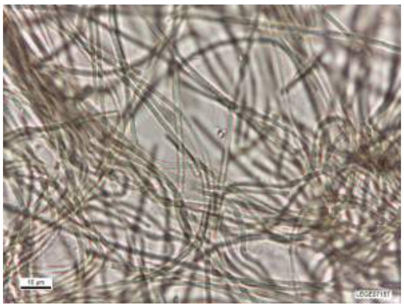
**Coidentity**	n.a.	**Morphometrics (Width × Length or Diameter) (µm)**	1,6±0,2 × 3,2±0,4
**Other Code(s)**	LEAN 071	**Taxonomic notes/diacritical features**	
**Parent Sample**	P.07 LAV 3 - ABA	**Macroscopic growth features**	color: olive green; forming a leather-like biofilm
**Isolation Date**		**Environment**	aquatic, marine
**Order (Older Classification Scheme)**	Oscillatoriales	**Habitat Sample Description**	tide pool, rock surface scraping
**Order 2014 (Modern Taxonomy)**	undefined	**Ecophysiological traits**	
**Taxonomy Notes**	Paratype of Lusitaniella coriacea Ramos, Brito et Kaštovský gen. nov., sp. nov.	**Notes on ecophysiological traits**	
**Accession Number(s)_16S**	HQ832916	**Isolator**	Vitor Ramos
**Accession Number(s)_others (product/gene /region)**		**Medium**	MN w/ vit. B12
**Collector**	Rui Seabra/Vitor Ramos	**Strain Status**	non-axenic, unicyanobacterial
**Collection Date**	2007/05/15	**Preservation type**	subculturing
**Location**	Portugal: Praia de Lavadores, Canidelo	**Light:dark cycle**	12:12-h
**Latitude & Longitude**	41.12919 N 8.668578 W	**Temperature (ºC)**	19
		**Light intensity**	10–30 µmol photons m−2 s−1
**References Publicly Available Articles**	Brito et al. (2012) Syst. Appl. Microbiol. 35, 110–119; Leão et al. (2013) Mar. Drugs 11 (4), 1316-1335; Dvořák et al. (2014) Mol. Ecol. 23, 5538–5551; Brito et al. (2017) Mol. Phylog. Evol. doi:10.1016/j.ympev.2017.03.006		
**References Publicly Available Theses**			

Strain ID	**Lusitaniella coriacea LEGE 07167**		
**Strain Taxonomy**	Lusitaniella coriacea Ramos, V; Brito, A & Kaštovský, J in Brito et al (2017)		
**Microphotograph 400×**	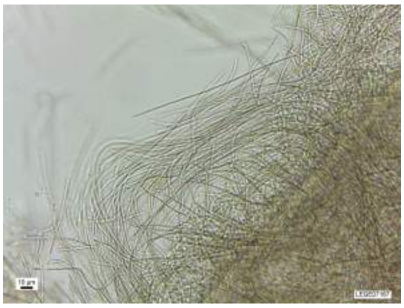	**Microphotograph 1000×**	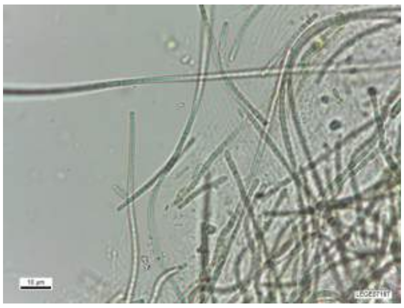
**Coidentity**	n.a.	**Morphometrics (Width × Length or Diameter) (µm)**	1,6±0,2 × 3,4±0,6
**Other Code(s)**	LEAN 081	**Taxonomic notes/diacritical features**	
**Parent Sample**	P.07 LAV 3 - FA	**Macroscopic growth features**	color: olive green; forming a leather-like biofilm
**Isolation Date**	2007	**Environment**	aquatic, marine
**Order (Older Classification Scheme)**	Oscillatoriales	**Habitat Sample Description**	tide puddle, rock surface scraping
**Order 2014 (Modern Taxonomy)**	Oscillatoriales	**Ecophysiological traits**	
**Taxonomy Notes**	This is the Type strain of Lusitaniella coriacea Ramos, Brito et Kaštovský gen. nov., sp. nov.	**Notes on ecophysiological traits**	
**Accession Number(s)_16S**	KU951853	**Isolator**	Vitor Ramos
**Accession Number(s)_others (product/gene /region)**	KC842317 (polyketide synthase)	**Medium**	MN w/ vit. B12
**Collector**	Rui Seabra/Vitor Ramos	**Strain Status**	non-axenic, unicyanobacterial
**Collection Date**	2007/05/15	**Preservation type**	subculturing
**Location**	Portugal: Praia de Lavadores, Canidelo	**Light:dark cycle**	12:12-h
**Latitude & Longitude**	41.12919 N 8.668578 W	**Temperature (ºC)**	19
		**Light intensity**	10–30 µmol photons m−2 s−1
**References Publicly Available Articles**	Brito et al. (2012) Syst. Appl. Microbiol. 35, 110–119; Costa et al. (2014) Mar. Drugs 12, 98-114; Brito et al. (2015) Algal Res. 9, 218-226; Brito et al. (2017) Mol. Phylog. Evol. doi:10.1016/j.ympev.2017.03.006		
**References Publicly Available Theses**			

**Strain ID**	**Lusitaniella coriacea LEGE 07180**		
**Strain Taxonomy**	Lusitaniella coriacea Ramos, V; Brito, A & Kaštovský, J in Brito et al (2017)		
**Microphotograph 400×**	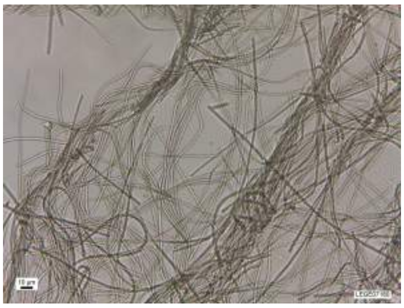	**Microphotograph 1000×**	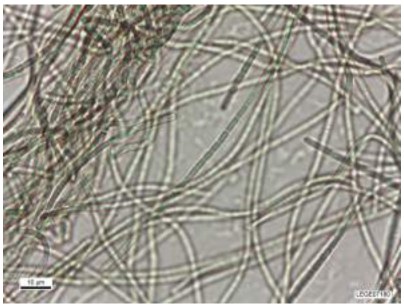
**Coidentity**	n.a.	**Morphometrics (Width × Length or Diameter) (µm)**	1,9±0,2 × 2,8±0,5
**Other Code(s)**	LEAN 094	**Taxonomic notes/diacritical features**	
**Parent Sample**	P.07 MOL 4 - AX	**Macroscopic growth features**	color: olive green; forming a leather-like biofilm
**Isolation Date**		**Environment**	aquatic, marine
**Order (Older Classification Scheme)**	Oscillatoriales	**Habitat Sample Description**	tide pool, on a submerged stone
**Order 2014 (Modern Taxonomy)**	undefined	**Ecophysiological traits**	
**Taxonomy Notes**		**Notes on ecophysiological traits**	
**Accession Number(s)_16S**	KU951855	**Isolator**	Vitor Ramos
**Accession Number(s)_others (product/gene /region)**		**Medium**	MN w/ vit. B12
**Collector**	Rui Seabra/Vitor Ramos	**Strain Status**	non-axenic, unicyanobacterial
**Collection Date**	2007/05/21	**Preservation type**	subculturing
**Location**	Portugal: Praia de Moledo, Caminha	**Light:dark cycle**	12:12-h
**Latitude & Longitude**	41.84963 N 8.866717 W	**Temperature (ºC)**	19
		**Light intensity**	10–30 µmol photons $m^{-2} s^{-1}$
**References Publicly Available Articles**			
**References Publicly Available Theses**			

Strain ID	**Microcoleus sp. LEGE 07076**		
**Strain Taxonomy**	Microcoleus Desmazières ex Gomont (1892)		
**Microphotograph 400×**	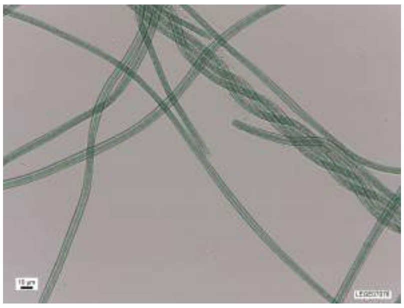	**Microphotograph 1000×**	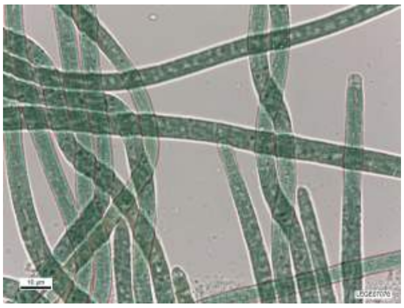
**Coidentity**	n.a.	**Morphometrics (Width × Length or Diameter) (µm)**	5,9±0,5 × 3,7±0,5
**Other Code(s)**		**Taxonomic notes/diacritical features**	facultative sheath
**Parent Sample**	VL 255	**Macroscopic growth features**	color: cyano; forming a smooth biofilm
**Isolation Date**		**Environment**	aquatic, brackish
**Order (Older Classification Scheme)**	Oscillatoriales	**Habitat Sample Description**	mesotidal zone, benthic
**Order 2014 (Modern Taxonomy)**	Oscillatoriales	**Ecophysiological traits**	
**Taxonomy Notes**		**Notes on ecophysiological traits**	
**Accession Number(s)_16S**	HM217081	**Isolator**	Viviana Lopes
**Accession Number(s)_others (product/gene /region)**		**Medium**	Z8
**Collector**	Viviana Lopes	**Strain Status**	non-axenic, unicyanobacterial
**Collection Date**	May-2007	**Preservation type**	subculturing
**Location**	Portugal: Minho estuary, Seixas	**Light:dark cycle**	12:12-h
**Latitude & Longitude**	41.90181 N 8.813139 W	**Temperature (ºC)**	19
		**Light intensity**	10–30 µmol photons m−2 s−1
**References Publicly Available Articles**	Lopes et al. (2010) Mar. Drugs 8, 471-482; Baptista et al. (2011) Toxicon 58, 410–414; Lopes et al. (2011) Toxicol. In Vitro 25, 944–950; Lopes & Vasconcelos (2011) Mar. Drugs 9, 790-802; Cianca et al. (2012) Amino Acids 42, 2473–2479; Lopes et al. (2012) Mar. Environ. Res. 73, 7–16; Almeida et al. (2015) Toxins 7(8), 2739-2756		
**References Publicly Available Theses**	Lopes V.R. (2011) PhD Thesis, FCUP, University of Porto, Porto, Portugal		

**Strain ID**	**Microcystis aeruginosa LEGE 00063**		
**Strain Taxonomy**	Microcystis aeruginosa (Kützing) Kützing, FT (1846)		
**Microphotograph 400×**	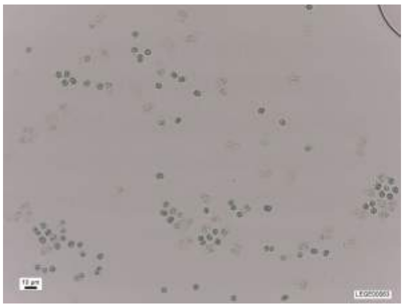	**Microphotograph 1000×**	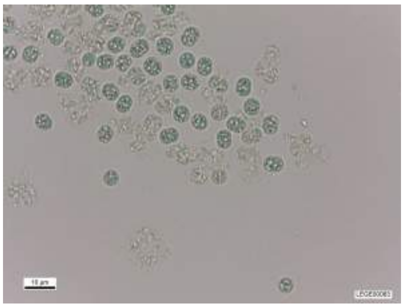
**Coidentity**	n.a.	**Morphometrics (Width × Length or Diameter) (µm)**	Ø 4,4±0,4
**Other Code(s)**	MG (original), M6	**Taxonomic notes/diacritical features**	vacuolated cells
**Parent Sample**		**Macroscopic growth features**	color: cyano; growing homogeneously
**Isolation Date**		**Environment**	aquatic, freshwater
**Order (Older Classification Scheme)**	Chroococcales	**Habitat Sample Description**	water sample, pond
**Order 2014 (Modern Taxonomy)**	Chroococcales	**Ecophysiological traits**	toxin production
**Taxonomy Notes**		**Notes on ecophysiological traits**	MC producing strain (MC-FR, MC-LR and MC-WR)
**Accession Number(s)_16S**	KU951737	**Isolator**	Carla Garcia
**Accession Number(s)_others (product/gene /region)**		**Medium**	Z8
**Collector**	Carla Garcia	**Strain Status**	non-axenic, unicyanobacterial
**Collection Date**	2000/05/16	**Preservation type**	subculturing
**Location**	Portugal: Lagoa de Mira	**Light:dark cycle**	12:12-h
**Latitude & Longitude**	unknown	**Temperature (ºC)**	19
		**Light intensity**	10–30 µmol photons m−2 s−1
**References Publicly Available Articles**	Saker et al. (2007) Appl. Microbiol. Biotechnol. 75, 441–449; Morais et al. (2008) Eur. Food Res. Technol. 227, 949–952; Fernandes et al. (2009) J. Exp. Zool. 311A, 226–230; Leão et al. (2009) Eur. J. Phycol. 44, 347-355; Martins et al. (2011) Mar. Drugs 9, 1715-1730; Fraga et al. (2014) Anal. Chim. Acta 850, 57-64		
**References Publicly Available Theses**	Garcia C. (2001) MSc Dissertation, FCUP, University of Porto, Porto, Portugal; Fernandes S. (2008) MSc Dissertation, FCUP, University of Porto, Porto, Portugal; Leão P.N. (2010) PhD Thesis, FCUP, University of Porto, Porto, Portugal; Mendes R.A. (2014) MSc Dissertation, FCUP, University of Porto, Porto, Portugal		

Strain ID	**Microcystis aeruginosa LEGE 00239**		
**Strain Taxonomy**	Microcystis aeruginosa (Kützing) Kützing, FT (1846)		
**Microphotograph 400×**	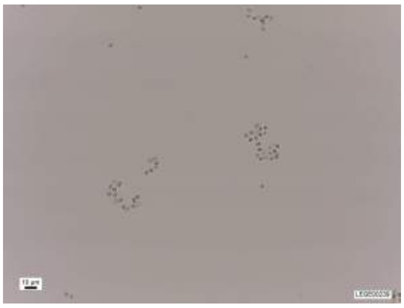	**Microphotograph 1000×**	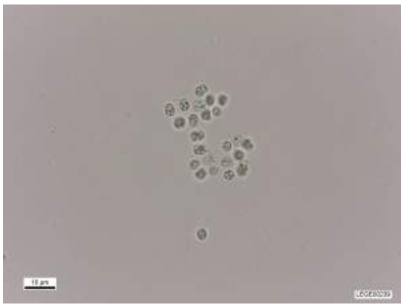
**Coidentity**	n.a.	**Morphometrics (Width × Length or Diameter) (µm)**	Ø 3,1±0,4
**Other Code(s)**	J11, LEANJ.11	**Taxonomic notes/diacritical features**	vacuolated cells
**Parent Sample**		**Macroscopic growth features**	color: cyano; growing homogeneously
**Isolation Date**		**Environment**	aquatic, freshwater
**Order (Older Classification Scheme)**	Chroococcales	**Habitat Sample Description**	water sample
**Order 2014 (Modern Taxonomy)**	Chroococcales	**Ecophysiological traits**	
**Taxonomy Notes**		**Notes on ecophysiological traits**	ANTX-a (+)/(-) by MS
**Accession Number(s)_16S**	KU951738	**Isolator**	Joana Osswald
**Accession Number(s)_others (product/gene /region)**		**Medium**	Z8
**Collector**	Joana Oswald	**Strain Status**	non-axenic, unicyanobacterial
**Collection Date**	2000/07/07	**Preservation type**	subculturing
**Location**	Portugal: Maranhão dam reservoir, campsite at Avis	**Light:dark cycle**	12:12-h
**Latitude & Longitude**	unknown	**Temperature (ºC)**	19
		**Light intensity**	10–30 µmol photons m−2 s−1
**References Publicly Available Articles**			
**References Publicly Available Theses**	Osswald J. (2002) MSc Dissertation, FCUP, University of Porto, Porto, Portugal; Osswald J. (2007) PhD Thesis, FCUP, University of Porto, Porto, Portugal		

Strain ID	**Microcystis aeruginosa LEGE 05195**		
**Strain Taxonomy**	Microcystis aeruginosa (Kützing) Kützing, FT (1846)		
**Microphotograph 400×**	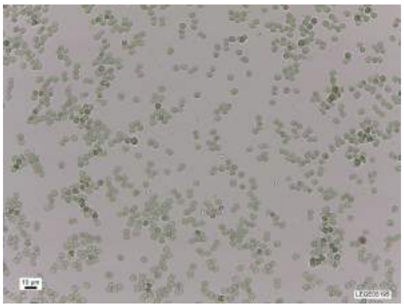	**Microphotograph 1000×**	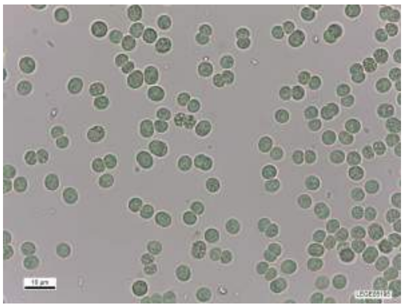
**Coidentity**	n.a.	**Morphometrics (Width × Length or Diameter) (µm)**	Ø 4,4±0,4
**Other Code(s)**	AM Vivo 11	**Taxonomic notes/diacritical features**	vacuolated cells
**Parent Sample**		**Macroscopic growth features**	color: cyano; forming a smooth biofilm
**Isolation Date**		**Environment**	aquatic, freshwater
**Order (Older Classification Scheme)**	Chroococcales	**Habitat Sample Description**	water sample
**Order 2014 (Modern Taxonomy)**	Chroococcales	**Ecophysiological traits**	toxin production
**Taxonomy Notes**		**Notes on ecophysiological traits**	MC producing strain (MC-LR, MC-RR, MC-YR)
**Accession Number(s)_16S**	KU951739	**Isolator**	Joana Martins
**Accession Number(s)_others (product/gene /region)**	KC311980 (16S-23S rRNA ITS), KC312007 (DNA gyrase subunit ß, gyrB), KC312034 (cell division protein, ftsZ)	**Medium**	Z8
**Collector**	Joana Martins	**Strain Status**	non-axenic, unicyanobacterial
**Collection Date**	2005/10/01	**Preservation type**	subculturing
**Location**	Portugal: Tamega River, Amarante	**Light:dark cycle**	12:12-h
**Latitude & Longitude**	unknown	**Temperature (ºC)**	19
		**Light intensity**	10–30 µmol photons m−2 s−1
**References Publicly Available Articles**	Leão et al. (2009) Eur. J. Phycol. 44, 347-355; Martins et al. (2009) Appl. Microbiol. Biotechnol. 82, 951–961; Stoichev et al. (2010) Chem. Ecol. 26(5), 395-399; Stoichev et al. (2011) Ecotoxicol. Environ. Saf. 74, 219–224; Fraga et al. (2014) Anal. Chim. Acta 850, 57-64; de Morais et al. (2014) Water Res. 52, 63-72; de Morais et al. (2014) Aquat. Toxicol. 150, 159–164; Almeida et al. (2015) Toxins 7(8), 2739-2756		
**References Publicly Available Theses**	Leão P.N. (2010) PhD Thesis, FCUP, University of Porto, Porto, Portugal; de Morais P. (2015) PhD Thesis, FCUP, University of Porto, Porto, Portugal		

Strain ID	**Microcystis aeruginosa LEGE 08327**		
**Strain Taxonomy**	Microcystis aeruginosa (Kützing) Kützing, FT (1846)		
**Microphotograph 400×**	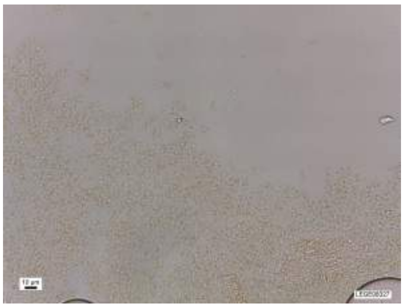	**Microphotograph 1000×**	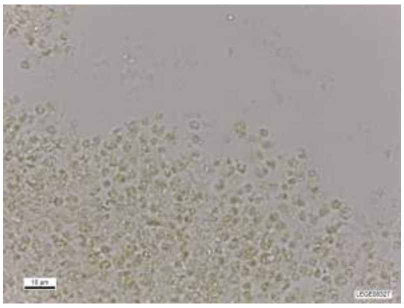
**Coidentity**	n.a.	**Morphometrics (Width × Length or Diameter) (µm)**	Ø 2,6±0,3
**Other Code(s)**		**Taxonomic notes/diacritical features**	
**Parent Sample**		**Macroscopic growth features**	color: cyano; forming aggregates/colonies
**Isolation Date**		**Environment**	aquatic, freshwater
**Order (Older Classification Scheme)**	Chroococcales	**Habitat Sample Description**	water sample
**Order 2014 (Modern Taxonomy)**	Chroococcales	**Ecophysiological traits**	
**Taxonomy Notes**		**Notes on ecophysiological traits**	
**Accession Number(s)_16S**	KU951740	**Isolator**	Vitor Vasconcelos
**Accession Number(s)_others (product/gene /region)**		**Medium**	Z8
**Collector**	Vitor Vasconcelos	**Strain Status**	non-axenic, unicyanobacterial
**Collection Date**	2008	**Preservation type**	subculturing
**Location**	Mexico: Lake Zumpango	**Light:dark cycle**	12:12-h
**Latitude & Longitude**	unknown	**Temperature (ºC)**	19
		**Light intensity**	10–30 µmol photons m−2 s−1
**References Publicly Available Articles**			
**References Publicly Available Theses**			

Strain ID	**Microcystis aeruginosa LEGE 08328**		
**Strain Taxonomy**	Microcystis aeruginosa (Kützing) Kützing, FT (1846)		
**Microphotograph 400×**	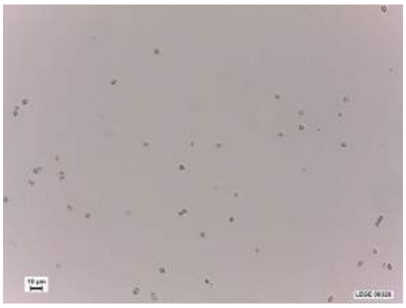	**Microphotograph 1000×**	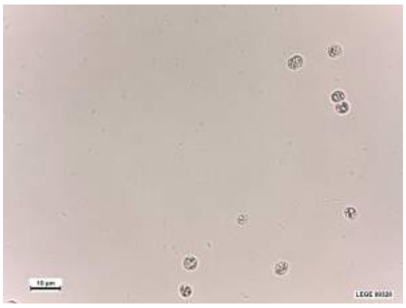
**Coidentity**	n.a.	**Morphometrics (Width × Length or Diameter) (µm)**	Ø 3,2±0,5
**Other Code(s)**		**Taxonomic notes/diacritical features**	
**Parent Sample**		**Macroscopic growth features**	color: green-brownish; forming aggregates/colonies
**Isolation Date**		**Environment**	aquatic, freshwater
**Order (Older Classification Scheme)**	Chroococcales	**Habitat Sample Description**	water sample
**Order 2014 (Modern Taxonomy)**	Chroococcales	**Ecophysiological traits**	
**Taxonomy Notes**		**Notes on ecophysiological traits**	
**Accession Number(s)_16S**	KF287006	**Isolator**	Vitor Vasconcelos
**Accession Number(s)_others (product/gene /region)**	KF008261 (cyanobactin, N-terminal protease (A)), KF287031 (16S-23S rRNA ITS), KF287056 (DNA gyrase subunit ß, gyrB), KF287081 (cell division protein, ftsZ)	**Medium**	Z8
**Collector**	Vitor Vasconcelos	**Strain Status**	non-axenic, unicyanobacterial, delicate
**Collection Date**	2008	**Preservation type**	subculturing
**Location**	Mexico: Lake Zumpango	**Light:dark cycle**	12:12-h
**Latitude & Longitude**	unknown	**Temperature (ºC)**	19
		**Light intensity**	10–30 µmol photons m−2 s−1
**References Publicly Available Articles**	Martins et al. (2013) Mar. Drugs 11 (12), 4902-4916; Moreira et al. (2014) Curr. Microbiol. 69, 628–633		
**References Publicly Available Theses**			

**Strain ID**	**Microcystis aeruginosa LEGE 08329**		
**Strain Taxonomy**	Microcystis aeruginosa (Kützing) Kützing, FT (1846)		
**Microphotograph 400×**	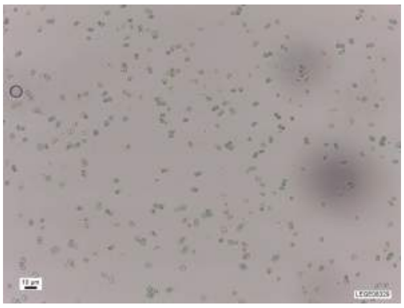	**Microphotograph 1000×**	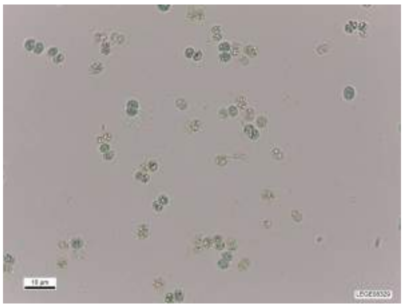
**Coidentity**	n.a.	**Morphometrics (Width × Length or Diameter) (µm)**	Ø 3,6±0,6
**Other Code(s)**		**Taxonomic notes/diacritical features**	
**Parent Sample**		**Macroscopic growth features**	color: cyano; growing homogeneously, but also forming aggregates
**Isolation Date**		**Environment**	aquatic, freshwater
**Order (Older Classification Scheme)**	Chroococcales	**Habitat Sample Description**	water sample
**Order 2014 (Modern Taxonomy)**	Chroococcales	**Ecophysiological traits**	
**Taxonomy Notes**		**Notes on ecophysiological traits**	
**Accession Number(s)_16S**	KF287007	**Isolator**	Vitor Vasconcelos
**Accession Number(s)_others (product/gene /region)**	KF287032 (16S-23S rRNA ITS), KF287057 (DNA gyrase subunit ß, gyrB), KF287082 (cell division protein, ftsZ)	**Medium**	Z8
**Collector**	Vitor Vasconcelos	**Strain Status**	non-axenic, unicyanobacterial
**Collection Date**	2008	**Preservation type**	subculturing
**Location**	Mexico: Cuemanco, man-made channel	**Light:dark cycle**	12:12-h
**Latitude & Longitude**	unknown	**Temperature (ºC)**	19
		**Light intensity**	10–30 µmol photons m−2 s−1
**References Publicly Available Articles**	Moreira et al. (2014) Curr. Microbiol. 69, 628–633		
**References Publicly Available Theses**			

**Strain ID**	**Microcystis aeruginosa LEGE 08330**		
**Strain Taxonomy**	Microcystis aeruginosa (Kützing) Kützing, FT (1846)		
**Microphotograph 400×**	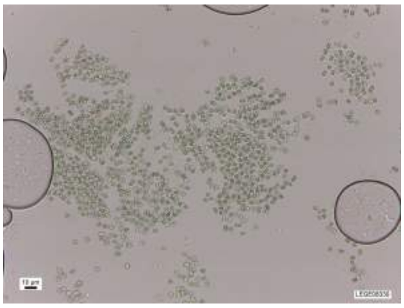	**Microphotograph 1000×**	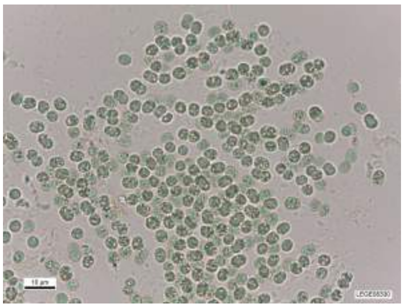
**Coidentity**	n.a.	**Morphometrics (Width × Length or Diameter) (µm)**	Ø 3,4±0,2
**Other Code(s)**		**Taxonomic notes/diacritical features**	mucilaginous colonies
**Parent Sample**		**Macroscopic growth features**	color: cyano; forming aggregates
**Isolation Date**		**Environment**	aquatic, freshwater
**Order (Older Classification Scheme)**	Chroococcales	**Habitat Sample Description**	water sample
**Order 2014 (Modern Taxonomy)**	Chroococcales	**Ecophysiological traits**	EPS production
**Taxonomy Notes**		**Notes on ecophysiological traits**	
**Accession Number(s)_16S**	KF287008	**Isolator**	Vitor Vasconcelos
**Accession Number(s)_others (product/gene /region)**	KF287033 (16S-23S rRNA ITS),<br>KF287058 (DNA gyrase subunit ß, gyrB),<br>KF287083 (cell division protein, ftsZ)	**Medium**	Z8
**Collector**	Vitor Vasconcelos	**Strain Status**	non-axenic, unicyanobacterial
**Collection Date**	2008	**Preservation type**	subculturing, cryopreserved
**Location**	Mexico: Chapultepec, artificial lake	**Light:dark cycle**	12:12-h
**Latitude & Longitude**	unknown	**Temperature (ºC)**	19
		**Light intensity**	10–30 µmol photons m−2 s−1
**References Publicly Available Articles**	Moreira et al. (2014) Curr. Microbiol. 69, 628–633		
**References Publicly Available Theses**			

Strain ID	**Microcystis aeruginosa LEGE 08331**		
**Strain Taxonomy**	Microcystis aeruginosa (Kützing) Kützing, FT (1846)		
**Microphotograph 400×**	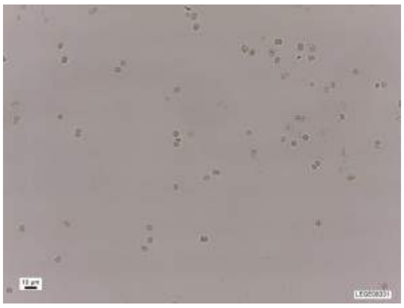	**Microphotograph 1000×**	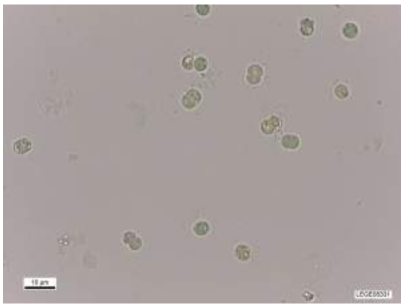
**Coidentity**	n.a.	**Morphometrics (Width × Length or Diameter) (µm)**	Ø 4,4±0,4
**Other Code(s)**		**Taxonomic notes/diacritical features**	
**Parent Sample**		**Macroscopic growth features**	color: cyano; growing homogeneously, but also forming aggregates
**Isolation Date**		**Environment**	aquatic, freshwater
**Order (Older Classification Scheme)**	Chroococcales	**Habitat Sample Description**	water sample
**Order 2014 (Modern Taxonomy)**	Chroococcales	**Ecophysiological traits**	
**Taxonomy Notes**		**Notes on ecophysiological traits**	
**Accession Number(s)_16S**	KC989707	**Isolator**	Vitor Vasconcelos
**Accession Number(s)_others (product/gene /region)**	KF008264 (cyanobactin, N-terminal protease (A))	**Medium**	Z8
**Collector**	Vitor Vasconcelos	**Strain Status**	non-axenic, unicyanobacterial
**Collection Date**	2008	**Preservation type**	subculturing
**Location**	Mexico: Cuemanco, man-made channel	**Light:dark cycle**	12:12-h
**Latitude & Longitude**	unknown	**Temperature (ºC)**	19
		**Light intensity**	10–30 µmol photons m−2 s−1
**References Publicly Available Articles**	Martins et al. (2013) Mar. Drugs 11 (12), 4902-4916		
**References Publicly Available Theses**			

**Strain ID**	**Microcystis aeruginosa LEGE 08354**		
**Strain Taxonomy**	Microcystis aeruginosa (Kützing) Kützing, FT (1846)		
**Microphotograph 400×**	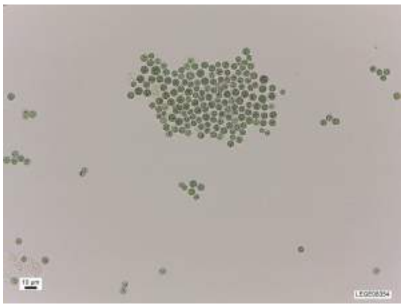	**Microphotograph 1000×**	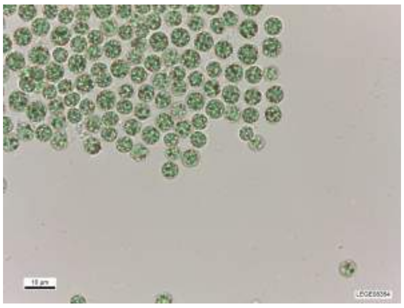
**Coidentity**	n.a.	**Morphometrics (Width × Length or Diameter) (µm)**	Ø 5,4±0,6
**Other Code(s)**	strain 9	**Taxonomic notes/diacritical features**	
**Parent Sample**	MMa15E	**Macroscopic growth features**	color: cyano; growing homogeneously, but also forming aggregates
**Isolation Date**		**Environment**	aquatic, freshwater
**Order (Older Classification Scheme)**	Chroococcales	**Habitat Sample Description**	water sample
**Order 2014 (Modern Taxonomy)**	Chroococcales	**Ecophysiological traits**	
**Taxonomy Notes**		**Notes on ecophysiological traits**	
**Accession Number(s)_16S**	KU951741	**Isolator**	Ana Regueiras
**Accession Number(s)_others (product/gene /region)**		**Medium**	Z8
**Collector**	Ana Regueiras	**Strain Status**	non-axenic, unicyanobacterial
**Collection Date**	2008/10/15	**Preservation type**	subculturing
**Location**	Portugal: Tamega River, Marco de Canaveses	**Light:dark cycle**	12:12-h
**Latitude & Longitude**	unknown	**Temperature (ºC)**	19
		**Light intensity**	10–30 µmol photons m−2 s−1
**References Publicly Available Articles**			
**References Publicly Available Theses**	Regueiras A. (2009) MSc Dissertation, ICBAS and FCUP, University of Porto, Porto, Portugal		

Strain ID	**Microcystis aeruginosa LEGE 11464**		
**Strain Taxonomy**	Microcystis aeruginosa (Kützing) Kützing, FT (1846)		
**Microphotograph 400×**	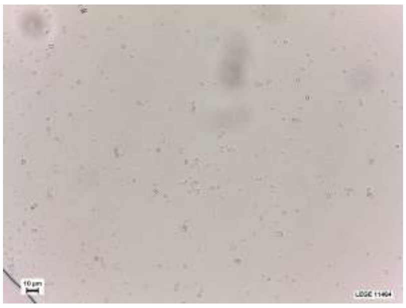	**Microphotograph 1000×**	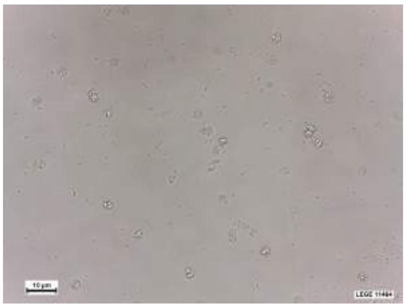
**Coidentity**	n.a.	**Morphometrics (Width × Length or Diameter) (µm)**	Ø 2,5±0,3
**Other Code(s)**	MGR 2.1	**Taxonomic notes/diacritical features**	
**Parent Sample**		**Macroscopic growth features**	color: cyano-greenish; growing homogeneously, but also forming aggregates
**Isolation Date**		**Environment**	aquatic, freshwater
**Order (Older Classification Scheme)**	Chroococcales	**Habitat Sample Description**	bloom sample
**Order 2014 (Modern Taxonomy)**	Chroococcales	**Ecophysiological traits**	
**Taxonomy Notes**		**Notes on ecophysiological traits**	
**Accession Number(s)_16S**	KU951742	**Isolator**	Cristiana Moreira
**Accession Number(s)_others (product/gene /region)**		**Medium**	Z8
**Collector**	Spyros Gkelis	**Strain Status**	non-axenic, unicyanobacterial
**Collection Date**	2011	**Preservation type**	subculturing
**Location**	Greece	**Light:dark cycle**	12:12-h
**Latitude & Longitude**	unknown	**Temperature (ºC)**	19
		**Light intensity**	10–30 µmol photons m−2 s−1
**References Publicly Available Articles**	Moreira et al. (2014) Curr. Microbiol. 69, 628–633		
**References Publicly Available Theses**			

**Strain ID**	**Microcystis aeruginosa LEGE 11465**		
**Strain Taxonomy**	Microcystis aeruginosa (Kützing) Kützing, FT (1846)		
**Microphotograph 400×**	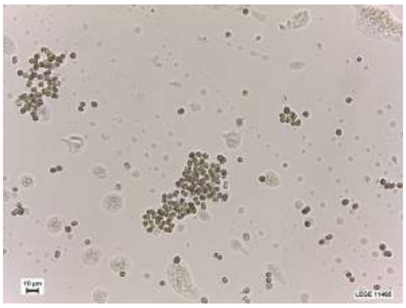	**Microphotograph 1000×**	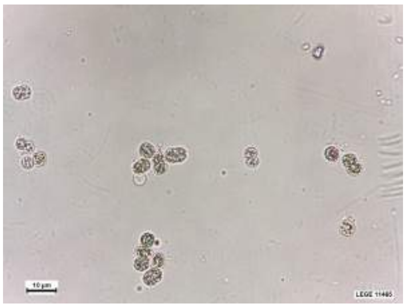
**Coidentity**	n.a.	**Morphometrics (Width × Length or Diameter) (µm)**	Ø 4,0±0,7
**Other Code(s)**	MGR 1.7	**Taxonomic notes/diacritical features**	
**Parent Sample**		**Macroscopic growth features**	color: cyano-greenish; growing homogeneously, but also forming aggregates
**Isolation Date**		**Environment**	aquatic, freshwater
**Order (Older Classification Scheme)**	Chroococcales	**Habitat Sample Description**	bloom sample
**Order 2014 (Modern Taxonomy)**	Chroococcales	**Ecophysiological traits**	
**Taxonomy Notes**		**Notes on ecophysiological traits**	
**Accession Number(s)_16S**		**Isolator**	Cristiana Moreira
**Accession Number(s)_others (product/gene /region)**		**Medium**	Z8
**Collector**	Spyros Gkelis	**Strain Status**	non-axenic, unicyanobacterial
**Collection Date**	2011	**Preservation type**	subculturing
**Location**	Greece	**Light:dark cycle**	12:12-h
**Latitude & Longitude**	unknown	**Temperature (ºC)**	19
		**Light intensity**	10–30 µmol photons m−2 s−1
**References Publicly Available Articles**			
**References Publicly Available Theses**			

**Strain ID**	**Microcystis aeruginosa LEGE 12460**		
**Strain Taxonomy**	Microcystis aeruginosa (Kützing) Kützing, FT (1846)		
**Microphotograph 400×**	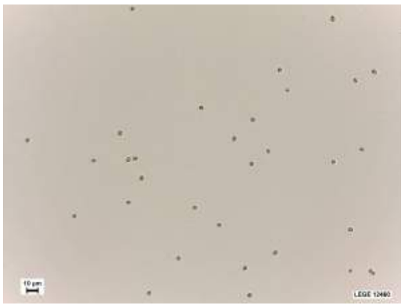	**Microphotograph 1000×**	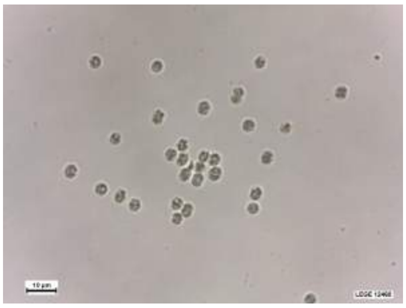
**Coidentity**	n.a.	**Morphometrics (Width × Length or Diameter) (µm)**	Ø 3,0±0,6
**Other Code(s)**		**Taxonomic notes/diacritical features**	
**Parent Sample**	5.1 Pampulha	**Macroscopic growth features**	color: cyano; growing homogeneously
**Isolation Date**		**Environment**	aquatic, freshwater
**Order (Older Classification Scheme)**	Chroococcales	**Habitat Sample Description**	bloom sample, pond
**Order 2014 (Modern Taxonomy)**	Chroococcales	**Ecophysiological traits**	
**Taxonomy Notes**		**Notes on ecophysiological traits**	
**Accession Number(s)_16S**	KU951743	**Isolator**	Cristiana Moreira
**Accession Number(s)_others (product/gene /region)**		**Medium**	Z8
**Collector**	Vitor Vasconcelos	**Strain Status**	non-axenic, unicyanobacterial
**Collection Date**	2012	**Preservation type**	subculturing
**Location**	Brazil: Lagoa da Pampulha, Belo Horizonte	**Light:dark cycle**	12:12-h
**Latitude & Longitude**	unknown	**Temperature (ºC)**	19
		**Light intensity**	10–30 µmol photons m−2 s−1
**References Publicly Available Articles**			
**References Publicly Available Theses**			

Strain ID	**Microcystis aeruginosa LEGE 12461**		
**Strain Taxonomy**	Microcystis aeruginosa (Kützing) Kützing, FT (1846)		
**Microphotograph 400×**	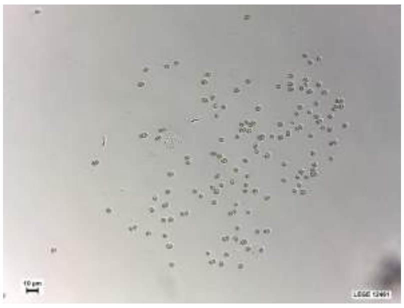	**Microphotograph 1000×**	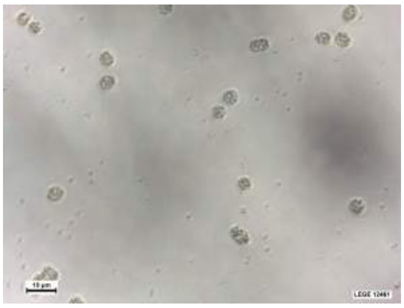
**Coidentity**	n.a.	**Morphometrics (Width × Length or Diameter) (µm)**	Ø 3,8±0,6
**Other Code(s)**		**Taxonomic notes/diacritical features**	
**Parent Sample**	5.2 Pampulha	**Macroscopic growth features**	color: cyano-greenish; growing homogeneously
**Isolation Date**		**Environment**	aquatic, freshwater
**Order (Older Classification Scheme)**	Chroococcales	**Habitat Sample Description**	bloom sample, pond
**Order 2014 (Modern Taxonomy)**	Chroococcales	**Ecophysiological traits**	
**Taxonomy Notes**		**Notes on ecophysiological traits**	
**Accession Number(s)_16S**	KU951744	**Isolator**	Cristiana Moreira
**Accession Number(s)_others (product/gene /region)**		**Medium**	Z8
**Collector**	Vitor Vasconcelos	**Strain Status**	non-axenic, unicyanobacterial
**Collection Date**	2012	**Preservation type**	subculturing
**Location**	Brazil: Lagoa da Pampulha, Belo Horizonte	**Light:dark cycle**	12:12-h
**Latitude & Longitude**	unknown	**Temperature (ºC)**	19
		**Light intensity**	10–30 µmol photons m−2 s−1
**References Publicly Available Articles**			
**References Publicly Available Theses**			

**Strain ID**	**Microcystis aeruginosa LEGE 12462**		
**Strain Taxonomy**	Microcystis aeruginosa (Kützing) Kützing, FT (1846)		
**Microphotograph 400×**	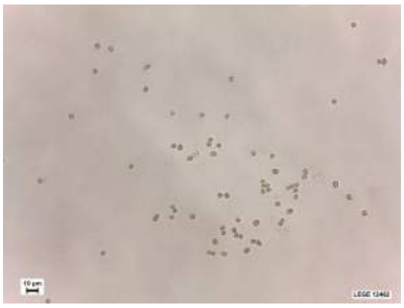	**Microphotograph 1000×**	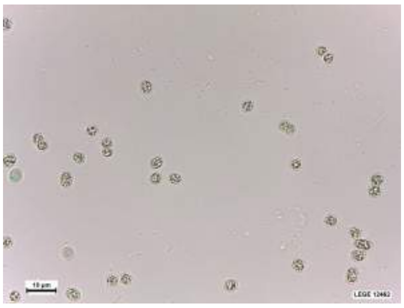
**Coidentity**	n.a.	**Morphometrics (Width × Length or Diameter) (µm)**	Ø 3,8±0,5
**Other Code(s)**		**Taxonomic notes/diacritical features**	
**Parent Sample**	5.3 Pampulha	**Macroscopic growth features**	color: cyano; growing homogeneously
**Isolation Date**		**Environment**	aquatic, freshwater
**Order (Older Classification Scheme)**	Chroococcales	**Habitat Sample Description**	bloom sample, pond
**Order 2014 (Modern Taxonomy)**	Chroococcales	**Ecophysiological traits**	
**Taxonomy Notes**		**Notes on ecophysiological traits**	
**Accession Number(s)_16S**	KU951745	**Isolator**	Cristiana Moreira
**Accession Number(s)_others (product/gene /region)**		**Medium**	Z8
**Collector**	Vitor Vasconcelos	**Strain Status**	non-axenic, unicyanobacterial
**Collection Date**	2012	**Preservation type**	subculturing
**Location**	Brazil: Lagoa da Pampulha, Belo Horizonte	**Light:dark cycle**	12:12-h
**Latitude & Longitude**	unknown	**Temperature (ºC)**	19
		**Light intensity**	10–30 µmol photons m−2 s−1
**References Publicly Available Articles**			
**References Publicly Available Theses**			

Strain ID	**Microcystis aeruginosa LEGE 12463**		
**Strain Taxonomy**	Microcystis aeruginosa (Kützing) Kützing, FT (1846)		
**Microphotograph 400×**	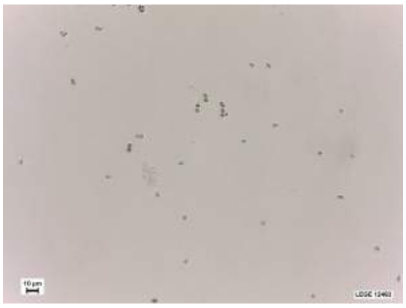	**Microphotograph 1000×**	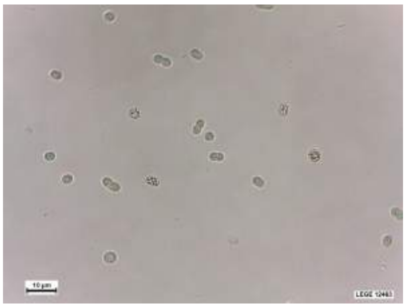
**Coidentity**	n.a.	**Morphometrics (Width × Length or Diameter) (µm)**	Ø 2,6±0,6
**Other Code(s)**		**Taxonomic notes/diacritical features**	
**Parent Sample**	5.4 Pampulha	**Macroscopic growth features**	color: cyano; growing homogeneously
**Isolation Date**		**Environment**	aquatic, freshwater
**Order (Older Classification Scheme)**	Chroococcales	**Habitat Sample Description**	bloom sample, pond
**Order 2014 (Modern Taxonomy)**	Chroococcales	**Ecophysiological traits**	
**Taxonomy Notes**		**Notes on ecophysiological traits**	
**Accession Number(s)_16S**	KU951746	**Isolator**	Cristiana Moreira
**Accession Number(s)_others (product/gene /region)**		**Medium**	Z8
**Collector**	Vitor Vasconcelos	**Strain Status**	non-axenic, unicyanobacterial
**Collection Date**	2012	**Preservation type**	subculturing
**Location**	Brazil: Lagoa da Pampulha, Belo Horizonte	**Light:dark cycle**	12:12-h
**Latitude & Longitude**	unknown	**Temperature (ºC)**	19
		**Light intensity**	10–30 µmol photons m−2 s−1
**References Publicly Available Articles**			
**References Publicly Available Theses**			

Strain ID	**Microcystis aeruginosa LEGE 91093**		
**Strain Taxonomy**	Microcystis aeruginosa (Kützing) Kützing, FT (1846)		
**Microphotograph 400×**	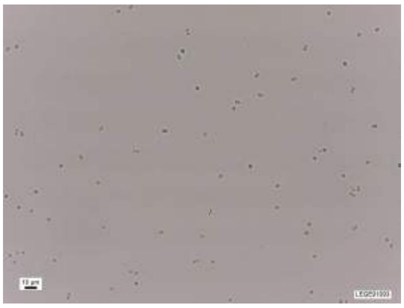	**Microphotograph 1000×**	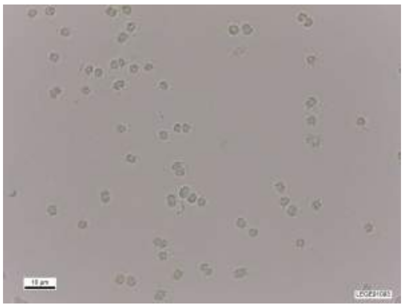
**Coidentity**	n.a.	**Morphometrics (Width × Length or Diameter) (µm)**	Ø 2,7±0,3
**Other Code(s)**	IZANCYA 1 , IZ1	**Taxonomic notes/diacritical features**	
**Parent Sample**		**Macroscopic growth features**	color: cyano; growing homogeneously
**Isolation Date**		**Environment**	aquatic, freshwater
**Order (Older Classification Scheme)**	Chroococcales	**Habitat Sample Description**	coastal lagoon, water sample
**Order 2014 (Modern Taxonomy)**	Chroococcales	**Ecophysiological traits**	toxin production
**Taxonomy Notes**		**Notes on ecophysiological traits**	MC producing strain (MC-LR, MC-LA)
**Accession Number(s)_16S**		**Isolator**	Vitor Vasconcelos
**Accession Number(s)_others (product/gene /region)**		**Medium**	Z8
**Collector**	Vitor Vasconcelos	**Strain Status**	non-axenic, unicyanobacterial
**Collection Date**	1991/04/13	**Preservation type**	subculturing, cryopreserved
**Location**	Portugal: Barrinha de Mira	**Light:dark cycle**	12:12-h
**Latitude & Longitude**	unknown	**Temperature (ºC)**	19
		**Light intensity**	10–30 µmol photons m−2 s−1
**References Publicly Available Articles**	Jokela et al. (2010) ChemBioChem 11, 1594-1599; Devlin et al. (2013) Anal. Chim. Acta. 769, 108– 113		
**References Publicly Available Theses**	Vasconcelos V.M. (1995) PhD Thesis, FCUP, University of Porto, Porto, Portugal		

**Strain ID**	**Microcystis aeruginosa LEGE 91094**		
**Strain Taxonomy**	Microcystis aeruginosa (Kützing) Kützing, FT (1846)		
**Microphotograph 400×**	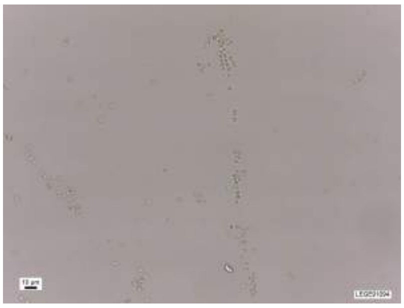	**Microphotograph 1000×**	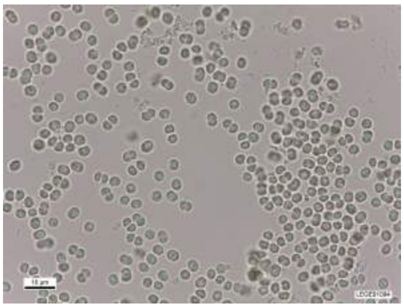
**Coidentity**	n.a.	**Morphometrics (Width × Length or Diameter) (µm)**	Ø 2,6±0,3
**Other Code(s)**	IZANCYA 2, IZ2	**Taxonomic notes/diacritical features**	
**Parent Sample**		**Macroscopic growth features**	color: cyano; growing homogeneously
**Isolation Date**		**Environment**	aquatic, freshwater
**Order (Older Classification Scheme)**	Chroococcales	**Habitat Sample Description**	pond, water sample
**Order 2014 (Modern Taxonomy)**	Chroococcales	**Ecophysiological traits**	toxin production
**Taxonomy Notes**		**Notes on ecophysiological traits**	MC producing strain (MC-LR and low amounts of MC-LA and [d-Asp3]MC-LR)
**Accession Number(s)_16S**	KU950712	**Isolator**	Vitor Vasconcelos
**Accession Number(s)_others (product/gene /region)**		**Medium**	Z8
**Collector**	Vitor Vasconcelos	**Strain Status**	non-axenic, unicyanobacterial
**Collection Date**	1991/04/13	**Preservation type**	subculturing
**Location**	Portugal: Lagoa de Mira	**Light:dark cycle**	12:12-h
**Latitude & Longitude**	unknown	**Temperature (ºC)**	19
		**Light intensity**	10–30 µmol photons m−2 s−1
**References Publicly Available Articles**	Vasconcelos (1995) Aquat. Toxicol. 32, 227-237; Amorim & Vasconcelos (1999) Toxicon 37, 1041-1052; Vasconcelos et al. (2001) Toxicon 39, 1461-1470; Martins et al. (2009) Toxicon 53, 409–416; Pereira et al. (2009) Bull. Environ. Contam. Toxicol. 83(1), 81-84; Jokela et al. (2010) ChemBioChem 11, 1594-1599; Leão et al. (2010) Proc. Natl. Acad. Sci. U.S.A. 107 (25), 11183-11188; Lopes & Vasconcelos (2011) Mar. Drugs 9, 790-802; Martins et al. (2011) Int. J. Mol. Sci. 12(12), 9172-9188; Campos et al. (2013) Ecotoxicol. Environ. Saf. 94, 45–53; Devlin et al. (2013) Anal. Chim. Acta. 769, 108–113; Pinheiro et al. (2013) Hydrobiologia 705(1), 27-42; Azevedo et al. (2014) Ecotoxicology 23, 107–121; Gutiérrez-Praena et al. (2014) Toxins 6, 1837-1854; Freitas et al. (2014) Food Chem. Toxicol. 66, 217-223; Carneiro et al. (2015) Toxins 7(6), 2096-2120; Freitas et al. (2015) Ecotoxicol. Environ. Saf. 116, 59-67; Freitas et al. (2015) Phytochemistry 110, 91-103; Pereira et al. (2015) Ecotoxicol. Environ. Saf. 118, 11-20; Pinho et al. (2015) Chem. Eng. J. 268, 144–152; Reis et al. (2015) Int. J. Mol. Sci. 16(4), 8397-8414; Pinheiro et al. (2016) Ecotoxicology, DOI: 10.1007/s10646-016-1633-y		
**References Publicly Available Theses**	Vasconcelos V.M. (1995) PhD Thesis, FCUP, University of Porto, Porto, Portugal; Amorim A. (1997) MSc Dissertation, FCUP, University of Porto, Porto, Portugal; Boas E. (1999) MSc Dissertation, FCUP, University of Porto, Porto, Portugal; Barros P. (2001) MSc Dissertation, FCUP, University of Porto, Porto, Portugal; Barros P. (2001) PhD Thesis, FCUP, University of Porto, Porto, Portugal; Leão P.N. (2010) PhD Thesis, FCUP, University of Porto, Porto, Portugal; Martins J.C. (2010) PhD Thesis, FCUP, University of Porto, Porto, Portugal; Monteiro M.B. (2011) MSc Dissertation, FCUP, University of Porto, Porto, Portugal; Caetano C. (2014) MSc Dissertation, ICBAS, University of Porto, Porto, Portugal; dos Santos C. (2014) MSc Dissertation, FCUP, University of Porto, Porto, Portugal; Freitas M. (2014) PhD Thesis, FCUP, University of Porto, Porto, Portugal; Machado J. (2014) MSc Dissertation, ICBAS, University of Porto, Porto, Portugal; Pinho L. (2014) PhD Thesis, FEUP, University of Porto, Porto, Portugal; Reis B. (2014) MSc Dissertation, FFUP, University of Porto, Porto, Portugal		

Strain ID	**Microcystis aeruginosa LEGE 91095**		
**Strain Taxonomy**	Microcystis aeruginosa (Kützing) Kützing, FT (1846)		
**Microphotograph 400×**	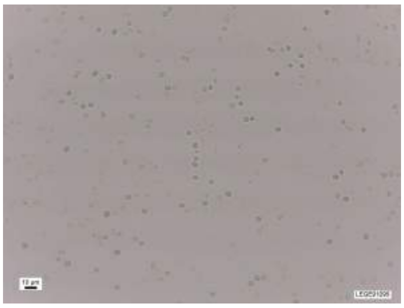	**Microphotograph 1000×**	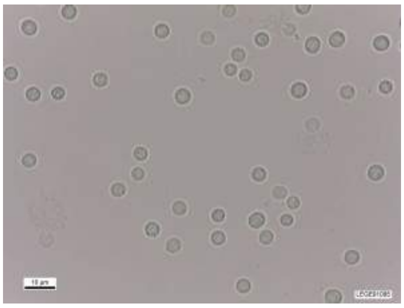
**Coidentity**	n.a.	**Morphometrics (Width × Length or Diameter) (µm)**	Ø 4,3±0,7
**Other Code(s)**	IZANCYA 25, IZ25	**Taxonomic notes/diacritical features**	
**Parent Sample**		**Macroscopic growth features**	color: cyano; growing homogeneously, but also forming aggregates
**Isolation Date**		**Environment**	aquatic, freshwater
**Order (Older Classification Scheme)**	Chroococcales	**Habitat Sample Description**	water sample
**Order 2014 (Modern Taxonomy)**	Chroococcales	**Ecophysiological traits**	toxin production
**Taxonomy Notes**		**Notes on ecophysiological traits**	MC producing strain (MC-LR, MC-YR)
**Accession Number(s)_16S**	KU951747	**Isolator**	Vitor Vasconcelos
**Accession Number(s)_others (product/gene /region)**	AY382534 (microcystin synthetase, mcyE), KC311987 (16S-23S rRNA ITS), KC312014 (DNA gyrase subunit ß, gyrB), KC312041 (cell division protein, ftsZ)	**Medium**	Z8
**Collector**	Vitor Vasconcelos	**Strain Status**	non-axenic, unicyanobacterial
**Collection Date**	Sep-1991	**Preservation type**	subculturing
**Location**	Portugal: Torrão dam reservoir, Tâmega river	**Light:dark cycle**	12:12-h
**Latitude & Longitude**	unknown	**Temperature (ºC)**	19
		**Light intensity**	10–30 µmol photons m−2 s−1
**References Publicly Available Articles**	Rantala et al. (2004) Proc. Natl. Acad. Sci. U.S.A. 101 (2), 568-573 (Table S1); Koskenniemi et al. (2007) Appl. Environ. Microbiol. 73, 2173–2179; Jokela et al. (2010) ChemBioChem 11, 1594-1599; Devlin et al. (2013) Anal. Chim. Acta. 769, 108– 113		
**References Publicly Available Theses**	Vasconcelos V.M. (1995) PhD Thesis, FCUP, University of Porto, Porto, Portugal		

Strain ID	**Microcystis aeruginosa LEGE 91096**		
**Strain Taxonomy**	Microcystis aeruginosa (Kützing) Kützing, FT (1846)		
**Microphotograph 400×**	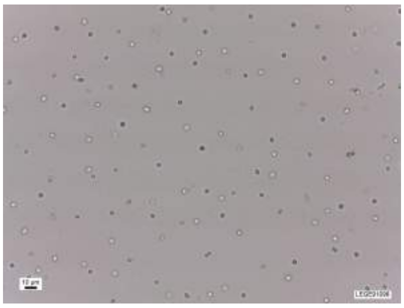	**Microphotograph 1000×**	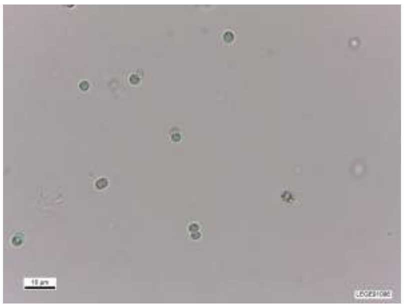
**Coidentity**	n.a.	**Morphometrics (Width × Length or Diameter) (µm)**	Ø 3,0±0,3
**Other Code(s)**	IZANCYA 33, IZ33	**Taxonomic notes/diacritical features**	vacuolated cells
**Parent Sample**		**Macroscopic growth features**	color: cyano; growing homogeneously
**Isolation Date**		**Environment**	aquatic, freshwater
**Order (Older Classification Scheme)**	Chroococcales	**Habitat Sample Description**	pond, water sample
**Order 2014 (Modern Taxonomy)**	Chroococcales	**Ecophysiological traits**	toxin production
**Taxonomy Notes**		**Notes on ecophysiological traits**	MC producing strain (MC-LR)
**Accession Number(s)_16S**		**Isolator**	Vitor Vasconcelos
**Accession Number(s)_others (product/gene /region)**		**Medium**	Z8
**Collector**	Vitor Vasconcelos	**Strain Status**	non-axenic, unicyanobacterial
**Collection Date**	Sep-1991	**Preservation type**	subculturing
**Location**	Portugal: Picote dam reservoir, Douro river	**Light:dark cycle**	12:12-h
**Latitude & Longitude**	unknown	**Temperature (ºC)**	19
		**Light intensity**	10–30 µmol photons m−2 s−1
**References Publicly Available Articles**	Pereira et al. (2009) Bull. Environ. Contam. Toxicol. 83(1), 81-84; Jokela et al. (2010) ChemBioChem 11, 1594-1599; Devlin et al. (2013) Anal. Chim. Acta. 769, 108– 113		
**References Publicly Available Theses**	Vasconcelos V.M. (1995) PhD Thesis, FCUP, University of Porto, Porto, Portugal		

Strain ID	**Microcystis aeruginosa LEGE 91338**		
**Strain Taxonomy**	Microcystis aeruginosa (Kützing) Kützing, FT (1846)		
**Microphotograph 400×**	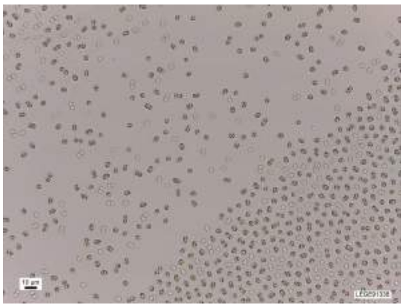	**Microphotograph 1000×**	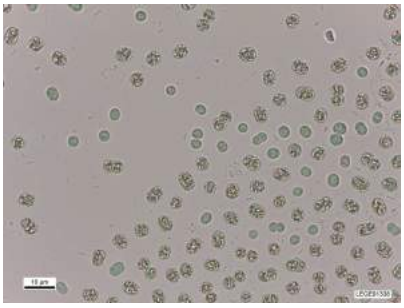
**Coidentity**	LEGE	**Morphometrics (Width × Length or Diameter) (µm)**	Ø 3,8±0,5
**Other Code(s)**	IZANCYA 3, IZ3	**Taxonomic notes/diacritical features**	mucilaginous colonies; vacuolated cells
**Parent Sample**		**Macroscopic growth features**	color: cyano; growing homogeneously, but also forming aggregates
**Isolation Date**		**Environment**	aquatic, freshwater
**Order (Older Classification Scheme)**	Chroococcales	**Habitat Sample Description**	coastal lagoon, fresh water sample
**Order 2014 (Modern Taxonomy)**	Chroococcales	**Ecophysiological traits**	toxin production, EPS production
**Taxonomy Notes**		**Notes on ecophysiological traits**	MC producing strain ([Asp3]MC-LR, MC-LR)
**Accession Number(s)_16S**		**Isolator**	Vitor Vasconcelos
**Accession Number(s)_others (product/gene /region)**		**Medium**	Z8
**Collector**	Vitor Vasconcelos	**Strain Status**	non-axenic, unicyanobacterial
**Collection Date**	1991/04/13	**Preservation type**	subculturing
**Location**	Portugal: Barrinha de Mira	**Light:dark cycle**	12:12-h
**Latitude & Longitude**	unknown	**Temperature (ºC)**	19
		**Light intensity**	10–30 µmol photons m−2 s−1
**References Publicly Available Articles**	Martins et al. (2009) Appl. Microbiol. Biotechnol. 82, 951–961; Pereira et al. (2009) Bull. Environ. Contam. Toxicol. 83(1), 81-84; Jokela et al. (2010) ChemBioChem 11, 1594-1599		
**References Publicly Available Theses**	Vasconcelos V.M. (1995) PhD Thesis, FCUP, University of Porto, Porto, Portugal; Fernandes E. (2001) MSc Dissertation, FCUP, University of Porto, Porto, Portugal		

Strain ID	**Microcystis aeruginosa LEGE 91339**		
**Strain Taxonomy**	Microcystis aeruginosa (Kützing) Kützing, FT (1846)		
**Microphotograph 400×**	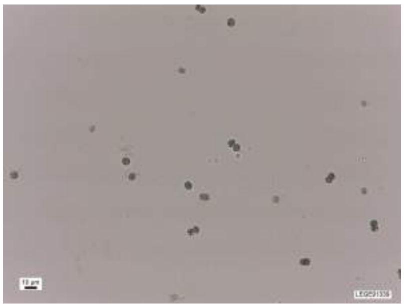	**Microphotograph 1000×**	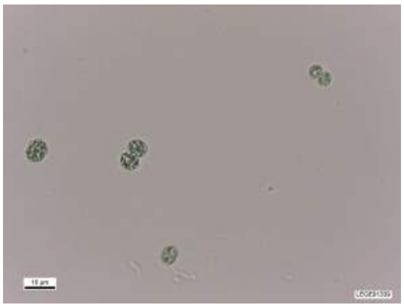
**Coidentity**	LEGE	**Morphometrics (Width × Length or Diameter) (µm)**	Ø 4,7±0,9
**Other Code(s)**	IZANCYA, IZANCYA 5	**Taxonomic notes/diacritical features**	
**Parent Sample**		**Macroscopic growth features**	color: cyano; growing homogeneously, but also forming aggregates
**Isolation Date**		**Environment**	aquatic, freshwater
**Order (Older Classification Scheme)**	Chroococcales	**Habitat Sample Description**	pond, water sample
**Order 2014 (Modern Taxonomy)**	Chroococcales	**Ecophysiological traits**	toxin production
**Taxonomy Notes**		**Notes on ecophysiological traits**	MC producing strain (MC-LR, MC-LA, MC-AR)
**Accession Number(s)_16S**	KC311962	**Isolator**	Vitor Vasconcelos
**Accession Number(s)_others (product/gene /region)**	AJ515456 (microcystin synthetase, mcyA), AY382533 (microcystin synthetase, mcyE), FN667620 (microviridin precursor, mdnA), FN668551 (microviridin , mdnB), KC311989 (16S-23S rRNA ITS), KC312016 (DNA gyrase subunit ß, gyrB), KC312043 (cell division protein, ftsZ)	**Medium**	Z8
**Collector**	Vitor Vasconcelos	**Strain Status**	non-axenic, unicyanobacterial
**Collection Date**	1991/06/10	**Preservation type**	subculturing
**Location**	Portugal: Lagoa de Mira	**Light:dark cycle**	12:12-h
**Latitude & Longitude**	unknown	**Temperature (ºC)**	19
		**Light intensity**	10–30 µmol photons m−2 s−1
**References Publicly Available Articles**	Hisbergues et al. (2003) Arch. Microbiol. 180 (6), 402-410; Rantala et al. (2004) Proc. Natl. Acad. Sci. U.S.A. 101 (2), 568-573 (Table S1); Allender et al. (2009) Appl. Environ. Microbiol. 75(11), 3598-3604; Pereira et al. (2009) Bull. Environ. Contam. Toxicol. 83(1), 81-84; Ziemert et al. (2010) Appl. Environ. Microbiol. 76 (11), 3568-3574		
**References Publicly Available Theses**	Vasconcelos V.M. (1995) PhD Thesis, FCUP, University of Porto, Porto, Portugal; Ziemert N. (2009) PhD Thesis, Faculty of Mathematics and Natural Sciences, Humboldt University of Berlin, Berlin, Germany		

**Strain ID**	**Microcystis aeruginosa LEGE 91341**		
**Strain Taxonomy**	Microcystis aeruginosa (Kützing) Kützing, FT (1846)		
**Microphotograph 400×**	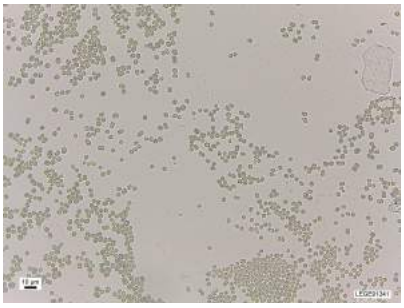	**Microphotograph 1000×**	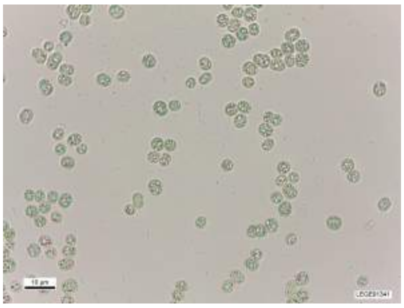
**Coidentity**	n.a.	**Morphometrics (Width × Length or Diameter) (µm)**	Ø 3,9±0,4
**Other Code(s)**	IZANCYA 12	**Taxonomic notes/diacritical features**	
**Parent Sample**		**Macroscopic growth features**	color: cyano; growing homogeneously, but also forming smooth biofilm
**Isolation Date**		**Environment**	aquatic, freshwater
**Order (Older Classification Scheme)**	Chroococcales	**Habitat Sample Description**	pond, water sample
**Order 2014 (Modern Taxonomy)**	Chroococcales	**Ecophysiological traits**	
**Taxonomy Notes**		**Notes on ecophysiological traits**	non-MC producing strain
**Accession Number(s)_16S**	KC311963	**Isolator**	Vitor Vasconcelos
**Accession Number(s)_others (product/gene /region)**	KC311990 (16S-23S rRNA ITS), KC312017 (DNA gyrase subunit ß, gyrB), KC312044 (cell division protein, ftsZ)	**Medium**	Z8
**Collector**	Vitor Vasconcelos	**Strain Status**	non-axenic, unicyanobacterial
**Collection Date**	1991/07/28	**Preservation type**	subculturing
**Location**	Portugal: Lagoa das Braças, Quiaios	**Light:dark cycle**	12:12-h
**Latitude & Longitude**	unknown	**Temperature (ºC)**	19
		**Light intensity**	10–30 µmol photons m−2 s−1
**References Publicly Available Articles**	Fraga et al. (2014) Anal. Chim. Acta 850, 57-64; Moreira et al. (2014) Curr. Microbiol. 69, 628–633		
**References Publicly Available Theses**	Vasconcelos V.M. (1995) PhD Thesis, FCUP, University of Porto, Porto, Portugal		

Strain ID	**Microcystis aeruginosa LEGE 91342**		
**Strain Taxonomy**	Microcystis aeruginosa (Kützing) Kützing, FT (1846)		
**Microphotograph 400×**	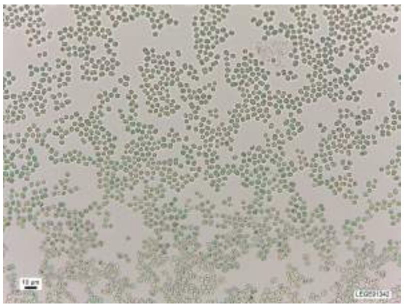	**Microphotograph 1000×**	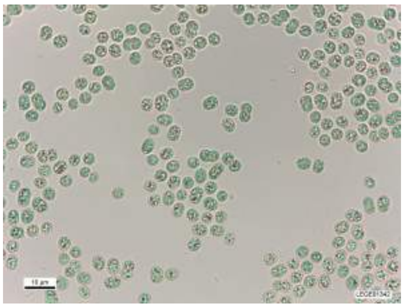
**Coidentity**	n.a.	**Morphometrics (Width × Length or Diameter) (µm)**	Ø 3,9±0,3
**Other Code(s)**	IZANCYA 14	**Taxonomic notes/diacritical features**	
**Parent Sample**		**Macroscopic growth features**	color: cyano; growing homogeneously
**Isolation Date**		**Environment**	aquatic, freshwater
**Order (Older Classification Scheme)**	Chroococcales	**Habitat Sample Description**	water sample
**Order 2014 (Modern Taxonomy)**	Chroococcales	**Ecophysiological traits**	
**Taxonomy Notes**		**Notes on ecophysiological traits**	non-MC producing strain
**Accession Number(s)_16S**	KC311959	**Isolator**	Vitor Vasconcelos
**Accession Number(s)_others (product/gene /region)**	KC311986 (16S-23S rRNA ITS), KC312013 (DNA gyrase subunit ß, gyrB), KC312040 (cell division protein, ftsZ)	**Medium**	Z8
**Collector**	Vitor Vasconcelos	**Strain Status**	non-axenic, unicyanobacterial
**Collection Date**	1991	**Preservation type**	subculturing, cryopreserved
**Location**	Portugal: Vilar dam reservoir, Távora river	**Light:dark cycle**	12:12-h
**Latitude & Longitude**	unknown	**Temperature (ºC)**	19
		**Light intensity**	10–30 µmol photons m−2 s−1
**References Publicly Available Articles**	Moreira et al. (2014) Curr. Microbiol. 69, 628–633		
**References Publicly Available Theses**	Vasconcelos V.M. (1995) PhD Thesis, FCUP, University of Porto, Porto, Portugal		

Strain ID	**Microcystis aeruginosa LEGE 91343**		
**Strain Taxonomy**	Microcystis aeruginosa (Kützing) Kützing, FT (1846)		
**Microphotograph 400×**	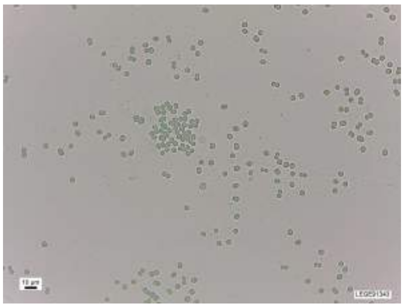	**Microphotograph 1000×**	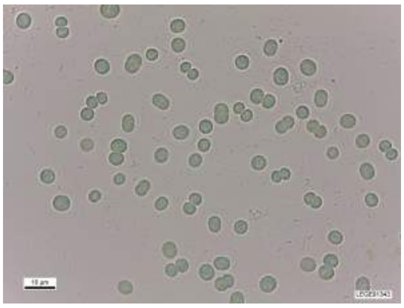
**Coidentity**	n.a.	**Morphometrics (Width × Length or Diameter) (µm)**	Ø 4,3±0,5
**Other Code(s)**	IZANCYA 21	**Taxonomic notes/diacritical features**	
**Parent Sample**		**Macroscopic growth features**	color: cyano; growing homogeneously; mucilaginous
**Isolation Date**		**Environment**	aquatic, freshwater
**Order (Older Classification Scheme)**	Chroococcales	**Habitat Sample Description**	water sample
**Order 2014 (Modern Taxonomy)**	Chroococcales	**Ecophysiological traits**	EPS production
**Taxonomy Notes**		**Notes on ecophysiological traits**	
**Accession Number(s)_16S**	KF287005	**Isolator**	Vitor Vasconcelos
**Accession Number(s)_others (product/gene /region)**	KF287030 (16S-23S rRNA ITS), KF287055 (DNA gyrase subunit ß, gyrB), KF287080 (cell division protein, ftsZ)	**Medium**	Z8
**Collector**	Vitor Vasconcelos	**Strain Status**	non-axenic, unicyanobacterial
**Collection Date**	1991	**Preservation type**	subculturing
**Location**	Portugal	**Light:dark cycle**	12:12-h
**Latitude & Longitude**	unknown	**Temperature (ºC)**	19
		**Light intensity**	10–30 µmol photons m−2 s−1
**References Publicly Available Articles**	Moreira et al. (2014) Curr. Microbiol. 69, 628–633		
**References Publicly Available Theses**			

Strain ID	**Microcystis aeruginosa LEGE 91344**		
**Strain Taxonomy**	Microcystis aeruginosa (Kützing) Kützing, FT (1846)		
**Microphotograph 400×**	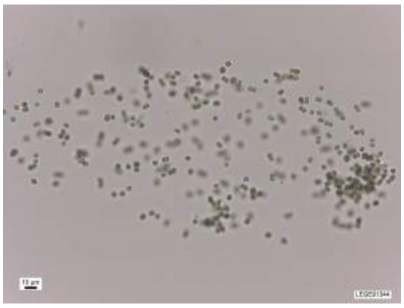	**Microphotograph 1000×**	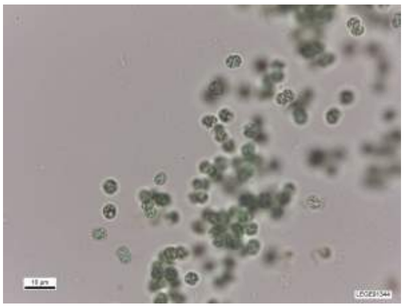
**Coidentity**	n.a.	**Morphometrics (Width × Length or Diameter) (µm)**	Ø 4,5±0,1
**Other Code(s)**	IZANCYA 22 , IZ22	**Taxonomic notes/diacritical features**	mucilaginous colonies
**Parent Sample**		**Macroscopic growth features**	color: cyano; forming aggregates
**Isolation Date**		**Environment**	aquatic, freshwater
**Order (Older Classification Scheme)**	Chroococcales	**Habitat Sample Description**	pond
**Order 2014 (Modern Taxonomy)**	Chroococcales	**Ecophysiological traits**	EPS production
**Taxonomy Notes**		**Notes on ecophysiological traits**	non-MC producing strain
**Accession Number(s)_16S**	KC311964	**Isolator**	Vitor Vasconcelos
**Accession Number(s)_others (product/gene /region)**	KC311991 (16S-23S rRNA ITS), KC312018 (DNA gyrase subunit ß, gyrB), KC312045 (cell division protein, ftsZ)	**Medium**	Z8
**Collector**	Vitor Vasconcelos	**Strain Status**	non-axenic, unicyanobacterial
**Collection Date**	1991/09/26	**Preservation type**	subculturing
**Location**	Portugal: Lagoa dos Teixoeiras, Tocha	**Light:dark cycle**	12:12-h
**Latitude & Longitude**	unknown	**Temperature (ºC)**	19
		**Light intensity**	10–30 µmol photons m−2 s−1
**References Publicly Available Articles**	Jokela et al. (2010) ChemBioChem 11, 1594-1599		
**References Publicly Available Theses**	Vasconcelos V.M. (1995) PhD Thesis, FCUP, University of Porto, Porto, Portugal		

Strain ID	**Microcystis aeruginosa LEGE 91347**		
**Strain Taxonomy**	Microcystis aeruginosa (Kützing) Kützing, FT (1846)		
**Microphotograph 400×**	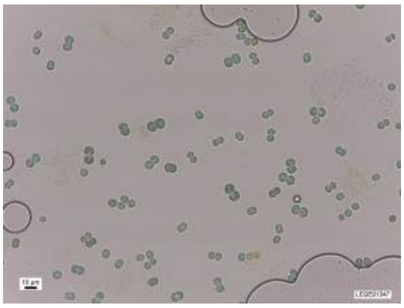	**Microphotograph 1000×**	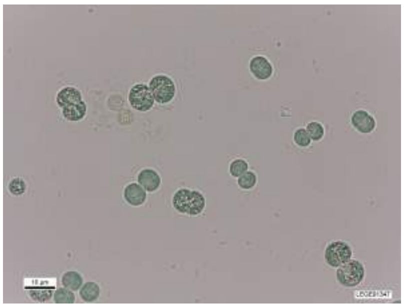
**Coidentity**	n.a.	**Morphometrics (Width × Length or Diameter) (µm)**	Ø 6,1±0,1
**Other Code(s)**	IZANCYA 31	**Taxonomic notes/diacritical features**	vacuolated cells
**Parent Sample**		**Macroscopic growth features**	color: cyano; growing homogeneously
**Isolation Date**		**Environment**	aquatic, freshwater
**Order (Older Classification Scheme)**	Chroococcales	**Habitat Sample Description**	water sample
**Order 2014 (Modern Taxonomy)**	Chroococcales	**Ecophysiological traits**	
**Taxonomy Notes**		**Notes on ecophysiological traits**	
**Accession Number(s)_16S**	KC311961	**Isolator**	Vitor Vasconcelos
**Accession Number(s)_others (product/gene /region)**	KF008262 (cyanobactin, N-terminal protease (A)), KC311988 (16S-23S rRNA ITS), KC312015 (DNA gyrase subunit ß, gyrB), KC312042 (cell division protein, ftsZ)	**Medium**	Z8
**Collector**	Vitor Vasconcelos	**Strain Status**	non-axenic, unicyanobacterial
**Collection Date**	Sep-1991	**Preservation type**	subculturing
**Location**	Portugal: Bemposta dam reservoir, Douro river	**Light:dark cycle**	12:12-h
**Latitude & Longitude**	unknown	**Temperature (ºC)**	19
		**Light intensity**	10–30 µmol photons m−2 s−1
**References Publicly Available Articles**	Martins et al. (2013) Mar. Drugs 11 (12), 4902-4916		
**References Publicly Available Theses**	Vasconcelos V.M. (1995) PhD Thesis, FCUP, University of Porto, Porto, Portugal		

**Strain ID**	**Microcystis aeruginosa LEGE 91349**		
**Strain Taxonomy**	Microcystis aeruginosa (Kützing) Kützing, FT (1846)		
**Microphotograph 400×**	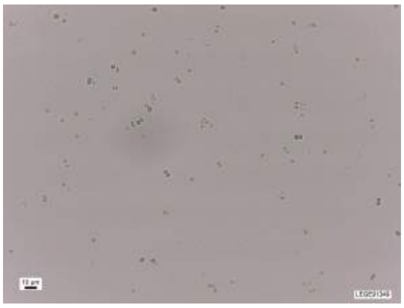	**Microphotograph 1000×**	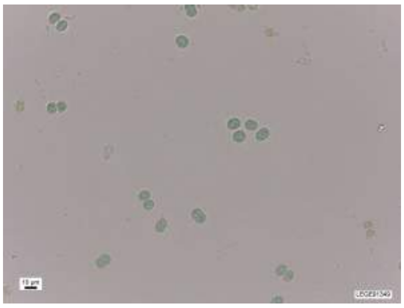
**Coidentity**	n.a.	**Morphometrics (Width × Length or Diameter) (µm)**	Ø 3,5±0,6
**Other Code(s)**	IZANCYA 34	**Taxonomic notes/diacritical features**	
**Parent Sample**		**Macroscopic growth features**	color: cyano; growing homogeneously; mucilaginous
**Isolation Date**		**Environment**	aquatic, freshwater
**Order (Older Classification Scheme)**	Chroococcales	**Habitat Sample Description**	water sample
**Order 2014 (Modern Taxonomy)**	Chroococcales	**Ecophysiological traits**	toxin production, EPS production
**Taxonomy Notes**		**Notes on ecophysiological traits**	MC producing strain (MC-LR, MC-LA)
**Accession Number(s)_16S**		**Isolator**	Vitor Vasconcelos
**Accession Number(s)_others (product/gene /region)**		**Medium**	Z8
**Collector**	Vitor Vasconcelos	**Strain Status**	non-axenic, unicyanobacterial
**Collection Date**	1991	**Preservation type**	subculturing
**Location**	Portugal	**Light:dark cycle**	12:12-h
**Latitude & Longitude**	unknown	**Temperature (ºC)**	19
		**Light intensity**	10–30 µmol photons m−2 s−1
**References Publicly Available Articles**			
**References Publicly Available Theses**	Vasconcelos V.M. (1995) PhD Thesis, FCUP, University of Porto, Porto, Portugal		

Strain ID	**Microcystis aeruginosa LEGE 91350**		
**Strain Taxonomy**	Microcystis aeruginosa (Kützing) Kützing, FT (1846)		
**Microphotograph 400×**	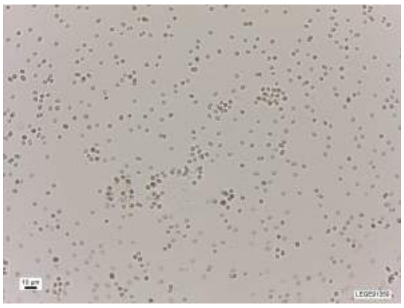	**Microphotograph 1000×**	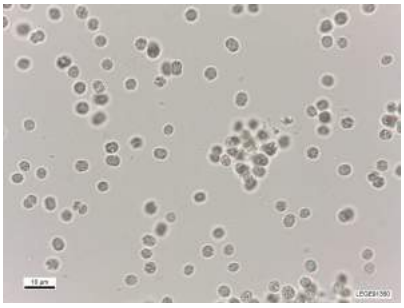
**Coidentity**	n.a.	**Morphometrics (Width × Length or Diameter) (µm)**	Ø 3,5±0,3
**Other Code(s)**	IZANCYA 36	**Taxonomic notes/diacritical features**	
**Parent Sample**		**Macroscopic growth features**	color: brownish; growing homogeneously, but also forming aggregates
**Isolation Date**		**Environment**	aquatic, freshwater
**Order (Older Classification Scheme)**	Chroococcales	**Habitat Sample Description**	pond
**Order 2014 (Modern Taxonomy)**	Chroococcales	**Ecophysiological traits**	
**Taxonomy Notes**		**Notes on ecophysiological traits**	non-MC producing strain
**Accession Number(s)_16S**		**Isolator**	Vitor Vasconcelos
**Accession Number(s)_others (product/gene /region)**		**Medium**	Z8
**Collector**	Vitor Vasconcelos	**Strain Status**	non-axenic, unicyanobacterial
**Collection Date**	1991/10/23	**Preservation type**	subculturing
**Location**	Portugal: Lagoa de Mira	**Light:dark cycle**	12:12-h
**Latitude & Longitude**	unknown	**Temperature (ºC)**	19
		**Light intensity**	10–30 µmol photons m−2 s−1
**References Publicly Available Articles**	Leikoski et al. (2012) PLoS One, 7(8), e43002		
**References Publicly Available Theses**	Vasconcelos V.M. (1995) PhD Thesis, FCUP, University of Porto, Porto, Portugal		

**Strain ID**	**Microcystis aeruginosa LEGE 91351**		
**Strain Taxonomy**	Microcystis aeruginosa (Kützing) Kützing, FT (1846)		
**Microphotograph 400×**	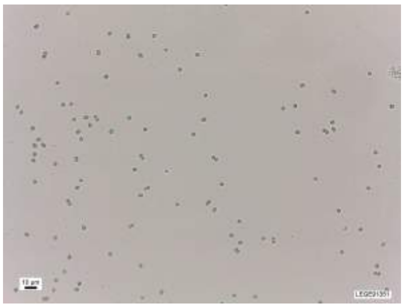	**Microphotograph 1000×**	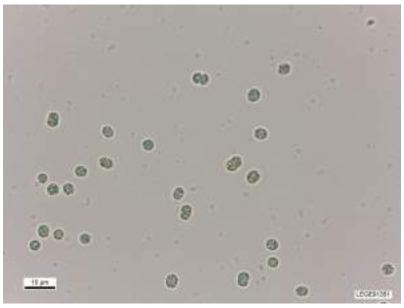
**Coidentity**	n.a.	**Morphometrics (Width × Length or Diameter) (µm)**	Ø 3,4±0,4
**Other Code(s)**	IZANCYA 41 , IZ41	**Taxonomic notes/diacritical features**	
**Parent Sample**		**Macroscopic growth features**	color: cyano; growing homogeneously; mucilaginous
**Isolation Date**		**Environment**	aquatic, freshwater
**Order (Older Classification Scheme)**	Chroococcales	**Habitat Sample Description**	pond
**Order 2014 (Modern Taxonomy)**	Chroococcales	**Ecophysiological traits**	EPS production
**Taxonomy Notes**		**Notes on ecophysiological traits**	non-MC producing strain
**Accession Number(s)_16S**	KC311966	**Isolator**	Vitor Vasconcelos
**Accession Number(s)_others (product/gene /region)**	KF008265 (cyanobactin, N-terminal protease (A)), KC311993 (16S-23S rRNA ITS), KC312020 (DNA gyrase subunit ß, gyrB), KC312047 (cell division protein, ftsZ)	**Medium**	Z8
**Collector**	Vitor Vasconcelos	**Strain Status**	non-axenic, unicyanobacterial
**Collection Date**	1991/11/15	**Preservation type**	subculturing
**Location**	Portugal: Lagoa das Braças, Quiaios	**Light:dark cycle**	12:12-h
**Latitude & Longitude**	unknown	**Temperature (ºC)**	19
		**Light intensity**	10–30 µmol photons m−2 s−1
**References Publicly Available Articles**	Pereira et al. (2009) Bull. Environ. Contam. Toxicol. 83(1), 81-84; Jokela et al. (2010) ChemBioChem 11, 1594-1599; Leikoski et al. (2012) PLoS One, 7(8), e43002; Martins et al. (2013) Mar. Drugs 11 (12), 4902-4916		
**References Publicly Available Theses**	Vasconcelos V.M. (1995) PhD Thesis, FCUP, University of Porto, Porto, Portugal; de Matos A. (2014) MSc Dissertation, FCUP, University of Porto, Porto, Portugal		

**Strain ID**	**Microcystis aeruginosa LEGE 91352**		
**Strain Taxonomy**	Microcystis aeruginosa (Kützing) Kützing, FT (1846)		
**Microphotograph 400×**	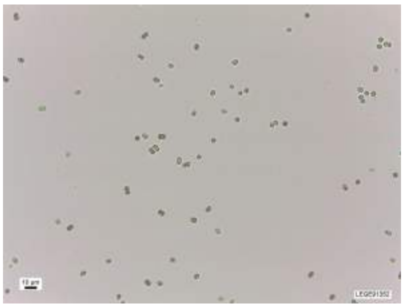	**Microphotograph 1000×**	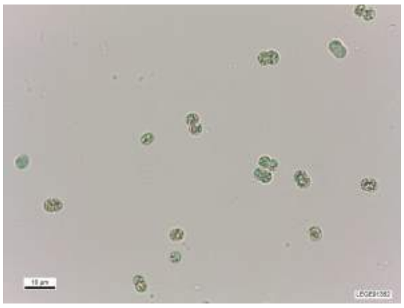
**Coidentity**	n.a.	**Morphometrics (Width × Length or Diameter) (µm)**	Ø 4,0±0,4
**Other Code(s)**	IZANCYA 42	**Taxonomic notes/diacritical features**	
**Parent Sample**		**Macroscopic growth features**	color: cyano; growing homogeneously
**Isolation Date**		**Environment**	aquatic, freshwater
**Order (Older Classification Scheme)**	Chroococcales	**Habitat Sample Description**	pond
**Order 2014 (Modern Taxonomy)**	Chroococcales	**Ecophysiological traits**	
**Taxonomy Notes**		**Notes on ecophysiological traits**	non-MC producing strain
**Accession Number(s)_16S**	KC311967	**Isolator**	Vitor Vasconcelos
**Accession Number(s)_others (product/gene /region)**	KC311994 (16S-23S rRNA ITS), KC312021 (DNA gyrase subunit ß, gyrB), KC312048 (cell division protein, ftsZ)	**Medium**	Z8
**Collector**	Vitor Vasconcelos	**Strain Status**	non-axenic, unicyanobacterial
**Collection Date**	1991/11/30	**Preservation type**	subculturing
**Location**	Portugal: Lagoa das Braças, Quiaios	**Light:dark cycle**	12:12-h
**Latitude & Longitude**	unknown	**Temperature (ºC)**	19
		**Light intensity**	10–30 µmol photons m−2 s−1
**References Publicly Available Articles**	Leikoski et al. (2012) PLoS One, 7(8), e43002		
**References Publicly Available Theses**	Vasconcelos V.M. (1995) PhD Thesis, FCUP, University of Porto, Porto, Portugal		

Strain ID	**Microcystis aeruginosa LEGE 91353**		
**Strain Taxonomy**	Microcystis aeruginosa (Kützing) Kützing, FT (1846)		
**Microphotograph 400×**	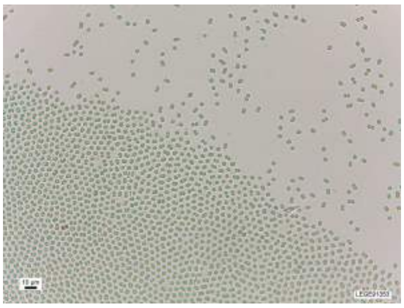	**Microphotograph 1000×**	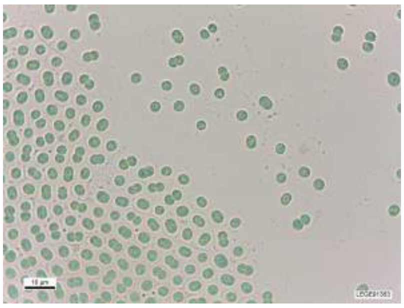
**Coidentity**	n.a.	**Morphometrics (Width × Length or Diameter) (µm)**	Ø 3,2±0,3
**Other Code(s)**	IZANCYA 43	**Taxonomic notes/diacritical features**	
**Parent Sample**		**Macroscopic growth features**	color: cyano; growing homogeneously; mucilaginous
**Isolation Date**		**Environment**	aquatic, freshwater
**Order (Older Classification Scheme)**	Chroococcales	**Habitat Sample Description**	pond
**Order 2014 (Modern Taxonomy)**	Chroococcales	**Ecophysiological traits**	EPS production
**Taxonomy Notes**		**Notes on ecophysiological traits**	non-MC producing strain
**Accession Number(s)_16S**		**Isolator**	Vitor Vasconcelos
**Accession Number(s)_others (product/gene /region)**		**Medium**	Z8
**Collector**	Vitor Vasconcelos	**Strain Status**	non-axenic, unicyanobacterial
**Collection Date**	1991/11/30	**Preservation type**	subculturing, cryopreserved
**Location**	Portugal: Lagoa das Braças, Quiaios	**Light:dark cycle**	12:12-h
**Latitude & Longitude**	unknown	**Temperature (ºC)**	19
		**Light intensity**	10–30 µmol photons m−2 s−1
**References Publicly Available Articles**			
**References Publicly Available Theses**	Vasconcelos V.M. (1995) PhD Thesis, FCUP, University of Porto, Porto, Portugal		

Strain ID	**Microcystis aeruginosa LEGE XX359**		
**Strain Taxonomy**	Microcystis aeruginosa (Kützing) Kützing, FT (1846)		
**Microphotograph 400×**	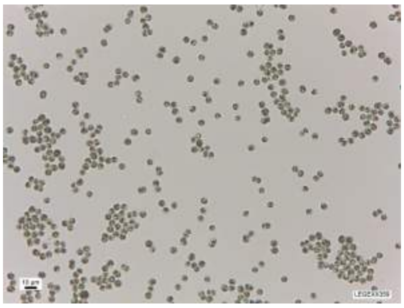	**Microphotograph 1000×**	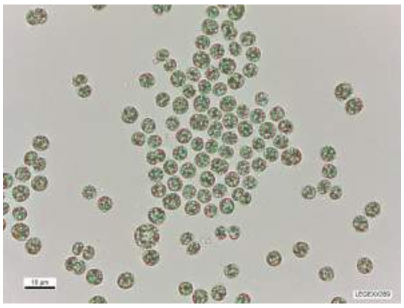
**Coidentity**	n.a.	**Morphometrics (Width × Length or Diameter) (µm)**	Ø 4,8±0,8
**Other Code(s)**	Marco 14	**Taxonomic notes/diacritical features**	
**Parent Sample**		**Macroscopic growth features**	color: cyano; growing homogeneously
**Isolation Date**		**Environment**	aquatic, freshwater
**Order (Older Classification Scheme)**	Chroococcales	**Habitat Sample Description**	water sample
**Order 2014 (Modern Taxonomy)**	Chroococcales	**Ecophysiological traits**	
**Taxonomy Notes**		**Notes on ecophysiological traits**	
**Accession Number(s)_16S**	KU951748	**Isolator**	Martin Saker
**Accession Number(s)_others (product/gene /region)**		**Medium**	Z8
**Collector**	Martin Saker	**Strain Status**	non-axenic, unicyanobacterial
**Collection Date**	unknown	**Preservation type**	subculturing
**Location**	Portugal: Marco de Canaveses	**Light:dark cycle**	12:12-h
**Latitude & Longitude**	unknown	**Temperature (ºC)**	19
		**Light intensity**	10–30 µmol photons m−2 s−1
**References Publicly Available Articles**			
**References Publicly Available Theses**			

Strain ID	**Microcystis sp. LEGE 00066**		
**Strain Taxonomy**	Microcystis Kützing, FT ex Lemmermann, E (1907)		
**Microphotograph 400×**	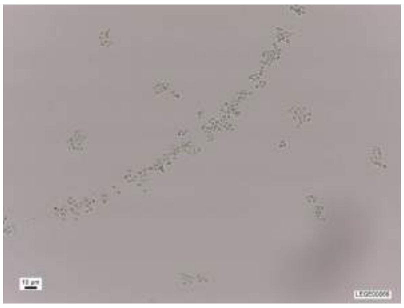	**Microphotograph 1000×**	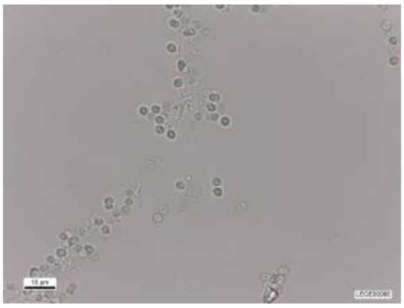
**Coidentity**	n.a.	**Morphometrics (Width × Length or Diameter) (µm)**	Ø 2,3±0,3
**Other Code(s)**		**Taxonomic notes/diacritical features**	
**Parent Sample**	Tak Mic OI	**Macroscopic growth features**	color: cyano; growing homogeneously
**Isolation Date**		**Environment**	aquatic, freshwater
**Order (Older Classification Scheme)**	Chroococcales	**Habitat Sample Description**	unknown
**Order 2014 (Modern Taxonomy)**	Chroococcales	**Ecophysiological traits**	
**Taxonomy Notes**		**Notes on ecophysiological traits**	
**Accession Number(s)_16S**	KU951749	**Isolator**	Martin Saker
**Accession Number(s)_others (product/gene /region)**		**Medium**	Z8
**Collector**	unknown	**Strain Status**	non-axenic, unicyanobacterial
**Collection Date**	2000	**Preservation type**	subculturing
**Location**	Morocco: Lake Takerkoust	**Light:dark cycle**	12:12-h
**Latitude & Longitude**	unknown	**Temperature (ºC)**	19
		**Light intensity**	10–30 µmol photons m−2 s−1
**References Publicly Available Articles**			
**References Publicly Available Theses**			

**Strain ID**	**Microcystis sp. LEGE 00258**		
**Strain Taxonomy**	Microcystis Kützing, FT ex Lemmermann, E (1907)		
**Microphotograph 400×**	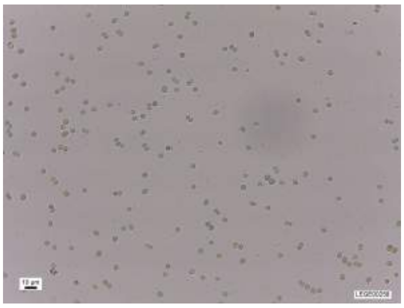	**Microphotograph 1000×**	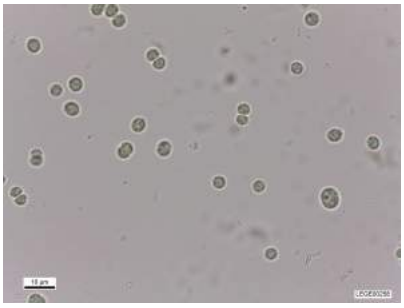
**Coidentity**	n.a.	**Morphometrics (Width × Length or Diameter) (µm)**	Ø 3,7±0,5
**Other Code(s)**	J44, LEANJ.44	**Taxonomic notes/diacritical features**	
**Parent Sample**		**Macroscopic growth features**	color: cyano; growing homogeneously
**Isolation Date**		**Environment**	aquatic, freshwater
**Order (Older Classification Scheme)**	Chroococcales	**Habitat Sample Description**	water sample
**Order 2014 (Modern Taxonomy)**	Chroococcales	**Ecophysiological traits**	
**Taxonomy Notes**		**Notes on ecophysiological traits**	
**Accession Number(s)_16S**	KU951750	**Isolator**	Joana Osswald
**Accession Number(s)_others (product/gene /region)**		**Medium**	Z8
**Collector**	Joana Oswald	**Strain Status**	non-axenic, unicyanobacterial
**Collection Date**	2000/08/17	**Preservation type**	subculturing
**Location**	Portugal: Marco de Canaveses	**Light:dark cycle**	12:12-h
**Latitude & Longitude**	unknown	**Temperature (ºC)**	19
		**Light intensity**	10–30 µmol photons m−2 s−1
**References Publicly Available Articles**			
**References Publicly Available Theses**			

**Strain ID**	**Microcystis sp. LEGE 08355**		
**Strain Taxonomy**	Microcystis Kützing, FT ex Lemmermann, E (1907)		
**Microphotograph 400×**	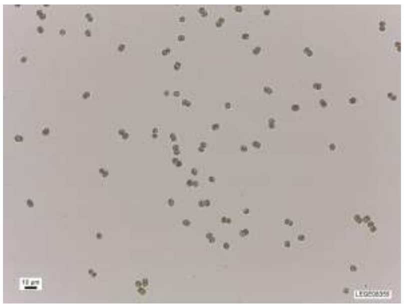	**Microphotograph 1000×**	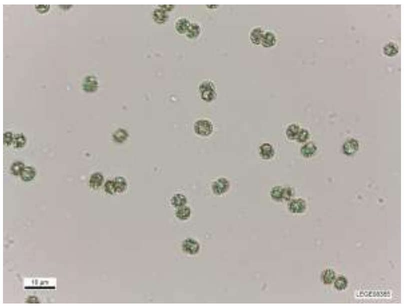
**Coidentity**	n.a.	**Morphometrics (Width × Length or Diameter) (µm)**	Ø 4,9±0,4
**Other Code(s)**	strain 16	**Taxonomic notes/diacritical features**	
**Parent Sample**	TMa 15EE	**Macroscopic growth features**	color: cyano; growing homogeneously, but also forming aggregates
**Isolation Date**		**Environment**	aquatic, freshwater
**Order (Older Classification Scheme)**	Chroococcales	**Habitat Sample Description**	water sample
**Order 2014 (Modern Taxonomy)**	Chroococcales	**Ecophysiological traits**	
**Taxonomy Notes**		**Notes on ecophysiological traits**	
**Accession Number(s)_16S**	KU951751	**Isolator**	Ana Regueiras
**Accession Number(s)_others (product/gene /region)**		**Medium**	Z8
**Collector**	Ana Regueiras	**Strain Status**	non-axenic, unicyanobacterial
**Collection Date**	2008/10/15	**Preservation type**	subculturing
**Location**	Portugal: Torrão reservoir	**Light:dark cycle**	12:12-h
**Latitude & Longitude**	unknown	**Temperature (ºC)**	19
		**Light intensity**	10–30 µmol photons m−2 s−1
**References Publicly Available Articles**			
**References Publicly Available Theses**	Regueiras A. (2009) MSc Dissertation, ICBAS and FCUP, University of Porto, Porto, Portugal		

Strain ID	**Microcystis sp. LEGE XX408**		
**Strain Taxonomy**	Microcystis Kützing, FT ex Lemmermann, E (1907)		
**Microphotograph 400×**	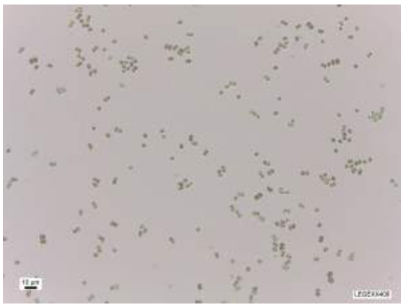	**Microphotograph 1000×**	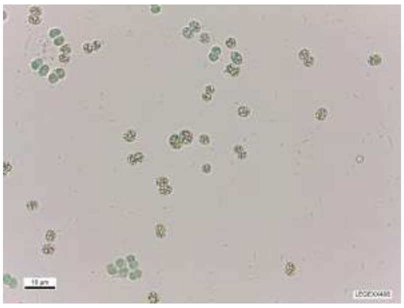
**Coidentity**	n.a.	**Morphometrics (Width × Length or Diameter) (µm)**	Ø 3,4±0,6
**Other Code(s)**	J87	**Taxonomic notes/diacritical features**	
**Parent Sample**		**Macroscopic growth features**	color: cyano; growing homogeneously
**Isolation Date**		**Environment**	aquatic, freshwater
**Order (Older Classification Scheme)**	Chroococcales	**Habitat Sample Description**	unknown
**Order 2014 (Modern Taxonomy)**	Chroococcales	**Ecophysiological traits**	
**Taxonomy Notes**		**Notes on ecophysiological traits**	
**Accession Number(s)_16S**	KU951752	**Isolator**	Joana Osswald
**Accession Number(s)_others (product/gene /region)**		**Medium**	Z8
**Collector**	Joana Osswald	**Strain Status**	non-axenic, unicyanobacterial
**Collection Date**	unknown	**Preservation type**	subculturing
**Location**	Portugal	**Light:dark cycle**	12:12-h
**Latitude & Longitude**	unknown	**Temperature (ºC)**	19
		**Light intensity**	10–30 µmol photons m−2 s−1
**References Publicly Available Articles**			
**References Publicly Available Theses**			

**Strain ID**	**Microcystis wesenbergii LEGE 08368**		
**Strain Taxonomy**	Microcystis wesenbergii (Komárek) Komárek, J ex Komárek, J in Kondrateva (1968)		
**Microphotograph 400×**	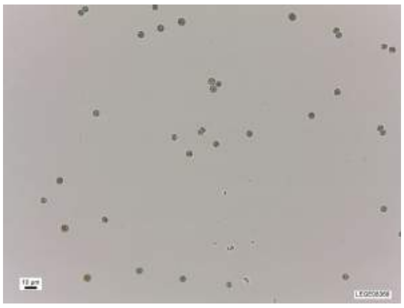	**Microphotograph 1000×**	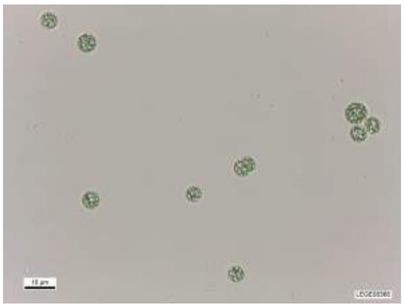
**Coidentity**	n.a.	**Morphometrics (Width × Length or Diameter) (µm)**	Ø 5,5±1,0
**Other Code(s)**		**Taxonomic notes/diacritical features**	
**Parent Sample**		**Macroscopic growth features**	color: cyano; growing homogeneously; mucilaginous
**Isolation Date**		**Environment**	aquatic, freshwater
**Order (Older Classification Scheme)**	Chroococcales	**Habitat Sample Description**	water sample
**Order 2014 (Modern Taxonomy)**	Chroococcales	**Ecophysiological traits**	EPS production
**Taxonomy Notes**		**Notes on ecophysiological traits**	
**Accession Number(s)_16S**	KU951753	**Isolator**	Ana Regueiras
**Accession Number(s)_others (product/gene /region)**		**Medium**	Z8
**Collector**	Ana Regueiras	**Strain Status**	non-axenic, unicyanobacterial
**Collection Date**	2008	**Preservation type**	subculturing
**Location**	Portugal	**Light:dark cycle**	12:12-h
**Latitude & Longitude**	unknown	**Temperature (ºC)**	19
		**Light intensity**	10–30 µmol photons m−2 s−1
**References Publicly Available Articles**			
**References Publicly Available Theses**			

**Strain ID**	**Myxosarcina sp. LEGE 06146**		
**Strain Taxonomy**	Myxosarcina Printz, H (1921)		
**Microphotograph 400×**	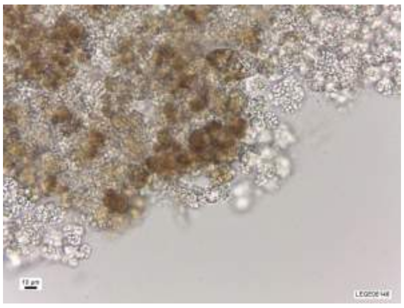	**Microphotograph 1000×**	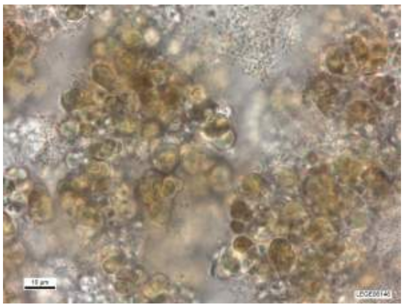
**Coidentity**	n.a.	**Morphometrics (Width × Length or Diameter) (µm)**	Ø 4,4±0,7 (more or less sperical cells); Sheath up to 0,9±0,2
**Other Code(s)**	LEAN 059	**Taxonomic notes/diacritical features**	with sheath
**Parent Sample**	O.06 MOL 10 - BBA/BD-B/C	**Macroscopic growth features**	color: brownish; forming colonies
**Isolation Date**		**Environment**	aquatic, marine
**Order (Older Classification Scheme)**	Pleurocapsales	**Habitat Sample Description**	wave-exposed tide pool, rock surface scraping
**Order 2014 (Modern Taxonomy)**	Pleurocapsales	**Ecophysiological traits**	
**Taxonomy Notes**		**Notes on ecophysiological traits**	
**Accession Number(s)_16S**	KU951754	**Isolator**	Vitor Ramos
**Accession Number(s)_others (product/gene /region)**	KC813170 (non-ribosomal peptide synthetase), KC842286-KC842288 (polyketide synthase)	**Medium**	MN and Z8 25‰ TM sea salt, w/ vit. B12
**Collector**	Rui Seabra	**Strain Status**	non-axenic, unicyanobacterial
**Collection Date**	2006/11/20	**Preservation type**	subculturing, cryopreserved
**Location**	Portugal: Praia de Moledo, Caminha	**Light:dark cycle**	14:10-h
**Latitude & Longitude**	41.84963 N 8.866717 W	**Temperature (ºC)**	25
		**Light intensity**	10–30 µmol photons m−2 s−1
**References Publicly Available Articles**	Brito et al. (2012) Syst. Appl. Microbiol. 35, 110–119; Brito et al. (2015) Algal Res. 9, 218-226		
**References Publicly Available Theses**			

**Strain ID**	**Nodosilinea (Leptolyngbya) antarctica LEGE 13457**		
**Strain Taxonomy**	Leptolyngbya antarctica (West & West, GS) Anagnostidis, K & Komárek, J (1988)		
**Microphotograph 400×**	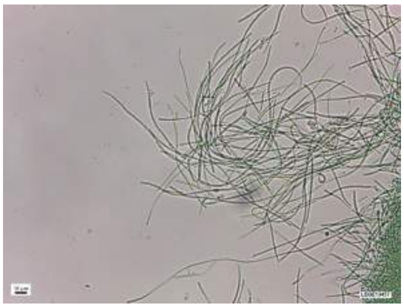	**Microphotograph 1000×**	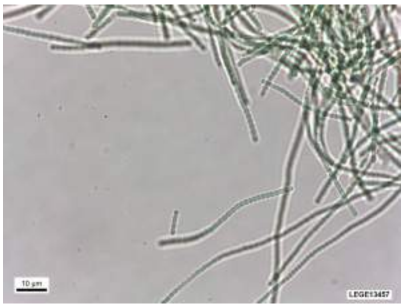
**Coidentity**	n.a.	**Morphometrics (Width × Length or Diameter) (µm)**	1,3±0,2 × 1,5±0,2
**Other Code(s)**		**Taxonomic notes/diacritical features**	trichomes constricted at cross-walls
**Parent Sample**	ANT#2.2 BZ	**Macroscopic growth features**	color: cyano; forming a smooth biofilm
**Isolation Date**		**Environment**	terrestrial
**Order (Older Classification Scheme)**	Oscillatoriales	**Habitat Sample Description**	polar, endolithic microbial community, on a sandstone
**Order 2014 (Modern Taxonomy)**	Synechococcales	**Ecophysiological traits**	
**Taxonomy Notes**		**Notes on ecophysiological traits**	dehydroabietic acid production (unknown ecophysiological role)
**Accession Number(s)_16S**	New Seq + KT951670	**Isolator**	Vitor Ramos
**Accession Number(s)_others (product/gene /region)**		**Medium**	Z8
**Collector**	Catarina Magalhães	**Strain Status**	non-axenic, unicyanobacterial
**Collection Date**	jan-2013	**Preservation type**	subculturing
**Location**	Antarctica: Victoria Valley, McMurdo Dry Valleys	**Light:dark cycle**	12:12-h
**Latitude & Longitude**	77.34183 S 161.6622 E	**Temperature (ºC)**	19
		**Light intensity**	10–30 µmol photons m−2 s−1
**References Publicly Available Articles**	Costa et al. (2016) Sci. Rep. 6, 23436		
**References Publicly Available Theses**			

Strain ID	**Nodosilinea (Leptolyngbya) antarctica LEGE 13458**		
**Strain Taxonomy**	Leptolyngbya antarctica (West & West, GS) Anagnostidis, K & Komárek, J (1988)		
**Microphotograph 400×**	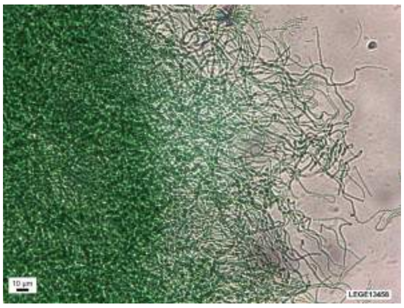	**Microphotograph 1000×**	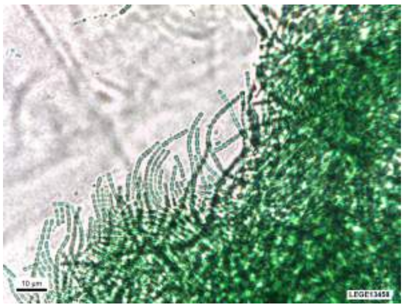
**Coidentity**	n.a.	**Morphometrics (Width × Length or Diameter) (µm)**	1,2±0,2 × 1,6±0,4
**Other Code(s)**		**Taxonomic notes/diacritical features**	trichomes constricted at cross-walls
**Parent Sample**	ANT#2.1 AA W3	**Macroscopic growth features**	color: cyano-greenish; forming a firm biofilm
**Isolation Date**		**Environment**	terrestrial
**Order (Older Classification Scheme)**	Oscillatoriales	**Habitat Sample Description**	polar, endolithic microbial community, on a sandstone
**Order 2014 (Modern Taxonomy)**	Synechococcales	**Ecophysiological traits**	
**Taxonomy Notes**		**Notes on ecophysiological traits**	
**Accession Number(s)_16S**	KU951755	**Isolator**	Vitor Ramos
**Accession Number(s)_others (product/gene /region)**		**Medium**	Z8
**Collector**	Catarina Magalhães	**Strain Status**	non-axenic, unicyanobacterial
**Collection Date**	jan-2013	**Preservation type**	subculturing
**Location**	Antarctica: Victoria Valley, McMurdo Dry Valleys	**Light:dark cycle**	12:12-h
**Latitude & Longitude**	77.34183 S 161.6622 E	**Temperature (ºC)**	19
		**Light intensity**	10–30 µmol photons m−2 s−1
**References Publicly Available Articles**			
**References Publicly Available Theses**			

Strain ID	**Nodosilinea cf. nodulosa LEGE 10377**		
**Strain Taxonomy**	Nodosilinea nodulosa (Li, Z & Brand, J) Perkerson, RB & Casamatta DA in Perkerson et al (2011)		
**Microphotograph 400×**	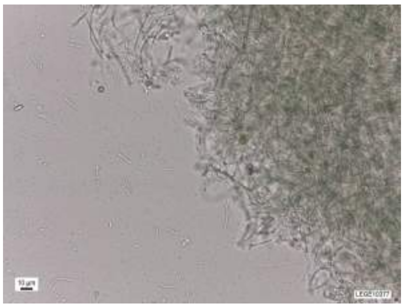	**Microphotograph 1000×**	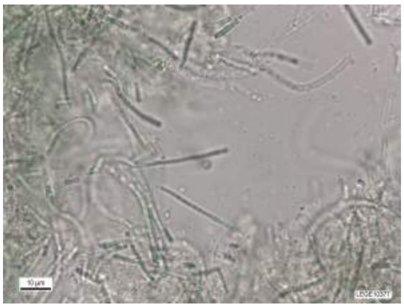
**Coidentity**	n.a.	**Morphometrics (Width × Length or Diameter) (µm)**	1,3±0,1 × 1,6±0,4
**Other Code(s)**	19E	**Taxonomic notes/diacritical features**	
**Parent Sample**		**Macroscopic growth features**	color: cyano; forming a smooth biofilm
**Isolation Date**		**Environment**	aquatic, marine
**Order (Older Classification Scheme)**	Oscillatoriales	**Habitat Sample Description**	intertidal zone, on a marine sponge
**Order 2014 (Modern Taxonomy)**	Synechococcales	**Ecophysiological traits**	
**Taxonomy Notes**		**Notes on ecophysiological traits**	
**Accession Number(s)_16S**	JQ927349	**Isolator**	Ana Regueiras
**Accession Number(s)_others (product/gene /region)**		**Medium**	Z8 25‰ TM sea salt w/ vit. B12
**Collector**	Ana Regueiras	**Strain Status**	non-axenic, unicyanobacterial, delicate
**Collection Date**	2010/11/24	**Preservation type**	subculturing
**Location**	Portugal: Praia da Aguda, Arcozelo	**Light:dark cycle**	12:12-h
**Latitude & Longitude**	41.04954 N 8.655339 W	**Temperature (ºC)**	19
		**Light intensity**	10–30 µmol photons m−2 s−1
**References Publicly Available Articles**			
**References Publicly Available Theses**	Vivas J. (2013) MSc Dissertation, FCUP, University of Porto, Porto, Portugal		

Strain ID	**Nodosilinea nodulosa LEGE 06101**		
**Strain Taxonomy**	Nodosilinea nodulosa (Li, Z & Brand, J) Perkerson, RB & Casamatta DA in Perkerson et al (2011)		
**Microphotograph 400×**	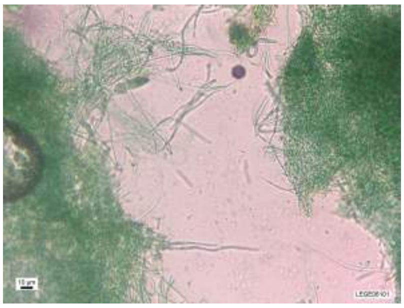	**Microphotograph 1000×**	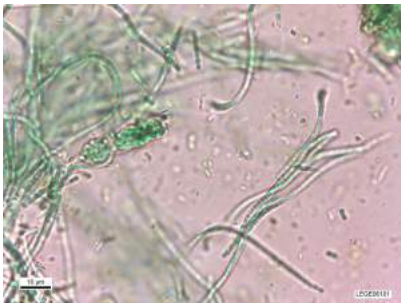
**Coidentity**	n.a.	**Morphometrics (Width × Length or Diameter) (µm)**	1,4±0,2 × 1,9±0,3
**Other Code(s)**	LEAN 005	**Taxonomic notes/diacritical features**	forming fascicles; nodules observed
**Parent Sample**	O.06 LUZ 3 - BC (I)	**Macroscopic growth features**	color: cyano; forming a smooth biofilm
**Isolation Date**		**Environment**	aquatic, marine
**Order (Older Classification Scheme)**	Oscillatoriales	**Habitat Sample Description**	intertidal zone, on a green macroalga
**Order 2014 (Modern Taxonomy)**	Synechococcales	**Ecophysiological traits**	
**Taxonomy Notes**		**Notes on ecophysiological traits**	
**Accession Number(s)_16S**	KU951756	**Isolator**	Vitor Ramos
**Accession Number(s)_others (product/gene /region)**		**Medium**	Z8 25‰ TM sea salt w/ vit. B12
**Collector**	Rui Seabra	**Strain Status**	non-axenic, unicyanobacterial
**Collection Date**	2006/12/12	**Preservation type**	subculturing
**Location**	Portugal: Praia da Luz, Lagos	**Light:dark cycle**	12:12-h
**Latitude & Longitude**	37.08622 N 8.725375 W	**Temperature (ºC)**	19
		**Light intensity**	10–30 µmol photons m−2 s−1
**References Publicly Available Articles**			
**References Publicly Available Theses**			

Strain ID	**Nodosilinea nodulosa LEGE 06102**		
**Strain Taxonomy**	Nodosilinea nodulosa (Li, Z & Brand, J) Perkerson, RB & Casamatta DA in Perkerson et al (2011)		
**Microphotograph 400×**	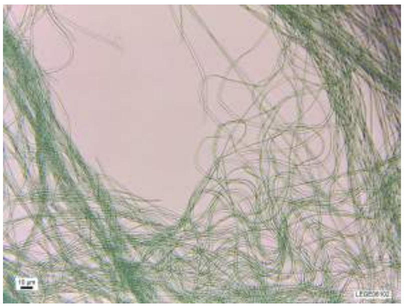	**Microphotograph 1000×**	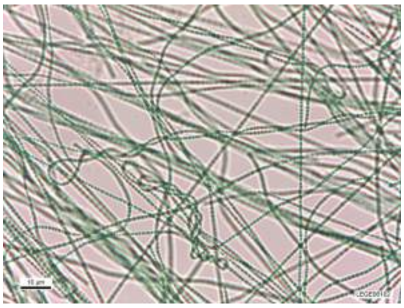
**Coidentity**	n.a.	**Morphometrics (Width × Length or Diameter) (µm)**	1,1±0,2 × 1,4±0,2
**Other Code(s)**	LEAN 006	**Taxonomic notes/diacritical features**	growing in circular fascicles; cell ultrastructure data available
**Parent Sample**	O.06 BAR 14 - BBAA	**Macroscopic growth features**	color: cyano; growing homogeneously; mucilaginous
**Isolation Date**		**Environment**	aquatic, marine
**Order (Older Classification Scheme)**	Oscillatoriales	**Habitat Sample Description**	tide pool, on a submerged stone
**Order 2014 (Modern Taxonomy)**	Synechococcales	**Ecophysiological traits**	EPS production
**Taxonomy Notes**		**Notes on ecophysiological traits**	bartolosides production (unknown ecophysiolgical role)
**Accession Number(s)_16S**	HQ832906	**Isolator**	Vitor Ramos
**Accession Number(s)_others (product/gene /region)**	KC842304, KC842305 (polyketide synthase)	**Medium**	Z8 25‰ TM sea salt w/ vit. B12
**Collector**	Rui Seabra/Vitor Ramos	**Strain Status**	non-axenic, unicyanobacterial
**Collection Date**	2006/11/21	**Preservation type**	subculturing, cryopreserved
**Location**	Portugal: Praia de São Bartolomeu do Mar, Esposende	**Light:dark cycle**	12:12-h
**Latitude & Longitude**	41.57377 N 8.798558 W	**Temperature (ºC)**	19
		**Light intensity**	10–30 µmol photons m−2 s−1
**References Publicly Available Articles**	Brito et al. (2012) Syst. Appl. Microbiol. 35, 110–119; Rastoll et al. (2013) J. Appl. Phycol. 25, 1483-1493; Costa et al. (2014) Mar. Drugs 12, 98-114; Brito et al. (2015) Algal Res. 9, 218-226; Leão et al. (2015) Angew. Chem. 127, 11215–11219; Costa et al. (2016) Sci. Rep. 6, 23436		
**References Publicly Available Theses**	Costa M.S. (2011) MSc Dissertation, ICBAS and FCUP, University of Porto, Porto, Portugal; Ribeiro M.J. (2012) MSc Dissertation, Department of Chemistry, University of Aveiro, Aveiro, Portugal		

**Strain ID**	**Nodosilinea nodulosa LEGE 06104**		
**Strain Taxonomy**	Nodosilinea nodulosa (Li, Z & Brand, J) Perkerson, RB & Casamatta DA in Perkerson et al (2011)		
**Microphotograph 400×**	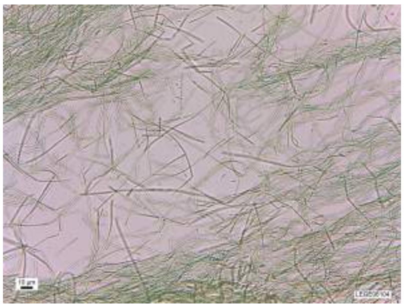	**Microphotograph 1000×**	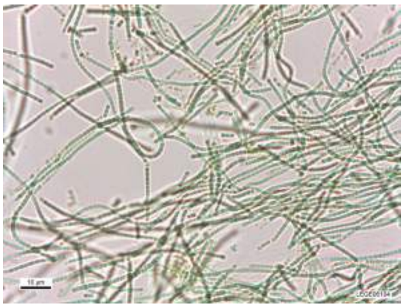
**Coidentity**	n.a.	**Morphometrics (Width × Length or Diameter) (µm)**	1,2±0,2 × 1,3±0,3
**Other Code(s)**	LEAN 008	**Taxonomic notes/diacritical features**	
**Parent Sample**	O.06 LUZ 10 - DC	**Macroscopic growth features**	color: cyano; forming a firm biofilm
**Isolation Date**		**Environment**	aquatic, marine
**Order (Older Classification Scheme)**	Oscillatoriales	**Habitat Sample Description**	tide puddle, rock surface scraping
**Order 2014 (Modern Taxonomy)**	Synechococcales	**Ecophysiological traits**	
**Taxonomy Notes**		**Notes on ecophysiological traits**	
**Accession Number(s)_16S**	KU569325	**Isolator**	Vitor Ramos
**Accession Number(s)_others (product/gene /region)**		**Medium**	Z8 25‰ TM sea salt w/ vit. B12
**Collector**	Rui Seabra	**Strain Status**	non-axenic, unicyanobacterial
**Collection Date**	2006/12/12	**Preservation type**	subculturing
**Location**	Portugal: Praia da Luz, Lagos	**Light:dark cycle**	12:12-h
**Latitude & Longitude**	37.08622 N 8.725375 W	**Temperature (ºC)**	19
		**Light intensity**	10–30 µmol photons m−2 s−1
**References Publicly Available Articles**	Costa et al. (2016) Sci. Rep. 6, 23436		
**References Publicly Available Theses**			

**Strain ID**	**Nodosilinea nodulosa LEGE 07084**		
**Strain Taxonomy**	Nodosilinea nodulosa (Li, Z & Brand, J) Perkerson, RB & Casamatta DA in Perkerson et al (2011)		
**Microphotograph 400×**	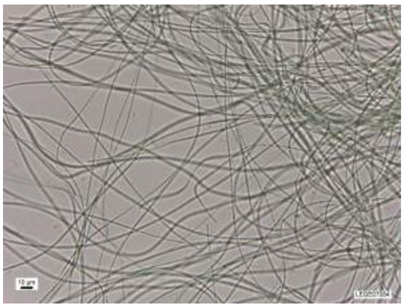	**Microphotograph 1000×**	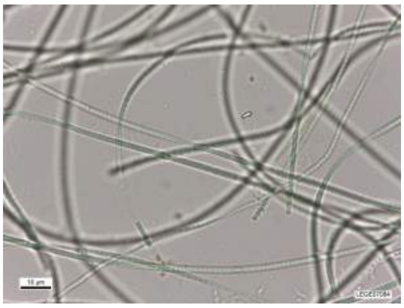
**Coidentity**	n.a.	**Morphometrics (Width × Length or Diameter) (µm)**	1,5±0,1 × 1,5±0,2
**Other Code(s)**		**Taxonomic notes/diacritical features**	with sheath
**Parent Sample**	VL 192	**Macroscopic growth features**	color: cyano; forming a firm biofilm
**Isolation Date**		**Environment**	aquatic, brackish
**Order (Older Classification Scheme)**	Oscillatoriales	**Habitat Sample Description**	mesotidal zone, benthic
**Order 2014 (Modern Taxonomy)**	Synechococcales	**Ecophysiological traits**	
**Taxonomy Notes**		**Notes on ecophysiological traits**	dehydroabietic acid and abietic acid production (unknown ecophysiological role)
**Accession Number(s)_16S**	HM217072	**Isolator**	Viviana Lopes
**Accession Number(s)_others (product/gene /region)**		**Medium**	Z8
**Collector**	Viviana Lopes	**Strain Status**	non-axenic, unicyanobacterial
**Collection Date**	Apr-2007	**Preservation type**	subculturing
**Location**	Portugal: Minho estuary, Caminha	**Light:dark cycle**	12:12-h
**Latitude & Longitude**	41.87933 N 8.838472 W	**Temperature (ºC)**	19
		**Light intensity**	10–30 µmol photons m−2 s−1
**References Publicly Available Articles**	Lopes et al. (2010) Mar. Drugs 8, 471-482; Baptista et al. (2011) Toxicon 58, 410–414; Lopes et al. (2011) Toxicol. In Vitro 25, 944–950; Lopes & Vasconcelos (2011) Mar. Drugs 9, 790-802; Cianca et al. (2012) Amino Acids 42, 2473–2479; Lopes et al. (2012) Mar. Environ. Res. 73, 7–16; Almeida et al. (2015) Toxins 7(8), 2739-2756; Costa et al. (2016) Sci. Rep. 6, 23436		
**References Publicly Available Theses**	Lopes V.R. (2011) PhD Thesis, FCUP, University of Porto, Porto, Portugal		

Strain ID	**Nodosilinea sp. LEGE 03283**		
**Strain Taxonomy**	Nodosilinea Perkerson, RB & Casamatta DA in Perkerson et al (2011)		
**Microphotograph 400×**	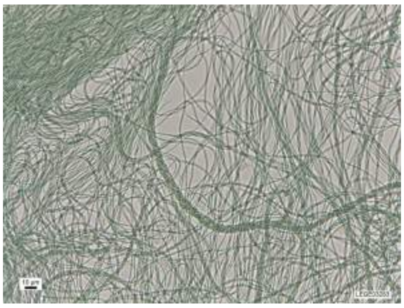	**Microphotograph 1000×**	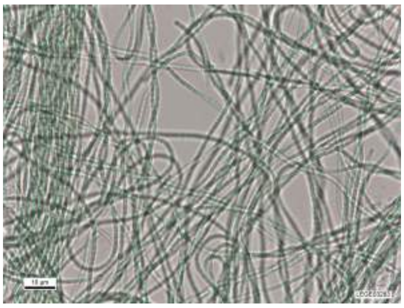
**Coidentity**	n.a.	**Morphometrics (Width × Length or Diameter) (µm)**	1,2±0,3 × 2,0±0,3
**Other Code(s)**	J77 X	**Taxonomic notes/diacritical features**	distinguishable centro- and chromatoplasm
**Parent Sample**		**Macroscopic growth features**	color: cyano; forming a smooth biofilm
**Isolation Date**		**Environment**	aquatic, freshwater
**Order (Older Classification Scheme)**	Oscillatoriales	**Habitat Sample Description**	water sample, dam reservoir
**Order 2014 (Modern Taxonomy)**	Synechococcales	**Ecophysiological traits**	
**Taxonomy Notes**		**Notes on ecophysiological traits**	
**Accession Number(s)_16S**	KU951757	**Isolator**	Joana Osswald
**Accession Number(s)_others (product/gene /region)**		**Medium**	Z8
**Collector**	Joana Oswald	**Strain Status**	non-axenic, unicyanobacterial
**Collection Date**	2003/10/02	**Preservation type**	subculturing
**Location**	Portugal: Maranhão dam reservoir, campsite at Montargil	**Light:dark cycle**	12:12-h
**Latitude & Longitude**	unknown	**Temperature (ºC)**	19
		**Light intensity**	10–30 µmol photons m−2 s−1
**References Publicly Available Articles**			
**References Publicly Available Theses**			

**Strain ID**	**Nodosilinea sp. LEGE 06001**		
**Strain Taxonomy**	Nodosilinea Perkerson, RB & Casamatta DA in Perkerson et al (2011)		
**Microphotograph 400×**	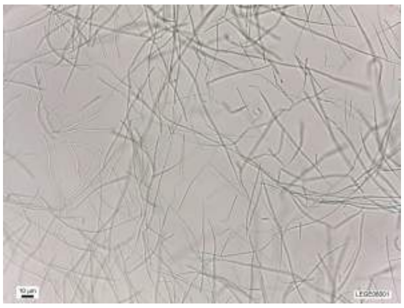	**Microphotograph 1000×**	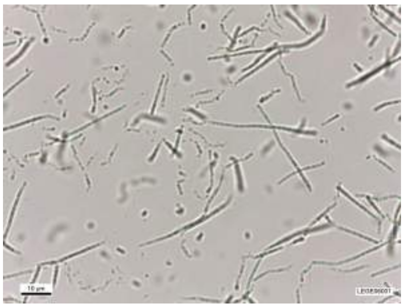
**Coidentity**	n.a.	**Morphometrics (Width × Length or Diameter) (µm)**	1,1±0,1 × 1,8±0,3
**Other Code(s)**	strain 20, LEAN 120	**Taxonomic notes/diacritical features**	distinguishable centro- and chromatoplasm
**Parent Sample**	Ref. 20, , planktonic	**Macroscopic growth features**	color: cyano; growing homogeneously
**Isolation Date**		**Environment**	aquatic, marine
**Order (Older Classification Scheme)**	Oscillatoriales	**Habitat Sample Description**	sea water sample, coastal, surf zone
**Order 2014 (Modern Taxonomy)**	Synechococcales	**Ecophysiological traits**	
**Taxonomy Notes**		**Notes on ecophysiological traits**	
**Accession Number(s)_16S**	KU951758	**Isolator**	Bárbara Frazão
**Accession Number(s)_others (product/gene /region)**		**Medium**	Z8 25‰ TM sea salt w/ vit. B12
**Collector**	Bárbara Frazão	**Strain Status**	non-axenic, unicyanobacterial
**Collection Date**	Fall 2006	**Preservation type**	subculturing
**Location**	Portugal: Praia de Buarcos	**Light:dark cycle**	12:12-h
**Latitude & Longitude**	40.15621 N 8.871803 W	**Temperature (ºC)**	19
		**Light intensity**	10–30 µmol photons m−2 s−1
**References Publicly Available Articles**			
**References Publicly Available Theses**			

Strain ID	**Nodosilinea sp. LEGE 06006**		
**Strain Taxonomy**	Nodosilinea Perkerson, RB & Casamatta DA in Perkerson et al (2011)		
**Microphotograph 400×**	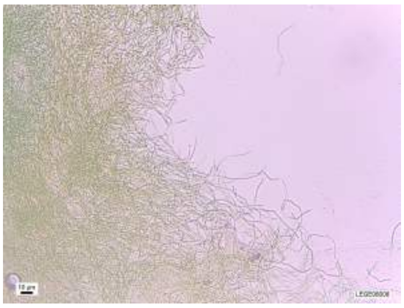	**Microphotograph 1000×**	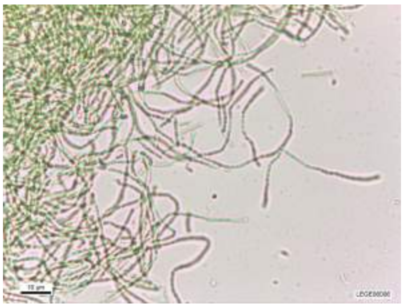
**Coidentity**	n.a.	**Morphometrics (Width × Length or Diameter) (µm)**	1,1±0,1 × 1,9±0,3
**Other Code(s)**	strain 1, LEAN 111	**Taxonomic notes/diacritical features**	
**Parent Sample**	Ref. 1, Moel planktonic	**Macroscopic growth features**	color: greenish; forming aggregates
**Isolation Date**		**Environment**	aquatic, marine
**Order (Older Classification Scheme)**	Oscillatoriales	**Habitat Sample Description**	sea water sample, coastal, surf zone
**Order 2014 (Modern Taxonomy)**	Synechococcales	**Ecophysiological traits**	
**Taxonomy Notes**		**Notes on ecophysiological traits**	
**Accession Number(s)_16S**	KU951759	**Isolator**	Bárbara Frazão
**Accession Number(s)_others (product/gene /region)**		**Medium**	Z8 25‰ TM sea salt w/ vit. B12
**Collector**	Bárbara Frazão	**Strain Status**	non-axenic, unicyanobacterial
**Collection Date**	Fall 2006	**Preservation type**	subculturing
**Location**	Portugal: São Pedro de Moel	**Light:dark cycle**	12:12-h
**Latitude & Longitude**	39.75529 N 9.033400 W	**Temperature (ºC)**	19
		**Light intensity**	10–30 µmol photons m−2 s−1
**References Publicly Available Articles**			
**References Publicly Available Theses**	Frazão B. (2008) MSc Dissertation, FCUP, University of Porto, Porto, Portugal		

Strain ID	**Nodosilinea sp. LEGE 06009**		
**Strain Taxonomy**	Nodosilinea Perkerson, RB & Casamatta DA in Perkerson et al (2011)		
**Microphotograph 400×**	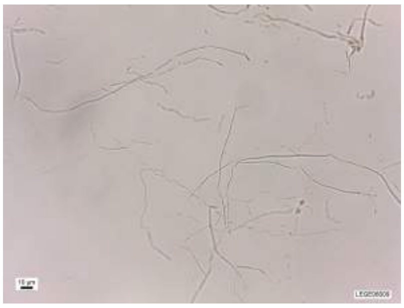	**Microphotograph 1000×**	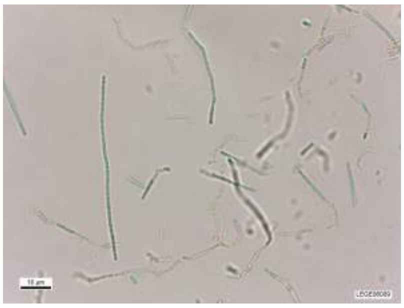
**Coidentity**	n.a.	**Morphometrics (Width × Length or Diameter) (µm)**	1,1±0,1 × 2,2±0,4
**Other Code(s)**	strain 16, LEAN 106	**Taxonomic notes/diacritical features**	
**Parent Sample**	Ref. 16	**Macroscopic growth features**	color: cyano; growing homogeneously; mucilaginous
**Isolation Date**		**Environment**	aquatic, marine
**Order (Older Classification Scheme)**	Oscillatoriales	**Habitat Sample Description**	intertidal zone, epilithic
**Order 2014 (Modern Taxonomy)**	Synechococcales	**Ecophysiological traits**	EPS production
**Taxonomy Notes**		**Notes on ecophysiological traits**	
**Accession Number(s)_16S**	JF708121	**Isolator**	Bárbara Frazão
**Accession Number(s)_others (product/gene /region)**	KC842331, KC842332 (polyketide synthase)	**Medium**	Z8 25‰ TM sea salt w/ vit. B12
**Collector**	Bárbara Frazão	**Strain Status**	non-axenic, unicyanobacterial
**Collection Date**	Fall 2006	**Preservation type**	subculturing
**Location**	Portugal: Praia da Foz do Arelho, Caldas da Rainha	**Light:dark cycle**	12:12-h
**Latitude & Longitude**	39.43327 N 9.230275 W	**Temperature (ºC)**	19
		**Light intensity**	10–30 µmol photons m−2 s−1
**References Publicly Available Articles**	Frazão et al. (2010) Mar. Drugs 8, 1908-1919; Brito et al. (2012) Syst. Appl. Microbiol. 35, 110–119; Rastoll et al. (2013) J. Appl. Phycol. 25, 1483-1493; Costa et al. (2014) Mar. Drugs 12, 98-114; Brito et al. (2015) Algal Res. 9, 218-226		
**References Publicly Available Theses**	Frazão B. (2008) MSc Dissertation, FCUP, University of Porto, Porto, Portugal		

Strain ID	**Nodosilinea sp. LEGE 06010**		
**Strain Taxonomy**	Nodosilinea Perkerson, RB & Casamatta DA in Perkerson et al (2011)		
**Microphotograph 400×**	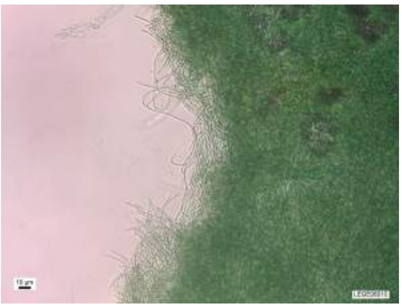	**Microphotograph 1000×**	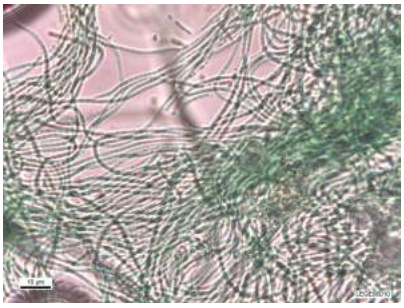
**Coidentity**	n.a.	**Morphometrics (Width × Length or Diameter) (µm)**	1,1±0,1 × 2,2±0,4
**Other Code(s)**	strain 17, LEAN 107	**Taxonomic notes/diacritical features**	
**Parent Sample**	Ref. 17	**Macroscopic growth features**	color: cyano; forming a smooth biofilm and aggregates
**Isolation Date**		**Environment**	aquatic, marine
**Order (Older Classification Scheme)**	Oscillatoriales	**Habitat Sample Description**	intertidal zone, wave-exposed rock
**Order 2014 (Modern Taxonomy)**	Synechococcales	**Ecophysiological traits**	
**Taxonomy Notes**		**Notes on ecophysiological traits**	
**Accession Number(s)_16S**	KU951760	**Isolator**	Bárbara Frazão
**Accession Number(s)_others (product/gene /region)**	HM124567 (microcystin synthetase, mcyE)	**Medium**	Z8 25‰ TM sea salt w/ vit. B12
**Collector**	Bárbara Frazão	**Strain Status**	non-axenic, unicyanobacterial
**Collection Date**	Fall 2006	**Preservation type**	subculturing
**Location**	Portugal: Praia da Foz do Arelho, Caldas da Rainha	**Light:dark cycle**	12:12-h
**Latitude & Longitude**	39.43327 N 9.230275 W	**Temperature (ºC)**	19
		**Light intensity**	10–30 µmol photons m−2 s−1
**References Publicly Available Articles**	Frazão et al. (2010) Mar. Drugs 8, 1908-1919		
**References Publicly Available Theses**	Frazão B. (2008) MSc Dissertation, FCUP, University of Porto, Porto, Portugal		

Strain ID	Nodosilinea sp. LEGE 06014		
**Strain Taxonomy**	Nodosilinea Perkerson, RB & Casamatta DA in Perkerson et al (2011)		
**Microphotograph 400×**	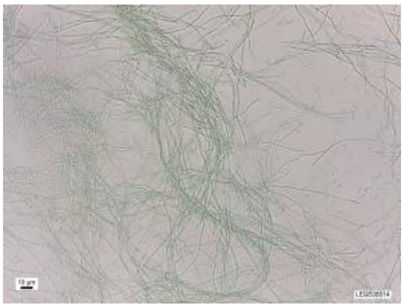	**Microphotograph 1000×**	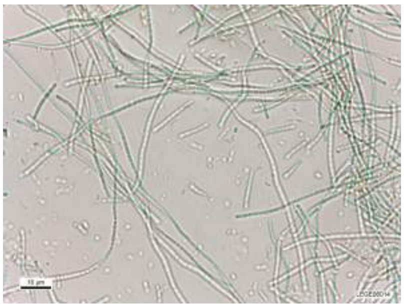
**Coidentity**	n.a.	**Morphometrics (Width × Length or Diameter) (µm)**	1,0±0,1 × 1,7±0,3
**Other Code(s)**	strain 19, LEAN 126	**Taxonomic notes/diacritical features**	
**Parent Sample**	Ref. 19, Arelho	**Macroscopic growth features**	color: cyano; growing homogeneously, but also forming aggregates; mucilaginous
**Isolation Date**		**Environment**	aquatic, marine
**Order (Older Classification Scheme)**	Oscillatoriales	**Habitat Sample Description**	intertidal zone, wave-exposed rock
**Order 2014 (Modern Taxonomy)**	Synechococcales	**Ecophysiological traits**	EPS production
**Taxonomy Notes**		**Notes on ecophysiological traits**	
**Accession Number(s)_16S**	KU951761	**Isolator**	Bárbara Frazão
**Accession Number(s)_others (product/gene /region)**		**Medium**	Z8 25‰ TM sea salt w/ vit. B12
**Collector**	Bárbara Frazão	**Strain Status**	non-axenic, unicyanobacterial
**Collection Date**	Fall 2006	**Preservation type**	subculturing
**Location**	Portugal: Praia da Foz do Arelho, Caldas da Rainha	**Light:dark cycle**	12:12-h
**Latitude & Longitude**	39.43327 N 9.230275 W	**Temperature (ºC)**	19
		**Light intensity**	10–30 µmol photons m−2 s−1
**References Publicly Available Articles**			
**References Publicly Available Theses**	Frazão B. (2008) MSc Dissertation, FCUP, University of Porto, Porto, Portugal		

**Strain ID**	**Nodosilinea sp. LEGE 06020**		
**Strain Taxonomy**	Nodosilinea Perkerson, RB & Casamatta DA in Perkerson et al (2011)		
**Microphotograph 400×**	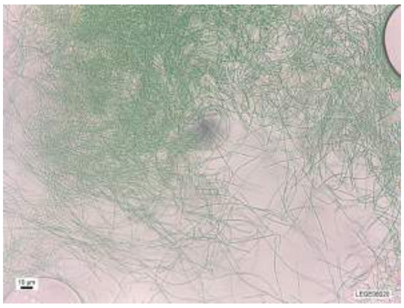	**Microphotograph 1000×**	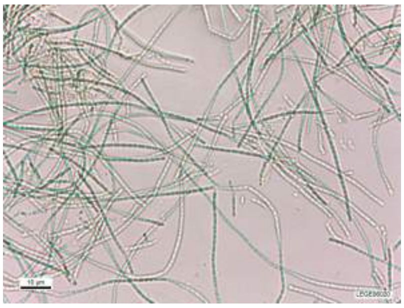
**Coidentity**	n.a.	**Morphometrics (Width × Length or Diameter) (µm)**	1,2±0,1 × 1,6±0,6
**Other Code(s)**	strain 8, LEAN 116	**Taxonomic notes/diacritical features**	
**Parent Sample**	Ref. 8, Coxos	**Macroscopic growth features**	color: cyano; forming a smooth biofilm and aggregates
**Isolation Date**		**Environment**	aquatic, marine
**Order (Older Classification Scheme)**	Oscillatoriales	**Habitat Sample Description**	sea water sample, coastal, surf zone
**Order 2014 (Modern Taxonomy)**	Synechococcales	**Ecophysiological traits**	
**Taxonomy Notes**		**Notes on ecophysiological traits**	
**Accession Number(s)_16S**	KU951762	**Isolator**	Bárbara Frazão
**Accession Number(s)_others (product/gene /region)**		**Medium**	Z8 25‰ TM sea salt w/ vit. B12
**Collector**	Bárbara Frazão	**Strain Status**	non-axenic, unicyanobacterial
**Collection Date**	Fall 2006	**Preservation type**	subculturing
**Location**	Portugal: Praia dos Coxos, Sto. Isidoro	**Light:dark cycle**	12:12-h
**Latitude & Longitude**	39.00455 N 9.425842 W	**Temperature (ºC)**	19
		**Light intensity**	10–30 µmol photons m−2 s−1
**References Publicly Available Articles**			
**References Publicly Available Theses**	Frazão B. (2008) MSc Dissertation, FCUP, University of Porto, Porto, Portugal; Macieira R. (2013) MSc Dissertation, FCUP, University of Porto, Porto, Portugal		

**Strain ID**	**Nodosilinea sp. LEGE 06022**		
**Strain Taxonomy**	Nodosilinea Perkerson, RB & Casamatta DA in Perkerson et al (2011)		
**Microphotograph 400×**	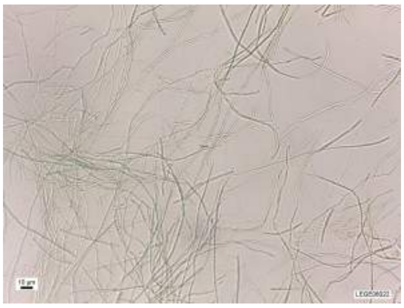	**Microphotograph 1000×**	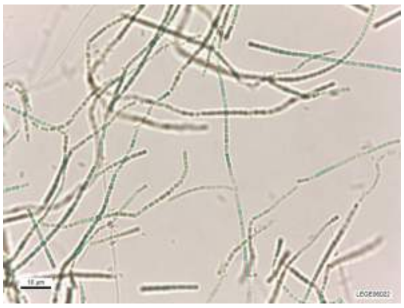
**Coidentity**	n.a.	**Morphometrics (Width × Length or Diameter) (µm)**	1,5±0,1 × 2,3±0,4
**Other Code(s)**	strain 21, LEAN 121	**Taxonomic notes/diacritical features**	
**Parent Sample**	Ref. 21, 3, Coxos	**Macroscopic growth features**	color: cyano; forming a smooth biofilm
**Isolation Date**		**Environment**	aquatic, marine
**Order (Older Classification Scheme)**	Oscillatoriales	**Habitat Sample Description**	intertidal zone, wave-sheltered rock
**Order 2014 (Modern Taxonomy)**	Synechococcales	**Ecophysiological traits**	
**Taxonomy Notes**		**Notes on ecophysiological traits**	
**Accession Number(s)_16S**	KU951763	**Isolator**	Bárbara Frazão
**Accession Number(s)_others (product/gene /region)**		**Medium**	Z8 25‰ TM sea salt w/ vit. B12
**Collector**	Bárbara Frazão	**Strain Status**	non-axenic, unicyanobacterial
**Collection Date**	Fall 2006	**Preservation type**	subculturing
**Location**	Portugal: Praia dos Coxos, Sto. Isidoro	**Light:dark cycle**	12:12-h
**Latitude & Longitude**	39.00455 N 9.425842 W	**Temperature (ºC)**	19
		**Light intensity**	10–30 µmol photons m−2 s−1
**References Publicly Available Articles**			
**References Publicly Available Theses**			

Strain ID	**Nodosilinea sp. LEGE 06069**		
**Strain Taxonomy**	Nodosilinea Perkerson, RB & Casamatta DA in Perkerson et al (2011)		
**Microphotograph 400×**	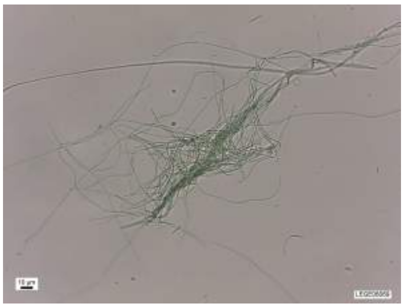	**Microphotograph 1000×**	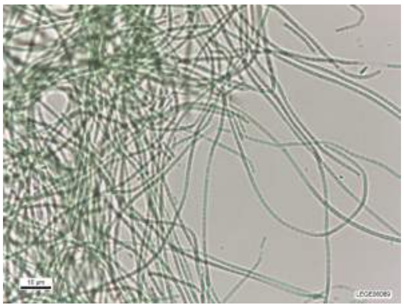
**Coidentity**	n.a.	**Morphometrics (Width × Length or Diameter) (µm)**	1,1±0,1 × 1,7±0,2
**Other Code(s)**		**Taxonomic notes/diacritical features**	with sheath
**Parent Sample**	VL 18	**Macroscopic growth features**	color: cyano; forming a firm biofilm
**Isolation Date**		**Environment**	aquatic, brackish
**Order (Older Classification Scheme)**	Oscillatoriales	**Habitat Sample Description**	mesotidal zone, benthic
**Order 2014 (Modern Taxonomy)**	Synechococcales	**Ecophysiological traits**	
**Taxonomy Notes**		**Notes on ecophysiological traits**	
**Accession Number(s)_16S**	HM217073	**Isolator**	Viviana Lopes
**Accession Number(s)_others (product/gene /region)**		**Medium**	Z8
**Collector**	Viviana Lopes	**Strain Status**	non-axenic, unicyanobacterial
**Collection Date**	Oct-2006	**Preservation type**	subculturing
**Location**	Portugal: Douro estuary, Porto	**Light:dark cycle**	12:12-h
**Latitude & Longitude**	41.14658 N 8.632556 W	**Temperature (ºC)**	19
		**Light intensity**	10–30 µmol photons m−2 s−1
**References Publicly Available Articles**	Lopes et al. (2010) Mar. Drugs 8, 471-482; Baptista et al. (2011) Toxicon 58, 410–414; Lopes & Vasconcelos (2011) Mar. Drugs 9, 790-802; Lopes et al. (2011) Toxicol. In Vitro 25, 944–950; Cianca et al. (2012) Amino Acids 42, 2473–2479; Lopes et al. (2012) Mar. Environ. Res. 73, 7–16; Rastoll et al. (2013) J. Appl. Phycol. 25, 1483-1493; Almeida et al. (2015) Toxins 7(8), 2739-2756		
**References Publicly Available Theses**	Lopes V.R. (2011) PhD Thesis, FCUP, University of Porto, Porto, Portugal		

**Strain ID**	**Nodosilinea sp. LEGE 06110**		
**Strain Taxonomy**	Nodosilinea Perkerson, RB & Casamatta DA in Perkerson et al (2011)		
**Microphotograph 400×**	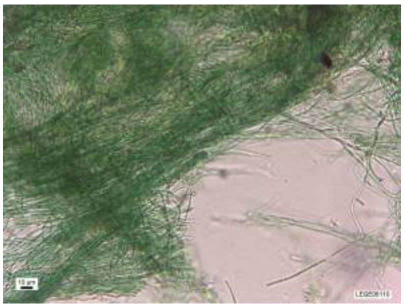	**Microphotograph 1000×**	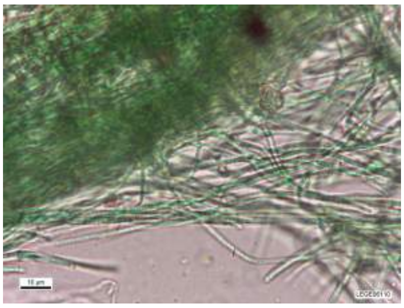
**Coidentity**	n.a.	**Morphometrics (Width × Length or Diameter) (µm)**	1,5±0,2 × 2,1±0,5
**Other Code(s)**	LEAN 015	**Taxonomic notes/diacritical features**	forming fascicles
**Parent Sample**	O.06 MOL 2 - AAD	**Macroscopic growth features**	color: cyano; forming a firm biofilm
**Isolation Date**		**Environment**	aquatic, marine
**Order (Older Classification Scheme)**	Oscillatoriales	**Habitat Sample Description**	tide puddle, on submerged stone
**Order 2014 (Modern Taxonomy)**	Synechococcales	**Ecophysiological traits**	
**Taxonomy Notes**		**Notes on ecophysiological traits**	
**Accession Number(s)_16S**	KU951764	**Isolator**	Vitor Ramos
**Accession Number(s)_others (product/gene /region)**	KC842285 (polyketide synthase)	**Medium**	Z8 25‰ TM sea salt w/ vit. B12
**Collector**	Rui Seabra/Vitor Ramos	**Strain Status**	non-axenic, unicyanobacterial
**Collection Date**	2006/11/20	**Preservation type**	subculturing
**Location**	Portugal: Praia de Moledo, Caminha	**Light:dark cycle**	12:12-h
**Latitude & Longitude**	41.84963 N 8.866717 W	**Temperature (ºC)**	19
		**Light intensity**	10–30 µmol photons m−2 s−1
**References Publicly Available Articles**	Brito et al. (2012) Syst. Appl. Microbiol. 35, 110–119; Brito et al. (2015) Algal Res. 9, 218-226; Costa et al. (2016) Sci. Rep. 6, 23436		
**References Publicly Available Theses**			

Strain ID	**Nodosilinea sp. LEGE 06115**		
**Strain Taxonomy**	Nodosilinea Perkerson, RB & Casamatta DA in Perkerson et al (2011)		
**Microphotograph 400×**	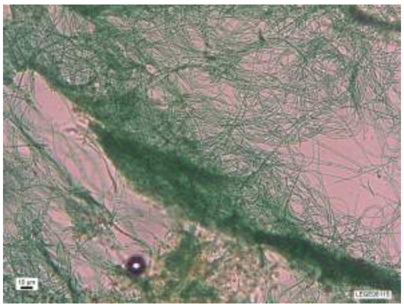	**Microphotograph 1000×**	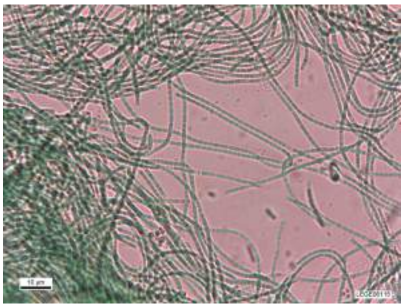
**Coidentity**	n.a.	**Morphometrics (Width × Length or Diameter) (µm)**	1,2±0,1 × 1,8±0,2
**Other Code(s)**	LEAN 020	**Taxonomic notes/diacritical features**	
**Parent Sample**	O.06 LUZ 3 - BCB	**Macroscopic growth features**	color: cyano; forming a firm biofilm
**Isolation Date**		**Environment**	aquatic, marine
**Order (Older Classification Scheme)**	Oscillatoriales	**Habitat Sample Description**	intertidal zone, on a green macroalga
**Order 2014 (Modern Taxonomy)**	Synechococcales	**Ecophysiological traits**	
**Taxonomy Notes**		**Notes on ecophysiological traits**	
**Accession Number(s)_16S**	KU951765	**Isolator**	Vitor Ramos
**Accession Number(s)_others (product/gene /region)**		**Medium**	Z8 25‰ TM sea salt w/ vit. B12
**Collector**	Rui Seabra	**Strain Status**	non-axenic, unicyanobacterial
**Collection Date**	2006/12/12	**Preservation type**	subculturing, cryopreserved
**Location**	Portugal: Praia da Luz, Lagos	**Light:dark cycle**	12:12-h
**Latitude & Longitude**	37.08622 N 8.725375 W	**Temperature (ºC)**	19
		**Light intensity**	10–30 µmol photons m−2 s−1
**References Publicly Available Articles**			
**References Publicly Available Theses**			

Strain ID	**Nodosilinea sp. LEGE 06119**		
**Strain Taxonomy**	Nodosilinea Perkerson, RB & Casamatta DA in Perkerson et al (2011)		
**Microphotograph 400×**	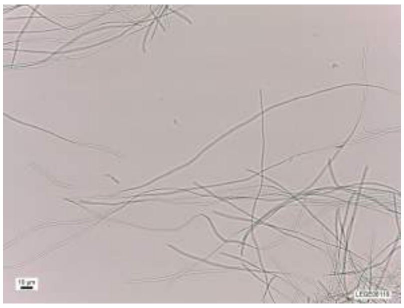	**Microphotograph 1000×**	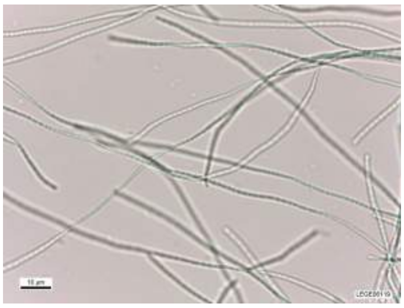
**Coidentity**	n.a.	**Morphometrics (Width × Length or Diameter) (µm)**	1,2±0,2 × 1,3±0,3
**Other Code(s)**	LEAN 024	**Taxonomic notes/diacritical features**	
**Parent Sample**	V.06 BUR p5 - AA	**Macroscopic growth features**	color: cyano; growing homogeneously; mucilaginous
**Isolation Date**		**Environment**	aquatic, marine
**Order (Older Classification Scheme)**	Oscillatoriales	**Habitat Sample Description**	sea water sample, coastal, surf zone
**Order 2014 (Modern Taxonomy)**	Synechococcales	**Ecophysiological traits**	EPS production
**Taxonomy Notes**		**Notes on ecophysiological traits**	
**Accession Number(s)_16S**	KU951766	**Isolator**	Vitor Ramos
**Accession Number(s)_others (product/gene /region)**		**Medium**	Z8 25‰ TM sea salt w/ vit. B12
**Collector**	Rui Seabra	**Strain Status**	non-axenic, unicyanobacterial
**Collection Date**	2006/07/17	**Preservation type**	subculturing
**Location**	Portugal: Praia de Burgau, Budens	**Light:dark cycle**	12:12-h
**Latitude & Longitude**	37.07156 N 8.774722 W	**Temperature (ºC)**	19
		**Light intensity**	10–30 µmol photons m−2 s−1
**References Publicly Available Articles**			
**References Publicly Available Theses**	Macieira R. (2013) MSc Dissertation, FCUP, University of Porto, Porto, Portugal		

**Strain ID**	**Nodosilinea sp. LEGE 06120**		
**Strain Taxonomy**	Nodosilinea Perkerson, RB & Casamatta DA in Perkerson et al (2011)		
**Microphotograph 400×**	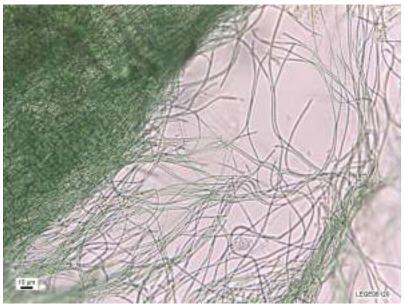	**Microphotograph 1000×**	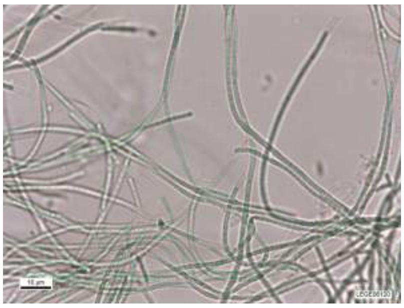
**Coidentity**	n.a.	**Morphometrics (Width × Length or Diameter) (µm)**	1,3±0,1 × 2,4±0,6
**Other Code(s)**	LEAN 025	**Taxonomic notes/diacritical features**	facultative sheath
**Parent Sample**	O.06 LUZ p5 - A	**Macroscopic growth features**	color: cyano; forming a firm biofilm
**Isolation Date**		**Environment**	aquatic, marine
**Order (Older Classification Scheme)**	Oscillatoriales	**Habitat Sample Description**	sea water sample, coastal, surf zone
**Order 2014 (Modern Taxonomy)**	Synechococcales	**Ecophysiological traits**	
**Taxonomy Notes**		**Notes on ecophysiological traits**	
**Accession Number(s)_16S**	KU951767	**Isolator**	Vitor Ramos
**Accession Number(s)_others (product/gene /region)**		**Medium**	Z8 25‰ TM sea salt w/ vit. B12
**Collector**	Rui Seabra	**Strain Status**	non-axenic, unicyanobacterial
**Collection Date**	2006/12/12	**Preservation type**	subculturing
**Location**	Portugal: Praia da Luz, Lagos	**Light:dark cycle**	12:12-h
**Latitude & Longitude**	37.08622 N 8.725375 W	**Temperature (ºC)**	19
		**Light intensity**	10–30 µmol photons m−2 s−1
**References Publicly Available Articles**			
**References Publicly Available Theses**	Macieira R. (2013) MSc Dissertation, FCUP, University of Porto, Porto, Portugal		

**Strain ID**	**Nodosilinea sp. LEGE 06121**		
**Strain Taxonomy**	Nodosilinea Perkerson, RB & Casamatta DA in Perkerson et al (2011)		
**Microphotograph 400×**	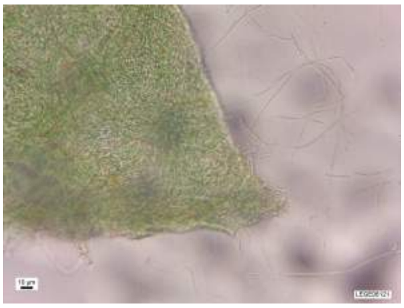	**Microphotograph 1000×**	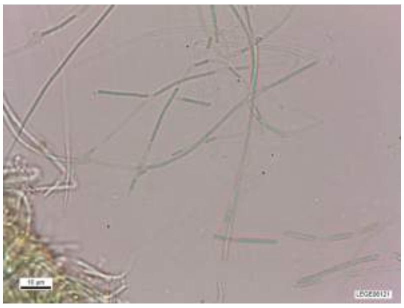
**Coidentity**	n.a.	**Morphometrics (Width × Length or Diameter) (μm)**	1,2±0,2 × 3,7±0,6
**Other Code(s)**	LEAN 027	**Taxonomic notes/diacritical features**	with sheath
**Parent Sample**	O.06 ODA 7 - CCA	**Macroscopic growth features**	color: cyano; forming a firm biofilm; mucilaginous
**Isolation Date**		**Environment**	aquatic, marine
**Order (Older Classification Scheme)**	Oscillatoriales	**Habitat Sample Description**	intertidal zone, wave-exposed rock, surface scraping
**Order 2014 (Modern Taxonomy)**	Synechococcales	**Ecophysiological traits**	EPS production
**Taxonomy Notes**		**Notes on ecophysiological traits**	
**Accession Number(s)_16S**	KU951768	**Isolator**	Vitor Ramos
**Accession Number(s)_others (product/gene /region)**	KC842362, KC842363 (polyketide synthase)	**Medium**	MN w/ vit. B12
**Collector**	Rui Seabra	**Strain Status**	non-axenic, unicyanobacterial
**Collection Date**	2006/12/12	**Preservation type**	subculturing
**Location**	Portugal: Praia de Olhos d'Água, Albufeira	**Light:dark cycle**	12:12-h
**Latitude & Longitude**	37.08973 N 8.191017 W	**Temperature (ºC)**	19
		**Light intensity**	10–30 µmol photons m−2 s−1
**References Publicly Available Articles**	Brito et al. (2012) Syst. Appl. Microbiol. 35, 110–119; Brito et al. (2015) Algal Res. 9, 218-226		
**References Publicly Available Theses**			

**Strain ID**	**Nodosilinea sp. LEGE 06124**		
**Strain Taxonomy**	Nodosilinea Perkerson, RB & Casamatta DA in Perkerson et al (2011)		
**Microphotograph 400×**	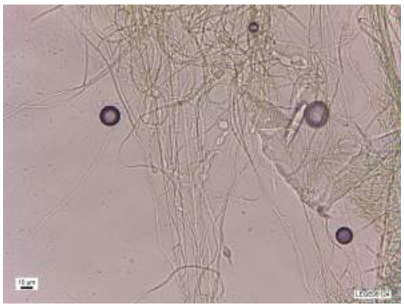	**Microphotograph 1000×**	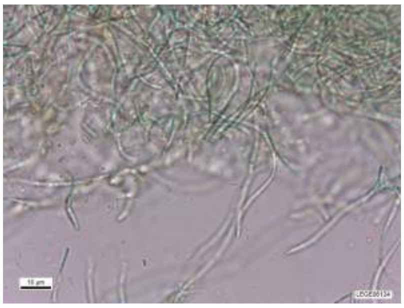
**Coidentity**	n.a.	**Morphometrics (Width × Length or Diameter) (µm)**	1,0±0,1 × 2,7±0,8
**Other Code(s)**	LEAN 029	**Taxonomic notes/diacritical features**	some helical coiled filaments and nodules; with sheath
**Parent Sample**	V.06 ODA 5 - BAA/BCA/ECA	**Macroscopic growth features**	color: cyano; forming a firm biofilm
**Isolation Date**		**Environment**	aquatic, marine
**Order (Older Classification Scheme)**	Oscillatoriales	**Habitat Sample Description**	wave-exposed tide puddle, epilithic
**Order 2014 (Modern Taxonomy)**	Synechococcales	**Ecophysiological traits**	
**Taxonomy Notes**		**Notes on ecophysiological traits**	
**Accession Number(s)_16S**	KU951769	**Isolator**	Vitor Ramos
**Accession Number(s)_others (product/gene /region)**		**Medium**	MN w/ vit. B12
**Collector**	Rui Seabra	**Strain Status**	non-axenic, unicyanobacterial
**Collection Date**	2006/12/12	**Preservation type**	subculturing
**Location**	Portugal: Praia de Olhos d'Água, Albufeira	**Light:dark cycle**	12:12-h
**Latitude & Longitude**	37.08973 N 8.191017 W	**Temperature (ºC)**	19
		**Light intensity**	10–30 µmol photons m−2 s−1
**References Publicly Available Articles**			
**References Publicly Available Theses**			

Strain ID	**Nodosilinea sp. LEGE 06129**		
**Strain Taxonomy**	Nodosilinea Perkerson, RB & Casamatta DA in Perkerson et al (2011)		
**Microphotograph 400×**	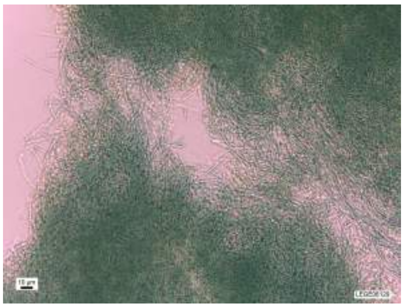	**Microphotograph 1000×**	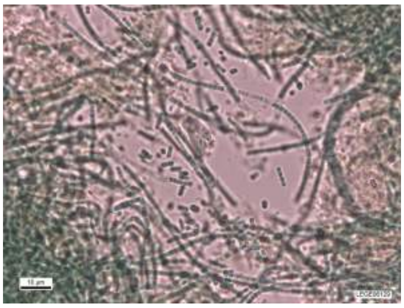
**Coidentity**	n.a.	**Morphometrics (Width × Length or Diameter) (µm)**	1,4±0,2 × 1,7±0,3
**Other Code(s)**	LEAN 037	**Taxonomic notes/diacritical features**	facultative sheath
**Parent Sample**	O.06 AGU p6 - AA/BA	**Macroscopic growth features**	color: cyano; forming a firm biofilm
**Isolation Date**		**Environment**	aquatic, marine
**Order (Older Classification Scheme)**	Oscillatoriales	**Habitat Sample Description**	sea water sample, coastal, surf zone
**Order 2014 (Modern Taxonomy)**	Synechococcales	**Ecophysiological traits**	
**Taxonomy Notes**		**Notes on ecophysiological traits**	
**Accession Number(s)_16S**	KU951770	**Isolator**	Vitor Ramos
**Accession Number(s)_others (product/gene /region)**		**Medium**	Z8 25‰ TM sea salt w/ vit. B12
**Collector**	Rui Seabra/Vitor Ramos	**Strain Status**	non-axenic, unicyanobacterial
**Collection Date**	2006/11/22	**Preservation type**	subculturing
**Location**	Portugal: Praia da Aguda, Arcozelo	**Light:dark cycle**	12:12-h
**Latitude & Longitude**	41.04954 N 8.655339 W	**Temperature (ºC)**	19
		**Light intensity**	10–30 µmol photons m−2 s−1
**References Publicly Available Articles**	Brito et al. (2012) Syst. Appl. Microbiol. 35, 110–119		
**References Publicly Available Theses**			

Strain ID	**Nodosilinea sp. LEGE 06133**		
**Strain Taxonomy**	Nodosilinea Perkerson, RB & Casamatta DA in Perkerson et al (2011)		
**Microphotograph 400×**	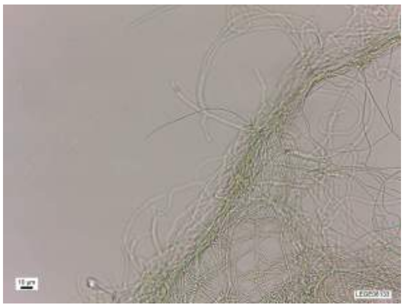	**Microphotograph 1000×**	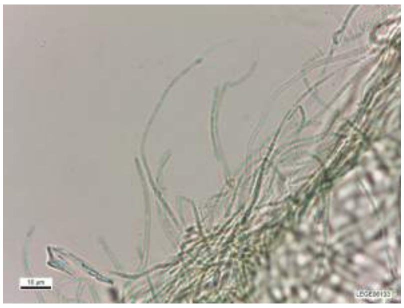
**Coidentity**	n.a.	**Morphometrics (Width × Length or Diameter) (µm)**	1,3±0,2 × 3,2±1,3
**Other Code(s)**	LEAN 042	**Taxonomic notes/diacritical features**	with sheath
**Parent Sample**	V.06 MOL 9 - A-B/C	**Macroscopic growth features**	color: cyano; forming a firm biofilm
**Isolation Date**		**Environment**	aquatic, marine
**Order (Older Classification Scheme)**	Oscillatoriales	**Habitat Sample Description**	tide pool, on a submerged stone
**Order 2014 (Modern Taxonomy)**	Synechococcales	**Ecophysiological traits**	
**Taxonomy Notes**		**Notes on ecophysiological traits**	
**Accession Number(s)_16S**	KU951771	**Isolator**	Vitor Ramos
**Accession Number(s)_others (product/gene /region)**		**Medium**	MN w/ vit. B12
**Collector**	Rui Seabra	**Strain Status**	non-axenic, unicyanobacterial
**Collection Date**	2006/11/20	**Preservation type**	subculturing
**Location**	Portugal: Praia de Moledo, Caminha	**Light:dark cycle**	12:12-h
**Latitude & Longitude**	41.84963 N 8.866717 W	**Temperature (ºC)**	19
		**Light intensity**	10–30 µmol photons m−2 s−1
**References Publicly Available Articles**	Leão et al. (2013) Mar. Drugs 11 (4), 1316-1335		
**References Publicly Available Theses**			

**Strain ID**	**Nodosilinea sp. LEGE 06145**		
**Strain Taxonomy**	Nodosilinea Perkerson, RB & Casamatta DA in Perkerson et al (2011)		
**Microphotograph 400×**	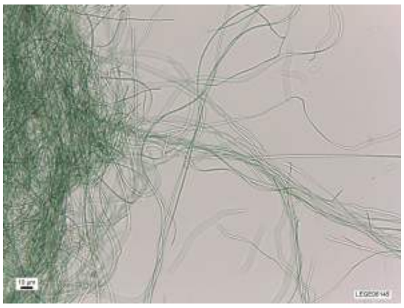	**Microphotograph 1000×**	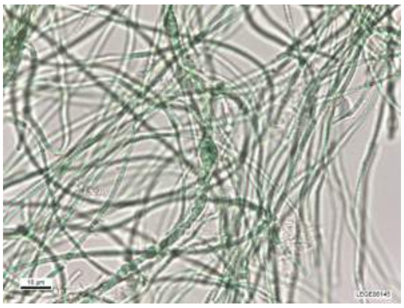
**Coidentity**	n.a.	**Morphometrics (Width × Length or Diameter) (µm)**	1,2±0,1 × 1,2±0,2
**Other Code(s)**	LEAN 058	**Taxonomic notes/diacritical features**	nodules observed
**Parent Sample**	O.06 MOL 1 - DA/DC/DD	**Macroscopic growth features**	color: cyano; forming a smooth biofilm
**Isolation Date**		**Environment**	aquatic, marine
**Order (Older Classification Scheme)**	Oscillatoriales	**Habitat Sample Description**	wave-exposed tide puddle, rock surface scraping
**Order 2014 (Modern Taxonomy)**	Synechococcales	**Ecophysiological traits**	
**Taxonomy Notes**		**Notes on ecophysiological traits**	
**Accession Number(s)_16S**	KU951772	**Isolator**	Vitor Ramos
**Accession Number(s)_others (product/gene /region)**		**Medium**	Z8 25‰ TM sea salt w/ vit. B12
**Collector**	Rui Seabra/Vitor Ramos	**Strain Status**	non-axenic, unicyanobacterial
**Collection Date**	2006/11/20	**Preservation type**	subculturing
**Location**	Portugal: Praia de Moledo, Caminha	**Light:dark cycle**	12:12-h
**Latitude & Longitude**	41.84963 N 8.866717 W	**Temperature (ºC)**	19
		**Light intensity**	10–30 µmol photons m−2 s−1
**References Publicly Available Articles**			
**References Publicly Available Theses**	Macieira R. (2013) MSc Dissertation, FCUP, University of Porto, Porto, Portugal		

**Strain ID**	**Nodosilinea sp. LEGE 06152**		
**Strain Taxonomy**	Nodosilinea Perkerson, RB & Casamatta DA in Perkerson et al (2011)		
**Microphotograph 400×**	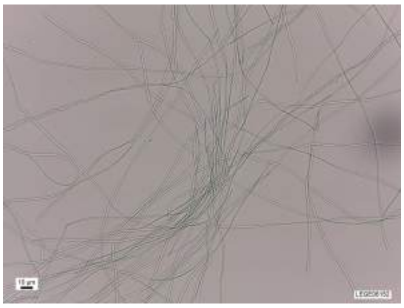	**Microphotograph 1000×**	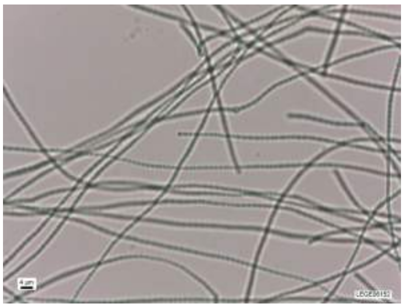
**Coidentity**	n.a.	**Morphometrics (Width × Length or Diameter) (µm)**	1,1±0,1 × 1,5±0,2
**Other Code(s)**	LEAN 066	**Taxonomic notes/diacritical features**	
**Parent Sample**	O.06 LAV p5 - AA	**Macroscopic growth features**	color: cyano; growing homogeneously; mucilaginous
**Isolation Date**		**Environment**	aquatic, marine
**Order (Older Classification Scheme)**	Oscillatoriales	**Habitat Sample Description**	sea water sample, coastal, surf zone
**Order 2014 (Modern Taxonomy)**	Synechococcales	**Ecophysiological traits**	EPS production
**Taxonomy Notes**		**Notes on ecophysiological traits**	
**Accession Number(s)_16S**	HQ832915	**Isolator**	Vitor Ramos
**Accession Number(s)_others (product/gene /region)**	KC842316 (polyketide synthase)	**Medium**	Z8 25‰ TM sea salt w/ vit. B12
**Collector**	Rui Seabra/Vitor Ramos	**Strain Status**	non-axenic, unicyanobacterial
**Collection Date**	2006/11/22	**Preservation type**	subculturing
**Location**	Portugal: Praia de Lavadores, Canidelo	**Light:dark cycle**	12:12-h
**Latitude & Longitude**	41.12919 N 8.668578 W	**Temperature (ºC)**	19
		**Light intensity**	10–30 µmol photons m−2 s−1
**References Publicly Available Articles**	Brito et al. (2012) Syst. Appl. Microbiol. 35, 110–119; Leão et al. (2013) Mar. Drugs 11 (4), 1316-1335; Costa et al. (2014) Mar. Drugs 12, 98-114; Brito et al. (2015) Algal Res. 9, 218-226		
**References Publicly Available Theses**	Costa M.S. (2011) MSc Dissertation, ICBAS and FCUP, University of Porto, Porto, Portugal		

Strain ID	**Nodosilinea sp. LEGE 06191**		
**Strain Taxonomy**	Nodosilinea Perkerson, RB & Casamatta DA in Perkerson et al (2011)		
**Microphotograph 400×**	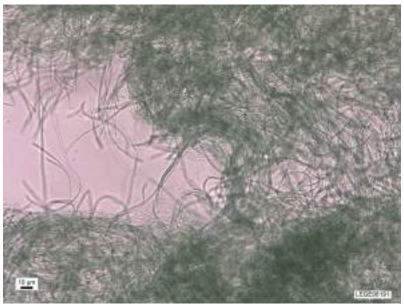	**Microphotograph 1000×**	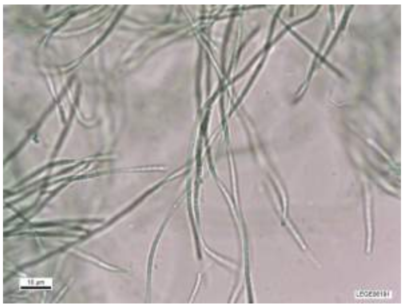
**Coidentity**	n.a.	**Morphometrics (Width × Length or Diameter) (µm)**	1,3±0,1 × 2,0±0,2
**Other Code(s)**	LEAN 156	**Taxonomic notes/diacritical features**	
**Parent Sample**	O.06 BUR p4 - CA	**Macroscopic growth features**	color: cyano; forming a firm biofilm
**Isolation Date**		**Environment**	aquatic, marine
**Order (Older Classification Scheme)**	Oscillatoriales	**Habitat Sample Description**	sea water sample, coastal, surf zone
**Order 2014 (Modern Taxonomy)**	Synechococcales	**Ecophysiological traits**	
**Taxonomy Notes**		**Notes on ecophysiological traits**	
**Accession Number(s)_16S**	KU951774	**Isolator**	Vitor Ramos
**Accession Number(s)_others (product/gene /region)**		**Medium**	Z8 25‰ TM sea salt w/ vit. B12
**Collector**	Rui Seabra	**Strain Status**	non-axenic, unicyanobacterial
**Collection Date**	2006/12/11	**Preservation type**	subculturing
**Location**	Portugal: Praia de Burgau, Budens	**Light:dark cycle**	12:12-h
**Latitude & Longitude**	37.07156 N 8.774722 W	**Temperature (ºC)**	19
		**Light intensity**	10–30 µmol photons m−2 s−1
**References Publicly Available Articles**	Leão et al. (2013) Mar. Drugs 11 (4), 1316-1335		
**References Publicly Available Theses**			

Strain ID	**Nodosilinea sp. LEGE 06193**		
**Strain Taxonomy**	Nodosilinea Perkerson, RB & Casamatta DA in Perkerson et al (2011)		
**Microphotograph 400×**	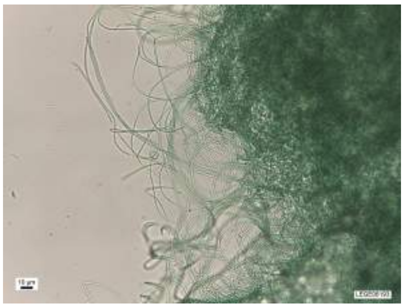	**Microphotograph 1000×**	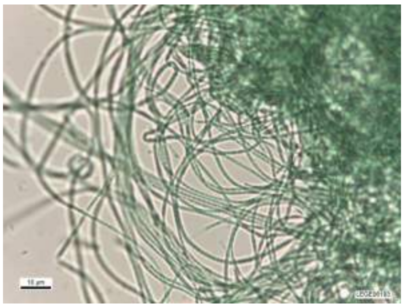
**Coidentity**	n.a.	**Morphometrics (Width × Length or Diameter) (µm)**	1,0±0,1 × 1,5±0,2
**Other Code(s)**	LEAN 158	**Taxonomic notes/diacritical features**	
**Parent Sample**	O.06 ODA p2 - AA	**Macroscopic growth features**	color: cyano; forming a firm biofilm
**Isolation Date**		**Environment**	aquatic, marine
**Order (Older Classification Scheme)**	Oscillatoriales	**Habitat Sample Description**	sea water sample, coastal, surf zone
**Order 2014 (Modern Taxonomy)**	Synechococcales	**Ecophysiological traits**	
**Taxonomy Notes**		**Notes on ecophysiological traits**	
**Accession Number(s)_16S**	KU951775	**Isolator**	Vitor Ramos
**Accession Number(s)_others (product/gene /region)**		**Medium**	Z8 25‰ TM sea salt w/ vit. B12
**Collector**	Rui Seabra	**Strain Status**	non-axenic, unicyanobacterial
**Collection Date**	2006/07/18	**Preservation type**	subculturing
**Location**	Portugal: Praia de Olhos d'Água, Albufeira	**Light:dark cycle**	12:12-h
**Latitude & Longitude**	37.08973 N 8.191017 W	**Temperature (ºC)**	19
		**Light intensity**	10–30 µmol photons m−2 s−1
**References Publicly Available Articles**			
**References Publicly Available Theses**			

Strain ID	**Nodosilinea sp. LEGE 06308**		
**Strain Taxonomy**	Nodosilinea Perkerson, RB & Casamatta DA in Perkerson et al (2011)		
**Microphotograph 400×**	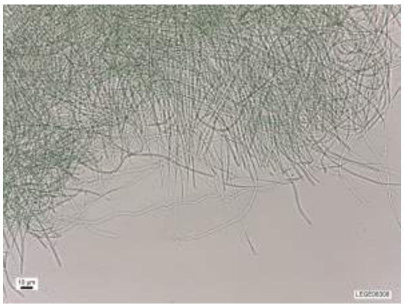	**Microphotograph 1000×**	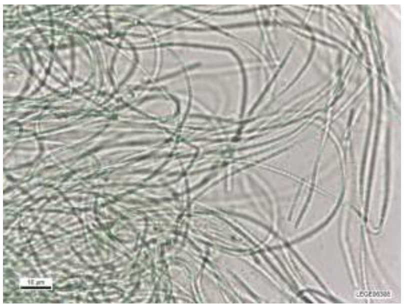
**Coidentity**	n.a.	**Morphometrics (Width × Length or Diameter) (µm)**	1,1±0,2 × 1,9±0,6
**Other Code(s)**		**Taxonomic notes/diacritical features**	
**Parent Sample**	71	**Macroscopic growth features**	color: cyano; forming a smooth biofilm
**Isolation Date**		**Environment**	aquatic, brackish
**Order (Older Classification Scheme)**	Oscillatoriales	**Habitat Sample Description**	mesotidal zone, benthic
**Order 2014 (Modern Taxonomy)**	Synechococcales	**Ecophysiological traits**	
**Taxonomy Notes**		**Notes on ecophysiological traits**	
**Accession Number(s)_16S**	HM217060	**Isolator**	Viviana Lopes
**Accession Number(s)_others (product/gene /region)**		**Medium**	Z8
**Collector**	Viviana Lopes	**Strain Status**	non-axenic, unicyanobacterial
**Collection Date**	2006	**Preservation type**	subculturing
**Location**	Portugal: Minho estuary, Caminha	**Light:dark cycle**	12:12-h
**Latitude & Longitude**	41.87933 N 8.838472 W	**Temperature (ºC)**	19
		**Light intensity**	10–30 µmol photons m−2 s−1
**References Publicly Available Articles**	Lopes et al. (2012) Mar. Environ. Res. 73, 7–16		
**References Publicly Available Theses**	Lopes V.R. (2011) PhD Thesis, FCUP, University of Porto, Porto, Portugal		

**Strain ID**	**Nodosilinea sp. LEGE 07080**		
**Strain Taxonomy**	Nodosilinea Perkerson, RB & Casamatta DA in Perkerson et al (2011)		
**Microphotograph 400×**	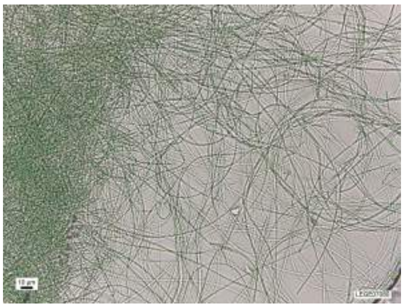	**Microphotograph 1000×**	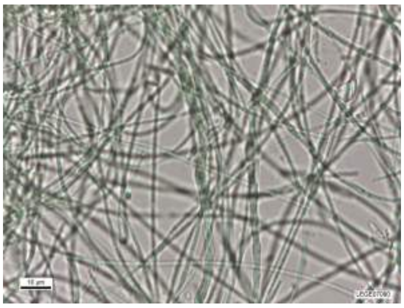
**Coidentity**	n.a.	**Morphometrics (Width × Length or Diameter) (µm)**	1,2±0,1 × 1,5±0,3
**Other Code(s)**		**Taxonomic notes/diacritical features**	facultative sheath
**Parent Sample**	VL 186	**Macroscopic growth features**	color: cyano; forming a smooth biofilm
**Isolation Date**		**Environment**	aquatic, brackish
**Order (Older Classification Scheme)**	Oscillatoriales	**Habitat Sample Description**	mesotidal zone, benthic
**Order 2014 (Modern Taxonomy)**	Synechococcales	**Ecophysiological traits**	
**Taxonomy Notes**		**Notes on ecophysiological traits**	
**Accession Number(s)_16S**	HM217085	**Isolator**	Viviana Lopes
**Accession Number(s)_others (product/gene /region)**		**Medium**	Z8
**Collector**	Viviana Lopes	**Strain Status**	non-axenic, unicyanobacterial
**Collection Date**	Apr-2007	**Preservation type**	subculturing
**Location**	Portugal: Minho estuary, Caminha	**Light:dark cycle**	12:12-h
**Latitude & Longitude**	41.86697 N 8.855833 W	**Temperature (ºC)**	19
		**Light intensity**	10–30 µmol photons m−2 s−1
**References Publicly Available Articles**	Lopes et al. (2010) Mar. Drugs 8, 471-482; Baptista et al. (2011) Toxicon 58, 410–414; Lopes et al. (2011) Toxicol. In Vitro 25, 944–950; Lopes & Vasconcelos (2011) Mar. Drugs 9, 790-802; Cianca et al. (2012) Amino Acids 42, 2473–2479; Lopes et al. (2012) Mar. Environ. Res. 73, 7–16; Almeida et al. (2015) Toxins 7(8), 2739-2756		
**References Publicly Available Theses**	Lopes V.R. (2011) PhD Thesis, FCUP, University of Porto, Porto, Portugal		

Strain ID	**Nodosilinea sp. LEGE 07085**		
**Strain Taxonomy**	Nodosilinea Perkerson, RB & Casamatta DA in Perkerson et al (2011)		
**Microphotograph 400×**	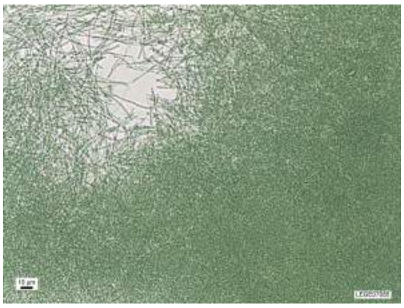	**Microphotograph 1000×**	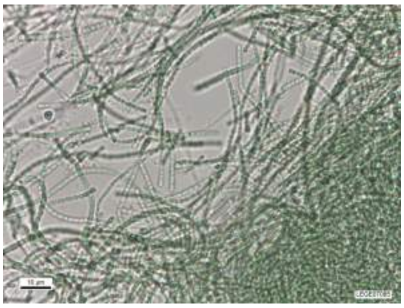
**Coidentity**	n.a.	**Morphometrics (Width × Length or Diameter) (µm)**	1,2±0,1 × 1,8±0,3
**Other Code(s)**		**Taxonomic notes/diacritical features**	trichomes constricted at cross-walls
**Parent Sample**	VL 213_1	**Macroscopic growth features**	color: cyano; forming a firm biofilm
**Isolation Date**		**Environment**	aquatic, brackish
**Order (Older Classification Scheme)**	Oscillatoriales	**Habitat Sample Description**	mesotidal zone, benthic
**Order 2014 (Modern Taxonomy)**	Synechococcales	**Ecophysiological traits**	
**Taxonomy Notes**		**Notes on ecophysiological traits**	
**Accession Number(s)_16S**	HM217079	**Isolator**	Viviana Lopes
**Accession Number(s)_others (product/gene /region)**		**Medium**	Z8
**Collector**	Viviana Lopes	**Strain Status**	non-axenic, unicyanobacterial
**Collection Date**	Apr-2007	**Preservation type**	subculturing
**Location**	Portugal: Douro estuary, Porto	**Light:dark cycle**	12:12-h
**Latitude & Longitude**	41.14792 N 8.651611 W	**Temperature (ºC)**	19
		**Light intensity**	10–30 µmol photons m−2 s−1
**References Publicly Available Articles**	Lopes et al. (2010) Mar. Drugs 8, 471-482; Baptista et al. (2011) Toxicon 58, 410–414; Lopes et al. (2011) Toxicol. In Vitro 25, 944–950; Lopes & Vasconcelos (2011) Mar. Drugs 9, 790-802; Cianca et al. (2012) Amino Acids 42, 2473–2479; Lopes et al. (2012) Mar. Environ. Res. 73, 7–16; Almeida et al. (2015) Toxins 7(8), 2739-2756		
**References Publicly Available Theses**	Lopes V.R. (2011) PhD Thesis, FCUP, University of Porto, Porto, Portugal		

**Strain ID**	**Nodosilinea sp. LEGE 07088**		
**Strain Taxonomy**	Nodosilinea Perkerson, RB & Casamatta DA in Perkerson et al (2011)		
**Microphotograph 400×**	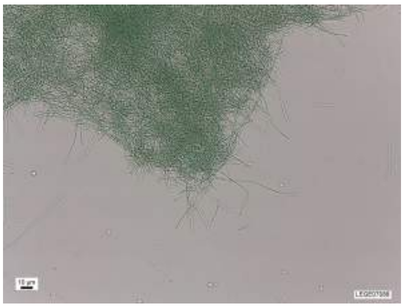	**Microphotograph 1000×**	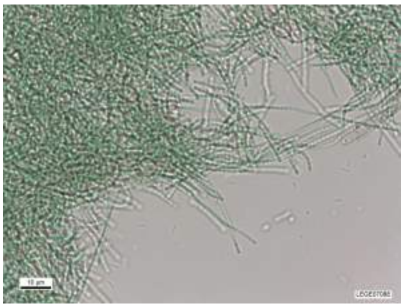
**Coidentity**	n.a.	**Morphometrics (Width × Length or Diameter) (µm)**	1,1±0,1 × 1,3±0,2
**Other Code(s)**		**Taxonomic notes/diacritical features**	trichomes constricted at cross-walls
**Parent Sample**	VL 359_2	**Macroscopic growth features**	color: cyano; forming a firm biofilm
**Isolation Date**		**Environment**	aquatic, brackish
**Order (Older Classification Scheme)**	Oscillatoriales	**Habitat Sample Description**	mesotidal zone, benthic
**Order 2014 (Modern Taxonomy)**	Synechococcales	**Ecophysiological traits**	
**Taxonomy Notes**		**Notes on ecophysiological traits**	
**Accession Number(s)_16S**	HM217064	**Isolator**	Viviana Lopes
**Accession Number(s)_others (product/gene /region)**		**Medium**	Z8
**Collector**	Viviana Lopes	**Strain Status**	non-axenic, unicyanobacterial
**Collection Date**	2006/10/10	**Preservation type**	subculturing
**Location**	Portugal: Douro estuary, Porto	**Light:dark cycle**	12:12-h
**Latitude & Longitude**	41.14658 N 8.657972 W	**Temperature (ºC)**	19
		**Light intensity**	10–30 µmol photons m−2 s−1
**References Publicly Available Articles**	Lopes et al. (2012) Mar. Environ. Res. 73, 7–16		
**References Publicly Available Theses**	Lopes V.R. (2011) PhD Thesis, FCUP, University of Porto, Porto, Portugal		

Strain ID	**Nodosilinea sp. LEGE 07091**		
**Strain Taxonomy**	Nodosilinea Perkerson, RB & Casamatta DA in Perkerson et al (2011)		
**Microphotograph 400×**	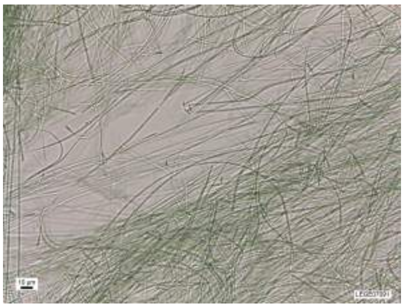	**Microphotograph 1000×**	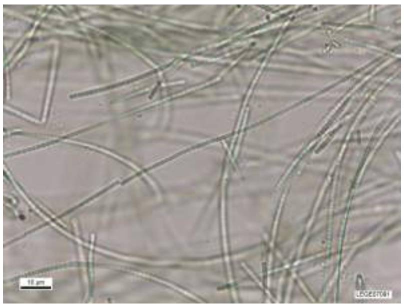
**Coidentity**	n.a.	**Morphometrics (Width × Length or Diameter) (µm)**	1,2±0,1 × 1,3±0,3
**Other Code(s)**		**Taxonomic notes/diacritical features**	trichomes constricted at cross-walls; with sheath
**Parent Sample**	VL 218_2	**Macroscopic growth features**	color: cyano; forming a firm biofilm; mucilaginous
**Isolation Date**		**Environment**	aquatic, brackish
**Order (Older Classification Scheme)**	Oscillatoriales	**Habitat Sample Description**	mesotidal zone, benthic
**Order 2014 (Modern Taxonomy)**	Synechococcales	**Ecophysiological traits**	EPS production
**Taxonomy Notes**		**Notes on ecophysiological traits**	
**Accession Number(s)_16S**	HM217068	**Isolator**	Viviana Lopes
**Accession Number(s)_others (product/gene /region)**		**Medium**	Z8
**Collector**	Viviana Lopes	**Strain Status**	non-axenic, unicyanobacterial
**Collection Date**	Apr-2007	**Preservation type**	subculturing
**Location**	Portugal: Douro estuary, Porto	**Light:dark cycle**	12:12-h
**Latitude & Longitude**	41.14658 N 8.632556 W	**Temperature (ºC)**	19
		**Light intensity**	10–30 µmol photons m−2 s−1
**References Publicly Available Articles**	Lopes et al. (2010) Mar. Drugs 8, 471-482; Baptista et al. (2011) Toxicon 58, 410–414; Lopes et al. (2011) Toxicol. In Vitro 25, 944–950; Lopes & Vasconcelos (2011) Mar. Drugs 9, 790-802; Cianca et al. (2012) Amino Acids 42, 2473–2479; Lopes et al. (2012) Mar. Environ. Res. 73, 7–16		
**References Publicly Available Theses**	Lopes V.R. (2011) PhD Thesis, FCUP, University of Porto, Porto, Portugal; Lopes V.R. (2011) PhD Thesis, FCUP, University of Porto, Porto, Portugal		

Strain ID	**Nodosilinea sp. LEGE 07298**		
**Strain Taxonomy**	Nodosilinea Perkerson, RB & Casamatta DA in Perkerson et al (2011)		
**Microphotograph 400×**	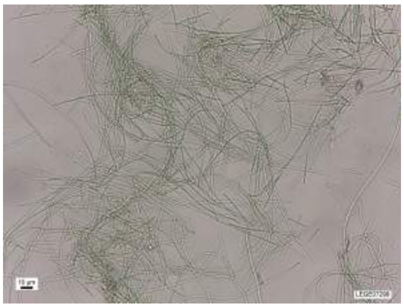	**Microphotograph 1000×**	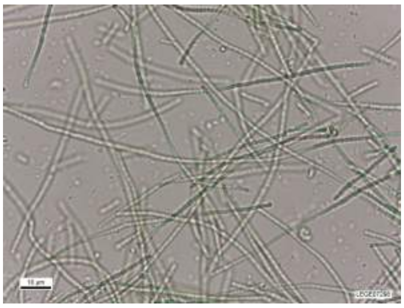
**Coidentity**	n.a.	**Morphometrics (Width × Length or Diameter) (µm)**	1,2±0,1 × 1,9±0,2
**Other Code(s)**		**Taxonomic notes/diacritical features**	
**Parent Sample**	VL PORT 110407	**Macroscopic growth features**	color: cyano; growing homogeneously, but also forming aggregates; mucilaginous
**Isolation Date**		**Environment**	aquatic, brackish
**Order (Older Classification Scheme)**	Oscillatoriales	**Habitat Sample Description**	water sample, on the estuary shore
**Order 2014 (Modern Taxonomy)**	Synechococcales	**Ecophysiological traits**	EPS production
**Taxonomy Notes**		**Notes on ecophysiological traits**	
**Accession Number(s)_16S**	HM217044	**Isolator**	Viviana Lopes
**Accession Number(s)_others (product/gene /region)**		**Medium**	Z8 25‰ TM sea salt w/ vit. B12
**Collector**	Viviana Lopes	**Strain Status**	non-axenic, unicyanobacterial
**Collection Date**	2007/04/11	**Preservation type**	subculturing
**Location**	Portugal: Douro estuary, Porto	**Light:dark cycle**	12:12-h
**Latitude & Longitude**	41.14658 N 8.657972 W	**Temperature (ºC)**	19
		**Light intensity**	10–30 µmol photons m−2 s−1
**References Publicly Available Articles**			
**References Publicly Available Theses**			

**Strain ID**	**Nodosilinea sp. LEGE 07309**		
**Strain Taxonomy**	Nodosilinea Perkerson, RB & Casamatta DA in Perkerson et al (2011)		
**Microphotograph 400×**	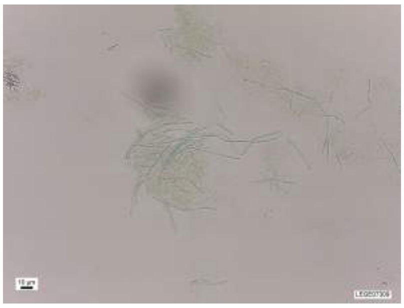	**Microphotograph 1000×**	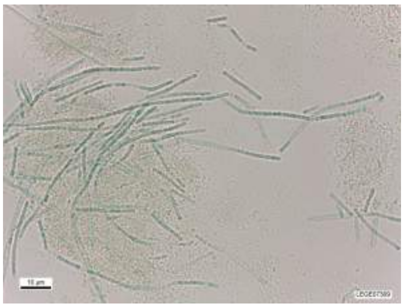
**Coidentity**	n.a.	**Morphometrics (Width × Length or Diameter) (µm)**	1,1±0,2 × 1,7±0,5
**Other Code(s)**		**Taxonomic notes/diacritical features**	
**Parent Sample**	217	**Macroscopic growth features**	color: cyano; growing homogeneously, but also forming aggregates
**Isolation Date**		**Environment**	aquatic, brackish
**Order (Older Classification Scheme)**	Oscillatoriales	**Habitat Sample Description**	mesotidal zone, benthic
**Order 2014 (Modern Taxonomy)**	Synechococcales	**Ecophysiological traits**	
**Taxonomy Notes**		**Notes on ecophysiological traits**	
**Accession Number(s)_16S**	HM217053	**Isolator**	Viviana Lopes
**Accession Number(s)_others (product/gene /region)**		**Medium**	Z8
**Collector**	Viviana Lopes	**Strain Status**	non-axenic, unicyanobacterial
**Collection Date**	2007	**Preservation type**	subculturing
**Location**	Portugal: Douro estuary, Porto	**Light:dark cycle**	12:12-h
**Latitude & Longitude**	41.14792 N 8.651611 W	**Temperature (ºC)**	19
		**Light intensity**	10–30 µmol photons m−2 s−1
**References Publicly Available Articles**	Lopes et al. (2012) Mar. Environ. Res. 73, 7–16		
**References Publicly Available Theses**	Lopes V.R. (2011) PhD Thesis, FCUP, University of Porto, Porto, Portugal		

Strain ID	**Nodosilinea sp. LEGE 07311**		
**Strain Taxonomy**	Nodosilinea Perkerson, RB & Casamatta DA in Perkerson et al (2011)		
**Microphotograph 400×**	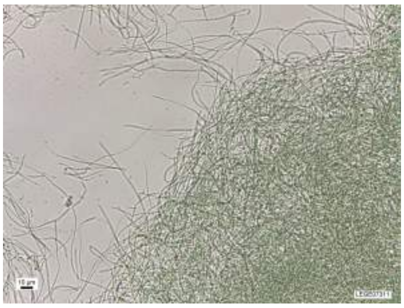	**Microphotograph 1000×**	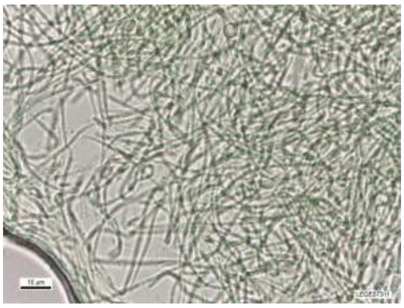
**Coidentity**	n.a.	**Morphometrics (Width × Length or Diameter) (µm)**	1,0±0,1 × 1,7±0,2
**Other Code(s)**		**Taxonomic notes/diacritical features**	with sheath
**Parent Sample**	303	**Macroscopic growth features**	color: cyano; forming a firm biofilm
**Isolation Date**		**Environment**	aquatic, brackish
**Order (Older Classification Scheme)**	Oscillatoriales	**Habitat Sample Description**	mesotidal zone, benthic
**Order 2014 (Modern Taxonomy)**	Synechococcales	**Ecophysiological traits**	
**Taxonomy Notes**		**Notes on ecophysiological traits**	
**Accession Number(s)_16S**	HM217056	**Isolator**	Viviana Lopes
**Accession Number(s)_others (product/gene /region)**		**Medium**	Z8
**Collector**	Viviana Lopes	**Strain Status**	non-axenic, unicyanobacterial
**Collection Date**	2007	**Preservation type**	subculturing
**Location**	Portugal: Douro estuary, Vila Nova de Gaia	**Light:dark cycle**	12:12-h
**Latitude & Longitude**	41.13656 N 8.660861 W	**Temperature (ºC)**	19
		**Light intensity**	10–30 µmol photons m−2 s−1
**References Publicly Available Articles**	Lopes et al. (2012) Mar. Environ. Res. 73, 7–16		
**References Publicly Available Theses**	Lopes V.R. (2011) PhD Thesis, FCUP, University of Porto, Porto, Portugal		

Strain ID	**Nodosilinea sp. LEGE 07314**		
**Strain Taxonomy**	Nodosilinea Perkerson, RB & Casamatta DA in Perkerson et al (2011)		
**Microphotograph 400×**	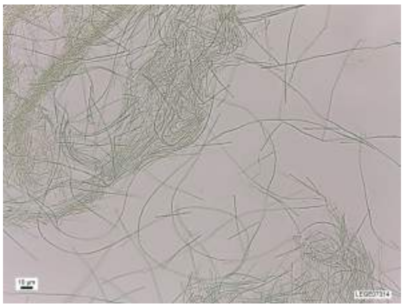	**Microphotograph 1000×**	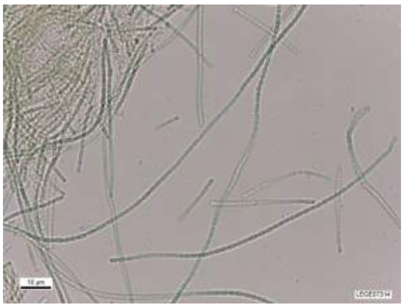
**Coidentity**	n.a.	**Morphometrics (Width × Length or Diameter) (µm)**	1,3±0,1 × 2,3±0,4
**Other Code(s)**		**Taxonomic notes/diacritical features**	trichomes constricted at cross-walls
**Parent Sample**	CO326	**Macroscopic growth features**	color: cyano; forming a smooth biofilm and aggregates; mucilaginous
**Isolation Date**		**Environment**	aquatic, brackish
**Order (Older Classification Scheme)**	Oscillatoriales	**Habitat Sample Description**	unknown
**Order 2014 (Modern Taxonomy)**	Synechococcales	**Ecophysiological traits**	EPS production
**Taxonomy Notes**		**Notes on ecophysiological traits**	
**Accession Number(s)_16S**	HM217061	**Isolator**	Viviana Lopes
**Accession Number(s)_others (product/gene /region)**		**Medium**	Z8
**Collector**	Viviana Lopes	**Strain Status**	non-axenic, unicyanobacterial
**Collection Date**	2006	**Preservation type**	subculturing
**Location**	Portugal: Minho estuary, Seixas	**Light:dark cycle**	12:12-h
**Latitude & Longitude**	41.88797 N 8.825722 W	**Temperature (ºC)**	19
		**Light intensity**	10–30 µmol photons m−2 s−1
**References Publicly Available Articles**	Lopes et al. (2012) Mar. Environ. Res. 73, 7–16		
**References Publicly Available Theses**	Lopes V.R. (2011) PhD Thesis, FCUP, University of Porto, Porto, Portugal		

**Strain ID**	**Nodosilinea sp. LEGE 07364**		
**Strain Taxonomy**	Nodosilinea Perkerson, RB & Casamatta DA in Perkerson et al (2011)		
**Microphotograph 400×**	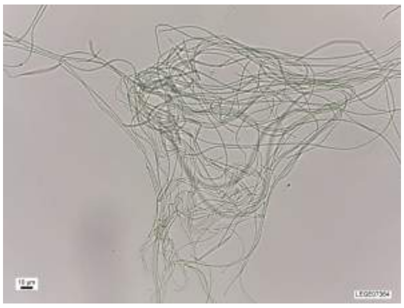	**Microphotograph 1000×**	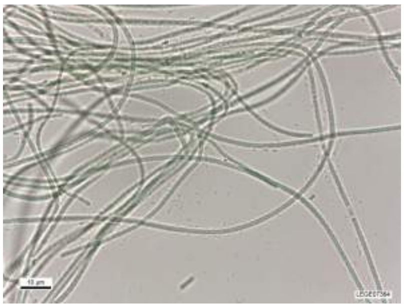
**Coidentity**	n.a.	**Morphometrics (Width × Length or Diameter) (µm)**	1,2±0,1 × 2,1±0,4
**Other Code(s)**		**Taxonomic notes/diacritical features**	trichomes constricted at cross-walls; with sheath
**Parent Sample**	VL MS 231	**Macroscopic growth features**	color: cyano; forming a firm biofilm
**Isolation Date**		**Environment**	aquatic, brackish
**Order (Older Classification Scheme)**	Oscillatoriales	**Habitat Sample Description**	mesotidal zone, benthic
**Order 2014 (Modern Taxonomy)**	Synechococcales	**Ecophysiological traits**	
**Taxonomy Notes**		**Notes on ecophysiological traits**	
**Accession Number(s)_16S**	KU951776	**Isolator**	Viviana Lopes
**Accession Number(s)_others (product/gene /region)**		**Medium**	Z8
**Collector**	Viviana Lopes	**Strain Status**	non-axenic, unicyanobacterial
**Collection Date**	2007	**Preservation type**	subculturing
**Location**	Portugal	**Light:dark cycle**	12:12-h
**Latitude & Longitude**	unknown	**Temperature (ºC)**	19
		**Light intensity**	10–30 µmol photons m−2 s−1
**References Publicly Available Articles**			
**References Publicly Available Theses**			

Strain ID	**Nodosilinea sp. LEGE 10376**		
**Strain Taxonomy**	Nodosilinea Perkerson, RB & Casamatta DA in Perkerson et al (2011)		
**Microphotograph 400×**	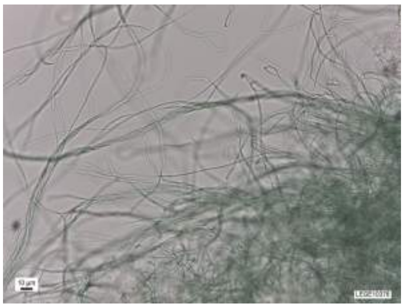	**Microphotograph 1000×**	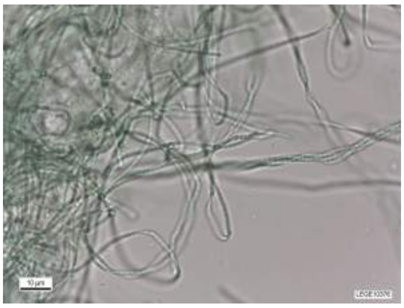
**Coidentity**	n.a.	**Morphometrics (Width × Length or Diameter) (µm)**	1,1±0,1 × 1,7±0,3
**Other Code(s)**	18A	**Taxonomic notes/diacritical features**	coiled trichomes; constricted at cross-walls
**Parent Sample**		**Macroscopic growth features**	color: reddish; forming aggregates
**Isolation Date**		**Environment**	aquatic, marine
**Order (Older Classification Scheme)**	Oscillatoriales	**Habitat Sample Description**	intertidal zone, on a marine sponge
**Order 2014 (Modern Taxonomy)**	Synechococcales	**Ecophysiological traits**	
**Taxonomy Notes**		**Notes on ecophysiological traits**	
**Accession Number(s)_16S**	KU951777	**Isolator**	Ana Regueiras
**Accession Number(s)_others (product/gene /region)**		**Medium**	Z8 25‰ TM sea salt w/ vit. B12
**Collector**	Ana Regueiras	**Strain Status**	non-axenic, unicyanobacterial
**Collection Date**	2010/11/06	**Preservation type**	subculturing
**Location**	Portugal: Porto Côvo	**Light:dark cycle**	12:12-h
**Latitude & Longitude**	37.86748 N 8.793414 W	**Temperature (ºC)**	19
		**Light intensity**	10–30 µmol photons m−2 s−1
**References Publicly Available Articles**			
**References Publicly Available Theses**	Vivas J. (2013) MSc Dissertation, FCUP, University of Porto, Porto, Portugal		

Strain ID	**Nodosilinea sp. LEGE 11429**		
**Strain Taxonomy**	Nodosilinea Perkerson, RB & Casamatta DA in Perkerson et al (2011)		
**Microphotograph 400×**	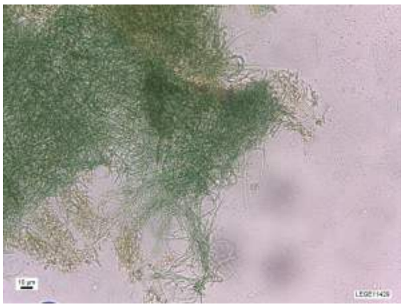	**Microphotograph 1000×**	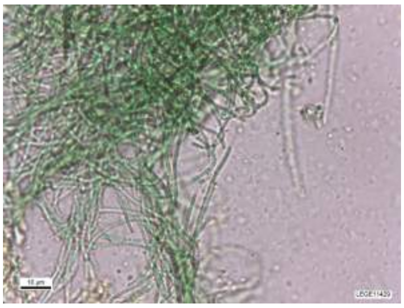
**Coidentity**	n.a.	**Morphometrics (Width × Length or Diameter) (μm)**	1,3±0,2 × 1,8±0,4
**Other Code(s)**		**Taxonomic notes/diacritical features**	
**Parent Sample**	8.003.1011_AP.F(A)b/i	**Macroscopic growth features**	color: cyano; forming a firm biofilm
**Isolation Date**		**Environment**	aquatic, marine
**Order (Older Classification Scheme)**	Oscillatoriales	**Habitat Sample Description**	subtidal sample, epilithic (13m depth), about 200 m off the shore
**Order 2014 (Modern Taxonomy)**	Synechococcales	**Ecophysiological traits**	
**Taxonomy Notes**		**Notes on ecophysiological traits**	
**Accession Number(s)_16S**	KU951778	**Isolator**	Ângela Pinheiro
**Accession Number(s)_others (product/gene /region)**		**Medium**	Z8 25‰ TM sea salt w/ vit. B12
**Collector**	Pedro Leão/Aldo Barreiro	**Strain Status**	non-axenic, unicyanobacterial
**Collection Date**	2011/10/03	**Preservation type**	subculturing
**Location**	Portugal: 'A Pedra', diving spot in front of the fort 'Castelo do Queijo'	**Light:dark cycle**	12:12-h
**Latitude & Longitude**	41.185809 N 8.719079 W	**Temperature (ºC)**	19
		**Light intensity**	10–30 μmol photons m−2 s−1
**References Publicly Available Articles**			
**References Publicly Available Theses**			

**Strain ID**	**Nodosilinea sp. LEGE 11468**		
**Strain Taxonomy**	Nodosilinea Perkerson, RB & Casamatta DA in Perkerson et al (2011)		
**Microphotograph 400×**	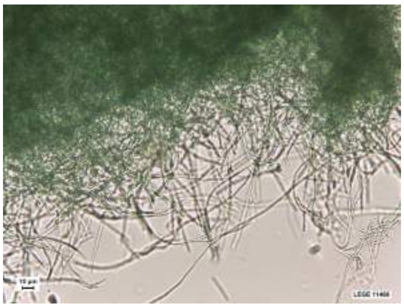	**Microphotograph 1000×**	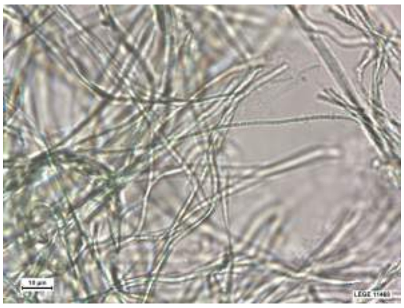
**Coidentity**	n.a.	**Morphometrics (Width × Length or Diameter) (µm)**	1,3±0,3 × 1,3±0,3
**Other Code(s)**		**Taxonomic notes/diacritical features**	
**Parent Sample**	8.003.1011	**Macroscopic growth features**	color: cyano; forming a firm biofilm
**Isolation Date**		**Environment**	aquatic, marine
**Order (Older Classification Scheme)**	Oscillatoriales	**Habitat Sample Description**	subtidal sample, epilithic (13m depth), about 200 m off the shore
**Order 2014 (Modern Taxonomy)**	Synechococcales	**Ecophysiological traits**	
**Taxonomy Notes**		**Notes on ecophysiological traits**	
**Accession Number(s)_16S**	KU951779	**Isolator**	Ângela Pinheiro
**Accession Number(s)_others (product/gene /region)**		**Medium**	Z8 25‰ TM sea salt w/ vit. B12
**Collector**	Pedro Leão/Aldo Barreiro	**Strain Status**	non-axenic, unicyanobacterial
**Collection Date**	2011/10/03	**Preservation type**	subculturing
**Location**	Portugal: 'A Pedra', diving spot in front of the fort 'Castelo do Queijo'	**Light:dark cycle**	12:12-h
**Latitude & Longitude**	41.185809 N 8.719079 W	**Temperature (ºC)**	19
		**Light intensity**	10–30 µmol photons m−2 s−1
**References Publicly Available Articles**			
**References Publicly Available Theses**			

Strain ID	**Nodosilinea-like sp. LEGE 11424**		
**Strain Taxonomy**	Nodosilinea Perkerson, RB & Casamatta DA in Perkerson et al (2011)		
**Microphotograph 400×**	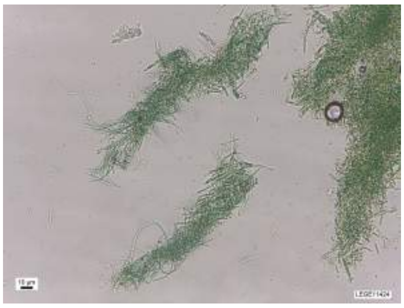	**Microphotograph 1000×**	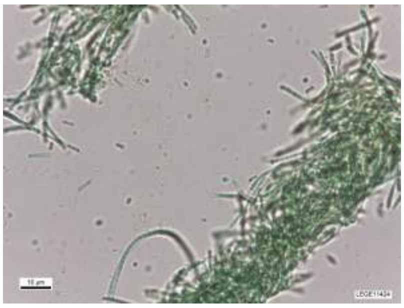
**Coidentity**	n.a.	**Morphometrics (Width × Length or Diameter) (µm)**	1,2±0,2 × 1,5±0,3
**Other Code(s)**		**Taxonomic notes/diacritical features**	filaments often short
**Parent Sample**	SC-f-Ai	**Macroscopic growth features**	color: cyano; forming a firm biofilm
**Isolation Date**		**Environment**	aquatic, marine
**Order (Older Classification Scheme)**	Oscillatoriales	**Habitat Sample Description**	subtidal sample, epilithic (10-13m depth), less than 1 km off the shore
**Order 2014 (Modern Taxonomy)**	not determined	**Ecophysiological traits**	
**Taxonomy Notes**		**Notes on ecophysiological traits**	
**Accession Number(s)_16S**		**Isolator**	Sofia Costa
**Accession Number(s)_others (product/gene /region)**		**Medium**	Z8 25‰ TM sea salt w/ vit. B12
**Collector**	Pedro Leão/Aldo Barreiro	**Strain Status**	non-axenic, unicyanobacterial
**Collection Date**	2011/09/26	**Preservation type**	subculturing
**Location**	Portugal: 'Pêlo Negro', diving spot near Leixões Harbour	**Light:dark cycle**	12:12-h
**Latitude & Longitude**	41.18557 N 8.719100 W	**Temperature (ºC)**	19
		**Light intensity**	10–30 µmol photons m−2 s−1
**References Publicly Available Articles**			
**References Publicly Available Theses**			

Strain ID	**Nodularia sp. LEGE 04288**		
**Strain Taxonomy**	Nodularia Mertens ex Bornet, É & Flahault, C (1886)		
**Microphotograph 400×**	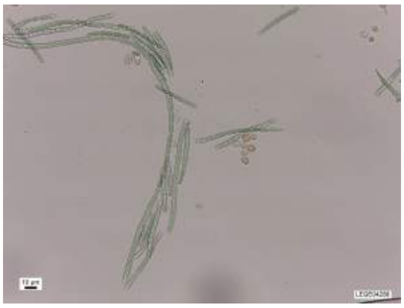	**Microphotograph 1000×**	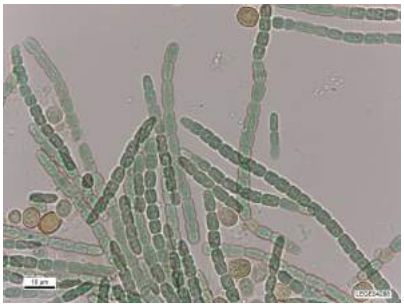
**Coidentity**	n.a.	**Morphometrics (Width × Length or Diameter) (µm)**	Ø 3,3±0,4 × 4,9±0,9 (Cells); 5,4±0,4 × 5,3±0,7 (Akinetes)
**Other Code(s)**	J82, LEANJ.82	**Taxonomic notes/diacritical features**	
**Parent Sample**		**Macroscopic growth features**	color: cyano; growing homogeneously
**Isolation Date**		**Environment**	aquatic, freshwater
**Order (Older Classification Scheme)**	Nostocales	**Habitat Sample Description**	water sample
**Order 2014 (Modern Taxonomy)**	Nostocales	**Ecophysiological traits**	
**Taxonomy Notes**		**Notes on ecophysiological traits**	
**Accession Number(s)_16S**	KU951780	**Isolator**	Joana Osswald
**Accession Number(s)_others (product/gene /region)**		**Medium**	Z8
**Collector**	Joana Osswald	**Strain Status**	non-axenic, unicyanobacterial
**Collection Date**	2004/07/13	**Preservation type**	subculturing
**Location**	Portugal: Marco de Canaveses	**Light:dark cycle**	12:12-h
**Latitude & Longitude**	unknown	**Temperature (ºC)**	19
		**Light intensity**	10–30 µmol photons m−2 s−1
**References Publicly Available Articles**			
**References Publicly Available Theses**	Macieira R. (2013) MSc Dissertation, FCUP, University of Porto, Porto, Portugal		

**Strain ID**	**Nodularia sp. LEGE 06071**		
**Strain Taxonomy**	Nodularia Mertens ex Bornet, É & Flahault, C (1886)		
**Microphotograph 400×**	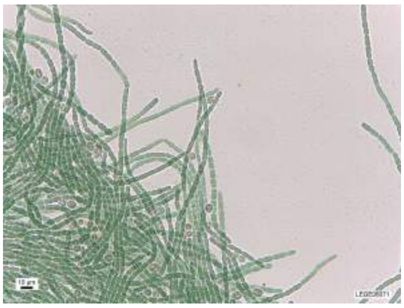	**Microphotograph 1000×**	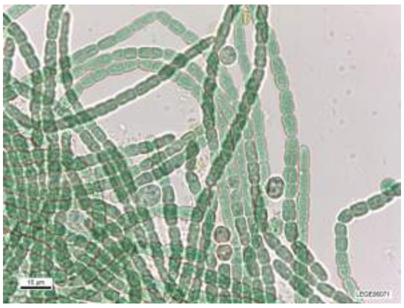
**Coidentity**	n.a.	**Morphometrics (Width × Length or Diameter) (µm)**	3,8±0,4 × 4,8±0,6 (Cells); 6,3±0,9 × 6,5±1,4 (akinetes)
**Other Code(s)**		**Taxonomic notes/diacritical features**	some conical-rounded terminal cells
**Parent Sample**	VL 100	**Macroscopic growth features**	color: cyano; forming a smooth biofilm
**Isolation Date**		**Environment**	aquatic, brackish
**Order (Older Classification Scheme)**	Nostocales	**Habitat Sample Description**	mesotidal zone, benthic
**Order 2014 (Modern Taxonomy)**	Nostocales	**Ecophysiological traits**	nitrogen-fixation, akinetes production
**Taxonomy Notes**		**Notes on ecophysiological traits**	
**Accession Number(s)_16S**	FJ830639	**Isolator**	Viviana Lopes
**Accession Number(s)_others (product/gene /region)**		**Medium**	Z8
**Collector**	Viviana Lopes	**Strain Status**	non-axenic, unicyanobacterial
**Collection Date**	2006/12/06	**Preservation type**	subculturing
**Location**	Portugal: Vouga estuary, Ria de Aveiro, Aveiro	**Light:dark cycle**	12:12-h
**Latitude & Longitude**	40.63825 N 8.659778 W	**Temperature (ºC)**	19
		**Light intensity**	10–30 µmol photons m−2 s−1
**References Publicly Available Articles**	Lopes et al. (2010) Mar. Drugs 8, 471-482; Baptista et al. (2011) Toxicon 58, 410–414; Lopes et al. (2011) Toxicol. In Vitro 25, 944–950; Cianca et al. (2012) Amino Acids 42, 2473–2479; Lopes et al. (2012) Mar. Environ. Res. 73, 7–16; Rastoll et al. (2013) J. Appl. Phycol. 25, 1483-1493; Almeida et al. (2015) Toxins 7(8), 2739-2756		
**References Publicly Available Theses**	Lopes V.R. (2011) PhD Thesis, FCUP, University of Porto, Porto, Portugal; Macieira R. (2013) MSc Dissertation, FCUP, University of Porto, Porto, Portugal		

Strain ID	**Nostoc aff. commune LEGE 04357**		
**Strain Taxonomy**	Nostoc commune Vaucher ex Bornet, É & Flahault, C (1888)		
**Microphotograph 400×**	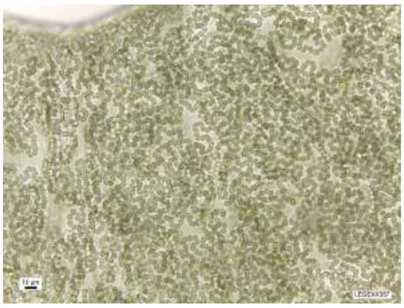	**Microphotograph 1000×**	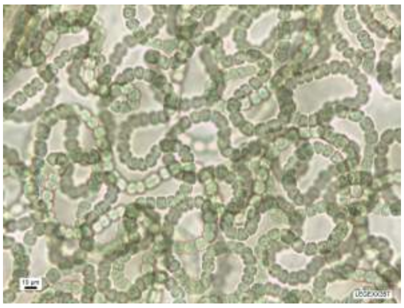
**Coidentity**	n.a.	**Morphometrics (Width × Length or Diameter) (µm)**	4,0±1,0 × 4,2±0,9 (Cells); Ø 4,5±0,7 (heterocytes)
**Other Code(s)**	Nostoc Site 3	**Taxonomic notes/diacritical features**	sac-like gelatinous thalli; with sheath
**Parent Sample**		**Macroscopic growth features**	color: olive green; forming aggregates
**Isolation Date**		**Environment**	unknown
**Order (Older Classification Scheme)**	Nostocales	**Habitat Sample Description**	unknown
**Order 2014 (Modern Taxonomy)**	Nostocales	**Ecophysiological traits**	
**Taxonomy Notes**		**Notes on ecophysiological traits**	
**Accession Number(s)_16S**	KF287120	**Isolator**	Martin Saker
**Accession Number(s)_others (product/gene /region)**	KF287121 (16S-23S rRNA ITS), KF287122 (rpoC1)	**Medium**	Z8
**Collector**	unknown	**Strain Status**	non-axenic, unicyanobacterial
**Collection Date**	2004	**Preservation type**	subculturing
**Location**	Morocco	**Light:dark cycle**	12:12-h
**Latitude & Longitude**	unknown	**Temperature (ºC)**	19
		**Light intensity**	10–30 µmol photons m−2 s−1
**References Publicly Available Articles**	Moreira et al. (2015) Arch. Microbiol. 197, 47–52		
**References Publicly Available Theses**			

Strain ID	**Nostoc cf. edaphicum LEGE 07299**		
**Strain Taxonomy**	Nostoc edaphicum Kondrateva, NV (1962)		
**Microphotograph 400×**	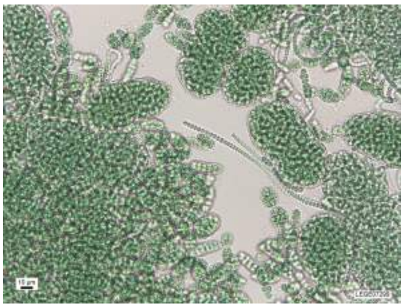	**Microphotograph 1000×**	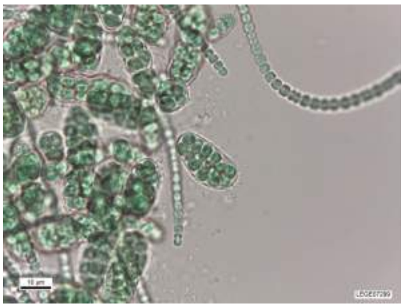
**Coidentity**	n.a.	**Morphometrics (Width × Length or Diameter) (µm)**	2,9±0,5 × 2,3±0,3 (Cells)
**Other Code(s)**		**Taxonomic notes/diacritical features**	sac-like gelatinous thalli; with sheath
**Parent Sample**	VL SE pico 250907	**Macroscopic growth features**	color: cyano; forming a firm biofilm and aggregates
**Isolation Date**		**Environment**	aquatic, brackish
**Order (Older Classification Scheme)**	Nostocales	**Habitat Sample Description**	water sample, on the estuary shore
**Order 2014 (Modern Taxonomy)**	Nostocales	**Ecophysiological traits**	nitrogen-fixation
**Taxonomy Notes**		**Notes on ecophysiological traits**	
**Accession Number(s)_16S**	HM217059	**Isolator**	Viviana Lopes
**Accession Number(s)_others (product/gene /region)**		**Medium**	Z8
**Collector**	Viviana Lopes	**Strain Status**	non-axenic, unicyanobacterial
**Collection Date**	2007/09/25	**Preservation type**	subculturing
**Location**	Portugal: Minho estuary, Seixas	**Light:dark cycle**	12:12-h
**Latitude & Longitude**	41.90181 N 8.813139 W	**Temperature (ºC)**	19
		**Light intensity**	10–30 µmol photons m−2 s−1
**References Publicly Available Articles**	Lopes et al. (2012) Mar. Environ. Res. 73, 7–16		
**References Publicly Available Theses**	Lopes V.R. (2011) PhD Thesis, FCUP, University of Porto, Porto, Portugal; Macieira R. (2013) MSc Dissertation, FCUP, University of Porto, Porto, Portugal		

**Strain ID**	**Nostoc sp. LEGE 06077**		
**Strain Taxonomy**	Nostoc Vaucher ex Bornet, É & Flahault, C (1886)		
**Microphotograph 400×**	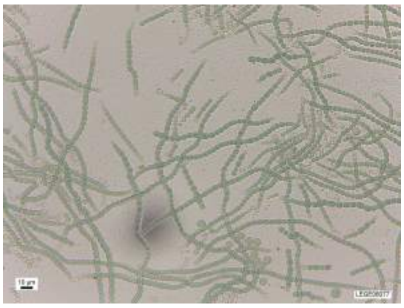	**Microphotograph 1000×**	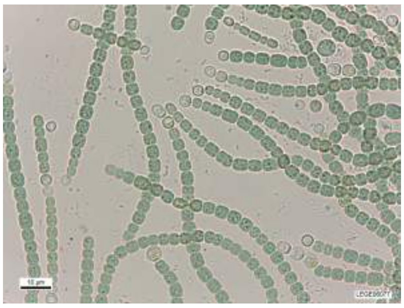
**Coidentity**	n.a.	**Morphometrics (Width × Length or Diameter) (μm)**	3,6±0,7 × 3,8±0,8 (Cells); Ø 3,3±0,4 (Heterocytes); Ø 7,0±1,3 (Akinetes)
**Other Code(s)**		**Taxonomic notes/diacritical features**	
**Parent Sample**	VL CO231106	**Macroscopic growth features**	color: cyano; forming aggregates; mucilaginous
**Isolation Date**		**Environment**	aquatic, brackish
**Order (Older Classification Scheme)**	Nostocales	**Habitat Sample Description**	water sample, on the estuary shore
**Order 2014 (Modern Taxonomy)**	Nostocales	**Ecophysiological traits**	nitrogen-fixation, EPS production
**Taxonomy Notes**		**Notes on ecophysiological traits**	dehydroabietic acid, abietic acid and anabaenopeptins A and D production (unknown ecophysiological role)
**Accession Number(s)_16S**	HM217071	**Isolator**	Viviana Lopes
**Accession Number(s)_others (product/gene /region)**		**Medium**	BG11o
**Collector**	Viviana Lopes	**Strain Status**	non-axenic, unicyanobacterial
**Collection Date**	2006/11/23	**Preservation type**	subculturing, cryopreserved
**Location**	Portugal: Minho estuary, Seixas	**Light:dark cycle**	12:12-h
**Latitude & Longitude**	41.88797 N 8.825722 W	**Temperature (ºC)**	19
		**Light intensity**	10–30 μmol photons m−2 s−1
**References Publicly Available Articles**	Lopes et al. (2010) Mar. Drugs 8, 471-482; Baptista et al. (2011) Toxicon 58, 410–414; Lopes et al. (2011) Toxicol. In Vitro 25, 944–950; Cianca et al. (2012) Amino Acids 42, 2473–2479; Cianca et al. (2012) Toxicon 59, 379–384; Lopes et al. (2012) Mar. Environ. Res. 73, 7–16; Almeida et al. (2015) Toxins 7(8), 2739-2756; Costa et al. (2016) Sci. Rep. 6, 23436		
**References Publicly Available Theses**	Lopes V.R. (2011) PhD Thesis, FCUP, University of Porto, Porto, Portugal; Macieira R. (2013) MSc Dissertation, FCUP, University of Porto, Porto, Portugal; Vasconcelos R. (2013) MSc Dissertation, ICBAS, University of Porto, Porto, Portugal; Monteiro M.I. (2015) MSc Dissertation, FCUP, University of Porto, Porto, Portugal		

**Strain ID**	**Nostoc sp. LEGE 06158**		
**Strain Taxonomy**	Nostoc Vaucher ex Bornet, É & Flahault, C (1886)		
**Microphotograph 400×**	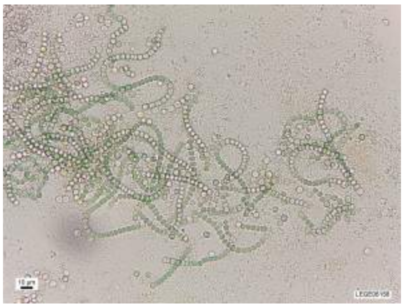	**Microphotograph 1000×**	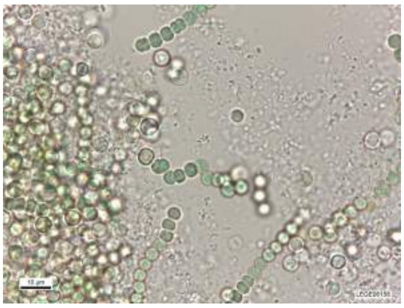
**Coidentity**	n.a.	**Morphometrics (Width × Length or Diameter) (µm)**	4,0±0,3 × 3,1±0,5 (Cells); Ø 4,3±0,9 (heterocytes)
**Other Code(s)**	LEAN 072	**Taxonomic notes/diacritical features**	cell ultrastructure data available
**Parent Sample**	O.O6 ODA 5 - ABCA	**Macroscopic growth features**	color: greenish; forming a firm biofilm
**Isolation Date**		**Environment**	aquatic, marine
**Order (Older Classification Scheme)**	Nostocales	**Habitat Sample Description**	wave-exposed tide puddle, epilithic
**Order 2014 (Modern Taxonomy)**	Nostocales	**Ecophysiological traits**	nitrogen-fixation
**Taxonomy Notes**		**Notes on ecophysiological traits**	
**Accession Number(s)_16S**	HQ832947	**Isolator**	Vitor Ramos
**Accession Number(s)_others (product/gene /region)**	KC256765 (dinitrogenase reductase, nifH), KC813190, KC813191 (non-ribosomal peptide synthetase), KC842366, KC842367 (polyketide synthase)	**Medium**	BG11o and Z8
**Collector**	Rui Seabra	**Strain Status**	non-axenic, unicyanobacterial
**Collection Date**	2006/12/12	**Preservation type**	subculturing
**Location**	Portugal: Praia de Olhos d'Água, Albufeira	**Light:dark cycle**	14:10-h
**Latitude & Longitude**	37.08973 N 8.191017 W	**Temperature (ºC)**	25
		**Light intensity**	10–30 µmol photons m−2 s−1
**References Publicly Available Articles**	Brito et al. (2012) Syst. Appl. Microbiol. 35, 110–119; Brito et al. (2015) Algal Res. 9, 218-226		
**References Publicly Available Theses**			

**Strain ID**	**Nostoc sp. LEGE 07365**		
**Strain Taxonomy**	Nostoc Vaucher ex Bornet, É & Flahault, C (1886)		
**Microphotograph 400×**	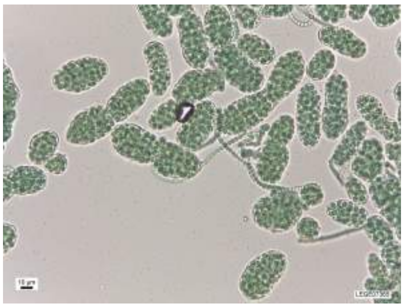	**Microphotograph 1000×**	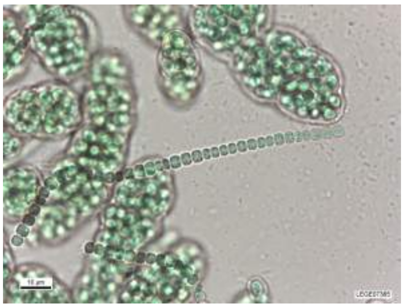
**Coidentity**	n.a.	**Morphometrics (Width × Length or Diameter) (µm)**	3,0±0,3 × 3,3±0,5
**Other Code(s)**		**Taxonomic notes/diacritical features**	sac-like gelatinous thalli
**Parent Sample**	VL MS 250	**Macroscopic growth features**	color: cyano; forming a smooth biofilm and aggregates
**Isolation Date**		**Environment**	aquatic, brackish
**Order (Older Classification Scheme)**	Nostocales	**Habitat Sample Description**	unknown
**Order 2014 (Modern Taxonomy)**	Nostocales	**Ecophysiological traits**	nitrogen-fixation
**Taxonomy Notes**		**Notes on ecophysiological traits**	dehydroabietic acid and abietic acid production (unknown ecophysiological role)
**Accession Number(s)_16S**	New Seq + KT951672	**Isolator**	Viviana Lopes
**Accession Number(s)_others (product/gene /region)**		**Medium**	Z8
**Collector**	Viviana Lopes	**Strain Status**	non-axenic, unicyanobacterial
**Collection Date**	2007	**Preservation type**	subculturing
**Location**	Portugal	**Light:dark cycle**	12:12-h
**Latitude & Longitude**	unknown	**Temperature (ºC)**	19
		**Light intensity**	10–30 µmol photons m−2 s−1
**References Publicly Available Articles**	Costa et al. (2016) Sci. Rep. 6, 23436		
**References Publicly Available Theses**			

**Strain ID**	**Nostoc sp. LEGE 12447**		
**Strain Taxonomy**	Nostoc Vaucher ex Bornet, É & Flahault, C (1886)		
**Microphotograph 400×**	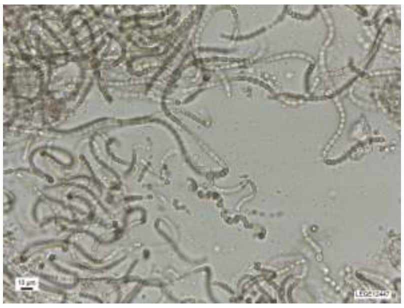	**Microphotograph 1000×**	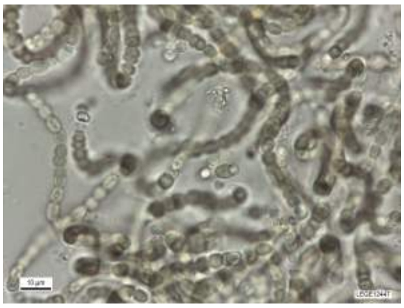
**Coidentity**	n.a.	**Morphometrics (Width × Length or Diameter) (µm)**	3,2±0,5 × 4,1±0,8 (Cells); Ø 3,4±0,8 (Heterocytes); 4,4±0,7 × 5,1±0,6 (Akinetes)
**Other Code(s)**	CR2	**Taxonomic notes/diacritical features**	terminal or intercalar heterocytes; with akinetes
**Parent Sample**		**Macroscopic growth features**	color: olive green; forming aggregates
**Isolation Date**		**Environment**	terrestrial
**Order (Older Classification Scheme)**	Nostocales	**Habitat Sample Description**	cycad coralloid root (host: Cycas revoluta)
**Order 2014 (Modern Taxonomy)**	Nostocales	**Ecophysiological traits**	
**Taxonomy Notes**		**Notes on ecophysiological traits**	
**Accession Number(s)_16S**	KU951781	**Isolator**	Ana L. Pereira
**Accession Number(s)_others (product/gene /region)**		**Medium**	BG11o
**Collector**	Ana L. Pereira	**Strain Status**	non-axenic, unicyanobacterial
**Collection Date**	2012	**Preservation type**	subculturing
**Location**	Portugal: Tropical Botanical Garden of Lisbon	**Light:dark cycle**	12:12-h
**Latitude & Longitude**	38.69819 N 9.203429 W	**Temperature (ºC)**	19
		**Light intensity**	10–30 µmol photons m−2 s−1
**References Publicly Available Articles**			
**References Publicly Available Theses**			

Strain ID	**Nostoc sp. LEGE 12448**		
**Strain Taxonomy**	Nostoc Vaucher ex Bornet, É & Flahault, C (1886)		
**Microphotograph 400×**	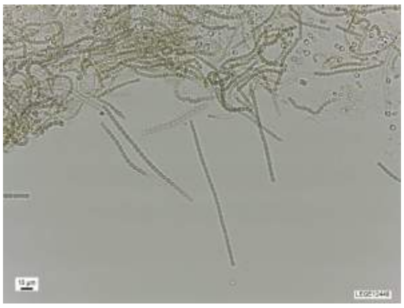	**Microphotograph 1000×**	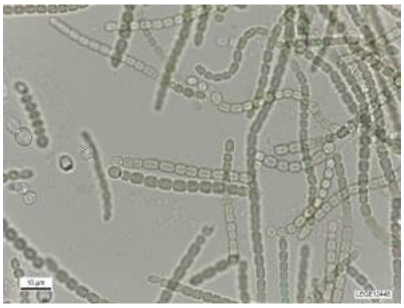
**Coidentity**	n.a.	**Morphometrics (Width × Length or Diameter) (µm)**	2,5±0,3 × 3,4±0,8 (Cells); 3,4±0,6 × 4,2±0,9 (Heterocytes)
**Other Code(s)**	Enc1	**Taxonomic notes/diacritical features**	straight trichomes; with terminal heterocytes
**Parent Sample**		**Macroscopic growth features**	color: olive green; forming a smooth biofilm and aggregates
**Isolation Date**		**Environment**	terrestrial
**Order (Older Classification Scheme)**	Nostocales	**Habitat Sample Description**	cycad coralloid root (host: Encephalartos sp.)
**Order 2014 (Modern Taxonomy)**	Nostocales	**Ecophysiological traits**	
**Taxonomy Notes**		**Notes on ecophysiological traits**	
**Accession Number(s)_16S**	KU951782	**Isolator**	Ana L. Pereira
**Accession Number(s)_others (product/gene /region)**		**Medium**	BG11o
**Collector**	Ana L. Pereira	**Strain Status**	non-axenic, unicyanobacterial
**Collection Date**	2012	**Preservation type**	subculturing
**Location**	Portugal: Botanical Garden of the University of Porto	**Light:dark cycle**	12:12-h
**Latitude & Longitude**	41.15364 N 8.642851 W	**Temperature (ºC)**	19
		**Light intensity**	10–30 µmol photons m−2 s−1
**References Publicly Available Articles**			
**References Publicly Available Theses**			

**Strain ID**	**Nostoc sp. LEGE 12449**		
**Strain Taxonomy**	Nostoc Vaucher ex Bornet, É & Flahault, C (1886)		
**Microphotograph 400×**	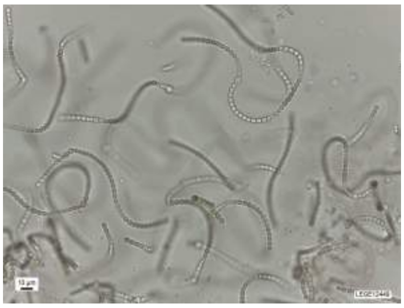	**Microphotograph 1000×**	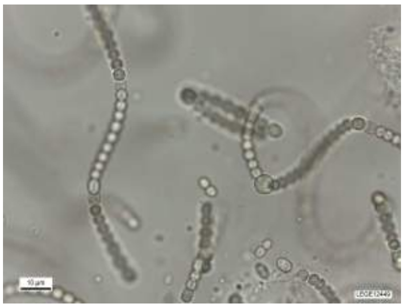
**Coidentity**	n.a.	**Morphometrics (Width × Length or Diameter) (µm)**	3,3±0,4 × 3,6±0,6 (Cells); Ø 4,4±0,4 (Heterocytes)
**Other Code(s)**	Eh1	**Taxonomic notes/diacritical features**	with spherical akinetes; with sheath
**Parent Sample**		**Macroscopic growth features**	color: dark olive green; forming aggregates
**Isolation Date**		**Environment**	terrestrial
**Order (Older Classification Scheme)**	Nostocales	**Habitat Sample Description**	cycad coralloid root (host: Encephalartos horridus)
**Order 2014 (Modern Taxonomy)**	Nostocales	**Ecophysiological traits**	
**Taxonomy Notes**		**Notes on ecophysiological traits**	
**Accession Number(s)_16S**	KU951783	**Isolator**	Ana L. Pereira
**Accession Number(s)_others (product/gene /region)**		**Medium**	BG11o
**Collector**	Ana L. Pereira	**Strain Status**	non-axenic, unicyanobacterial
**Collection Date**	2012	**Preservation type**	subculturing
**Location**	Portugal: Botanical Garden of the University of Coimbra	**Light:dark cycle**	12:12-h
**Latitude & Longitude**	40.20622 N 8.421698 W	**Temperature (ºC)**	19
		**Light intensity**	10–30 µmol photons m−2 s−1
**References Publicly Available Articles**			
**References Publicly Available Theses**			

Strain ID	**Nostoc sp. LEGE 12450**		
**Strain Taxonomy**	Nostoc Vaucher ex Bornet, É & Flahault, C (1886)		
**Microphotograph 400×**	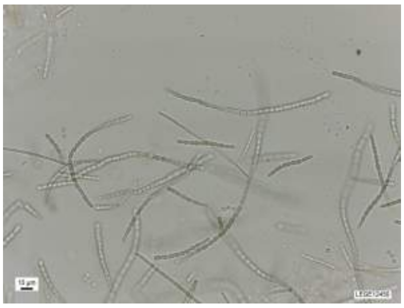	**Microphotograph 1000×**	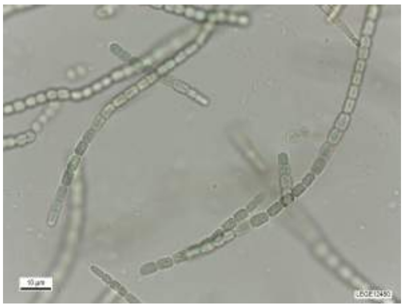
**Coidentity**	n.a.	**Morphometrics (Width × Length or Diameter) (µm)**	2,8±0,4 × 3,9±0,9 (Cells); 3,8±0,5 × 4,7±0,7 (Heterocytes)
**Other Code(s)**	Eh2	**Taxonomic notes/diacritical features**	trichomes forming anastomosis-like structures within sheaths
**Parent Sample**		**Macroscopic growth features**	color: dark olive green; forming aggregates
**Isolation Date**		**Environment**	terrestrial
**Order (Older Classification Scheme)**	Nostocales	**Habitat Sample Description**	cycad coralloid root (host: Encephalartos horridus)
**Order 2014 (Modern Taxonomy)**	Nostocales	**Ecophysiological traits**	
**Taxonomy Notes**		**Notes on ecophysiological traits**	
**Accession Number(s)_16S**	KU951784	**Isolator**	Ana L. Pereira
**Accession Number(s)_others (product/gene /region)**		**Medium**	BG11o
**Collector**	Ana L. Pereira	**Strain Status**	non-axenic, unicyanobacterial
**Collection Date**	2012	**Preservation type**	subculturing, cryopreserved
**Location**	Portugal: Tropical Botanical Garden of Lisbon	**Light:dark cycle**	12:12-h
**Latitude & Longitude**	38.69819 N 9.203429 W	**Temperature (ºC)**	19
		**Light intensity**	10–30 µmol photons m−2 s−1
**References Publicly Available Articles**			
**References Publicly Available Theses**			

**Strain ID**	**Nostoc sp. LEGE 12451**		
**Strain Taxonomy**	Nostoc Vaucher ex Bornet, É & Flahault, C (1886)		
**Microphotograph 400×**	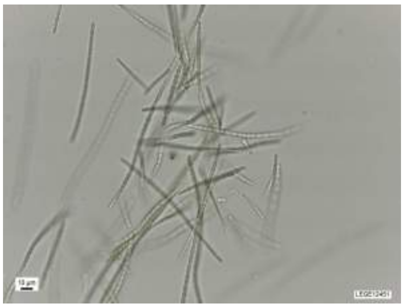	**Microphotograph 1000×**	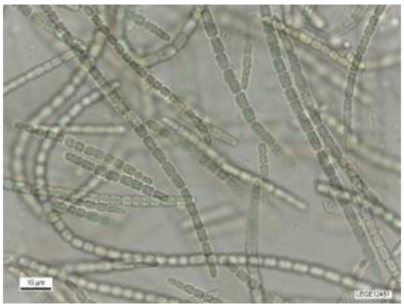
**Coidentity**	n.a.	**Morphometrics (Width × Length or Diameter) (µm)**	2,5±0,4 × 3,8±0,8 (Cells); 3,2±1,5 × 4,5±0,6 (Heterocytes); 3,4±0,3 × 5,7±0,6 (Akinetes)
**Other Code(s)**	Ep1	**Taxonomic notes/diacritical features**	trichomes forming anastomosis-like structures within sheaths; with intercalar heterocytes
**Parent Sample**		**Macroscopic growth features**	color: olive green; forming aggregates
**Isolation Date**		**Environment**	terrestrial
**Order (Older Classification Scheme)**	Nostocales	**Habitat Sample Description**	cycad coralloid root (host: Encephalartos paucidentatus)
**Order 2014 (Modern Taxonomy)**	Nostocales	**Ecophysiological traits**	
**Taxonomy Notes**		**Notes on ecophysiological traits**	
**Accession Number(s)_16S**	KU951785	**Isolator**	Ana L. Pereira
**Accession Number(s)_others (product/gene /region)**		**Medium**	BG11o
**Collector**	Ana L. Pereira	**Strain Status**	non-axenic, unicyanobacterial
**Collection Date**	2012	**Preservation type**	subculturing, cryopreserved
**Location**	Portugal: Tropical Botanical Garden of Lisbon	**Light:dark cycle**	12:12-h
**Latitude & Longitude**	38.69819 N 9.203429 W	**Temperature (ºC)**	19
		**Light intensity**	10–30 µmol photons m−2 s−1
**References Publicly Available Articles**			
**References Publicly Available Theses**			

Strain ID	**Nostoc sp. LEGE 12453**		
**Strain Taxonomy**	Nostoc Vaucher ex Bornet, É & Flahault, C (1886)		
**Microphotograph 400×**	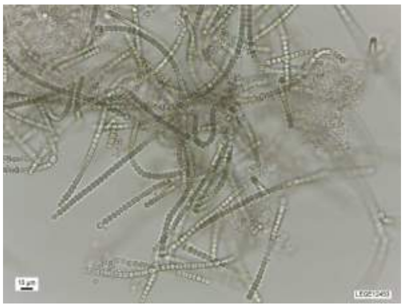	**Microphotograph 1000×**	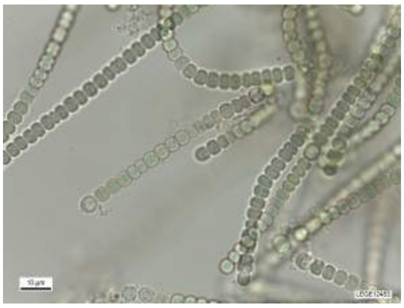
**Coidentity**	n.a.	**Morphometrics (Width × Length or Diameter) (µm)**	3,8±0,3 × 3,3±0,5 (Cells); Ø 4,2±0,6 (Heterocytes)
**Other Code(s)**	Ea1	**Taxonomic notes/diacritical features**	barrel-shaped cells
**Parent Sample**		**Macroscopic growth features**	color: greenish; forming aggregates
**Isolation Date**		**Environment**	terrestrial
**Order (Older Classification Scheme)**	Nostocales	**Habitat Sample Description**	cycad coralloid root (host: Encephalartos altensteinii)
**Order 2014 (Modern Taxonomy)**	Nostocales	**Ecophysiological traits**	
**Taxonomy Notes**		**Notes on ecophysiological traits**	
**Accession Number(s)_16S**	KU951786	**Isolator**	Ana L. Pereira
**Accession Number(s)_others (product/gene /region)**		**Medium**	BG11o
**Collector**	Ana L. Pereira	**Strain Status**	non-axenic, unicyanobacterial
**Collection Date**	2012	**Preservation type**	subculturing
**Location**	Portugal: Tropical Botanical Garden of Lisbon	**Light:dark cycle**	12:12-h
**Latitude & Longitude**	38.69819 N 9.203429 W	**Temperature (ºC)**	19
		**Light intensity**	10–30 µmol photons m−2 s−1
**References Publicly Available Articles**			
**References Publicly Available Theses**			

Strain ID	**Nostoc sp. LEGE 12454**		
**Strain Taxonomy**	Nostoc Vaucher ex Bornet, É & Flahault, C (1886)		
**Microphotograph 400×**	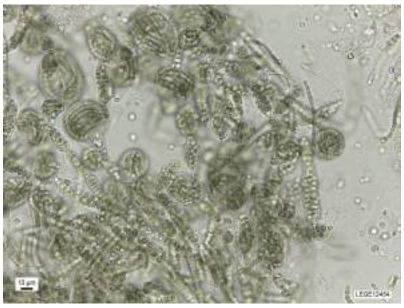	**Microphotograph 1000×**	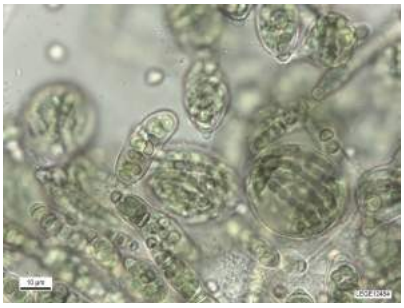
**Coidentity**	n.a.	**Morphometrics (Width × Length or Diameter) (µm)**	3,0±0,5 × 3,3±0,8 (Cells); Ø 3,6±0,2 (Heterocytes)
**Other Code(s)**	EL1	**Taxonomic notes/diacritical features**	sac-like gelatinous thalli; with sheath
**Parent Sample**		**Macroscopic growth features**	color: olive green; forming aggregates
**Isolation Date**		**Environment**	terrestrial
**Order (Older Classification Scheme)**	Nostocales	**Habitat Sample Description**	cycad coralloid root (host: Encephalartos lebomboensis)
**Order 2014 (Modern Taxonomy)**	Nostocales	**Ecophysiological traits**	
**Taxonomy Notes**		**Notes on ecophysiological traits**	
**Accession Number(s)_16S**	KU951787	**Isolator**	Ana L. Pereira
**Accession Number(s)_others (product/gene /region)**		**Medium**	BG11o
**Collector**	Ana L. Pereira	**Strain Status**	non-axenic, unicyanobacterial
**Collection Date**	2012	**Preservation type**	subculturing
**Location**	Portugal: Tropical Botanical Garden of Lisbon	**Light:dark cycle**	12:12-h
**Latitude & Longitude**	38.69819 N 9.203429 W	**Temperature (ºC)**	19
		**Light intensity**	10–30 µmol photons m−2 s−1
**References Publicly Available Articles**			
**References Publicly Available Theses**			

**Strain ID**	**Nostoc sp. LEGE 12456**		
**Strain Taxonomy**	Nostoc Vaucher ex Bornet, É & Flahault, C (1886)		
**Microphotograph 400×**	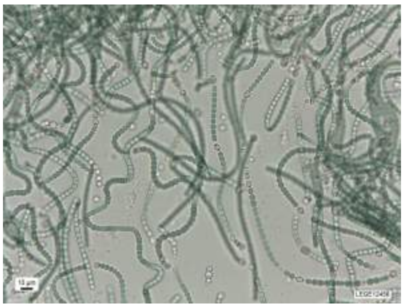	**Microphotograph 1000×**	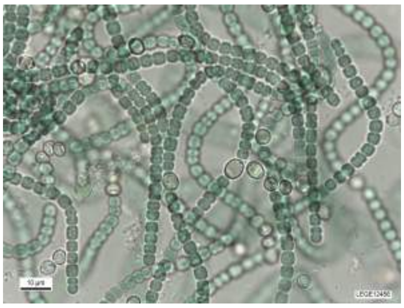
**Coidentity**	n.a.	**Morphometrics (Width × Length or Diameter) (µm)**	3,5±0,5 × 3,9±0,5 (Cells); Ø 4,1±0,4 (Heterocytes)
**Other Code(s)**	ELeh2	**Taxonomic notes/diacritical features**	barrel-shaped cells
**Parent Sample**		**Macroscopic growth features**	color: cyano; forming aggregates
**Isolation Date**		**Environment**	terrestrial
**Order (Older Classification Scheme)**	Nostocales	**Habitat Sample Description**	cycad coralloid root (host: Encephalartos lebomboensis)
**Order 2014 (Modern Taxonomy)**	Nostocales	**Ecophysiological traits**	
**Taxonomy Notes**		**Notes on ecophysiological traits**	
**Accession Number(s)_16S**	KU951788	**Isolator**	Ana L. Pereira
**Accession Number(s)_others (product/gene /region)**		**Medium**	BG11o
**Collector**	Ana L. Pereira	**Strain Status**	non-axenic, unicyanobacterial
**Collection Date**	2012	**Preservation type**	subculturing
**Location**	Portugal: Tropical Botanical Garden of Lisbon	**Light:dark cycle**	12:12-h
**Latitude & Longitude**	38.69819 N 9.203429 W	**Temperature (ºC)**	19
		**Light intensity**	10–30 µmol photons m−2 s−1
**References Publicly Available Articles**			
**References Publicly Available Theses**			

**Strain ID**	**Nostoc sp. LEGE 13413**		
**Strain Taxonomy**	Nostoc Vaucher ex Bornet, É & Flahault, C (1886)		
**Microphotograph 400×**	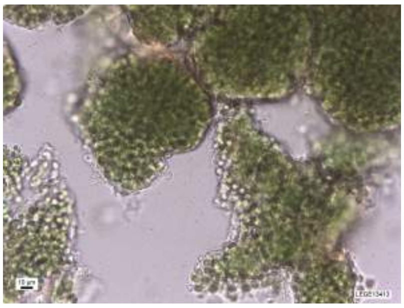	**Microphotograph 1000×**	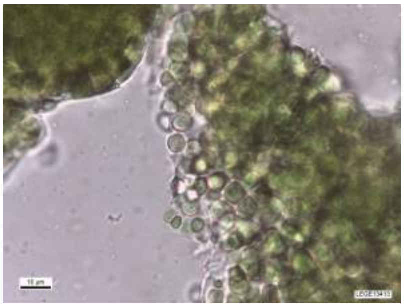
**Coidentity**	n.a.	**Morphometrics (Width × Length or Diameter) (µm)**	5,5±0,7 × 5,2±0,7
**Other Code(s)**		**Taxonomic notes/diacritical features**	large colonies of filaments, within mucilaginous envelopes; with sheath
**Parent Sample**	S5F sal	**Macroscopic growth features**	color: cyano; forming aggregates
**Isolation Date**		**Environment**	unknown
**Order (Older Classification Scheme)**	Nostocales	**Habitat Sample Description**	"Spirulina" dietary supplements, tablet form. Producer/brand: Biover
**Order 2014 (Modern Taxonomy)**	Nostocales	**Ecophysiological traits**	nitrogen-fixation
**Taxonomy Notes**		**Notes on ecophysiological traits**	
**Accession Number(s)_16S**		**Isolator**	Dina Gomes
**Accession Number(s)_others (product/gene /region)**		**Medium**	Z8 25‰ TM sea salt w/ vit. B12
**Collector**	Dina Gomes	**Strain Status**	non-axenic, unicyanobacterial
**Collection Date**	2013	**Preservation type**	subculturing
**Location**	original source unknown: acquired in the Porto metropolitan area, Portugal	**Light:dark cycle**	12:12-h
**Latitude & Longitude**	unknown	**Temperature (ºC)**	19
		**Light intensity**	10–30 µmol photons m−2 s−1
**References Publicly Available Articles**			
**References Publicly Available Theses**	Gomes D. (2013) MSc Dissertation, ICBAS, University of Porto, Porto, Portugal		

**Strain ID**	**Phormidium cf. irriguum LEGE 00055**		
**Strain Taxonomy**	Phormidium irriguum (Kützing ex Gomont) Anagnostidis, K & Komárek, J (1988)		
**Microphotograph 400×**	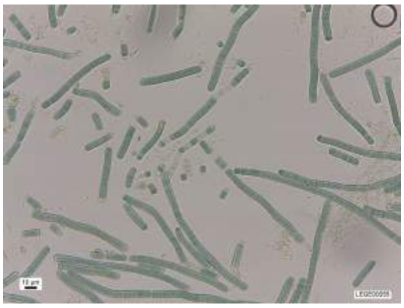	**Microphotograph 1000×**	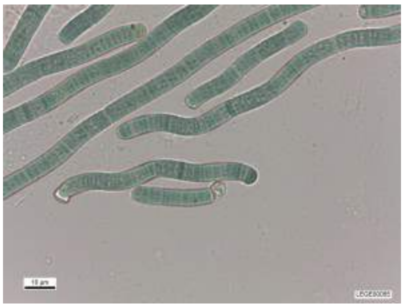
**Coidentity**	n.a.	**Morphometrics (Width × Length or Diameter) (µm)**	5,4±0,4 × 2,5±0,6
**Other Code(s)**		**Taxonomic notes/diacritical features**	with sheath
**Parent Sample**	Infout Osc I	**Macroscopic growth features**	color: cyano; forming a firm biofilm and aggregates
**Isolation Date**		**Environment**	aquatic, freshwater
**Order (Older Classification Scheme)**	Oscillatoriales	**Habitat Sample Description**	water sample
**Order 2014 (Modern Taxonomy)**	Oscillatoriales	**Ecophysiological traits**	
**Taxonomy Notes**		**Notes on ecophysiological traits**	
**Accession Number(s)_16S**	KU951790	**Isolator**	Martin Saker
**Accession Number(s)_others (product/gene /region)**		**Medium**	Z8
**Collector**	unknown	**Strain Status**	non-axenic, unicyanobacterial
**Collection Date**	2000	**Preservation type**	subculturing
**Location**	Morocco: Imfout reservoir, Oum Rabiaa basin	**Light:dark cycle**	12:12-h
**Latitude & Longitude**	unknown	**Temperature (ºC)**	19
		**Light intensity**	10–30 µmol photons m−2 s−1
**References Publicly Available Articles**			
**References Publicly Available Theses**			

Strain ID	**Phormidium laetevirens LEGE 06103**		
**Strain Taxonomy**	Phormidium laetevirens (Crouan, P & Crouan, H ex Gomont) Anagnostidis, K & Komárek, J (1988)		
**Microphotograph 400×**	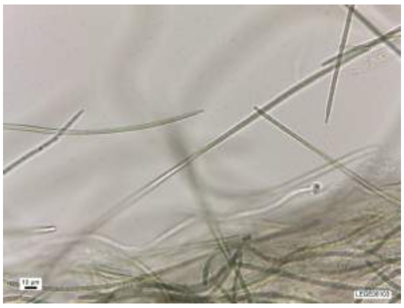	**Microphotograph 1000×**	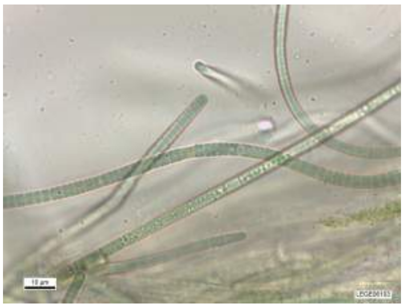
**Coidentity**	n.a.	**Morphometrics (Width × Length or Diameter) (µm)**	3,5±0,5 × 2,0±0,4
**Other Code(s)**	LEAN 007	**Taxonomic notes/diacritical features**	gliding motility
**Parent Sample**	V.06 LUZ 11 - ABE	**Macroscopic growth features**	color: olive green; forming a leather-like biofilm
**Isolation Date**		**Environment**	aquatic, marine
**Order (Older Classification Scheme)**	Oscillatoriales	**Habitat Sample Description**	tide puddle, rock surface scraping
**Order 2014 (Modern Taxonomy)**	undefined	**Ecophysiological traits**	
**Taxonomy Notes**		**Notes on ecophysiological traits**	
**Accession Number(s)_16S**	JF708120	**Isolator**	Vitor Ramos
**Accession Number(s)_others (product/gene /region)**		**Medium**	Z8 25‰ TM sea salt w/ vit. B12
**Collector**	Rui Seabra	**Strain Status**	non-axenic, unicyanobacterial
**Collection Date**	2006/07/17	**Preservation type**	subculturing
**Location**	Portugal: Praia da Luz, Lagos	**Light:dark cycle**	14:10-h
**Latitude & Longitude**	37.08622 N 8.725375 W	**Temperature (ºC)**	25
		**Light intensity**	10–30 µmol photons m−2 s−1
**References Publicly Available Articles**	Brito et al. (2012) Syst. Appl. Microbiol. 35, 110–119		
**References Publicly Available Theses**			

**Strain ID**	**Phormidium sp. LEGE 00064**		
**Strain Taxonomy**	Phormidium Kützing ex Gomont (1892)		
**Microphotograph 400×**	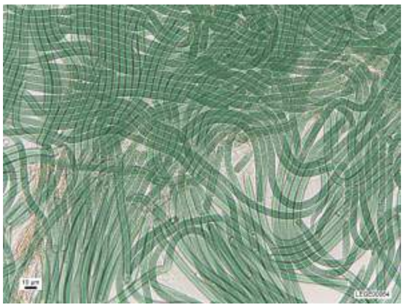	**Microphotograph 1000×**	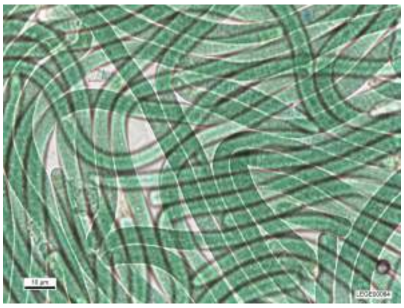
**Coidentity**	n.a.	**Morphometrics (Width × Length or Diameter) (µm)**	4,8±0,6 × 3,0±1,3
**Other Code(s)**		**Taxonomic notes/diacritical features**	some capitate terminal cells
**Parent Sample**	Site2 Osc II	**Macroscopic growth features**	color: cyano; forming a smooth biofilm and aggregates
**Isolation Date**		**Environment**	aquatic, freshwater
**Order (Older Classification Scheme)**	Oscillatoriales	**Habitat Sample Description**	unknown
**Order 2014 (Modern Taxonomy)**	not determined	**Ecophysiological traits**	
**Taxonomy Notes**		**Notes on ecophysiological traits**	
**Accession Number(s)_16S**		**Isolator**	Martin Saker
**Accession Number(s)_others (product/gene /region)**		**Medium**	Z8
**Collector**	unknown	**Strain Status**	non-axenic, unicyanobacterial
**Collection Date**	2000	**Preservation type**	subculturing
**Location**	Morocco: Lake Takerkoust	**Light:dark cycle**	12:12-h
**Latitude & Longitude**	unknown	**Temperature (ºC)**	19
		**Light intensity**	10–30 µmol photons m−2 s−1
**References Publicly Available Articles**			
**References Publicly Available Theses**			

**Strain ID**	**Phormidium sp. LEGE 00065**		
**Strain Taxonomy**	Phormidium Kützing ex Gomont (1892)		
**Microphotograph 400×**	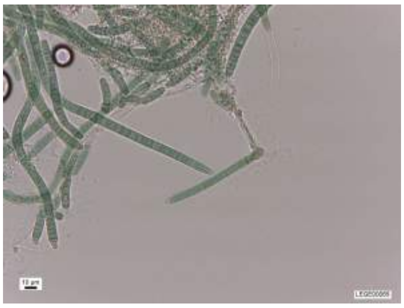	**Microphotograph 1000×**	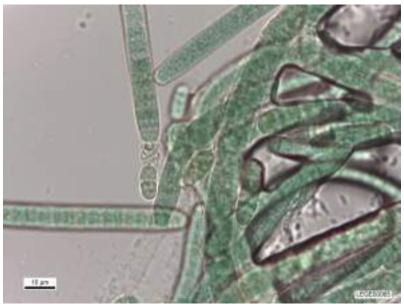
**Coidentity**	n.a.	**Morphometrics (Width × Length or Diameter) (µm)**	7,5±1,5 × 3,5±0,7
**Other Code(s)**	Site Big 4	**Taxonomic notes/diacritical features**	terminal cells sometimes capitated or showing thickness of outer wall/calyptra(?); hormogonia; with sheath
**Parent Sample**	Site4 Big	**Macroscopic growth features**	color: cyano; forming a smooth biofilm and aggregates
**Isolation Date**		**Environment**	aquatic, freshwater
**Order (Older Classification Scheme)**	Oscillatoriales	**Habitat Sample Description**	unknown
**Order 2014 (Modern Taxonomy)**	not determined	**Ecophysiological traits**	
**Taxonomy Notes**		**Notes on ecophysiological traits**	
**Accession Number(s)_16S**		**Isolator**	Martin Saker
**Accession Number(s)_others (product/gene /region)**		**Medium**	Z8
**Collector**	unknown	**Strain Status**	non-axenic, unicyanobacterial
**Collection Date**	2000	**Preservation type**	subculturing
**Location**	Morocco: Oukaïmeden, reservoir	**Light:dark cycle**	12:12-h
**Latitude & Longitude**	unknown	**Temperature (ºC)**	19
		**Light intensity**	10–30 µmol photons m−2 s−1
**References Publicly Available Articles**	Leão et al. (2009) Eur. J. Phycol. 44, 347-355		
**References Publicly Available Theses**	Leão P.N. (2010) PhD Thesis, FCUP, University of Porto, Porto, Portugal		

Strain ID	**Phormidium sp. LEGE 05292**		
**Strain Taxonomy**	Phormidium Kützing ex Gomont (1892)		
**Microphotograph 400×**	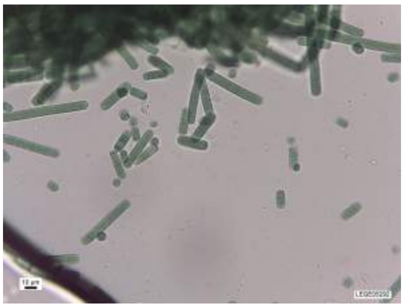	**Microphotograph 1000×**	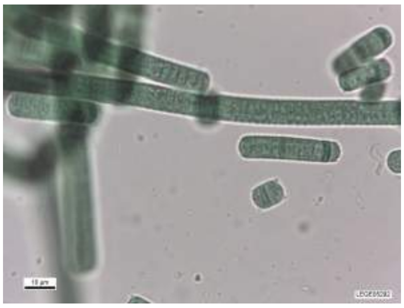
**Coidentity**	n.a.	**Morphometrics (Width × Length or Diameter) (µm)**	7,1±0,5 × 2,6±0,3
**Other Code(s)**	OSC, OSC AP 1	**Taxonomic notes/diacritical features**	with necridic cells and hormogonia; with sheath
**Parent Sample**		**Macroscopic growth features**	color: cyano; forming a firm biofilm
**Isolation Date**		**Environment**	aquatic, freshwater
**Order (Older Classification Scheme)**	Oscillatoriales	**Habitat Sample Description**	biofilm
**Order 2014 (Modern Taxonomy)**	Oscillatoriales	**Ecophysiological traits**	allelopathy
**Taxonomy Notes**		**Notes on ecophysiological traits**	portoamides production
**Accession Number(s)_16S**	GU085101	**Isolator**	Joana Martins
**Accession Number(s)_others (product/gene /region)**		**Medium**	Z8
**Collector**	António P. Carvalho	**Strain Status**	non-axenic, unicyanobacterial
**Collection Date**	2005	**Preservation type**	subculturing
**Location**	Portugal: on an indoor aquarium wall, at Porto	**Light:dark cycle**	12:12-h
**Latitude & Longitude**	unknown	**Temperature (ºC)**	19
		**Light intensity**	10–30 µmol photons m−2 s−1
**References Publicly Available Articles**	Leão et al. (2009) Eur. J. Phycol. 44, 347-355; Leão et al. (2010) Proc. Natl. Acad. Sci. U.S.A. 107 (25), 11183-11188; Leão et al. (2012) Microb. Ecol. 63, 85–95; Barreiro & Vasconcelos (2014) Aquat. Ecol. 48(2), 191-205		
**References Publicly Available Theses**	Leão P.N. (2010) PhD Thesis, FCUP, University of Porto, Porto, Portugal; Ribeiro T.A. (2014) MSc Dissertation, Department of Chemistry, University of Aveiro, Aveiro, Portugal		

Strain ID	**Planktothrix mougeotii LEGE 06222**		
**Strain Taxonomy**	Planktothrix mougeotii (Kützing ex Lemmermann) Suda, S; Watanabe, M; Otsuka, S; Mahakahant, A; Yongmanitchai, W; Nopartnaraporn, N; Liu, Y & Day, JG (2002)		
**Microphotograph 400×**	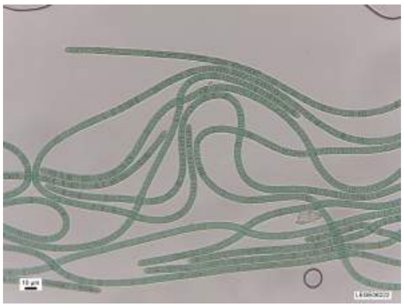	**Microphotograph 1000×**	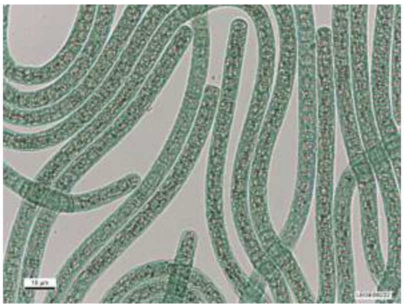
**Coidentity**	n.a.	**Morphometrics (Width × Length or Diameter) (µm)**	5,3±0,3 × 3,3±0,5
**Other Code(s)**		**Taxonomic notes/diacritical features**	trichomes with vacuolated cells, hormogonia and necridic cells; facultative sheath
**Parent Sample**	A3 24/10 - 5	**Macroscopic growth features**	color: cyano; forming a smooth biofilm and tuft-like aggregates
**Isolation Date**		**Environment**	aquatic, freshwater
**Order (Older Classification Scheme)**	Oscillatoriales	**Habitat Sample Description**	biofilm, from a secondary decanter tank bank
**Order 2014 (Modern Taxonomy)**	undefined	**Ecophysiological traits**	
**Taxonomy Notes**		**Notes on ecophysiological traits**	
**Accession Number(s)_16S**	KU951791	**Isolator**	Joana Martins
**Accession Number(s)_others (product/gene /region)**		**Medium**	Z8
**Collector**	Joana Martins	**Strain Status**	non-axenic, unicyanobacterial
**Collection Date**	2006/10/27	**Preservation type**	subculturing, cryopreserved
**Location**	Portugal: wastewater treatment plant at Febros river, Vila Nova de Gaia	**Light:dark cycle**	12:12-h
**Latitude & Longitude**	41.11812 N 8.570944 W	**Temperature (ºC)**	19
		**Light intensity**	10–30 µmol photons m−2 s−1
**References Publicly Available Articles**			
**References Publicly Available Theses**	Macieira R. (2013) MSc Dissertation, FCUP, University of Porto, Porto, Portugal		

Strain ID	**Planktothrix mougeotii LEGE 06223**		
**Strain Taxonomy**	Planktothrix mougeotii (Kützing ex Lemmermann) Suda, S; Watanabe, M; Otsuka, S; Mahakahant, A; Yongmanitchai, W; Nopartnaraporn, N; Liu, Y & Day, JG (2002)		
**Microphotograph 400×**	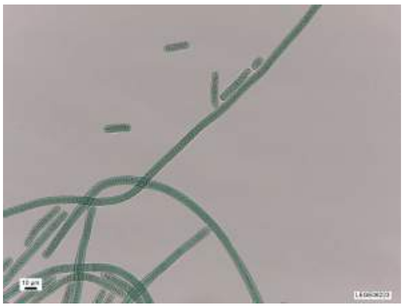	**Microphotograph 1000×**	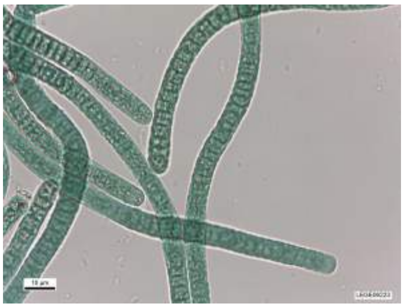
**Coidentity**	n.a.	**Morphometrics (Width × Length or Diameter) (µm)**	6,0±0,4 × 2,8±0,4
**Other Code(s)**		**Taxonomic notes/diacritical features**	trichomes with vacuolated cells, hormogonia and necridic cells; facultative sheath
**Parent Sample**	A5 24/11 - 11	**Macroscopic growth features**	color: cyano; forming a smooth biofilm and tuft-like aggregates
**Isolation Date**		**Environment**	aquatic, freshwater
**Order (Older Classification Scheme)**	Oscillatoriales	**Habitat Sample Description**	floating masses, from a secondary decanter tank bank
**Order 2014 (Modern Taxonomy)**	undefined	**Ecophysiological traits**	
**Taxonomy Notes**		**Notes on ecophysiological traits**	
**Accession Number(s)_16S**	KU951792	**Isolator**	Joana Martins
**Accession Number(s)_others (product/gene /region)**		**Medium**	Z8
**Collector**	Joana Martins	**Strain Status**	non-axenic, unicyanobacterial
**Collection Date**	24-Nov-2006	**Preservation type**	subculturing
**Location**	Portugal: wastewater treatment plant at Febros river, Vila Nova de Gaia	**Light:dark cycle**	12:12-h
**Latitude & Longitude**	41.11812 N 8.570944 W	**Temperature (ºC)**	19
		**Light intensity**	10–30 µmol photons m−2 s−1
**References Publicly Available Articles**			
**References Publicly Available Theses**			

**Strain ID**	**Planktothrix mougeotii LEGE 06225**		
**Strain Taxonomy**	Planktothrix mougeotii (Kützing ex Lemmermann) Suda, S; Watanabe, M; Otsuka, S; Mahakahant, A; Yongmanitchai, W; Nopartnaraporn, N; Liu, Y & Day, JG (2002)		
**Microphotograph 400×**	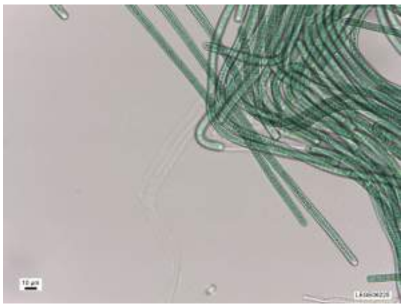	**Microphotograph 1000×**	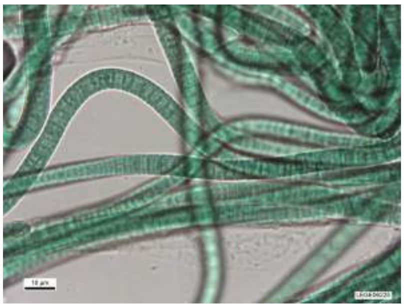
**Coidentity**	n.a.	**Morphometrics (Width × Length or Diameter) (µm)**	6,5±1,0 × 3,1±0,4
**Other Code(s)**		**Taxonomic notes/diacritical features**	trichomes with vacuolated cells, hormogonia and necridic cells; facultative sheath
**Parent Sample**	A5 24/10 - 14	**Macroscopic growth features**	color: cyano; forming a smooth biofilm and aggregates
**Isolation Date**		**Environment**	aquatic, freshwater
**Order (Older Classification Scheme)**	Oscillatoriales	**Habitat Sample Description**	floating masses, from a secondary decanter tank bank
**Order 2014 (Modern Taxonomy)**	undefined	**Ecophysiological traits**	
**Taxonomy Notes**		**Notes on ecophysiological traits**	
**Accession Number(s)_16S**	KU951793	**Isolator**	Joana Martins
**Accession Number(s)_others (product/gene /region)**		**Medium**	Z8
**Collector**	Joana Martins	**Strain Status**	non-axenic, unicyanobacterial
**Collection Date**	2006/10/27	**Preservation type**	subculturing, cryopreserved
**Location**	Portugal: wastewater treatment plant at Febros river, Vila Nova de Gaia	**Light:dark cycle**	12:12-h
**Latitude & Longitude**	41.11812 N 8.570944 W	**Temperature (ºC)**	19
		**Light intensity**	10–30 µmol photons m−2 s−1
**References Publicly Available Articles**			
**References Publicly Available Theses**			

Strain ID	**Planktothrix mougeotii LEGE 06226**		
**Strain Taxonomy**	Planktothrix mougeotii (Kützing ex Lemmermann) Suda, S; Watanabe, M; Otsuka, S; Mahakahant, A; Yongmanitchai, W; Nopartnaraporn, N; Liu, Y & Day, JG (2002)		
**Microphotograph 400×**	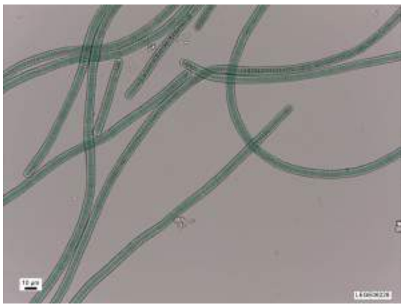	**Microphotograph 1000×**	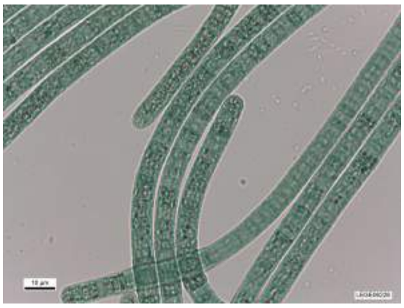
**Coidentity**	n.a.	**Morphometrics (Width × Length or Diameter) (µm)**	6,7±0,6 × 2,5±0,3
**Other Code(s)**		**Taxonomic notes/diacritical features**	trichomes with vacuolated cells, hormogonia and necridic cells; facultative sheath
**Parent Sample**	A5 - 9	**Macroscopic growth features**	color: cyano; forming a smooth biofilm and aggregates
**Isolation Date**		**Environment**	aquatic, freshwater
**Order (Older Classification Scheme)**	Oscillatoriales	**Habitat Sample Description**	floating masses, from a secondary decanter tank bank
**Order 2014 (Modern Taxonomy)**	undefined	**Ecophysiological traits**	
**Taxonomy Notes**		**Notes on ecophysiological traits**	
**Accession Number(s)_16S**	KU951794	**Isolator**	Joana Martins
**Accession Number(s)_others (product/gene /region)**		**Medium**	Z8
**Collector**	Joana Martins	**Strain Status**	non-axenic, unicyanobacterial
**Collection Date**	Oct-2006	**Preservation type**	subculturing, cryopreserved
**Location**	Portugal: wastewater treatment plant at Febros river, Vila Nova de Gaia	**Light:dark cycle**	12:12-h
**Latitude & Longitude**	41.11812 N 8.570944 W	**Temperature (ºC)**	19
		**Light intensity**	10–30 µmol photons m−2 s−1
**References Publicly Available Articles**			
**References Publicly Available Theses**			

Strain ID	**Planktothrix mougeotii LEGE 07229**		
**Strain Taxonomy**	Planktothrix mougeotii (Kützing ex Lemmermann) Suda, S; Watanabe, M; Otsuka, S; Mahakahant, A; Yongmanitchai, W; Nopartnaraporn, N; Liu, Y & Day, JG (2002)		
**Microphotograph 400×**	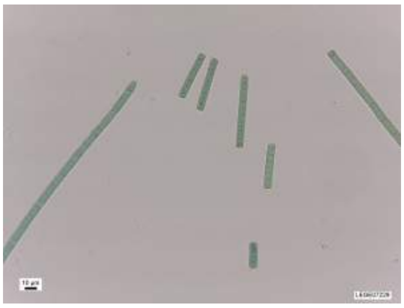	**Microphotograph 1000×**	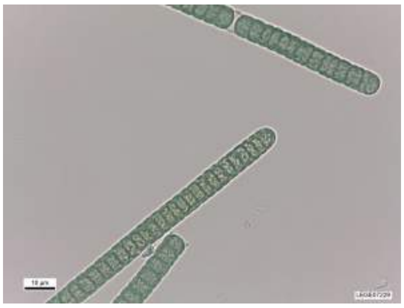
**Coidentity**	n.a.	**Morphometrics (Width × Length or Diameter) (µm)**	7,0±1,2 × 3,1±0,6
**Other Code(s)**		**Taxonomic notes/diacritical features**	trichomes with vacuolated cells, hormogonia and necridic cells; facultative sheath
**Parent Sample**	A5 JL - 4	**Macroscopic growth features**	color: cyano; forming a smooth biofilm and tuft-like aggregates
**Isolation Date**		**Environment**	aquatic, freshwater
**Order (Older Classification Scheme)**	Oscillatoriales	**Habitat Sample Description**	floating masses, from a secondary decanter tank bank
**Order 2014 (Modern Taxonomy)**	undefined	**Ecophysiological traits**	
**Taxonomy Notes**		**Notes on ecophysiological traits**	
**Accession Number(s)_16S**		**Isolator**	Joana Martins
**Accession Number(s)_others (product/gene /region)**		**Medium**	Z8
**Collector**	Joana Martins	**Strain Status**	non-axenic, unicyanobacterial
**Collection Date**	Jul-2007	**Preservation type**	subculturing, cryopreserved
**Location**	Portugal: wastewater treatment plant at Febros river, Vila Nova de Gaia	**Light:dark cycle**	12:12-h
**Latitude & Longitude**	41.11812 N 8.570944 W	**Temperature (ºC)**	19
		**Light intensity**	10–30 µmol photons m−2 s−1
**References Publicly Available Articles**			
**References Publicly Available Theses**			

Strain ID	**Planktothrix mougeotii LEGE 07230**		
**Strain Taxonomy**	Planktothrix mougeotii (Kützing ex Lemmermann) Suda, S; Watanabe, M; Otsuka, S; Mahakahant, A; Yongmanitchai, W; Nopartnaraporn, N; Liu, Y & Day, JG (2002)		
**Microphotograph 400×**	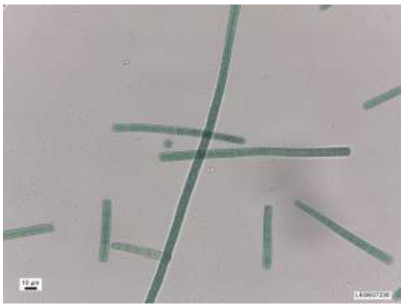	**Microphotograph 1000×**	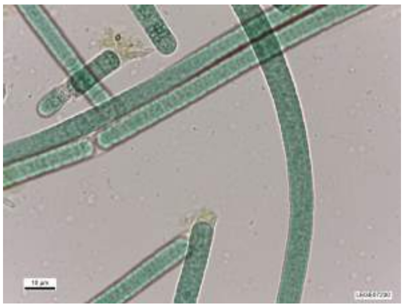
**Coidentity**	n.a.	**Morphometrics (Width × Length or Diameter) (µm)**	6,5±0,8 × 2,4±0,4
**Other Code(s)**		**Taxonomic notes/diacritical features**	trichomes with vacuolated cells, hormogonia and necridic cells; facultative sheath
**Parent Sample**	A5 JL - 5	**Macroscopic growth features**	color: cyano; forming a smooth biofilm and aggregates
**Isolation Date**		**Environment**	aquatic, freshwater
**Order (Older Classification Scheme)**	Oscillatoriales	**Habitat Sample Description**	floating masses, from a secondary decanter tank bank
**Order 2014 (Modern Taxonomy)**	undefined	**Ecophysiological traits**	
**Taxonomy Notes**		**Notes on ecophysiological traits**	
**Accession Number(s)_16S**		**Isolator**	Joana Martins
**Accession Number(s)_others (product/gene /region)**		**Medium**	Z8
**Collector**	Joana Martins	**Strain Status**	non-axenic, unicyanobacterial
**Collection Date**	Jul-2007	**Preservation type**	subculturing
**Location**	Portugal: wastewater treatment plant at Febros river, Vila Nova de Gaia	**Light:dark cycle**	12:12-h
**Latitude & Longitude**	41.11812 N 8.570944 W	**Temperature (ºC)**	19
		**Light intensity**	10–30 µmol photons m−2 s−1
**References Publicly Available Articles**			
**References Publicly Available Theses**			

Strain ID	**Planktothrix mougeotii LEGE 07231**		
**Strain Taxonomy**	Planktothrix mougeotii (Kützing ex Lemmermann) Suda, S; Watanabe, M; Otsuka, S; Mahakahant, A; Yongmanitchai, W; Nopartnaraporn, N; Liu, Y & Day, JG (2002)		
**Microphotograph 400×**	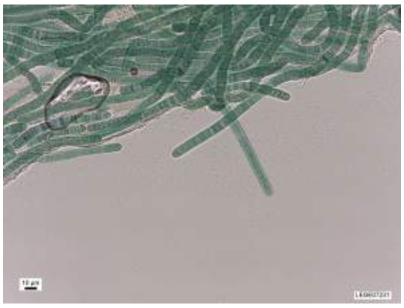	**Microphotograph 1000×**	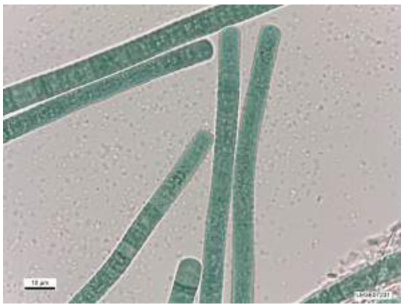
**Coidentity**	n.a.	**Morphometrics (Width × Length or Diameter) (µm)**	6,5±1,0 × 2,3±0,4
**Other Code(s)**		**Taxonomic notes/diacritical features**	trichomes with vacuolated cells, hormogonia and necridic cells; facultative sheath
**Parent Sample**	A5 J5	**Macroscopic growth features**	color: cyano; forming a smooth biofilm
**Isolation Date**		**Environment**	aquatic, freshwater
**Order (Older Classification Scheme)**	Oscillatoriales	**Habitat Sample Description**	floating masses, from a secondary decanter tank bank
**Order 2014 (Modern Taxonomy)**	undefined	**Ecophysiological traits**	
**Taxonomy Notes**		**Notes on ecophysiological traits**	
**Accession Number(s)_16S**	KU951795	**Isolator**	Joana Martins
**Accession Number(s)_others (product/gene /region)**		**Medium**	Z8
**Collector**	Joana Martins	**Strain Status**	non-axenic, unicyanobacterial
**Collection Date**	Jun-2007	**Preservation type**	subculturing
**Location**	Portugal: wastewater treatment plant at Febros river, Vila Nova de Gaia	**Light:dark cycle**	12:12-h
**Latitude & Longitude**	41.11812 N 8.570944 W	**Temperature (ºC)**	19
		**Light intensity**	10–30 µmol photons m−2 s−1
**References Publicly Available Articles**			
**References Publicly Available Theses**			

Strain ID	**Planktothrix sp. LEGE XX280**		
**Strain Taxonomy**	Planktothrix Anagnostidis, K & Komárek, J (1988)		
**Microphotograph 400×**	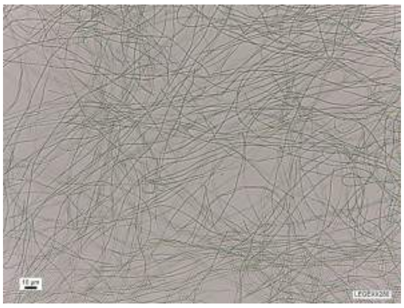	**Microphotograph 1000×**	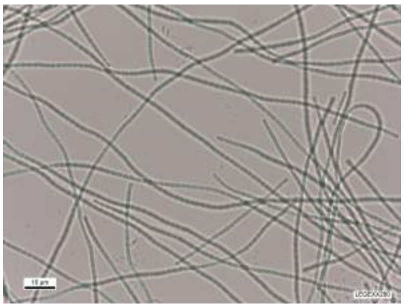
**Coidentity**	n.a.	**Morphometrics (Width × Length or Diameter) (µm)**	1,3±0,3 × 1,9±0,3
**Other Code(s)**	PP	**Taxonomic notes/diacritical features**	
**Parent Sample**		**Macroscopic growth features**	color: cyano; growing homogeneously; mucilaginous
**Isolation Date**		**Environment**	aquatic, freshwater
**Order (Older Classification Scheme)**	Oscillatoriales	**Habitat Sample Description**	unknown
**Order 2014 (Modern Taxonomy)**	not determined	**Ecophysiological traits**	EPS production
**Taxonomy Notes**		**Notes on ecophysiological traits**	
**Accession Number(s)_16S**		**Isolator**	Joana Osswald
**Accession Number(s)_others (product/gene /region)**		**Medium**	
**Collector**	Joana Osswald	**Strain Status**	non-axenic, unicyanobacterial
**Collection Date**	unknown	**Preservation type**	subculturing
**Location**	Portugal: Guadiana river	**Light:dark cycle**	12:12-h
**Latitude & Longitude**	unknown	**Temperature (ºC)**	19
		**Light intensity**	10–30 µmol photons m−2 s−1
**References Publicly Available Articles**	Leão et al. (2009) Eur. J. Phycol. 44, 347-355		
**References Publicly Available Theses**	Leão P.N. (2010) PhD Thesis, FCUP, University of Porto, Porto, Portugal		

**Strain ID**	**Plectonema cf. radiosum LEGE 06105**		
**Strain Taxonomy**	Plectonema radiosum (Schiedermayer) Gomont (1892)		
**Microphotograph 400×**	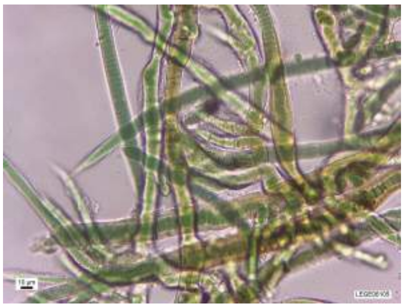	**Microphotograph 1000×**	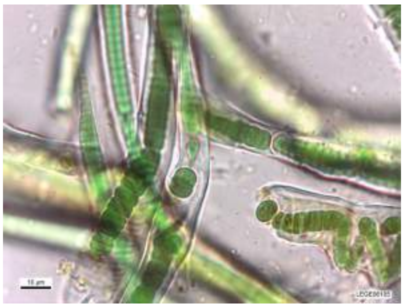
**Coidentity**	n.a.	**Morphometrics (Width × Length or Diameter) (µm)**	5,7±1,6 × 3,4±0,8 (Cells); Sheath up to 2,7±1,2
**Other Code(s)**	LEAN 009	**Taxonomic notes/diacritical features**	false-branching observed; with necridic cells; with sheath
**Parent Sample**	O.06 LUZ 3 - BC (II)	**Macroscopic growth features**	color: cyano; forming tuft-like aggregates
**Isolation Date**		**Environment**	aquatic, marine
**Order (Older Classification Scheme)**	Nostocales**	**Habitat Sample Description**	intertidal zone, on green macroalga
**Order 2014 (Modern Taxonomy)**	Nostocales**	**Ecophysiological traits**	
**Taxonomy Notes**	**see genus description in Komarek & Anagnostidis (2007)	**Notes on ecophysiological traits**	dehydroabietic acid production (unknown ecophysiological role)
**Accession Number(s)_16S**	KU951796	**Isolator**	Vitor Ramos
**Accession Number(s)_others (product/gene /region)**	KC813187 (non-ribosomal peptide synthetase), KC842355, KC842356 (polyketide synthase)	**Medium**	MN w/ vit. B12
**Collector**	Rui Seabra	**Strain Status**	non-axenic, unicyanobacterial
**Collection Date**	2006/12/12	**Preservation type**	subculturing, cryopreserved
**Location**	Portugal: Praia da Luz, Lagos	**Light:dark cycle**	12:12-h
**Latitude & Longitude**	37.08622 N 8.725375 W	**Temperature (ºC)**	19
		**Light intensity**	10–30 µmol photons m−2 s−1
**References Publicly Available Articles**	Brito et al. (2012) Syst. Appl. Microbiol. 35, 110–119; Rastoll et al. (2013) J. Appl. Phycol. 25, 1483-1493; Brito et al. (2015) Algal Res. 9, 218-226; Costa et al. (2016) Sci. Rep. 6, 23436		
**References Publicly Available Theses**			

Strain ID	**Plectonema cf. radiosum LEGE 06114**		
**Strain Taxonomy**	Plectonema radiosum (Schiedermayer) Gomont (1892)		
**Microphotograph 400×**	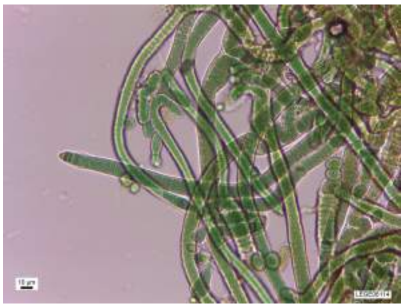	**Microphotograph 1000×**	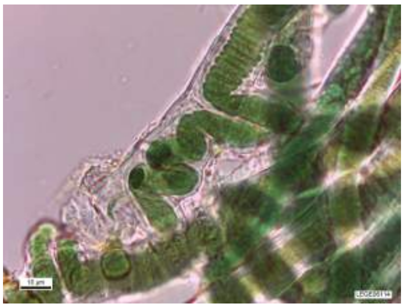
**Coidentity**	n.a.	**Morphometrics (Width × Length or Diameter) (µm)**	9,2±1,5 × 3,0±1,1 ; Sheath up to 2,8µm wide
**Other Code(s)**	LEAN 019	**Taxonomic notes/diacritical features**	with necridic cells and hormogonia; false-branching observed
**Parent Sample**	O.06 LUZ 3 - BCA	**Macroscopic growth features**	color: cyano; forming aggregates
**Isolation Date**		**Environment**	aquatic, marine
**Order (Older Classification Scheme)**	Nostocales**	**Habitat Sample Description**	intertidal zone, on a green macroalga
**Order 2014 (Modern Taxonomy)**	Nostocales**	**Ecophysiological traits**	
**Taxonomy Notes**	**see genus description in Komarek & Anagnostidis (2007)	**Notes on ecophysiological traits**	
**Accession Number(s)_16S**		**Isolator**	Vitor Ramos
**Accession Number(s)_others (product/gene /region)**		**Medium**	Z8 25‰ TM sea salt w/ vit. B12
**Collector**	Rui Seabra	**Strain Status**	non-axenic, unicyanobacterial
**Collection Date**	2006/12/12	**Preservation type**	subculturing
**Location**	Portugal: Praia da Luz, Lagos	**Light:dark cycle**	12:12-h
**Latitude & Longitude**	37.08622 N 8.725375 W	**Temperature (ºC)**	19
		**Light intensity**	10–30 µmol photons m−2 s−1
**References Publicly Available Articles**	Rastoll et al. (2013) J. Appl. Phycol. 25, 1483-1493		
**References Publicly Available Theses**			

Strain ID **Pseudanabaena aff. mucicola LEGE 00260**

**Strain Taxonomy**	Pseudanabaena mucicola (Naumann & Huber-Pestalozzi) Schwabe (1964)		
**Microphotograph 400×**	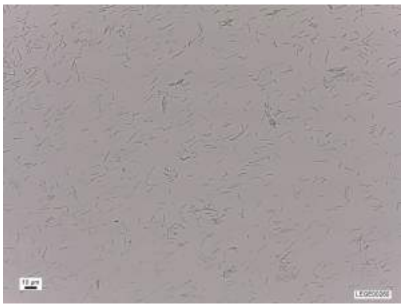	**Microphotograph 1000×**	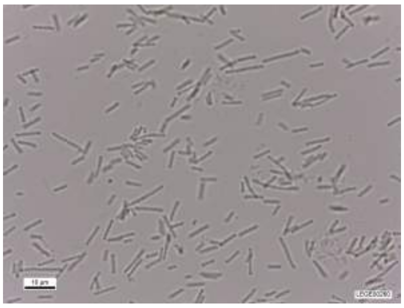
**Coidentity**	n.a.	**Morphometrics (Width × Length or Diameter) (µm)**	1,0±0,2 × 1,5±0,4
**Other Code(s)**	J48, LEANJ.48	**Taxonomic notes/diacritical features**	short filaments, usually with less than four cells
**Parent Sample**		**Macroscopic growth features**	color: cyano; growing homogeneously; mucilaginous
**Isolation Date**		**Environment**	aquatic, freshwater
**Order (Older Classification Scheme)**	Oscillatoriales	**Habitat Sample Description**	water sample
**Order 2014 (Modern Taxonomy)**	Synechococcales	**Ecophysiological traits**	EPS production
**Taxonomy Notes**		**Notes on ecophysiological traits**	non-ANTX-a producing strain
**Accession Number(s)_16S**	KU951797	**Isolator**	Joana Osswald
**Accession Number(s)_others (product/gene /region)**		**Medium**	Z8
**Collector**	Joana Oswald	**Strain Status**	non-axenic, unicyanobacterial
**Collection Date**	2000/09/12	**Preservation type**	subculturing
**Location**	Portugal: Chaves	**Light:dark cycle**	12:12-h
**Latitude & Longitude**	unknown	**Temperature (ºC)**	19
		**Light intensity**	10–30 µmol photons m−2 s−1
**References Publicly Available Articles**			
**References Publicly Available Theses**			

Strain ID	**Pseudanabaena cf. curta LEGE 07169**		
**Strain Taxonomy**	Pseudanabaena curta (Hollerbach) Cronberg & Komárek (1994)		
**Microphotograph 400×**	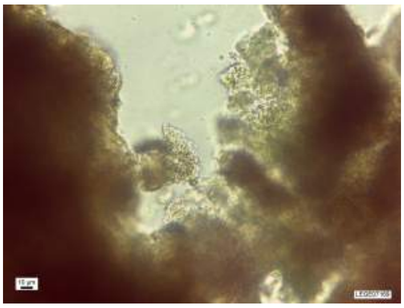	**Microphotograph 1000×**	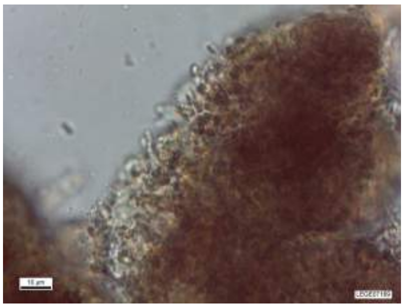
**Coidentity**	n.a.	**Morphometrics (Width × Length or Diameter) (µm)**	1,7±0,4 × 1,4 ±0,3
**Other Code(s)**	LEAN 083	**Taxonomic notes/diacritical features**	mucilaginous clusters of short filaments, constricted at cross-walls
**Parent Sample**	P.07 AGU 4 - BDA	**Macroscopic growth features**	color: dark green-brownish; forming aggregates/colonies
**Isolation Date**		**Environment**	aquatic, marine
**Order (Older Classification Scheme)**	Oscillatoriales	**Habitat Sample Description**	intertidal zone, wave-exposed rock
**Order 2014 (Modern Taxonomy)**	not determined	**Ecophysiological traits**	EPS production
**Taxonomy Notes**		**Notes on ecophysiological traits**	capsules
**Accession Number(s)_16S**	HQ832923	**Isolator**	Vitor Ramos
**Accession Number(s)_others (product/gene /region)**	KC842326, KC842327 (polyketide synthase)	**Medium**	MN w/ vit. B12
**Collector**	Rui Seabra/Vitor Ramos	**Strain Status**	non-axenic, unicyanobacterial
**Collection Date**	2007/05/15	**Preservation type**	subculturing
**Location**	Portugal: Praia da Aguda, Arcozelo	**Light:dark cycle**	12:12-h
**Latitude & Longitude**	41.04954 N 8.655339 W	**Temperature (ºC)**	19
		**Light intensity**	10–30 µmol photons m−2 s−1
**References Publicly Available Articles**	Brito et al. (2012) Syst. Appl. Microbiol. 35, 110–119; Leão et al. (2013) Mar. Drugs 11 (4), 1316-1335; Costa et al. (2014) Mar. Drugs 12, 98-114; Brito et al. (2015) Algal Res. 9, 218-226		
**References Publicly Available Theses**			

Strain ID	**Pseudanabaena cf. curta LEGE 10371**		
**Strain Taxonomy**	Pseudanabaena curta (Hollerbach) Cronberg & Komárek (1994)		
**Microphotograph 400×**	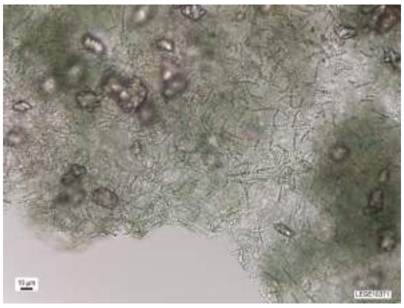	**Microphotograph 1000×**	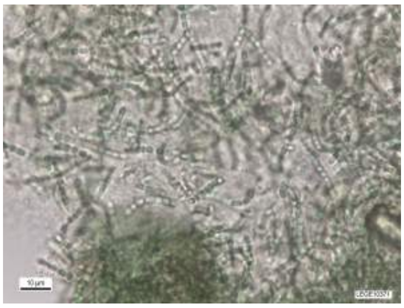
**Coidentity**	n.a.	**Morphometrics (Width × Length or Diameter) (µm)**	1,4±0,2 × 2,2±0,5
**Other Code(s)**	13F	**Taxonomic notes/diacritical features**	short trichomes, constricted at cross-walls
**Parent Sample**		**Macroscopic growth features**	color: cyano; forming a firm biofilm
**Isolation Date**		**Environment**	aquatic, marine
**Order (Older Classification Scheme)**	Oscillatoriales	**Habitat Sample Description**	intertidal zone, on a marine sponge
**Order 2014 (Modern Taxonomy)**	Synechococcales	**Ecophysiological traits**	
**Taxonomy Notes**		**Notes on ecophysiological traits**	
**Accession Number(s)_16S**	KU951798	**Isolator**	Ana Regueiras
**Accession Number(s)_others (product/gene /region)**		**Medium**	MN w/ vit. B12
**Collector**	Ana Regueiras	**Strain Status**	non-axenic, unicyanobacterial
**Collection Date**	2010/10/12	**Preservation type**	subculturing
**Location**	Portugal: Praia da Memória	**Light:dark cycle**	12:12-h
**Latitude & Longitude**	41.23119 N 8.721750 W	**Temperature (ºC)**	19
		**Light intensity**	10–30 µmol photons m−2 s−1
**References Publicly Available Articles**			
**References Publicly Available Theses**	Vivas J. (2013) MSc Dissertation, FCUP, University of Porto, Porto, Portugal		

**Strain ID**	**Rivularia sp. LEGE 07159**		
**Strain Taxonomy**	Rivularia Agardh, C ex Bornet, É & Flahault, C (1886)		
**Microphotograph 400×**	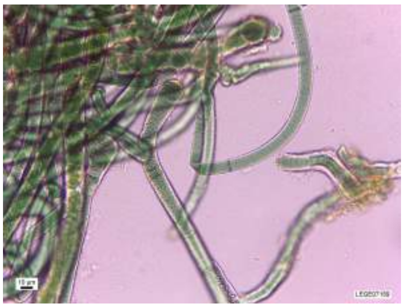	**Microphotograph 1000×**	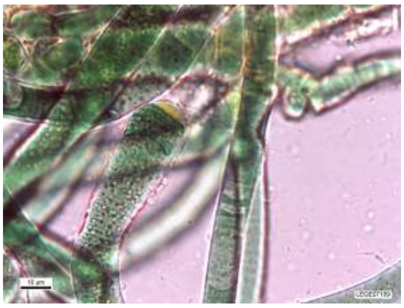
**Coidentity**	n.a.	**Morphometrics (Width × Length or Diameter) (µm)**	10,7±2,8 × 4,5±1,9 (Cells); Ø 8,6±2,3 (Hemispheric Heterocytes); Sheath up to 2,2±0,9
**Other Code(s)**	LEAN 073	**Taxonomic notes/diacritical features**	heteropolar trichomes; false-branching observed; with necridic cells and hormogonia; cell ultrastructure data available; with sheath
**Parent Sample**	P.07 BUR 6 - p96, dil. 1/4	**Macroscopic growth features**	color: cyano; forming tuft-like aggregates
**Isolation Date**		**Environment**	aquatic, marine
**Order (Older Classification Scheme)**	Nostocales	**Habitat Sample Description**	tide pool, rock surface scraping
**Order 2014 (Modern Taxonomy)**	Nostocales	**Ecophysiological traits**	nitrogen-fixation
**Taxonomy Notes**		**Notes on ecophysiological traits**	
**Accession Number(s)_16S**	KC989702	**Isolator**	Vitor Ramos
**Accession Number(s)_others (product/gene /region)**	KC256768 (dinitrogenase reductase, nifH), KC813185, KC813186 (non-ribosomal peptide synthetase), KC842351-KC842353 (polyketide synthase), KF008259 (cyanobactin, N-terminal protease (A))	**Medium**	MN w/ vit. B12
**Collector**	Rui Seabra/Vitor Ramos	**Strain Status**	non-axenic, unicyanobacterial
**Collection Date**	2007/05/02	**Preservation type**	subculturing
**Location**	Portugal: Praia de Burgau, Budens	**Light:dark cycle**	12:12-h
**Latitude & Longitude**	37.07156 N 8.774722 W	**Temperature (ºC)**	19
		**Light intensity**	10–30 µmol photons m−2 s−1
**References Publicly Available Articles**	Brito et al. (2012) Syst. Appl. Microbiol. 35, 110–119; Martins et al. (2013) Mar. Drugs 11 (12), 4902-4916; Rastoll et al. (2013) J. Appl. Phycol. 25, 1483-1493; Brito et al. (2015) Algal Res. 9, 218-226		
**References Publicly Available Theses**			

**Strain ID**	**Rivularia sp. LEGE 07177**		
**Strain Taxonomy**	Rivularia Agardh, C ex Bornet, É & Flahault, C (1886)		
**Microphotograph 400×**	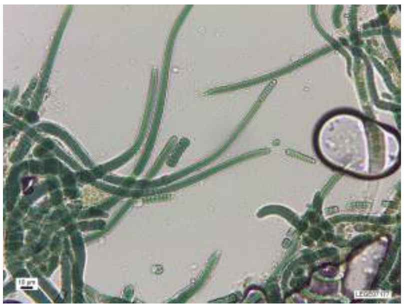	**Microphotograph 1000×**	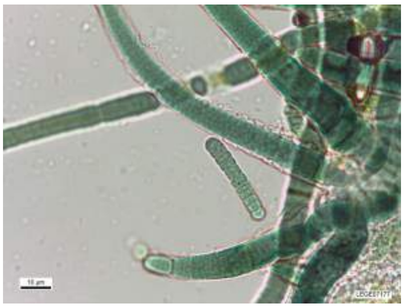
**Coidentity**	n.a.	**Morphometrics (Width × Length or Diameter) (µm)**	5,1±1,2 × 1,9±0,8 (Cells); Sheath up to 1,1±0,3
**Other Code(s)**	LEAN 091	**Taxonomic notes/diacritical features**	heteropolar trichomes; with necridic cells and hormogonia; with sheath
**Parent Sample**	P.07 MAR 5 - BCB	**Macroscopic growth features**	color: cyano; forming tuft-like aggregates
**Isolation Date**	2007	**Environment**	aquatic, marine
**Order (Older Classification Scheme)**	Nostocales	**Habitat Sample Description**	intertidal zone, on green macroalga
**Order 2014 (Modern Taxonomy)**	Nostocales	**Ecophysiological traits**	nitrogen-fixation
**Taxonomy Notes**		**Notes on ecophysiological traits**	
**Accession Number(s)_16S**	KU951799	**Isolator**	Vitor Ramos
**Accession Number(s)_others (product/gene /region)**	KC813184 (non-ribosomal peptide synthetase), KC842345, KC842346 (polyketide synthase)	**Medium**	MN w/ vit. B12
**Collector**	Rui Seabra/Vitor Ramos	**Strain Status**	non-axenic, unicyanobacterial
**Collection Date**	2007/05/02	**Preservation type**	subculturing
**Location**	Portugal: Praia do Martinhal, Vila do Bispo	**Light:dark cycle**	12:12-h
**Latitude & Longitude**	37.01869 N 8.926714 W	**Temperature (ºC)**	19
		**Light intensity**	10–30 µmol photons m−2 s−1
**References Publicly Available Articles**	Brito et al. (2012) Syst. Appl. Microbiol. 35, 110–119; Leão et al. (2013) Mar. Drugs 11 (4), 1316-1335; Brito et al. (2015) Algal Res. 9, 218-226		
**References Publicly Available Theses**			

**Strain ID**	**Rivularia sp. LEGE 07178**		
**Strain Taxonomy**	Rivularia Agardh, C ex Bornet, É & Flahault, C (1886)		
**Microphotograph 400×**	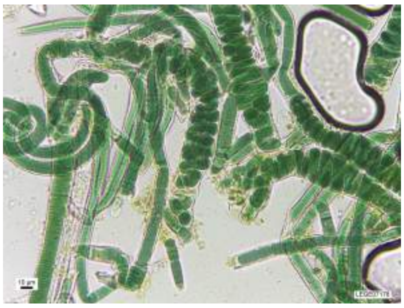	**Microphotograph 1000×**	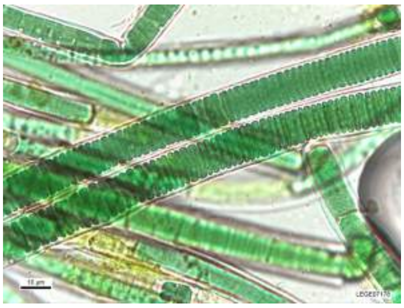
**Coidentity**	n.a.	**Morphometrics (Width × Length or Diameter) (µm)**	9,1±2,3 × 2,8±0,6 (Cells); Sheath up to 1,9±0,6
**Other Code(s)**	LEAN 092	**Taxonomic notes/diacritical features**	heteropolar trichomes; false-branching observed; with necridic cells and hormogonia
**Parent Sample**	P.07 BUR 6 - p96, dil. 1/4 - AB	**Macroscopic growth features**	color: cyano; forming tuft-like aggregates
**Isolation Date**		**Environment**	aquatic, marine
**Order (Older Classification Scheme)**	Nostocales	**Habitat Sample Description**	intertidal zone, rock surface scraping
**Order 2014 (Modern Taxonomy)**	Nostocales	**Ecophysiological traits**	nitrogen-fixation
**Taxonomy Notes**		**Notes on ecophysiological traits**	
**Accession Number(s)_16S**		**Isolator**	Vitor Ramos
**Accession Number(s)_others (product/gene /region)**		**Medium**	MN w/ vit. B12
**Collector**	Rui Seabra/Vitor Ramos	**Strain Status**	non-axenic, unicyanobacterial
**Collection Date**	2007/05/02	**Preservation type**	subculturing
**Location**	Portugal: Praia de Burgau, Budens	**Light:dark cycle**	12:12-h
**Latitude & Longitude**	37.07156 N 8.774722 W	**Temperature (ºC)**	19
		**Light intensity**	10–30 µmol photons m−2 s−1
**References Publicly Available Articles**			
**References Publicly Available Theses**	Macieira R. (2013) MSc Dissertation, FCUP, University of Porto, Porto, Portugal		

Strain ID	**Romeria aff. gracilis LEGE 07310**		
**Strain Taxonomy**	Romeria gracilis (Koczwara) Koczwara ex Geitler, L (1932)		
**Microphotograph 400×**	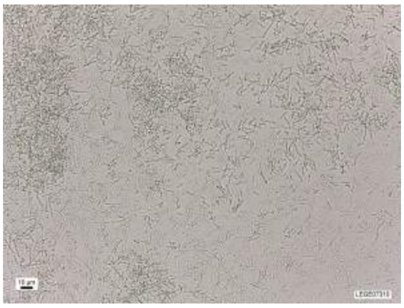	**Microphotograph 1000×**	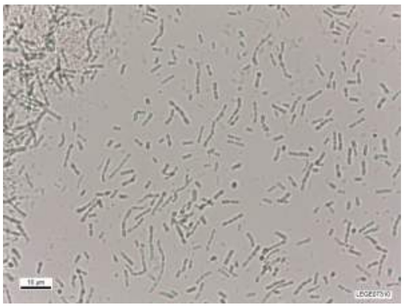
**Coidentity**	n.a.	**Morphometrics (Width × Length or Diameter) (µm)**	1,0±0,1 × 2,0±0,5
**Other Code(s)**		**Taxonomic notes/diacritical features**	irregular shaped cells; some elongated, involution cells observed
**Parent Sample**	240	**Macroscopic growth features**	color: cyano; growing homogeneously, but also forming aggregates
**Isolation Date**		**Environment**	aquatic, brackish
**Order (Older Classification Scheme)**	Oscillatoriales	**Habitat Sample Description**	mesotidal zone, benthic
**Order 2014 (Modern Taxonomy)**	Synechococcales	**Ecophysiological traits**	
**Taxonomy Notes**		**Notes on ecophysiological traits**	
**Accession Number(s)_16S**	HM217057	**Isolator**	Viviana Lopes
**Accession Number(s)_others (product/gene /region)**		**Medium**	Z8
**Collector**	Viviana Lopes	**Strain Status**	non-axenic, unicyanobacterial
**Collection Date**	2007	**Preservation type**	subculturing
**Location**	Portugal: Minho estuary, Caminha	**Light:dark cycle**	12:12-h
**Latitude & Longitude**	41.86697 N 8.855833 W	**Temperature (ºC)**	19
		**Light intensity**	10–30 µmol photons m−2 s−1
**References Publicly Available Articles**	Lopes et al. (2012) Mar. Environ. Res. 73, 7–16; Costa et al. (2014) Mar. Drugs 12, 98-114		
**References Publicly Available Theses**	Lopes V.R. (2011) PhD Thesis, FCUP, University of Porto, Porto, Portugal; Macieira R. (2013) MSc Dissertation, FCUP, University of Porto, Porto, Portugal		

**Strain ID**	**Schizothrix aff. septentrionalis LEGE 07164**
**Strain Taxonomy**	Schizothrix septentrionalis Gomont (1899)

**Microphotograph 400×**	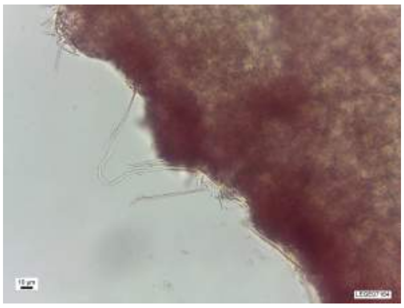	**Microphotograph 1000×**	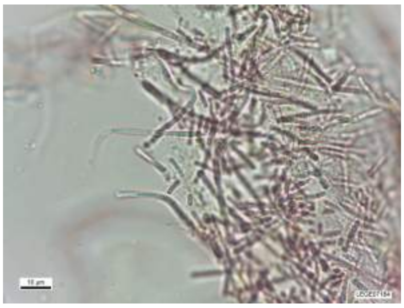
**Coidentity**	n.a.	**Morphometrics (Width × Length or Diameter) (µm)**	1,8±0,3 × 1,3±0,2
**Other Code(s)**	LEAN 078	**Taxonomic notes/diacritical features**	
**Parent Sample**	P.07 MOL 8 - p96, dil. 1/2	**Macroscopic growth features**	color: brown-reddish; forming a firm biofilm and aggregates
**Isolation Date**		**Environment**	aquatic, marine
**Order (Older Classification Scheme)**	Oscillatoriales	**Habitat Sample Description**	intertidal zone, on a brown macroalga
**Order 2014 (Modern Taxonomy)**	Synechococcales	**Ecophysiological traits**	
**Taxonomy Notes**		**Notes on ecophysiological traits**	
**Accession Number(s)_16S**	KU951800	**Isolator**	Vitor Ramos
**Accession Number(s)_others (product/gene /region)**	KC813173 (non-ribosomal peptide synthetase), KC842290 (polyketide synthase)	**Medium**	MN w/ vit. B12
**Collector**	Rui Seabra/Vitor Ramos	**Strain Status**	non-axenic, unicyanobacterial
**Collection Date**	2007/05/21	**Preservation type**	subculturing
**Location**	Portugal: Praia de Moledo, Caminha	**Light:dark cycle**	12:12-h
**Latitude & Longitude**	41.84963 N 8.866717 W	**Temperature (ºC)**	19
		**Light intensity**	10–30 µmol photons m−2 s−1
**References Publicly Available Articles**	Brito et al. (2012) Syst. Appl. Microbiol. 35, 110–119; Leão et al. (2013) Mar. Drugs 11 (4), 1316-1335; Brito et al. (2015) Algal Res. 9, 218-226		
**References Publicly Available Theses**			

**Strain ID**	**Scytonema sp. LEGE 07182**		
**Strain Taxonomy**	Scytonema Agardh, C ex Bornet, É & Flahault, C (1886)		
**Microphotograph 400×**	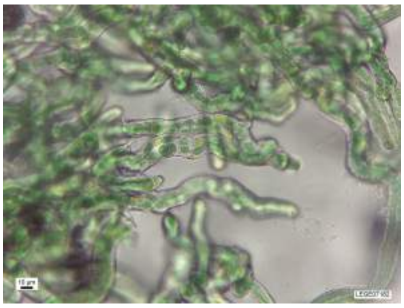	**Microphotograph 1000×**	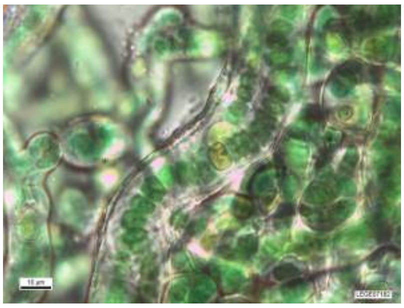
**Coidentity**	n.a.	**Morphometrics (Width × Length or Diameter) (µm)**	7,4±2,1 × 7,7±1,3 (Cells); 7,4±2,3 × 7,1±1,3 (Heterocytes); Sheath up to 2,8±0,7
**Other Code(s)**	LEAN 096	**Taxonomic notes/diacritical features**	terminal and/or rarely intercalar heterocytes; with sheath
**Parent Sample**	P.07 MOL 5- FCAC/E/D	**Macroscopic growth features**	color: cyano; forming tuft-like aggregates
**Isolation Date**		**Environment**	aquatic, marine
**Order (Older Classification Scheme)**	Nostocales	**Habitat Sample Description**	intertidal zone, rock surface scraping
**Order 2014 (Modern Taxonomy)**	Nostocales	**Ecophysiological traits**	nitrogen-fixation
**Taxonomy Notes**		**Notes on ecophysiological traits**	
**Accession Number(s)_16S**		**Isolator**	Vitor Ramos
**Accession Number(s)_others (product/gene /region)**		**Medium**	MN w/ vit. B12
**Collector**	Rui Seabra/Vitor Ramos	**Strain Status**	non-axenic, unicyanobacterial
**Collection Date**	2007/05/21	**Preservation type**	subculturing
**Location**	Portugal: Praia de Moledo, Caminha	**Light:dark cycle**	12:12-h
**Latitude & Longitude**	41.84963 N 8.866717 W	**Temperature (ºC)**	19
		**Light intensity**	10–30 µmol photons m−2 s−1
**References Publicly Available Articles**			
**References Publicly Available Theses**	Macieira R. (2013) MSc Dissertation, FCUP, University of Porto, Porto, Portugal		

Strain ID	**Scytonema sp. LEGE 07189**		
**Strain Taxonomy**	Scytonema Agardh, C ex Bornet, É & Flahault, C (1886)		
**Microphotograph 400×**	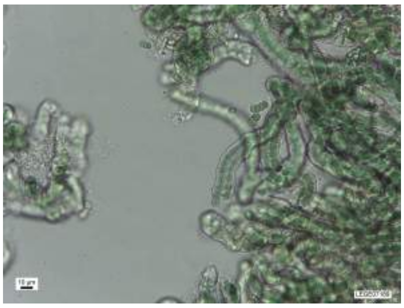	**Microphotograph 1000×**	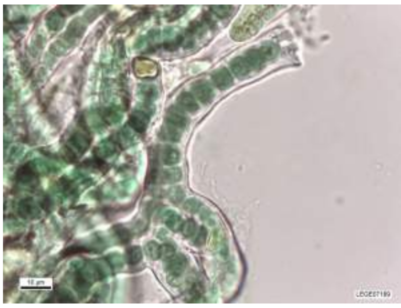
**Coidentity**	n.a.	**Morphometrics (Width × Length or Diameter) (µm)**	7,0±1,3 × 6,1±2,0 (cells); 7,7±2,0 × 8,0±1,6 (Heterocytes); Sheath up to 2,1±0,5
**Other Code(s)**	LEAN 154	**Taxonomic notes/diacritical features**	intercalar heterocytes; often, filaments consist of two trichomes enveloped in a common sheath; hormogonia; with sheath
**Parent Sample**	P.07 MOL 5 - FCBA	**Macroscopic growth features**	color: cyano; forming tuft-like aggregates
**Isolation Date**		**Environment**	aquatic, marine
**Order (Older Classification Scheme)**	Nostocales	**Habitat Sample Description**	intertidal zone, rock surface scraping
**Order 2014 (Modern Taxonomy)**	Nostocales	**Ecophysiological traits**	nitrogen-fixation
**Taxonomy Notes**		**Notes on ecophysiological traits**	
**Accession Number(s)_16S**	HQ832905	**Isolator**	Vitor Ramos
**Accession Number(s)_others (product/gene /region)**	KC842295-KC842297 (polyketide synthase)	**Medium**	MN w/ vit. B12
**Collector**	Rui Seabra/Vitor Ramos	**Strain Status**	non-axenic, unicyanobacterial
**Collection Date**	2007/05/21	**Preservation type**	subculturing
**Location**	Portugal: Praia de Moledo, Caminha	**Light:dark cycle**	12:12-h
**Latitude & Longitude**	41.84963 N 8.866717 W	**Temperature (ºC)**	19
		**Light intensity**	10–30 µmol photons m−2 s−1
**References Publicly Available Articles**	Brito et al. (2012) Syst. Appl. Microbiol. 35, 110–119; Brito et al. (2015) Algal Res. 9, 218-226		
**References Publicly Available Theses**	Macieira R. (2013) MSc Dissertation, FCUP, University of Porto, Porto, Portugal		

**Strain ID**	**Sphaerospermopsis sp. LEGE 00249**		
**Strain Taxonomy**	Sphaerospermopsis Zapomelová, E; Jezberová, J; Hrouzek, P; Hisem, D; Reháková, K & Komárková, J (2010)		
**Microphotograph 400×**	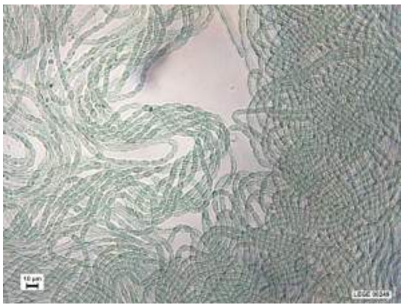	**Microphotograph 1000×**	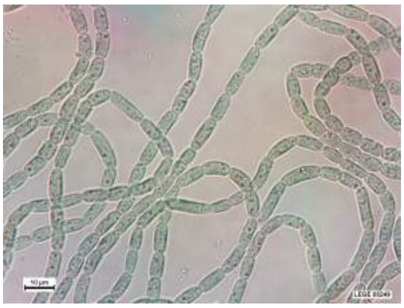
**Coidentity**	n.a.	**Morphometrics (Width × Length or Diameter) (µm)**	3,5±0,4 × 7,9±1,1
**Other Code(s)**		**Taxonomic notes/diacritical features**	
**Parent Sample**		**Macroscopic growth features**	color: cyano; growing homogeneously
**Isolation Date**		**Environment**	aquatic, freshwater
**Order (Older Classification Scheme)**	Nostocales	**Habitat Sample Description**	water sample
**Order 2014 (Modern Taxonomy)**	Nostocales	**Ecophysiological traits**	
**Taxonomy Notes**		**Notes on ecophysiological traits**	
**Accession Number(s)_16S**	KC989701	**Isolator**	Joana Osswald
**Accession Number(s)_others (product/gene /region)**	KF008258 (cyanobactin, N-terminal protease (A))	**Medium**	Z8
**Collector**	Joana Oswald	**Strain Status**	non-axenic, unicyanobacterial
**Collection Date**	2000/07/07	**Preservation type**	subculturing
**Location**	Portugal: Maranhão dam reservoir, campsite at Montargil	**Light:dark cycle**	12:12-h
**Latitude & Longitude**	unknown	**Temperature (ºC)**	19
		**Light intensity**	10–30 µmol photons m−2 s−1
**References Publicly Available Articles**	Martins et al. (2013) Mar. Drugs 11 (12), 4902-4916		
**References Publicly Available Theses**			

Strain ID	**Sphaerospermopsis sp. LEGE 02266**		
**Strain Taxonomy**	Sphaerospermopsis Zapomelová, E; Jezberová, J; Hrouzek, P; Hisem, D; Reháková, K & Komárková, J (2010)		
**Microphotograph 400×**	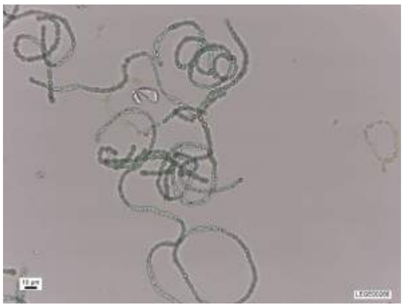	**Microphotograph 1000×**	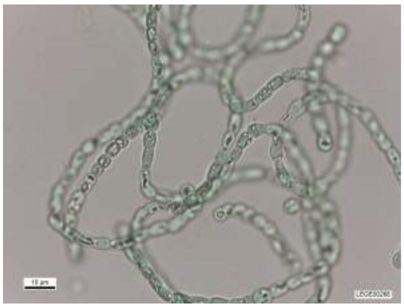
**Coidentity**	n.a.	**Morphometrics (Width × Length or Diameter) (µm)**	3,3±0,9 × 4,9±1,0
**Other Code(s)**	J59, LEANJ.59	**Taxonomic notes/diacritical features**	highly curved or coiled trichomes; vacuolated cells
**Parent Sample**		**Macroscopic growth features**	color: cyano; growing homogeneously, but also forming aggregates
**Isolation Date**		**Environment**	aquatic, freshwater
**Order (Older Classification Scheme)**	Nostocales	**Habitat Sample Description**	water sample
**Order 2014 (Modern Taxonomy)**	Nostocales	**Ecophysiological traits**	
**Taxonomy Notes**		**Notes on ecophysiological traits**	
**Accession Number(s)_16S**	KU951801	**Isolator**	Joana Osswald
**Accession Number(s)_others (product/gene /region)**		**Medium**	Z8
**Collector**	Joana Oswald	**Strain Status**	non-axenic, unicyanobacterial
**Collection Date**	2002/07/25	**Preservation type**	subculturing
**Location**	Portugal: Maranhão dam reservoir, Benavila, Avis	**Light:dark cycle**	12:12-h
**Latitude & Longitude**	unknown	**Temperature (ºC)**	19
		**Light intensity**	10–30 µmol photons m−2 s−1
**References Publicly Available Articles**			
**References Publicly Available Theses**			

**Strain ID**	**Sphaerospermopsis sp. LEGE 08334**		
**Strain Taxonomy**	Sphaerospermopsis Zapomelová, E; Jezberová, J; Hrouzek, P; Hisem, D; Reháková, K & Komárková, J (2010)		
**Microphotograph 400×**	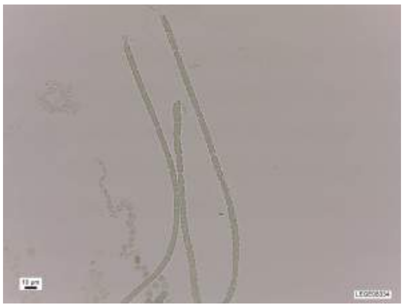	**Microphotograph 1000×**	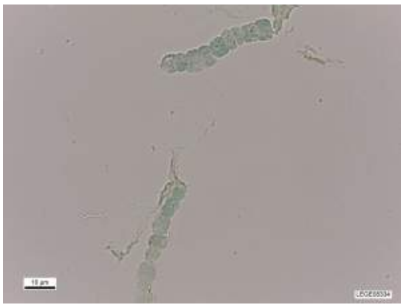
**Coidentity**	n.a.	**Morphometrics (Width × Length or Diameter) (µm)**	4,6±0,5 × 4,0±0,6
**Other Code(s)**		**Taxonomic notes/diacritical features**	
**Parent Sample**		**Macroscopic growth features**	color: cyano; forming a smooth biofilm
**Isolation Date**		**Environment**	aquatic, freshwater
**Order (Older Classification Scheme)**	Nostocales	**Habitat Sample Description**	water sample
**Order 2014 (Modern Taxonomy)**	Nostocales	**Ecophysiological traits**	nitrogen-fixation
**Taxonomy Notes**		**Notes on ecophysiological traits**	
**Accession Number(s)_16S**	KU951802	**Isolator**	Vitor Vasconcelos
**Accession Number(s)_others (product/gene /region)**		**Medium**	Z8
**Collector**	Vitor Vasconcelos	**Strain Status**	non-axenic, unicyanobacterial
**Collection Date**	2008	**Preservation type**	subculturing
**Location**	Mexico: Lake Zumpango	**Light:dark cycle**	12:12-h
**Latitude & Longitude**	unknown	**Temperature (ºC)**	19
		**Light intensity**	10–30 µmol photons m−2 s−1
**References Publicly Available Articles**			
**References Publicly Available Theses**	Macieira R. (2013) MSc Dissertation, FCUP, University of Porto, Porto, Portugal		

**Strain ID**	**Sphaerospermopsis sp. LEGE 08335**		
**Strain Taxonomy**	Sphaerospermopsis Zapomelová, E; Jezberová, J; Hrouzek, P; Hisem, D; Reháková, K & Komárková, J (2010)		
**Microphotograph 400×**	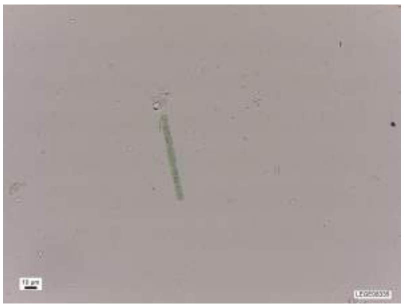	**Microphotograph 1000×**	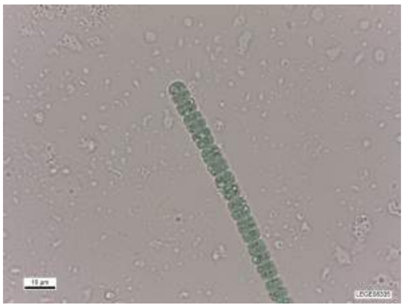
**Coidentity**	n.a.	**Morphometrics (Width × Length or Diameter) (µm)**	5,3±0,3 × 3,1±0,7
**Other Code(s)**		**Taxonomic notes/diacritical features**	
**Parent Sample**		**Macroscopic growth features**	color: cyano; forming a smooth biofilm
**Isolation Date**		**Environment**	aquatic, freshwater
**Order (Older Classification Scheme)**	Nostocales	**Habitat Sample Description**	water sample
**Order 2014 (Modern Taxonomy)**	Nostocales	**Ecophysiological traits**	nitrogen-fixation
**Taxonomy Notes**		**Notes on ecophysiological traits**	
**Accession Number(s)_16S**	KU951803	**Isolator**	Vitor Vasconcelos
**Accession Number(s)_others (product/gene /region)**		**Medium**	Z8
**Collector**	Vitor Vasconcelos	**Strain Status**	non-axenic, unicyanobacterial, delicate
**Collection Date**	2008	**Preservation type**	subculturing
**Location**	Mexico: Lake Zumpango	**Light:dark cycle**	12:12-h
**Latitude & Longitude**	unknown	**Temperature (ºC)**	19
		**Light intensity**	10–30 µmol photons m−2 s−1
**References Publicly Available Articles**			
**References Publicly Available Theses**			

**Strain ID**	**Spirulina sp. LEGE 11439**		
**Strain Taxonomy**	Spirulina Turpin ex Gomont (1892)		
**Microphotograph 400×**	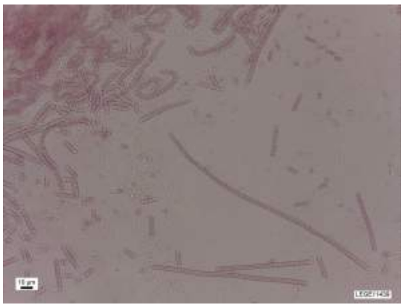	**Microphotograph 1000×**	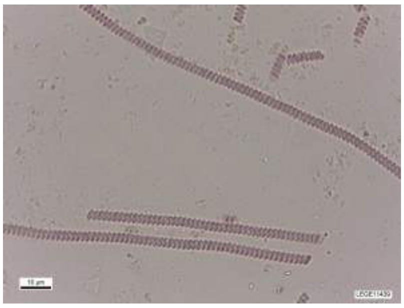
**Coidentity**	n.a.	**Morphometrics (Width × Length or Diameter) (µm)**	Cells 0,8±0,1 wide; 1,6±0,2 (distance between colis); Coils 2,6±0,1 wide
**Other Code(s)**		**Taxonomic notes/diacritical features**	helical tightly-coiled trichomes
**Parent Sample**	1.003.10.11	**Macroscopic growth features**	color: reddish; forming a firm biofilm
**Isolation Date**		**Environment**	aquatic, marine
**Order (Older Classification Scheme)**	Oscillatoriales	**Habitat Sample Description**	subtidal sample, epilithic (13m depth), about 200 m off the shore
**Order 2014 (Modern Taxonomy)**	Oscillatoriales	**Ecophysiological traits**	
**Taxonomy Notes**		**Notes on ecophysiological traits**	
**Accession Number(s)_16S**	KU951804	**Isolator**	Ângela Pinheiro
**Accession Number(s)_others (product/gene /region)**		**Medium**	Z8 25‰ TM sea salt w/ vit. B12
**Collector**	Pedro Leão/Aldo Barreiro	**Strain Status**	non-axenic, unicyanobacterial
**Collection Date**	2011/10/03	**Preservation type**	subculturing
**Location**	Portugal: 'A Pedra', diving spot in front of the fort 'Castelo do Queijo'	**Light:dark cycle**	12:12-h
**Latitude & Longitude**	41.185809 N 8.719079 W	**Temperature (ºC)**	19
		**Light intensity**	5 µmol photons m−2 s−1
**References Publicly Available Articles**			
**References Publicly Available Theses**			

Strain ID	**Synechococcus cf. nidulans LEGE 06322**		
**Strain Taxonomy**	Synechococcus nidulans (Pringsheim) Komárek in Bourrelly (1970)		
**Microphotograph 400×**	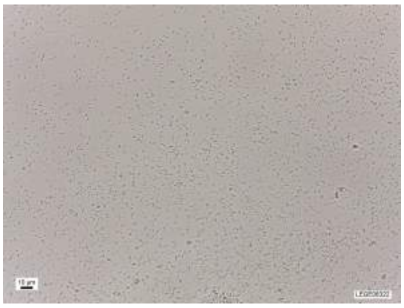	**Microphotograph 1000×**	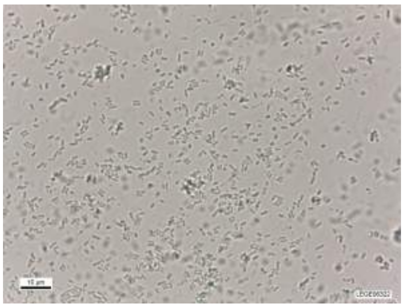
**Coidentity**	n.a.	**Morphometrics (Width × Length or Diameter) (µm)**	0,7±0,1 × 0,8±0,1 (rod-shaped cells); 0,8±0,1 × 1,7±0,2 (elongated cells); 0,8±0,1 × 3,5±1,0 (Involution cells)
**Other Code(s)**	Sup61206	**Taxonomic notes/diacritical features**	rod-shaped cells; some elongated, involution cells observed
**Parent Sample**	n.d.	**Macroscopic growth features**	color: cyano; growing homogeneously; mucilaginous
**Isolation Date**		**Environment**	aquatic, brackish
**Order (Older Classification Scheme)**	Chroococcales	**Habitat Sample Description**	mesotidal zone, benthic
**Order 2014 (Modern Taxonomy)**	Synechococcales	**Ecophysiological traits**	EPS production
**Taxonomy Notes**		**Notes on ecophysiological traits**	
**Accession Number(s)_16S**	HM217065	**Isolator**	Viviana Lopes
**Accession Number(s)_others (product/gene /region)**		**Medium**	Z8
**Collector**	Viviana Lopes	**Strain Status**	non-axenic, unicyanobacterial
**Collection Date**	2007/04/03	**Preservation type**	subculturing
**Location**	Portugal: Vouga estuary, Gafanha da Encarnação, Ílhavo	**Light:dark cycle**	12:12-h
**Latitude & Longitude**	40.62903 N 8.745167 W	**Temperature (ºC)**	19
		**Light intensity**	10–30 µmol photons m−2 s−1
**References Publicly Available Articles**	Lopes et al. (2012) Mar. Environ. Res. 73, 7–16		
**References Publicly Available Theses**	Lopes V.R. (2011) PhD Thesis, FCUP, University of Porto, Porto, Portugal		

Strain ID	**Synechococcus nidulans LEGE 06156**		
**Strain Taxonomy**	Synechococcus nidulans (Pringsheim) Komárek in Bourrelly (1970)		
**Microphotograph 400×**	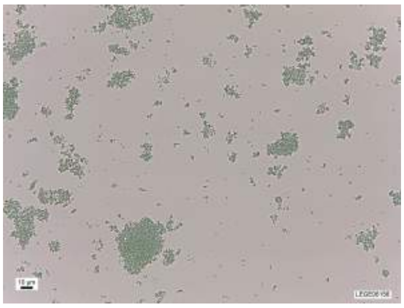	**Microphotograph 1000×**	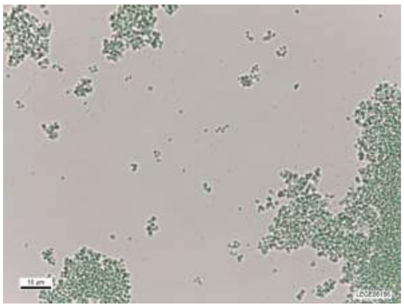
**Coidentity**	n.a.	**Morphometrics (Width × Length or Diameter) (µm)**	Ø 1,2±0,1
**Other Code(s)**	LEAN 070	**Taxonomic notes/diacritical features**	several cell clusters
**Parent Sample**	V.06 BUR p3 - A	**Macroscopic growth features**	color: cyano; growing homogeneously, but also forming a firm biofilm and aggregates
**Isolation Date**		**Environment**	aquatic, marine
**Order (Older Classification Scheme)**	Chroococcales	**Habitat Sample Description**	sea water sample, coastal, surf zone
**Order 2014 (Modern Taxonomy)**	Synechococcales	**Ecophysiological traits**	
**Taxonomy Notes**		**Notes on ecophysiological traits**	
**Accession Number(s)_16S**		**Isolator**	Vitor Ramos
**Accession Number(s)_others (product/gene /region)**		**Medium**	Z8 25‰ TM sea salt w/ vit. B12
**Collector**	Rui Seabra	**Strain Status**	non-axenic, unicyanobacterial
**Collection Date**	2006/07/17	**Preservation type**	subculturing
**Location**	Portugal: Praia de Burgau, Budens	**Light:dark cycle**	12:12-h
**Latitude & Longitude**	37.07156 N 8.774722 W	**Temperature (ºC)**	19
		**Light intensity**	10–30 µmol photons m−2 s−1
**References Publicly Available Articles**			
**References Publicly Available Theses**	Costa M.S. (2011) MSc Dissertation, ICBAS and FCUP, University of Porto, Porto, Portugal; Macieira R. (2013) MSc Dissertation, FCUP, University of Porto, Porto, Portugal		

**Strain ID**	**Synechococcus nidulans LEGE 07171**		
**Strain Taxonomy**	Synechococcus nidulans (Pringsheim) Komárek in Bourrelly (1970)		
**Microphotograph 400×**	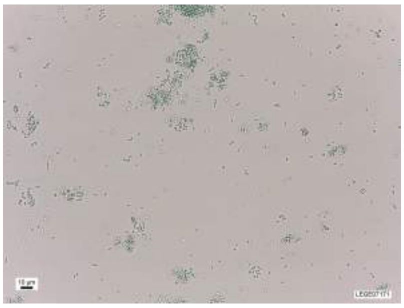	**Microphotograph 1000×**	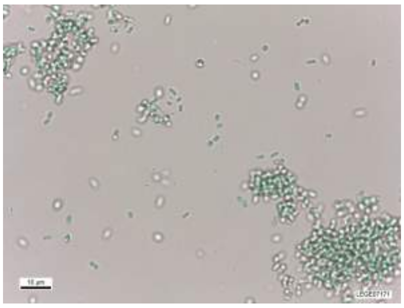
**Coidentity**	n.a.	**Morphometrics (Width × Length or Diameter) (µm)**	Ø 1,2±0,2
**Other Code(s)**	LEAN 085	**Taxonomic notes/diacritical features**	
**Parent Sample**	P.07 BUR 5 - AA	**Macroscopic growth features**	color: cyano; forming a firm biofilm; mucilaginous
**Isolation Date**		**Environment**	aquatic, marine
**Order (Older Classification Scheme)**	Chroococcales	**Habitat Sample Description**	tide puddle, air-exposed rock surface scraping
**Order 2014 (Modern Taxonomy)**	Synechococcales	**Ecophysiological traits**	
**Taxonomy Notes**		**Notes on ecophysiological traits**	
**Accession Number(s)_16S**	KU951805	**Isolator**	Vitor Ramos
**Accession Number(s)_others (product/gene /region)**		**Medium**	Z8 25‰ TM sea salt w/ vit. B12
**Collector**	Rui Seabra/Vitor Ramos	**Strain Status**	non-axenic, unicyanobacterial
**Collection Date**	2007/05/02	**Preservation type**	subculturing, cryopreserved
**Location**	Portugal: Praia de Burgau, Budens	**Light:dark cycle**	12:12-h
**Latitude & Longitude**	37.07156 N 8.774722 W	**Temperature (ºC)**	19
		**Light intensity**	10–30 µmol photons m−2 s−1
**References Publicly Available Articles**	Brito et al. (2012) Syst. Appl. Microbiol. 35, 110–119; Costa et al. (2014) Mar. Drugs 12, 98-114; Dvořák et al. (2014) Mol. Ecol. 23, 5538–5551		
**References Publicly Available Theses**			

**Strain ID**	**Synechococcus sp. LEGE 06306**		
**Strain Taxonomy**	Synechococcus Nägeli, C (1849)		
**Microphotograph 400×**	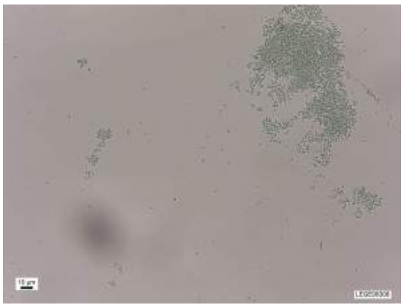	**Microphotograph 1000×**	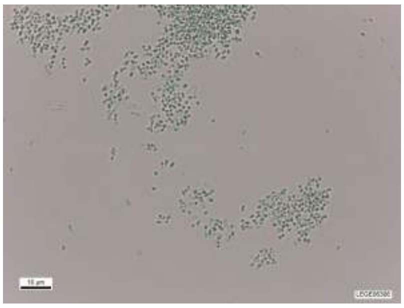
**Coidentity**	n.a.	**Morphometrics (Width × Length or Diameter) (µm)**	Ø 1,0±0,2 (Cells); Involution cells up to 4,1±2,1 length
**Other Code(s)**		**Taxonomic notes/diacritical features**	
**Parent Sample**	IN67	**Macroscopic growth features**	color: cyano; growing homogeneously
**Isolation Date**		**Environment**	aquatic, brackish
**Order (Older Classification Scheme)**	Chroococcales	**Habitat Sample Description**	mesotidal zone, benthic
**Order 2014 (Modern Taxonomy)**	Synechococcales	**Ecophysiological traits**	
**Taxonomy Notes**		**Notes on ecophysiological traits**	
**Accession Number(s)_16S**	HM217052	**Isolator**	Viviana Lopes
**Accession Number(s)_others (product/gene /region)**		**Medium**	Z8
**Collector**	Viviana Lopes	**Strain Status**	non-axenic, unicyanobacterial
**Collection Date**	2006	**Preservation type**	subculturing
**Location**	Portugal: Minho estuary, Caminha	**Light:dark cycle**	12:12-h
**Latitude & Longitude**	41.86697 N 8.855833 W	**Temperature (ºC)**	19
		**Light intensity**	10–30 µmol photons m−2 s−1
**References Publicly Available Articles**	Lopes et al. (2012) Mar. Environ. Res. 73, 7–16		
**References Publicly Available Theses**	Lopes V.R. (2011) PhD Thesis, FCUP, University of Porto, Porto, Portugal		

**Strain ID**	**Synechococcus sp. LEGE 06324**		
**Strain Taxonomy**	Synechococcus Nägeli, C (1849)		
**Microphotograph 400×**	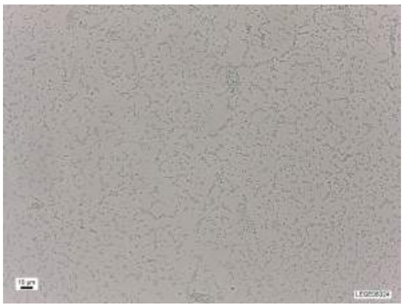	**Microphotograph 1000×**	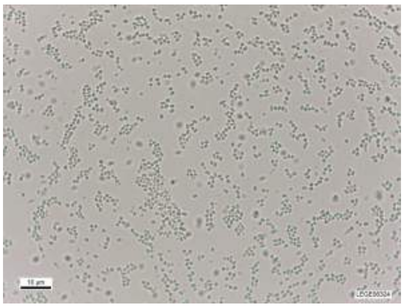
**Coidentity**	n.a.	**Morphometrics (Width × Length or Diameter) (µm)**	Ø 0,9±0,1
**Other Code(s)**		**Taxonomic notes/diacritical features**	rod-shaped cells; some elongated, involution cells observed
**Parent Sample**	CO85	**Macroscopic growth features**	color: cyano; growing homogeneously
**Isolation Date**		**Environment**	aquatic, brackish
**Order (Older Classification Scheme)**	Chroococcales	**Habitat Sample Description**	mesotidal zone, benthic
**Order 2014 (Modern Taxonomy)**	Synechococcales	**Ecophysiological traits**	
**Taxonomy Notes**		**Notes on ecophysiological traits**	
**Accession Number(s)_16S**	HM217046	**Isolator**	Viviana Lopes
**Accession Number(s)_others (product/gene /region)**		**Medium**	Z8
**Collector**	Viviana Lopes	**Strain Status**	non-axenic, unicyanobacterial
**Collection Date**	2006	**Preservation type**	subculturing
**Location**	Portugal: Minho estuary, Seixas	**Light:dark cycle**	12:12-h
**Latitude & Longitude**	41.88797 N 8.825722 W	**Temperature (ºC)**	19
		**Light intensity**	10–30 µmol photons m−2 s−1
**References Publicly Available Articles**	Lopes et al. (2012) Mar. Environ. Res. 73, 7–16		
**References Publicly Available Theses**	Lopes V.R. (2011) PhD Thesis, FCUP, University of Porto, Porto, Portugal		

Strain ID	**Synechococcus sp. LEGE 07172**		
**Strain Taxonomy**	Synechococcus Nägeli, C (1849)		
**Microphotograph 400×**	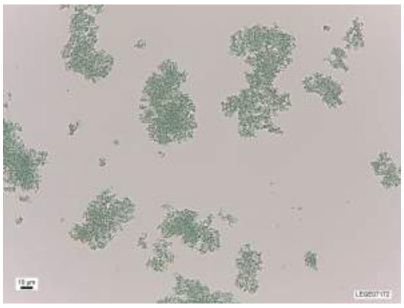	**Microphotograph 1000×**	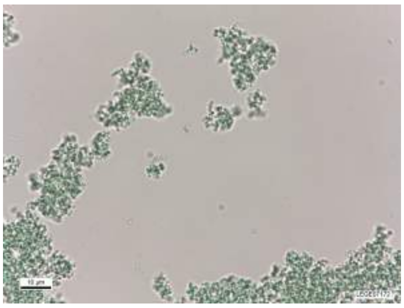
**Coidentity**	n.a.	**Morphometrics (Width × Length or Diameter) (µm)**	Ø 0,8±0,2
**Other Code(s)**	LEAN 086	**Taxonomic notes/diacritical features**	many cluster of cells; pseudofilaments observed
**Parent Sample**	P.07 ODA 4 - CA	**Macroscopic growth features**	color: cyano; forming a firm biofilm and also aggregates
**Isolation Date**		**Environment**	aquatic, marine
**Order (Older Classification Scheme)**	Chroococcales	**Habitat Sample Description**	tide puddle, on a submerged stone
**Order 2014 (Modern Taxonomy)**	not determined	**Ecophysiological traits**	
**Taxonomy Notes**		**Notes on ecophysiological traits**	
**Accession Number(s)_16S**	HQ832950	**Isolator**	Vitor Ramos
**Accession Number(s)_others (product/gene /region)**		**Medium**	Z8 25‰ TM sea salt w/ vit. B12
**Collector**	Rui Seabra/Vitor Ramos	**Strain Status**	non-axenic, unicyanobacterial
**Collection Date**	2007/05/02	**Preservation type**	subculturing
**Location**	Portugal: Praia de Olhos d'Água, Albufeira	**Light:dark cycle**	12:12-h
**Latitude & Longitude**	37.08973 N 8.191017 W	**Temperature (ºC)**	19
		**Light intensity**	10–30 µmol photons m−2 s−1
**References Publicly Available Articles**	Brito et al. (2012) Syst. Appl. Microbiol. 35, 110–119; Costa et al. (2014) Mar. Drugs 12, 98-114		
**References Publicly Available Theses**			

**Strain ID**	**Synechococcus sp. LEGE 11379**		
**Strain Taxonomy**	Synechococcus Nägeli, C (1849)		
**Microphotograph 400×**	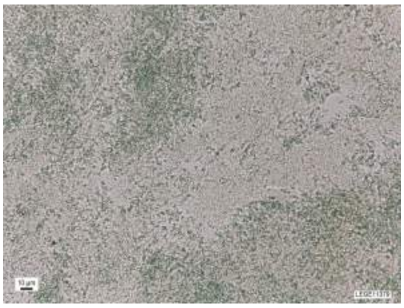	**Microphotograph 1000×**	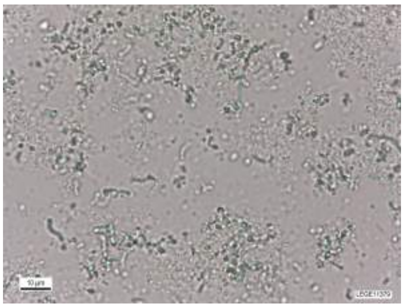
**Coidentity**	n.a.	**Morphometrics (Width × Length or Diameter) (µm)**	Ø 0,7±0,2 (Spherical Cells); 1,1±0,4 × 8,9±9,8 (Involution cells); 1,2±0,3 × 1,7±0,4 (Elongated cells)
**Other Code(s)**	19G	**Taxonomic notes/diacritical features**	some sigmoid, elongated, involution cells
**Parent Sample**		**Macroscopic growth features**	color: cyano; forming a smooth biofilm
**Isolation Date**		**Environment**	aquatic, marine
**Order (Older Classification Scheme)**	Chroococcales	**Habitat Sample Description**	intertidal zone, on a marine sponge
**Order 2014 (Modern Taxonomy)**	not determined	**Ecophysiological traits**	
**Taxonomy Notes**		**Notes on ecophysiological traits**	
**Accession Number(s)_16S**		**Isolator**	Ana Regueiras
**Accession Number(s)_others (product/gene /region)**		**Medium**	Z8 25‰ TM sea salt w/ vit. B12
**Collector**	Ana Regueiras	**Strain Status**	non-axenic, unicyanobacterial
**Collection Date**	2011	**Preservation type**	subculturing
**Location**	Portugal	**Light:dark cycle**	12:12-h
**Latitude & Longitude**	unknown	**Temperature (ºC)**	19
		**Light intensity**	10–30 µmol photons m−2 s−1
**References Publicly Available Articles**			
**References Publicly Available Theses**			

**Strain ID**	**Synechococcus sp. LEGE 11381**		
**Strain Taxonomy**	Synechococcus Nägeli, C (1849)		
**Microphotograph 400×**	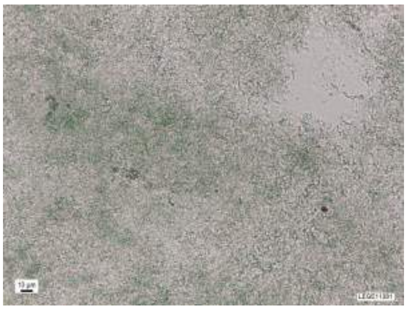	**Microphotograph 1000×**	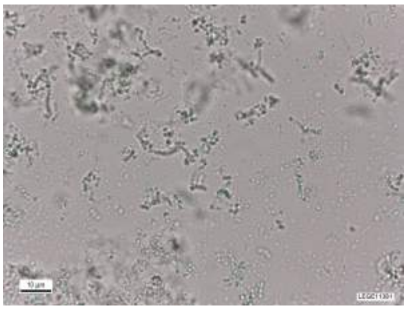
**Coidentity**	n.a.	**Morphometrics (Width × Length or Diameter) (µm)**	Ø 0,7±0,1 (Spherical Cells); 1,2±0,2 × 17,0±10,0 (Involution cells); 1,0±0,1 × 1,2±0,2 (Elongated cells)
**Other Code(s)**	21C	**Taxonomic notes/diacritical features**	some elongated, involution cells observed
**Parent Sample**		**Macroscopic growth features**	color: cyano
**Isolation Date**		**Environment**	aquatic, marine
**Order (Older Classification Scheme)**	Chroococcales	**Habitat Sample Description**	intertidal zone, on a marine sponge
**Order 2014 (Modern Taxonomy)**	Synechococcales	**Ecophysiological traits**	
**Taxonomy Notes**		**Notes on ecophysiological traits**	
**Accession Number(s)_16S**	JQ927352	**Isolator**	Ana Regueiras
**Accession Number(s)_others (product/gene /region)**		**Medium**	Z8 25‰ TM sea salt w/ vit. B12
**Collector**	Ana Regueiras	**Strain Status**	non-axenic, unicyanobacterial
**Collection Date**	2011/07/18	**Preservation type**	subculturing
**Location**	Portugal: Praia da Memória, Matosinhos	**Light:dark cycle**	12:12-h
**Latitude & Longitude**	41.23119 N 8.721750 W	**Temperature (ºC)**	19
		**Light intensity**	10–30 µmol photons m−2 s−1
**References Publicly Available Articles**			
**References Publicly Available Theses**			

Strain ID	**Synechococcus sp. LEGE 11394**		
**Strain Taxonomy**	Synechococcus Nägeli, C (1849)		
**Microphotograph 400×**	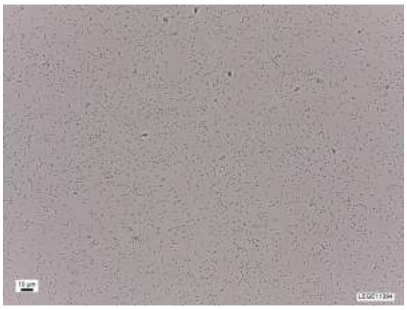	**Microphotograph 1000×**	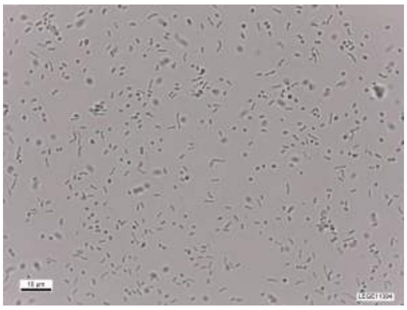
**Coidentity**	n.a.	**Morphometrics (Width × Length or Diameter) (µm)**	Ø 1,1±0,1 (Cells); 0,6±0,1 × 3,2±0,8 (Involution cells);
**Other Code(s)**		**Taxonomic notes/diacritical features**	some elongated, involution cells observed
**Parent Sample**	EB3-I3-A (JM)	**Macroscopic growth features**	color: cyano; growing homogeneously
**Isolation Date**		**Environment**	aquatic, hypersaline
**Order (Older Classification Scheme)**	Chroococcales	**Habitat Sample Description**	upper layer of a microbial mat
**Order 2014 (Modern Taxonomy)**	Synechococcales	**Ecophysiological traits**	
**Taxonomy Notes**		**Notes on ecophysiological traits**	
**Accession Number(s)_16S**	KU951806	**Isolator**	Vitor Ramos
**Accession Number(s)_others (product/gene /region)**		**Medium**	MN w/ vit. B12
**Collector**	Frederico Sobrinho/Sinda Carvalhal	**Strain Status**	non-axenic, unicyanobacterial
**Collection Date**	9-Feb-2011	**Preservation type**	subculturing
**Location**	Brazil: Pitanguinha lagoon (Araruama system), in the state of Rio de Janeiro	**Light:dark cycle**	12:12-h
**Latitude & Longitude**	22.9275 S 42.35556 W	**Temperature (ºC)**	19
		**Light intensity**	10–30 µmol photons m−2 s−1
**References Publicly Available Articles**			
**References Publicly Available Theses**			

Strain ID	**Synechococcus sp. LEGE 11428**		
**Strain Taxonomy**	Synechococcus Nägeli, C (1849)		
**Microphotograph 400×**	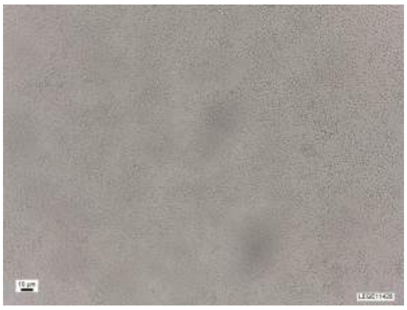	**Microphotograph 1000×**	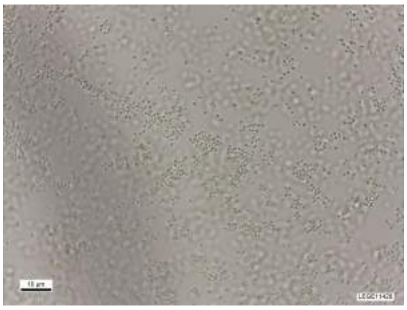
**Coidentity**	n.a.	**Morphometrics (Width × Length or Diameter) (µm)**	Ø 0,9±0,1
**Other Code(s)**		**Taxonomic notes/diacritical features**	pseudofilaments observed
**Parent Sample**	8.003.1011_AP.G	**Macroscopic growth features**	color: cyano; forming aggregates
**Isolation Date**		**Environment**	aquatic, marine
**Order (Older Classification Scheme)**	Chroococcales	**Habitat Sample Description**	subtidal sample, epilithic (13m depth), about 200 m off the shore
**Order 2014 (Modern Taxonomy)**	Synechococcales	**Ecophysiological traits**	
**Taxonomy Notes**		**Notes on ecophysiological traits**	
**Accession Number(s)_16S**	ready to submit	**Isolator**	Ângela Pinheiro
**Accession Number(s)_others (product/gene /region)**		**Medium**	Z8 25‰ TM sea salt w/ vit. B12
**Collector**	Pedro Leão/Aldo Barreiro	**Strain Status**	non-axenic, unicyanobacterial
**Collection Date**	2011/10/03	**Preservation type**	subculturing
**Location**	Portugal: 'A Pedra', diving spot in front of the fort 'Castelo do Queijo'	**Light:dark cycle**	12:12-h
**Latitude & Longitude**	41.185809 N 8.719079 W	**Temperature (ºC)**	19
**References Publicly Available Articles**		**Light intensity**	10–30 µmol photons m−2 s−1
**References Publicly Available Theses**			

Strain ID	**Synechococcus sp. LEGE 11466**		
**Strain Taxonomy**	Synechococcus Nägeli, C (1849)		
**Microphotograph 400×**	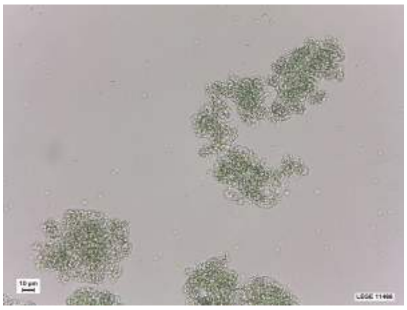	**Microphotograph 1000×**	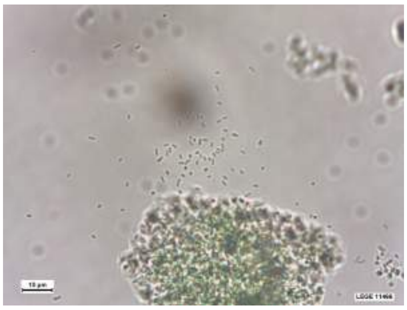
**Coidentity**	n.a.	**Morphometrics (Width × Length or Diameter) (µm)**	Ø 0,7±0,1
**Other Code(s)**		**Taxonomic notes/diacritical features**	rod-shaped cells; some cell clusters
**Parent Sample**	3.002.0911	**Macroscopic growth features**	color: cyano; growing homogeneously
**Isolation Date**		**Environment**	aquatic, marine
**Order (Older Classification Scheme)**	Chroococcales	**Habitat Sample Description**	subtidal sample, epilithic (10-13m depth), less than 1 km off the shore
**Order 2014 (Modern Taxonomy)**	Synechococcales	**Ecophysiological traits**	
**Taxonomy Notes**		**Notes on ecophysiological traits**	
**Accession Number(s)_16S**	KU951807	**Isolator**	Sébastien Brûle/Ângela Pinheiro
**Accession Number(s)_others (product/gene /region)**		**Medium**	Z8 25‰ TM sea salt w/ vit. B12
**Collector**	Pedro Leão/Aldo Barreiro	**Strain Status**	non-axenic, unicyanobacterial
**Collection Date**	2011/09/26	**Preservation type**	subculturing
**Location**	Portugal: 'Pêlo Negro', diving spot near Leixões Harbour	**Light:dark cycle**	12:12-h
**Latitude & Longitude**	41.18557 N 8.719100 W	**Temperature (ºC)**	19
		**Light intensity**	10–30 µmol photons m−2 s−1
**References Publicly Available Articles**			
**References Publicly Available Theses**			

Strain ID	**Synechococcus sp. LEGE 11471**		
**Strain Taxonomy**	Synechococcus Nägeli, C (1849)		
**Microphotograph 400×**	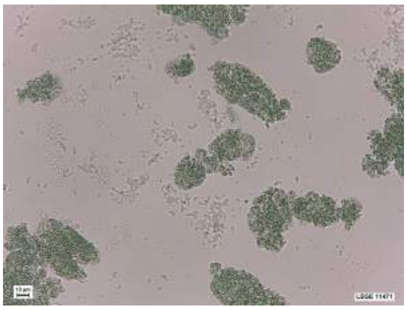	**Microphotograph 1000×**	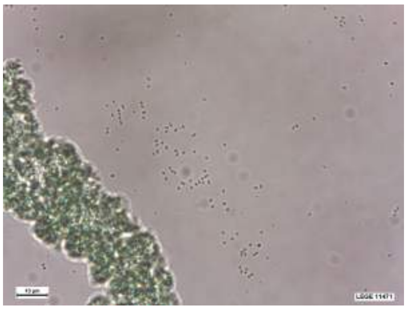
**Coidentity**	n.a.	**Morphometrics (Width × Length or Diameter) (µm)**	Ø 0,8±0,1
**Other Code(s)**		**Taxonomic notes/diacritical features**	shortly rod-shaped cells
**Parent Sample**	11.002.0911	**Macroscopic growth features**	color: cyano; forming aggregates
**Isolation Date**		**Environment**	aquatic, marine
**Order (Older Classification Scheme)**	Chroococcales	**Habitat Sample Description**	subtidal sample, epilithic (10-13m depth), less than 1 km off the shore
**Order 2014 (Modern Taxonomy)**	Synechococcales	**Ecophysiological traits**	
**Taxonomy Notes**		**Notes on ecophysiological traits**	
**Accession Number(s)_16S**	ready to submit	**Isolator**	Ângela Pinheiro
**Accession Number(s)_others (product/gene /region)**		**Medium**	Z8 25‰ TM sea salt w/ vit. B12
**Collector**	Pedro Leão/Aldo Barreiro	**Strain Status**	non-axenic, unicyanobacterial
**Collection Date**	2011/09/26	**Preservation type**	subculturing
**Location**	Portugal: 'Pêlo Negro', diving spot near Leixões Harbour	**Light:dark cycle**	12:12-h
**Latitude & Longitude**	41.18557 N 8.719100 W	**Temperature (ºC)**	19
		**Light intensity**	10–30 µmol photons m−2 s−1
**References Publicly Available Articles**			
**References Publicly Available Theses**			

Strain ID	**Synechocystis salina LEGE 00027**		
**Strain Taxonomy**	Synechocystis salina Wislouch (1924)		
**Microphotograph 400×**	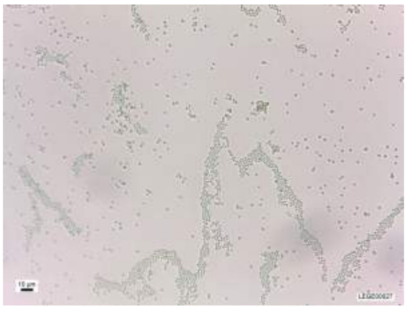	**Microphotograph 1000×**	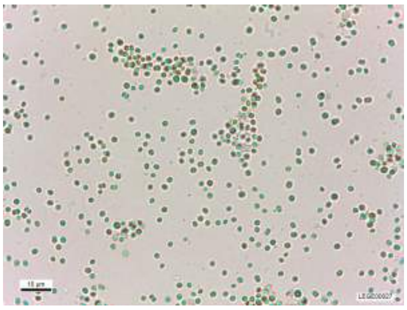
**Coidentity**	n.a.	**Morphometrics (Width × Length or Diameter) (µm)**	Ø 2,0±0,3
**Other Code(s)**	strain 17, LEANCYA 17, LEAN 201	**Taxonomic notes/diacritical features**	
**Parent Sample**	Burgau	**Macroscopic growth features**	color: cyano; forming a smooth biofilm
**Isolation Date**		**Environment**	aquatic, marine
**Order (Older Classification Scheme)**	Chroococcales	**Habitat Sample Description**	intertidal zone, biofilm on a rock
**Order 2014 (Modern Taxonomy)**	Chroococcales**	**Ecophysiological traits**	
**Taxonomy Notes**	**inconsistent; assignment supported by phylogeny	**Notes on ecophysiological traits**	
**Accession Number(s)_16S**	KU951808	**Isolator**	Rosario Martins
**Accession Number(s)_others (product/gene /region)**		**Medium**	Z8 25‰ TM sea salt w/ vit. B12
**Collector**	Rosario Martins	**Strain Status**	non-axenic, unicyanobacterial
**Collection Date**	2000/09/02	**Preservation type**	subculturing
**Location**	Portugal: Praia de Burgau, Budens	**Light:dark cycle**	12:12-h
**Latitude & Longitude**	37.07156 N 8.774722 W	**Temperature (ºC)**	19
		**Light intensity**	10–30 µmol photons m−2 s−1
**References Publicly Available Articles**	Martins et al. (2005) Toxicon 46, 454–464; Martins et al. (2007) Toxicon 50, 791–799; Martins et al. (2008) Mar. Drugs 6(1), 1-11		
**References Publicly Available Theses**	Martins R. (2005) PhD Thesis, FCUP, University of Porto, Porto, Portugal		

**Strain ID**	**Synechocystis salina LEGE 00028**		
**Strain Taxonomy**	Synechocystis salina Wislouch (1924)		
**Microphotograph 400×**	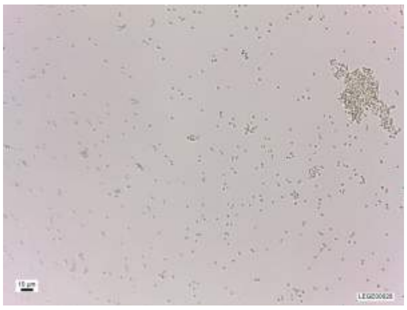	**Microphotograph 1000×**	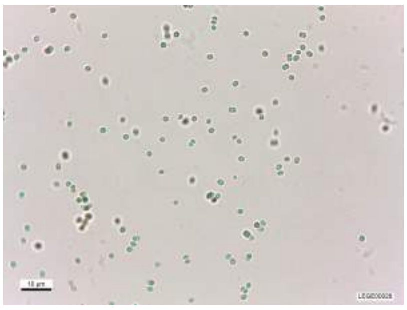
**Coidentity**	n.a.	**Morphometrics (Width × Length or Diameter) (µm)**	Ø 1,8±0,2
**Other Code(s)**	strain 18, LEANCYA 18, LEAN 202	**Taxonomic notes/diacritical features**	
**Parent Sample**	Tamariz	**Macroscopic growth features**	color: cyano; growing homogeneously; mucilaginous
**Isolation Date**		**Environment**	aquatic, marine
**Order (Older Classification Scheme)**	Chroococcales	**Habitat Sample Description**	sea water sample, intertidal zone, from a tide pool
**Order 2014 (Modern Taxonomy)**	Chroococcales**	**Ecophysiological traits**	EPS production
**Taxonomy Notes**	**inconsistent; assignment supported by phylogeny	**Notes on ecophysiological traits**	
**Accession Number(s)_16S**	KU951809	**Isolator**	Rosario Martins
**Accession Number(s)_others (product/gene /region)**		**Medium**	Z8 25‰ TM sea salt w/ vit. B12
**Collector**	Rosario Martins	**Strain Status**	non-axenic, unicyanobacterial
**Collection Date**	2000/07/22	**Preservation type**	subculturing
**Location**	Portugal: Praia do Tamariz, Estoril	**Light:dark cycle**	12:12-h
**Latitude & Longitude**	38.70287 N 9.399350 W	**Temperature (ºC)**	19
		**Light intensity**	10–30 µmol photons m−2 s−1
**References Publicly Available Articles**	Martins et al. (2005) Toxicon 46, 454–464; Martins et al. (2007) Toxicon 50, 791–799; Martins et al. (2008) Mar. Drugs 6(1), 1-11		
**References Publicly Available Theses**	Martins R. (2005) PhD Thesis, FCUP, University of Porto, Porto, Portugal		

**Strain ID**	**Synechocystis salina LEGE 00029**		
**Strain Taxonomy**	Synechocystis salina Wislouch (1924)		
**Microphotograph 400×**	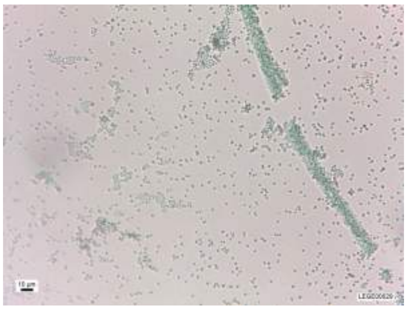	**Microphotograph 1000×**	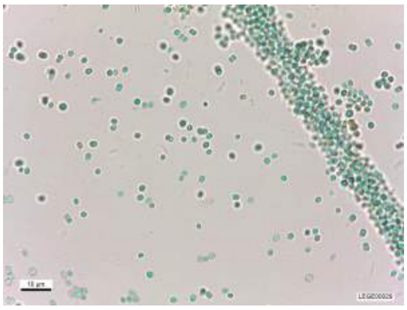
**Coidentity**	n.a.	**Morphometrics (Width × Length or Diameter) (µm)**	Ø 1,9±0,2
**Other Code(s)**	strain 19, LEANCYA 19, LEAN 203	**Taxonomic notes/diacritical features**	
**Parent Sample**	Martinhal (fil.)	**Macroscopic growth features**	color: cyano; growing homogeneously; mucilaginous
**Isolation Date**		**Environment**	aquatic, marine
**Order (Older Classification Scheme)**	Chroococcales	**Habitat Sample Description**	sea water sample, intertidal zone, from a tide pool
**Order 2014 (Modern Taxonomy)**	Chroococcales**	**Ecophysiological traits**	EPS production
**Taxonomy Notes**	**inconsistent; assignment supported by phylogeny	**Notes on ecophysiological traits**	
**Accession Number(s)_16S**	KU951810	**Isolator**	Rosario Martins
**Accession Number(s)_others (product/gene /region)**		**Medium**	Z8 25‰ TM sea salt w/ vit. B12
**Collector**	Rosario Martins	**Strain Status**	non-axenic, unicyanobacterial
**Collection Date**	2000/09/03	**Preservation type**	subculturing
**Location**	Portugal: Praia do Martinhal, Vila do Bispo	**Light:dark cycle**	12:12-h
**Latitude & Longitude**	37.01869 N 8.926714 W	**Temperature (ºC)**	19
		**Light intensity**	10–30 µmol photons m−2 s−1
**References Publicly Available Articles**	Martins et al. (2005) Toxicon 46, 454–464; Martins et al. (2007) Toxicon 50, 791–799; Martins et al. (2008) Mar. Drugs 6(1), 1-11		
**References Publicly Available Theses**	Martins R. (2005) PhD Thesis, FCUP, University of Porto, Porto, Portugal		

Strain ID	**Synechocystis salina LEGE 00030**		
**Strain Taxonomy**	Synechocystis salina Wislouch (1924)		
**Microphotograph 400×**	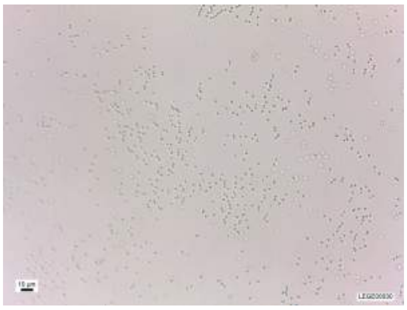	**Microphotograph 1000×**	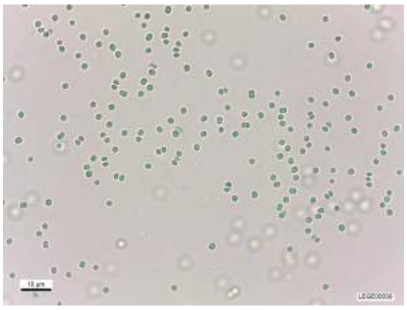
**Coidentity**	n.a.	**Morphometrics (Width × Length or Diameter) (µm)**	Ø 1,8±0,2
**Other Code(s)**	LEAN 204	**Taxonomic notes/diacritical features**	
**Parent Sample**	Aguda 1 planct.	**Macroscopic growth features**	color: cyano; growing homogeneously
**Isolation Date**		**Environment**	aquatic, marine
**Order (Older Classification Scheme)**	Chroococcales	**Habitat Sample Description**	sea water sample, intertidal zone, from a tide pool
**Order 2014 (Modern Taxonomy)**	Chroococcales**	**Ecophysiological traits**	
**Taxonomy Notes**	**inconsistent; assignment supported by phylogeny	**Notes on ecophysiological traits**	
**Accession Number(s)_16S**	KU951811	**Isolator**	Rosario Martins
**Accession Number(s)_others (product/gene /region)**		**Medium**	Z8 25‰ TM sea salt w/ vit. B12
**Collector**	Rosario Martins	**Strain Status**	non-axenic, unicyanobacterial
**Collection Date**	2000/05/31	**Preservation type**	subculturing
**Location**	Portugal: Praia da Aguda, Arcozelo	**Light:dark cycle**	12:12-h
**Latitude & Longitude**	41.04954 N 8.655339 W	**Temperature (ºC)**	19
		**Light intensity**	10–30 µmol photons m−2 s−1
**References Publicly Available Articles**			
**References Publicly Available Theses**			

**Strain ID**	**Synechocystis salina LEGE 00031**		
**Strain Taxonomy**	Synechocystis salina Wislouch (1924)		
**Microphotograph 400×**	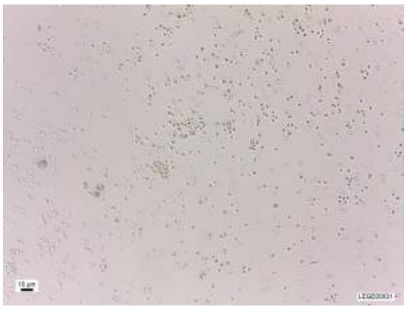	**Microphotograph 1000×**	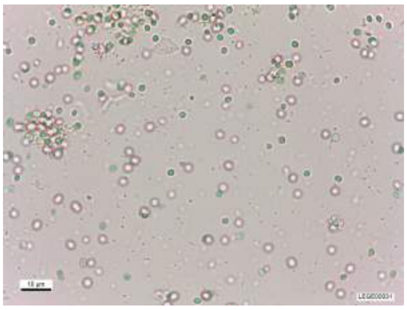
**Coidentity**	n.a.	**Morphometrics (Width × Length or Diameter) (µm)**	Ø 2,1±0,2
**Other Code(s)**	strain 5, LEANCYA 5, LEAN 205	**Taxonomic notes/diacritical features**	
**Parent Sample**	Moledo 1	**Macroscopic growth features**	color: cyano; forming aggregates
**Isolation Date**		**Environment**	aquatic, marine
**Order (Older Classification Scheme)**	Chroococcales	**Habitat Sample Description**	intertidal zone, Sabellaria alveolata reef
**Order 2014 (Modern Taxonomy)**	Chroococcales**	**Ecophysiological traits**	
**Taxonomy Notes**	**inconsistent; assignment supported by phylogeny	**Notes on ecophysiological traits**	
**Accession Number(s)_16S**	KU951812	**Isolator**	Rosario Martins
**Accession Number(s)_others (product/gene /region)**		**Medium**	Z8 25‰ TM sea salt w/ vit. B12
**Collector**	Rosario Martins	**Strain Status**	non-axenic, unicyanobacterial
**Collection Date**	2000/06/18	**Preservation type**	subculturing
**Location**	Portugal: Praia de Moledo, Caminha	**Light:dark cycle**	12:12-h
**Latitude & Longitude**	41.84963 N 8.866717 W	**Temperature (ºC)**	19
		**Light intensity**	10–30 µmol photons m−2 s−1
**References Publicly Available Articles**	Martins et al. (2005) Toxicon 46, 454–464; Martins et al. (2007) Toxicon 50, 791–799; Martins et al. (2008) Mar. Drugs 6(1), 1-11		
**References Publicly Available Theses**	Martins R. (2005) PhD Thesis, FCUP, University of Porto, Porto, Portugal		

**Strain ID**	**Synechocystis salina LEGE 00032**		
**Strain Taxonomy**	Synechocystis salina Wislouch (1924)		
**Microphotograph 400×**	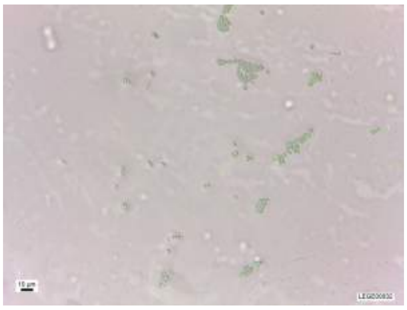	**Microphotograph 1000×**	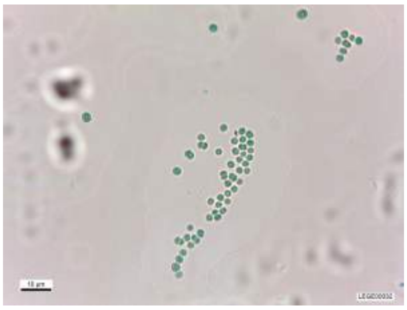
**Coidentity**	n.a.	**Morphometrics (Width × Length or Diameter) (µm)**	Ø 2,0±0,2
**Other Code(s)**	strain LEANCYA 21, LEAN 206	**Taxonomic notes/diacritical features**	
**Parent Sample**	Praia da Luz	**Macroscopic growth features**	color: cyano; forming a smooth biofilm
**Isolation Date**		**Environment**	aquatic, marine
**Order (Older Classification Scheme)**	Chroococcales	**Habitat Sample Description**	intertidal zone, marine sponge
**Order 2014 (Modern Taxonomy)**	Chroococcales**	**Ecophysiological traits**	
**Taxonomy Notes**	**inconsistent; assignment supported by phylogeny	**Notes on ecophysiological traits**	
**Accession Number(s)_16S**	KU951813	**Isolator**	Rosario Martins
**Accession Number(s)_others (product/gene /region)**		**Medium**	Z8 25‰ TM sea salt w/ vit. B12
**Collector**	Rosario Martins	**Strain Status**	non-axenic, unicyanobacterial
**Collection Date**	2000/09/02	**Preservation type**	subculturing
**Location**	Portugal: Praia da Luz, Lagos	**Light:dark cycle**	12:12-h
**Latitude & Longitude**	37.08622 N 8.725375 W	**Temperature (ºC)**	19
		**Light intensity**	10–30 µmol photons m−2 s−1
**References Publicly Available Articles**	Martins et al. (2005) Toxicon 46, 454–464; Martins et al. (2007) Toxicon 50, 791–799; Martins et al. (2008) Mar. Drugs 6(1), 1-11; Ribeiro et al. (2008) Int. J. Environ. Anal. Chem. 88(10), 701-710		
**References Publicly Available Theses**	Martins R. (2005) PhD Thesis, FCUP, University of Porto, Porto, Portugal		

Strain ID

Synechocystis salina LEGE 00036

**Strain Taxonomy**	Synechocystis salina Wislouch (1924)		
**Microphotograph 400×**	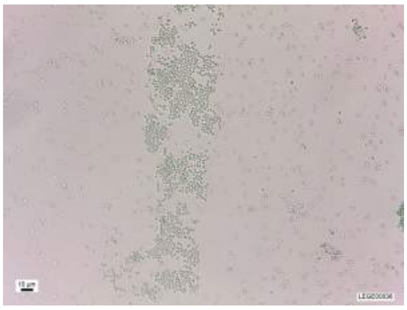	**Microphotograph 1000×**	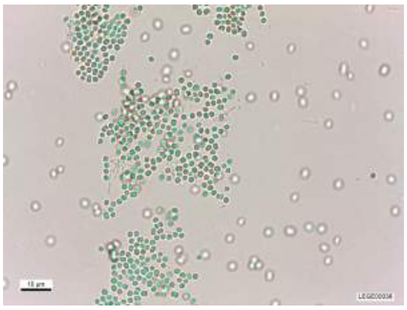
**Coidentity**	n.a.	**Morphometrics (Width × Length or Diameter) (µm)**	Ø 1,5±0,1
**Other Code(s)**	strain 16, LEANCYA 16, LEAN 210	**Taxonomic notes/diacritical features**	
**Parent Sample**	Burgau 11	**Macroscopic growth features**	color: cyano; growing homogeneously
**Isolation Date**		**Environment**	aquatic, marine
**Order (Older Classification Scheme)**	Chroococcales	**Habitat Sample Description**	intertidal zone, biofilm on a rock
**Order 2014 (Modern Taxonomy)**	Chroococcales**	**Ecophysiological traits**	
**Taxonomy Notes**	**inconsistent; assignment supported by phylogeny	**Notes on ecophysiological traits**	
**Accession Number(s)_16S**	KU951814	**Isolator**	Rosario Martins
**Accession Number(s)_others (product/gene /region)**		**Medium**	Z8 25‰ TM sea salt w/ vit. B12
**Collector**	Rosario Martins	**Strain Status**	non-axenic, unicyanobacterial
**Collection Date**	2000/09/02	**Preservation type**	subculturing
**Location**	Portugal: Praia de Burgau, Budens	**Light:dark cycle**	12:12-h
**Latitude & Longitude**	37.07156 N 8.774722 W	**Temperature (ºC)**	19
		**Light intensity**	10–30 µmol photons m−2 s−1
**References Publicly Available Articles**	Martins et al. (2005) Toxicon 46, 454–464; Martins et al. (2007) Toxicon 50, 791–799; Martins et al. (2008) Mar. Drugs 6(1), 1-11		
**References Publicly Available Theses**	Martins R. (2005) PhD Thesis, FCUP, University of Porto, Porto, Portugal		

**Strain ID**	**Synechocystis salina LEGE 00037**		
**Strain Taxonomy**	Synechocystis salina Wislouch (1924)		
**Microphotograph 400×**	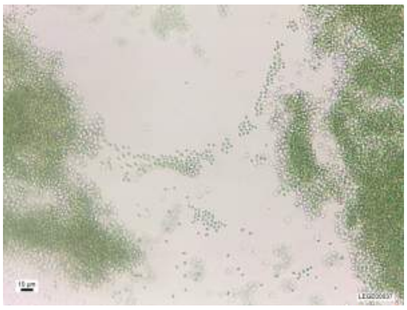	**Microphotograph 1000×**	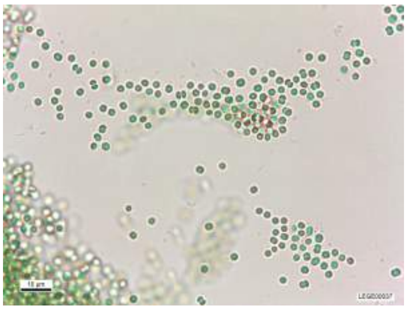
**Coidentity**	n.a.	**Morphometrics (Width × Length or Diameter) (µm)**	Ø 2,3±0,3
**Other Code(s)**	strain 1, LEANCYA 1, LEAN 211	**Taxonomic notes/diacritical features**	
**Parent Sample**	Moledo 7	**Macroscopic growth features**	color: cyano; forming aggregates
**Isolation Date**		**Environment**	aquatic, marine
**Order (Older Classification Scheme)**	Chroococcales	**Habitat Sample Description**	intertidal zone, microbial mat on a Sabellaria alveolata reef
**Order 2014 (Modern Taxonomy)**	Chroococcales**	**Ecophysiological traits**	
**Taxonomy Notes**	**inconsistent; assignment supported by phylogeny	**Notes on ecophysiological traits**	
**Accession Number(s)_16S**	KU951815	**Isolator**	Rosario Martins
**Accession Number(s)_others (product/gene /region)**		**Medium**	Z8 25‰ TM sea salt w/ vit. B12
**Collector**	Rosario Martins	**Strain Status**	non-axenic, unicyanobacterial
**Collection Date**	2000/07/15	**Preservation type**	subculturing
**Location**	Portugal: Praia de Moledo, Caminha	**Light:dark cycle**	12:12-h
**Latitude & Longitude**	41.84963 N 8.866717 W	**Temperature (ºC)**	19
		**Light intensity**	10–30 µmol photons m−2 s−1
**References Publicly Available Articles**	Martins et al. (2005) Toxicon 46, 454–464; Selheim et al. (2005) Aquat. Toxicol. 74, 294–306; Martins et al. (2007) Toxicon 50, 791–799; Martins et al. (2008) Mar. Drugs 6(1), 1-11		
**References Publicly Available Theses**	Martins R. (2005) PhD Thesis, FCUP, University of Porto, Porto, Portugal		

Strain ID	**Synechocystis salina LEGE 00038**		
**Strain Taxonomy**	Synechocystis salina Wislouch (1924)		
**Microphotograph 400×**	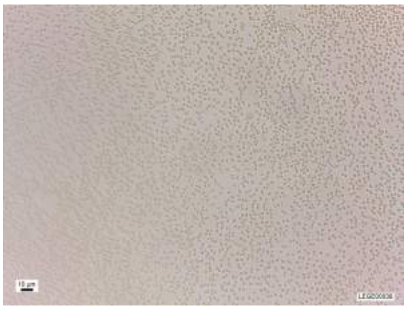	**Microphotograph 1000×**	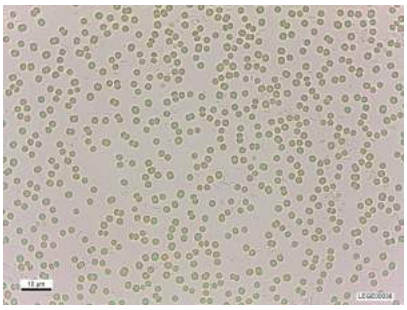
**Coidentity**	n.a.	**Morphometrics (Width × Length or Diameter) (µm)**	Ø 1,9±0,1
**Other Code(s)**	LEAN 212	**Taxonomic notes/diacritical features**	
**Parent Sample**	Mindelo 13	**Macroscopic growth features**	color: cyano; forming a smooth biofilm; mucilaginous
**Isolation Date**		**Environment**	aquatic, marine
**Order (Older Classification Scheme)**	Chroococcales	**Habitat Sample Description**	intertidal zone, Sabellaria alveolata reef
**Order 2014 (Modern Taxonomy)**	Chroococcales**	**Ecophysiological traits**	EPS production
**Taxonomy Notes**	**inconsistent; assignment supported by phylogeny	**Notes on ecophysiological traits**	
**Accession Number(s)_16S**	KU951816	**Isolator**	Rosario Martins
**Accession Number(s)_others (product/gene /region)**		**Medium**	Z8 25‰ TM sea salt w/ vit. B12
**Collector**	Rosario Martins	**Strain Status**	non-axenic, unicyanobacterial
**Collection Date**	2000/06/18	**Preservation type**	subculturing
**Location**	Portugal: Praia do Mindelo, Vila do Conde	**Light:dark cycle**	12:12-h
**Latitude & Longitude**	41.30995 N 8.740322 W	**Temperature (ºC)**	19
		**Light intensity**	10–30 µmol photons m−2 s−1
**References Publicly Available Articles**			
**References Publicly Available Theses**			

Strain ID	**Synechocystis salina LEGE 00040**		
**Strain Taxonomy**	Synechocystis salina Wislouch (1924)		
**Microphotograph 400×**	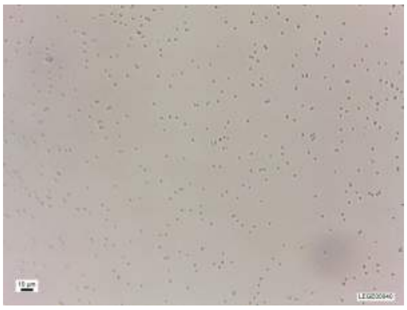	**Microphotograph 1000×**	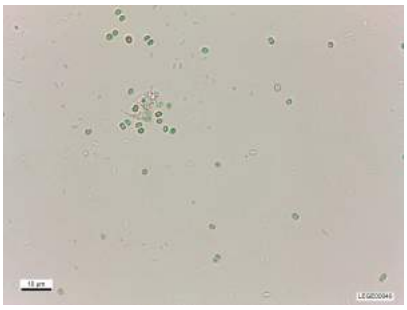
**Coidentity**	n.a.	**Morphometrics (Width × Length or Diameter) (µm)**	Ø 1,9±0,2
**Other Code(s)**	LEAN 214	**Taxonomic notes/diacritical features**	
**Parent Sample**	VPA 4 (n)	**Macroscopic growth features**	color: cyano; growing homogeneously, but also forming aggregates
**Isolation Date**		**Environment**	aquatic, marine
**Order (Older Classification Scheme)**	Chroococcales	**Habitat Sample Description**	intertidal zone, on a Patella sp. shell
**Order 2014 (Modern Taxonomy)**	Chroococcales**	**Ecophysiological traits**	
**Taxonomy Notes**	**inconsistent; assignment supported by phylogeny	**Notes on ecophysiological traits**	
**Accession Number(s)_16S**	KU951817	**Isolator**	Rosario Martins
**Accession Number(s)_others (product/gene /region)**		**Medium**	Z8 25‰ TM sea salt w/ vit. B12
**Collector**	Rosario Martins	**Strain Status**	non-axenic, unicyanobacterial
**Collection Date**	2000/07/15	**Preservation type**	subculturing
**Location**	Portugal: Praia de Vila Praia de Âncora, Caminha	**Light:dark cycle**	12:12-h
**Latitude & Longitude**	41.81427 N 8.865131 W	**Temperature (ºC)**	19
		**Light intensity**	10–30 µmol photons m−2 s−1
**References Publicly Available Articles**			
**References Publicly Available Theses**			

Strain ID	**Synechocystis salina LEGE 00041**		
**Strain Taxonomy**	Synechocystis salina Wislouch (1924)		
**Microphotograph 400×**	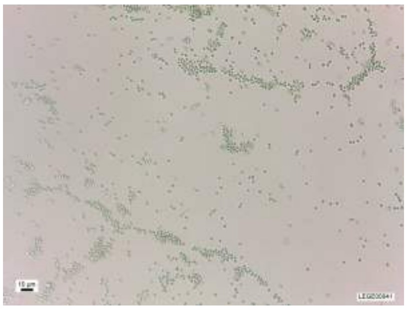	**Microphotograph 1000×**	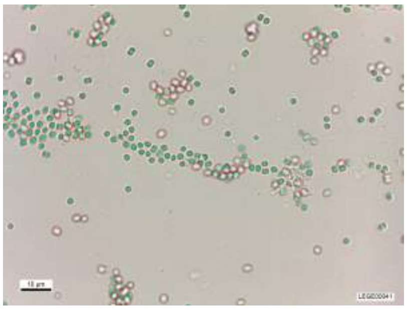
**Coidentity**	n.a.	**Morphometrics (Width × Length or Diameter) (µm)**	Ø 1,9±0,5
**Other Code(s)**	strain 7, LEANCYA 7, LEAN 215	**Taxonomic notes/diacritical features**	
**Parent Sample**	Espinho 4 planct.	**Macroscopic growth features**	color: cyano; forming a smooth biofilm; mucilaginous
**Isolation Date**		**Environment**	aquatic, marine
**Order (Older Classification Scheme)**	Chroococcales	**Habitat Sample Description**	sea water sample, intertidal zone, from a tide pool
**Order 2014 (Modern Taxonomy)**	Chroococcales**	**Ecophysiological traits**	EPS production
**Taxonomy Notes**	**inconsistent; assignment supported by phylogeny	**Notes on ecophysiological traits**	
**Accession Number(s)_16S**	KU951818	**Isolator**	Rosario Martins
**Accession Number(s)_others (product/gene /region)**		**Medium**	Z8 25‰ TM sea salt w/ vit. B12
**Collector**	Rosario Martins	**Strain Status**	non-axenic, unicyanobacterial
**Collection Date**	2000/06/02	**Preservation type**	subculturing, cryopreserved
**Location**	Portugal: Praia de Espinho	**Light:dark cycle**	12:12-h
**Latitude & Longitude**	41.00847 N 8.646958 W	**Temperature (ºC)**	19
		**Light intensity**	10–30 µmol photons m−2 s−1
**References Publicly Available Articles**	Martins et al. (2005) Toxicon 46, 454–464; Martins et al. (2007) Toxicon 50, 791–799; Martins et al. (2008) Mar. Drugs 6(1), 1-11		
**References Publicly Available Theses**	Martins R. (2005) PhD Thesis, FCUP, University of Porto, Porto, Portugal		

Strain ID	**Synechocystis salina LEGE 06099**		
**Strain Taxonomy**	Synechocystis salina Wislouch (1924)		
**Microphotograph 400×**	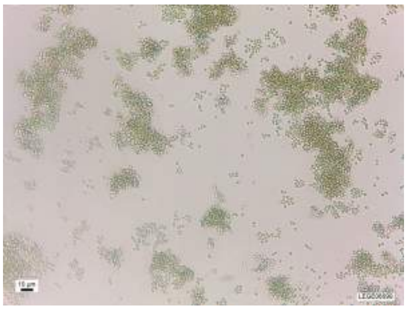	**Microphotograph 1000×**	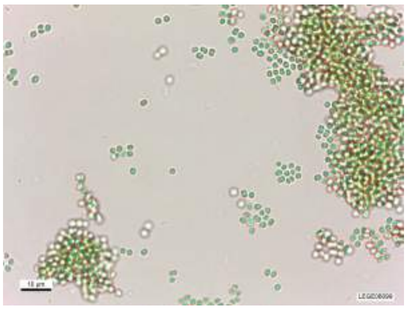
**Coidentity**	n.a.	**Morphometrics (Width × Length or Diameter) (µm)**	Ø 1,8±0,2
**Other Code(s)**	LEAN 003	**Taxonomic notes/diacritical features**	cell ultrastructure data available
**Parent Sample**	O.06 MOL 19 - GADA	**Macroscopic growth features**	color: cyano; forming aggregates
**Isolation Date**		**Environment**	aquatic, marine
**Order (Older Classification Scheme)**	Chroococcales	**Habitat Sample Description**	wave-exposed tide pool, rock surface scraping
**Order 2014 (Modern Taxonomy)**	Chroococcales**	**Ecophysiological traits**	
**Taxonomy Notes**	**inconsistent; assignment supported by phylogeny	**Notes on ecophysiological traits**	bartolosides and dehydroabietic acid production (unknown ecophysiological role)
**Accession Number(s)_16S**	KU951819	**Isolator**	Vitor Ramos
**Accession Number(s)_others (product/gene /region)**	KC813168, KC813169 (non-ribosomal peptide synthetase), KC842284 (polyketide synthase)	**Medium**	Z8 25‰ TM sea salt w/ vit. B12
**Collector**	Rui Seabra/Vitor Ramos	**Strain Status**	non-axenic, unicyanobacterial
**Collection Date**	2006/11/20	**Preservation type**	subculturing
**Location**	Portugal: Praia de Moledo, Caminha	**Light:dark cycle**	12:12-h
**Latitude & Longitude**	41.84963 N 8.866717 W	**Temperature (ºC)**	19
		**Light intensity**	10–30 µmol photons m−2 s−1
**References Publicly Available Articles**	Lopes & Vasconcelos (2011) Mar. Drugs 9, 790-802; Brito et al. (2012) Syst. Appl. Microbiol. 35, 110–119; Costa et al. (2014) Mar. Drugs 12, 98-114; Brito et al. (2015) Algal Res. 9, 218-226; Costa et al. (2016) Sci. Rep. 6, 23436		
**References Publicly Available Theses**	Afonso T. (2014) MSc Dissertation, FCUP, University of Porto, Porto, Portugal; Couto M.S. (2015) MSc Dissertation, ESTSP, Polytechnic Institute of Porto, Porto, Portugal		

Strain ID	**Synechocystis salina LEGE 06155**		
**Strain Taxonomy**	Synechocystis salina Wislouch (1924)		
**Microphotograph 400×**	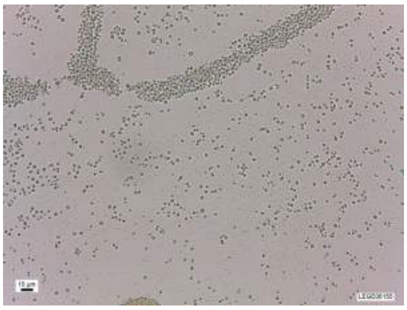	**Microphotograph 1000×**	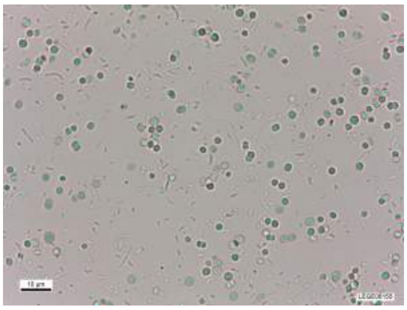
**Coidentity**	n.a.	**Morphometrics (Width × Length or Diameter) (µm)**	Ø 2,1±0,2
**Other Code(s)**	LEAN 069	**Taxonomic notes/diacritical features**	
**Parent Sample**	O.06 BAR 8 - E	**Macroscopic growth features**	color: cyano; forming a firm biofilm
**Isolation Date**		**Environment**	aquatic, marine
**Order (Older Classification Scheme)**	Chroococcales	**Habitat Sample Description**	tide pool, rock surface scraping
**Order 2014 (Modern Taxonomy)**	Chroococcales**	**Ecophysiological traits**	
**Taxonomy Notes**	**inconsistent; assignment supported by phylogeny	**Notes on ecophysiological traits**	bartolosides production (unknown ecophysiological role)
**Accession Number(s)_16S**	HQ832911	**Isolator**	Vitor Ramos
**Accession Number(s)_others (product/gene /region)**		**Medium**	MN and Z8 25‰ TM sea salt, w/ vit. B12
**Collector**	Rui Seabra/Vitor Ramos	**Strain Status**	non-axenic, unicyanobacterial
**Collection Date**	2006/11/21	**Preservation type**	subculturing
**Location**	Portugal: Praia de São Bartolomeu do Mar, Esposende	**Light:dark cycle**	12:12-h/14:10-h
**Latitude & Longitude**	41.57377 N 8.798558 W	**Temperature (ºC)**	19/25
		**Light intensity**	10–30 µmol photons m^{-2} s^{-1}
**References Publicly Available Articles**	Brito et al. (2012) Syst. Appl. Microbiol. 35, 110–119; Costa et al. (2014) Mar. Drugs 12, 98-114; Leão et al. (2015) Angew. Chem. 54 (38), 11063-11067		
**References Publicly Available Theses**	Ribeiro M.J. (2012) MSc Dissertation, Department of Chemistry, University of Aveiro, Aveiro, Portugal; Freitas S. (2013) MSc Dissertation, ICBAS, University of Porto, Porto, Portugal; Macieira R. (2013) MSc Dissertation, FCUP, University of Porto, Porto, Portugal		

Strain ID	**Synechocystis sp. LEGE 06005**		
**Strain Taxonomy**	Synechocystis Sauvageau, C (1892)		
**Microphotograph 400×**	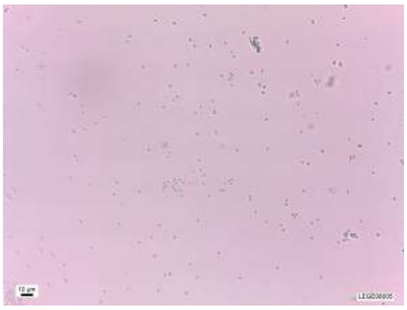	**Microphotograph 1000×**	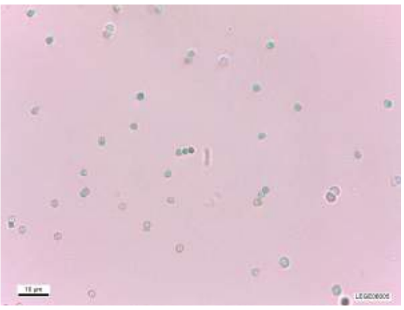
**Coidentity**	n.a.	**Morphometrics (Width × Length or Diameter) (µm)**	Ø 1,8±0,3
**Other Code(s)**	strain 6, LEAN 109	**Taxonomic notes/diacritical features**	
**Parent Sample**	Ref. 6	**Macroscopic growth features**	color: cyano; growing homogeneously
**Isolation Date**		**Environment**	aquatic, marine
**Order (Older Classification Scheme)**	Chroococcales	**Habitat Sample Description**	sea water sample, coastal, surf zone
**Order 2014 (Modern Taxonomy)**	Chroococcales**	**Ecophysiological traits**	
**Taxonomy Notes**	**inconsistent; assignment supported by phylogeny	**Notes on ecophysiological traits**	
**Accession Number(s)_16S**	KU951820	**Isolator**	Bárbara Frazão
**Accession Number(s)_others (product/gene /region)**		**Medium**	Z8 25‰ TM sea salt w/ vit. B12
**Collector**	Bárbara Frazão	**Strain Status**	non-axenic, unicyanobacterial
**Collection Date**	Fall 2006	**Preservation type**	subculturing
**Location**	Portugal: São Pedro de Moel	**Light:dark cycle**	12:12-h
**Latitude & Longitude**	39.75529 N 9.033400 W	**Temperature (ºC)**	19
		**Light intensity**	10–30 µmol photons m−2 s−1
**References Publicly Available Articles**	Frazão et al. (2010) Mar. Drugs 8, 1908-1919; Rastoll et al. (2013) J. Appl. Phycol. 25, 1483-1493; Costa et al. (2014) Mar. Drugs 12, 98-114		
**References Publicly Available Theses**	Frazão B. (2008) MSc Dissertation, FCUP, University of Porto, Porto, Portugal		

**Strain ID**	**Synechocystis sp. LEGE 06017**		
**Strain Taxonomy**	Synechocystis Sauvageau, C (1892)		
**Microphotograph 400×**	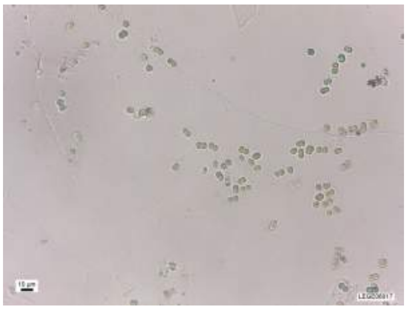	**Microphotograph 1000×**	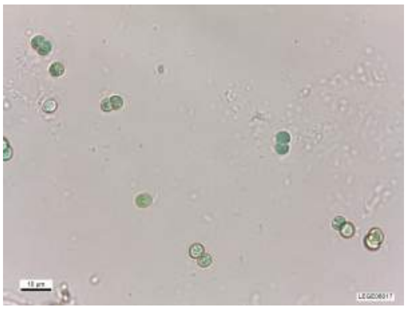
**Coidentity**	LEGE 06019	**Morphometrics (Width × Length or Diameter) (µm)**	Ø 4,5±0,5
**Other Code(s)**	strain 3, LEAN 101	**Taxonomic notes/diacritical features**	
**Parent Sample**	Ref. 3	**Macroscopic growth features**	color: cyano
**Isolation Date**		**Environment**	aquatic, marine
**Order (Older Classification Scheme)**	Chroococcales	**Habitat Sample Description**	sea water sample, coastal, surf zone
**Order 2014 (Modern Taxonomy)**	Chroococcales**	**Ecophysiological traits**	
**Taxonomy Notes**	**inconsistent; assignment supported by phylogeny	**Notes on ecophysiological traits**	
**Accession Number(s)_16S**		**Isolator**	Bárbara Frazão
**Accession Number(s)_others (product/gene /region)**		**Medium**	Z8 25‰ TM sea salt w/ vit. B12
**Collector**	Bárbara Frazão	**Strain Status**	non-axenic, unicyanobacterial
**Collection Date**	Fall 2006	**Preservation type**	subculturing
**Location**	Portugal: Praia dos Coxos, Sto. Isidoro	**Light:dark cycle**	12:12-h
**Latitude & Longitude**	39.00455 N 9.425842 W	**Temperature (ºC)**	19
		**Light intensity**	10–30 µmol photons m−2 s−1
**References Publicly Available Articles**			
**References Publicly Available Theses**	Frazão B. (2008) MSc Dissertation, FCUP, University of Porto, Porto, Portugal		

Strain ID	**Synechocystis sp. LEGE 06025**		
**Strain Taxonomy**	Synechocystis Sauvageau, C (1892)		
**Microphotograph 400×**	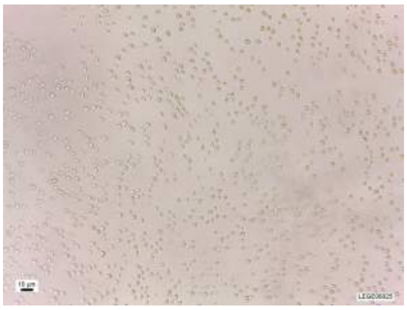	**Microphotograph 1000×**	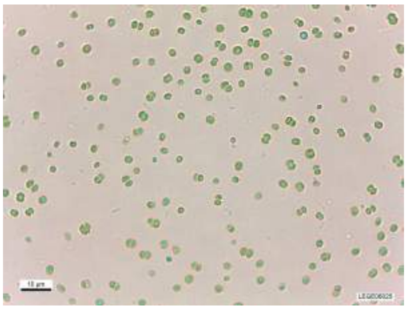
**Coidentity**	n.a.	**Morphometrics (Width × Length or Diameter) (µm)**	Ø 2,7±0,4
**Other Code(s)**	strain 5, LEAN 115	**Taxonomic notes/diacritical features**	
**Parent Sample**	Ref. 5, 1 Empa	**Macroscopic growth features**	color: cyano; growing homogeneously, but also forming aggregates
**Isolation Date**		**Environment**	aquatic, marine
**Order (Older Classification Scheme)**	Chroococcales	**Habitat Sample Description**	intertidal zone, wave-exposed rock
**Order 2014 (Modern Taxonomy)**	Chroococcales**	**Ecophysiological traits**	
**Taxonomy Notes**	**inconsistent; assignment supported by phylogeny	**Notes on ecophysiological traits**	
**Accession Number(s)_16S**	KU951821	**Isolator**	Bárbara Frazão
**Accession Number(s)_others (product/gene /region)**		**Medium**	Z8 25‰ TM sea salt w/ vit. B12
**Collector**	Bárbara Frazão	**Strain Status**	non-axenic, unicyanobacterial
**Collection Date**	Fall 2006	**Preservation type**	subculturing
**Location**	Portugal: Praia da Empa, Ericeira	**Light:dark cycle**	12:12-h
**Latitude & Longitude**	38.98079 N 9.421944 W	**Temperature (ºC)**	19
		**Light intensity**	10–30 µmol photons m−2 s−1
**References Publicly Available Articles**			
**References Publicly Available Theses**	Frazão B. (2008) MSc Dissertation, FCUP, University of Porto, Porto, Portugal		

**Strain ID**	**Synechocystis sp. LEGE 06079**		
**Strain Taxonomy**	Synechocystis Sauvageau, C (1892)		
**Microphotograph 400×**	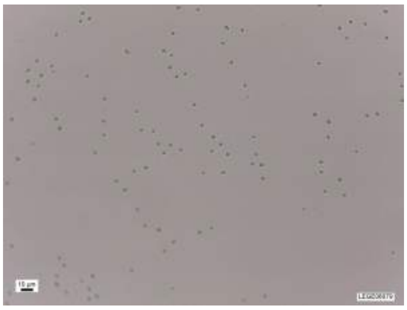	**Microphotograph 1000×**	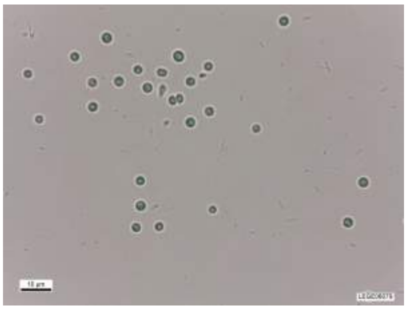
**Coidentity**	n.a.	**Morphometrics (Width × Length or Diameter) (µm)**	Ø 2,7±0,2
**Other Code(s)**		**Taxonomic notes/diacritical features**	
**Parent Sample**	VL 27	**Macroscopic growth features**	color: cyano; growing homogeneously; mucilaginous
**Isolation Date**		**Environment**	aquatic, brackish
**Order (Older Classification Scheme)**	Chroococcales	**Habitat Sample Description**	mesotidal zone, benthic
**Order 2014 (Modern Taxonomy)**	Chroococcales**	**Ecophysiological traits**	EPS production
**Taxonomy Notes**	**inconsistent; assignment supported by phylogeny	**Notes on ecophysiological traits**	dehydroabietic acid production (unknown ecophysiological production)
**Accession Number(s)_16S**	HM217076	**Isolator**	Viviana Lopes
**Accession Number(s)_others (product/gene /region)**		**Medium**	Z8
**Collector**	Viviana Lopes	**Strain Status**	non-axenic, unicyanobacterial
**Collection Date**	2006/10/27	**Preservation type**	subculturing, cryopreserved
**Location**	Portugal: Douro estuary, Vila Nova de Gaia	**Light:dark cycle**	12:12-h
**Latitude & Longitude**	41.13742 N 8.663667 W	**Temperature (ºC)**	19
		**Light intensity**	10–30 µmol photons m−2 s−1
**References Publicly Available Articles**	Lopes et al. (2010) Mar. Drugs 8, 471-482; Baptista et al. (2011) Toxicon 58, 410–414; Lopes et al. (2011) Toxicol. In Vitro 25, 944–950; Cianca et al. (2012) Amino Acids 42, 2473–2479; Lopes et al. (2012) Mar. Environ. Res. 73, 7–16; Almeida et al. (2015) Toxins 7(8), 2739-2756; Baptista et al. (2015) Environ. Sci. Pollut. Res., 22, 12501-12510; Costa et al. (2016) Sci. Rep. 6, 23436		
**References Publicly Available Theses**	Lopes V.R. (2011) PhD Thesis, FCUP, University of Porto, Porto, Portugal; Vasconcelos R. (2013) MSc Dissertation, ICBAS, University of Porto, Porto, Portugal;		

**Strain ID**	**Synechocystis sp. LEGE 06083**		
**Strain Taxonomy**	Synechocystis Sauvageau, C (1892)		
**Microphotograph 400×**	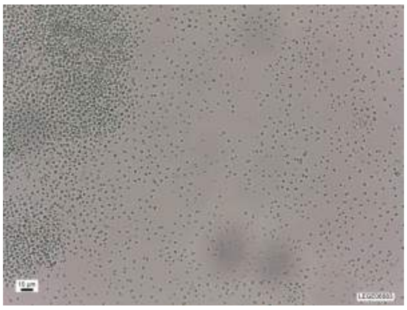	**Microphotograph 1000×**	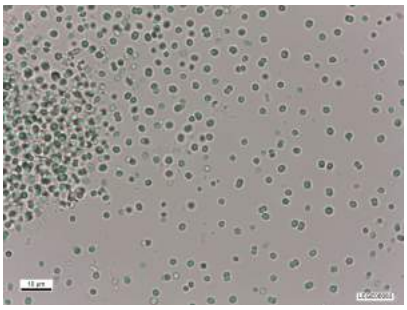
**Coidentity**	n.a.	**Morphometrics (Width × Length or Diameter) (µm)**	Ø 1,9±0,2
**Other Code(s)**		**Taxonomic notes/diacritical features**	distinguishable centro- and chromatoplasm
**Parent Sample**	VL PORTp131006	**Macroscopic growth features**	color: cyano; mucilaginous
**Isolation Date**		**Environment**	aquatic, brackish
**Order (Older Classification Scheme)**	Chroococcales	**Habitat Sample Description**	water sample, on the estuary shore
**Order 2014 (Modern Taxonomy)**	Chroococcales**	**Ecophysiological traits**	EPS production
**Taxonomy Notes**	**inconsistent; assignment supported by phylogeny	**Notes on ecophysiological traits**	
**Accession Number(s)_16S**	HM217084	**Isolator**	Viviana Lopes
**Accession Number(s)_others (product/gene /region)**		**Medium**	Z8 25‰ TM sea salt w/ vit. B12
**Collector**	Viviana Lopes	**Strain Status**	non-axenic, unicyanobacterial
**Collection Date**	2006/10/13	**Preservation type**	subculturing
**Location**	Portugal: Douro estuary, Porto	**Light:dark cycle**	12:12-h
**Latitude & Longitude**	41.14658 N 8.657972 W	**Temperature (ºC)**	19
		**Light intensity**	10–30 µmol photons m−2 s−1
**References Publicly Available Articles**	Lopes et al. (2010) Mar. Drugs 8, 471-482; Baptista et al. (2011) Toxicon 58, 410–414; Lopes et al. (2011) Toxicol. In Vitro 25, 944–950; Cianca et al. (2012) Amino Acids 42, 2473–2479; ; Lopes et al. (2012) Mar. Environ. Res. 73, 7–16; Almeida et al. (2015) Toxins 7(8), 2739-2756		
**References Publicly Available Theses**	Lopes V.R. (2011) PhD Thesis, FCUP, University of Porto, Porto, Portugal		

Strain ID	**Synechocystis sp. LEGE 07073**		
**Strain Taxonomy**	Synechocystis Sauvageau, C (1892)		
**Microphotograph 400×**	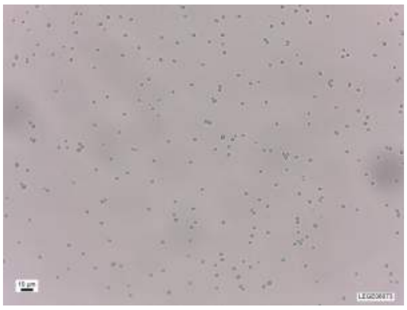	**Microphotograph 1000×**	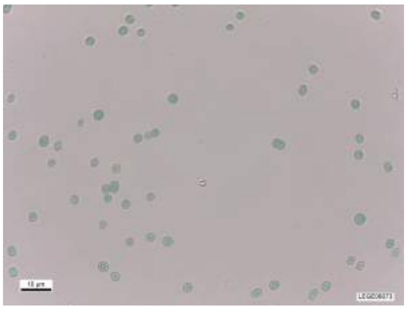
**Coidentity**	n.a.	**Morphometrics (Width × Length or Diameter) (µm)**	Ø 2,5±0,2
**Other Code(s)**		**Taxonomic notes/diacritical features**	
**Parent Sample**	VL JPp260307	**Macroscopic growth features**	color: cyano; growing homogeneously
**Isolation Date**		**Environment**	aquatic, brackish
**Order (Older Classification Scheme)**	Chroococcales	**Habitat Sample Description**	water sample, on the estuary shore
**Order 2014 (Modern Taxonomy)**	Chroococcales**	**Ecophysiological traits**	
**Taxonomy Notes**	**inconsistent; assignment supported by phylogeny	**Notes on ecophysiological traits**	
**Accession Number(s)_16S**	HM217083	**Isolator**	Viviana Lopes
**Accession Number(s)_others (product/gene /region)**		**Medium**	Z8
**Collector**	Viviana Lopes	**Strain Status**	non-axenic, unicyanobacterial
**Collection Date**	2007/03/26	**Preservation type**	subculturing
**Location**	Portugal: Vouga estuary, Ria de Aveiro, São Jacinto	**Light:dark cycle**	12:12-h
**Latitude & Longitude**	40.66644 N 8.725806 W	**Temperature (ºC)**	19
		**Light intensity**	10–30 µmol photons m−2 s−1
**References Publicly Available Articles**	Lopes et al. (2010) Mar. Drugs 8, 471-482; Baptista et al. (2011) Toxicon 58, 410–414; Lopes et al. (2011) Toxicol. In Vitro 25, 944–950; Cianca et al. (2012) Amino Acids 42, 2473–2479; ; Lopes et al. (2012) Mar. Environ. Res. 73, 7–16; Almeida et al. (2015) Toxins 7(8), 2739-2756		
**References Publicly Available Theses**	Lopes V.R. (2011) PhD Thesis, FCUP, University of Porto, Porto, Portugal		

**Strain ID**	**Synechocystis sp. LEGE 07211**		
**Strain Taxonomy**	Synechocystis Sauvageau, C (1892)		
**Microphotograph 400×**	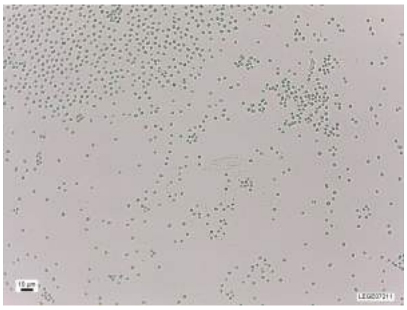	**Microphotograph 1000×**	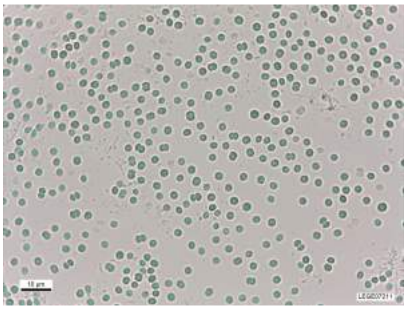
**Coidentity**	n.a.	**Morphometrics (Width × Length or Diameter) (µm)**	Ø 2,1±0,2
**Other Code(s)**	LEAN JM 47	**Taxonomic notes/diacritical features**	
**Parent Sample**	A1 AG - 6 PQN δ y	**Macroscopic growth features**	color: cyano; growing homogeneously, but also forming aggregates
**Isolation Date**		**Environment**	aquatic, freshwater
**Order (Older Classification Scheme)**	Chroococcales	**Habitat Sample Description**	biofilm, from a biological treatment tank outlet
**Order 2014 (Modern Taxonomy)**	Chroococcales**	**Ecophysiological traits**	
**Taxonomy Notes**	**inconsistent; assignment supported by phylogeny	**Notes on ecophysiological traits**	
**Accession Number(s)_16S**	KU951822	**Isolator**	Joana Martins
**Accession Number(s)_others (product/gene /region)**		**Medium**	Z8
**Collector**	Joana Martins	**Strain Status**	non-axenic, unicyanobacterial
**Collection Date**	Aug-2007	**Preservation type**	subculturing, cryopreserved
**Location**	Portugal: wastewater treatment plant at Febros river, Vila Nova de Gaia	**Light:dark cycle**	12:12-h
**Latitude & Longitude**	41.11812 N 8.570944 W	**Temperature (ºC)**	19
		**Light intensity**	10–30 µmol photons m−2 s−1
**References Publicly Available Articles**			
**References Publicly Available Theses**	Martins J. (2008) MSc Dissertation, FFUP, University of Porto, Porto, Portugal		

**Strain ID**	**Synechocystis sp. LEGE 07367**		
**Strain Taxonomy**	Synechocystis Sauvageau, C (1892)		
**Microphotograph 400×**	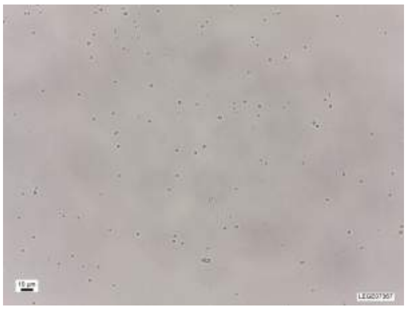	**Microphotograph 1000×**	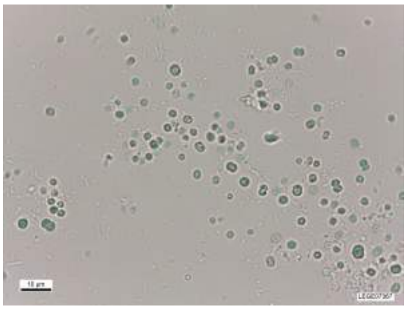
**Coidentity**	n.a.	**Morphometrics (Width × Length or Diameter) (µm)**	Ø 2,3±0,4
**Other Code(s)**		**Taxonomic notes/diacritical features**	
**Parent Sample**	VL JPp160107	**Macroscopic growth features**	color: cyano; growing homogeneously; mucilaginous
**Isolation Date**		**Environment**	aquatic, brackish
**Order (Older Classification Scheme)**	Chroococcales	**Habitat Sample Description**	water sample, near the shore
**Order 2014 (Modern Taxonomy)**	Chroococcales**	**Ecophysiological traits**	EPS production
**Taxonomy Notes**	**inconsistent; assignment supported by phylogeny	**Notes on ecophysiological traits**	
**Accession Number(s)_16S**	KU951823	**Isolator**	Viviana Lopes
**Accession Number(s)_others (product/gene /region)**		**Medium**	Z8
**Collector**	Viviana Lopes	**Strain Status**	non-axenic, unicyanobacterial
**Collection Date**	2007/01/16	**Preservation type**	subculturing
**Location**	Portugal: Vouga estuary	**Light:dark cycle**	12:12-h
**Latitude & Longitude**	unknown	**Temperature (ºC)**	19
		**Light intensity**	10–30 µmol photons m−2 s−1
**References Publicly Available Articles**			
**References Publicly Available Theses**			

**Strain ID**	**Tolypothrix sp. LEGE 11397**		
**Strain Taxonomy**	Tolypothrix Kützing ex Bornet, É & Flahault, C (1886)		
**Microphotograph 400×**	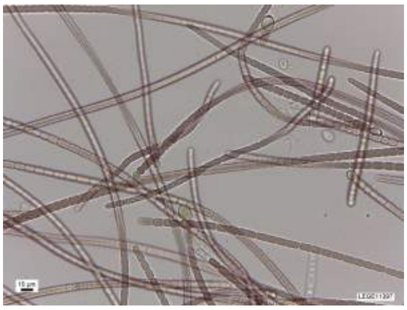	**Microphotograph 1000×**	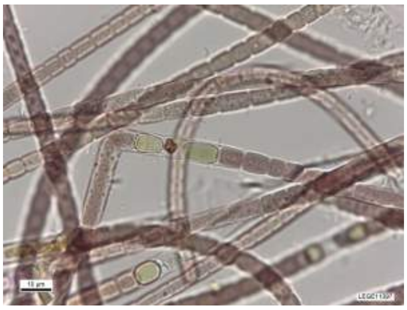
**Coidentity**	n.a.	**Morphometrics (Width × Length or Diameter) (µm)**	5,3±0,4 × 7,7±1,2 (Cells); Ø 6,0±1,3 (Terminal Heterocytes); 6,0±0,4 × 10,1±2,0 (Intercalar Heterocytes); 6,4±0,4 × 10,2±1,6 (Akinetes)
**Other Code(s)**		**Taxonomic notes/diacritical features**	terminal and intercalar heterocytes; hormogonia and necridic cells; facultative sheath
**Parent Sample**	Fi 1501	**Macroscopic growth features**	color: brownish; forming a firm biofilm
**Isolation Date**		**Environment**	aquatic, freshwater
**Order (Older Classification Scheme)**	Nostocales	**Habitat Sample Description**	in an aquarium with Azolla sp.
**Order 2014 (Modern Taxonomy)**	Nostocales	**Ecophysiological traits**	nitrogen-fixation, EPS production
**Taxonomy Notes**		**Notes on ecophysiological traits**	
**Accession Number(s)_16S**	KJ004418	**Isolator**	Ana L. Pereira
**Accession Number(s)_others (product/gene /region)**		**Medium**	BG11o
**Collector**	Ana L. Pereira	**Strain Status**	non-axenic, unicyanobacterial
**Collection Date**	Fall 2011	**Preservation type**	subculturing
**Location**	Portugal	**Light:dark cycle**	12:12-h
**Latitude & Longitude**	unknown	**Temperature (ºC)**	19
		**Light intensity**	10–30 µmol photons m−2 s−1
**References Publicly Available Articles**			
**References Publicly Available Theses**	Macieira R. (2013) MSc Dissertation, FCUP, University of Porto, Porto, Portugal		

**Strain ID**	**Toxifilum mysidocida LEGE 06108**		
**Strain Taxonomy**	Toxifilum mysidocida Zimba, PV; Huang, IS; Foley, JE & Linton, EW (2017)		
**Microphotograph 400×**	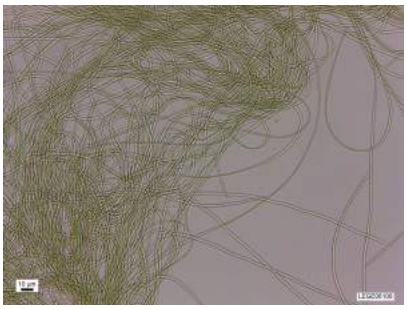	**Microphotograph 1000×**	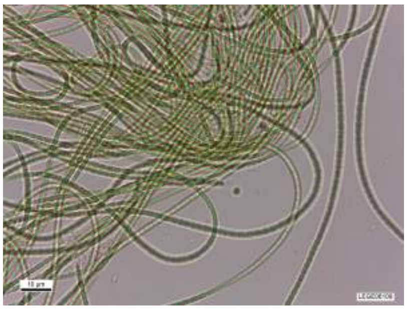
**Coidentity**	n.a.	**Morphometrics (Width × Length or Diameter) (µm)**	1,5±0,3 × 2,3±0,4
**Other Code(s)**	LEAN 013	**Taxonomic notes/diacritical features**	
**Parent Sample**	O.06 LUZ 10 - BAAA	**Macroscopic growth features**	color: cyano; growing homogeneously; mucilaginous
**Isolation Date**		**Environment**	aquatic, marine
**Order (Older Classification Scheme)**	Oscillatoriales	**Habitat Sample Description**	tide puddle, rock surface scraping
**Order 2014 (Modern Taxonomy)**	not determined	**Ecophysiological traits**	
**Taxonomy Notes**		**Notes on ecophysiological traits**	
**Accession Number(s)_16S**	HQ832942	**Isolator**	Vitor Ramos
**Accession Number(s)_others (product/gene /region)**	KC842357 (polyketide synthase)	**Medium**	Z8 25‰ TM sea salt w/ vit. B12
**Collector**	Rui Seabra	**Strain Status**	non-axenic, unicyanobacterial
**Collection Date**	2006/12/12	**Preservation type**	subculturing
**Location**	Portugal: Praia da Luz, Lagos	**Light:dark cycle**	12:12-h
**Latitude & Longitude**	37.08622 N 8.725375 W	**Temperature (ºC)**	19
		**Light intensity**	10–30 µmol photons m−2 s−1
**References Publicly Available Articles**	Brito et al. (2012) Syst. Appl. Microbiol. 35, 110–119; Costa et al. (2014) Mar. Drugs 12, 98-114; Brito et al. (2015) Algal Res. 9, 218-226; Zimba et al. (2016) J. Phycol. doi:10.1111/jpy.12490		
**References Publicly Available Theses**	Costa M.S. (2011) MSc Dissertation, ICBAS and FCUP, University of Porto, Porto, Portugal		

Strain ID	**Tychonema bornetii LEGE 14444**		
**Strain Taxonomy**	Tychonema bornetii (Zukal) Anagnostidis, K & Komárek, J (1988)		
**Microphotograph 400×**	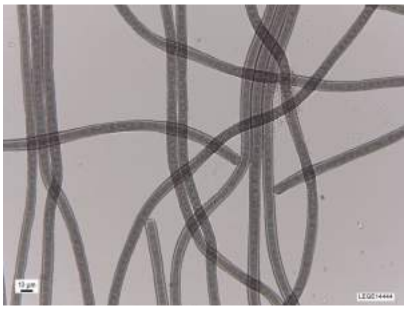	**Microphotograph 1000×**	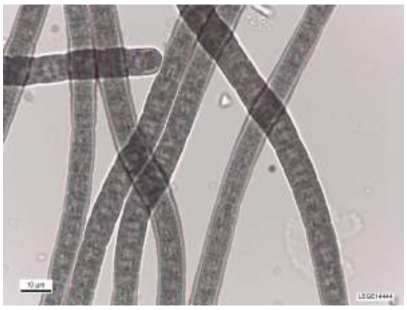
**Coidentity**	n.a.	**Morphometrics (Width × Length or Diameter) (µm)**	7,7±0,7 × 2,7±0,5
**Other Code(s)**		**Taxonomic notes/diacritical features**	straight to curved trichomes; with calyptra; hormogonia; with sheath
**Parent Sample**	Col.1 GAAA	**Macroscopic growth features**	color: dark olive green; forming a firm biofilm
**Isolation Date**		**Environment**	aquatic, freshwater
**Order (Older Classification Scheme)**	Oscillatoriales	**Habitat Sample Description**	water sample
**Order 2014 (Modern Taxonomy)**	Oscillatoriales	**Ecophysiological traits**	
**Taxonomy Notes**		**Notes on ecophysiological traits**	
**Accession Number(s)_16S**	KU951825	**Isolator**	Vitor Ramos
**Accession Number(s)_others (product/gene /region)**		**Medium**	Z8
**Collector**	Vitor Vasconcelos	**Strain Status**	non-axenic, unicyanobacterial
**Collection Date**	2014	**Preservation type**	subculturing
**Location**	Colombia	**Light:dark cycle**	12:12-h
**Latitude & Longitude**	unknown	**Temperature (ºC)**	19
		**Light intensity**	10–30 µmol photons m−2 s−1
**References Publicly Available Articles**			
**References Publicly Available Theses**			

**Strain ID**	**Tychonema sp. LEGE 06204**		
**Strain Taxonomy**	Tychonema Anagnostidis, K & Komárek, J (1988)		
**Microphotograph 400×**	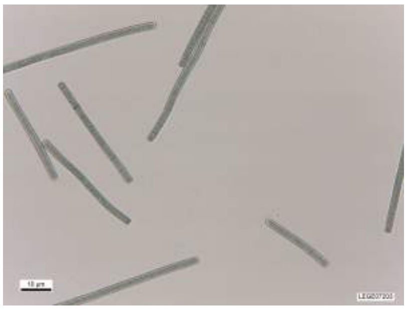	**Microphotograph 1000×**	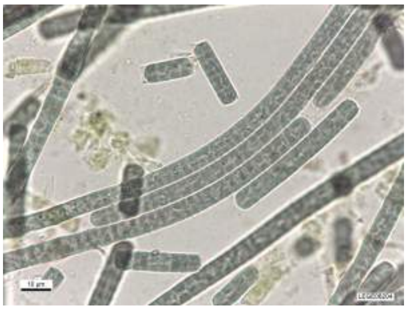
**Coidentity**	n.a.	**Morphometrics (Width × Length or Diameter) (µm)**	5,8±0,3 × 3,4±0,7
**Other Code(s)**		**Taxonomic notes/diacritical features**	trichomes sometimes slightly bent and narrowed toward ends; terminal cells rounded, blunted or capitate, often with a narrow calyptra; keritomized chromatoplasma; facultative sheath
**Parent Sample**	A1 24/10 - 1	**Macroscopic growth features**	color: olive green; forming a firm biofilm
**Isolation Date**		**Environment**	aquatic, freshwater
**Order (Older Classification Scheme)**	Oscillatoriales	**Habitat Sample Description**	biofilm, from a secondary decanter tank bank
**Order 2014 (Modern Taxonomy)**	Oscillatoriales	**Ecophysiological traits**	
**Taxonomy Notes**	Phormidium sp., group VII (Komárek & Anagnostidis, 2007)	**Notes on ecophysiological traits**	earth odour (geosmin?)
**Accession Number(s)_16S**	KC989699	**Isolator**	Joana Martins
**Accession Number(s)_others (product/gene /region)**	KF008256 (cyanobactin, N-terminal protease (A))	**Medium**	Z8
**Collector**	Joana Martins	**Strain Status**	non-axenic, unicyanobacterial
**Collection Date**	2006/10/27	**Preservation type**	subculturing
**Location**	Portugal: wastewater treatment plant at Febros river, Vila Nova de Gaia	**Light:dark cycle**	12:12-h
**Latitude & Longitude**	41.11812 N 8.570944 W	**Temperature (ºC)**	19
		**Light intensity**	10–30 µmol photons m−2 s−1
**References Publicly Available Articles**	Martins et al. (2013) Mar. Drugs 11 (12), 4902-4916		
**References Publicly Available Theses**			

**Strain ID**	**Tychonema sp. LEGE 06205**		
**Strain Taxonomy**	Tychonema Anagnostidis, K & Komárek, J (1988)		
**Microphotograph 400×**	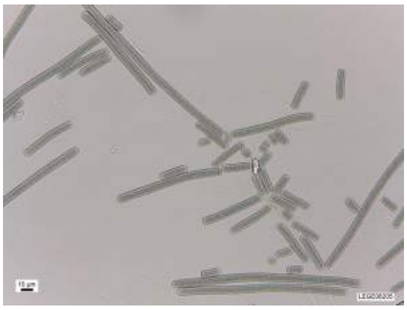	**Microphotograph 1000×**	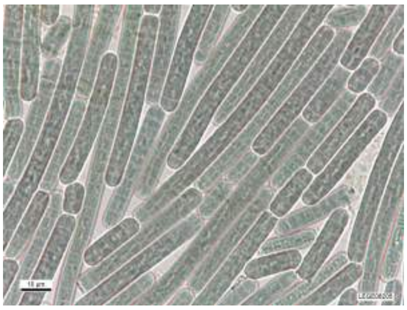
**Coidentity**	n.a.	**Morphometrics (Width × Length or Diameter) (µm)**	5,4±0,5 × 3,0±0,4
**Other Code(s)**		**Taxonomic notes/diacritical features**	trichomes sometimes slightly bent and narrowed toward ends; terminal cells rounded, blunted or capitate, often with a narrow calyptra; keritomized chromatoplasma; facultative sheath
**Parent Sample**	A1 24/10 - 7	**Macroscopic growth features**	color: olive green; forming a firm biofilm
**Isolation Date**		**Environment**	aquatic, freshwater
**Order (Older Classification Scheme)**	Oscillatoriales	**Habitat Sample Description**	biofilm, from a secondary decanter tank bank
**Order 2014 (Modern Taxonomy)**	Oscillatoriales	**Ecophysiological traits**	
**Taxonomy Notes**		**Notes on ecophysiological traits**	
**Accession Number(s)_16S**	KU951826	**Isolator**	Joana Martins
**Accession Number(s)_others (product/gene /region)**		**Medium**	Z8
**Collector**	Joana Martins	**Strain Status**	non-axenic, unicyanobacterial
**Collection Date**	2006/10/27	**Preservation type**	subculturing
**Location**	Portugal: wastewater treatment plant at Febros river, Vila Nova de Gaia	**Light:dark cycle**	12:12-h
**Latitude & Longitude**	41.11812 N 8.570944 W	**Temperature (ºC)**	19
**References Publicly Available Articles**		**Light intensity**	10–30 µmol photons m−2 s−1
**References Publicly Available Theses**			

Strain ID	**Tychonema sp. LEGE 06206**		
**Strain Taxonomy**	Tychonema Anagnostidis, K & Komárek, J (1988)		
**Microphotograph 400×**	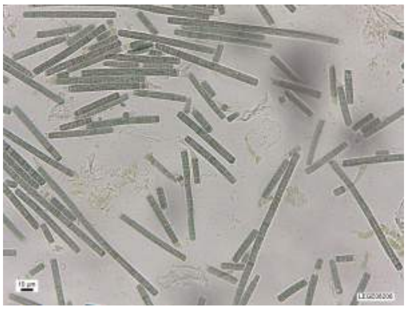	**Microphotograph 1000×**	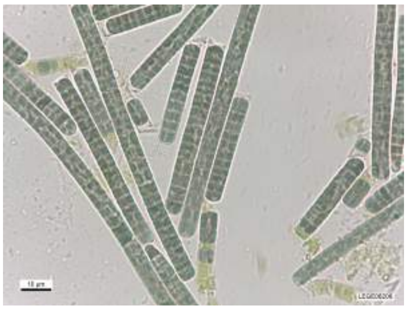
**Coidentity**	n.a.	**Morphometrics (Width × Length or Diameter) (µm)**	5,2±0,5 × 2,9±0,5
**Other Code(s)**		**Taxonomic notes/diacritical features**	trichomes sometimes slightly bent and narrowed toward ends; terminal cells rounded, blunted or capitate, often with a narrow calyptra; keritomized chromatoplasma; facultative sheath
**Parent Sample**	A1 27/11 - 3	**Macroscopic growth features**	color: olive green; forming a firm biofilm
**Isolation Date**		**Environment**	aquatic, freshwater
**Order (Older Classification Scheme)**	Oscillatoriales	**Habitat Sample Description**	biofilm, from a secondary decanter tank bank
**Order 2014 (Modern Taxonomy)**	Oscillatoriales	**Ecophysiological traits**	
**Taxonomy Notes**		**Notes on ecophysiological traits**	
**Accession Number(s)_16S**	KU951827	**Isolator**	Joana Martins
**Accession Number(s)_others (product/gene /region)**		**Medium**	Z8
**Collector**	Joana Martins	**Strain Status**	non-axenic, unicyanobacterial
**Collection Date**	27-Nov-2006	**Preservation type**	subculturing
**Location**	Portugal: wastewater treatment plant at Febros river, Vila Nova de Gaia	**Light:dark cycle**	12:12-h
**Latitude & Longitude**	41.11812 N 8.570944 W	**Temperature (ºC)**	19
**References Publicly Available Articles**		**Light intensity**	10–30 µmol photons m−2 s−1
**References Publicly Available Theses**			

**Strain ID**	**Tychonema sp. LEGE 06207**		
**Strain Taxonomy**	Tychonema Anagnostidis, K & Komárek, J (1988)		
**Microphotograph 400×**	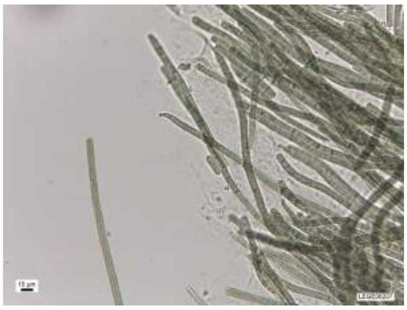	**Microphotograph 1000×**	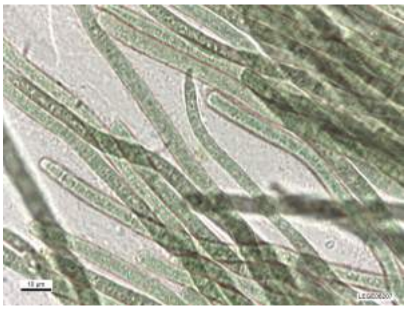
**Coidentity**	n.a.	**Morphometrics (Width × Length or Diameter) (µm)**	4,6±0,6 × 3,2±0,5
**Other Code(s)**		**Taxonomic notes/diacritical features**	trichomes sometimes slightly bent and narrowed toward ends; terminal cells rounded, blunted or capitate, often with a narrow calyptra; keritomized chromatoplasma; facultative sheath
**Parent Sample**	A1 29/12 - 1	**Macroscopic growth features**	color: olive green; forming a firm biofilm
**Isolation Date**		**Environment**	aquatic, freshwater
**Order (Older Classification Scheme)**	Oscillatoriales	**Habitat Sample Description**	biofilm, from a secondary decanter tank bank
**Order 2014 (Modern Taxonomy)**	Oscillatoriales	**Ecophysiological traits**	
**Taxonomy Notes**		**Notes on ecophysiological traits**	
**Accession Number(s)_16S**	KU951828	**Isolator**	Joana Martins
**Accession Number(s)_others (product/gene /region)**		**Medium**	Z8
**Collector**	Joana Martins	**Strain Status**	non-axenic, unicyanobacterial
**Collection Date**	29-Dec-2006	**Preservation type**	subculturing
**Location**	Portugal: wastewater treatment plant at Febros river, Vila Nova de Gaia	**Light:dark cycle**	12:12-h
**Latitude & Longitude**	41.11812 N 8.570944 W	**Temperature (ºC)**	19
		**Light intensity**	10–30 µmol photons m−2 s−1
**References Publicly Available Articles**			
**References Publicly Available Theses**			

**Strain ID**	**Tychonema sp. LEGE 06208**		
**Strain Taxonomy**	Tychonema Anagnostidis, K & Komárek, J (1988)		
**Microphotograph 400×**	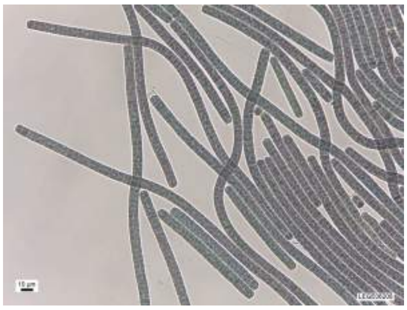	**Microphotograph 1000×**	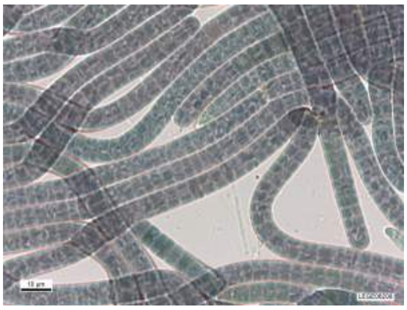
**Coidentity**	n.a.	**Morphometrics (Width × Length or Diameter) (µm)**	6,8±0,6 × 3,4±0,6
**Other Code(s)**		**Taxonomic notes/diacritical features**	trichomes sometimes slightly bent and narrowed toward ends; terminal cells rounded, blunted or capitate, often with a narrow calyptra; keritomized chromatoplasma; facultative sheath
**Parent Sample**	A1 29/12 - 5	**Macroscopic growth features**	color: olive green; forming a firm biofilm
**Isolation Date**		**Environment**	aquatic, freshwater
**Order (Older Classification Scheme)**	Oscillatoriales	**Habitat Sample Description**	biofilm, from a secondary decanter tank bank
**Order 2014 (Modern Taxonomy)**	Oscillatoriales	**Ecophysiological traits**	
**Taxonomy Notes**		**Notes on ecophysiological traits**	
**Accession Number(s)_16S**	KU951829	**Isolator**	Joana Martins
**Accession Number(s)_others (product/gene /region)**		**Medium**	Z8
**Collector**	Joana Martins	**Strain Status**	non-axenic, unicyanobacterial
**Collection Date**	29-Dec-2006	**Preservation type**	subculturing
**Location**	Portugal: wastewater treatment plant at Febros river, Vila Nova de Gaia	**Light:dark cycle**	12:12-h
**Latitude & Longitude**	41.11812 N 8.570944 W	**Temperature (ºC)**	19
		**Light intensity**	10–30 µmol photons m−2 s−1
**References Publicly Available Articles**			
**References Publicly Available Theses**			

Strain ID	**Tychonema sp. LEGE 06220**		
**Strain Taxonomy**	Tychonema Anagnostidis, K & Komárek, J (1988)		
**Microphotograph 400×**	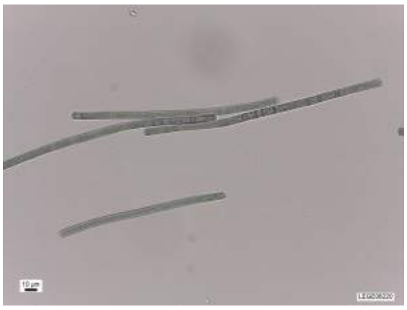	**Microphotograph 1000×**	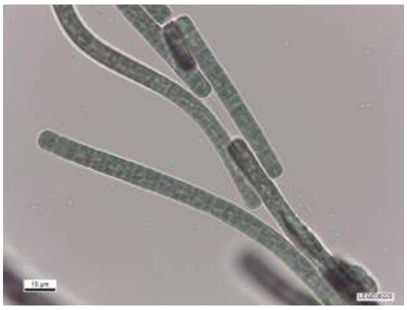
**Coidentity**	n.a.	**Morphometrics (Width × Length or Diameter) (µm)**	5,8±0,6 × 3,8±0,6
**Other Code(s)**		**Taxonomic notes/diacritical features**	trichomes sometimes slightly bent and narrowed toward ends; terminal cells rounded, blunted or capitate, often with a narrow calyptra; keritomized chromatoplasma; facultative sheath
**Parent Sample**	A1 OUT - 2 16i	**Macroscopic growth features**	color: olive green; forming a firm biofilm
**Isolation Date**		**Environment**	aquatic, freshwater
**Order (Older Classification Scheme)**	Oscillatoriales	**Habitat Sample Description**	biofilm, from a secondary decanter tank bank
**Order 2014 (Modern Taxonomy)**	Oscillatoriales	**Ecophysiological traits**	
**Taxonomy Notes**		**Notes on ecophysiological traits**	
**Accession Number(s)_16S**	KU951830	**Isolator**	Joana Martins
**Accession Number(s)_others (product/gene /region)**		**Medium**	Z8
**Collector**	Joana Martins	**Strain Status**	non-axenic, unicyanobacterial
**Collection Date**	Oct-2006	**Preservation type**	subculturing
**Location**	Portugal: wastewater treatment plant at Febros river, Vila Nova de Gaia	**Light:dark cycle**	12:12-h
**Latitude & Longitude**	41.11812 N 8.570944 W	**Temperature (ºC)**	19
		**Light intensity**	10–30 µmol photons m−2 s−1
**References Publicly Available Articles**			
**References Publicly Available Theses**			

Strain ID	**Tychonema sp. LEGE 06363**		
**Strain Taxonomy**	Tychonema Anagnostidis, K & Komárek, J (1988)		
**Microphotograph 400×**	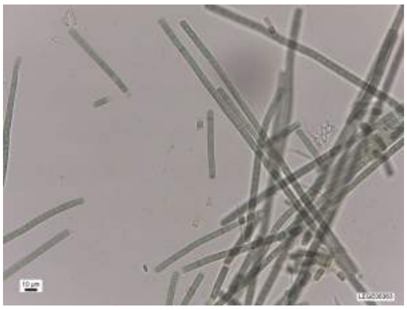	**Microphotograph 1000×**	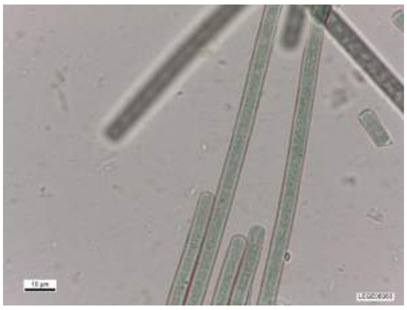
**Coidentity**	n.a.	**Morphometrics (Width × Length or Diameter) (µm)**	5,0±0,5 × 3,0±0,5
**Other Code(s)**		**Taxonomic notes/diacritical features**	necridic cells; with sheath
**Parent Sample**		**Macroscopic growth features**	color: cyano; forming a firm biofilm
**Isolation Date**		**Environment**	aquatic, freshwater
**Order (Older Classification Scheme)**	Oscillatoriales	**Habitat Sample Description**	biofilm, from a biological treatment tank outlet
**Order 2014 (Modern Taxonomy)**	Oscillatoriales	**Ecophysiological traits**	
**Taxonomy Notes**		**Notes on ecophysiological traits**	earth odour (geosmin?)
**Accession Number(s)_16S**	KC989700	**Isolator**	Joana Martins
**Accession Number(s)_others (product/gene /region)**	KF008257 (cyanobactin, N-terminal protease (A))	**Medium**	Z8
**Collector**	Joana Martins	**Strain Status**	non-axenic, unicyanobacterial
**Collection Date**	Oct-2006	**Preservation type**	subculturing
**Location**	Portugal: wastewater treatment plant at Febros river, Vila Nova de Gaia	**Light:dark cycle**	12:12-h
**Latitude & Longitude**	41.11812 N 8.570944 W	**Temperature (ºC)**	19
		**Light intensity**	10–30 µmol photons m−2 s−1
**References Publicly Available Articles**	Martins et al. (2013) Mar. Drugs 11 (12), 4902-4916; Costa et al. (2016) Sci. Rep. 6, 23436		
**References Publicly Available Theses**			

Strain ID	**Tychonema sp. LEGE 07196**		
**Strain Taxonomy**	Tychonema Anagnostidis, K & Komárek, J (1988)		
**Microphotograph 400×**	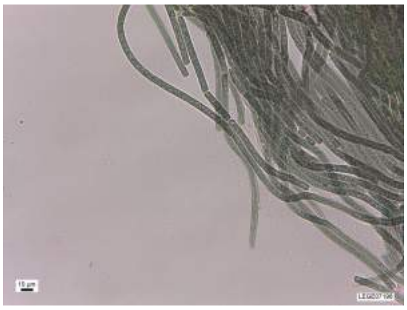	**Microphotograph 1000×**	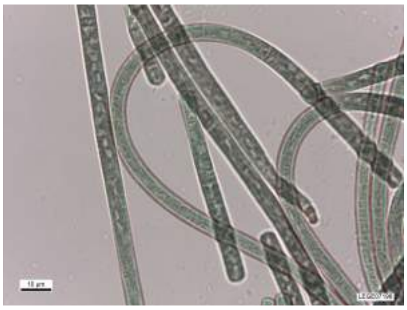
**Coidentity**	n.a.	**Morphometrics (Width × Length or Diameter) (µm)**	5,0±0,5 × 3,9±0,5
**Other Code(s)**		**Taxonomic notes/diacritical features**	trichomes sometimes slightly bent and narrowed toward ends; terminal cells rounded, blunted or capitate, often with a narrow calyptra; keritomized chromatoplasma; facultative sheath
**Parent Sample**	A1 01/07 - 1	**Macroscopic growth features**	color: olive green; forming a firm biofilm
**Isolation Date**		**Environment**	aquatic, freshwater
**Order (Older Classification Scheme)**	Oscillatoriales	**Habitat Sample Description**	biofilm, from a biological treatment tank outlet
**Order 2014 (Modern Taxonomy)**	Oscillatoriales	**Ecophysiological traits**	
**Taxonomy Notes**		**Notes on ecophysiological traits**	
**Accession Number(s)_16S**	KU951831	**Isolator**	Joana Martins
**Accession Number(s)_others (product/gene /region)**		**Medium**	Z8
**Collector**	Joana Martins	**Strain Status**	non-axenic, unicyanobacterial
**Collection Date**	Jan-2007	**Preservation type**	subculturing
**Location**	Portugal: wastewater treatment plant at Febros river, Vila Nova de Gaia	**Light:dark cycle**	12:12-h
**Latitude & Longitude**	41.11812 N 8.570944 W	**Temperature (ºC)**	19
		**Light intensity**	10–30 µmol photons m−2 s−1
**References Publicly Available Articles**			
**References Publicly Available Theses**			

Strain ID	**Tychonema sp. LEGE 07197**		
**Strain Taxonomy**	Tychonema Anagnostidis, K & Komárek, J (1988)		
**Microphotograph 400×**	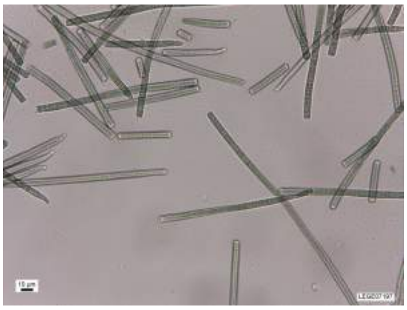	**Microphotograph 1000×**	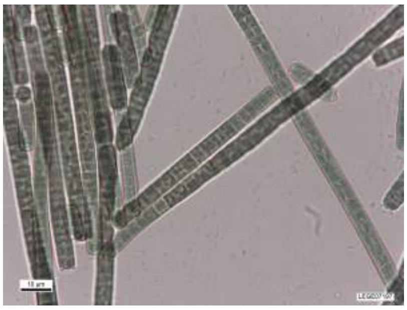
**Coidentity**	n.a.	**Morphometrics (Width × Length or Diameter) (µm)**	5,5±0,1 × 3,6±0,5
**Other Code(s)**		**Taxonomic notes/diacritical features**	trichomes sometimes slightly bent and narrowed toward ends; terminal cells rounded, blunted or capitate, often with a narrow calyptra; keritomized chromatoplasma; facultative sheath
**Parent Sample**	A1 01/07 - 5	**Macroscopic growth features**	color: olive green; forming a firm biofilm
**Isolation Date**		**Environment**	aquatic, freshwater
**Order (Older Classification Scheme)**	Oscillatoriales	**Habitat Sample Description**	biofilm, from a biological treatment tank outlet
**Order 2014 (Modern Taxonomy)**	Oscillatoriales	**Ecophysiological traits**	
**Taxonomy Notes**		**Notes on ecophysiological traits**	
**Accession Number(s)_16S**	KU951832	**Isolator**	Joana Martins
**Accession Number(s)_others (product/gene /region)**		**Medium**	Z8
**Collector**	Joana Martins	**Strain Status**	non-axenic, unicyanobacterial
**Collection Date**	Jan-2007	**Preservation type**	subculturing
**Location**	Portugal: wastewater treatment plant at Febros river, Vila Nova de Gaia	**Light:dark cycle**	12:12-h
**Latitude & Longitude**	41.11812 N 8.570944 W	**Temperature (ºC)**	19
		**Light intensity**	10–30 µmol photons m−2 s−1
**References Publicly Available Articles**			
**References Publicly Available Theses**			

Strain ID	**Tychonema sp. LEGE 07198**		
**Strain Taxonomy**	Tychonema Anagnostidis, K & Komárek, J (1988)		
**Microphotograph 400×**	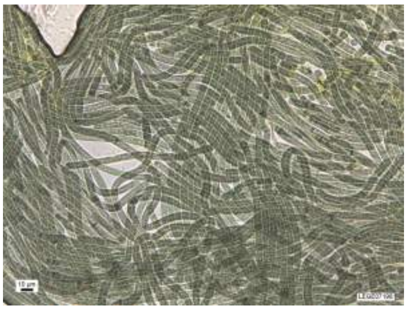	**Microphotograph 1000×**	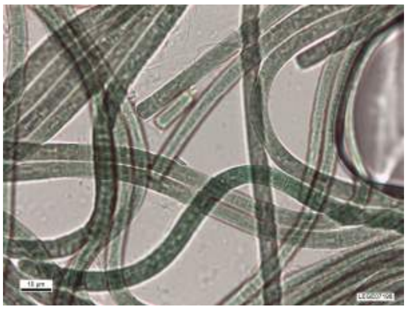
**Coidentity**	n.a.	**Morphometrics (Width × Length or Diameter) (µm)**	5,2±0,4 × 2,6±0,6
**Other Code(s)**		**Taxonomic notes/diacritical features**	trichomes sometimes slightly bent and narrowed toward ends; terminal cells rounded, blunted or capitate, often with a narrow calyptra; keritomized chromatoplasma; facultative sheath
**Parent Sample**	A1 01/07 - 8	**Macroscopic growth features**	color: olive green; forming a firm biofilm
**Isolation Date**		**Environment**	aquatic, freshwater
**Order (Older Classification Scheme)**	Oscillatoriales	**Habitat Sample Description**	biofilm, from a biological treatment tank outlet
**Order 2014 (Modern Taxonomy)**	Oscillatoriales	**Ecophysiological traits**	
**Taxonomy Notes**		**Notes on ecophysiological traits**	
**Accession Number(s)_16S**	KU951833	**Isolator**	Joana Martins
**Accession Number(s)_others (product/gene /region)**		**Medium**	Z8
**Collector**	Joana Martins	**Strain Status**	non-axenic, unicyanobacterial
**Collection Date**	Jan-2007	**Preservation type**	subculturing
**Location**	Portugal: wastewater treatment plant at Febros river, Vila Nova de Gaia	**Light:dark cycle**	12:12-h
**Latitude & Longitude**	41.11812 N 8.570944 W	**Temperature (ºC)**	19
		**Light intensity**	10–30 µmol photons m−2 s−1
**References Publicly Available Articles**			
**References Publicly Available Theses**			

**Strain ID**	**Tychonema sp. LEGE 07199**		
**Strain Taxonomy**	Tychonema Anagnostidis, K & Komárek, J (1988)		
**Microphotograph 400×**	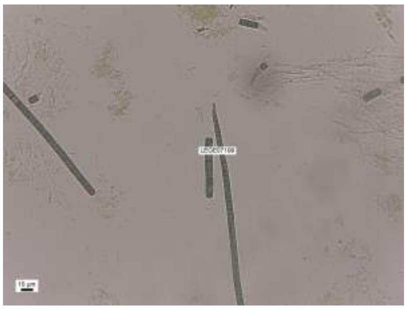	**Microphotograph 1000×**	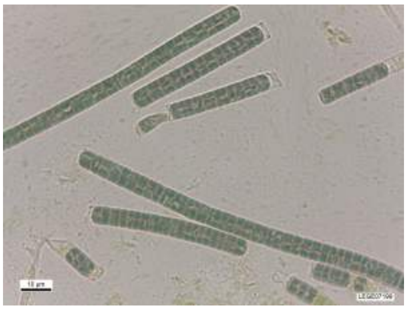
**Coidentity**	n.a.	**Morphometrics (Width × Length or Diameter) (µm)**	5,8±0,6 × 2,7±0,5
**Other Code(s)**		**Taxonomic notes/diacritical features**	trichomes sometimes slightly bent and narrowed toward ends; terminal cells rounded, blunted or capitate, often with a narrow calyptra; keritomized chromatoplasma; facultative sheath
**Parent Sample**	A1 01/07 - 12	**Macroscopic growth features**	color: olive green; forming a firm biofilm
**Isolation Date**		**Environment**	aquatic, freshwater
**Order (Older Classification Scheme)**	Oscillatoriales	**Habitat Sample Description**	biofilm, from a biological treatment tank outlet
**Order 2014 (Modern Taxonomy)**	Oscillatoriales	**Ecophysiological traits**	
**Taxonomy Notes**		**Notes on ecophysiological traits**	
**Accession Number(s)_16S**	KU951834	**Isolator**	Joana Martins
**Accession Number(s)_others (product/gene /region)**		**Medium**	Z8
**Collector**	Joana Martins	**Strain Status**	non-axenic, unicyanobacterial
**Collection Date**	Jan-2007	**Preservation type**	subculturing
**Location**	Portugal: wastewater treatment plant at Febros river, Vila Nova de Gaia	**Light:dark cycle**	12:12-h
**Latitude & Longitude**	41.11812 N 8.570944 W	**Temperature (ºC)**	19
		**Light intensity**	10–30 µmol photons m−2 s−1
**References Publicly Available Articles**			
**References Publicly Available Theses**			

Strain ID	**Tychonema sp. LEGE 07200**		
**Strain Taxonomy**	Tychonema Anagnostidis, K & Komárek, J (1988)		
**Microphotograph 400×**	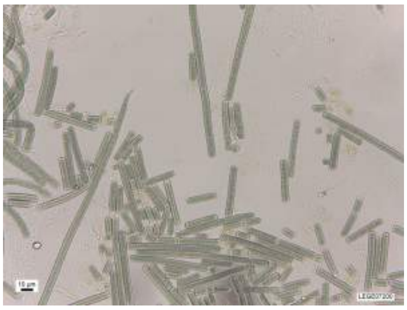	**Microphotograph 1000×**	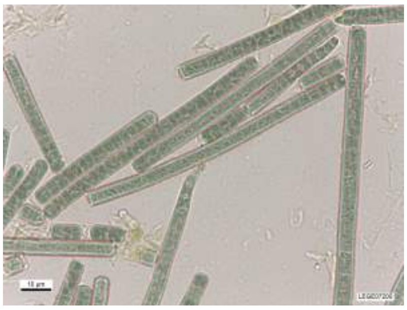
**Coidentity**	n.a.	**Morphometrics (Width × Length or Diameter) (µm)**	4,9±0,4 × 2,9±0,5
**Other Code(s)**		**Taxonomic notes/diacritical features**	trichomes sometimes slightly bent and narrowed toward ends; terminal cells rounded, blunted or capitate, often with a narrow calyptra; keritomized chromatoplasma; facultative sheath
**Parent Sample**	A1 01/07 - 13	**Macroscopic growth features**	color: olive green; forming a firm biofilm
**Isolation Date**		**Environment**	aquatic, freshwater
**Order (Older Classification Scheme)**	Oscillatoriales	**Habitat Sample Description**	biofilm, from a biological treatment tank outlet
**Order 2014 (Modern Taxonomy)**	Oscillatoriales	**Ecophysiological traits**	
**Taxonomy Notes**		**Notes on ecophysiological traits**	
**Accession Number(s)_16S**	KU951835	**Isolator**	Joana Martins
**Accession Number(s)_others (product/gene /region)**		**Medium**	Z8
**Collector**	Joana Martins	**Strain Status**	non-axenic, unicyanobacterial
**Collection Date**	Jan-2007	**Preservation type**	subculturing
**Location**	Portugal: wastewater treatment plant at Febros river, Vila Nova de Gaia	**Light:dark cycle**	12:12-h
**Latitude & Longitude**	41.11812 N 8.570944 W	**Temperature (ºC)**	19
		**Light intensity**	10–30 µmol photons m−2 s−1
**References Publicly Available Articles**			
**References Publicly Available Theses**			

Strain ID	**Tychonema sp. LEGE 07202**		
**Strain Taxonomy**	Tychonema Anagnostidis, K & Komárek, J (1988)		
**Microphotograph 400×**	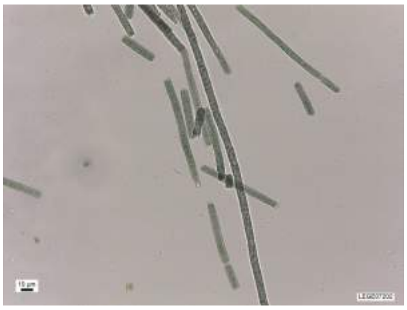	**Microphotograph 1000×**	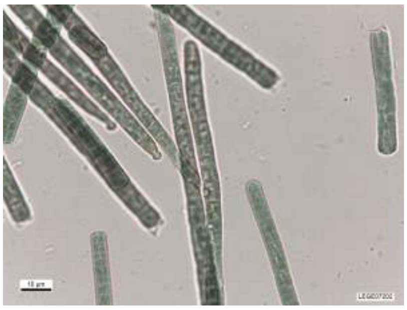
**Coidentity**	n.a.	**Morphometrics (Width × Length or Diameter) (µm)**	5,4±0,6 × 3,4±0,9
**Other Code(s)**		**Taxonomic notes/diacritical features**	trichomes sometimes slightly bent and narrowed toward ends; terminal cells rounded, blunted or capitate, often with a narrow calyptra; keritomized chromatoplasma; facultative sheath
**Parent Sample**	A1 02/07 - 6	**Macroscopic growth features**	color: olive green; forming a firm biofilm
**Isolation Date**		**Environment**	aquatic, freshwater
**Order (Older Classification Scheme)**	Oscillatoriales	**Habitat Sample Description**	biofilm, from a biological treatment tank outlet
**Order 2014 (Modern Taxonomy)**	Oscillatoriales	**Ecophysiological traits**	
**Taxonomy Notes**		**Notes on ecophysiological traits**	
**Accession Number(s)_16S**	KU951836	**Isolator**	Joana Martins
**Accession Number(s)_others (product/gene /region)**		**Medium**	Z8
**Collector**	Joana Martins	**Strain Status**	non-axenic, unicyanobacterial
**Collection Date**	Feb-2007	**Preservation type**	subculturing
**Location**	Portugal: wastewater treatment plant at Febros river, Vila Nova de Gaia	**Light:dark cycle**	12:12-h
**Latitude & Longitude**	41.11812 N 8.570944 W	**Temperature (ºC)**	19
		**Light intensity**	10–30 µmol photons m−2 s−1
**References Publicly Available Articles**			
**References Publicly Available Theses**			

Strain ID	**Tychonema sp. LEGE 07203**		
**Strain Taxonomy**	Tychonema Anagnostidis, K & Komárek, J (1988)		
**Microphotograph 400×**	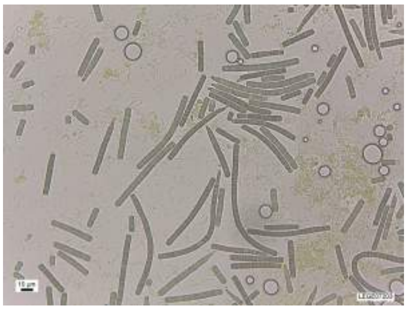	**Microphotograph 1000×**	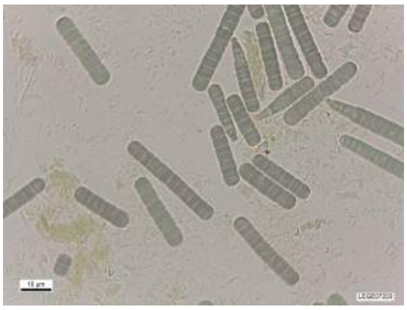
**Coidentity**	n.a.	**Morphometrics (Width × Length or Diameter) (µm)**	4,5±0,5 × 2,4±0,6
**Other Code(s)**		**Taxonomic notes/diacritical features**	trichomes sometimes slightly bent and narrowed toward ends; terminal cells rounded, blunted or capitate, often with a narrow calyptra; keritomized chromatoplasma; facultative sheath
**Parent Sample**	A1 04/07 - 6	**Macroscopic growth features**	color: olive green; forming a firm biofilm
**Isolation Date**		**Environment**	aquatic, freshwater
**Order (Older Classification Scheme)**	Oscillatoriales	**Habitat Sample Description**	biofilm, from a biological treatment tank outlet
**Order 2014 (Modern Taxonomy)**	Oscillatoriales	**Ecophysiological traits**	
**Taxonomy Notes**		**Notes on ecophysiological traits**	
**Accession Number(s)_16S**	KU951837	**Isolator**	Joana Martins
**Accession Number(s)_others (product/gene /region)**		**Medium**	Z8
**Collector**	Joana Martins	**Strain Status**	non-axenic, unicyanobacterial
**Collection Date**	Apr-2007	**Preservation type**	subculturing
**Location**	Portugal: wastewater treatment plant at Febros river, Vila Nova de Gaia	**Light:dark cycle**	12:12-h
**Latitude & Longitude**	41.11812 N 8.570944 W	**Temperature (ºC)**	19
		**Light intensity**	10–30 µmol photons m−2 s−1
**References Publicly Available Articles**			
**References Publicly Available Theses**			

Strain ID	**Tychonema sp. LEGE 07213**		
**Strain Taxonomy**	Tychonema Anagnostidis, K & Komárek, J (1988)		
**Microphotograph 400×**	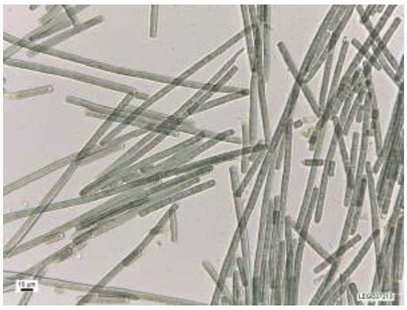	**Microphotograph 1000×**	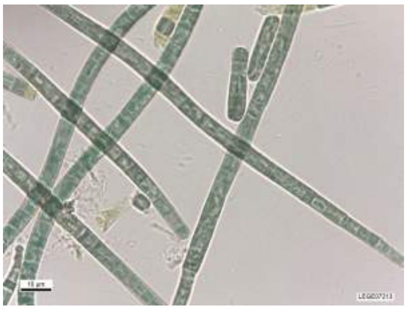
**Coidentity**	n.a.	**Morphometrics (Width × Length or Diameter) (µm)**	4,5±0,4 × 3,9±0,6
**Other Code(s)**		**Taxonomic notes/diacritical features**	trichomes sometimes slightly bent and narrowed toward ends; terminal cells rounded, blunted or capitate, often with a narrow calyptra; keritomized chromatoplasma; facultative sheath
**Parent Sample**	A1 JAN - 6 19i	**Macroscopic growth features**	color: olive green; forming a firm biofilm
**Isolation Date**		**Environment**	aquatic, freshwater
**Order (Older Classification Scheme)**	Oscillatoriales	**Habitat Sample Description**	biofilm, from a biological treatment tank outlet
**Order 2014 (Modern Taxonomy)**	Oscillatoriales	**Ecophysiological traits**	
**Taxonomy Notes**		**Notes on ecophysiological traits**	
**Accession Number(s)_16S**	KU951838	**Isolator**	Joana Martins
**Accession Number(s)_others (product/gene /region)**		**Medium**	Z8
**Collector**	Joana Martins	**Strain Status**	non-axenic, unicyanobacterial
**Collection Date**	Jan-2007	**Preservation type**	subculturing
**Location**	Portugal: wastewater treatment plant at Febros river, Vila Nova de Gaia	**Light:dark cycle**	12:12-h
**Latitude & Longitude**	41.11812 N 8.570944 W	**Temperature (ºC)**	19
**References Publicly Available Articles**		**Light intensity**	10–30 µmol photons m−2 s−1
**References Publicly Available Theses**			

**Strain ID**	**Tychonema sp. LEGE 07214**		
**Strain Taxonomy**	Tychonema Anagnostidis, K & Komárek, J (1988)		
**Microphotograph 400×**	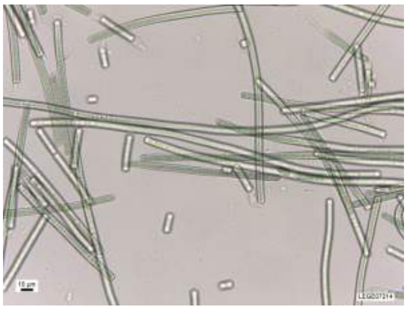	**Microphotograph 1000×**	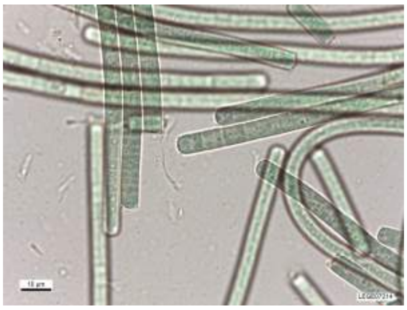
**Coidentity**	n.a.	**Morphometrics (Width × Length or Diameter) (µm)**	5,9±0,4 × 3,9±0,7
**Other Code(s)**		**Taxonomic notes/diacritical features**	trichomes sometimes slightly bent and narrowed toward ends; terminal cells rounded, blunted or capitate, often with a narrow calyptra; keritomized chromatoplasma; facultative sheath
**Parent Sample**	A1 JAN - 17 10i	**Macroscopic growth features**	color: olive green; forming a firm biofilm
**Isolation Date**		**Environment**	aquatic, freshwater
**Order (Older Classification Scheme)**	Oscillatoriales	**Habitat Sample Description**	biofilm, from a biological treatment tank outlet
**Order 2014 (Modern Taxonomy)**	Oscillatoriales	**Ecophysiological traits**	
**Taxonomy Notes**		**Notes on ecophysiological traits**	earth odour (geosmin?)
**Accession Number(s)_16S**	KU951839	**Isolator**	Joana Martins
**Accession Number(s)_others (product/gene /region)**		**Medium**	Z8
**Collector**	Joana Martins	**Strain Status**	non-axenic, unicyanobacterial
**Collection Date**	Jan-2007	**Preservation type**	subculturing
**Location**	Portugal: wastewater treatment plant at Febros river, Vila Nova de Gaia	**Light:dark cycle**	12:12-h
**Latitude & Longitude**	41.11812 N 8.570944 W	**Temperature (ºC)**	19
		**Light intensity**	10–30 µmol photons m−2 s−1
**References Publicly Available Articles**			
**References Publicly Available Theses**			

**Strain ID**	**Tychonema sp. LEGE 07215**		
**Strain Taxonomy**	Tychonema Anagnostidis, K & Komárek, J (1988)		
**Microphotograph 400×**	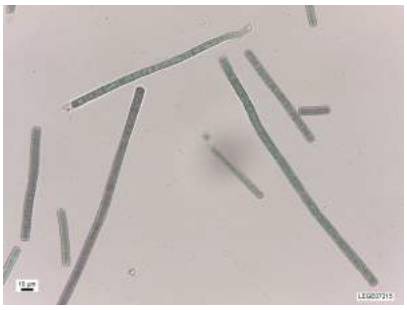	**Microphotograph 1000×**	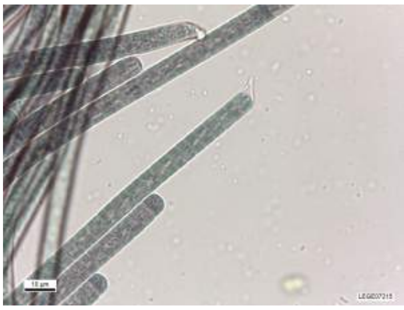
**Coidentity**	n.a.	**Morphometrics (Width × Length or Diameter) (µm)**	6,6±0,4 × 4,2±0,4
**Other Code(s)**		**Taxonomic notes/diacritical features**	trichomes sometimes slightly bent and narrowed toward ends; terminal cells rounded, blunted or capitate, often with a narrow calyptra; keritomized chromatoplasma; facultative sheath
**Parent Sample**	A1 JAN - 17 17i	**Macroscopic growth features**	color: olive green; forming a firm biofilm
**Isolation Date**		**Environment**	aquatic, freshwater
**Order (Older Classification Scheme)**	Oscillatoriales	**Habitat Sample Description**	biofilm, from a biological treatment tank outlet
**Order 2014 (Modern Taxonomy)**	Oscillatoriales	**Ecophysiological traits**	
**Taxonomy Notes**	Phormidium sp., group VII (Komárek & Anagnostidis, 2007)	**Notes on ecophysiological traits**	
**Accession Number(s)_16S**	KC989698	**Isolator**	Joana Martins
**Accession Number(s)_others (product/gene /region)**	KF008255 (cyanobactin, N-terminal protease (A))	**Medium**	Z8
**Collector**	Joana Martins	**Strain Status**	non-axenic, unicyanobacterial
**Collection Date**	Jan-2007	**Preservation type**	subculturing
**Location**	Portugal: wastewater treatment plant at Febros river, Vila Nova de Gaia	**Light:dark cycle**	12:12-h
**Latitude & Longitude**	41.11812 N 8.570944 W	**Temperature (ºC)**	19
		**Light intensity**	10–30 µmol photons m−2 s−1
**References Publicly Available Articles**	Martins et al. (2013) Mar. Drugs 11 (12), 4902-4916		
**References Publicly Available Theses**			

Strain ID	**Tychonema sp. LEGE 07216**		
**Strain Taxonomy**	Tychonema Anagnostidis, K & Komárek, J (1988)		
**Microphotograph 400×**	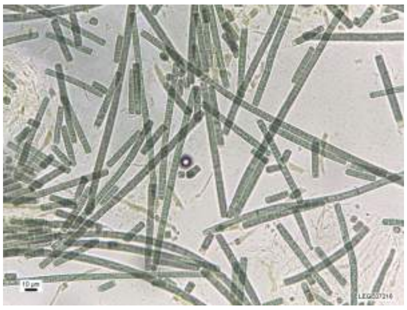	**Microphotograph 1000×**	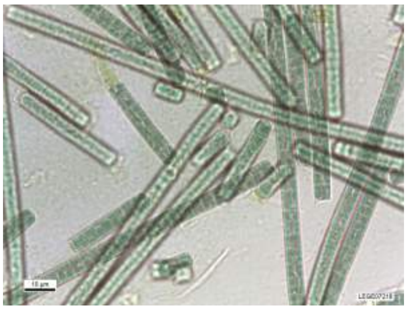
**Coidentity**	n.a.	**Morphometrics (Width × Length or Diameter) (µm)**	5,6±0,4 × 3,7±0,7
**Other Code(s)**		**Taxonomic notes/diacritical features**	trichomes sometimes slightly bent and narrowed toward ends; terminal cells rounded, blunted or capitate, often with a narrow calyptra; keritomized chromatoplasma; facultative sheath
**Parent Sample**	A1 MAR - P1 24i	**Macroscopic growth features**	color: olive green; forming a firm biofilm
**Isolation Date**		**Environment**	aquatic, freshwater
**Order (Older Classification Scheme)**	Oscillatoriales	**Habitat Sample Description**	biofilm, from a biological treatment tank outlet
**Order 2014 (Modern Taxonomy)**	Oscillatoriales	**Ecophysiological traits**	
**Taxonomy Notes**		**Notes on ecophysiological traits**	
**Accession Number(s)_16S**	KU951840	**Isolator**	Joana Martins
**Accession Number(s)_others (product/gene /region)**		**Medium**	Z8
**Collector**	Joana Martins	**Strain Status**	non-axenic, unicyanobacterial
**Collection Date**	Mar-2007	**Preservation type**	subculturing
**Location**	Portugal: wastewater treatment plant at Febros river, Vila Nova de Gaia	**Light:dark cycle**	12:12-h
**Latitude & Longitude**	41.11812 N 8.570944 W	**Temperature (ºC)**	19
**References Publicly Available Articles**		**Light intensity**	10–30 µmol photons m−2 s−1
**References Publicly Available Theses**			

**Strain ID**	**Tychonema sp. LEGE 07217**		
**Strain Taxonomy**	Tychonema Anagnostidis, K & Komárek, J (1988)		
**Microphotograph 400×**	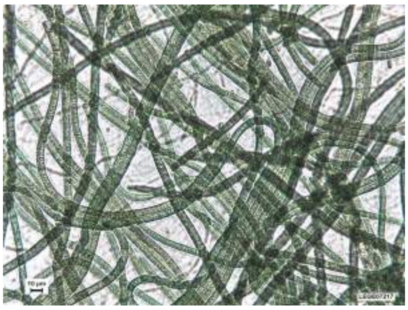	**Microphotograph 1000×**	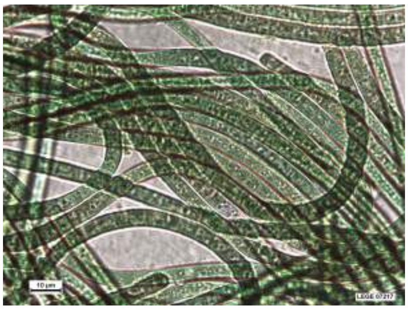
**Coidentity**	n.a.	**Morphometrics (Width × Length or Diameter) (µm)**	5,3±0,5 × 3,8±0,6
**Other Code(s)**		**Taxonomic notes/diacritical features**	trichomes sometimes slightly bent and narrowed toward ends; terminal cells rounded, blunted or capitate, often with a narrow calyptra; keritomized chromatoplasma; facultative sheath
**Parent Sample**	A1 MAR - 4 23i	**Macroscopic growth features**	color: olive green; forming a firm biofilm
**Isolation Date**		**Environment**	aquatic, freshwater
**Order (Older Classification Scheme)**	Oscillatoriales	**Habitat Sample Description**	biofilm, from a biological treatment tank outlet
**Order 2014 (Modern Taxonomy)**	Oscillatoriales	**Ecophysiological traits**	
**Taxonomy Notes**		**Notes on ecophysiological traits**	
**Accession Number(s)_16S**	KU951841	**Isolator**	Joana Martins
**Accession Number(s)_others (product/gene /region)**		**Medium**	Z8
**Collector**	Joana Martins	**Strain Status**	non-axenic, unicyanobacterial, delicate
**Collection Date**	Mar-2007	**Preservation type**	subculturing
**Location**	Portugal: wastewater treatment plant at Febros river, Vila Nova de Gaia	**Light:dark cycle**	12:12-h
**Latitude & Longitude**	41.11812 N 8.570944 W	**Temperature (ºC)**	19
		**Light intensity**	10–30 µmol photons m−2 s−1
**References Publicly Available Articles**			
**References Publicly Available Theses**			

Strain ID	**Tychonema sp. LEGE 07221**		
**Strain Taxonomy**	Tychonema Anagnostidis, K & Komárek, J (1988)		
**Microphotograph 400×**	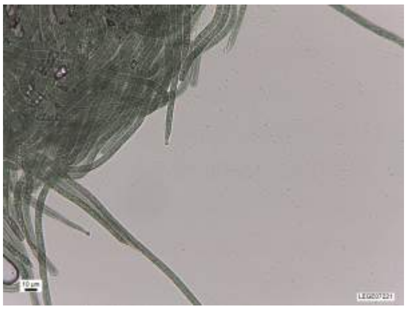	**Microphotograph 1000×**	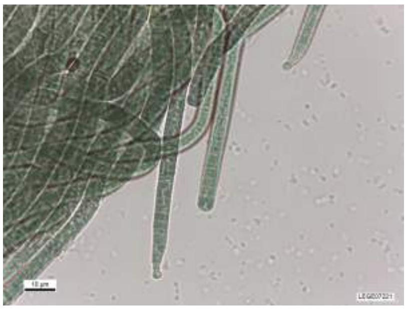
**Coidentity**	n.a.	**Morphometrics (Width × Length or Diameter) (µm)**	5,4±0,6 × 3,7±0,7
**Other Code(s)**		**Taxonomic notes/diacritical features**	trichomes sometimes slightly bent and narrowed toward ends; terminal cells rounded, blunted or capitate, often with a narrow calyptra; keritomized chromatoplasma; facultative sheath
**Parent Sample**	A1 F2	**Macroscopic growth features**	color: olive green; forming a firm biofilm
**Isolation Date**		**Environment**	aquatic, freshwater
**Order (Older Classification Scheme)**	Oscillatoriales	**Habitat Sample Description**	biofilm, from a biological treatment tank outlet
**Order 2014 (Modern Taxonomy)**	Oscillatoriales	**Ecophysiological traits**	
**Taxonomy Notes**		**Notes on ecophysiological traits**	
**Accession Number(s)_16S**	KU951842	**Isolator**	Joana Martins
**Accession Number(s)_others (product/gene /region)**		**Medium**	Z8
**Collector**	Joana Martins	**Strain Status**	non-axenic, unicyanobacterial
**Collection Date**	Feb-2007	**Preservation type**	subculturing
**Location**	Portugal: wastewater treatment plant at Febros river, Vila Nova de Gaia	**Light:dark cycle**	12:12-h
**Latitude & Longitude**	41.11812 N 8.570944 W	**Temperature (ºC)**	19
		**Light intensity**	10–30 µmol photons m−2 s−1
**References Publicly Available Articles**			
**References Publicly Available Theses**			

Strain ID	**aff. Leptolyngbya ectocarpi LEGE 11473**		
**Strain Taxonomy**	Leptolyngbya ectocarpi (Gomont) Anagnostidis, K & Komárek, J (1988)		
**Microphotograph 400×**	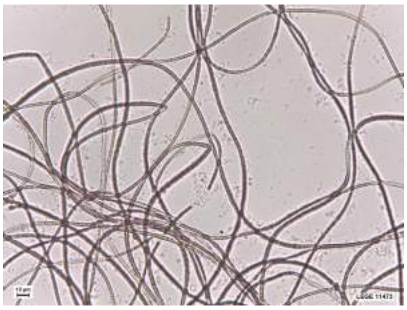	**Microphotograph 1000×**	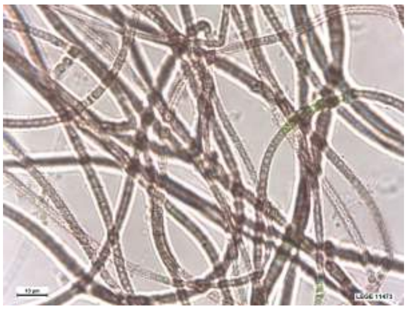
**Coidentity**	n.a.	**Morphometrics (Width × Length or Diameter) (µm)**	2,5±0,4 × 2,2±0,5
**Other Code(s)**		**Taxonomic notes/diacritical features**	with sheath
**Parent Sample**	3.001.0911	**Macroscopic growth features**	color: brownish; forming a smooth biofilm and aggregates
**Isolation Date**		**Environment**	aquatic, marine
**Order (Older Classification Scheme)**	Oscillatoriales	**Habitat Sample Description**	subtidal sample, epilithic (6 m depth), near the south quay of the Leixões harbour
**Order 2014 (Modern Taxonomy)**	Synechococcales	**Ecophysiological traits**	
**Taxonomy Notes**		**Notes on ecophysiological traits**	
**Accession Number(s)_16S**	KU951663	**Isolator**	Ângela Pinheiro
**Accession Number(s)_others (product/gene /region)**		**Medium**	Z8 25‰ TM sea salt w/ vit. B12
**Collector**	Pedro Leão/Aldo Barreiro	**Strain Status**	non-axenic, unicyanobacterial
**Collection Date**	2011/09/26	**Preservation type**	subculturing
**Location**	Portugal: at [115], diving spot near Leixões Harbour	**Light:dark cycle**	12:12-h
**Latitude & Longitude**	41.17652 N 8.700633 W	**Temperature (ºC)**	19
		**Light intensity**	10–30 µmol photons m−2 s−1
**References Publicly Available Articles**			
**References Publicly Available Theses**			

Strain ID	**aff. Leptolyngbya ectocarpi LEGE 11478**		
**Strain Taxonomy**	Leptolyngbya ectocarpi (Gomont) Anagnostidis, K & Komárek, J (1988)		
**Microphotograph 400×**	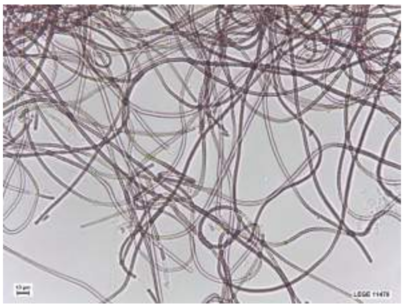	**Microphotograph 1000×**	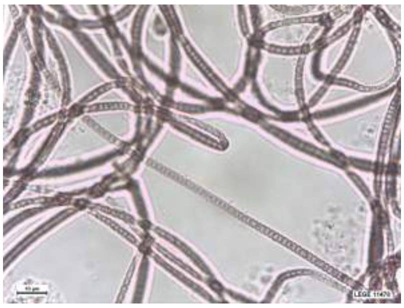
**Coidentity**	n.a.	**Morphometrics (Width × Length or Diameter) (µm)**	2,0±0,3 × 1,5±0,5
**Other Code(s)**		**Taxonomic notes/diacritical features**	with isodiametric cells; with sheath
**Parent Sample**	3.002.0911	**Macroscopic growth features**	color: brownish; forming a firm biofilm
**Isolation Date**		**Environment**	aquatic, marine
**Order (Older Classification Scheme)**	Oscillatoriales	**Habitat Sample Description**	subtidal sample, epilithic (10-13m depth), less than 1 km off the shore
**Order 2014 (Modern Taxonomy)**	Synechococcales	**Ecophysiological traits**	
**Taxonomy Notes**		**Notes on ecophysiological traits**	
**Accession Number(s)_16S**	ready to submit	**Isolator**	Ângela Pinheiro
**Accession Number(s)_others (product/gene /region)**		**Medium**	Z8 25‰ TM sea salt w/ vit. B12
**Collector**	Pedro Leão/Aldo Barreiro	**Strain Status**	non-axenic, unicyanobacterial
**Collection Date**	2011/09/26	**Preservation type**	subculturing
**Location**	Portugal: 'Pêlo Negro', diving spot near Leixões Harbour	**Light:dark cycle**	12:12-h
**Latitude & Longitude**	41.18557 N 8.719100 W	**Temperature (ºC)**	19
		**Light intensity**	10–30 µmol photons m−2 s−1
**References Publicly Available Articles**			
**References Publicly Available Theses**			

Strain ID	**aff. Nodosilinea sp. LEGE 06148**		
**Strain Taxonomy**	Nodosilinea Perkerson, RB & Casamatta DA in Perkerson et al (2011)		
**Microphotograph 400×**	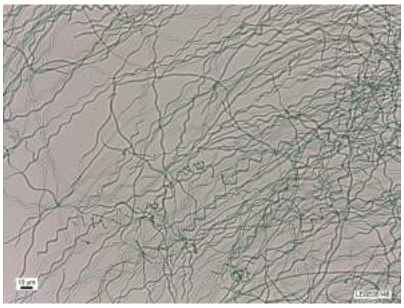	**Microphotograph 1000×**	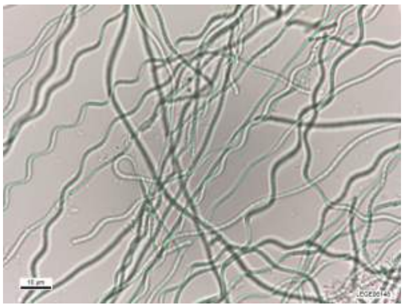
**Coidentity**	n.a.	**Morphometrics (Width × Length or Diameter) (µm)**	1,3±0,2 × 1,8±0,3
**Other Code(s)**	LEAN 061	**Taxonomic notes/diacritical features**	loose helical coiled filaments observed
**Parent Sample**	O.06 MOL 19 - GEA-B	**Macroscopic growth features**	color: cyano; forming a firm biofilm
**Isolation Date**		**Environment**	aquatic, marine
**Order (Older Classification Scheme)**	Oscillatoriales	**Habitat Sample Description**	intertidal zone, wave-exposed rock surface scraping
**Order 2014 (Modern Taxonomy)**	Synechococcales	**Ecophysiological traits**	
**Taxonomy Notes**		**Notes on ecophysiological traits**	
**Accession Number(s)_16S**	KU951664	**Isolator**	Vitor Ramos
**Accession Number(s)_others (product/gene /region)**		**Medium**	Z8 25‰ TM sea salt w/ vit. B12
**Collector**	Rui Seabra/Vitor Ramos	**Strain Status**	non-axenic, unicyanobacterial
**Collection Date**	2006/11/20	**Preservation type**	subculturing
**Location**	Portugal: Praia de Moledo, Caminha	**Light:dark cycle**	12:12-h
**Latitude & Longitude**	41.84963 N 8.866717 W	**Temperature (ºC)**	19
		**Light intensity**	10–30 µmol photons m−2 s−1
**References Publicly Available Articles**	Leão et al. (2013) Mar. Drugs 11 (4), 1316-1335		
**References Publicly Available Theses**			

Strain ID	**aff. Nodosilinea sp. LEGE 06149.2**		
**Strain Taxonomy**	Nodosilinea Perkerson, RB & Casamatta DA in Perkerson et al (2011)		
**Microphotograph 400×**	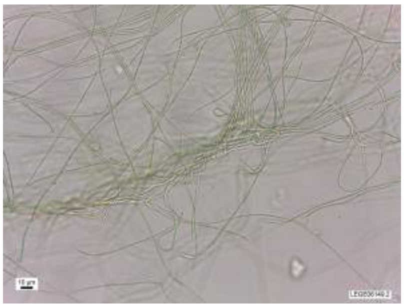	**Microphotograph 1000×**	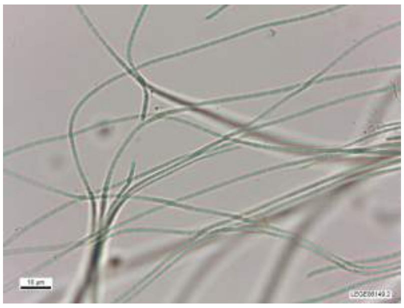
**Coidentity**	n.a.	**Morphometrics (Width × Length or Diameter) (µm)**	1,3±0,2 × 3,5±0,6
**Other Code(s)**	LEAN 062.2	**Taxonomic notes/diacritical features**	with sheath
**Parent Sample**	O.06 MOL 2 - DAA/DB-A/C	**Macroscopic growth features**	color: cyano; forming a firm biofilm
**Isolation Date**		**Environment**	aquatic, marine
**Order (Older Classification Scheme)**	Oscillatoriales	**Habitat Sample Description**	tide puddle, on a submerged stone
**Order 2014 (Modern Taxonomy)**	Synechococcales	**Ecophysiological traits**	
**Taxonomy Notes**		**Notes on ecophysiological traits**	
**Accession Number(s)_16S**	KU951773	**Isolator**	Vitor Ramos
**Accession Number(s)_others (product/gene /region)**		**Medium**	MN w/ vit. B12
**Collector**	Rui Seabra/Vitor Ramos	**Strain Status**	non-axenic, unicyanobacterial
**Collection Date**	2006/11/20	**Preservation type**	subculturing
**Location**	Portugal: Praia de Moledo, Caminha	**Light:dark cycle**	12:12-h
**Latitude & Longitude**	41.84963 N 8.866717 W	**Temperature (ºC)**	19
		**Light intensity**	10–30 µmol photons m−2 s−1
**References Publicly Available Articles**			
**References Publicly Available Theses**			

**Strain ID**	**aff. Planktothrix mougeotii LEGE 06224**		
**Strain Taxonomy**	Planktothrix mougeotii (Kützing ex Lemmermann) Suda, S; Watanabe, M; Otsuka, S; Mahakahant, A; Yongmanitchai, W; Nopartnaraporn, N; Liu, Y & Day, JG (2002)		
**Microphotograph 400×**	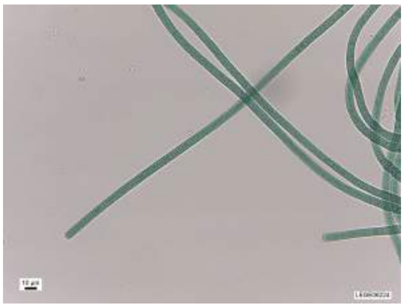	**Microphotograph 1000×**	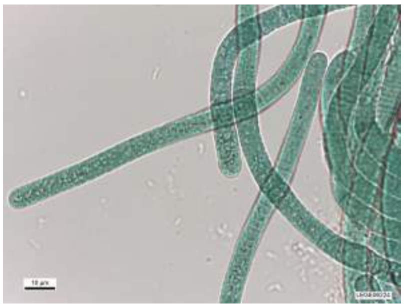
**Coidentity**	n.a.	**Morphometrics (Width × Length or Diameter) (µm)**	6,0±0,7 × 2,4±0,3
**Other Code(s)**		**Taxonomic notes/diacritical features**	trichomes with vacuolated cells, hormogonia and necridic cells; facultative sheath
**Parent Sample**	A5 24/10 - 11	**Macroscopic growth features**	color: cyano; forming a smooth biofilm and aggregates
**Isolation Date**		**Environment**	aquatic, freshwater
**Order (Older Classification Scheme)**	Oscillatoriales	**Habitat Sample Description**	floating masses, from a secondary decanter tank bank
**Order 2014 (Modern Taxonomy)**	undefined	**Ecophysiological traits**	
**Taxonomy Notes**		**Notes on ecophysiological traits**	
**Accession Number(s)_16S**	KU951665	**Isolator**	Joana Martins
**Accession Number(s)_others (product/gene /region)**		**Medium**	Z8
**Collector**	Joana Martins	**Strain Status**	non-axenic, unicyanobacterial
**Collection Date**	2006/10/27	**Preservation type**	subculturing
**Location**	Portugal: wastewater treatment plant at Febros river, Vila Nova de Gaia	**Light:dark cycle**	12:12-h
**Latitude & Longitude**	41.11812 N 8.570944 W	**Temperature (ºC)**	19
		**Light intensity**	10–30 µmol photons m−2 s−1
**References Publicly Available Articles**			
**References Publicly Available Theses**			

Strain ID	**aff. Planktothrix mougeotii LEGE 07227**		
**Strain Taxonomy**	Planktothrix mougeotii (Kützing ex Lemmermann) Suda, S; Watanabe, M; Otsuka, S; Mahakahant, A; Yongmanitchai, W; Nopartnaraporn, N; Liu, Y & Day, JG (2002)		
**Microphotograph 400×**	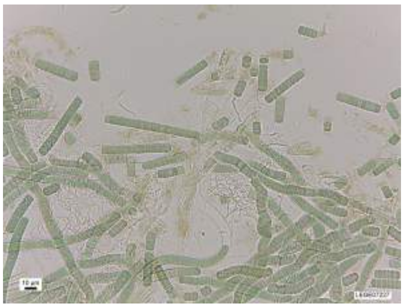	**Microphotograph 1000×**	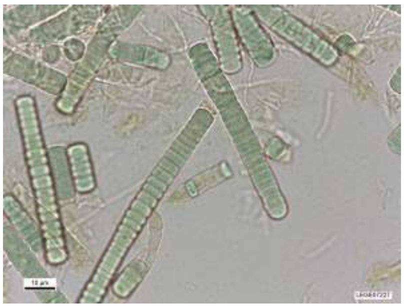
**Coidentity**	n.a.	**Morphometrics (Width × Length or Diameter) (µm)**	6,8±0,8 × 2,6±0,4
**Other Code(s)**		**Taxonomic notes/diacritical features**	trichomes slightly constricted at cross-walls; discoid cells; hormogonia and necridic cells; with sheath
**Parent Sample**	A5 M2 29i	**Macroscopic growth features**	color: cyano; forming a firm biofilm
**Isolation Date**		**Environment**	aquatic, freshwater
**Order (Older Classification Scheme)**	Oscillatoriales	**Habitat Sample Description**	floating masses, from a secondary decanter tank bank
**Order 2014 (Modern Taxonomy)**	undefined	**Ecophysiological traits**	
**Taxonomy Notes**		**Notes on ecophysiological traits**	
**Accession Number(s)_16S**	KU951666	**Isolator**	Joana Martins
**Accession Number(s)_others (product/gene /region)**		**Medium**	Z8
**Collector**	Joana Martins	**Strain Status**	non-axenic, unicyanobacterial
**Collection Date**	May-2007	**Preservation type**	subculturing
**Location**	Portugal: wastewater treatment plant at Febros river, Vila Nova de Gaia	**Light:dark cycle**	12:12-h
**Latitude & Longitude**	41.11812 N 8.570944 W	**Temperature (ºC)**	19
		**Light intensity**	10–30 µmol photons m−2 s−1
**References Publicly Available Articles**			
**References Publicly Available Theses**			

Strain ID	**aff. Roholtiella sp. LEGE 12411**		
**Strain Taxonomy**	Roholtiella Bohunická, M, Pietrasiak, N & Johansen, JR in Bohunická et al (2015)		
**Microphotograph 400×**	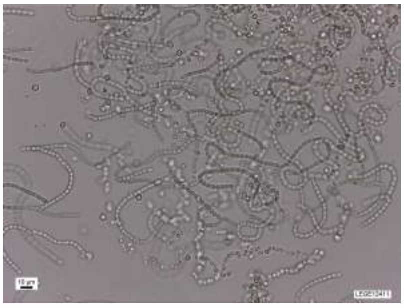	**Microphotograph 1000×**	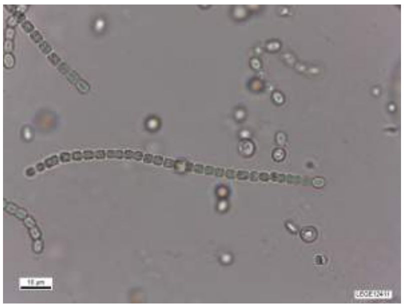
**Coidentity**	n.a.	**Morphometrics (Width × Length or Diameter) (µm)**	3,0±0,2 × 3,2±0,5 (cells); 3,7±0,5 × 3,1±0,5 (heterocytes)
**Other Code(s)**		**Taxonomic notes/diacritical features**	some sac-like gelatinous thalli; with sheath
**Parent Sample**	MOS 7-BAA	**Macroscopic growth features**	color: greenish; forming aggregates; mucilaginous
**Isolation Date**		**Environment**	aquatic, freshwater
**Order (Older Classification Scheme)**	Nostocales	**Habitat Sample Description**	benthic mat, on an ephemeral stream
**Order 2014 (Modern Taxonomy)**	Nostocales	**Ecophysiological traits**	nitrogen-fixation, EPS production
**Taxonomy Notes**		**Notes on ecophysiological traits**	
**Accession Number(s)_16S**	KU951667	**Isolator**	Vitor Ramos
**Accession Number(s)_others (product/gene /region)**		**Medium**	BG11o
**Collector**	Vitor Ramos	**Strain Status**	non-axenic, unicyanobacterial
**Collection Date**	2012	**Preservation type**	subculturing
**Location**	Portugal: Requeixada, Mós, Torre de Moncorvo	**Light:dark cycle**	12:12-h
**Latitude & Longitude**	unknown	**Temperature (ºC)**	19
		**Light intensity**	10–30 µmol photons m−2 s−1
**References Publicly Available Articles**			
**References Publicly Available Theses**			

**Strain ID**	**cf. Oculatella sp. LEGE 06141**		
**Strain Taxonomy**	Oculatella Zammit, G; Billi, D & Albertano, P (2012)		
**Microphotograph 400×**	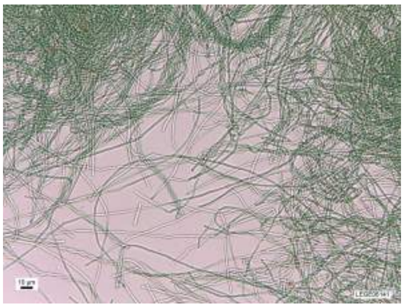	**Microphotograph 1000×**	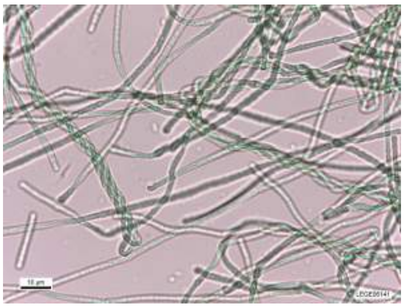
**Coidentity**	n.a.	**Morphometrics (Width × Length or Diameter) (µm)**	1,5±0,2 × 3,4±0,4
**Other Code(s)**	LEAN 052	**Taxonomic notes/diacritical features**	some filaments loosely coiled; terminal cells sometimes irregularly shaped
**Parent Sample**	O.06 LUZ 3 - CAA	**Macroscopic growth features**	color: cyano; forming a smooth biofilm
**Isolation Date**		**Environment**	aquatic, marine
**Order (Older Classification Scheme)**	Oscillatoriales	**Habitat Sample Description**	intertidal zone, on green macroalga
**Order 2014 (Modern Taxonomy)**	Synechococcales	**Ecophysiological traits**	
**Taxonomy Notes**		**Notes on ecophysiological traits**	
**Accession Number(s)_16S**	KU951789	**Isolator**	Vitor Ramos
**Accession Number(s)_others (product/gene /region)**		**Medium**	Z8 25‰ TM sea salt w/ vit. B12
**Collector**	Rui Seabra	**Strain Status**	non-axenic, unicyanobacterial
**Collection Date**	2006/12/12	**Preservation type**	subculturing
**Location**	Portugal: Praia da Luz, Lagos	**Light:dark cycle**	12:12-h
**Latitude & Longitude**	37.08622 N 8.725375 W	**Temperature (ºC)**	19
		**Light intensity**	10–30 µmol photons m−2 s−1
**References Publicly Available Articles**			
**References Publicly Available Theses**			

**Strain ID**	**cf. Oxynema acuminatum LEGE 06072**		
**Strain Taxonomy**	Oxynema acuminatum (Gomont) Chatchawan, T; Komárek, J; Strunecky, O; Smarda, J & Peerapornpisal, Y (2012)		
**Microphotograph 400×**	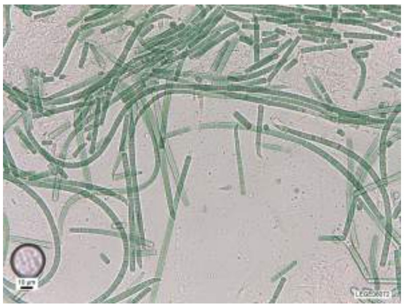	**Microphotograph 1000×**	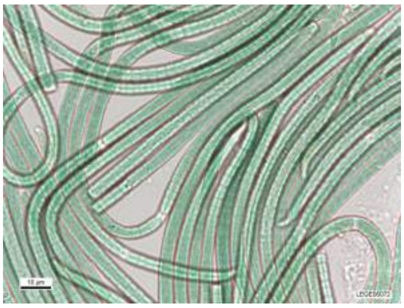
**Coidentity**	n.a.	**Morphometrics (Width × Length or Diameter) (µm)**	4,1±0,3 × 2,9±0,9
**Other Code(s)**		**Taxonomic notes/diacritical features**	sometimes, the end of the trichome is distinctly hooked; facultative sheath
**Parent Sample**	VL 104	**Macroscopic growth features**	color: cyano; forming a firm biofilm
**Isolation Date**		**Environment**	aquatic, brackish
**Order (Older Classification Scheme)**	Oscillatoriales	**Habitat Sample Description**	mesotidal zone, benthic
**Order 2014 (Modern Taxonomy)**	Oscillatoriales	**Ecophysiological traits**	
**Taxonomy Notes**	Phormidium sp., group II (Komárek & Anagnostidis, 2007)	**Notes on ecophysiological traits**	
**Accession Number(s)_16S**	HM217077	**Isolator**	Viviana Lopes
**Accession Number(s)_others (product/gene /region)**		**Medium**	Z8
**Collector**	Viviana Lopes	**Strain Status**	non-axenic, unicyanobacterial
**Collection Date**	Dec-2006	**Preservation type**	subculturing
**Location**	Portugal: Vouga estuary, Ria de Aveiro, São Jacinto	**Light:dark cycle**	12:12-h
**Latitude & Longitude**	40.66644 N 8.725806 W	**Temperature (ºC)**	19
		**Light intensity**	10–30 µmol photons m−2 s−1
**References Publicly Available Articles**	Lopes et al. (2010) Mar. Drugs 8, 471-482; Baptista et al. (2011) Toxicon 58, 410–414; Lopes et al. (2011) Toxicol. In Vitro 25, 944–950; Lopes & Vasconcelos (2011) Mar. Drugs 9, 790-802; Cianca et al. (2012) Amino Acids 42, 2473–2479; Lopes et al. (2012) Mar. Environ. Res. 73, 7–16; Almeida et al. (2015) Toxins 7(8), 2739-2756		
**References Publicly Available Theses**	Lopes V.R. (2011) PhD Thesis, FCUP, University of Porto, Porto, Portugal; Macieira R. (2013) MSc Dissertation, FCUP, University of Porto, Porto, Portugal		

**Strain ID**	**cf. Oxynema acuminatum LEGE 06078**		
**Strain Taxonomy**	Oxynema acuminatum (Gomont) Chatchawan, T; Komárek, J; Strunecky, O; Smarda, J & Peerapornpisal, Y (2012)		
**Microphotograph 400×**	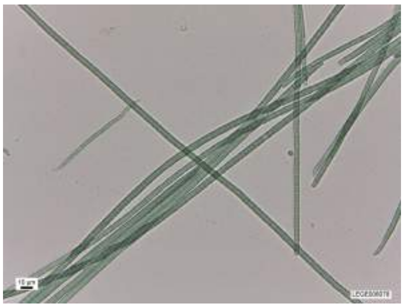	**Microphotograph 1000×**	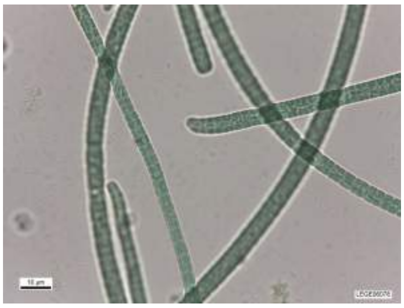
**Coidentity**	n.a.	**Morphometrics (Width × Length or Diameter) (µm)**	4,7±0,5 × 2,9±0,5
**Other Code(s)**		**Taxonomic notes/diacritical features**	sometimes, the end of the trichome is distinctly hooked; hormogonia
**Parent Sample**	VL 26	**Macroscopic growth features**	color: cyano; forming a smooth biofilm
**Isolation Date**		**Environment**	aquatic, brackish
**Order (Older Classification Scheme)**	Oscillatoriales	**Habitat Sample Description**	mesotidal zone, benthic
**Order 2014 (Modern Taxonomy)**	Oscillatoriales	**Ecophysiological traits**	
**Taxonomy Notes**	Phormidium sp., group II (Komárek & Anagnostidis, 2007)	**Notes on ecophysiological traits**	
**Accession Number(s)_16S**	HM217075	**Isolator**	Viviana Lopes
**Accession Number(s)_others (product/gene /region)**		**Medium**	Z8
**Collector**	Viviana Lopes	**Strain Status**	non-axenic, unicyanobacterial
**Collection Date**	2006/10/27	**Preservation type**	subculturing
**Location**	Portugal: Douro estuary, Vila Nova de Gaia	**Light:dark cycle**	12:12-h
**Latitude & Longitude**	41.13742 N 8.663667 W	**Temperature (ºC)**	19
		**Light intensity**	10–30 µmol photons m−2 s−1
**References Publicly Available Articles**	Lopes et al. (2010) Mar. Drugs 8, 471-482; Baptista et al. (2011) Toxicon 58, 410–414; Lopes et al. (2011) Toxicol. In Vitro 25, 944–950; Lopes & Vasconcelos (2011) Mar. Drugs 9, 790-802; Cianca et al. (2012) Amino Acids 42, 2473–2479; Lopes et al. (2012) Mar. Environ. Res. 73, 7–16; Rastoll et al. (2013) J. Appl. Phycol. 25, 1483-1493; Almeida et al. (2015) Toxins 7(8), 2739-2756		
**References Publicly Available Theses**	Lopes V.R. (2011) PhD Thesis, FCUP, University of Porto, Porto, Portugal; Macieira R. (2013) MSc Dissertation, FCUP, University of Porto, Porto, Portugal		

**Strain ID**	**cf. Phormidesmis sp. LEGE 10370**		
**Strain Taxonomy**	Phormidesmis Turicchia, S; Ventura, S; Komárková, J & Komárek, J (2009)		
**Microphotograph 400×**	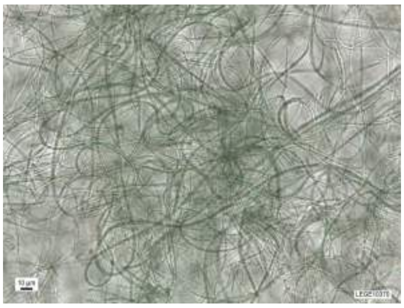	**Microphotograph 1000×**	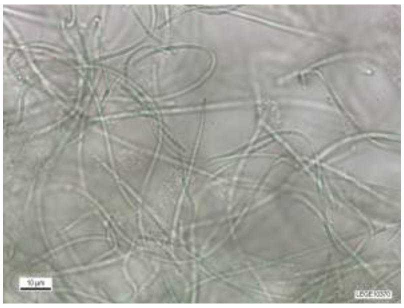
**Coidentity**	n.a.	**Morphometrics (Width × Length or Diameter) (µm)**	1,2±0,2 × 1,5±0,3
**Other Code(s)**	12A	**Taxonomic notes/diacritical features**	trichomes constricted at cross-walls; facultative sheath
**Parent Sample**		**Macroscopic growth features**	color: cyano; forming a smooth biofilm
**Isolation Date**		**Environment**	aquatic, marine
**Order (Older Classification Scheme)**	Oscillatoriales	**Habitat Sample Description**	intertidal zone, on a marine sponge
**Order 2014 (Modern Taxonomy)**	Synechococcales	**Ecophysiological traits**	
**Taxonomy Notes**		**Notes on ecophysiological traits**	
**Accession Number(s)_16S**	JQ927344	**Isolator**	Ana Regueiras
**Accession Number(s)_others (product/gene /region)**		**Medium**	Z8 25‰ TM sea salt w/ vit. B12
**Collector**	Ana Regueiras	**Strain Status**	non-axenic, unicyanobacterial
**Collection Date**	2010/09/10	**Preservation type**	subculturing
**Location**	Portugal: Praia da Memória	**Light:dark cycle**	12:12-h
**Latitude & Longitude**	41.23119 N 8.721750 W	**Temperature (ºC)**	19
		**Light intensity**	10–30 µmol photons m−2 s−1
**References Publicly Available Articles**			
**References Publicly Available Theses**			

**Strain ID**	**cf. Phormidesmis sp. LEGE 11430**		
**Strain Taxonomy**	Phormidesmis Turicchia, S; Ventura, S; Komárková, J & Komárek, J (2009)		
**Microphotograph 400×**	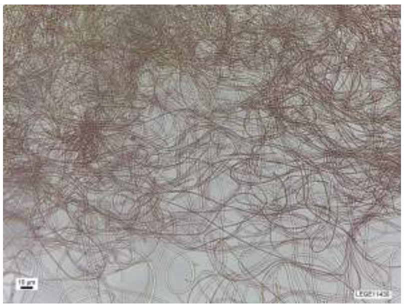	**Microphotograph 1000×**	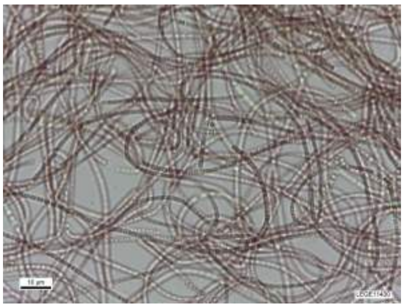
**Coidentity**	n.a.	**Morphometrics (Width × Length or Diameter) (µm)**	1,4±0,2 × 1,8±0,2
**Other Code(s)**		**Taxonomic notes/diacritical features**	
**Parent Sample**	12.003.1011_AP.H(2)1.A	**Macroscopic growth features**	color: brownish; forming a firm biofilm
**Isolation Date**		**Environment**	aquatic, marine
**Order (Older Classification Scheme)**	Oscillatoriales	**Habitat Sample Description**	subtidal sample, epilithic (13m depth), about 200 m off the shore
**Order 2014 (Modern Taxonomy)**	Synechococcales	**Ecophysiological traits**	
**Taxonomy Notes**		**Notes on ecophysiological traits**	
**Accession Number(s)_16S**	KU951669	**Isolator**	Ângela Pinheiro
**Accession Number(s)_others (product/gene /region)**		**Medium**	Z8 25‰ TM sea salt w/ vit. B12
**Collector**	Pedro Leão/Aldo Barreiro	**Strain Status**	non-axenic, unicyanobacterial
**Collection Date**	2011/10/03	**Preservation type**	subculturing, cryopreserved
**Location**	Portugal: 'A Pedra', diving spot in front of the fort 'Castelo do Queijo'	**Light:dark cycle**	12:12-h
**Latitude & Longitude**	41.185809 N 8.719079 W	**Temperature (ºC)**	19
		**Light intensity**	10–30 µmol photons m−2 s−1
**References Publicly Available Articles**			
**References Publicly Available Theses**			

Strain ID	**cf. Phormidesmis sp. LEGE 11434**		
**Strain Taxonomy**	Phormidesmis Turicchia, S; Ventura, S; Komárková, J & Komárek, J (2009)		
**Microphotograph 400×**	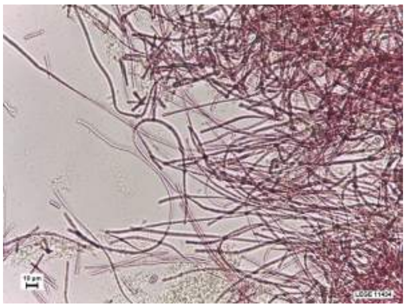	**Microphotograph 1000×**	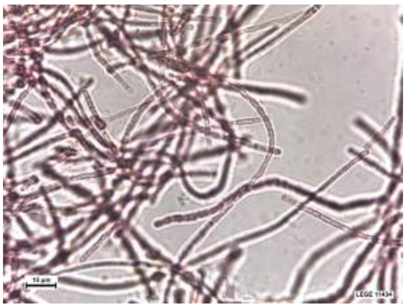
**Coidentity**	n.a.	**Morphometrics (Width × Length or Diameter) (µm)**	1,4±0,2 × 3,7±1,0
**Other Code(s)**		**Taxonomic notes/diacritical features**	with sheath
**Parent Sample**	2.002.09.11_AP.L/a	**Macroscopic growth features**	color: reddish; forming a smooth biofilm and aggregates
**Isolation Date**		**Environment**	aquatic, marine
**Order (Older Classification Scheme)**	Oscillatoriales	**Habitat Sample Description**	subtidal sample, epilithic (13m depth), about 200 m off the shore
**Order 2014 (Modern Taxonomy)**	Synechococcales	**Ecophysiological traits**	
**Taxonomy Notes**		**Notes on ecophysiological traits**	
**Accession Number(s)_16S**	KU951670	**Isolator**	Ângela Pinheiro
**Accession Number(s)_others (product/gene /region)**		**Medium**	Z8 25‰ TM sea salt w/ vit. B12
**Collector**	Pedro Leão/Aldo Barreiro	**Strain Status**	non-axenic, unicyanobacterial
**Collection Date**	2011/10/03	**Preservation type**	subculturing
**Location**	Portugal: 'A Pedra', diving spot in front of the fort 'Castelo do Queijo'	**Light:dark cycle**	12:12-h
**Latitude & Longitude**	41.185809 N 8.719079 W	**Temperature (ºC)**	19
		**Light intensity**	10–30 µmol photons m−2 s−1
**References Publicly Available Articles**			
**References Publicly Available Theses**			

Strain ID	**cf. Phormidesmis sp. LEGE 11475**		
**Strain Taxonomy**	Phormidesmis Turicchia, S; Ventura, S; Komárková, J & Komárek, J (2009)		
**Microphotograph 400×**	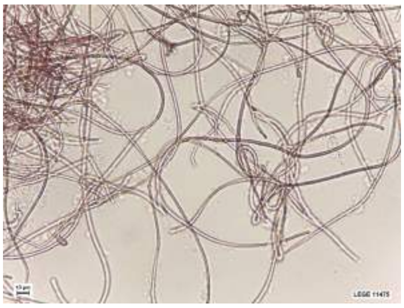	**Microphotograph 1000×**	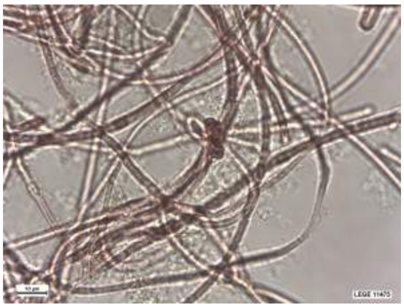
**Coidentity**	n.a.	**Morphometrics (Width × Length or Diameter) (μm)**	2,1±0,4 × 1,8±0,3
**Other Code(s)**		**Taxonomic notes/diacritical features**	with sheath
**Parent Sample**	9.003.1011	**Macroscopic growth features**	color: brownish; growing homogeneously and forming a smooth biofilm
**Isolation Date**		**Environment**	aquatic, marine
**Order (Older Classification Scheme)**	Oscillatoriales	**Habitat Sample Description**	subtidal sample, epilithic (13m depth), about 200 m off the shore
**Order 2014 (Modern Taxonomy)**	Synechococcales	**Ecophysiological traits**	
**Taxonomy Notes**		**Notes on ecophysiological traits**	
**Accession Number(s)_16S**	KU951671	**Isolator**	Ângela Pinheiro
**Accession Number(s)_others (product/gene /region)**		**Medium**	Z8 25‰ TM sea salt w/ vit. B12
**Collector**	Pedro Leão/Aldo Barreiro	**Strain Status**	non-axenic, unicyanobacterial, delicate
**Collection Date**	2011/10/03	**Preservation type**	subculturing
**Location**	Portugal: 'A Pedra', diving spot in front of the fort 'Castelo do Queijo'	**Light:dark cycle**	12:12-h
**Latitude & Longitude**	41.185809 N 8.719079 W	**Temperature (ºC)**	19
		**Light intensity**	10–30 μmol photons m−2 s−1
**References Publicly Available Articles**			
**References Publicly Available Theses**			

**Strain ID**	**cf. Phormidesmis sp. LEGE 11477**		
**Strain Taxonomy**	Phormidesmis Turicchia, S; Ventura, S; Komárková, J & Komárek, J (2009)		
**Microphotograph 400×**	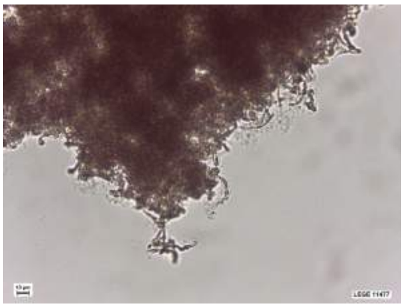	**Microphotograph 1000×**	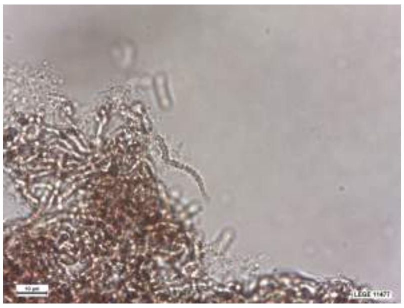
**Coidentity**	n.a.	**Morphometrics (Width × Length or Diameter) (µm)**	1,5±0,3 × 1,4±0,4
**Other Code(s)**		**Taxonomic notes/diacritical features**	with facultative sheath
**Parent Sample**	2.001.0911	**Macroscopic growth features**	color: brownish; growing homogeneously and forming a firm biofilm
**Isolation Date**		**Environment**	aquatic, marine
**Order (Older Classification Scheme)**	Oscillatoriales	**Habitat Sample Description**	subtidal sample, epilithic (6 m depth), near the south quay of the Leixões harbour
**Order 2014 (Modern Taxonomy)**	Synechococcales	**Ecophysiological traits**	
**Taxonomy Notes**		**Notes on ecophysiological traits**	
**Accession Number(s)_16S**	KU951672	**Isolator**	Ângela Pinheiro
**Accession Number(s)_others (product/gene /region)**		**Medium**	Z8 25‰ TM sea salt w/ vit. B12
**Collector**	Pedro Leão/Aldo Barreiro	**Strain Status**	non-axenic, unicyanobacterial
**Collection Date**	2011/09/26	**Preservation type**	subculturing
**Location**	Portugal: at [115], diving spot near Leixões Harbour	**Light:dark cycle**	12:12-h
**Latitude & Longitude**	41.17652 N 8.700633 W	**Temperature (ºC)**	19
		**Light intensity**	10–30 µmol photons m−2 s−1
**References Publicly Available Articles**			
**References Publicly Available Theses**			

**Strain ID**	**cf. Romeria sp. LEGE 06003**		
**Strain Taxonomy**	Romeria Koczwara, M in Geitler, L (1932)		
**Microphotograph 400×**	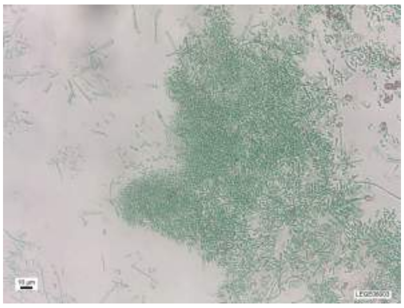	**Microphotograph 1000×**	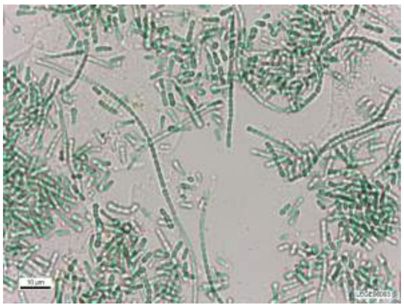
**Coidentity**	n.a.	**Morphometrics (Width × Length or Diameter) (µm)**	1,4±0,2 × 2,0±0,5
**Other Code(s)**	LEAN 124	**Taxonomic notes/diacritical features**	mainly short trichomes
**Parent Sample**	1 Buarcos	**Macroscopic growth features**	color: cyano; forming aggregates
**Isolation Date**		**Environment**	aquatic, marine
**Order (Older Classification Scheme)**	Oscillatoriales	**Habitat Sample Description**	intertidal zone
**Order 2014 (Modern Taxonomy)**	Synechococcales	**Ecophysiological traits**	
**Taxonomy Notes**	Pseudanabaena aff. curta/cf. Romeria sp.	**Notes on ecophysiological traits**	
**Accession Number(s)_16S**	KU951865	**Isolator**	Bárbara Frazão
**Accession Number(s)_others (product/gene /region)**		**Medium**	Z8 25‰ TM sea salt w/ vit. B12
**Collector**	Bárbara Frazão	**Strain Status**	non-axenic, unicyanobacterial
**Collection Date**	Fall 2006	**Preservation type**	subculturing
**Location**	Portugal: Praia de Buarcos	**Light:dark cycle**	12:12-h
**Latitude & Longitude**	40.15621 N 8.871803 W	**Temperature (ºC)**	19
		**Light intensity**	10–30 µmol photons m−2 s−1
**References Publicly Available Articles**			
**References Publicly Available Theses**			

**Strain ID**	**cf. Romeria sp. LEGE 06013**		
**Strain Taxonomy**	Romeria Koczwara, M in Geitler, L (1932)		
**Microphotograph 400×**	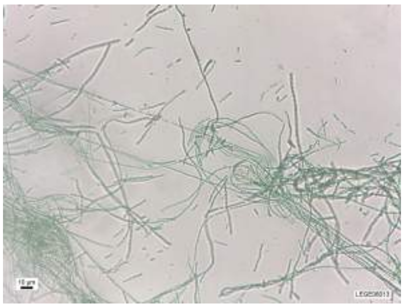	**Microphotograph 1000×**	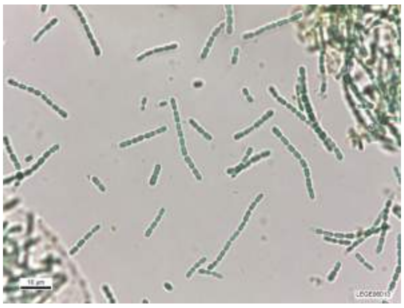
**Coidentity**	n.a.	**Morphometrics (Width × Length or Diameter) (µm)**	1,4±0,1 × 2,2±0,4
**Other Code(s)**	strain 15, LEAN 118	**Taxonomic notes/diacritical features**	irregular filaments, with terminal cells conical-rounded; constricted at cross-walls; facultative sheath
**Parent Sample**	Ref. 15, Arelho 1	**Macroscopic growth features**	color: cyano; growing homogeneously, but also forming aggregates
**Isolation Date**		**Environment**	aquatic, marine
**Order (Older Classification Scheme)**	Oscillatoriales	**Habitat Sample Description**	intertidal zone, wave-exposed rock
**Order 2014 (Modern Taxonomy)**	Synechococcales	**Ecophysiological traits**	
**Taxonomy Notes**		**Notes on ecophysiological traits**	
**Accession Number(s)_16S**	KU951673	**Isolator**	Bárbara Frazão
**Accession Number(s)_others (product/gene /region)**	KC842334, KC842335 (polyketide synthase)	**Medium**	Z8 25‰ TM sea salt w/ vit. B12
**Collector**	Bárbara Frazão	**Strain Status**	non-axenic, unicyanobacterial
**Collection Date**	Fall 2006	**Preservation type**	subculturing, cryopreserved
**Location**	Portugal: Praia da Foz do Arelho, Caldas da Rainha	**Light:dark cycle**	12:12-h
**Latitude & Longitude**	39.43327 N 9.230275 W	**Temperature (ºC)**	19
		**Light intensity**	10–30 µmol photons m−2 s−1
**References Publicly Available Articles**	Brito et al. (2012) Syst. Appl. Microbiol. 35, 110–119; Leão et al. (2013) Mar. Drugs 11 (4), 1316-1335; Rastoll et al. (2013) J. Appl. Phycol. 25, 1483-1493; Costa et al. (2014) Mar. Drugs 12, 98-114; Brito et al. (2015) Algal Res. 9, 218-226		
**References Publicly Available Theses**	Frazão B. (2008) MSc Dissertation, FCUP, University of Porto, Porto, Portugal; Ribeiro M.J. (2012) MSc Dissertation, Department of Chemistry, University of Aveiro, Aveiro, Portugal; Macieira R. (2013) MSc Dissertation, FCUP, University of Porto, Porto, Portugal		

Strain ID	**cf. Romeria sp. LEGE 11469**		
**Strain Taxonomy**	Romeria Koczwara, M in Geitler, L (1932)		
**Microphotograph 400×**	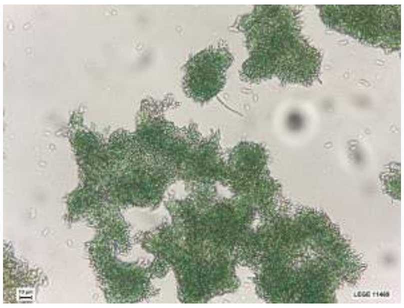	**Microphotograph 1000×**	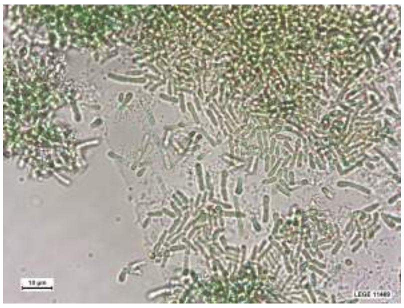
**Coidentity**	n.a.	**Morphometrics (Width × Length or Diameter) (µm)**	1,3±0,2 × 1,9±0,4
**Other Code(s)**		**Taxonomic notes/diacritical features**	short trichomes, constricted at cross-walls
**Parent Sample**	15.003.1011	**Macroscopic growth features**	color: cyano; growing homogeneously and forming a smooth biofilm
**Isolation Date**		**Environment**	aquatic, marine
**Order (Older Classification Scheme)**	Oscillatoriales	**Habitat Sample Description**	subtidal sample, epilithic (13m depth), about 200 m off the shore
**Order 2014 (Modern Taxonomy)**	Synechococcales	**Ecophysiological traits**	
**Taxonomy Notes**		**Notes on ecophysiological traits**	
**Accession Number(s)_16S**	KU951858	**Isolator**	Ângela Pinheiro
**Accession Number(s)_others (product/gene /region)**		**Medium**	Z8 25‰ TM sea salt w/ vit. B12
**Collector**	Pedro Leão/Aldo Barreiro	**Strain Status**	non-axenic, unicyanobacterial
**Collection Date**	2011/10/03	**Preservation type**	subculturing
**Location**	Portugal: 'A Pedra', diving spot in front of the fort 'Castelo do Queijo'	**Light:dark cycle**	12:12-h
**Latitude & Longitude**	41.185809 N 8.719079 W	**Temperature (ºC)**	19
		**Light intensity**	10–30 µmol photons m−2 s−1
**References Publicly Available Articles**			
**References Publicly Available Theses**			

Strain ID	unidentified Nostocales LEGE 03272		
Strain Taxonomy			
Microphotograph 400×	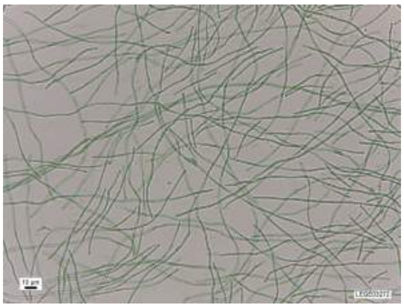	Microphotograph 1000×	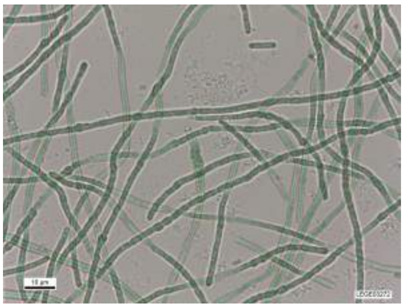
Coidentity	n.a.	Morphometrics (Width × Length or Diameter) (µm)	1,7±0,2 × 3,2±0,6
Other Code(s)	J68, LEANJ.68	Taxonomic notes/diacritical features	trichomes with irregularly shaped cells and anastomosis-like structures
Parent Sample		Macroscopic growth features	color: cyano; forming a smooth biofilm; mucilaginous
Isolation Date		Environment	aquatic, freshwater
Order (Older Classification Scheme)	Nostocales	Habitat Sample Description	water sample
Order 2014 (Modern Taxonomy)	Nostocales	Ecophysiological traits	EPS production
Taxonomy Notes		Notes on ecophysiological traits	
Accession Number(s)_16S		Isolator	Joana Osswald
Accession Number(s)_others (product/gene /region)		Medium	Z8
Collector	Joana Oswald	Strain Status	non-axenic, unicyanobacterial
Collection Date	2003/09/26	Preservation type	subculturing
Location	Portugal: Lagoa dos Salgados, Albufeira	Light:dark cycle	12:12-h
Latitude & Longitude	unknown	Temperature (ºC)	19
		Light intensity	10–30 µmol photons m−2 s−1
References Publicly Available Articles			
References Publicly Available Theses			

Strain ID	unidentified Nostocales LEGE 06106		
**Strain Taxonomy**			
**Microphotograph 400×**	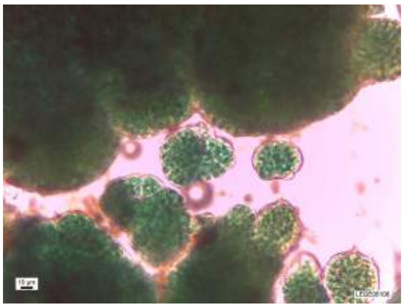	**Microphotograph 1000×**	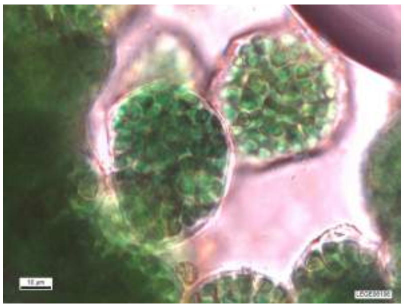
**Coidentity**	n.a.	**Morphometrics (Width × Length or Diameter) (µm)**	Ø 4,7±0,8 (Cells); Ø 45,1±10,1 (Colonies)
**Other Code(s)**	LEAN 010	**Taxonomic notes/diacritical features**	colonies of filaments, within mucilaginous envelopes
**Parent Sample**	O.06 BAR 10 - ABA	**Macroscopic growth features**	color: cyano; forming aggregates
**Isolation Date**		**Environment**	aquatic, marine
**Order (Older Classification Scheme)**	Nostocales	**Habitat Sample Description**	tide pool, rock surface scraping
**Order 2014 (Modern Taxonomy)**	Nostocales	**Ecophysiological traits**	nitrogen-fixation
**Taxonomy Notes**	Hapalosiphonaceae-like cyanobacterium	**Notes on ecophysiological traits**	
**Accession Number(s)_16S**	KU951876	**Isolator**	Vitor Ramos
**Accession Number(s)_others (product/gene /region)**	KC243672 (dinitrogenase reductase, nifH), KC813176 (non-ribosomal peptide synthetase), KC842306-KC842308 (polyketide synthase)	**Medium**	Z8 25‰ TM sea salt w/ vit. B12
**Collector**	Rui Seabra/Vitor Ramos	**Strain Status**	non-axenic, unicyanobacterial
**Collection Date**	2006/11/21	**Preservation type**	subculturing
**Location**	Portugal: Praia de São Bartolomeu do Mar, Esposende	**Light:dark cycle**	12:12-h
**Latitude & Longitude**	41.57377 N 8.798558 W	**Temperature (ºC)**	19
		**Light intensity**	10–30 µmol photons m−2 s−1
**References Publicly Available Articles**	Brito et al. (2012) Syst. Appl. Microbiol. 35, 110–119; Brito et al. (2015) Algal Res. 9, 218-226		
**References Publicly Available Theses**			

Strain ID	unidentified Nostocales LEGE 06150		
**Strain Taxonomy**			
**Microphotograph 400×**	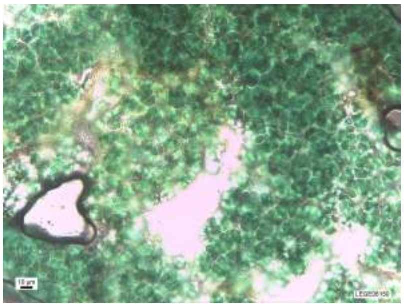	**Microphotograph 1000×**	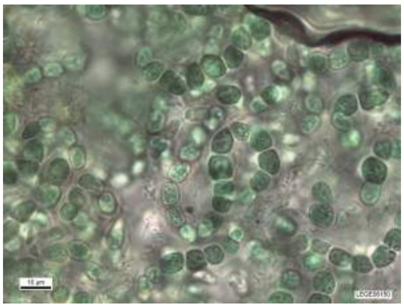
**Coidentity**	n.a.	**Morphometrics (Width × Length or Diameter) (µm)**	5,5±0,7 × 7,3±1,4
**Other Code(s)**	LEAN 063	**Taxonomic notes/diacritical features**	sac-like gelatinous thalli; true branching (?) observed; with sheath
**Parent Sample**	O.06 BAR 10 - ACB-J	**Macroscopic growth features**	color: cyano; forming aggregates
**Isolation Date**		**Environment**	aquatic, marine
**Order (Older Classification Scheme)**	Nostocales	**Habitat Sample Description**	tide pool, rock surface scraping
**Order 2014 (Modern Taxonomy)**	Nostocales	**Ecophysiological traits**	nitrogen-fixation
**Taxonomy Notes**	Hapalosiphonaceae-like cyanobacterium	**Notes on ecophysiological traits**	
**Accession Number(s)_16S**	HQ832910	**Isolator**	Vitor Ramos
**Accession Number(s)_others (product/gene /region)**	KC256764 (dinitrogenase reductase, nifH), KC813179 (non-ribosomal peptide synthetase), KC842313, KC842313 (polyketide synthase)	**Medium**	Z8 25‰ TM sea salt w/ vit. B12
**Collector**	Rui Seabra/Vitor Ramos	**Strain Status**	non-axenic, unicyanobacterial
**Collection Date**	2006/11/21	**Preservation type**	subculturing
**Location**	Portugal: Praia de São Bartolomeu do Mar, Esposende	**Light:dark cycle**	12:12-h
**Latitude & Longitude**	41.57377 N 8.798558 W	**Temperature (ºC)**	19
		**Light intensity**	10–30 µmol photons m−2 s−1
**References Publicly Available Articles**	Brito et al. (2012) Syst. Appl. Microbiol. 35, 110–119; Brito et al. (2015) Algal Res. 9, 218-226		
**References Publicly Available Theses**			

Strain ID	unidentified Nostocales LEGE 11386		
**Strain Taxonomy**			
**Microphotograph 400×**	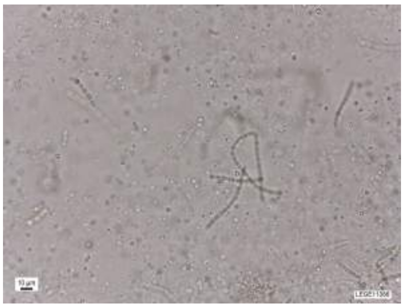	**Microphotograph 1000×**	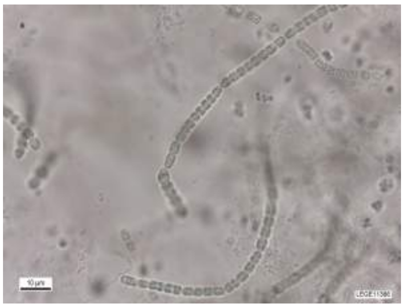
**Coidentity**	n.a.	**Morphometrics (Width × Length or Diameter) (µm)**	3,2±0,6 × 4,2±1,3
**Other Code(s)**	1MC	**Taxonomic notes/diacritical features**	
**Parent Sample**		**Macroscopic growth features**	
**Isolation Date**		**Environment**	aquatic, marine
**Order (Older Classification Scheme)**	Nostocales	**Habitat Sample Description**	intertidal zone, on a marine sponge
**Order 2014 (Modern Taxonomy)**	Nostocales	**Ecophysiological traits**	nitrogen-fixation
**Taxonomy Notes**		**Notes on ecophysiological traits**	
**Accession Number(s)_16S**	KU951877	**Isolator**	Ana Regueiras
**Accession Number(s)_others (product/gene /region)**		**Medium**	BG11o 25‰ TM sea salt w/ vit. B12
**Collector**	Ana Regueiras	**Strain Status**	non-axenic, unicyanobacterial
**Collection Date**	2011	**Preservation type**	subculturing
**Location**	Portugal	**Light:dark cycle**	12:12-h
**Latitude & Longitude**	unknown	**Temperature (ºC)**	19
		**Light intensity**	10–30 µmol photons m−2 s−1
**References Publicly Available Articles**			
**References Publicly Available Theses**	Vivas J. (2013) MSc Dissertation, FCUP, University of Porto, Porto, Portugal		

Strain ID	**unidentified Nostocales LEGE 12452**		
**Strain Taxonomy**			
**Microphotograph 400×**	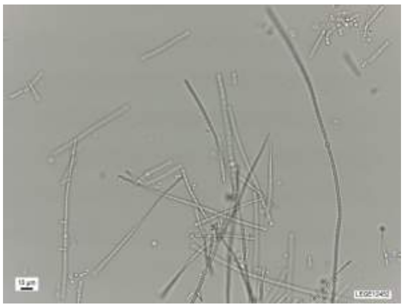	**Microphotograph 1000×**	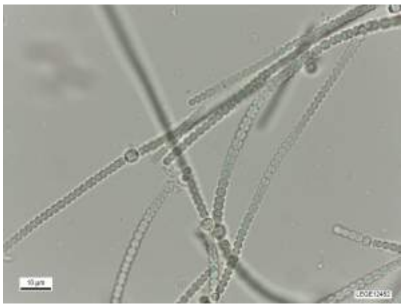
**Coidentity**	n.a.	**Morphometrics (Width × Length or Diameter) (µm)**	2,2±0,3 × 2,2±0,6 (Cells); Ø 3,3±1,7 (Heterocytes)
**Other Code(s)**	Ev1	**Taxonomic notes/diacritical features**	
**Parent Sample**		**Macroscopic growth features**	color: olive green; growing homogeneously; mucilaginous
**Isolation Date**		**Environment**	terrestrial
**Order (Older Classification Scheme)**	Nostocales	**Habitat Sample Description**	cycad coralloid root (host: Encephalartos villosus)
**Order 2014 (Modern Taxonomy)**	Nostocales	**Ecophysiological traits**	EPS production
**Taxonomy Notes**		**Notes on ecophysiological traits**	
**Accession Number(s)_16S**		**Isolator**	Ana L. Pereira
**Accession Number(s)_others (product/gene /region)**		**Medium**	BG11o
**Collector**	Ana L. Pereira	**Strain Status**	non-axenic, unicyanobacterial
**Collection Date**	2012	**Preservation type**	subculturing
**Location**	Portugal: Tropical Botanical Garden of Lisbon	**Light:dark cycle**	12:12-h
**Latitude & Longitude**	38.69819 N 9.203429 W	**Temperature (ºC)**	19
		**Light intensity**	10–30 µmol photons m−2 s−1
**References Publicly Available Articles**			
**References Publicly Available Theses**			

Strain ID	**unidentified Nostocales LEGE 12455**		
**Strain Taxonomy**			
**Microphotograph 400×**	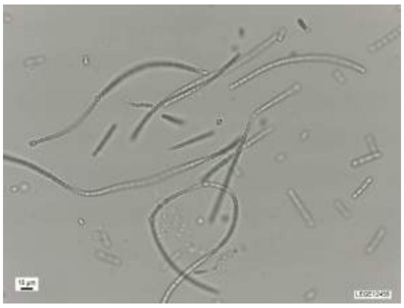	**Microphotograph 1000×**	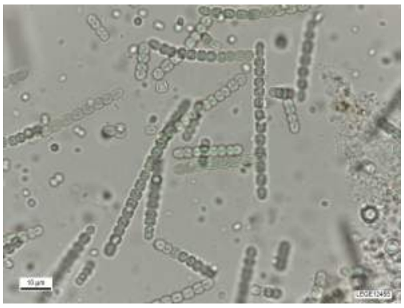
**Coidentity**	n.a.	**Morphometrics (Width × Length or Diameter) (µm)**	2,8±0,2 × 3,1±0,6 (Cells); Ø 3,8±0,5 (Heterocytes)
**Other Code(s)**	ELeh1	**Taxonomic notes/diacritical features**	
**Parent Sample**		**Macroscopic growth features**	color: olive green; forming aggregates
**Isolation Date**		**Environment**	terrestrial
**Order (Older Classification Scheme)**	Nostocales	**Habitat Sample Description**	cycad coralloid root (host: Encephalartos lehmannii)
**Order 2014 (Modern Taxonomy)**	Nostocales	**Ecophysiological traits**	
**Taxonomy Notes**		**Notes on ecophysiological traits**	
**Accession Number(s)_16S**		**Isolator**	Ana L. Pereira
**Accession Number(s)_others (product/gene /region)**		**Medium**	BG11o
**Collector**	Ana L. Pereira	**Strain Status**	non-axenic, unicyanobacterial
**Collection Date**	2012	**Preservation type**	subculturing
**Location**	Portugal: Tropical Botanical Garden of Lisbon	**Light:dark cycle**	12:12-h
**Latitude & Longitude**	38.69819 N 9.203429 W	**Temperature (ºC)**	19
		**Light intensity**	10–30 µmol photons m−2 s−1
**References Publicly Available Articles**			
**References Publicly Available Theses**			

**Strain ID**	**unidentified Nostocales LEGE 14445**		
**Strain Taxonomy**			
**Microphotograph 400×**	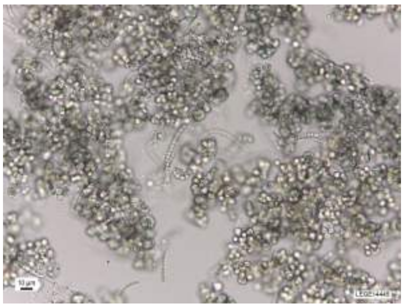	**Microphotograph 1000×**	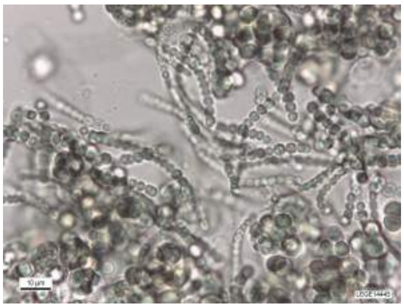
**Coidentity**	n.a.	**Morphometrics (Width × Length or Diameter) (µm)**	2,6±0,5 × 2,6±0,6 (Filaments); 4,0±0,9 (Spherical cells)
**Other Code(s)**		**Taxonomic notes/diacritical features**	life cycle showing a "pleurocapsalean" stage
**Parent Sample**	Col.1 HB	**Macroscopic growth features**	color: grayish-green; forming aggregates
**Isolation Date**		**Environment**	aquatic, freshwater
**Order (Older Classification Scheme)**	Nostocales	**Habitat Sample Description**	water sample
**Order 2014 (Modern Taxonomy)**	Nostocales	**Ecophysiological traits**	
**Taxonomy Notes**		**Notes on ecophysiological traits**	
**Accession Number(s)_16S**	KU951824	**Isolator**	Vitor Ramos
**Accession Number(s)_others (product/gene /region)**		**Medium**	Z8
**Collector**	Vitor Vasconcelos	**Strain Status**	non-axenic, unicyanobacterial
**Collection Date**	2014	**Preservation type**	subculturing, cryopreserved
**Location**	Colombia	**Light:dark cycle**	12:12-h
**Latitude & Longitude**	unknown	**Temperature (ºC)**	19
		**Light intensity**	10–30 µmol photons m−2 s−1
**References Publicly Available Articles**			
**References Publicly Available Theses**			

Strain ID	**unidentified Nostocales LEGE XX254**		
**Strain Taxonomy**			
**Microphotograph 400×**	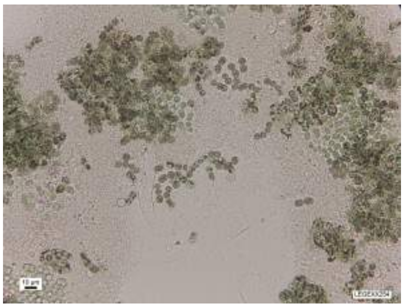	**Microphotograph 1000×**	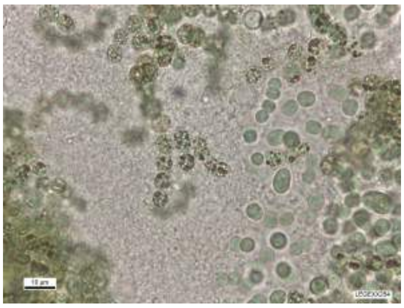
**Coidentity**	n.a.	**Morphometrics (Width × Length or Diameter) (µm)**	5,3±1,8 × 5,3±1,2 (cells); Ø 5,6±0,7 (Spherical Heterocytes)
**Other Code(s)**	J84, LEANJ.84	**Taxonomic notes/diacritical features**	highly coiled trichomes; vacuolated cells
**Parent Sample**		**Macroscopic growth features**	color: cyano; forming aggregates
**Isolation Date**		**Environment**	aquatic, freshwater
**Order (Older Classification Scheme)**	Nostocales	**Habitat Sample Description**	unknown
**Order 2014 (Modern Taxonomy)**	Nostocales	**Ecophysiological traits**	
**Taxonomy Notes**		**Notes on ecophysiological traits**	
**Accession Number(s)_16S**		**Isolator**	Joana Osswald
**Accession Number(s)_others (product/gene /region)**		**Medium**	Z8
**Collector**	Joana Osswald	**Strain Status**	non-axenic, unicyanobacterial
**Collection Date**	unknown	**Preservation type**	subculturing
**Location**	Portugal: Raia riverside, municipality of Moura	**Light:dark cycle**	12:12-h
**Latitude & Longitude**	unknown	**Temperature (ºC)**	19
		**Light intensity**	10–30 µmol photons m−2 s−1
**References Publicly Available Articles**			
**References Publicly Available Theses**			

**Strain ID**	**unidentified Nostocales LEGE XX276**		
**Strain Taxonomy**			
**Microphotograph 400×**	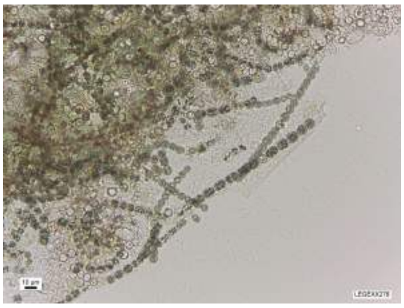	**Microphotograph 1000×**	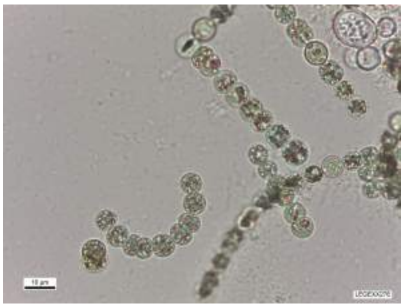
**Coidentity**	n.a.	**Morphometrics (Width × Length or Diameter) (µm)**	6,1±1,6 × 6,0±1,5 (Cells); Ø 6,0±0,7 (Heterocytes); 10,5±1,5 × 16±2,0 (Akinetes)
**Other Code(s)**	J89, LEANJ.89	**Taxonomic notes/diacritical features**	straight to coiled trichomes; vacuolated cells
**Parent Sample**		**Macroscopic growth features**	color: cyano; forming aggregates
**Isolation Date**		**Environment**	aquatic, freshwater
**Order (Older Classification Scheme)**	Nostocales	**Habitat Sample Description**	unknown
**Order 2014 (Modern Taxonomy)**	Nostocales	**Ecophysiological traits**	
**Taxonomy Notes**		**Notes on ecophysiological traits**	
**Accession Number(s)_16S**		**Isolator**	Joana Osswald
**Accession Number(s)_others (product/gene /region)**		**Medium**	Z8
**Collector**	Joana Osswald	**Strain Status**	non-axenic, unicyanobacterial
**Collection Date**	unknown	**Preservation type**	subculturing
**Location**	Portugal: Raia riverside, municipality of Moura	**Light:dark cycle**	12:12-h
**Latitude & Longitude**	unknown	**Temperature (ºC)**	19
		**Light intensity**	10–30 µmol photons m−2 s−1
**References Publicly Available Articles**			
**References Publicly Available Theses**			

Strain ID	unidentified Oscillatoriales LEGE 00049		
**Strain Taxonomy**			
**Microphotograph 400×**	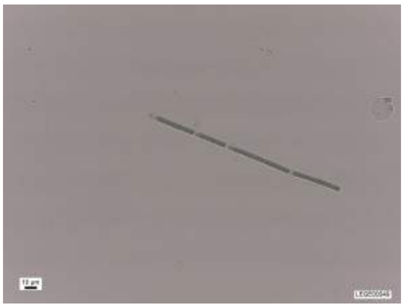	**Microphotograph 1000×**	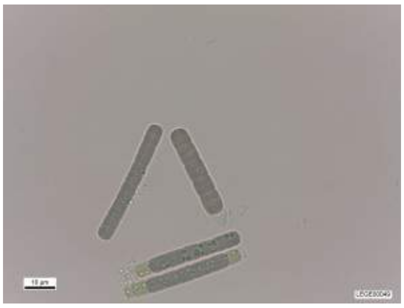
**Coidentity**	n.a.	**Morphometrics (Width × Length or Diameter) (µm)**	5,1±0,8 × 2,4±1,2
**Other Code(s)**		**Taxonomic notes/diacritical features**	trichomes sometimes bent toward ends; hormogonia; with sheath
**Parent Sample**	Alisir Osc	**Macroscopic growth features**	color: cyano; forming a firm biofilm and tuft-like aggregates
**Isolation Date**		**Environment**	aquatic, freshwater
**Order (Older Classification Scheme)**	Oscillatoriales	**Habitat Sample Description**	unknown
**Order 2014 (Modern Taxonomy)**	not determined	**Ecophysiological traits**	
**Taxonomy Notes**		**Notes on ecophysiological traits**	
**Accession Number(s)_16S**		**Isolator**	Martin Saker
**Accession Number(s)_others (product/gene /region)**		**Medium**	Z8
**Collector**	unknown	**Strain Status**	non-axenic, unicyanobacterial
**Collection Date**	2000	**Preservation type**	subculturing
**Location**	Morocco: Al Massira	**Light:dark cycle**	12:12-h
**Latitude & Longitude**	unknown	**Temperature (ºC)**	19
**References Publicly Available Articles**		**Light intensity**	10–30 µmol photons m−2 s−1
**References Publicly Available Theses**			

**Strain ID**	**unidentified Oscillatoriales LEGE 11385**		
**Strain Taxonomy**			
**Microphotograph 400×**	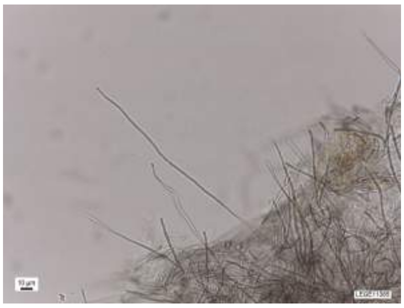	**Microphotograph 1000×**	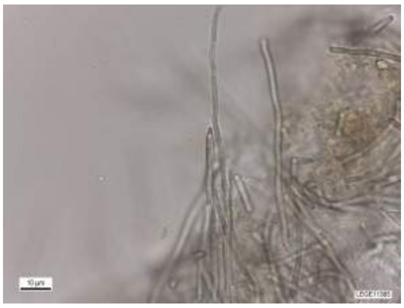
**Coidentity**	n.a.	**Morphometrics (Width × Length or Diameter) (µm)**	1,7±0,3 × 3,5±0,9
**Other Code(s)**	21D	**Taxonomic notes/diacritical features**	trichomes constricted at cross-walls, with their ends often distinctly hooked; distinguishable centro- and chromatoplasm; facultative sheath
**Parent Sample**		**Macroscopic growth features**	color: olive green
**Isolation Date**		**Environment**	aquatic, marine
**Order (Older Classification Scheme)**	Oscillatoriales	**Habitat Sample Description**	intertidal zone, on a marine sponge
**Order 2014 (Modern Taxonomy)**	not determined	**Ecophysiological traits**	
**Taxonomy Notes**		**Notes on ecophysiological traits**	
**Accession Number(s)_16S**		**Isolator**	Ana Regueiras
**Accession Number(s)_others (product/gene /region)**		**Medium**	Z8 25‰ TM sea salt w/ vit. B12
**Collector**	Ana Regueiras	**Strain Status**	non-axenic, unicyanobacterial
**Collection Date**	2011	**Preservation type**	subculturing
**Location**	Portugal	**Light:dark cycle**	12:12-h
**Latitude & Longitude**	unknown	**Temperature (ºC)**	19
		**Light intensity**	10–30 µmol photons m−2 s−1
**References Publicly Available Articles**			
**References Publicly Available Theses**			

**Strain ID**	**unidentified Oscillatoriales LEGE 11467**		
**Strain Taxonomy**			
**Microphotograph 400×**	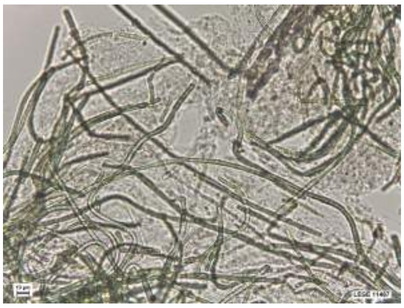	**Microphotograph 1000×**	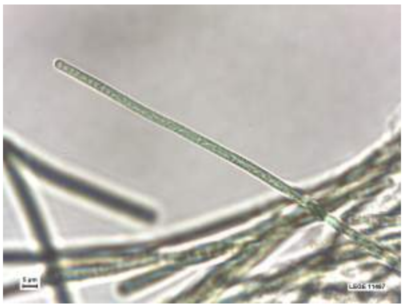
**Coidentity**	n.a.	**Morphometrics (Width × Length or Diameter) (µm)**	3,3±0,6 × 1,8±0,4
**Other Code(s)**		**Taxonomic notes/diacritical features**	straight trichomes
**Parent Sample**	8.003.1011	**Macroscopic growth features**	color: dark cyano; forming a firm biofilm
**Isolation Date**		**Environment**	aquatic, marine
**Order (Older Classification Scheme)**	Oscillatoriales	**Habitat Sample Description**	subtidal sample, epilithic (13m depth), about 200 m off the shore
**Order 2014 (Modern Taxonomy)**	Oscillatoriales	**Ecophysiological traits**	
**Taxonomy Notes**	Phormidium-like sp.	**Notes on ecophysiological traits**	
**Accession Number(s)_16S**	KU951878	**Isolator**	Sébastien Brûle/Ângela Pinheiro
**Accession Number(s)_others (product/gene /region)**		**Medium**	Z8 25‰ TM sea salt w/ vit. B12
**Collector**	Pedro Leão/Aldo Barreiro	**Strain Status**	non-axenic, unicyanobacterial
**Collection Date**	2011/10/03	**Preservation type**	subculturing
**Location**	Portugal: 'A Pedra', diving spot in front of the fort 'Castelo do Queijo'	**Light:dark cycle**	12:12-h
**Latitude & Longitude**	41.185809 N 8.719079 W	**Temperature (ºC)**	19
		**Light intensity**	10–30 µmol photons m−2 s−1
**References Publicly Available Articles**			
**References Publicly Available Theses**			

Strain ID	**unidentified Oscillatoriales LEGE 11472**		
**Strain Taxonomy**			
**Microphotograph 400×**	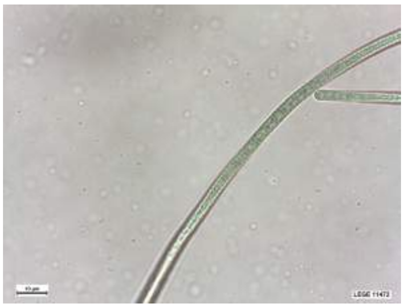	**Microphotograph 1000×**	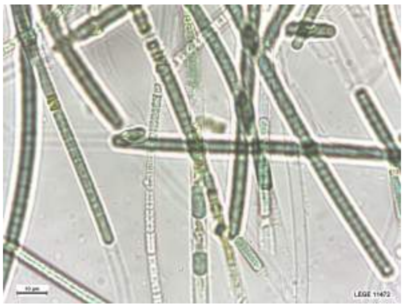
**Coidentity**	n.a.	**Morphometrics (Width × Length or Diameter) (µm)**	3,0±0,7 × 2,6±1,0
**Other Code(s)**		**Taxonomic notes/diacritical features**	keratomized chromatoplasma; with sheath
**Parent Sample**	8.003.1011	**Macroscopic growth features**	color: cyano-greenish; forming a firm/leather-like biofilm
**Isolation Date**		**Environment**	aquatic, marine
**Order (Older Classification Scheme)**	Oscillatoriales	**Habitat Sample Description**	subtidal sample, epilithic (13m depth), about 200 m off the shore
**Order 2014 (Modern Taxonomy)**	not determined	**Ecophysiological traits**	
**Taxonomy Notes**	Phormidium-like sp.	**Notes on ecophysiological traits**	
**Accession Number(s)_16S**		**Isolator**	Ângela Pinheiro
**Accession Number(s)_others (product/gene /region)**		**Medium**	Z8 25‰ TM sea salt w/ vit. B12
**Collector**	Pedro Leão/Aldo Barreiro	**Strain Status**	non-axenic, unicyanobacterial
**Collection Date**	2011/10/03	**Preservation type**	subculturing
**Location**	Portugal: 'A Pedra', diving spot in front of the fort 'Castelo do Queijo'	**Light:dark cycle**	12:12-h
**Latitude & Longitude**	41.185809 N 8.719079 W	**Temperature (ºC)**	19
		**Light intensity**	10–30 µmol photons m−2 s−1
**References Publicly Available Articles**			
**References Publicly Available Theses**			

**Strain ID**	**unidentified Pleurocapsales LEGE 06147**		
**Strain Taxonomy**			
**Microphotograph 400×**	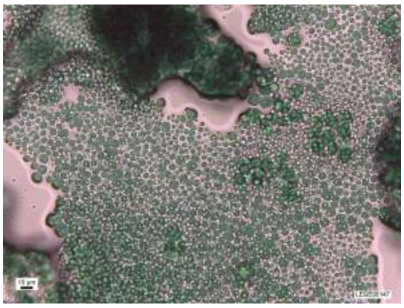	**Microphotograph 1000×**	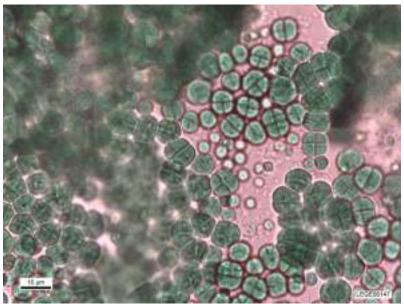
**Coidentity**	n.a.	**Morphometrics (Width × Length or Diameter) (µm)**	Ø 3,6±0,4 (Cells); Ø 7,7±0,8 (Group of 4 Cells)
**Other Code(s)**	LEAN 060	**Taxonomic notes/diacritical features**	colonial; with baeocytes
**Parent Sample**	O.06 MOL 14 - C	**Macroscopic growth features**	color: cyano; forming a firm biofilm and also aggregates
**Isolation Date**		**Environment**	aquatic, marine
**Order (Older Classification Scheme)**	Pleurocapsales	**Habitat Sample Description**	intertidal zone, wave-exposed rock surface scraping
**Order 2014 (Modern Taxonomy)**	Pleurocapsales	**Ecophysiological traits**	
**Taxonomy Notes**		**Notes on ecophysiological traits**	
**Accession Number(s)_16S**	KU951879	**Isolator**	Vitor Ramos
**Accession Number(s)_others (product/gene /region)**		**Medium**	Z8 25‰ TM sea salt w/ vit. B12
**Collector**	Rui Seabra/Vitor Ramos	**Strain Status**	non-axenic, unicyanobacterial
**Collection Date**	2006/11/20	**Preservation type**	subculturing
**Location**	Portugal: Praia de Moledo, Caminha	**Light:dark cycle**	12:12-h
**Latitude & Longitude**	41.84963 N 8.866717 W	**Temperature (ºC)**	19
		**Light intensity**	10–30 µmol photons m−2 s−1
**References Publicly Available Articles**	Rastoll et al. (2013) J. Appl. Phycol. 25, 1483-1493		
**References Publicly Available Theses**			

Strain ID	unidentified Pleurocapsales LEGE 10410		
**Strain Taxonomy**			
**Microphotograph 400×**	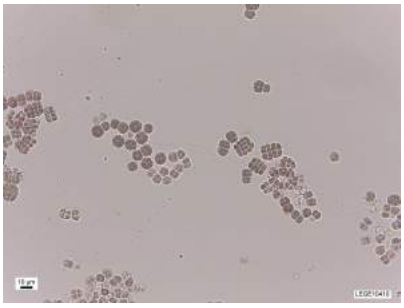	**Microphotograph 1000×**	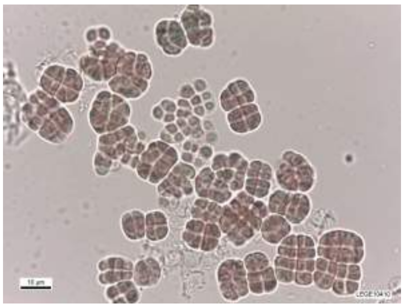
**Coidentity**	n.a.	**Morphometrics (Width × Length or Diameter) (µm)**	Ø 2,1±0,4 (more or less spherical cells); Ø 12,0±2,2 (Clusters of 14-16 cells)
**Other Code(s)**		**Taxonomic notes/diacritical features**	
**Parent Sample**	22T-2AAA	**Macroscopic growth features**	color: brown-reddish; forming aggregates
**Isolation Date**		**Environment**	aquatic, marine
**Order (Older Classification Scheme)**	Pleurocapsales	**Habitat Sample Description**	intertidal zone, green biofilm on a pebblestone
**Order 2014 (Modern Taxonomy)**	Pleurocapsales	**Ecophysiological traits**	
**Taxonomy Notes**		**Notes on ecophysiological traits**	
**Accession Number(s)_16S**	KU951880	**Isolator**	Vitor Ramos
**Accession Number(s)_others (product/gene /region)**		**Medium**	MN w/ vit. B12
**Collector**	Vitor Ramos	**Strain Status**	non-axenic, unicyanobacterial
**Collection Date**	2010/04/28	**Preservation type**	subculturing
**Location**	Portugal: Vila Nova de Mil Fontes	**Light:dark cycle**	12:12-h
**Latitude & Longitude**	37.719139 N 8.791014 W	**Temperature (ºC)**	19
		**Light intensity**	10–30 µmol photons m−2 s−1
**References Publicly Available Articles**			
**References Publicly Available Theses**	Afonso T. (2014) MSc Dissertation, FCUP, University of Porto, Porto, Portugal		

Strain ID	unidentified Pseudanabaenaceae cyanobacterium LEGE 06112		
**Strain Taxonomy**			
**Microphotograph 400×**	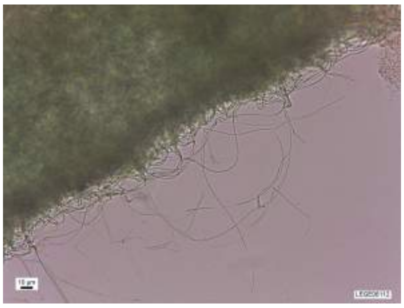	**Microphotograph 1000×**	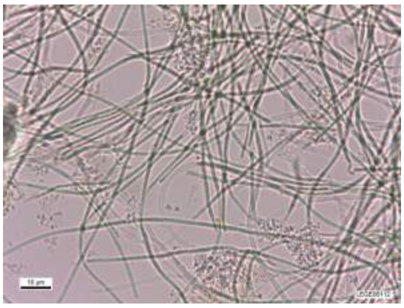
**Coidentity**	n.a.	**Morphometrics (Width × Length or Diameter) (µm)**	1,2±0,2 x 3,5±1,0
**Other Code(s)**	LEAN 017	**Taxonomic notes/diacritical features**	hardly distinguished cell walls; facultative sheath
**Parent Sample**	O.06 MAR 5 - GA	**Macroscopic growth features**	color: cyano; forming a firm biofilm
**Isolation Date**		**Environment**	aquatic, marine
**Order (Older Classification Scheme)**	Oscillatoriales	**Habitat Sample Description**	wave-exposed tide pool, rock surface
**Order 2014 (Modern Taxonomy)**	not determined	**Ecophysiological traits**	
**Taxonomy Notes**		**Notes on ecophysiological traits**	
**Accession Number(s)_16S**		**Isolator**	Vitor Ramos
**Accession Number(s)_others (product/gene /region)**		**Medium**	MN w/ vit. B12
**Collector**	Rui Seabra	**Strain Status**	non-axenic, unicyanobacterial
**Collection Date**	2006/12/11	**Preservation type**	subculturing
**Location**	Portugal: Praia do Martinhal, Vila do Bispo	**Light:dark cycle**	12:12-h
**Latitude & Longitude**	37.01869 N 8.926714 W	**Temperature (ºC)**	19
		**Light intensity**	10–30 µmol photons m−2 s−1
**References Publicly Available Articles**			
**References Publicly Available Theses**			

Strain ID	unidentified Pseudanabaenaceae cyanobacterium LEGE 13415		
**Strain Taxonomy**			
**Microphotograph 400×**	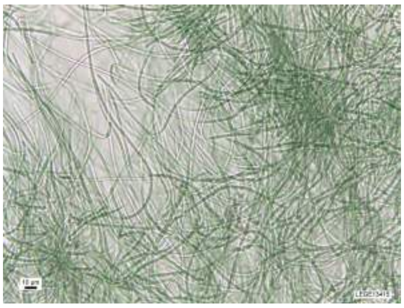	**Microphotograph 1000×**	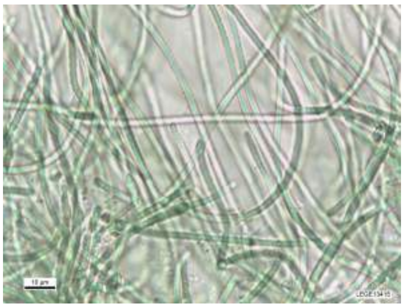
**Coidentity**	n.a.	**Morphometrics (Width × Length or Diameter) (µm)**	1,9±0,2 × 1,6±0,3
**Other Code(s)**	C1F	**Taxonomic notes/diacritical features**	
**Parent Sample**		**Macroscopic growth features**	color: cyano; forming a smooth biofilm
**Isolation Date**		**Environment**	unknown
**Order (Older Classification Scheme)**	Oscillatoriales	**Habitat Sample Description**	"Chlorella" dietary supplement, powder form. Producer/brand: Iswari
**Order 2014 (Modern Taxonomy)**	not determined	**Ecophysiological traits**	
**Taxonomy Notes**	Leptolyngbya-like sp.	**Notes on ecophysiological traits**	
**Accession Number(s)_16S**		**Isolator**	Dina Gomes
**Accession Number(s)_others (product/gene /region)**		**Medium**	Z8
**Collector**	Dina Gomes	**Strain Status**	non-axenic, unicyanobacterial
**Collection Date**	2013	**Preservation type**	subculturing
**Location**	original source unknown: acquired in the Porto metropolitan area, Portugal	**Light:dark cycle**	12:12-h
**Latitude & Longitude**	unknown	**Temperature (ºC)**	19
		**Light intensity**	10–30 µmol photons m−2 s−1
**References Publicly Available Articles**			
**References Publicly Available Theses**	Gomes D. (2013) MSc Dissertation, ICBAS, University of Porto, Porto, Portugal		

Strain ID	**unidentified colonial Chroococcidiopsidales LEGE 13417**		
**Strain Taxonomy**			
**Microphotograph 400×**	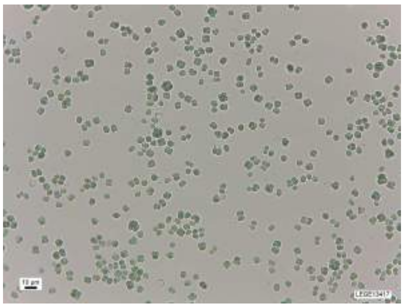	**Microphotograph 1000×**	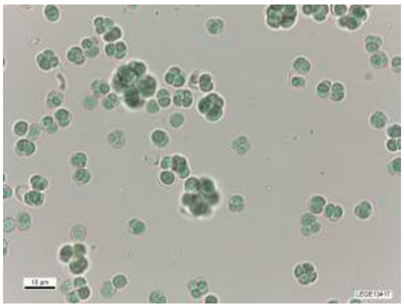
**Coidentity**	n.a.	**Morphometrics (Width × Length or Diameter) (µm)**	Ø 3,4±0,6 (Cells); Ø 5,1±0,8 (Group of 4 Cells)
**Other Code(s)**	C4C	**Taxonomic notes/diacritical features**	with sheath
**Parent Sample**		**Macroscopic growth features**	color: cyano; forming a smooth biofilm and aggregates
**Isolation Date**		**Environment**	unknown
**Order (Older Classification Scheme)**	Chroococcales	**Habitat Sample Description**	"Chlorella" dietary supplement, tablet form. Producer/brand: Be-Life
**Order 2014 (Modern Taxonomy)**	Chroococcidiopsidales	**Ecophysiological traits**	
**Taxonomy Notes**	Chroococcus-like sp.	**Notes on ecophysiological traits**	
**Accession Number(s)_16S**	KU951843	**Isolator**	Dina Gomes
**Accession Number(s)_others (product/gene /region)**		**Medium**	Z8
**Collector**	Dina Gomes	**Strain Status**	non-axenic, unicyanobacterial
**Collection Date**	2013	**Preservation type**	subculturing
**Location**	original source unknown: acquired in the Porto metropolitan area, Portugal	**Light:dark cycle**	12:12-h
**Latitude & Longitude**	unknown	**Temperature (ºC)**	19
		**Light intensity**	10–30 µmol photons m−2 s−1
**References Publicly Available Articles**			
**References Publicly Available Theses**	Gomes D. (2013) MSc Dissertation, ICBAS, University of Porto, Porto, Portugal		

Strain ID	**unidentified colonial Chroococcidiopsidales LEGE 13419**		
**Strain Taxonomy**			
**Microphotograph 400×**	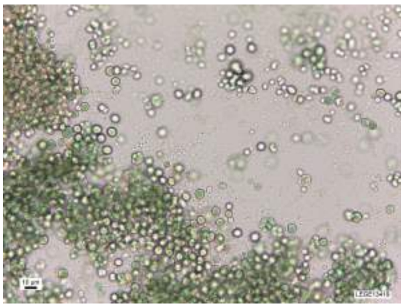	**Microphotograph 1000×**	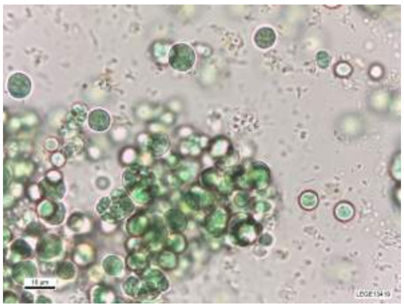
**Coidentity**	n.a.	**Morphometrics (Width × Length or Diameter) (µm)**	Ø 5,9±1,3
**Other Code(s)**	S1C	**Taxonomic notes/diacritical features**	baeocytes observed; with sheath
**Parent Sample**		**Macroscopic growth features**	color: cyano; forming aggregates
**Isolation Date**		**Environment**	unknown
**Order (Older Classification Scheme)**	Chroococcales	**Habitat Sample Description**	"Spirulina" dietary supplements, powder form. Producer/brand: Iswari
**Order 2014 (Modern Taxonomy)**	Chroococcidiopsidales	**Ecophysiological traits**	
**Taxonomy Notes**	Gloeocapsa-like sp.	**Notes on ecophysiological traits**	
**Accession Number(s)_16S**	KU951844	**Isolator**	Dina Gomes
**Accession Number(s)_others (product/gene /region)**		**Medium**	Z8
**Collector**	Dina Gomes	**Strain Status**	non-axenic, unicyanobacterial
**Collection Date**	2013	**Preservation type**	subculturing
**Location**	original source unknown: acquired in the Porto metropolitan area, Portugal	**Light:dark cycle**	12:12-h
**Latitude & Longitude**	unknown	**Temperature (ºC)**	19
		**Light intensity**	10–30 µmol photons m−2 s−1
**References Publicly Available Articles**			
**References Publicly Available Theses**	Gomes D. (2013) MSc Dissertation, ICBAS, University of Porto, Porto, Portugal		

Strain ID	**unidentified colonial Chroococcidiopsidales LEGE 13423**		
**Strain Taxonomy**			
**Microphotograph 400×**	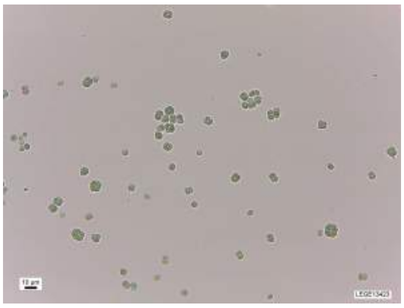	**Microphotograph 1000×**	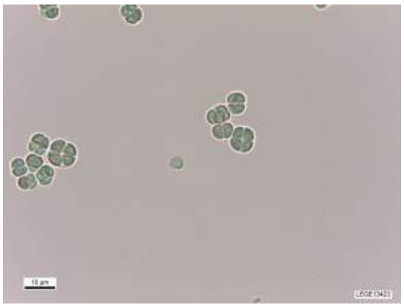
**Coidentity**	n.a.	**Morphometrics (Width × Length or Diameter) (µm)**	Ø 3,6±0,6 (more or less spherical cells)
**Other Code(s)**	S6C	**Taxonomic notes/diacritical features**	with sheath
**Parent Sample**		**Macroscopic growth features**	color: cyano; forming aggregates
**Isolation Date**		**Environment**	unknown
**Order (Older Classification Scheme)**	Chroococcales	**Habitat Sample Description**	"Spirulina" dietary supplements, capsules form. Producer/brand: Solaray
**Order 2014 (Modern Taxonomy)**	Chroococcidiopsidales	**Ecophysiological traits**	
**Taxonomy Notes**	Gloeocapsopsis-like sp.	**Notes on ecophysiological traits**	
**Accession Number(s)_16S**	KU951845	**Isolator**	Dina Gomes
**Accession Number(s)_others (product/gene /region)**		**Medium**	Z8
**Collector**	Dina Gomes	**Strain Status**	non-axenic, unicyanobacterial
**Collection Date**	2013	**Preservation type**	subculturing
**Location**	original source unknown: acquired in the Porto metropolitan area, Portugal	**Light:dark cycle**	12:12-h
**Latitude & Longitude**	unknown	**Temperature (ºC)**	19
		**Light intensity**	10–30 µmol photons m−2 s−1
**References Publicly Available Articles**			
**References Publicly Available Theses**	Gomes D. (2013) MSc Dissertation, ICBAS, University of Porto, Porto, Portugal		

Strain ID	unidentified colonial Synechococcales LEGE 06192		
**Strain Taxonomy**			
**Microphotograph 400×**	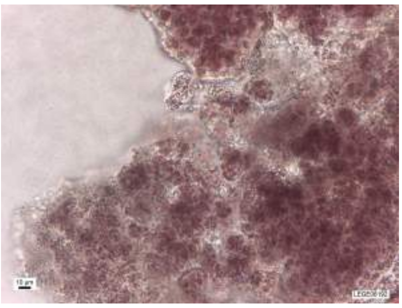	**Microphotograph 1000×**	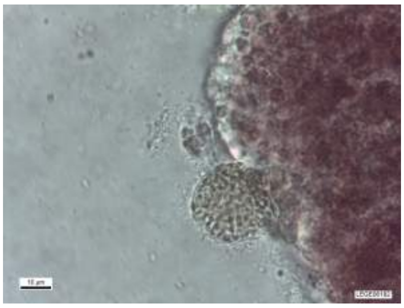
**Coidentity**	n.a.	**Morphometrics (Width × Length or Diameter) (µm)**	Ø 1,3±0,2 (Spherical Cells)
**Other Code(s)**	LEAN 157	**Taxonomic notes/diacritical features**	mucilaginous clusters of cells
**Parent Sample**	O.06 BUR p4 - AA	**Macroscopic growth features**	color: brown-reddish; forming aggregates/colonies
**Isolation Date**	2007	**Environment**	aquatic, marine
**Order (Older Classification Scheme)**	Chroococcales	**Habitat Sample Description**	coastal sea water, surf zone
**Order 2014 (Modern Taxonomy)**	Synechococcales	**Ecophysiological traits**	EPS production
**Taxonomy Notes**		**Notes on ecophysiological traits**	capsules
**Accession Number(s)_16S**	KC249951	**Isolator**	Vitor Ramos
**Accession Number(s)_others (product/gene /region)**		**Medium**	MN w/ vit. B12
**Collector**	Rui Seabra	**Strain Status**	non-axenic, unicyanobacterial
**Collection Date**	2006/12/11	**Preservation type**	subculturing
**Location**	Portugal: Praia de Burgau, Budens	**Light:dark cycle**	12:12-h
**Latitude & Longitude**	37.07156 N 8.774722 W	**Temperature (ºC)**	19
		**Light intensity**	10–30 µmol photons m−2 s−1
**References Publicly Available Articles**	Leão et al. (2013) Mar. Drugs 11 (4), 1316-1335; Rastoll et al. (2013) J. Appl. Phycol. 25, 1483-1493; Dvořák et al. (2014) Mol. Ecol. 23, 5538–5551		
**References Publicly Available Theses**			

Strain ID	**unidentified colonial Synechococcales LEGE 10388**		
**Strain Taxonomy**			
**Microphotograph 400×**	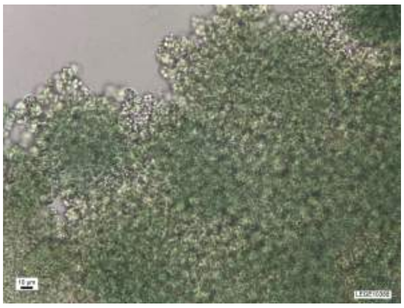	**Microphotograph 1000×**	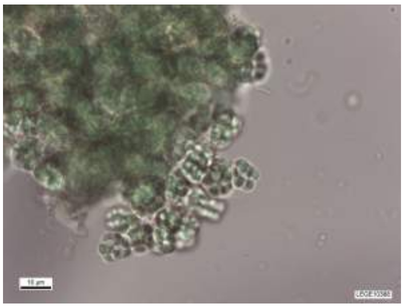
**Coidentity**	n.a.	**Morphometrics (Width × Length or Diameter) (µm)**	Ø 1,8±0,4 (more or less spherical cells)
**Other Code(s)**		**Taxonomic notes/diacritical features**	distinguishable centro- and chromatoplasm; clusters of less than 16 cells
**Parent Sample**	27C-1-B	**Macroscopic growth features**	color: cyano; forming aggregates
**Isolation Date**		**Environment**	aquatic, marine
**Order (Older Classification Scheme)**	Chroococcales	**Habitat Sample Description**	intertidal zone, from a dark mat, on a wave exposed rock
**Order 2014 (Modern Taxonomy)**	Synechococcales	**Ecophysiological traits**	
**Taxonomy Notes**	Gloeothece-like sp.	**Notes on ecophysiological traits**	dehydroabietic acid production (unknown ecophysiological role)
**Accession Number(s)_16S**	New Seq + KT951669	**Isolator**	Vitor Ramos
**Accession Number(s)_others (product/gene /region)**		**Medium**	MN w/ vit. B12
**Collector**	Vitor Ramos	**Strain Status**	non-axenic, unicyanobacterial
**Collection Date**	2010/04/28	**Preservation type**	subculturing
**Location**	Portugal: Vila Nova de Mil Fontes	**Light:dark cycle**	12:12-h
**Latitude & Longitude**	37.719139 N 8.791014 W	**Temperature (ºC)**	19
		**Light intensity**	10–30 µmol photons m−2 s−1
**References Publicly Available Articles**	Costa et al. (2016) Sci. Rep. 6, 23436		
**References Publicly Available Theses**			

Strain ID	unidentified colonial Synechococcales LEGE 10400		
Strain Taxonomy			
Microphotograph 400×	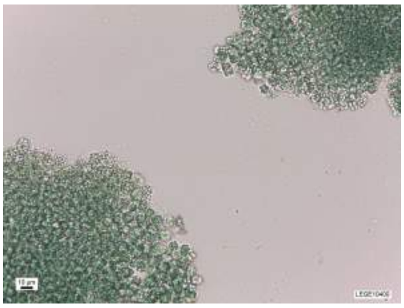	Microphotograph 1000×	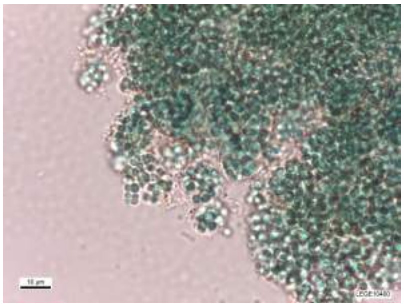
Coidentity	n.a.	Morphometrics (Width × Length or Diameter) (µm)	Ø 1,7±0,4 (more or less spherical cells)
Other Code(s)		Taxonomic notes/diacritical features	forming nanocytes?; with sheath
Parent Sample	57-AAAA	Macroscopic growth features	color: cyano; forming aggregates
Isolation Date		Environment	aquatic, marine
Order (Older Classification Scheme)	Chroococcales	Habitat Sample Description	intertidal zone, from a brown mat
Order 2014 (Modern Taxonomy)	Synechococcales	Ecophysiological traits	
Taxonomy Notes		Notes on ecophysiological traits	
Accession Number(s)_16S	KU951846	Isolator	Vitor Ramos
Accession Number(s)_others (product/gene /region)		Medium	MN w/ vit. B12
Collector	Vitor Ramos	Strain Status	non-axenic, unicyanobacterial
Collection Date	2010/07/14	Preservation type	subculturing
Location	Portugal: São Martinho do Porto bay, near the entrance of the sea	Light:dark cycle	12:12-h
Latitude & Longitude	39.51130 N 9.141137 W	Temperature (ºC)	19
		Light intensity	10–30 µmol photons m−2 s−1
References Publicly Available Articles			
References Publicly Available Theses			

**Strain ID**	**unidentified filamentous Chroococcales LEGE 11427**		
**Strain Taxonomy**			
**Microphotograph 400×**	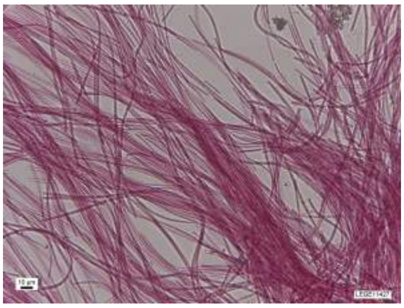	**Microphotograph 1000×**	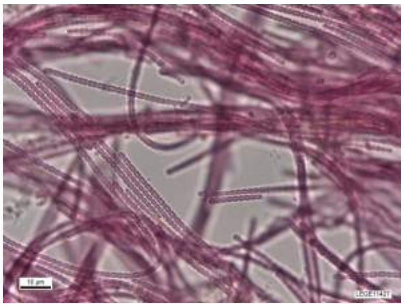
**Coidentity**	n.a.	**Morphometrics (Width × Length or Diameter) (µm)**	1,8±0,2 × 1,8±0,3
**Other Code(s)**		**Taxonomic notes/diacritical features**	trichomes constricted at cross-walls
**Parent Sample**	1.002.0911_AP.I(1)A-1	**Macroscopic growth features**	color: reddish; forming a firm biofilm
**Isolation Date**		**Environment**	aquatic, marine
**Order (Older Classification Scheme)**	Oscillatoriales	**Habitat Sample Description**	subtidal sample, epilithic (10-13m depth), less than 1 km off the shore
**Order 2014 (Modern Taxonomy)**	Chroococcales	**Ecophysiological traits**	
**Taxonomy Notes**	Pseudanabaenaceae cyanobacterium	**Notes on ecophysiological traits**	
**Accession Number(s)_16S**	KU951847	**Isolator**	Ângela Pinheiro
**Accession Number(s)_others (product/gene /region)**		**Medium**	Z8 25‰ TM sea salt w/ vit. B12
**Collector**	Pedro Leão/Aldo Barreiro	**Strain Status**	non-axenic, unicyanobacterial
**Collection Date**	2011/09/26	**Preservation type**	subculturing
**Location**	Portugal: 'Pêlo Negro', diving spot near Leixões Harbour	**Light:dark cycle**	12:12-h
**Latitude & Longitude**	41.18557 N 8.719100 W	**Temperature (ºC)**	19
		**Light intensity**	5 µmol photons m−2 s−1
**References Publicly Available Articles**			
**References Publicly Available Theses**			

Strain ID	**unidentified filamentous Chroococcales LEGE 11438**		
**Strain Taxonomy**			
**Microphotograph 400×**	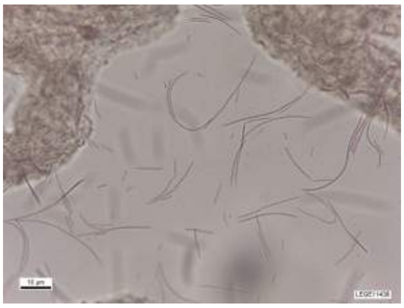	**Microphotograph 1000×**	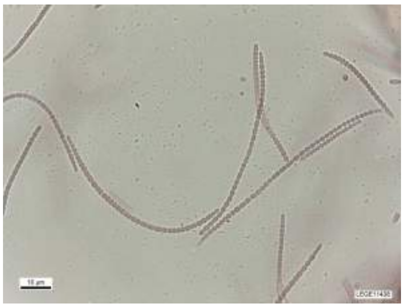
**Coidentity**	n.a.	**Morphometrics (Width × Length or Diameter) (µm)**	1,4±0,1 × 1,4±0,3
**Other Code(s)**		**Taxonomic notes/diacritical features**	gliding motility; conical-rounded terminal cells; trichomes constricted at cross-walls
**Parent Sample**	1.001.09.11 MN a	**Macroscopic growth features**	color: brownish; forming aggregates
**Isolation Date**		**Environment**	aquatic, marine
**Order (Older Classification Scheme)**	Oscillatoriales	**Habitat Sample Description**	subtidal sample, epilithic (6 m depth), near the south quay of the Leixões harbour
**Order 2014 (Modern Taxonomy)**	Chroococcales	**Ecophysiological traits**	
**Taxonomy Notes**		**Notes on ecophysiological traits**	
**Accession Number(s)_16S**	KU951848	**Isolator**	Sofia Costa
**Accession Number(s)_others (product/gene /region)**		**Medium**	MN w/ vit. B12
**Collector**	Pedro Leão/Aldo Barreiro	**Strain Status**	non-axenic, unicyanobacterial
**Collection Date**	2011/09/26	**Preservation type**	subculturing
**Location**	Portugal: at [115], diving spot near Leixões Harbour	**Light:dark cycle**	12:12-h
**Latitude & Longitude**	41.17652 N 8.700633 W	**Temperature (ºC)**	19
		**Light intensity**	5 µmol photons m−2 s−1
**References Publicly Available Articles**			
**References Publicly Available Theses**			

Strain ID	**unidentified filamentous Synechococcales LEGE 00039**		
**Strain Taxonomy**			
**Microphotograph 400×**	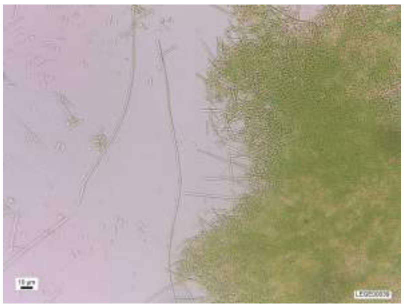	**Microphotograph 1000×**	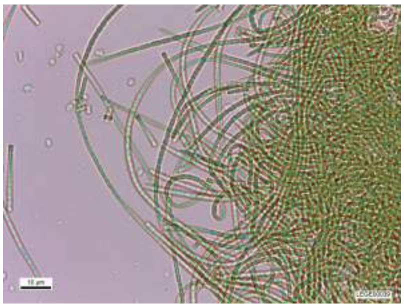
**Coidentity**	n.a.	**Morphometrics (Width × Length or Diameter) (µm)**	1,4±0,2 × 2,0±0,4
**Other Code(s)**	strain 14, LEANCYA 14, LEAN 213	**Taxonomic notes/diacritical features**	
**Parent Sample**	Aguda 6	**Macroscopic growth features**	color: cyano; growing homogeneously; mucilaginous
**Isolation Date**		**Environment**	aquatic, marine
**Order (Older Classification Scheme)**	Oscillatoriales	**Habitat Sample Description**	intertidal zone, on a Patella sp. shell
**Order 2014 (Modern Taxonomy)**	Synechococcales	**Ecophysiological traits**	EPS production
**Taxonomy Notes**		**Notes on ecophysiological traits**	
**Accession Number(s)_16S**	KU951863	**Isolator**	Rosario Martins
**Accession Number(s)_others (product/gene /region)**		**Medium**	Z8 25‰ TM sea salt w/ vit. B12
**Collector**	Rosario Martins	**Strain Status**	non-axenic, unicyanobacterial
**Collection Date**	2000/05/31	**Preservation type**	subculturing
**Location**	Portugal: Praia da Aguda, Arcozelo	**Light:dark cycle**	12:12-h
**Latitude & Longitude**	41.04954 N 8.655339 W	**Temperature (ºC)**	19
		**Light intensity**	10–30 µmol photons m−2 s−1
**References Publicly Available Articles**	Martins et al. (2005) Toxicon 46, 454–464		
**References Publicly Available Theses**	Martins R. (2005) PhD Thesis, FCUP, University of Porto, Porto, Portugal		

Strain ID	**unidentified filamentous Synechococcales LEGE 00052**		
**Strain Taxonomy**			
**Microphotograph 400×**	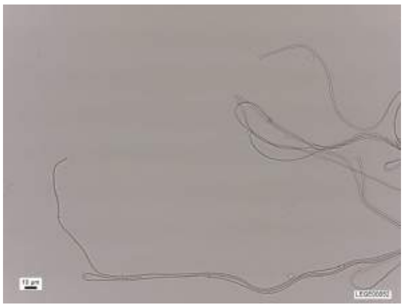	**Microphotograph 1000×**	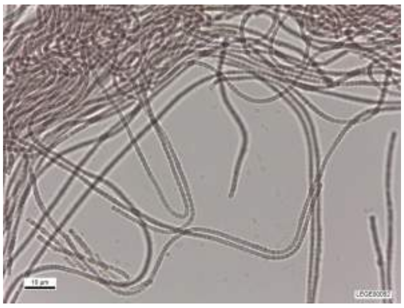
**Coidentity**	n.a.	**Morphometrics (Width × Length or Diameter) (µm)**	1,3±0,2 × 1,4±0,2
**Other Code(s)**		**Taxonomic notes/diacritical features**	trichomes slightly constricted at cross-walls
**Parent Sample**	EDDAH Osc I	**Macroscopic growth features**	color: olive-green; forming aggregates
**Isolation Date**		**Environment**	aquatic, freshwater
**Order (Older Classification Scheme)**	Oscillatoriales	**Habitat Sample Description**	unknown
**Order 2014 (Modern Taxonomy)**	Synechococcales	**Ecophysiological traits**	
**Taxonomy Notes**	Pseudanabaenaceae cyanobacterium	**Notes on ecophysiological traits**	
**Accession Number(s)_16S**	KU951850	**Isolator**	Martin Saker
**Accession Number(s)_others (product/gene /region)**		**Medium**	Z8
**Collector**	unknown	**Strain Status**	non-axenic, unicyanobacterial
**Collection Date**	2000	**Preservation type**	subculturing
**Location**	Morocco: Oued Mellah	**Light:dark cycle**	12:12-h
**Latitude & Longitude**	unknown	**Temperature (ºC)**	19
		**Light intensity**	10–30 µmol photons m−2 s−1
**References Publicly Available Articles**	Leão et al. (2009) Eur. J. Phycol. 44, 347-355		
**References Publicly Available Theses**	Leão P.N. (2010) PhD Thesis, FCUP, University of Porto, Porto, Portugal		

Strain ID	**unidentified filamentous Synechococcales LEGE 00053**		
**Strain Taxonomy**			
**Microphotograph 400×**	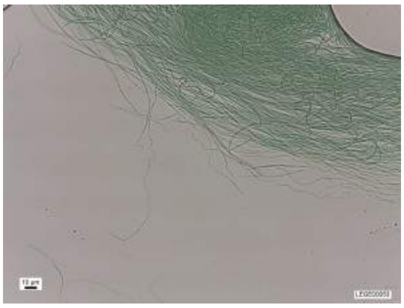	**Microphotograph 1000×**	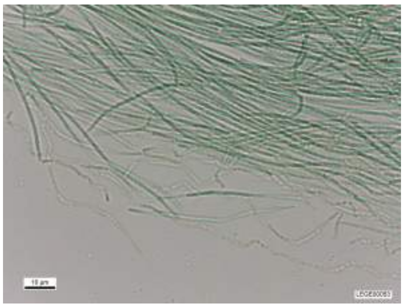
**Coidentity**	n.a.	**Morphometrics (Width × Length or Diameter) (µm)**	1,2±0,3 × 1,7±0,6
**Other Code(s)**		**Taxonomic notes/diacritical features**	
**Parent Sample**	EDDAH Osc II	**Macroscopic growth features**	color: cyano; growing homogeneously, but also forming aggregates
**Isolation Date**		**Environment**	aquatic, freshwater
**Order (Older Classification Scheme)**	Oscillatoriales	**Habitat Sample Description**	unknown
**Order 2014 (Modern Taxonomy)**	Synechococcales	**Ecophysiological traits**	
**Taxonomy Notes**	Pseudanabaenaceae cyanobacterium	**Notes on ecophysiological traits**	
**Accession Number(s)_16S**	KU951864	**Isolator**	Martin Saker
**Accession Number(s)_others (product/gene /region)**		**Medium**	Z8
**Collector**	unknown	**Strain Status**	non-axenic, unicyanobacterial
**Collection Date**	2000	**Preservation type**	subculturing
**Location**	Morocco: Oued Mellah	**Light:dark cycle**	12:12-h
**Latitude & Longitude**	unknown	**Temperature (ºC)**	19
		**Light intensity**	10–30 µmol photons m−2 s−1
**References Publicly Available Articles**	Leão et al. (2009) Eur. J. Phycol. 44, 347-355		
**References Publicly Available Theses**	Leão P.N. (2010) PhD Thesis, FCUP, University of Porto, Porto, Portugal		

Strain ID	unidentified filamentous Synechococcales LEGE 06018		
Strain Taxonomy			
Microphotograph 400×	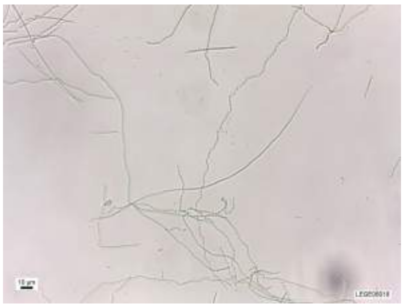	Microphotograph 1000×	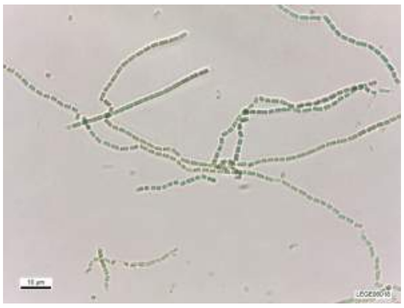
Coidentity	n.a.	Morphometrics (Width × Length or Diameter) (µm)	1,5±0,2 × 2,5±0,4
Other Code(s)	strain 14, LEAN 105	Taxonomic notes/diacritical features	
Parent Sample	Ref. 14	Macroscopic growth features	color: cyano; forming a smooth biofilm; mucilaginous
Isolation Date		Environment	aquatic, marine
Order (Older Classification Scheme)	Oscillatoriales	Habitat Sample Description	intertidal zone, wave-exposed rock
Order 2014 (Modern Taxonomy)	Synechococcales	Ecophysiological traits	EPS production
Taxonomy Notes		Notes on ecophysiological traits	
Accession Number(s)_16S	HM124562	Isolator	Bárbara Frazão
Accession Number(s)_others (product/gene /region)	HM124566 (microcystin synthetase, mcyE)	Medium	Z8 25‰ TM sea salt w/ vit. B12
Collector	Bárbara Frazão	Strain Status	non-axenic, unicyanobacterial
Collection Date	Fall 2006	Preservation type	subculturing, cryopreserved
Location	Portugal: Praia dos Coxos, Sto. Isidoro	Light:dark cycle	12:12-h
Latitude & Longitude	39.00455 N 9.425842 W	Temperature (ºC)	19
		Light intensity	10–30 µmol photons m−2 s−1
References Publicly Available Articles	Frazão et al. (2010) Mar. Drugs 8, 1908-1919; Rastoll et al. (2013) J. Appl. Phycol. 25, 1483-1493		
References Publicly Available Theses	Frazão B. (2008) MSc Dissertation, FCUP, University of Porto, Porto, Portugal		

**Strain ID**	**unidentified filamentous Synechococcales LEGE 06021**		
**Strain Taxonomy**			
**Microphotograph 400×**	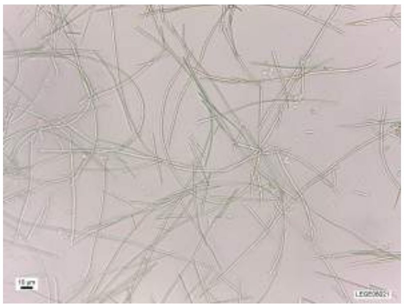	**Microphotograph 1000×**	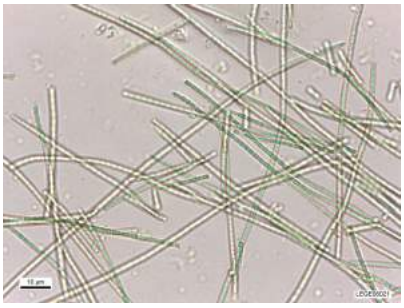
**Coidentity**	n.a.	**Morphometrics (Width × Length or Diameter) (µm)**	1,4±0,1 × 2,1±0,4
**Other Code(s)**	strain 18, LEAN 119	**Taxonomic notes/diacritical features**	
**Parent Sample**	Ref. 18, 2 Coxos	**Macroscopic growth features**	color: cyano; forming a smooth biofilm; mucilaginous
**Isolation Date**		**Environment**	aquatic, marine
**Order (Older Classification Scheme)**	Oscillatoriales	**Habitat Sample Description**	intertidal zone, wave-exposed rock
**Order 2014 (Modern Taxonomy)**	Synechococcales	**Ecophysiological traits**	EPS production
**Taxonomy Notes**	Pseudanabaenaceae cyanobacterium	**Notes on ecophysiological traits**	
**Accession Number(s)_16S**	KU951866	**Isolator**	Bárbara Frazão
**Accession Number(s)_others (product/gene /region)**		**Medium**	Z8 25‰ TM sea salt w/ vit. B12
**Collector**	Bárbara Frazão	**Strain Status**	non-axenic, unicyanobacterial
**Collection Date**	Fall 2006	**Preservation type**	subculturing
**Location**	Portugal: Praia dos Coxos, Sto. Isidoro	**Light:dark cycle**	12:12-h
**Latitude & Longitude**	39.00455 N 9.425842 W	**Temperature (ºC)**	19
		**Light intensity**	10–30 µmol photons m−2 s−1
**References Publicly Available Articles**			
**References Publicly Available Theses**	Frazão B. (2008) MSc Dissertation, FCUP, University of Porto, Porto, Portugal		

Strain ID	**unidentified filamentous Synechococcales LEGE 06070**		
**Strain Taxonomy**			
**Microphotograph 400×**	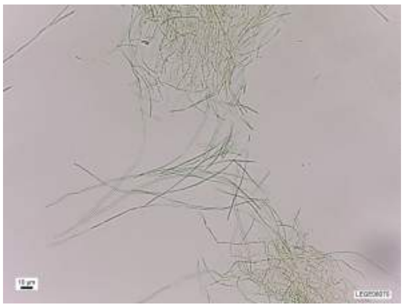	**Microphotograph 1000×**	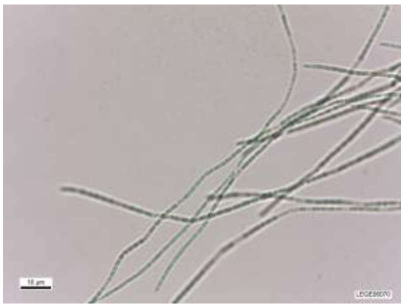
**Coidentity**	n.a.	**Morphometrics (Width × Length or Diameter) (µm)**	1,4±0,2 × 2,1±0,2
**Other Code(s)**		**Taxonomic notes/diacritical features**	
**Parent Sample**	VL 19	**Macroscopic growth features**	color: cyano; forming aggregates
**Isolation Date**		**Environment**	aquatic, brackish
**Order (Older Classification Scheme)**	Oscillatoriales	**Habitat Sample Description**	mesotidal zone, benthic
**Order 2014 (Modern Taxonomy)**	Synechococcales	**Ecophysiological traits**	
**Taxonomy Notes**		**Notes on ecophysiological traits**	
**Accession Number(s)_16S**	HM217074	**Isolator**	Viviana Lopes
**Accession Number(s)_others (product/gene /region)**		**Medium**	Z8
**Collector**	Viviana Lopes	**Strain Status**	non-axenic, unicyanobacterial
**Collection Date**	Oct-2006	**Preservation type**	subculturing
**Location**	Portugal: Douro estuary, Porto	**Light:dark cycle**	12:12-h
**Latitude & Longitude**	41.14658 N 8.632556 W	**Temperature (ºC)**	19
		**Light intensity**	10–30 µmol photons m−2 s−1
**References Publicly Available Articles**	Lopes et al. (2010) Mar. Drugs 8, 471-482; Baptista et al. (2011) Toxicon 58, 410–414; Lopes & Vasconcelos (2011) Mar. Drugs 9, 790-802; Lopes et al. (2011) Toxicol. In Vitro 25, 944–950; Cianca et al. (2012) Amino Acids 42, 2473–2479; Lopes et al. (2012) Mar. Environ. Res. 73, 7–16; Rastoll et al. (2013) J. Appl. Phycol. 25, 1483-1493; Almeida et al. (2015) Toxins 7(8), 2739-2756		
**References Publicly Available Theses**	Lopes V.R. (2011) PhD Thesis, FCUP, University of Porto, Porto, Portugal		

Strain ID	unidentified filamentous Synechococcales LEGE 06116		
**Strain Taxonomy**			
**Microphotograph 400×**	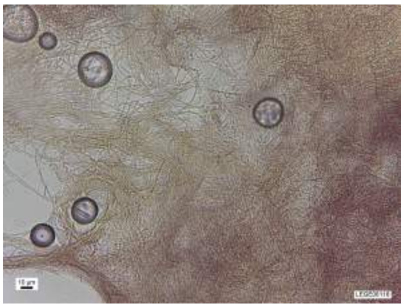	**Microphotograph 1000×**	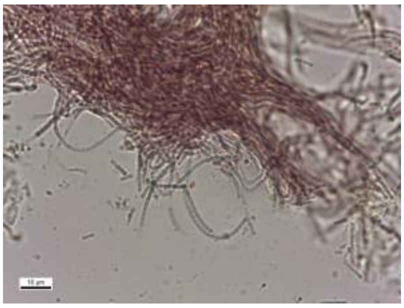
**Coidentity**	n.a.	**Morphometrics (Width × Length or Diameter) (µm)**	1,1±0,1 × 1,4±0,3
**Other Code(s)**	LEAN 021	**Taxonomic notes/diacritical features**	
**Parent Sample**	O.06 MAR 5 - A	**Macroscopic growth features**	color: brownish; forming a firm biofilm
**Isolation Date**		**Environment**	aquatic, marine
**Order (Older Classification Scheme)**	Oscillatoriales	**Habitat Sample Description**	wave-exposed tide pool, rock surface scraping
**Order 2014 (Modern Taxonomy)**	Synechococcales	**Ecophysiological traits**	
**Taxonomy Notes**		**Notes on ecophysiological traits**	
**Accession Number(s)_16S**	KU951867	**Isolator**	Vitor Ramos
**Accession Number(s)_others (product/gene /region)**	KC842341, KC842342 (polyketide synthase)	**Medium**	MN w/ vit. B12
**Collector**	Rui Seabra	**Strain Status**	non-axenic, unicyanobacterial
**Collection Date**	2006/12/11	**Preservation type**	subculturing
**Location**	Portugal: Praia do Martinhal, Vila do Bispo	**Light:dark cycle**	12:12-h
**Latitude & Longitude**	37.01869 N 8.926714 W	**Temperature (ºC)**	19
		**Light intensity**	10–30 µmol photons m−2 s−1
**References Publicly Available Articles**	Brito et al. (2012) Syst. Appl. Microbiol. 35, 110–119; Leão et al. (2013) Mar. Drugs 11 (4), 1316-1335; Brito et al. (2015) Algal Res. 9, 218-226		
**References Publicly Available Theses**			

Strain ID	**unidentified filamentous Synechococcales LEGE 06118**		
**Strain Taxonomy**			
**Microphotograph 400×**	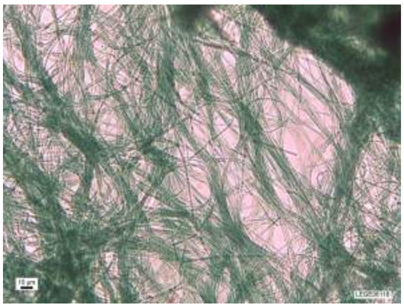	**Microphotograph 1000×**	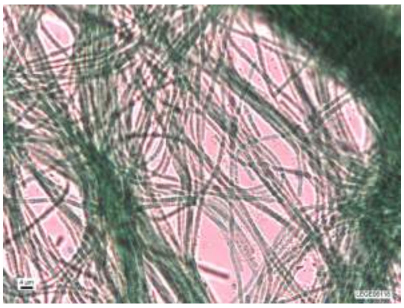
**Coidentity**	n.a.	**Morphometrics (Width × Length or Diameter) (µm)**	1,3±0,1 × 2,2±0,2
**Other Code(s)**	LEAN 023	**Taxonomic notes/diacritical features**	forming fascicles; cell ultrastructure data available
**Parent Sample**	O.06 LUZ 10 - BAAB	**Macroscopic growth features**	color: cyano; forming a firm biofilm
**Isolation Date**		**Environment**	aquatic, marine
**Order (Older Classification Scheme)**	Oscillatoriales	**Habitat Sample Description**	tide puddle, rock surface scraping
**Order 2014 (Modern Taxonomy)**	Synechococcales	**Ecophysiological traits**	
**Taxonomy Notes**		**Notes on ecophysiological traits**	
**Accession Number(s)_16S**	KU951868	**Isolator**	Vitor Ramos
**Accession Number(s)_others (product/gene /region)**	KC842358 (polyketide synthase)	**Medium**	Z8 25‰ TM sea salt w/ vit. B12
**Collector**	Rui Seabra	**Strain Status**	non-axenic, unicyanobacterial
**Collection Date**	2006/12/12	**Preservation type**	subculturing
**Location**	Portugal: Praia da Luz, Lagos	**Light:dark cycle**	12:12-h
**Latitude & Longitude**	37.08622 N 8.725375 W	**Temperature (ºC)**	19
		**Light intensity**	10–30 µmol photons m−2 s−1
**References Publicly Available Articles**	Brito et al. (2012) Syst. Appl. Microbiol. 35, 110–119; Costa et al. (2014) Mar. Drugs 12, 98-114; Brito et al. (2015) Algal Res. 9, 218-226		
**References Publicly Available Theses**			

**Strain ID**	**unidentified filamentous Synechococcales LEGE 06144**		
**Strain Taxonomy**			
**Microphotograph 400×**	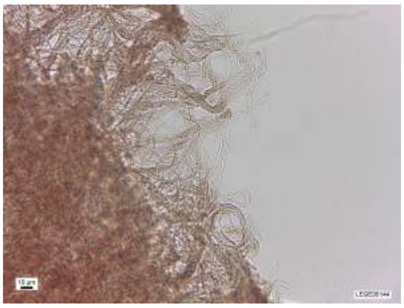	**Microphotograph 1000×**	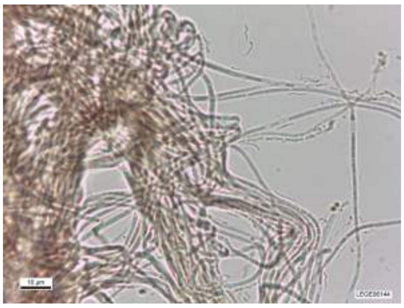
**Coidentity**	n.a.	**Morphometrics (Width × Length or Diameter) (µm)**	1,4±0,2 × 3,3±0,4
**Other Code(s)**	LEAN 056	**Taxonomic notes/diacritical features**	forming fascicles
**Parent Sample**	V.06 BUR 3 - CC	**Macroscopic growth features**	color: brownish; forming a firm biofilm
**Isolation Date**		**Environment**	aquatic, marine
**Order (Older Classification Scheme)**	Oscillatoriales	**Habitat Sample Description**	intertidal zone, wave-sheltered zone, sand
**Order 2014 (Modern Taxonomy)**	Synechococcales	**Ecophysiological traits**	
**Taxonomy Notes**		**Notes on ecophysiological traits**	
**Accession Number(s)_16S**	HQ832937	**Isolator**	Vitor Ramos
**Accession Number(s)_others (product/gene /region)**	KC842348-KC842350 (polyketide synthase)	**Medium**	MN w/ vit. B12
**Collector**	Rui Seabra	**Strain Status**	non-axenic, unicyanobacterial
**Collection Date**	2006/07/17	**Preservation type**	subculturing
**Location**	Portugal: Praia de Burgau, Budens	**Light:dark cycle**	12:12-h
**Latitude & Longitude**	37.07156 N 8.774722 W	**Temperature (ºC)**	19
		**Light intensity**	10–30 µmol photons m−2 s−1
**References Publicly Available Articles**	Brito et al. (2012) Syst. Appl. Microbiol. 35, 110–119; Leão et al. (2013) Mar. Drugs 11 (4), 1316-1335; Costa et al. (2014) Mar. Drugs 12, 98-114; Brito et al. (2015) Algal Res. 9, 218-226		
**References Publicly Available Theses**			

Strain ID	unidentified filamentous Synechococcales LEGE 07075		
**Strain Taxonomy**			
**Microphotograph 400×**	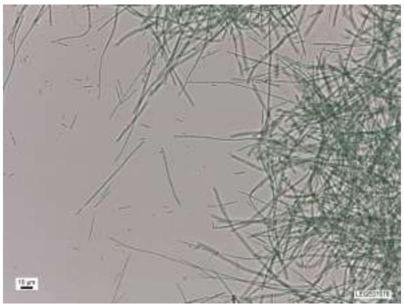	**Microphotograph 1000×**	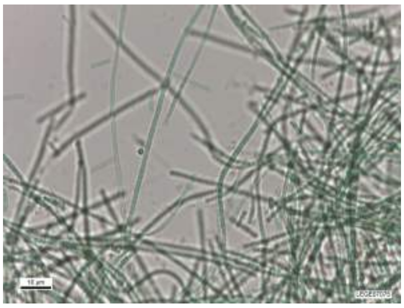
**Coidentity**	n.a.	**Morphometrics (Width × Length or Diameter) (µm)**	1,2±0,2 × 1,3±0,2
**Other Code(s)**		**Taxonomic notes/diacritical features**	facultative sheath
**Parent Sample**	VL 218	**Macroscopic growth features**	color: cyano; forming a firm biofilm
**Isolation Date**		**Environment**	aquatic, brackish
**Order (Older Classification Scheme)**	Oscillatoriales	**Habitat Sample Description**	mesotidal zone, benthic
**Order 2014 (Modern Taxonomy)**	Synechococcales	**Ecophysiological traits**	
**Taxonomy Notes**		**Notes on ecophysiological traits**	
**Accession Number(s)_16S**	HM217080	**Isolator**	Viviana Lopes
**Accession Number(s)_others (product/gene /region)**		**Medium**	Z8
**Collector**	Viviana Lopes	**Strain Status**	non-axenic, unicyanobacterial
**Collection Date**	Apr-2007	**Preservation type**	subculturing
**Location**	Portugal: Douro estuary, Porto	**Light:dark cycle**	12:12-h
**Latitude & Longitude**	41.14658 N 8.632556 W	**Temperature (ºC)**	19
		**Light intensity**	10–30 µmol photons m−2 s−1
**References Publicly Available Articles**	Lopes et al. (2010) Mar. Drugs 8, 471-482; Baptista et al. (2011) Toxicon 58, 410–414; Lopes et al. (2011) Toxicol. In Vitro 25, 944–950; Lopes & Vasconcelos (2011) Mar. Drugs 9, 790-802; Cianca et al. (2012) Amino Acids 42, 2473–2479; Lopes et al. (2012) Mar. Environ. Res. 73, 7–16; Dvořák et al. (2014) Mol. Ecol. 23, 5538–5551; Almeida et al. (2015) Toxins 7(8), 2739-2756		
**References Publicly Available Theses**	Lopes V.R. (2011) PhD Thesis, FCUP, University of Porto, Porto, Portugal		

Strain ID	**unidentified filamentous Synechococcales LEGE 07089**		
**Strain Taxonomy**			
**Microphotograph 400×**	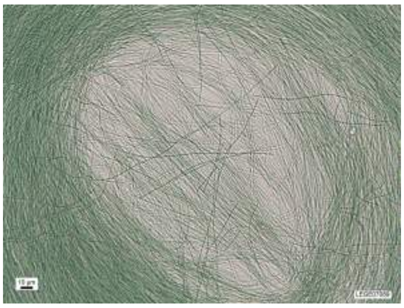	**Microphotograph 1000×**	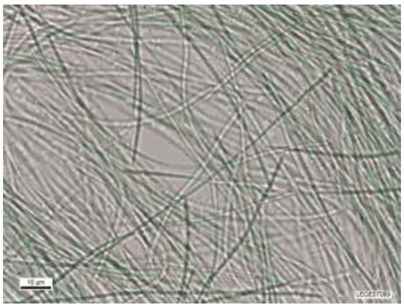
**Coidentity**	n.a.	**Morphometrics (Width × Length or Diameter) (µm)**	1,0±0,2 × 2,7±0,7
**Other Code(s)**		**Taxonomic notes/diacritical features**	
**Parent Sample**	VL 362	**Macroscopic growth features**	color: cyano; forming a firm biofilm
**Isolation Date**		**Environment**	aquatic, brackish
**Order (Older Classification Scheme)**	Oscillatoriales	**Habitat Sample Description**	mesotidal zone, benthic
**Order 2014 (Modern Taxonomy)**	Synechococcales	**Ecophysiological traits**	
**Taxonomy Notes**		**Notes on ecophysiological traits**	
**Accession Number(s)_16S**	HM217063	**Isolator**	Viviana Lopes
**Accession Number(s)_others (product/gene /region)**		**Medium**	Z8
**Collector**	Viviana Lopes	**Strain Status**	non-axenic, unicyanobacterial
**Collection Date**	2006/10/10	**Preservation type**	subculturing
**Location**	Portugal: Douro estuary, Porto	**Light:dark cycle**	12:12-h
**Latitude & Longitude**	41.14658 N 8.657972 W	**Temperature (ºC)**	19
		**Light intensity**	10–30 µmol photons m−2 s−1
**References Publicly Available Articles**	Lopes et al. (2012) Mar. Environ. Res. 73, 7–16		
**References Publicly Available Theses**	Lopes V.R. (2011) PhD Thesis, FCUP, University of Porto, Porto, Portugal		

**Strain ID**	**unidentified filamentous Synechococcales LEGE 07160**		
**Strain Taxonomy**			
**Microphotograph 400×**	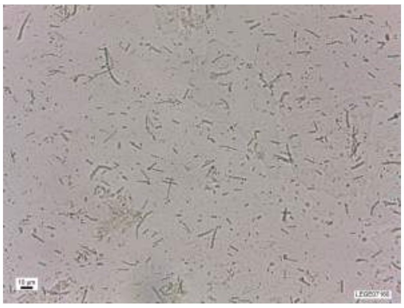	**Microphotograph 1000×**	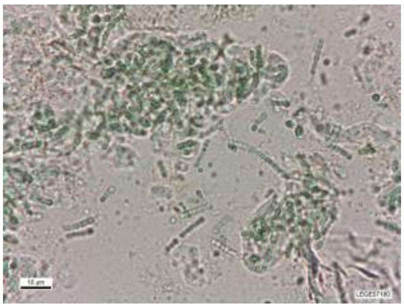
**Coidentity**	n.a.	**Morphometrics (Width × Length or Diameter) (µm)**	1,6±0,2 × 1,6±0,3
**Other Code(s)**	LEAN 074	**Taxonomic notes/diacritical features**	colonies of filaments, within mucilaginous envelopes; filaments constricted at cross-walls
**Parent Sample**	P.07 ODA 6 - p96, dil. 1/4	**Macroscopic growth features**	color: cyano; forming aggregates
**Isolation Date**		**Environment**	aquatic, marine
**Order (Older Classification Scheme)**	Oscillatoriales	**Habitat Sample Description**	tide puddle, on a submerged stone
**Order 2014 (Modern Taxonomy)**	Synechococcales	**Ecophysiological traits**	
**Taxonomy Notes**		**Notes on ecophysiological traits**	
**Accession Number(s)_16S**	KU951869	**Isolator**	Vitor Ramos
**Accession Number(s)_others (product/gene /region)**	KC813192 (non-ribosomal peptide synthetase), KC842368-KC842370 (polyketide synthase)	**Medium**	MN w/ vit. B12
**Collector**	Rui Seabra/Vitor Ramos	**Strain Status**	non-axenic, unicyanobacterial
**Collection Date**	2007/05/02	**Preservation type**	subculturing
**Location**	Portugal: Praia de Olhos d'Água, Albufeira	**Light:dark cycle**	12:12-h
**Latitude & Longitude**	37.08973 N 8.191017 W	**Temperature (ºC)**	19
		**Light intensity**	10–30 µmol photons m−2 s−1
**References Publicly Available Articles**	Brito et al. (2012) Syst. Appl. Microbiol. 35, 110–119; Dvořák et al. (2014) Mol. Ecol. 23, 5538–5551; Costa et al. (2014) Mar. Drugs 12, 98-114; Brito et al. (2015) Algal Res. 9, 218-226		
**References Publicly Available Theses**			

Strain ID	**unidentified filamentous Synechococcales LEGE 07163**		
**Strain Taxonomy**			
**Microphotograph 400×**	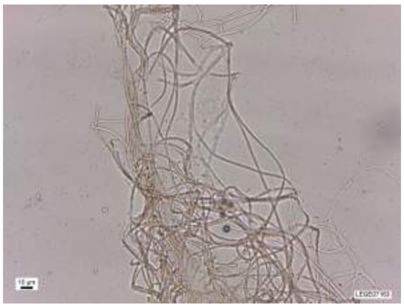	**Microphotograph 1000×**	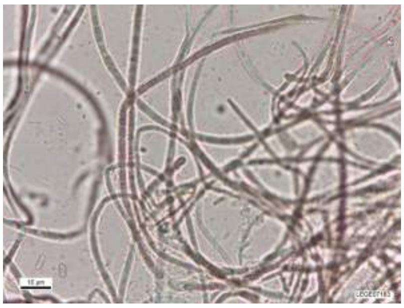
**Coidentity**	n.a.	**Morphometrics (Width × Length or Diameter) (µm)**	2,0±0,4 × 3,5±0,9
**Other Code(s)**	LEAN 077	**Taxonomic notes/diacritical features**	with sheath
**Parent Sample**	P.07 MOL 1 - BC	**Macroscopic growth features**	color: brownish; forming a firm biofilm and aggregates
**Isolation Date**		**Environment**	aquatic, marine
**Order (Older Classification Scheme)**	Oscillatoriales	**Habitat Sample Description**	intertidal zone, on a Mytilus sp. shell
**Order 2014 (Modern Taxonomy)**	Synechococcales	**Ecophysiological traits**	
**Taxonomy Notes**		**Notes on ecophysiological traits**	
**Accession Number(s)_16S**	HQ832900	**Isolator**	Vitor Ramos
**Accession Number(s)_others (product/gene /region)**	KC813171, KC813172 (non-ribosomal peptide synthetase), KC842289 (polyketide synthase)	**Medium**	MN w/ vit. B12
**Collector**	Rui Seabra/Vitor Ramos	**Strain Status**	non-axenic, unicyanobacterial
**Collection Date**	2007/05/21	**Preservation type**	subculturing
**Location**	Portugal: Praia de Moledo, Caminha	**Light:dark cycle**	12:12-h
**Latitude & Longitude**	41.84963 N 8.866717 W	**Temperature (ºC)**	19
		**Light intensity**	10–30 µmol photons m−2 s−1
**References Publicly Available Articles**	Brito et al. (2012) Syst. Appl. Microbiol. 35, 110–119; Costa et al. (2014) Mar. Drugs 12, 98-114; Brito et al. (2015) Algal Res. 9, 218-226		
**References Publicly Available Theses**			

**Strain ID**	**unidentified filamentous Synechococcales LEGE 07185**

Strain Taxonomy

**Microphotograph 400×**	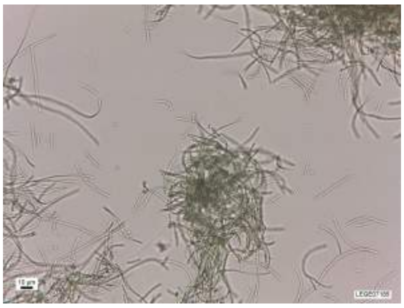	**Microphotograph 1000×**	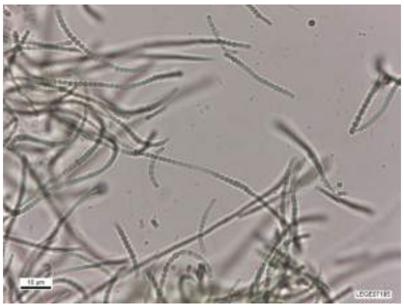
**Coidentity**	n.a.	**Morphometrics (Width × Length or Diameter) (µm)**	1,3±0,2 × 1,4±0,3
**Other Code(s)**	LEAN 099	**Taxonomic notes/diacritical features**	filaments constricted at cross-walls; distinguishable centro- and chromatoplasm
**Parent Sample**	P.07 LAV 6 - p96, dil. 1/2, ABA	**Macroscopic growth features**	color: olive green; forming a firm biofilm and also aggregates
**Isolation Date**		**Environment**	aquatic, marine
**Order (Older Classification Scheme)**	Oscillatoriales	**Habitat Sample Description**	tide pool, on a submerged stone
**Order 2014 (Modern Taxonomy)**	Synechococcales	**Ecophysiological traits**	
**Taxonomy Notes**		**Notes on ecophysiological traits**	
**Accession Number(s)_16S**	KU951870	**Isolator**	Vitor Ramos
**Accession Number(s)_others (product/gene /region)**		**Medium**	Z8 25‰ TM sea salt w/ vit. B12
**Collector**	Rui Seabra/Vitor Ramos	**Strain Status**	non-axenic, unicyanobacterial
**Collection Date**	2007/05/15	**Preservation type**	subculturing
**Location**	Portugal: Praia de Lavadores, Canidelo	**Light:dark cycle**	12:12-h
**Latitude & Longitude**	41.12919 N 8.668578 W	**Temperature (ºC)**	19
		**Light intensity**	10–30 µmol photons m−2 s−1
**References Publicly Available Articles**			
**References Publicly Available Theses**	Macieira R. (2013) MSc Dissertation, FCUP, University of Porto, Porto, Portugal		

Strain ID	unidentified filamentous Synechococcales LEGE 07190		
**Strain Taxonomy**			
**Microphotograph 400×**	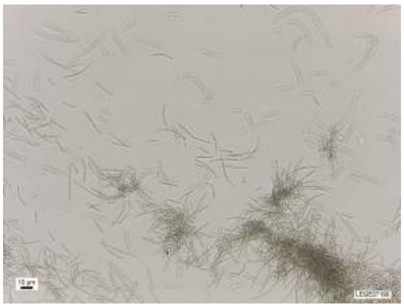	**Microphotograph 1000×**	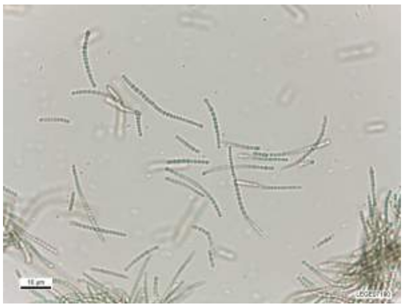
**Coidentity**	n.a.	**Morphometrics (Width × Length or Diameter) (µm)**	1,2±0,1 × 1,1±0,2
**Other Code(s)**	LEAN 155	**Taxonomic notes/diacritical features**	filaments constricted at cross-walls
**Parent Sample**	P.07 LAV 6 - p96, dil. 1/2, ACA	**Macroscopic growth features**	color: olive green; forming a firm biofilm and also aggregates
**Isolation Date**		**Environment**	aquatic, marine
**Order (Older Classification Scheme)**	Oscillatoriales	**Habitat Sample Description**	sea water sample, coastal, surf zone
**Order 2014 (Modern Taxonomy)**	Synechococcales	**Ecophysiological traits**	
**Taxonomy Notes**		**Notes on ecophysiological traits**	
**Accession Number(s)_16S**	HQ832919	**Isolator**	Vitor Ramos
**Accession Number(s)_others (product/gene /region)**	KC842320, KC842321 (polyketide synthase)	**Medium**	Z8 25‰ TM sea salt w/ vit. B12
**Collector**	Rui Seabra/Vitor Ramos	**Strain Status**	non-axenic, unicyanobacterial
**Collection Date**	2007/05/15	**Preservation type**	subculturing
**Location**	Portugal: Praia de Lavadores, Canidelo	**Light:dark cycle**	12:12-h
**Latitude & Longitude**	41.12919 N 8.668578 W	**Temperature (ºC)**	19
		**Light intensity**	10–30 µmol photons m−2 s−1
**References Publicly Available Articles**	Brito et al. (2012) Syst. Appl. Microbiol. 35, 110–119; Brito et al. (2015) Algal Res. 9, 218-226		
**References Publicly Available Theses**			

Strain ID	**unidentified filamentous Synechococcales LEGE 07319**		
**Strain Taxonomy**			
**Microphotograph 400×**	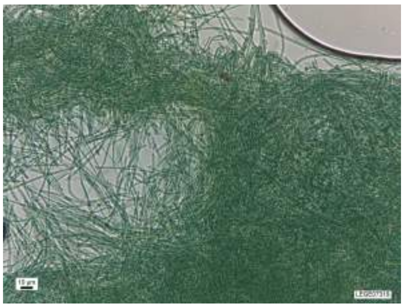	**Microphotograph 1000×**	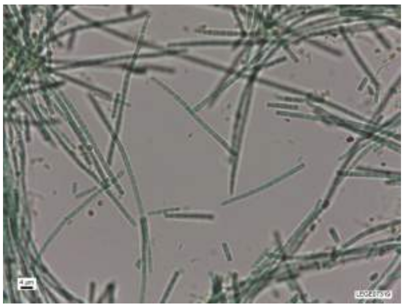
**Coidentity**	n.a.	**Morphometrics (Width × Length or Diameter) (µm)**	1,4±0,1 × 2,4±0,4
**Other Code(s)**		**Taxonomic notes/diacritical features**	
**Parent Sample**	SAL269	**Macroscopic growth features**	color: cyano; forming a firm biofilm
**Isolation Date**		**Environment**	aquatic, brackish
**Order (Older Classification Scheme)**	Oscillatoriales	**Habitat Sample Description**	unknown
**Order 2014 (Modern Taxonomy)**	Synechococcales	**Ecophysiological traits**	
**Taxonomy Notes**		**Notes on ecophysiological traits**	
**Accession Number(s)_16S**	HM217045	**Isolator**	Viviana Lopes
**Accession Number(s)_others (product/gene /region)**		**Medium**	MN w/ vit. B12
**Collector**	Viviana Lopes	**Strain Status**	non-axenic, unicyanobacterial
**Collection Date**	2007	**Preservation type**	subculturing
**Location**	Portugal: Vouga estuary, Ria de Aveiro, Aveiro	**Light:dark cycle**	12:12-h
**Latitude & Longitude**	40.63825 N 8.659778 W	**Temperature (ºC)**	19
		**Light intensity**	10–30 µmol photons m−2 s−1
**References Publicly Available Articles**	Lopes et al. (2012) Mar. Environ. Res. 73, 7–16		
**References Publicly Available Theses**	Lopes V.R. (2011) PhD Thesis, FCUP, University of Porto, Porto, Portugal		

Strain ID	**unidentified filamentous Synechococcales LEGE 08404**		
**Strain Taxonomy**			
**Microphotograph 400×**	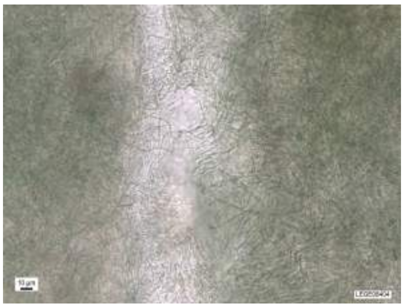	**Microphotograph 1000×**	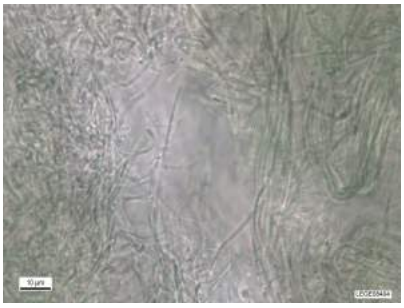
**Coidentity**	n.a.	**Morphometrics (Width × Length or Diameter) (µm)**	1,1±0,1 × 2,2±0,5
**Other Code(s)**		**Taxonomic notes/diacritical features**	with sheath
**Parent Sample**	P.08 LUZ-3-EACA	**Macroscopic growth features**	color: cyano
**Isolation Date**		**Environment**	aquatic, marine
**Order (Older Classification Scheme)**	Oscillatoriales	**Habitat Sample Description**	intertidal zone, tide puddle, biofilm on a rock
**Order 2014 (Modern Taxonomy)**	Synechococcales	**Ecophysiological traits**	
**Taxonomy Notes**	Pseudanabaenaceae cyanobacterium	**Notes on ecophysiological traits**	
**Accession Number(s)_16S**	KU951871	**Isolator**	Vitor Ramos
**Accession Number(s)_others (product/gene /region)**		**Medium**	Z8 25‰ TM sea salt w/ vit. B12
**Collector**	Vitor Ramos	**Strain Status**	non-axenic, unicyanobacterial
**Collection Date**	May-2008	**Preservation type**	subculturing
**Location**	Portugal: Praia da Luz, Lagos	**Light:dark cycle**	14:10-h
**Latitude & Longitude**	37.08622 N 8.725375 W	**Temperature (ºC)**	25
		**Light intensity**	10–30 µmol photons m−2 s−1
**References Publicly Available Articles**			
**References Publicly Available Theses**			

Strain ID	**unidentified filamentous Synechococcales LEGE 10405**		
**Strain Taxonomy**			
**Microphotograph 400×**	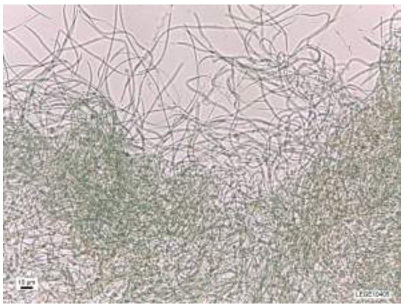	**Microphotograph 1000×**	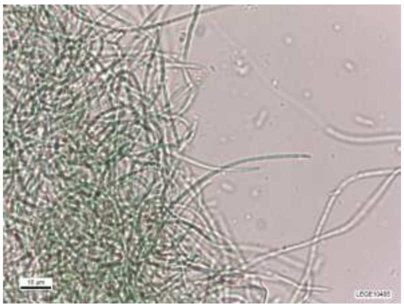
**Coidentity**	n.a.	**Morphometrics (Width × Length or Diameter) (µm)**	1,1±0,1 × 2,5±0,6
**Other Code(s)**		**Taxonomic notes/diacritical features**	with sheath
**Parent Sample**	S2-BA	**Macroscopic growth features**	color: cyano; forming a firm biofilm
**Isolation Date**		**Environment**	aquatic, marine
**Order (Older Classification Scheme)**	Oscillatoriales	**Habitat Sample Description**	intertidal zone
**Order 2014 (Modern Taxonomy)**	Synechococcales	**Ecophysiological traits**	
**Taxonomy Notes**		**Notes on ecophysiological traits**	
**Accession Number(s)_16S**	KU951872	**Isolator**	Vitor Ramos
**Accession Number(s)_others (product/gene /region)**		**Medium**	MN w/ vit. B12
**Collector**	Bárbara Frazão	**Strain Status**	non-axenic, unicyanobacterial
**Collection Date**	2010/04/01	**Preservation type**	subculturing
**Location**	Portugal: Selvagens Islands	**Light:dark cycle**	12:12-h
**Latitude & Longitude**	30.03458 N 16.03267 W	**Temperature (ºC)**	19
		**Light intensity**	10–30 µmol photons m−2 s−1
**References Publicly Available Articles**			
**References Publicly Available Theses**			

Strain ID	**unidentified filamentous Synechococcales LEGE 11384**		
**Strain Taxonomy**			
**Microphotograph 400×**	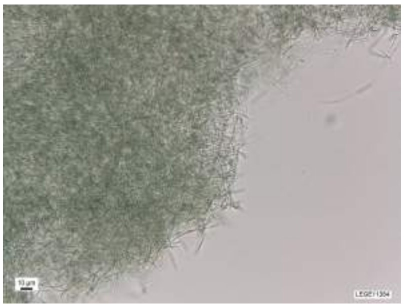	**Microphotograph 1000×**	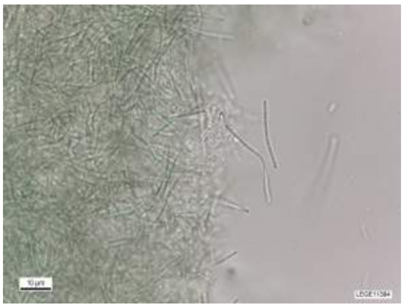
**Coidentity**	n.a.	**Morphometrics (Width × Length or Diameter) (µm)**	0,9±0,1 × 2,5±0,4
**Other Code(s)**	23D	**Taxonomic notes/diacritical features**	
**Parent Sample**		**Macroscopic growth features**	color: cyano
**Isolation Date**		**Environment**	aquatic, marine
**Order (Older Classification Scheme)**	Oscillatoriales	**Habitat Sample Description**	intertidal zone, on a marine sponge
**Order 2014 (Modern Taxonomy)**	Synechococcales	**Ecophysiological traits**	
**Taxonomy Notes**		**Notes on ecophysiological traits**	
**Accession Number(s)_16S**	JQ927355	**Isolator**	Ana Regueiras
**Accession Number(s)_others (product/gene /region)**		**Medium**	MN w/ vit. B12
**Collector**	Ana Regueiras	**Strain Status**	non-axenic, unicyanobacterial
**Collection Date**	2011/09/29	**Preservation type**	subculturing
**Location**	Portugal: beach near Lavra, Matosinhos	**Light:dark cycle**	12:12-h
**Latitude & Longitude**	41.23319 N 8.723944 W	**Temperature (ºC)**	19
		**Light intensity**	10–30 µmol photons m−2 s−1
**References Publicly Available Articles**			
**References Publicly Available Theses**	Vivas J. (2013) MSc Dissertation, FCUP, University of Porto, Porto, Portugal		

**Strain ID**	**unidentified filamentous Synechococcales LEGE 11392**		
**Strain Taxonomy**			
**Microphotograph 400×**	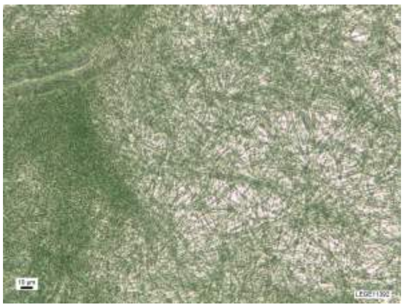	**Microphotograph 1000×**	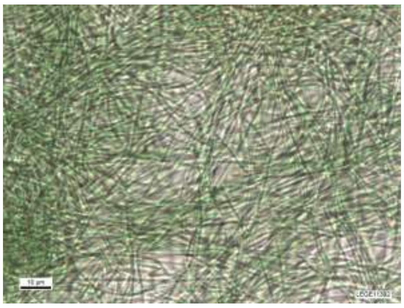
**Coidentity**	n.a.	**Morphometrics (Width × Length or Diameter) (µm)**	1,0±0,1 × 4,7±1,0
**Other Code(s)**		**Taxonomic notes/diacritical features**	bright refractive granules (gas vesicles?) at cell ends
**Parent Sample**	EB3-1-EAA	**Macroscopic growth features**	color: cyano; forming a firm biofilm
**Isolation Date**		**Environment**	aquatic, hypersaline
**Order (Older Classification Scheme)**	Oscillatoriales	**Habitat Sample Description**	upper layer of a microbial mat
**Order 2014 (Modern Taxonomy)**	Synechococcales	**Ecophysiological traits**	
**Taxonomy Notes**		**Notes on ecophysiological traits**	
**Accession Number(s)_16S**	KU951873	**Isolator**	Vitor Ramos
**Accession Number(s)_others (product/gene /region)**		**Medium**	MN w/ vit. B12
**Collector**	Frederico Sobrinho/Sinda Carvalhal	**Strain Status**	non-axenic, unicyanobacterial
**Collection Date**	9-Feb-2011	**Preservation type**	subculturing
**Location**	Brazil: Pitanguinha lagoon (Araruama system), in the state of Rio de Janeiro	**Light:dark cycle**	12:12-h
**Latitude & Longitude**	22.9275 S 42.35556 W	**Temperature (ºC)**	19
		**Light intensity**	10–30 µmol photons m−2 s−1
**References Publicly Available Articles**			
**References Publicly Available Theses**			

Strain ID	unidentified filamentous Synechococcales LEGE 11395		
**Strain Taxonomy**			
**Microphotograph 400×**	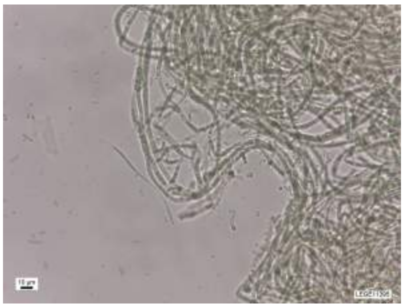	**Microphotograph 1000×**	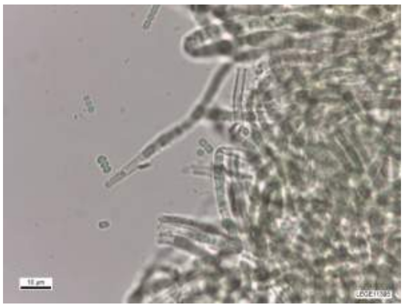
**Coidentity**	n.a.	**Morphometrics (Width × Length or Diameter) (µm)**	2,6±0,6 × 2,0±0,4
**Other Code(s)**		**Taxonomic notes/diacritical features**	trichomes constricted at cross-walls; with sheath
**Parent Sample**	EB5-1-EAA (EB5=EB2)	**Macroscopic growth features**	color: cyano; forming a firm biofilm and aggregates
**Isolation Date**		**Environment**	aquatic, hypersaline
**Order (Older Classification Scheme)**	Oscillatoriales	**Habitat Sample Description**	upper layer of a microbial mat
**Order 2014 (Modern Taxonomy)**	Synechococcales	**Ecophysiological traits**	
**Taxonomy Notes**		**Notes on ecophysiological traits**	
**Accession Number(s)_16S**	KU951874	**Isolator**	Vitor Ramos
**Accession Number(s)_others (product/gene /region)**		**Medium**	MN w/ vit. B12
**Collector**	Frederico Sobrinho/Sinda Carvalhal	**Strain Status**	non-axenic, unicyanobacterial
**Collection Date**	9-Feb-2011	**Preservation type**	subculturing
**Location**	Brazil: Pernambuco lagoon (Araruama system), in the state of Rio de Janeiro	**Light:dark cycle**	12:12-h
**Latitude & Longitude**	22.93056 S 42.32389 W	**Temperature (ºC)**	19
		**Light intensity**	10–30 µmol photons m−2 s−1
**References Publicly Available Articles**			
**References Publicly Available Theses**			

Strain ID	unidentified filamentous Synechococcales LEGE 12432		
**Strain Taxonomy**			
**Microphotograph 400×**	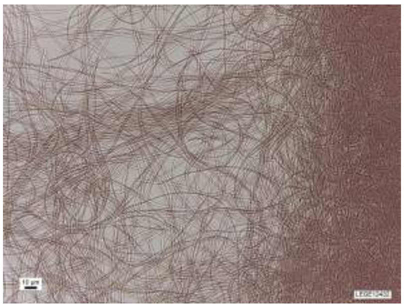	**Microphotograph 1000×**	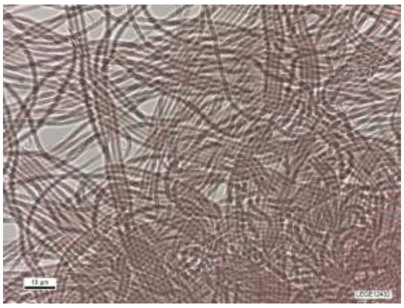
**Coidentity**	n.a.	**Morphometrics (Width × Length or Diameter) (µm)**	1,2±0,1 × 2,0±0,4
**Other Code(s)**		**Taxonomic notes/diacritical features**	trichomes constricted at cross-walls
**Parent Sample**	LC3-AAA	**Macroscopic growth features**	color: brownish; forming a smooth biofilm
**Isolation Date**		**Environment**	aquatic, freshwater
**Order (Older Classification Scheme)**	Oscillatoriales	**Habitat Sample Description**	water sample
**Order 2014 (Modern Taxonomy)**	Synechococcales	**Ecophysiological traits**	
**Taxonomy Notes**	Pseudanabaenaceae cyanobacterium	**Notes on ecophysiological traits**	
**Accession Number(s)_16S**	KU951859	**Isolator**	Vitor Ramos
**Accession Number(s)_others (product/gene /region)**		**Medium**	Z8
**Collector**	Jorge Nimptsch	**Strain Status**	non-axenic, unicyanobacterial
**Collection Date**	2012	**Preservation type**	subculturing
**Location**	Chile: Ranco Lake, La Araucania region	**Light:dark cycle**	12:12-h
**Latitude & Longitude**	unknown	**Temperature (ºC)**	19
		**Light intensity**	10–30 µmol photons m−2 s−1
**References Publicly Available Articles**			
**References Publicly Available Theses**			

Strain ID	**unidentified filamentous Synechococcales LEGE 13422**		
**Strain Taxonomy**			
**Microphotograph 400×**	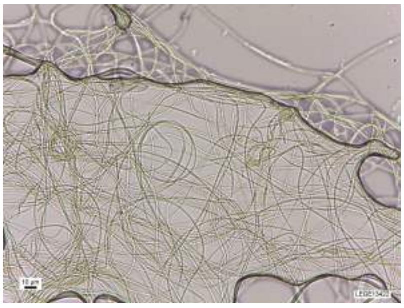	**Microphotograph 1000×**	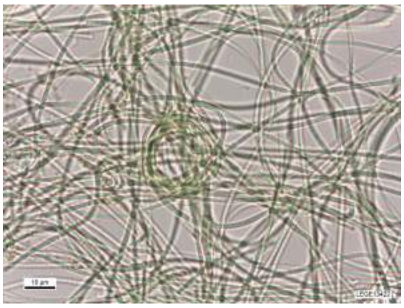
**Coidentity**	n.a.	**Morphometrics (Width × Length or Diameter) (µm)**	1,4±0,4 × 1,3±0,2
**Other Code(s)**	S6F	**Taxonomic notes/diacritical features**	some helix-coiled trichomes; nodules observed
**Parent Sample**		**Macroscopic growth features**	color: greenish; forming a smooth biofilm
**Isolation Date**		**Environment**	unknown
**Order (Older Classification Scheme)**	Oscillatoriales	**Habitat Sample Description**	"Spirulina" dietary supplements, capsules form. Producer/brand: Solaray
**Order 2014 (Modern Taxonomy)**	Synechococcales	**Ecophysiological traits**	
**Taxonomy Notes**	Nodosilinea-like sp.	**Notes on ecophysiological traits**	
**Accession Number(s)_16S**	KU951875	**Isolator**	Dina Gomes
**Accession Number(s)_others (product/gene /region)**		**Medium**	Z8
**Collector**	Dina Gomes	**Strain Status**	non-axenic, unicyanobacterial
**Collection Date**	2013	**Preservation type**	subculturing
**Location**	original source unknown: acquired in the Porto metropolitan area, Portugal	**Light:dark cycle**	12:12-h
**Latitude & Longitude**	unknown	**Temperature (ºC)**	19
		**Light intensity**	10–30 µmol photons m−2 s−1
**References Publicly Available Articles**			
**References Publicly Available Theses**	Gomes D. (2013) MSc Dissertation, ICBAS, University of Porto, Porto, Portugal		

Strain ID	**unidentified filamentous cyanobacterium LEGE 00033**		
**Strain Taxonomy**			
**Microphotograph 400×**	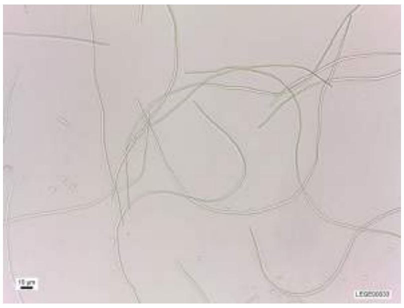	**Microphotograph 1000×**	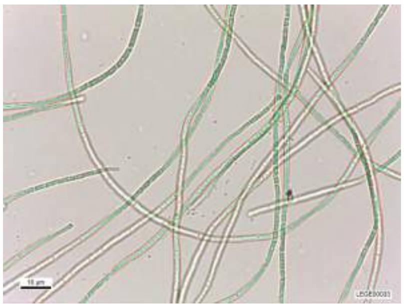
**Coidentity**	n.a.	**Morphometrics (Width × Length or Diameter) (µm)**	1,6±0,1 × 2,8±0,6
**Other Code(s)**	LEAN 207	**Taxonomic notes/diacritical features**	distinguishable centro- and chromatoplasm
**Parent Sample**	Aguda 18	**Macroscopic growth features**	color: cyano; forming a smooth biofilm
**Isolation Date**		**Environment**	aquatic, marine
**Order (Older Classification Scheme)**	Oscillatoriales	**Habitat Sample Description**	intertidal zone, Sabellaria alveolata reef
**Order 2014 (Modern Taxonomy)**	undefined	**Ecophysiological traits**	
**Taxonomy Notes**		**Notes on ecophysiological traits**	
**Accession Number(s)_16S**	KU951849	**Isolator**	Rosario Martins
**Accession Number(s)_others (product/gene /region)**		**Medium**	Z8 25‰ TM sea salt w/ vit. B12
**Collector**	Rosario Martins	**Strain Status**	non-axenic, unicyanobacterial
**Collection Date**	2000/05/31	**Preservation type**	subculturing
**Location**	Portugal: Praia da Aguda, Arcozelo	**Light:dark cycle**	12:12-h
**Latitude & Longitude**	41.04954 N 8.655339 W	**Temperature (ºC)**	19
		**Light intensity**	10–30 µmol photons m−2 s−1
**References Publicly Available Articles**			
**References Publicly Available Theses**			

**Strain ID**	**unidentified filamentous cyanobacterium LEGE 00060**		
**Strain Taxonomy**			
**Microphotograph 400×**	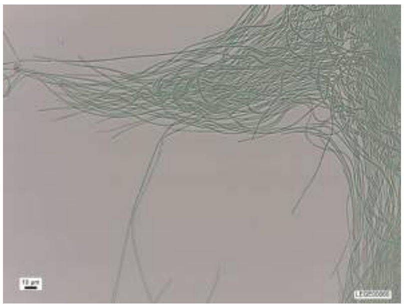	**Microphotograph 1000×**	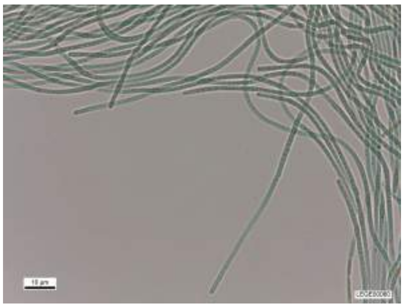
**Coidentity**	n.a.	**Morphometrics (Width × Length or Diameter) (µm)**	1,5±0,3 × 1,9±0,8
**Other Code(s)**		**Taxonomic notes/diacritical features**	trichomes with "meristematic" regions
**Parent Sample**	Melah	**Macroscopic growth features**	color: cyano; forming a firm biofilm
**Isolation Date**		**Environment**	aquatic, freshwater
**Order (Older Classification Scheme)**	Oscillatoriales	**Habitat Sample Description**	unknown
**Order 2014 (Modern Taxonomy)**	undefined	**Ecophysiological traits**	
**Taxonomy Notes**	Pseudanabaenaceae cyanobacterium	**Notes on ecophysiological traits**	
**Accession Number(s)_16S**	KU951851	**Isolator**	Martin Saker
**Accession Number(s)_others (product/gene /region)**		**Medium**	Z8
**Collector**	unknown	**Strain Status**	non-axenic, unicyanobacterial
**Collection Date**	2000	**Preservation type**	subculturing, cryopreserved
**Location**	Morocco: Oued Mellah	**Light:dark cycle**	12:12-h
**Latitude & Longitude**	unknown	**Temperature (ºC)**	19
		**Light intensity**	10–30 µmol photons m−2 s−1
**References Publicly Available Articles**	Leão et al. (2009) Eur. J. Phycol. 44, 347-355		
**References Publicly Available Theses**	Leão P.N. (2010) PhD Thesis, FCUP, University of Porto, Porto, Portugal		

**Strain ID**	**unidentified filamentous cyanobacterium LEGE 06007**		
**Strain Taxonomy**			
**Microphotograph 400×**	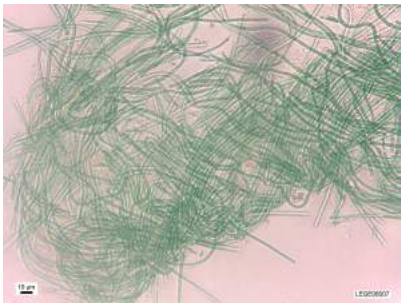	**Microphotograph 1000×**	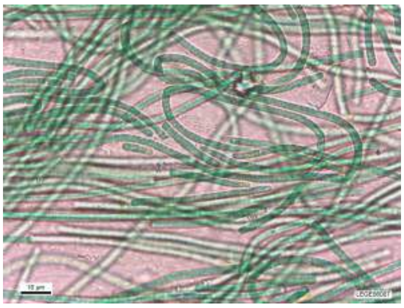
**Coidentity**	n.a.	**Morphometrics (Width × Length or Diameter) (µm)**	2,2±0,3 × 2,5±0,4
**Other Code(s)**	strain 10, LEAN 103	**Taxonomic notes/diacritical features**	with necridic cells and hormogonia; narrow sheath
**Parent Sample**	Ref. 10	**Macroscopic growth features**	color: cyano; forming a smooth biofilm
**Isolation Date**		**Environment**	aquatic, marine
**Order (Older Classification Scheme)**	Oscillatoriales	**Habitat Sample Description**	intertidal zone, wave-exposed rock
**Order 2014 (Modern Taxonomy)**	undefined	**Ecophysiological traits**	
**Taxonomy Notes**		**Notes on ecophysiological traits**	
**Accession Number(s)_16S**	KU951852	**Isolator**	Bárbara Frazão
**Accession Number(s)_others (product/gene /region)**		**Medium**	Z8 25‰ TM sea salt w/ vit. B12
**Collector**	Bárbara Frazão	**Strain Status**	non-axenic, unicyanobacterial
**Collection Date**	Fall 2006	**Preservation type**	subculturing
**Location**	Portugal: Praia de São Martinho do Porto	**Light:dark cycle**	12:12-h
**Latitude & Longitude**	39.50694 N 9.136311 W	**Temperature (ºC)**	19
		**Light intensity**	10–30 µmol photons m−2 s−1
**References Publicly Available Articles**	Frazão et al. (2010) Mar. Drugs 8, 1908-1919		
**References Publicly Available Theses**	Frazão B. (2008) MSc Dissertation, FCUP, University of Porto, Porto, Portugal; Macieira R. (2013) MSc Dissertation, FCUP, University of Porto, Porto, Portugal		

**Strain ID**	**unidentified filamentous cyanobacterium LEGE 07170**
**Strain Taxonomy**	

**Microphotograph 400×**	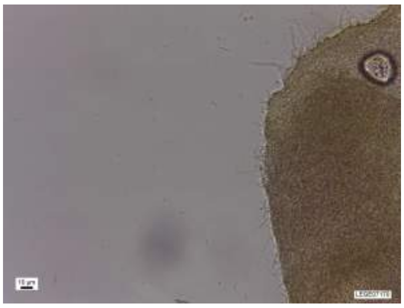	**Microphotograph 1000×**	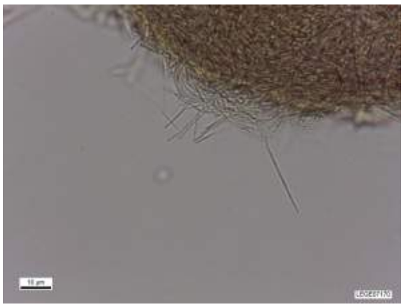
**Coidentity**	n.a.	**Morphometrics (Width × Length or Diameter) (µm)**	0,9±0,2 × 1,0±0,2
**Other Code(s)**	LEAN 084	**Taxonomic notes/diacritical features**	hardly distinguished cell walls
**Parent Sample**	P.07 ODA 7 - BBA	**Macroscopic growth features**	color: dark green; forming a firm biofilm
**Isolation Date**		**Environment**	aquatic, marine
**Order (Older Classification Scheme)**	Oscillatoriales	**Habitat Sample Description**	wave-exposed tide puddle
**Order 2014 (Modern Taxonomy)**	undefined	**Ecophysiological traits**	
**Taxonomy Notes**		**Notes on ecophysiological traits**	
**Accession Number(s)_16S**	KU951854	**Isolator**	Vitor Ramos
**Accession Number(s)_others (product/gene /region)**	KC256771 (dinitrogenase reductase, nifH), KC842371 (polyketide synthase)	**Medium**	MN w/ vit. B12
**Collector**	Rui Seabra/Vitor Ramos	**Strain Status**	non-axenic, unicyanobacterial
**Collection Date**	2007/05/02	**Preservation type**	subculturing
**Location**	Portugal: Praia de Olhos d'Água, Albufeira	**Light:dark cycle**	12:12-h
**Latitude & Longitude**	37.08973 N 8.191017 W	**Temperature (ºC)**	19
		**Light intensity**	10–30 µmol photons m−2 s−1

**References Publicly Available Articles**	Brito et al. (2012) Syst. Appl. Microbiol. 35, 110–119; Brito et al. (2015) Algal Res. 9, 218-226
**References Publicly Available Theses**	Macieira R. (2013) MSc Dissertation, FCUP, University of Porto, Porto, Portugal

**Strain ID**	**unidentified filamentous cyanobacterium LEGE 07209**		
**Strain Taxonomy**			
**Microphotograph 400×**	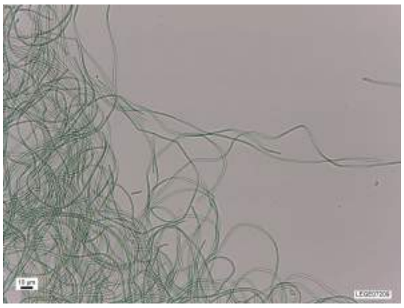	**Microphotograph 1000×**	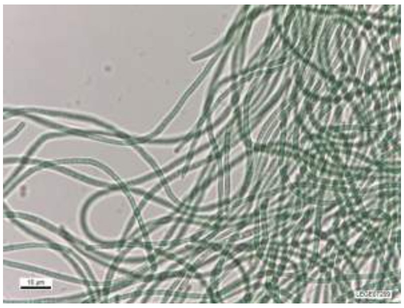
**Coidentity**	n.a.	**Morphometrics (Width × Length or Diameter) (µm)**	1,4±0,2 × 2,9±1,2
**Other Code(s)**		**Taxonomic notes/diacritical features**	
**Parent Sample**	A1 AG - 4 PQN	**Macroscopic growth features**	color: cyano; forming a smooth biofilm and aggregates
**Isolation Date**		**Environment**	aquatic, freshwater
**Order (Older Classification Scheme)**	Oscillatoriales	**Habitat Sample Description**	biofilm, from a biological treatment tank outlet
**Order 2014 (Modern Taxonomy)**	undefined	**Ecophysiological traits**	
**Taxonomy Notes**	Pseudanabaenaceae cyanobacterium	**Notes on ecophysiological traits**	
**Accession Number(s)_16S**	KU951856	**Isolator**	Joana Martins
**Accession Number(s)_others (product/gene /region)**		**Medium**	Z8
**Collector**	Joana Martins	**Strain Status**	non-axenic, unicyanobacterial
**Collection Date**	Aug-2007	**Preservation type**	subculturing
**Location**	Portugal: wastewater treatment plant at Febros river, Vila Nova de Gaia	**Light:dark cycle**	12:12-h
**Latitude & Longitude**	41.11812 N 8.570944 W	**Temperature (ºC)**	19
		**Light intensity**	10–30 µmol photons m−2 s−1
**References Publicly Available Articles**			
**References Publicly Available Theses**			

Strain ID	unidentified filamentous cyanobacterium LEGE 07212		
**Strain Taxonomy**			
**Microphotograph 400×**	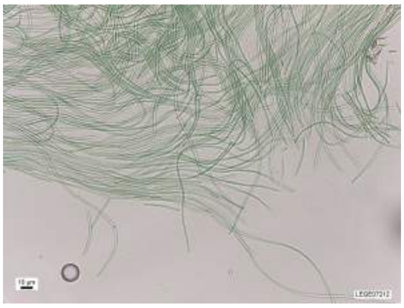	**Microphotograph 1000×**	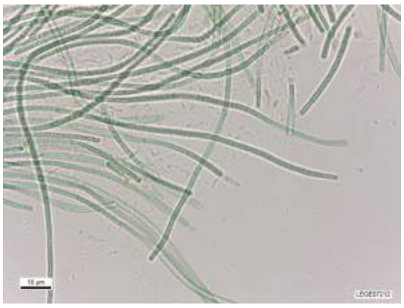
**Coidentity**	n.a.	**Morphometrics (Width × Length or Diameter) (µm)**	1,4±0,2 × 2,0±0,4
**Other Code(s)**		**Taxonomic notes/diacritical features**	facultative sheath
**Parent Sample**	A1 AG - 7 PQN	**Macroscopic growth features**	color: cyano; forming a smooth biofilm and aggregates
**Isolation Date**		**Environment**	aquatic, freshwater
**Order (Older Classification Scheme)**	Oscillatoriales	**Habitat Sample Description**	biofilm, from a biological treatment tank outlet
**Order 2014 (Modern Taxonomy)**	undefined	**Ecophysiological traits**	
**Taxonomy Notes**	Pseudanabaenaceae cyanobacterium	**Notes on ecophysiological traits**	
**Accession Number(s)_16S**	KU951857	**Isolator**	Joana Martins
**Accession Number(s)_others (product/gene /region)**		**Medium**	Z8
**Collector**	Joana Martins	**Strain Status**	non-axenic, unicyanobacterial
**Collection Date**	Aug-2007	**Preservation type**	subculturing
**Location**	Portugal: wastewater treatment plant at Febros river, Vila Nova de Gaia	**Light:dark cycle**	12:12-h
**Latitude & Longitude**	41.11812 N 8.570944 W	**Temperature (ºC)**	19
		**Light intensity**	10–30 µmol photons m−2 s−1
**References Publicly Available Articles**			
**References Publicly Available Theses**			

Strain ID	**unidentified filamentous cyanobacterium LEGE 11480**		
**Strain Taxonomy**			
**Microphotograph 400×**	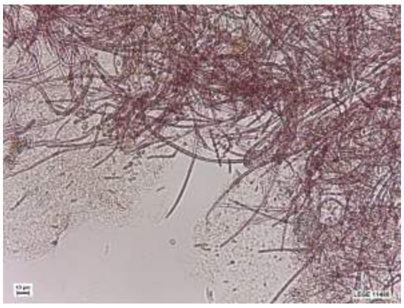	**Microphotograph 1000×**	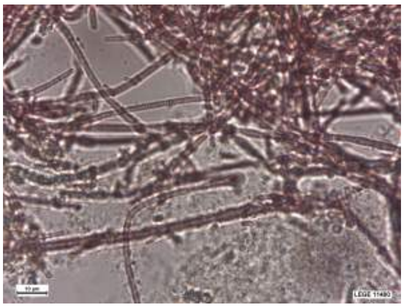
**Coidentity**	n.a.	**Morphometrics (Width × Length or Diameter) (µm)**	1,9±0,3 × 1,2±0,4
**Other Code(s)**		**Taxonomic notes/diacritical features**	with sheath
**Parent Sample**	5.002.1011	**Macroscopic growth features**	color: brown-reddish; forming a smooth biofilm and aggregates
**Isolation Date**		**Environment**	aquatic, marine
**Order (Older Classification Scheme)**	Oscillatoriales	**Habitat Sample Description**	subtidal sample, epilithic (13m depth), about 200 m off the shore
**Order 2014 (Modern Taxonomy)**	not determined	**Ecophysiological traits**	
**Taxonomy Notes**	Pseudanabaenaceae cyanobacterium	**Notes on ecophysiological traits**	
**Accession Number(s)_16S**	ready to submit	**Isolator**	Ângela Pinheiro
**Accession Number(s)_others (product/gene /region)**		**Medium**	Z8 25‰ TM sea salt w/ vit. B12
**Collector**	Pedro Leão/Aldo Barreiro	**Strain Status**	non-axenic, unicyanobacterial
**Collection Date**	2011/10/03	**Preservation type**	subculturing
**Location**	Portugal: 'A Pedra', diving spot in front of the fort 'Castelo do Queijo'	**Light:dark cycle**	12:12-h
**Latitude & Longitude**	41.185809 N 8.719079 W	**Temperature (ºC)**	19
		**Light intensity**	10–30 µmol photons m−2 s−1
**References Publicly Available Articles**			
**References Publicly Available Theses**			

Strain ID	**unidentified filamentous cyanobacterium LEGE 12433**		
**Strain Taxonomy**			
**Microphotograph 400×**	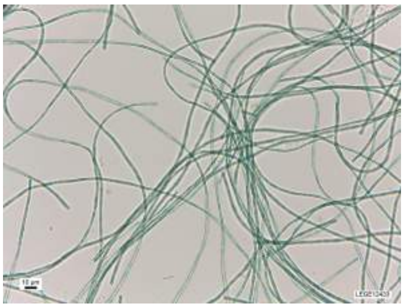	**Microphotograph 1000×**	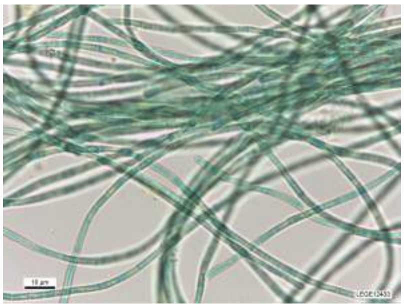
**Coidentity**	n.a.	**Morphometrics (Width × Length or Diameter) (µm)**	1,9±0,2 × 5,7±0,7
**Other Code(s)**		**Taxonomic notes/diacritical features**	
**Parent Sample**	LC5-BAAA	**Macroscopic growth features**	color: cyano; forming a smooth biofilm
**Isolation Date**		**Environment**	aquatic, freshwater
**Order (Older Classification Scheme)**	Oscillatoriales	**Habitat Sample Description**	water sample
**Order 2014 (Modern Taxonomy)**	undefined	**Ecophysiological traits**	
**Taxonomy Notes**	Pseudanabaenaceae cyanobacterium	**Notes on ecophysiological traits**	earth odour (geosmin?)
**Accession Number(s)_16S**	KU951860	**Isolator**	Vitor Ramos
**Accession Number(s)_others (product/gene /region)**		**Medium**	Z8
**Collector**	Jorge Nimptsch	**Strain Status**	non-axenic, unicyanobacterial
**Collection Date**	2012	**Preservation type**	subculturing
**Location**	Chile: Caburgua Lake, La Araucania region	**Light:dark cycle**	12:12-h
**Latitude & Longitude**	unknown	**Temperature (ºC)**	19
		**Light intensity**	10–30 µmol photons m−2 s−1
**References Publicly Available Articles**			
**References Publicly Available Theses**			

Strain ID	**unidentified filamentous cyanobacterium LEGE 12440**		
**Strain Taxonomy**			
**Microphotograph 400×**	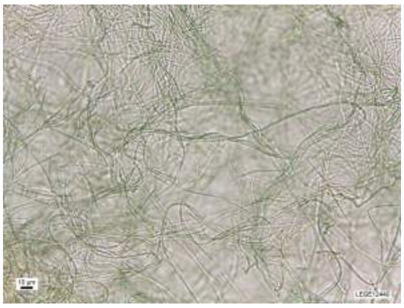	**Microphotograph 1000×**	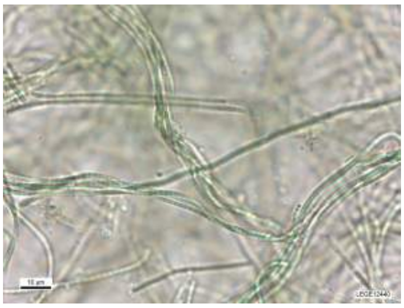
**Coidentity**	n.a.	**Morphometrics (Width × Length or Diameter) (µm)**	1,4±0,2 × 1,8±0,3
**Other Code(s)**		**Taxonomic notes/diacritical features**	
**Parent Sample**	EB4 - 10 A/2	**Macroscopic growth features**	color: cyano; forming a firm biofilm
**Isolation Date**		**Environment**	aquatic, hypersaline
**Order (Older Classification Scheme)**	Oscillatoriales	**Habitat Sample Description**	upper layer of a microbial mat
**Order 2014 (Modern Taxonomy)**	not determined	**Ecophysiological traits**	
**Taxonomy Notes**	Pseudanabaenaceae cyanobacterium	**Notes on ecophysiological traits**	
**Accession Number(s)_16S**	ready to submit	**Isolator**	Ângela Pinheiro/Vitor Ramos
**Accession Number(s)_others (product/gene /region)**		**Medium**	MN w/ vit. B12
**Collector**	Vitor Vasconcelos	**Strain Status**	non-axenic, unicyanobacterial
**Collection Date**	2012	**Preservation type**	subculturing
**Location**	Brazil: Vermelha lagoon (Araruama system), in the state of Rio de Janeiro	**Light:dark cycle**	12:12-h
**Latitude & Longitude**	unknown	**Temperature (ºC)**	19
		**Light intensity**	10–30 µmol photons m−2 s−1
**References Publicly Available Articles**			
**References Publicly Available Theses**			

Strain ID	**unidentified filamentous cyanobacterium LEGE 12441**		
**Strain Taxonomy**			
**Microphotograph 400×**	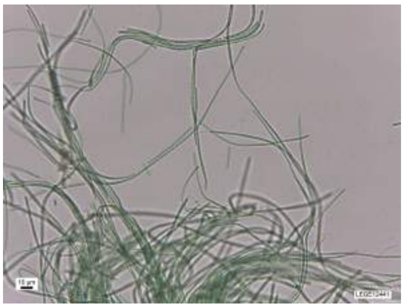	**Microphotograph 1000×**	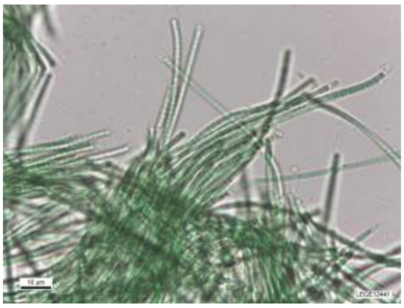
**Coidentity**	n.a.	**Morphometrics (Width × Length or Diameter) (µm)**	1,6±0,2 × 1,5±0,3
**Other Code(s)**		**Taxonomic notes/diacritical features**	forming fascicles; with necridic cells and hormogonia; with sheath
**Parent Sample**	EB4 - 6 A/1a	**Macroscopic growth features**	color: cyano; forming a firm biofilm
**Isolation Date**		**Environment**	aquatic, hypersaline
**Order (Older Classification Scheme)**	Oscillatoriales	**Habitat Sample Description**	upper layer of a microbial mat
**Order 2014 (Modern Taxonomy)**	undefined	**Ecophysiological traits**	
**Taxonomy Notes**	Pseudanabaenaceae cyanobacterium	**Notes on ecophysiological traits**	
**Accession Number(s)_16S**	ready to submit	**Isolator**	Ângela Pinheiro/Vitor Ramos
**Accession Number(s)_others (product/gene /region)**		**Medium**	Z8 25‰ TM sea salt w/ vit. B12
**Collector**	Vitor Vasconcelos	**Strain Status**	non-axenic, unicyanobacterial
**Collection Date**	2012	**Preservation type**	subculturing
**Location**	Brazil: Vermelha lagoon (Araruama system), in the state of Rio de Janeiro	**Light:dark cycle**	12:12-h
**Latitude & Longitude**	unknown	**Temperature (ºC)**	19
		**Light intensity**	10–30 µmol photons m−2 s−1
**References Publicly Available Articles**			
**References Publicly Available Theses**			

Strain ID	unidentified filamentous cyanobacterium LEGE XX061		
Strain Taxonomy			
Microphotograph 400×	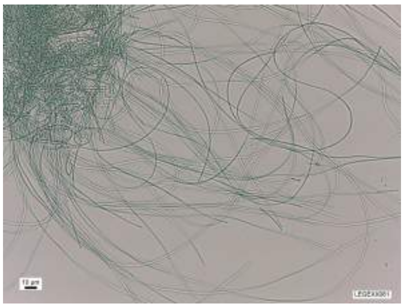	Microphotograph 1000×	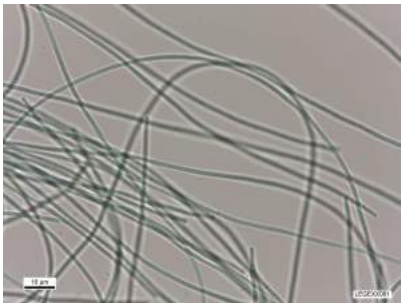
Coidentity	n.a.	Morphometrics (Width × Length or Diameter) (µm)	1,3±0,1 × 1,2±0,4
Other Code(s)		Taxonomic notes/diacritical features	
Parent Sample		Macroscopic growth features	color: cyano; forming a firm biofilm and tuft-like aggregates
Isolation Date		Environment	aquatic, freshwater
Order (Older Classification Scheme)	Oscillatoriales	Habitat Sample Description	water sample
Order 2014 (Modern Taxonomy)	undefined	Ecophysiological traits	
Taxonomy Notes	Pseudanabaenaceae cyanobacterium	Notes on ecophysiological traits	
Accession Number(s)_16S	KU951861	Isolator	Martin Saker
Accession Number(s)_others (product/gene /region)		Medium	Z8
Collector	Martin Saker	Strain Status	non-axenic, unicyanobacterial
Collection Date	unknown	Preservation type	subculturing
Location	Portugal: Maranhão dam reservoir, Avis	Light:dark cycle	12:12-h
Latitude & Longitude	unknown	Temperature (ºC)	19
		Light intensity	10–30 µmol photons m−2 s−1
References Publicly Available Articles			
References Publicly Available Theses			

Strain ID	unidentified filamentous cyanobacterium LEGE XX062		
**Strain Taxonomy**			
**Microphotograph 400×**	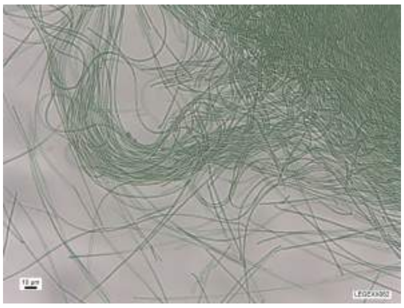	**Microphotograph 1000×**	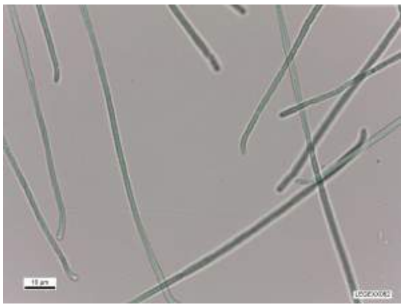
**Coidentity**	n.a.	**Morphometrics (Width × Length or Diameter) (µm)**	1,6±0,2 × 4,0±0,1
**Other Code(s)**		**Taxonomic notes/diacritical features**	often, the end of the trichome is distinctly hooked
**Parent Sample**		**Macroscopic growth features**	color: cyano; forming a firm biofilm
**Isolation Date**		**Environment**	unknown
**Order (Older Classification Scheme)**	Oscillatoriales	**Habitat Sample Description**	unknown
**Order 2014 (Modern Taxonomy)**	undefined	**Ecophysiological traits**	EPS production
**Taxonomy Notes**	unidentified Oscillatoriales	**Notes on ecophysiological traits**	
**Accession Number(s)_16S**	KU951862	**Isolator**	Martin Saker
**Accession Number(s)_others (product/gene /region)**		**Medium**	Z8
**Collector**	Martin Saker	**Strain Status**	non-axenic, unicyanobacterial
**Collection Date**	unknown	**Preservation type**	subculturing
**Location**	unknown	**Light:dark cycle**	12:12-h
**Latitude & Longitude**	unknown	**Temperature (ºC)**	19
		**Light intensity**	10–30 µmol photons m−2 s−1
**References Publicly Available Articles**			
**References Publicly Available Theses**			

**Strain ID**	**unidentified unicellular Chroococcales LEGE 06019**		
**Strain Taxonomy**			
**Microphotograph 400×**	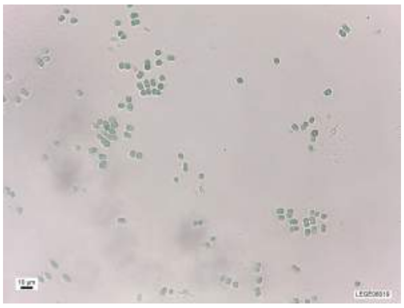	**Microphotograph 1000×**	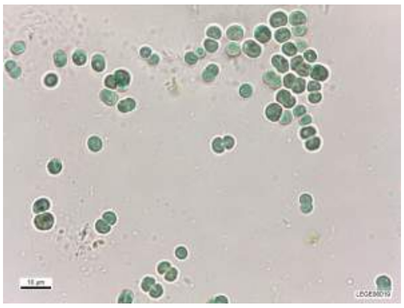
**Coidentity**	LEGE 06017	**Morphometrics (Width × Length or Diameter) (µm)**	Ø 4,3±0,5
**Other Code(s)**	strain 3, LEAN 113	**Taxonomic notes/diacritical features**	
**Parent Sample**	Ref. 3	**Macroscopic growth features**	color: cyano; forming aggregates
**Isolation Date**		**Environment**	aquatic, marine
**Order (Older Classification Scheme)**	Chroococcales	**Habitat Sample Description**	sea water sample, coastal, surf zone
**Order 2014 (Modern Taxonomy)**	Chroococcales	**Ecophysiological traits**	
**Taxonomy Notes**	Geminocystis-like sp.	**Notes on ecophysiological traits**	
**Accession Number(s)_16S**	KU951881	**Isolator**	Bárbara Frazão
**Accession Number(s)_others (product/gene /region)**		**Medium**	Z8 25‰ TM sea salt w/ vit. B12
**Collector**	Bárbara Frazão	**Strain Status**	non-axenic, unicyanobacterial
**Collection Date**	Fall 2006	**Preservation type**	subculturing, cryopreserved
**Location**	Portugal: Praia dos Coxos, Sto. Isidoro	**Light:dark cycle**	12:12-h
**Latitude & Longitude**	39.00455 N 9.425842 W	**Temperature (ºC)**	19
		**Light intensity**	10–30 µmol photons m−2 s−1
**References Publicly Available Articles**			
**References Publicly Available Theses**	Frazão B. (2008) MSc Dissertation, FCUP, University of Porto, Porto, Portugal		

Strain ID	unidentified unicellular Chroococcales LEGE 11426		
Strain Taxonomy			
Microphotograph 400×	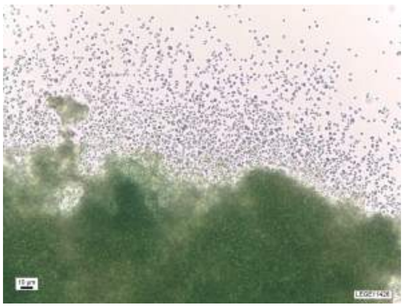	Microphotograph 1000×	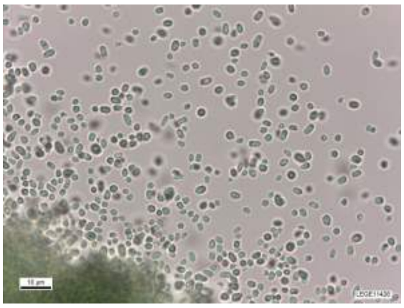
Coidentity	n.a.	Morphometrics (Width × Length or Diameter) (µm)	Ø 1,9±0,4 (Spherical Cells); 1,7±0,1 × 3,1±0,3 (rod-shaped cells)
Other Code(s)		Taxonomic notes/diacritical features	irregular to rod-shaped cells
Parent Sample	SC-IAi	Macroscopic growth features	color: cyano; forming tuft-like aggregates
Isolation Date		Environment	aquatic, marine
Order (Older Classification Scheme)	Chroococcales	Habitat Sample Description	subtidal sample, benthic (10-13m depth)
Order 2014 (Modern Taxonomy)	Chroococcales	Ecophysiological traits	
Taxonomy Notes		Notes on ecophysiological traits	
Accession Number(s)_16S	KU951883	Isolator	Sofia Costa
Accession Number(s)_others (product/gene /region)		Medium	MN w/ vit. B12
Collector	Pedro Leão/Aldo Barreiro	Strain Status	non-axenic, unicyanobacterial
Collection Date	Fall 2011	Preservation type	subculturing
Location	Portugal: less than 1 km off the shore, near Leixoes Harbour	Light:dark cycle	12:12-h
Latitude & Longitude	41.185809 N 8.719079 W	Temperature (ºC)	19
		Light intensity	10–30 µmol photons m−2 s−1
References Publicly Available Articles			
References Publicly Available Theses			

**Strain ID**	**unidentified unicellular Synechococcales LEGE 06307**		
**Strain Taxonomy**			
**Microphotograph 400×**	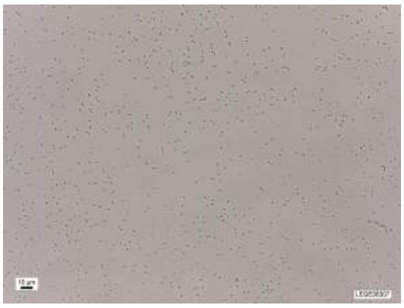	**Microphotograph 1000×**	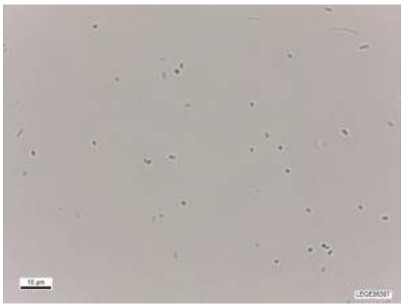
**Coidentity**	n.a.	**Morphometrics (Width × Length or Diameter) (µm)**	Ø 1,1±0,2 (spherical cells); 1,1± 0,1 × 1,7±0,1 (elongated cells)
**Other Code(s)**	IN69	**Taxonomic notes/diacritical features**	short rod-shaped cells; some elongated, involution cells observed
**Parent Sample**	n.d.	**Macroscopic growth features**	color: cyano; growing homogeneously; mucilaginous
**Isolation Date**		**Environment**	aquatic, brackish
**Order (Older Classification Scheme)**	Chroococcales	**Habitat Sample Description**	mesotidal zone, benthic
**Order 2014 (Modern Taxonomy)**	Synechococcales	**Ecophysiological traits**	EPS production
**Taxonomy Notes**		**Notes on ecophysiological traits**	
**Accession Number(s)_16S**	HM217055	**Isolator**	Viviana Lopes
**Accession Number(s)_others (product/gene /region)**		**Medium**	Z8
**Collector**	Viviana Lopes	**Strain Status**	non-axenic, unicyanobacterial
**Collection Date**	2006/11/23	**Preservation type**	subculturing
**Location**	Portugal: Minho estuary, Caminha	**Light:dark cycle**	12:12-h
**Latitude & Longitude**	41.86697 N 8.855833 W	**Temperature (ºC)**	19
		**Light intensity**	10–30 µmol photons m−2 s−1
**References Publicly Available Articles**	Lopes et al. (2012) Mar. Environ. Res. 73, 7–16		
**References Publicly Available Theses**	Lopes V.R. (2011) PhD Thesis, FCUP, University of Porto, Porto, Portugal		

Strain ID	unidentified unicellular Synechococcales LEGE 06316		
Strain Taxonomy			
Microphotograph 400×	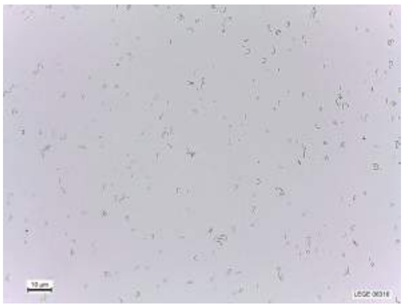	Microphotograph 1000×	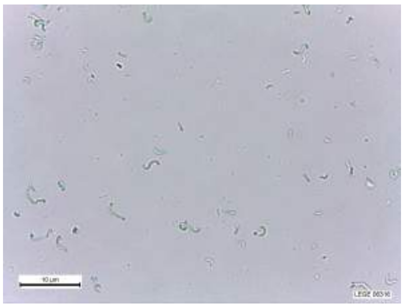
Coidentity	n.a.	Morphometrics (Width × Length or Diameter) (µm)	1,4±0,2 × 2,0±0,5
Other Code(s)	IN231106	Taxonomic notes/diacritical features	
Parent Sample	n.d.	Macroscopic growth features	color: cyano; growing homogeneously and also forming a firm biofilm
Isolation Date		Environment	aquatic, brackish
Order (Older Classification Scheme)	Chroococcales	Habitat Sample Description	water sample, on the estuary shore
Order 2014 (Modern Taxonomy)	Synechococcales	Ecophysiological traits	
Taxonomy Notes		Notes on ecophysiological traits	
Accession Number(s)_16S	HM217058	Isolator	Viviana Lopes
Accession Number(s)_others (product/gene /region)		Medium	Z8
Collector	Viviana Lopes	Strain Status	non-axenic, unicyanobacterial
Collection Date	2006/11/23	Preservation type	subculturing
Location	Portugal: Minho estuary, Caminha	Light:dark cycle	12:12-h
Latitude & Longitude	41.86697 N 8.855833 W	Temperature (ºC)	19
		Light intensity	10–30 µmol photons m−2 s−1
References Publicly Available Articles	Lopes et al. (2012) Mar. Environ. Res. 73, 7–16		
References Publicly Available Theses	Lopes V.R. (2011) PhD Thesis, FCUP, University of Porto, Porto, Portugal		

**Strain ID**	**unidentified unicellular Synechococcales LEGE 07074**		
**Strain Taxonomy**			
**Microphotograph 400×**	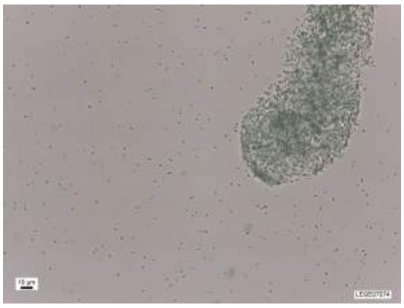	**Microphotograph 1000×**	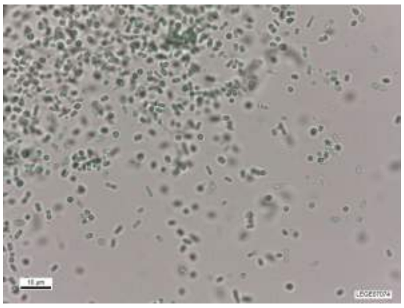
**Coidentity**	n.a.	**Morphometrics (Width × Length or Diameter) (µm)**	Ø 1,3±0,2 (Spherical Cells); 1,2±0,2 × 1,5±0,2 (Rod-shaped cells)
**Other Code(s)**		**Taxonomic notes/diacritical features**	rod-shaped cells; forming pseudofilaments; facultative sheath
**Parent Sample**	VL 206	**Macroscopic growth features**	color: cyano; growing homogeneously, but also forming aggregates
**Isolation Date**		**Environment**	aquatic, brackish
**Order (Older Classification Scheme)**	Chroococcales	**Habitat Sample Description**	mesotidal zone, benthic
**Order 2014 (Modern Taxonomy)**	Synechococcales	**Ecophysiological traits**	
**Taxonomy Notes**		**Notes on ecophysiological traits**	
**Accession Number(s)_16S**	HM217078	**Isolator**	Viviana Lopes
**Accession Number(s)_others (product/gene /region)**		**Medium**	Z8
**Collector**	Viviana Lopes	**Strain Status**	non-axenic, unicyanobacterial
**Collection Date**	Apr-2007	**Preservation type**	subculturing
**Location**	Portugal: Douro estuary, Porto	**Light:dark cycle**	12:12-h
**Latitude & Longitude**	41.14658 N 8.657972 W	**Temperature (ºC)**	19
		**Light intensity**	10–30 µmol photons m−2 s−1
**References Publicly Available Articles**	Lopes et al. (2010) Mar. Drugs 8, 471-482; Baptista et al. (2011) Toxicon 58, 410–414; Lopes et al. (2011) Toxicol. In Vitro 25, 944–950; Cianca et al. (2012) Amino Acids 42, 2473–2479; Lopes et al. (2012) Mar. Environ. Res. 73, 7–16		
**References Publicly Available Theses**	Lopes V.R. (2011) PhD Thesis, FCUP, University of Porto, Porto, Portugal		

Strain ID	**unidentified unicellular Synechococcales LEGE 08333**		
**Strain Taxonomy**			
**Microphotograph 400×**	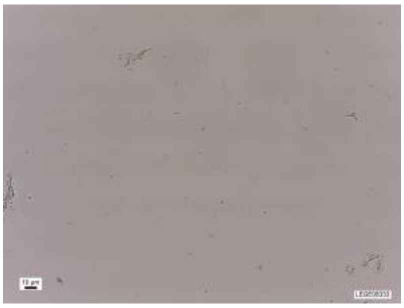	**Microphotograph 1000×**	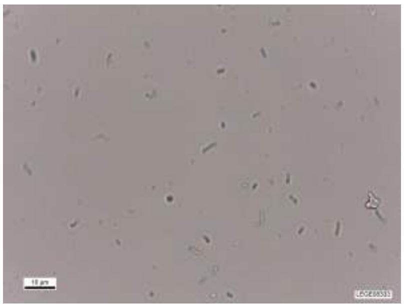
**Coidentity**	n.a.	**Morphometrics (Width × Length or Diameter) (µm)**	Ø 1,2±0,3 (Cells); 1,2±0,3 × 2,8±1,1 (Involution Cells)
**Other Code(s)**		**Taxonomic notes/diacritical features**	
**Parent Sample**		**Macroscopic growth features**	color: cyano; forming a smooth biofilm; mucilaginous
**Isolation Date**		**Environment**	aquatic, freshwater
**Order (Older Classification Scheme)**	Chroococcales	**Habitat Sample Description**	water sample
**Order 2014 (Modern Taxonomy)**	Synechococcales	**Ecophysiological traits**	EPS production
**Taxonomy Notes**		**Notes on ecophysiological traits**	
**Accession Number(s)_16S**	KU951884	**Isolator**	Vitor Vasconcelos
**Accession Number(s)_others (product/gene /region)**		**Medium**	Z8
**Collector**	Vitor Vasconcelos	**Strain Status**	non-axenic, unicyanobacterial
**Collection Date**	2008	**Preservation type**	subculturing
**Location**	Mexico: Lake Zumpango	**Light:dark cycle**	12:12-h
**Latitude & Longitude**	unknown	**Temperature (ºC)**	19
		**Light intensity**	10–30 µmol photons m−2 s−1
**References Publicly Available Articles**			
**References Publicly Available Theses**			

Strain ID	unidentified unicellular Synechococcales LEGE 09398		
Strain Taxonomy			
Microphotograph 400×	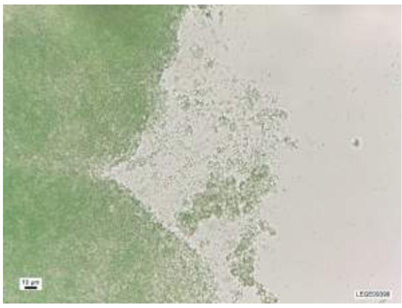	Microphotograph 1000×	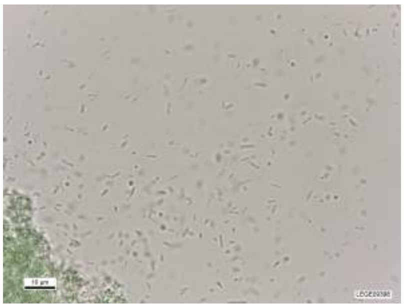
Coidentity	n.a.	Morphometrics (Width × Length or Diameter) (µm)	Ø 0,8±0,1 (Spherical Cells); 0,7±0,1 × 3,1±0,7 (Involution Cells); 0,8±0,1 ×1,4±0,4 (Elongated cells)
Other Code(s)		Taxonomic notes/diacritical features	some elongated, involution cells observed; several cell clusters
Parent Sample	Vela 1,2 um A	Macroscopic growth features	color: cyano; growing homogeneously, but also forming aggregates
Isolation Date		Environment	aquatic, freshwater
Order (Older Classification Scheme)	Chroococcales	Habitat Sample Description	water sample, from a pond
Order 2014 (Modern Taxonomy)	Synechococcales	Ecophysiological traits	
Taxonomy Notes		Notes on ecophysiological traits	
Accession Number(s)_16S	KU951885	Isolator	Vitor Ramos
Accession Number(s)_others (product/gene /region)		Medium	Z8
Collector	António J. Martins	Strain Status	non-axenic, unicyanobacterial
Collection Date	2009	Preservation type	subculturing
Location	Portugal: Lagoa da Vela, Mira	Light:dark cycle	12:12-h
Latitude & Longitude	unknown	Temperature (ºC)	19
		Light intensity	10–30 µmol photons m−2 s−1
References Publicly Available Articles			
References Publicly Available Theses			

Strain ID	unidentified unicellular Synechococcales LEGE 10387		
**Strain Taxonomy**			
**Microphotograph 400×**	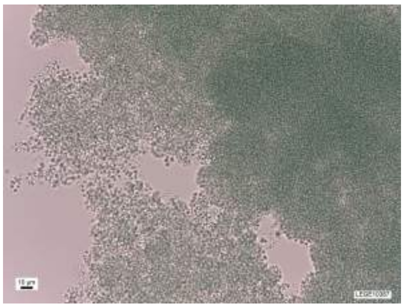	**Microphotograph 1000×**	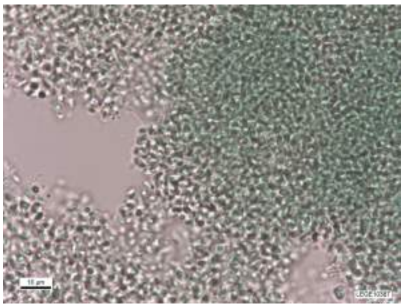
**Coidentity**	n.a.	**Morphometrics (Width × Length or Diameter) (µm)**	1,2±0,2 × 2,1±0,6 (Rod-shaped cells)
**Other Code(s)**		**Taxonomic notes/diacritical features**	rod-shaped cells; several cell clusters
**Parent Sample**	23C-1-A	**Macroscopic growth features**	color: cyano; forming aggregates
**Isolation Date**		**Environment**	aquatic, marine
**Order (Older Classification Scheme)**	Chroococcales	**Habitat Sample Description**	intertidal zone, from a dry dark mat, on a rock
**Order 2014 (Modern Taxonomy)**	Synechococcales	**Ecophysiological traits**	
**Taxonomy Notes**	Aphanothece-like sp., colonial?	**Notes on ecophysiological traits**	
**Accession Number(s)_16S**	KU951886	**Isolator**	Vitor Ramos
**Accession Number(s)_others (product/gene /region)**		**Medium**	MN w/ vit. B12
**Collector**	Vitor Ramos	**Strain Status**	non-axenic, unicyanobacterial
**Collection Date**	2010/04/28	**Preservation type**	subculturing, cryopreserved
**Location**	Portugal: Vila Nova de Mil Fontes	**Light:dark cycle**	12:12-h
**Latitude & Longitude**	37.719139 N 8.791014 W	**Temperature (ºC)**	19
		**Light intensity**	10–30 µmol photons m−2 s−1
**References Publicly Available Articles**			
**References Publicly Available Theses**			

References

Komárek, J. (2013) Cyanoprokaryota. 3. Teil/3rd Part: Heterocytous Genera, In Süsswasserflora von Mitteleuropa Bd. 19/2, Büdel, B., Gärtner, G., Krienitz, L. & Schager, l M. (eds.), Springer Spektrum. p. 1131

Komárek, J. & Anagnostidis, K. (1998) Cyanoprokaryota, 1. Teil/1st Part: Chroococcales, In Süsswasserflora von Mitteleuropa, Bd 19/1, Ettl, H., Gärtner, G., Heynig, H. & Mollenhauer, D. (eds.), Gustav Fischer Verlag. p. 548

Komárek, J. & Anagnostidis, K. (2005) Cyanoprokaryota. 2. Teil/2nd Part: Oscillatoriales, In Süsswasserflora von Mitteleuropa Bd. 19/2, Büdel, B., Krienitz, L., Gärtner, G. & Schagerl, M. (eds.), Elsevier/Spektrum. p. 759

Kotai, J. (1972) Instructions for preparation of modified nutrient solution Z8 for algae. Norwegian Institute for Water Research, Oslo, 11(69), 5

Rippka, R. (1988) Isolation and purification of cyanobacteria. *Methods in Enzymology*, 167, 3-27

Rippka, R., Waterbury J.B., Herdman M. & Castenholz R.W. (2001) Subsection II (formerly Pleurocapsales Geitler 1925, emend. Waterbury and Stanier 1978), in: Boone, D.R., Castenholz, R.W. (Eds.), Bergey's Manual of Systematic Bacteriology, vol. 1, 2nd ed., Springer, New York, pp. 514–539

Stewart, I., Carmichael, W. W., Sadler, R., McGregor, G. B., Reardon, K., Eaglesham, G. K., et al. (2009). Occupational and environmental hazard assessments for the isolation, purification and toxicity testing of cyanobacterial toxins. Environmental Health, 8(1), 52.

Waterbury, J.B. & Stanier, R.Y. (1978) Patterns of growth and development in pleurocapsalean cyanobacteria. *Microbiological Reviews*, 42(1), 2

Literature for LEGE Strains

Asterisks (*) indicate publications related with natural products production (e.g. studies on ecotoxicology, toxicity, bioactivity screening, isolation of compounds)

Afonso, T. (2014) Bioassay-guided isolation of cyanobacterial metabolites with anticancer activity, MSc Dissertation, FCUP, University of Porto, Porto, Portugal *

Allender, C.J., LeCleir, G.R., Rinta-Kanto, J.M., Small, R.L., Satchwell, M.F., Boyer, G.L. & Wilhelm, S.W. (2009) Identifying the source of unknown microcystin genes and predicting microcystin variants by comparing genes within uncultured cyanobacterial cells. *Applied and Environmental Microbiology*, 75(11), 3598-3604 *

Almeida, J.R., Freitas, M., Cruz, S., Leão, P.N., Vasconcelos, V. & Cunha, I. (2015) Acetylcholinesterase in Biofouling Species: Characterization and Mode of Action of Cyanobacteria-Derived Antifouling Agents. *Toxins*, 7(8), 2739-2756 *

Amorim, Á. & Vasconcelos, V. (1999) Dynamics of microcystins in the mussel *Mytilus galloprovincialis*. *Toxicon*, 37(7), 1041-1052 *

Amorim, A. (1997) Acumulação de microcistinas por *Mytilus galloprovinvialis* Lamarck, MSc Dissertation, FCUP, University of Porto, Porto, Portugal [in Portuguese] *

Antunes, J.T., Leão, P.N. & Vasconcelos, V.M. (2012) Influence of biotic and abiotic factors on the allelopathic activity of the cyanobacterium *Cylindrospermopsis raciborskii* strain LEGE 99043. *Microbial Ecology*, 64(3), 584-592 *

Antunes, J. (2011) Allelopathic activity of the cyanobacterium *Cylindrospermopsis raciborskii*, MSc Dissertation, FCUP, University of Porto, Porto, Portugal *

Azevedo, C.C., Azevedo, J., Osório, H., Vasconcelos, V. & Campos, A. (2014) Early physiological and biochemical responses of rice seedlings to low concentration of microcystin-LR. *Ecotoxicology*, 23(2), 107-121 *

Banker, R., Carmeli, S., Hadas, O., Teltsch, B., Porat, R. & Sukenik, A. (1997) Identification of cylindrospermopsin in *Aphanizomenon ovalisporum* (Cyanophyceae) isolated from Lake Kinneret, Israel. *Journal of Phycology*, 33(4), 613-616 *

Baptista, M.S., Cianca, R.C., Lopes, V.R., Almeida, C.M.R. & Vasconcelos, V.M. (2011) Determination of the non protein amino acid β-N-methylamino-L-alanine in estuarine cyanobacteria by capillary electrophoresis. *Toxicon*, 58(5), 410-414 *

Baptista, M.S., Vasconcelos, R.G., Ferreira, P.C., Almeida, C.M.R. & Vasconcelos, V.M. (2015) Assessment of the non-protein amino acid BMAA in Mediterranean mussel *Mytilus galloprovincialis* after feeding with estuarine cyanobacteria. *Environmental Science and Pollution Research*, 22(16), 12501-12510 *

Barreiro, A. & Vasconcelos, V.M. (2014) Interactions between allelopathic properties and growth kynetics in four freshwater phytoplankton species studied by model simulations. *Aquatic Ecology*, 48(2), 191-205

Barros, P. (2001) Efeitos tóxicos de cianobactérias em Cladóceros, MSc Dissertation, FCUP, University of Porto, Porto, Portugal [in Portuguese] *

Berry, J.P., Gibbs, P.D., Schmale, M.C. & Saker, M.L. (2009) Toxicity of cylindrospermopsin, and other apparent metabolites from *Cylindrospermopsis raciborskii* and *Aphanizomenon ovalisporum*, to the zebrafish (Danio rerio) embryo. *Toxicon*, 53(2), 289-299 *

Boas, E. (1999) A Influência da temperatura, da intensidade luminosa e de coagulantes/floculantes na libertação e degradação de microcistinas por *Microcystis aeruginosa* Kützing ex Lemmermann, MSc Dissertation, FCUP, University of Porto, Porto, Portugal [in Portuguese] *

Brito, Â., Ramos, V., Seabra, R., Santos, A., Santos, C.L., Lopo, M., et al. (2012) Culture-dependent characterization of cyanobacterial diversity in the intertidal zones of the Portuguese coast: A polyphasic study. *Systematic and Applied Microbiology*, 35(2), 110-119 *

Brito, Â., Gaifem, J., Ramos, V., Glukhov, E., Dorrestein, P.C., Gerwick, W.H., et al. (2015) Bioprospecting Portuguese Atlantic coast cyanobacteria for bioactive secondary metabolites reveals untapped chemodiversity. *Algal Research*, 9, 218-226 *

Brito, Â., Ramos, V., Mota, R., Lima, S., Santos, A., Vieira, J., et al. (2017) Description of new genera and species of marine cyanobacteria from the Portuguese Atlantic coast. *Molecular Phylogenetics and Evolution*, 111, 18-34

Caetano, C. (2014) Expressão proteica diferencial das enzimas de destoxificação glutationa transferases em *Mytilus Galloprovincialis* após exposição a microcistinas, MSc Dissertation, ICBAS, University of Porto, Porto, Portugal [in Portuguese] *

Campos, A., Araújo, P., Pinheiro, C., Azevedo, J., Osório, H. & Vasconcelos, V. (2013) Effects on growth, antioxidant enzyme activity and levels of extracellular proteins in the green alga *Chlorella vulgaris* exposed to crude cyanobacterial extracts and pure microcystin and cylindrospermopsin. *Ecotoxicology and Environmental Safety*, 94, 45-53.*

Carneiro, M., Reis, B., Azevedo, J., Campos, A., Osório, H., Vasconcelos, V. & Martins, J.C. (2015) Glutathione transferases responses induced by microcystin-LR in the gills and hepatopancreas of the clam *Venerupis philippinarum*. *Toxins*, 7(6), 2096-2120.*

Chaves, R. (2013) Detection of ribosomal peptides in cyanobacterial strains, MSc Dissertation, ICBAS, University of Porto, Porto, Portugal *

Cianca, R.C.C., Baptista, M.S., Lopes, V.R. & Vasconcelos, V.M. (2012) The non-protein amino acid β-N-methylamino-l-alanine in Portuguese cyanobacterial isolates. *Amino Acids*, 42(6), 2473-2479 *

Cianca, R.C.C., Baptista, M.S., da Silva, L.P., Lopes, V.R. & Vasconcelos, V.M. (2012) Reversed-phase HPLC/FD method for the quantitative analysis of the neurotoxin BMAA (β-N-methylamino-l-alanine) in cyanobacteria. *Toxicon*, 59(3), 379-384 *

Costa, M., Garcia, M., Costa-Rodrigues, J., Costa, M.S., Ribeiro, M.J., Fernandes, M.H., et al. (2013) Exploring bioactive properties of marine cyanobacteria isolated from the Portuguese coast: high potential as a source of anticancer compounds. *Marine Drugs*, 12(1), 98-114 *

Costa, M.S., Costa, M., Ramos, V., Leão, P.N., Barreiro, A., Vasconcelos, V. & Martins, R. (2015) Picocyanobacteria from a clade of marine cyanobium revealed bioactive potential against microalgae, bacteria, and marine invertebrates. *Journal of Toxicology and Environmental Health, Part A*, 78(7), 432-442 *

Costa, M.S., Rego, A., Ramos, V., Afonso, T.B., Freitas, S., Preto, M., et al. (2016) The conifer biomarkers dehydroabietic and abietic acids are widespread in Cyanobacteria. *Scientific Reports*, 6, 23436 *

Costa, M.S. (2011) Cianobactérias marinhas: toxicidade face a fitoplâncton e invertebrados marinhos; toxicidade face a bactérias; citotoxicidade em linhagens celulares tumorais e normais humanas, MSc Dissertation, ICBAS and FCUP, University of Porto, Porto, Portugal [in Portuguese] *

Couto, M.S. (2015) Molecular and biochemical mechanisms involved in the toxicity of a marine cyanobacteria compound on cancer cell lines, MSc Dissertation, ESTSP, Polytechnic Institute of Porto, Porto, Portugal *

de Matos, A. (2014) Development of molecular tools for the early warning of potentially toxic cyanobacteria, MSc Dissertation, FCUP, University of Porto, Porto, Portugal *

de Morais, P., Stoichev, T., Basto, M.C.P., Ramos, V., Vasconcelos, V.M. & Vasconcelos, M.T.S. (2014) Pentachlorophenol toxicity to a mixture of *Microcystis aeruginosa* and *Chlorella vulgaris* cultures. *Aquatic Toxicology*, 150, 159-164.

de Morais, P., Stoichev, T., Basto, M.C.P., Ramos, V., Vasconcelos, V.M. & Vasconcelos, M.T.S. (2014) Cyanobacterium *Microcystis aeruginosa* response to pentachlorophenol and comparison with that of the microalga *Chlorella vulgaris*. *Water Research*, 52, 63-72.

de Morais, P. (2013) Fate and effects of chlorophenols to phytoplankton species from Portuguese freshwaters, PhD Thesis, FCUP, University of Porto, Porto, Portugal

Devlin, S., Meneely, J.P., Greer, B., Greef, C., Lochhead, M.J. & Elliott, C.T. (2013) Next generation planar waveguide detection of microcystins in freshwater and cyanobacterial extracts, utilising a novel lysis method for portable sample preparation and analysis. *Analytica Chimica Acta*, 769, 108-113 *

dos Santos, C. (2014) Bioacumulação de cianotoxinas em microalgas e plantas: Avaliação de risco de cianobactérias tóxicas, MSc Dissertation, FCUP, University of Porto, Porto, Portugal [in Portuguese] *

Dvořák, P., Casamatta, D. A., Poulíčková, A., Hašler, P., Ondřej, V. & Sanges, R. (2014) *Synechococcus*: 3 billion years of global dominance. *Molecular Ecology*, 23(22), 5538-5551

Fernandes, E. (2001) Avaliação da toxicidade de cianobactérias para *Brachydanio rerio* utilizando ensaios a diferentes níveis de organização biológica, MSc Dissertation, FCUP, University of Porto, Porto, Portugal [in Portuguese] *

Fernandes, S., Welker, M. & Vasconcelos, V.M. (2009) Changes in the GST activity of the mussel *Mytilus galloprovincialis* during exposure and depuration of microcystins. *Journal of Experimental Zoology Part A: Ecological Genetics and Physiology*, 311(3), 226-230 *

Fraga, M., Vilariño, N., Louzao, M.C., Rodríguez, L.P., Alfonso, A., Campbell, K., et al. (2014) Multi-detection method for five common microalgal toxins based on the use of microspheres coupled to a flow-cytometry system. *Analytica Chimica Acta*, 850, 57-64 *

Frazão, B. (2008) Ocorrência e ecotoxicologia de cianobactérias marinhas, MSc Dissertation, FCUP, University of Porto, Porto, Portugal Portugal [in Portuguese] *

Frazão, B., Martins, R. & Vasconcelos, V. (2010) Are known cyanotoxins involved in the toxicity of picoplanktonic and filamentous North Atlantic marine cyanobacteria?. *Marine Drugs*, 8(6), 1908-1919 *

Freitas, M., Azevedo, J., Carvalho, A.P., Campos, A. & Vasconcelos, V. (2014) Effects of storage, processing and proteolytic digestion on microcystin-LR concentration in edible clams. *Food and Chemical Toxicology*, 66, 217-223 *

Freitas, M., Azevedo, J., Pinto, E., Neves, J., Campos, A. & Vasconcelos, V. (2015) Effects of microcystin-LR, cylindrospermopsin and a microcystin-LR/cylindrospermopsin mixture on growth, oxidative stress and mineral content in lettuce plants (*Lactuca sativa* L.). *Ecotoxicology and Environmental Safety*, 116, 59-67 *

Freitas, M., Campos, A., Azevedo, J., Barreiro, A., Planchon, S., Renaut, J. & Vasconcelos, V. (2015) Lettuce (*Lactuca sativa* L.) leaf-proteome profiles after exposure to cylindrospermopsin and a microcystin-LR/cylindrospermopsin mixture: a concentration-dependent response. *Phytochemistry*, 110, 91-103 *

Freitas, M. (2014) Effects of interaction, availability and bioaccessibility of microcystin-LR and cylindrospermopsin in terrestrial and aquatic species, PhD Thesis, FCUP, University of Porto, Porto, Portugal *

Freitas, S. (2013) Toxicological assessment of marine cyanobacterial extracts in human tumor cell lines – Proteomic and gene expression approach, MSc Dissertation, ICBAS, University of Porto, Porto, Portugal *

Garcia, C. (2001) Isolamento, cultura e avaliação de toxicidade de estirpes de cianobactérias da lagoa de Mira, MSc Dissertation, FCUP, University of Porto, Porto, Portugal [in Portuguese] *

Gomes, D. (2013) Pesquisa de cianobactérias, cianotoxinas e bactérias patogénicas em suplementos alimentares (*Chlorella* e *Spirulina*), MSc Dissertation, ICBAS, University of Porto, Porto, Portugal [in Portuguese] *

Gutiérrez-Praena, D., Campos, A., Azevedo, J., Neves, J., Freitas, M., Guzmán-Guillén, R., et al. (2014) Exposure of *Lycopersicon Esculentum* to microcystin-LR: Effects in the leaf proteome and toxin translocation from water to leaves and fruits. *Toxins*, 6(6), 1837-1854 *

Hisbergues, M., Christiansen, G., Rouhiainen, L., Sivonen, K. & Börner, T. (2003) PCR-based identification of microcystin-producing genotypes of different cyanobacterial genera. *Archives of Microbiology*, 180(6), 402-410 *

Jokela, J., Herfindal, L., Wahlsten, M., Permi, P., Selheim, F., Vasconçelos, V., et al. (2010) A novel cyanobacterial nostocyclopeptide is a potent antitoxin against microcystins. *ChemBioChem*, 11(11), 1594-1599 *

Koskenniemi, K., Lyra, C., Rajaniemi-Wacklin, P., Jokela, J. & Sivonen, K. (2007) Quantitative real-time PCR detection of toxic *Nodularia* cyanobacteria in the Baltic Sea. *Applied and Environmental Microbiology*, 73(7), 2173-2179 *

Leão, P.N., Vasconcelos, M.T.S. & Vasconcelos, V.M. (2009) Allelopathic activity of cyanobacteria on green microalgae at low cell densities. *European Journal of Phycology*, 44(3), 347-355 *

Leão, P.N., Pereira, A.R., Liu, W.T., Ng, J., Pevzner, P.A., Dorrestein, P.C., et al. (2010) Synergistic allelochemicals from a freshwater cyanobacterium. *Proceedings of the National Academy of Sciences*, 107(25), 11183-11188 *

Leão, P.N., Ramos, V., Vale, M., Machado, J.P. & Vasconcelos, V.M. (2012) Microbial community changes elicited by exposure to cyanobacterial allelochemicals. *Microbial Ecology*, 63(1), 85-95 *

Leão, P.N., Ramos, V., Gonçalves, P.B., Viana, F., Lage, O.M., Gerwick, W.H. & Vasconcelos, V.M. (2013) Chemoecological screening reveals high bioactivity in diverse culturable portuguese marine cyanobacteria. *Marine Drugs*, 11(4), 1316-1335 *

Leão, P.N., Costa, M., Ramos, V., Pereira, A.R., Fernandes, V.C., Domingues, V.F., et al. (2013) Antitumor activity of hierridin B, a cyanobacterial secondary metabolite found in both filamentous and unicellular marine strains. *PloS One*, 8(7), e69562 *

Leão, P.N., Nakamura, H., Costa, M., Pereira, A.R., Martins, R., Vasconcelos, V., et al. (2016) Biosynthesis-Assisted Structural Elucidation of the Bartolosides, Chlorinated Aromatic Glycolipids from Cyanobacteria. *Angewandte Chemie International Edition*, 127, 11215-11219 *

Leão, P.N. (2010) Allelopathic properties of cyanobacteria isolated from Portuguese freshwaters, PhD Thesis, FCUP, University of Porto, Porto, Portugal *

Leikoski, N., Fewer, D.P., Jokela, J., Alakoski, P., Wahlsten, M. & Sivonen, K. (2012) Analysis of an inactive cyanobactin biosynthetic gene cluster leads to discovery of new natural products from strains of the genus Microcystis. *PLoS One*, 7(8), e43002 *

Lopes, V.R. & Vasconcelos, V.M. (2011) Bioactivity of benthic and picoplanktonic estuarine cyanobacteria on growth of photoautotrophs: inhibition versus stimulation. *Marine Drugs*, 9(5), 790-802 *

Lopes, V.R., Fernández, N., Martins, R.F. & Vasconcelos, V. (2010) Primary screening of the bioactivity of brackishwater cyanobacteria: Toxicity of crude extracts to *Artemia salina* larvae and *Paracentrotus lividus* embryos. *Marine Drugs*, 8(3), 471-482 *

Lopes, V.R., Schmidtke, M., Fernandes, M.H., Martins, R. & Vasconcelos, V. (2011) Cytotoxicity in L929 fibroblasts and inhibition of herpes simplex virus type 1 Kupka by estuarine cyanobacteria extracts. *Toxicology in Vitro*, 25(4), 944-950 *

Lopes, V.R., Ramos, V., Martins, A., Sousa, M., Welker, M., Antunes, A. & Vasconcelos, V.M. (2012) Phylogenetic, chemical and morphological diversity of cyanobacteria from Portuguese temperate estuaries. *Marine Environmental Research*, 73, 7-16 *

Lopes, V.R. (2011) Screening, isolation and characterization of bioactive substances of estuarine cyanobacteria, PhD Thesis, FCUP, University of Porto, Porto, Portugal *

Machado, J. (2014) Implicações do uso de água contaminada com microcistina na qualidade de *Daucus carota* (Cenoura), MSc Dissertation, ICBAS, University of Porto, Porto, Portugal [in Portuguese] *

Macieira, R. (2013) Propriedades antibióticas de cianobactérias marinhas em busca de novos antibióticos, MSc Dissertation, FCUP, University of Porto, Porto, Portugal [in Portuguese] *

Martins, R., Pereira, P., Welker, M., Fastner, J. & Vasconcelos, V.M. (2005) Toxicity of culturable cyanobacteria strains isolated from the Portuguese coast. *Toxicon*, 46(4), 454-464 *

Martins, R., Fernandez, N., Beiras, R. & Vasconcelos, V. (2007) Toxicity assessment of crude and partially purified extracts of marine *Synechocystis* and *Synechococcus* cyanobacterial strains in marine invertebrates. *Toxicon*, 50(6), 791-799 *

Martins, R.F., Ramos, M.F., Herfindal, L., Sousa, J.A., Skærven, K. & Vasconcelos, V.M. (2008) Antimicrobial and cytotoxic assessment of marine cyanobacteria-*Synechocystis* and *Synechococcus*. *Marine Drugs*, 6(1), 1-11 *

Martins, J., Saker, M.L., Moreira, C., Welker, M., Fastner, J. & Vasconcelos, V.M. (2009) Peptide diversity in strains of the cyanobacterium *Microcystis aeruginosa* isolated from Portuguese water supplies. *Applied Microbiology and Biotechnology*, 82(5), 951-961 *

Martins, J.C., Leão, P.N. & Vasconcelos, V. (2009) Differential protein expression in *Corbicula fluminea* upon exposure to a *Microcystis aeruginosa* toxic strain. *Toxicon*, 53(4), 409-416 *

Martins, J.C., Machado, J., Martins, A., Azevedo, J., OlivaTeles, L. & Vasconcelos, V. (2011) Dynamics of protein phosphatase gene expression in *Corbicula fluminea* exposed to microcystin-LR and to toxic *Microcystis aeruginosa* cells. *International Journal of Molecular Sciences*, 12(12), 9172-9188 *

Martins, A., Moreira, C., Vale, M., Freitas, M., Regueiras, A., Antunes, A. & Vasconcelos, V. (2011). Seasonal dynamics of *Microcystis* spp. and their toxigenicity as assessed by qPCR in a temperate reservoir. *Marine Drugs*, 9(10), 1715-1730 *

Martins, J., Leão, P.N., Ramos, V. & Vasconcelos, V. (2013) N-terminal protease gene phylogeny reveals the potential for novel cyanobactin diversity in cyanobacteria. *Marine Drugs*, 11(12), 4902-4916 *

Martins, J. (2008) Ocorrência, diversidade e toxicidade de cianobactérias numa ETAR. Impacto na comunidade microbiana, MSc Dissertation, FFUP, University of Porto, Porto, Portugal [in Portuguese] *

Martins, J.C. (2010) Metabolization and bioavailability of cyanobacterial hepatotoxins - microcystins – in contaminated organisms, PhD Thesis, FCUP, University of Porto, Porto, Portugal *

Martins, R. (2005) Ecotoxicology of marine cyanobacteria from the Portuguese coast, PhD Thesis, FCUP, University of Porto, Porto, Portugal *

Mendes, R.A. (2014) Cyanotoxin detection in freshwater systems from the North and Center of Portugal, MSc Dissertation, FCUP, University of Porto, Porto, Portugal *

Metcalf, J.S., Beattie, K.A., Saker, M.L. & Codd, G.A. (2002) Effects of organic solvents on the high performance liquid chromatographic analysis of the cyanobacterial toxin cylindrospermopsin and its recovery from environmental eutrophic waters by solid phase extraction. *FEMS Microbiology Letters*, 216(2), 159-164 *

Monteiro, M.B. (2011) *Azolla-Anabaena*: simbiose na fitorremediação de água com microcistinas, MSc Dissertation, FCUP, University of Porto, Porto, Portugal [in Portuguese] *

Monteiro, M.I. (2015) Molecular mechanisms of BMAA stress-response and detoxification in *Mytilus galloprovincialis*, MSc Dissertation, FCUP, University of Porto, Porto, Portugal *

Morais, J., Augusto, M., Carvalho, A.P., Vale, M. & Vasconcelos, V.M. (2008) Cyanobacteria hepatotoxins, microcystins: bioavailability in contaminated mussels exposed to different environmental conditions. *European Food Research and Technology*, 227(3), 949 *

Moreira, C., Martins, A., Azevedo, J., Freitas, M., Regueiras, A., Vale, M., et al. (2011) Application of real-time PCR in the assessment of the toxic cyanobacterium *Cylindrospermopsis raciborskii* abundance and toxicological potential. *Applied Microbiology and Biotechnology*, 92(1), 189-197 *

Moreira, C., Fathalli, A., Vasconcelos, V. & Antunes, A. (2011) Genetic diversity and structure of the invasive toxic cyanobacterium *Cylindrospermopsis raciborskii*. *Current Microbiology*, 62(5), 1590-1595

Moreira, C., Spillane, C., Fathalli, A., Vasconcelos, V. & Antunes, A. (2014) African origin and Europe-Mediated global dispersal of The cyanobacterium *Microcystis aeruginosa*. *Current Microbiology*, 69(5), 628-633

Moreira, C., Fathalli, A., Vasconcelos, V. & Antunes, A. (2015) Phylogeny and biogeography of the invasive cyanobacterium *Cylindrospermopsis raciborskii*. *Archives of Microbiology*, 197(1), 47-52 *

Neilan, B.A., Saker, M.L., Fastner, J., Törökné, A. & Burns, B.P. (2003) Phylogeography of the invasive cyanobacterium *Cylindrospermopsis raciborskii*. *Molecular Ecology*, 12(1), 133-140*

Nogueira, I.C., Saker, M.L., Pflugmacher, S., Wiegand, C. & Vasconcelos, V. M. (2004) Toxicity of the cyanobacterium *Cylindrospermopsis raciborskii* to *Daphnia magna*. *Environmental Toxicology*, 19(5), 453-459 *

Osswald, J., Rellán, S., Gago, A. & Vasconcelos, V. (2007) Toxicology and detection methods of the alkaloid neurotoxin produced by cyanobacteria, anatoxin-a. *Environment International*, 33(8), 1070-1089 *

Osswald, J., Rellán, S., Gago, A. & Vasconcelos, V. (2008) Uptake and depuration of anatoxin-a by the mussel *Mytilus galloprovincialis* (Lamarck, 1819) under laboratory conditions. *Chemosphere*, 72(9), 1235-1241*

Osswald, J., Rellán, S., Gago-Martinez, A. & Vasconcelos, V. (2009) Production of anatoxin-a by cyanobacterial strains isolated from Portuguese fresh water systems. *Ecotoxicology*, 18(8), 1110-1115 *

Osswald, J. (2002) Estudo da toxicidade de cianobactérias diazotróficas da Albufeira do Maranhão (Portugal), MSc Dissertation, FCUP, University of Porto, Porto, Portugal [in Portuguese] *

Osswald, J. (2007) Occurrence of anatoxin-a producing cyanobacterial and evaluation of its public health risk, in Portugal, PhD Thesis, FCUP, University of Porto, Porto, Portugal *

Pereira, S., Saker, M.L., Vale, M. & Vasconcelos, V.M. (2009) Comparison of sensitivity of grasses (*Lolium perenne* L. and *Festuca rubra* L.) and lettuce (*Lactuca sativa* L.) exposed to water contaminated with microcystins. *Bulletin of Environmental Contamination and Toxicology*, 83(1), 81-84 *

Pereira, A.L., Monteiro, B., Azevedo, J., Campos, A., Osório, H. & Vasconcelos, V. (2015) Effects of the naturally-occurring contaminant microcystins on the *Azolla filiculoides–Anabaena azollae* symbiosis. *Ecotoxicology and Environmental Safety*, 118, 11-20 *

Pinheiro, C., Azevedo, J., Campos, A., Loureiro, S. & Vasconcelos, V. (2013) Absence of negative allelopathic effects of cylindrospermopsin and microcystin-LR on selected marine and freshwater phytoplankton species. *Hydrobiologia*, 705(1), 27-42 *

Pinheiro, C., Azevedo, J., Campos, A., Vasconcelos, V. & Loureiro, S. (2016) The interactive effects of microcystin-LR and cylindrospermopsin on the growth rate of the freshwater algae *Chlorella vulgaris*. *Ecotoxicology*, 25(4), 745-758 *

Pinho, L.X., Azevedo, J., Brito, Â., Santos, A., Tamagnini, P., Vilar, V.J., et al. (2015) Effect of TiO 2 photocatalysis on the destruction of *Microcystis aeruginosa* cells and degradation of cyanotoxins microcystin-LR and cylindrospermopsin. *Chemical Engineering Journal*, 268, 144-152

Pinho, L. (2014) Photocatalytic degradation of cyanobacteria and cyanotoxins using suspended and immobilized TiO2, PhD Thesis, FEUP, University of Porto, Porto, Portugal *

Puerto, M., Campos, A., Prieto, A., Cameán, A., de Almeida, A.M., Coelho, A.V. & Vasconcelos, V. (2011) Differential protein expression in two bivalve species - *Mytilus galloprovincialis* and *Corbicula fluminea* - exposed to *Cylindrospermopsis raciborskii* cells. *Aquatic Toxicology*, 101(1), 109-116 *

Ramos, V., Seabra, R., Brito, Â., Santos, A., Santos, C.L., Lopo, M, et al. (2010) Characterization of an intertidal cyanobacterium that constitutes a separate clade together with thermophilic strains. *European Journal of Phycology*, 45(4), 394-403

Rantala, A., Fewer, D.P., Hisbergues, M., Rouhiainen, L., Vaitomaa, J., Börner, T. & Sivonen, K. (2004) Phylogenetic evidence for the early evolution of microcystin synthesis. *Proceedings of the National Academy of Sciences of the United States of America*, 101(2), 568-573 (Table S1) *

Rantala-Ylinen, A., Känä, S., Wang, H., Rouhiainen, L., Wahlsten, M., Rizzi, E., et al. (2011) Anatoxin-a synthetase gene cluster of the cyanobacterium *Anabaena* sp. strain 37 and molecular methods to detect potential producers. *Applied and Environmental Microbiology*, 77(20), 7271-7278 *

Rastoll, M.J., Ouahid, Y., Martín-Gordillo, F., Ramos, V., Vasconcelos, V. & Del Campo, F.F. (2013) The development of a cryopreservation method suitable for a large cyanobacteria collection. *Journal of Applied Phycology*, 25(5), 1483-1493

Regueiras, A. (2009) Comparação de métodos de identificação e quantificação de cianobactérias e suas toxinas na Albufeira do Torrão (Rio Tâmega), MSc Dissertation, ICBAS and FCUP, University of Porto, Porto, Portugal [in Portuguese] *

Reis, B. (2014) Transcriptional responses of glutathione transferase genes in *Ruditapes philippinarum* exposed to microcystin-LR: Expression patterns and biomarker discovery, MSc Dissertation, FFUP, University of Porto, Porto, Portugal *

Reis, B., Carneiro, M., Machado, J., Azevedo, J., Vasconcelos, V. & Martins, J.C. (2015) Transcriptional responses of glutathione transferase genes in *Ruditapes philippinarum* exposed to microcystin-LR. *International Journal of Molecular Sciences*, 16(4), 8397-8414 *

Ribeiro, J.P.N., Lopes, J.A. & Fachini, A. (2008) Cleansing contaminated seawaters using marine cyanobacteria: evaluation of trace metal removal from the medium. *International Journal of Environmental and Analytical Chemistry*, 88(10), 701-710

Ribeiro, M.J. (2012) Marine cyanobacteria: evaluation of the anticancer potential, MSc Dissertation, Department of Chemistry, University of Aveiro, Aveiro, Portugal *

Ribeiro, T.A. (2014) Bioatividade de portoamidas em células de cancro: mecanismo molecular, MSc Dissertation, Department of Chemistry, University of Aveiro, Aveiro, Portugal [in Portuguese] *

Saker, M.L. & Eaglesham, G.K. (1999) The accumulation of cylindrospermopsin from the cyanobacterium *Cylindrospermopsis raciborskii* in tissues of the Redclaw crayfish *Cherax quadricarinatus*. *Toxicon*, 37(7), 1065-1077 *

Saker, M.L. & Griffiths, D.J. (2000) The effect of temperature on growth and cylindrospermopsin content of seven isolates of *Cylindrospermopsis raciborskii* (Nostocales, Cyanophyceae) from water bodies in northern Australia. *Phycologia*, 39(4), 349-354 *

Saker, M.L. & Neilan, B.A. (2001) Varied diazotrophies, morphologies, and toxicities of genetically similar isolates of *Cylindrospermopsis raciborskii* (Nostocales, Cyanophyceae) from northern Australia. *Applied and Environmental Microbiology*, 67(4), 1839-1845 *

Saker, M.L., Nogueira, I.C., Vasconcelos, V.M., Neilan, B.A., Eaglesham, G.K. & Pereira, P. (2003) First report and toxicological assessment of the cyanobacterium *Cylindrospermopsis raciborskii* from Portuguese freshwaters. *Ecotoxicology and Environmental Safety*, 55(2), 243-250 *

Saker, M.L., Nogueira, I.C. & Vasconcelos, V.M. (2003) Distribution and toxicity of *Cylindrospermopsis raciborskii* (Cyanobacteria) in Portuguese freshwaters. *Limnetica*, 22(3), 129-136 *

Saker, M.L., Neilan, B.A., Fastner, J., Törökné, A. & Burns, B.P. (2003) Phylogeography of the invasive cyanobacterium *Cylindrospermopsis raciborskii*. *Molecular Ecology*, 12(1), 133-140*

Saker, M.L., Nogueira, I.C. & Vasconcelos, V.M. (2004) Distribution and toxicity of *Cylindrospermopsis raciborskii* (Cyanobacteria) in Portuguese freshwaters. *Limnetica*, 23(1-2), 145-152 *

Saker, M.L., Vale, M., Kramer, D. & Vasconcelos, V.M. (2007) Molecular techniques for the early warning of toxic cyanobacteria blooms in freshwater lakes and rivers. *Applied Microbiology and Biotechnology*, 75(2), 441-449 *

Saker, M.L. (2000) Cyanobacterial blooms in tropical north Queensland water bodies, PhD Thesis, James Cook University, Townsville, Australia *

Sanchez, J.A., Otero, P., Alfonso, A., Ramos, V., Vasconcelos, V., Aráoz, R., et al. (2014) Detection of anatoxin-a and three analogs in *Anabaena* spp. cultures: New fluorescence polarization assay and toxin profile by LC-MS/MS. *Toxins*, 6(2), 402-415 *

Selheim, F., Herfindal, L., Martins, R., Vasconcelos, V. & Døskeland, S.O. (2005) Neuro-apoptogenic and blood platelet targeting toxins in benthic marine cyanobacteria from the Portuguese coast. *Aquatic Toxicology*, 74(4), 294-306 *

Silva, P. & Vasconcelos, V. (2010) Allelopathic effect of *Cylindrospermopsis raciborskii* extracts on the germination and growth of several plant species. *Chemistry and Ecology*, 26(4), 263-271 *

Sivonen, K., Himberg, K., Luukkainen, R., Niemelä, S.I., Poon, G.K. & Codd, G.A. (1989) Preliminary characterization of neurotoxic cyanobacteria blooms and strains from Finland. *Environmental Toxicology*, 4(3), 339-352 *

Stoichev, T., Basto, M.C.P., Vasconcelos, V. & Vasconcelos, M.T.S. (2010) Fate and effects of nonylphenol in the presence of the cyanobacterium *Microcystis aeruginosa*. *Chemistry and Ecology*, 26(5), 395-399 *

Stoichev, T., Baptista, M.S., Basto, M.C.P., Vasconcelos, V.M. & Vasconcelos, M.T.S. (2011) Effects of minocycline and its degradation products on the growth of *Microcystis aeruginosa*. *Ecotoxicology and Environmental Safety*, 74(3), 219-224 *

Stucken, K., Murillo, A.A., Soto-Liebe, K., Fuentes-Valdés, J.J., Méndez, M.A. & Vásquez, M. (2009) Toxicity phenotype does not correlate with phylogeny of *Cylindrospermopsis raciborskii* strains. *Systematic and Applied Microbiology*, 32(1), 37-48 *

Vasconcelos, V.M. (1995) Uptake and depuration of the heptapeptide toxin microcystin-LR in *Mytilus galloprovincialis*. *Aquatic Toxicology*, 32(2), 227-237 *

Vasconcelos, V., Oliveira, S. & Teles, F.O. (2001) Impact of a toxic and a non-toxic strain of *Microcystis aeruginosa* on the crayfish *Procambarus clarkii*. *Toxicon*, 39(10), 1461-1470 *

Vasconcelos, R. (2013) BMAA toxicological assessment using marine invertebrates as targets, MSc Dissertation, ICBAS, University of Porto, Porto, Portugal *

Vasconcelos, V.M. (1995) Toxicologia de cianobactérias: Distribuição de cianobactérias tóxicas e suas toxinas em águas doces portuguesas. Bioacumulação em bivalves, PhD Thesis, FCUP, University of Porto, Porto, Portugal [in Portuguese] *

Vivas, J. (2013) N2 fixation by marine sponge associated cyanobacteria from the Portuguese coast, MSc Dissertation, FCUP, University of Porto, Porto, Portugal

Ziemert, N., Ishida, K., Weiz, A., Hertweck, C. & Dittmann, E. (2010) Exploiting the natural diversity of microviridin gene clusters for discovery of novel tricyclic depsipeptides. *Applied and Environmental Microbiology*, 76(11), 3568-3574 *

Ziemert, N. (2009) Identification and characterisation of ribosomal biosynthesis pathways of two cyclic peptides from cyanobacteria, PhD Thesis, Faculty of Mathematics and Natural Sciences, Humboldt University of Berlin, Berlin, Germany *

Zimba, P.V., Huang, I., Foley, J.E. & Linton, E.W. (2016) Identification of a new-to-science cyanobacterium, *Toxifilum mysidocida* gen. nov. & sp. nov. (Cyanobacteria, Cyanophyceae). *Journal of Phycology*, 53(1), 188-197

Index of strains (alphabetical order)

Order	Strain ID	Catalog Page
**Chroococcales**		
	Cyanobacterium stanieri LEGE 03274	19
	Geminobacterium atlanticum LEGE 07459	91
	Gloeocapsa-like sp. LEGE 10406	92
	Gloeocapsopsis sp. LEGE 13414	94
	Microcystis aeruginosa LEGE 00063	118
	Microcystis aeruginosa LEGE 00239	119
	Microcystis aeruginosa LEGE 05195	120
	Microcystis aeruginosa LEGE 08327	121
	Microcystis aeruginosa LEGE 08328	122
	Microcystis aeruginosa LEGE 08329	123
	Microcystis aeruginosa LEGE 08330	124
	Microcystis aeruginosa LEGE 08331	125
	Microcystis aeruginosa LEGE 08354	126
	Microcystis aeruginosa LEGE 11464	127
	Microcystis aeruginosa LEGE 11465	128
	Microcystis aeruginosa LEGE 12460	129
	Microcystis aeruginosa LEGE 12461	130
	Microcystis aeruginosa LEGE 12462	131
	Microcystis aeruginosa LEGE 12463	132
	Microcystis aeruginosa LEGE 91093	133
	Microcystis aeruginosa LEGE 91094	134
	Microcystis aeruginosa LEGE 91095	135
	Microcystis aeruginosa LEGE 91096	136
	Microcystis aeruginosa LEGE 91338	137
	Microcystis aeruginosa LEGE 91339	138
	Microcystis aeruginosa LEGE 91341	139
	Microcystis aeruginosa LEGE 91342	140
	Microcystis aeruginosa LEGE 91343	141
	Microcystis aeruginosa LEGE 91344	142

Microcystis aeruginosa LEGE 91347 143
Microcystis aeruginosa LEGE 91349 144
Microcystis aeruginosa LEGE 91350 145
Microcystis aeruginosa LEGE 91351 146
Microcystis aeruginosa LEGE 91352 147
Microcystis aeruginosa LEGE 91353 148
Microcystis aeruginosa LEGE XX359 149
Microcystis sp. LEGE 00066 150
Microcystis sp. LEGE 00258 151
Microcystis sp. LEGE 08355 152
Microcystis sp. LEGE XX408 153
Microcystis wesenbergii LEGE 08368 154
Synechocystis salina LEGE 00027 256
Synechocystis salina LEGE 00028 257
Synechocystis salina LEGE 00029 258
Synechocystis salina LEGE 00030 259
Synechocystis salina LEGE 00031 260
Synechocystis salina LEGE 00032 261
Synechocystis salina LEGE 00036 262
Synechocystis salina LEGE 00037 263
Synechocystis salina LEGE 00038 264
Synechocystis salina LEGE 00040 265
Synechocystis salina LEGE 00041 266
Synechocystis salina LEGE 06099 267
Synechocystis salina LEGE 06155 268
Synechocystis sp. LEGE 06005 269
Synechocystis sp. LEGE 06017 270
Synechocystis sp. LEGE 06025 271
Synechocystis sp. LEGE 06079 272
Synechocystis sp. LEGE 06083 273
Synechocystis sp. LEGE 07073 274
Synechocystis sp. LEGE 07211 275
Synechocystis sp. LEGE 07367 276
unidentified filamentous Chroococcales LEGE 11427 341

	unidentified filamentous Chroococcales LEGE 11438	342
	unidentified unicellular Chroococcales LEGE 06019	378
	unidentified unicellular Chroococcales LEGE 11426	379
**Chroococcidiopsidales**		
	Gloeocapsopsis crepidinum LEGE 06123	93
	Gloeocapsopsis sp. LEGE 13420	95
	Gloeocapsopsis sp. LEGE 13421	96
	unidentified colonial Chroococcidiopsidales LEGE 13417	335
	unidentified colonial Chroococcidiopsidales LEGE 13419	336
	unidentified colonial Chroococcidiopsidales LEGE 13423	337
**Nostocales**		
	Anabaena aphanizomenoides LEGE 00250	3
	Anabaena cf. *cylindrica* LEGE 00235	4
	Anabaena sp. LEGE X-002	5
	Calothrix sp. LEGE 06100	7
	Calothrix sp. LEGE 06122	8
	Chrysosporum ovalisporum LEGE X-001	12
	Cuspidothrix issatschenkoi LEGE 00247	15
	Cuspidothrix issatschenkoi LEGE 03282	16
	Cuspidothrix issatschenkoi LEGE 03284	17
	Cuspidothrix issatschenkoi LEGE 03285	18
	Cylindrospermopsis raciborskii LEGE 95046	68
	Cylindrospermopsis raciborskii LEGE 97047	69
	Cylindrospermopsis raciborskii LEGE 99043	70
	Cylindrospermopsis raciborskii LEGE 99044	71
	Cylindrospermopsis raciborskii LEGE 99045	72
	Cylindrospermopsis raciborskii LEGE 99048	73
	Desmonostoc muscorum LEGE 12446	74
	Dolichospermum flosaquae LEGE 02268	75
	Dolichospermum flosaquae LEGE 04289	76
	Dolichospermum sp. LEGE 00234	77
	Dolichospermum sp. LEGE 00240	78
	Dolichospermum sp. LEGE 00241	79
	Dolichospermum sp. LEGE 00246	80

Dolichospermum sp. LEGE 00248 81
Dolichospermum sp. LEGE 00259 82
Dolichospermum sp. LEGE 00263 83
Dolichospermum sp. LEGE 03277 84
Dolichospermum sp. LEGE 03278 85
Fortiea sp. LEGE XX443 86
Nodularia sp. LEGE 04288 198
Nodularia sp. LEGE 06071 199
Nostoc aff. *commune* LEGE 04357 200
Nostoc cf. *edaphicum* LEGE 07299 201
Nostoc sp. LEGE 06077 202
Nostoc sp. LEGE 06158 203
Nostoc sp. LEGE 07365 204
Nostoc sp. LEGE 12447 205
Nostoc sp. LEGE 12448 206
Nostoc sp. LEGE 12449 207
Nostoc sp. LEGE 12450 208
Nostoc sp. LEGE 12451 209
Nostoc sp. LEGE 12453 210
Nostoc sp. LEGE 12454 211
Nostoc sp. LEGE 12456 212
Nostoc sp. LEGE 13413 213
Plectonema cf. *radiosum* LEGE 06105 227
Plectonema cf. *radiosum* LEGE 06114 228
Rivularia sp. LEGE 07159 232
Rivularia sp. LEGE 07177 233
Rivularia sp. LEGE 07178 234
aff. *Roholtiella* sp. LEGE 12411 306
Scytonema sp. LEGE 07182 237
Scytonema sp. LEGE 07189 238
Sphaerospermopsis sp. LEGE 00249 239
Sphaerospermopsis sp. LEGE 02266 240
Sphaerospermopsis sp. LEGE 08334 241
Sphaerospermopsis sp. LEGE 08335 242

	Tolypothrix sp. LEGE 11397	277
	unidentified Nostocales LEGE 03272	318
	unidentified Nostocales LEGE 06106	319
	unidentified Nostocales LEGE 06150	320
	unidentified Nostocales LEGE 11386	321
	unidentified Nostocales LEGE 12452	322
	unidentified Nostocales LEGE 12455	323
	unidentified Nostocales LEGE 14445	324
	unidentified Nostocales LEGE XX254	325
	unidentified Nostocales LEGE XX276	326
**Oscillatoriales**		
	Coleofasciculus sp. LEGE 07081	13
	Coleofasciculus sp. LEGE 07092	14
	Limnoraphis robusta LEGE XX358	111
	Lusitaniella coriacea LEGE 06111	113
	Lusitaniella coriacea LEGE 07157	114
	Lusitaniella coriacea LEGE 07167	115
	Lusitaniella coriacea LEGE 07180	116
	Microcoleus sp. LEGE 07076	117
	cf. *Oxynema acuminatum* LEGE 06072	308
	cf. *Oxynema acuminatum* LEGE 06078	309
	Phormidium laetevirens LEGE 06103	215
	Phormidium sp. LEGE 00064	216
	Phormidium sp. LEGE 00065	217
	Phormidium cf. *irriguum* LEGE 00055	214
	Phormidium sp. LEGE 05292	218
	Planktothrix mougeotii LEGE 06222	219
	Planktothrix mougeotii LEGE 06223	220
	aff. *Planktothrix mougeotii* LEGE 06224	304
	Planktothrix mougeotii LEGE 06225	221
	Planktothrix mougeotii LEGE 06226	222
	aff. *Planktothrix mougeotii* LEGE 07227	305
	Planktothrix mougeotii LEGE 07229	223
	Planktothrix mougeotii LEGE 07230	224

	Planktothrix mougeotii LEGE 07231	225
	Planktothrix sp. LEGE XX280	226
	Spirulina sp. LEGE 11439	243
	Tychonema bornetii LEGE 14444	279
	Tychonema sp. LEGE 06204	280
	Tychonema sp. LEGE 06205	281
	Tychonema sp. LEGE 06206	282
	Tychonema sp. LEGE 06207	283
	Tychonema sp. LEGE 06208	284
	Tychonema sp. LEGE 06220	285
	Tychonema sp. LEGE 06363	286
	Tychonema sp. LEGE 07196	287
	Tychonema sp. LEGE 07197	288
	Tychonema sp. LEGE 07198	289
	Tychonema sp. LEGE 07199	290
	Tychonema sp. LEGE 07200	291
	Tychonema sp. LEGE 07202	292
	Tychonema sp. LEGE 07203	293
	Tychonema sp. LEGE 07213	294
	Tychonema sp. LEGE 07214	295
	Tychonema sp. LEGE 07215	296
	Tychonema sp. LEGE 07216	297
	Tychonema sp. LEGE 07217	298
	Tychonema sp. LEGE 07221	299
	unidentified Oscillatoriales LEGE 00049	327
	unidentified Oscillatoriales LEGE 11385	328
	unidentified Oscillatoriales LEGE 11467	329
	unidentified Oscillatoriales LEGE 11472	330
**Pleurocapsales**		
	Chroococcidiopsis sp. LEGE 06174	9
	Chroococcopsis sp. LEGE 07168	10
	Chroococcopsis sp. LEGE 07187	11
	Hyella patelloides LEGE 07179	98
	Myxosarcina sp. LEGE 06146	155

	unidentified Pleurocapsales LEGE 06147	331
	unidentified Pleurocapsales LEGE 10410	332
**Synechococcales**		
	Alkalinema aff. p*antanalense* LEGE 15481	2
	Calenema singularis LEGE 06188	6
	Cyanobium aff. *gracile* LEGE 07366	20
	Cyanobium gracile LEGE 00054	21
	Cyanobium gracile LEGE 09399	22
	Cyanobium gracile LEGE 12431	23
	Cyanobium sp. LEGE 00034	24
	Cyanobium sp. LEGE 00035	25
	Cyanobium sp. LEGE 06002	26
	Cyanobium sp. LEGE 06004	27
	Cyanobium sp. LEGE 06008	28
	Cyanobium sp. LEGE 06011	29
	Cyanobium sp. LEGE 06012	30
	Cyanobium sp. LEGE 06015	31
	Cyanobium sp. LEGE 06016	32
	Cyanobium sp. LEGE 06023	33
	Cyanobium sp. LEGE 06024	34
	Cyanobium sp. LEGE 06026	35
	Cyanobium sp. LEGE 06068	36
	Cyanobium sp. LEGE 06097	37
	Cyanobium sp. LEGE 06098	38
	Cyanobium sp. LEGE 06109	39
	Cyanobium sp. LEGE 06113	40
	Cyanobium sp. LEGE 06127	41
	Cyanobium sp. LEGE 06130	42
	Cyanobium sp. LEGE 06134	43
	Cyanobium sp. LEGE 06135	44
	Cyanobium sp. LEGE 06137	45
	Cyanobium sp. LEGE 06138	46
	Cyanobium sp. LEGE 06139	47
	Cyanobium sp. LEGE 06140	48

Cyanobium sp. LEGE 06142 49
Cyanobium sp. LEGE 06143 50
Cyanobium sp. LEGE 06184 51
Cyanobium sp. LEGE 07153 52
Cyanobium sp. LEGE 07173 53
Cyanobium sp. LEGE 07175 54
Cyanobium sp. LEGE 07183 55
Cyanobium sp. LEGE 07186 56
Cyanobium sp. LEGE 07293 57
Cyanobium sp. LEGE 07313 58
Cyanobium sp. LEGE 07318 59
Cyanobium sp. LEGE 10374 60
Cyanobium sp. LEGE 10375 61
Cyanobium sp. LEGE 11435 62
Cyanobium sp. LEGE 11436 63
Cyanobium sp. LEGE 11437 64
Cyanobium sp. LEGE 11470 65
Cyanobium sp. LEGE 11482 66
Cyanobium sp. LEGE XX442 67
Geitlerinema sp. LEGE 11390 87
Geitlerinema sp. LEGE 11391 88
Geitlerinema sp. LEGE 11393 89
Geitlerinema sp. LEGE 11396 90
Halomicronema cf. *metazoicum* LEGE 07132 97
Jaaginema litorale LEGE 07176 99
Leptolyngbya cf. *ectocarpi* LEGE 11389 100
Leptolyngbya ectocarpi LEGE 11425 102
aff. *Leptolyngbya ectocarpi* LEGE 11473 300
Leptolyngbya ectocarpi LEGE 11474 103
aff. *Leptolyngbya ectocarpi* LEGE 11478 301
Leptolyngbya cf. *ectocarpi* LEGE 11479 101
Leptolyngbya minuta LEGE 07128 104
Leptolyngbya saxicola LEGE 06131 105
Leptolyngbya sp. LEGE 06117 106

Leptolyngbya sp. LEGE 06361 107
Leptolyngbya sp. LEGE 07154 108
Leptolyngbya-like sp. LEGE 13412 110
Leptolyngbya sp. LEGE 13418 109
Limnothrix sp. LEGE 00237 112
Nodosilinea (*Leptolyngby*a) *antarctica* LEGE 13457 156
Nodosilinea (Leptolyngbya) antarctica LEGE 13458 157
Nodosilinea cf. *nodulosa* LEGE 10377 158
Nodosilinea nodulosa LEGE 06101 159
Nodosilinea nodulosa LEGE 06102 160
Nodosilinea nodulosa LEGE 06104 161
Nodosilinea nodulosa LEGE 07084 162
Nodosilinea sp. LEGE 03283 163
Nodosilinea sp. LEGE 06001 164
Nodosilinea sp. LEGE 06006 165
Nodosilinea sp. LEGE 06009 166
Nodosilinea sp. LEGE 06010 167
Nodosilinea sp. LEGE 06014 168
Nodosilinea sp. LEGE 06020 169
Nodosilinea sp. LEGE 06022 170
Nodosilinea sp. LEGE 06069 171
Nodosilinea sp. LEGE 06110 172
Nodosilinea sp. LEGE 06115 173
Nodosilinea sp. LEGE 06119 174
Nodosilinea sp. LEGE 06120 175
Nodosilinea sp. LEGE 06121 176
Nodosilinea sp. LEGE 06124 177
Nodosilinea sp. LEGE 06129 178
Nodosilinea sp. LEGE 06133 179
Nodosilinea sp. LEGE 06145 180
aff. *Nodosilinea* sp. LEGE 06148 302
aff. *Nodosilinea* sp. LEGE 06149.2 303
Nodosilinea sp. LEGE 06152 181
Nodosilinea sp. LEGE 06191 182

Nodosilinea sp. LEGE 06193	183
Nodosilinea sp. LEGE 06308	184
Nodosilinea sp. LEGE 07080	185
Nodosilinea sp. LEGE 07085	186
Nodosilinea sp. LEGE 07088	187
Nodosilinea sp. LEGE 07091	188
Nodosilinea sp. LEGE 07298	189
Nodosilinea sp. LEGE 07309	190
Nodosilinea sp. LEGE 07311	191
Nodosilinea sp. LEGE 07314	192
Nodosilinea sp. LEGE 07364	193
Nodosilinea sp. LEGE 10376	194
Nodosilinea-like sp. LEGE 11424	197
Nodosilinea sp. LEGE 11429	195
Nodosilinea sp. LEGE 11468	196
cf. *Oculatella* sp. LEGE 06141	307
cf. *Phormidesmis* sp. LEGE 10370	310
cf. *Phormidesmis* sp. LEGE 11430	311
cf. *Phormidesmis* sp. LEGE 11434	312
cf. *Phormidesmis* sp. LEGE 11475	313
cf. *Phormidesmis* sp. LEGE 11477	314
Pseudanabaena aff. *mucicola* LEGE 00260	229
Pseudanabaena cf. *curta* LEGE 07169	230
Pseudanabaena cf. *curta* LEGE 10371	231
cf. *Romeria* sp. LEGE 06003	315
cf. *Romeria* sp. LEGE 06013	316
cf. *Romeria* sp. LEGE 11469	317
Romeria aff. *gracilis* LEGE 07310	235
Schizothrix aff. *septentrionalis* LEGE 07164	236
Synechococcus cf. *nidulans* LEGE 06322	244
Synechococcus nidulans LEGE 06156	245
Synechococcus nidulans LEGE 07171	246
Synechococcus sp. LEGE 06306	247
Synechococcus sp. LEGE 06324	248

Synechococcus sp. LEGE 07172 249
Synechococcus sp. LEGE 11379 250
Synechococcus sp. LEGE 11381 251
Synechococcus sp. LEGE 11394 252
Synechococcus sp. LEGE 11428 253
Synechococcus sp. LEGE 11466 253
Synechococcus sp. LEGE 11471 255
Toxifilum mysidocida LEGE 06108 278
unidentified colonial Synechococcales LEGE 06192 338
unidentified colonial Synechococcales LEGE 10388 339
unidentified colonial Synechococcales LEGE 10400 340
unidentified filamentous Synechococcales LEGE 00039 343
unidentified filamentous Synechococcales LEGE 00052 344
unidentified filamentous Synechococcales LEGE 00053 345
unidentified filamentous Synechococcales LEGE 06018 346
unidentified filamentous Synechococcales LEGE 06021 347
unidentified filamentous Synechococcales LEGE 06070 348
unidentified filamentous Synechococcales LEGE 06116 349
unidentified filamentous Synechococcales LEGE 06118 350
unidentified filamentous Synechococcales LEGE 06144 351
unidentified filamentous Synechococcales LEGE 07075 352
unidentified filamentous Synechococcales LEGE 07089 353
unidentified filamentous Synechococcales LEGE 07160 354
unidentified filamentous Synechococcales LEGE 07163 355
unidentified filamentous Synechococcales LEGE 07185 356
unidentified filamentous Synechococcales LEGE 07190 357
unidentified filamentous Synechococcales LEGE 07319 358
unidentified filamentous Synechococcales LEGE 08404 359
unidentified filamentous Synechococcales LEGE 10405 360
unidentified filamentous Synechococcales LEGE 11384 361
unidentified filamentous Synechococcales LEGE 11392 362
unidentified filamentous Synechococcales LEGE 11395 363
unidentified filamentous Synechococcales LEGE 12432 364
unidentified filamentous Synechococcales LEGE 13422 365

unidentified Pseudanabaenaceae LEGE 06112 333
unidentified Pseudanabaenaceae LEGE 13415 334
unidentified unicellular Synechococcales LEGE 06307 380
unidentified unicellular Synechococcales LEGE 06316 381
unidentified unicellular Synechococcales LEGE 07074 382
unidentified unicellular Synechococcales LEGE 08333 383
unidentified unicellular Synechococcales LEGE 09398 384
unidentified unicellular Synechococcales LEGE 10387 385

Unclear Taxa

unidentified filamentous cyanobacterium LEGE 00033 366
unidentified filamentous cyanobacterium LEGE 00060 367
unidentified filamentous cyanobacterium LEGE 06007 368
unidentified filamentous cyanobacterium LEGE 07170 369
unidentified filamentous cyanobacterium LEGE 07209 370
unidentified filamentous cyanobacterium LEGE 07212 371
unidentified filamentous cyanobacterium LEGE 11480 372
unidentified filamentous cyanobacterium LEGE 12433 373
unidentified filamentous cyanobacterium LEGE 12440 374
unidentified filamentous cyanobacterium LEGE 12441 375
unidentified filamentous cyanobacterium LEGE XX061 376
unidentified filamentous cyanobacterium LEGE XX062 377
